# Differential DNA methylation and expression of inflammatory and zinc transporter genes defines subgroups of osteoarthritic hip patients

**DOI:** 10.1136/annrheumdis-2014-206752

**Published:** 2015-04-08

**Authors:** Michael D Rushton, David A Young, John Loughlin, Louise N Reynard

**Affiliations:** Musculoskeletal Research Group, Institute of Cellular Medicine, Newcastle University, Newcastle upon Tyne, UK

**Keywords:** Inflammation, Osteoarthritis, Chondrocytes

## Abstract

**Objectives:**

We have previously shown that the cartilage DNA methylome delineates two clusters of osteoarthritic (OA) hip patients, characterised by differential methylation of inflammatory genes, while others have demonstrated a link between zinc homeostasis and inflammation in OA. We aimed to investigate these effects at the methylation and gene expression level.

**Methods:**

We used our previously generated methylation data while quantitative PCR was used to measure gene expression using RNA from the hip cartilage of members of both clusters and from control individuals without hip OA.

**Results:**

One of the OA clusters is characterised by the promoter hypomethylation and increased expression of inflammation-associated genes including IL1A and TNF. Furthermore, we show that the increase in expression of these genes is accompanied by increased expression of several zinc transporter genes. In addition, the zinc responsive transcription factor MTF1 is also upregulated, which is accompanied by an increase in the expression of its targets the metalloproteinases MMP13 and ADAMTS5.

**Conclusions:**

We have identified a subgroup of OA hip patients that are epigenetically and transcriptiomically characterised by a cartilage inflammatory phenotype with concurrent differential regulation of zinc regulators. The identification of subgroups enhances stratified phenotyping of OA patients and has important implications for future therapeutic applications.

## Introduction

Osteoarthritis is characterised by the focal loss of articular cartilage at the synovial joints.^1^ ^2^ Genetic susceptibility has an established role in osteoarthritic (OA) aetiology, while recent studies have highlighted the crucial impact of epigenetics, specifically DNA CpG methylation, on the disease.^3^ Initial methylation studies employed a candidate gene approach, whereas more recent studies have used powerful genome-wide DNA CpG methylation arrays to reveal the unique landscape of the OA cartilage DNA methylome.^3–6^ These have reported widespread cartilage methylation changes between OA and non-OA individuals. Our previous study revealed subgroups, or clusters, of OA individuals based on their DNA methylome.^5^ For the 23 OA hip patients examined in that study, two very distinct clusters were observed based on the highly significant differential methylation of inflammation/immune-related genes.

Although OA has historically been classified as non-inflammatory, it is now becoming clear that inflammation plays a role in some patients.^7^ Much of the attention on OA inflammation has focused on the synovium, with the synovial fluid containing elevated levels of proinflammatory factors.^8^ Some studies have suggested a possible role for the chondrocyte in inflammation,^9^ with OA theorised to result from an unresolved chronic wound healing response in cartilage that involves the activation of inflammatory cytokines.^10^ ^11^ In vitro studies have also shown that chondrocytes upregulate matrix-degrading enzymes in response to proinflammatory factors such as IL-1β, thought to be mediated at least in part through increased activity of the zinc–ZIP8–MTF1 axis.^11^ ^12^ This highlights that zinc homeostasis is also a key player in OA pathogenesis, linking inflammation with cartilage loss.

In our previous study, the 23 OA hip patients grouped into clusters 1 and 2 containing 11 and 12 patients, respectively, with cluster 2 demonstrating hypomethylation of the inflammation/immune-related genes.^5^ In that report, we had also studied 21 patients who were free of hip OA and had undergone hip replacement due to a neck of femur (NOF) fracture. These were more similar to OA cluster 1 with respect to the methylation of the inflammation/immune-related genes. In the present study, we investigated the two OA hip clusters and the NOF patients in more detail at the DNA methylation and gene expression level, with a focus on immune-related genes and zinc homeostasis.

## Materials and methods

Patient information is shown in online supplementary table S1, and a list of the primers used is shown in online supplementary table S2. A detailed description of the material and methods can be found in the online supplementary text.

## Results

### Inflammatory/immunity genes are demethylated in a subset of OA hip samples

We used our previously generated methylation data to search for sites that were significantly differentially methylated between OA hip cluster 2 (12 patients) and both OA hip cluster 1 (11 patients) and the 21 NOF patients. In total, we identified 10 064 differentially methylated CpG loci, of which 4308 were hypomethylated and 5756 were hypermethylated in OA hip cluster 2 compared with both NOF and OA hip cluster 1 (see online supplementary tables S3 and S4). We performed gene ontology term analysis of both hypermethylated and hypomethylated genes (see online supplementary tables S5 and S6). Analysis of the hypomethylated genes revealed a striking enrichment of terms related to inflammation and immunity (table 1), matching our previous results where we had compared just the two OA hip clusters.^5^

**Table 1:** Example of inflammation/immune-related gene ontology (GO) terms for genes hypomethylated in osteoarthritic hip cluster 2

GO term	Number of genes	Bonferroni p value
Immune system process	198	1.28×10^−19^
Immune response	306	5.76×10^−15^
Defence response	128	2.1×10^−11^
Regulation of immune response	83	1.87×10^−09^
Innate immune response	87	3.78×10^−09^
Activation of immune response	45	1.04×10^−05^
Immune system development	62	1.18×10^−05^
Lymphocyte activation	32	3.41×10^−04^
Regulation of leucocyte activation	43	5.58×10^−04^
Positive regulation of T cell activation	27	5.91×10^−04^
Regulation of defence response	52	1.06×10^−03^
Regulation of IL-1β production	11	2.35×10^−03^
Regulation of cytokine production	48	3.14×10^−03^
Positive regulation of leucocyte-mediated immunity	15	1.01×10^−02^
Regulation of T cell activation	30	1.11×10^−02^
Leucocyte differentiation	21	1.13×10^−02^
T cell co-stimulation	14	1.26×10^−02^
Response to interferon-gamma	18	1.44×10^−02^
Lymphocyte co-stimulation	14	1.48×10^−02^
Regulation of lymphocyte-mediated immunity	16	2.69×10^−02^

### Analysis of the methylation of the promoters of inflammation-associated genes

**Figure 1:**
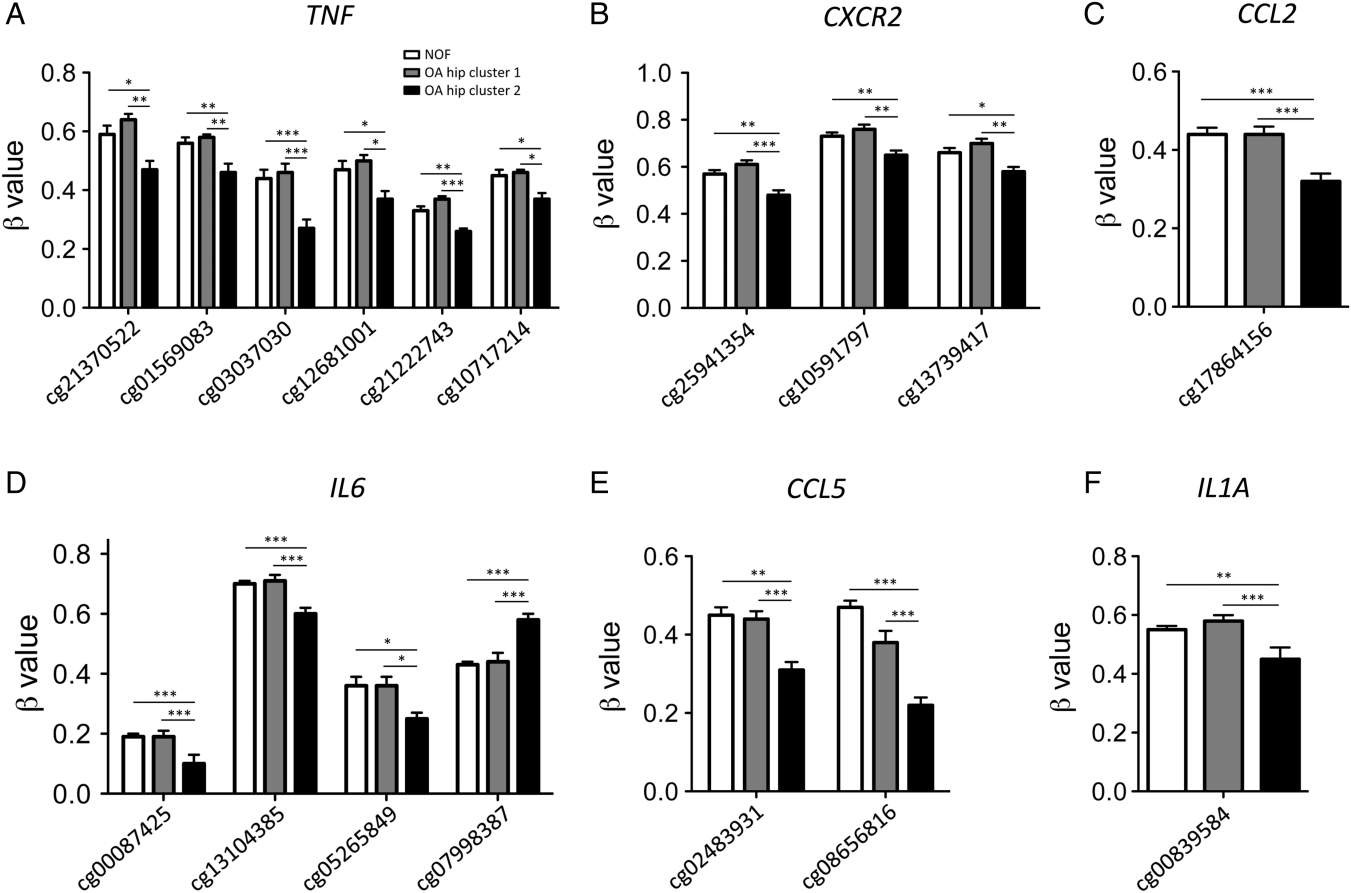
The promoters of inflammatory genes are demethylated in a subgroup of osteoarthritic (OA) hip patients. The level of methylation is shown as the β value obtained from the HumanMethylation450 BeadChip array for a total of 21 neck of femur (NOF) patients, 11 OA hip cluster 1 patients and 12 OA hip cluster 2 patients. The methylation levels are shown for six inflammation-associated genes; (A) *TNF*; (B) *CXCR2*; (C) *CCL2*; (D) *IL6*; (E) *CCL5*; (F) *IL1A*. Data are shown as the mean and the standard error of the mean and statistical analysis was performed by one-way analysis of variance with the Tukey test. ***p<0.001, **p<0.01, *p<0.05.

**Figure 2:**
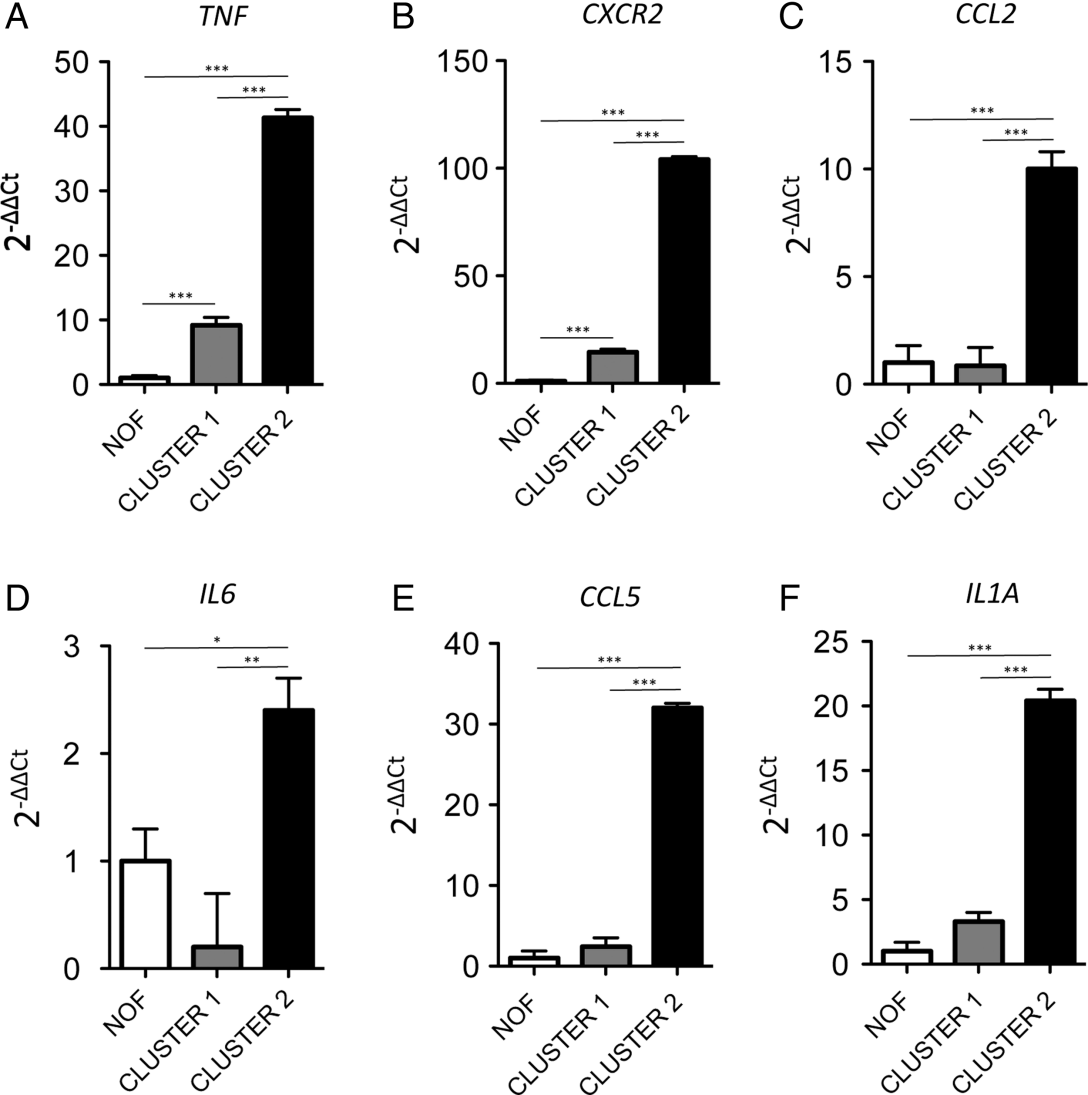
Demethylation of the promoter is accompanied by an increase in the expression of inflammation-associated genes. Gene expression was measured in five neck of femur (NOF), five osteoarthritic (OA) hip cluster 1 and six OA hip cluster 2 patients who had all been included in the original methylation analysis. Data are presented as 2^−ΔΔCt^ with the NOF data serving as the control group and are shown as the mean and the standard error of the mean. Statistical analysis was performed by one-way analysis of variance with the Tukey test. ***p<0.001, **p<0.01, *p<0.05. Gene expression was measured in the six previously identified differentially methylated genes; (A) *TNF*; (B) *CXCR2*; (C) *CCL2*; (D) *IL6*; (E) *CCL5*; (F) *IL1A*.

Hypomethylation of the inflammation-associated genes predominantly occurred within their promoters. We therefore analysed the methylation status of the promoters of a group of six inflammatory-related genes, selected on the basis that they contained at least one differentially methylated probe from our genome-wide analysis. In this analysis, we also included probes that were genome-wide significant irrespective of the difference in methylation. The genes selected coded for factors known to have a role in OA and were *TNF*, *IL6*, *IL1A*, the IL8 receptor *CXCR2* and the chemokines *CCL5* and CCL2. Information regarding the CpG probes studied for each gene can be found in online supplementary table S7 (the CpG probes listed as residing in the gene body are in regions defined by ENCODE (http://genome.ucsc.edu/ENCODE/) as having promoter activity). Figure 1 shows that in OA hip cluster 2 these inflammatory-associated genes undergo promoter demethylation in comparison to both OA hip cluster 1 and NOF, the only exception being one of the four *IL6* probes (cg07998387). Promoter demethylation was accompanied by an increase in expression of all six genes in OA hip cluster 2 (figure 2). The correlation between DNA methylation and gene expression is shown in online supplementary figures S1 and S2, and table S8.

### Regulation of zinc transporters in the inflammation-associated group of individuals

**Figure 3:**
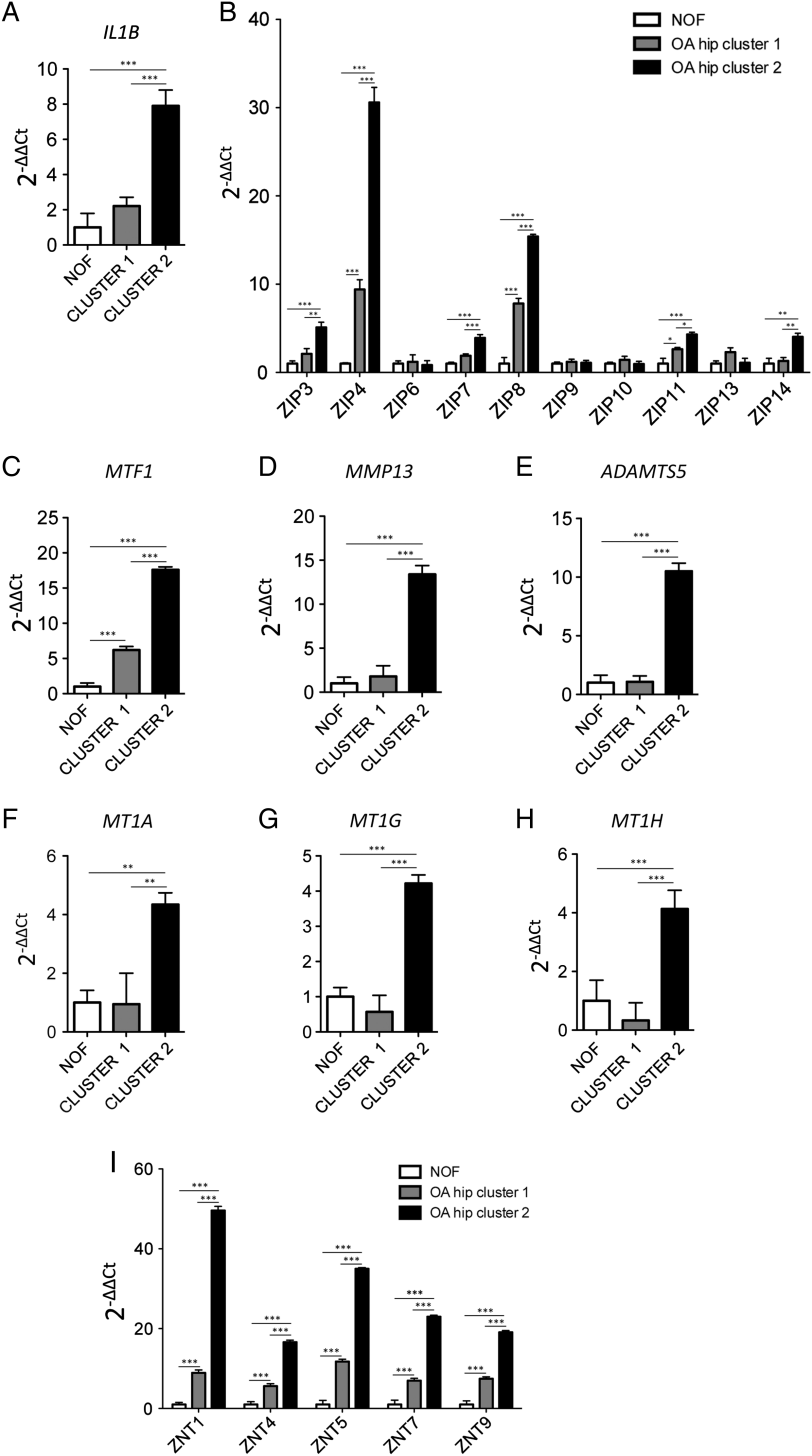
The *ZIP* group of zinc transporter genes are upregulated in the inflammation-associated subgroup of osteoarthritic (OA) hip patients. Gene expression was measured in five neck of femur (NOF), five OA hip cluster 1 and six OA hip cluster 2 patients. Data are presented as 2^−ΔΔCt^ with the NOF data serving as the control group and are shown as the mean and the standard error of the mean. Statistical analysis was performed by one-way analysis of variance with the Tukey test. ***p<0.001, **p<0.01, *p<0.05. (A) Analysis of *IL1B* expression. (B) Analysis of the ZIP group of zinc transporter genes. (C) Gene expression analysis of the zinc transporter gene regulator *MTF1*. Gene expression analysis of the MTF1 targets (D) *MMP13* and (E) *ADAMTS5*. Gene expression analysis of the metallothioneins (F) *MT1A*; (G) *MT1G* and (H) *MT1H*. (I) Gene expression analysis of the ZnT group of zinc transporter genes.

It has been shown that elevated intracellular zinc levels are involved in the upregulation of metalloproteinases in OA.^12^ Intriguingly, inflammatory factors such as IL1 are known to regulate the ZIP group of zinc transporters, including in mouse articular chondrocytes.^13^ We initially observed that the zinc transporter genes *ZIP4* (*SLC39A4*), *ZIP7* (*SLC39A7*), *ZIP11* (*SLC39A11*) and *ZIP14* (*SLC39A14*) were differentially methylated between our two OA hip clusters (see online supplementary figure S3 and table S9). We therefore investigated whether the zinc transporter genes were differentially expressed between the clusters. In the recent report highlighting the role of zinc homeostasis in OA, IL-1β was revealed to be a pivotal regulator of *ZIP* genes.^12^ ^13^ We therefore specifically investigated its gene in this context and observed that *IL1B* is upregulated in OA hip cluster 2 (figure 3A). The expression of all 14 zinc importers (*ZIP1*-*14; SLC39A1-14*) was then assessed. *ZIP1*, *ZIP2*, *ZIP5* and *ZIP12* were not expressed in hip cartilage (data not shown) but 6 of the 10 importers that are expressed are upregulated in cluster 2, with expression correlating positively with methylation (see figure 3B, online supplementary figure S4 and table S10). Further to this, the expression of the six *ZIP* genes correlated positively with the level of expression of proinflammatory genes and these correlations were consistently significant for *IL1A* and *TNF* (see online supplementary figures 5 and 6 and table S11). Furthermore, we observed upregulation in cluster 2 of *MT1A*, *MT1G* and *MT1H*, which code for zinc binding metallothioneins that control intracellular zinc levels (figure 3F–H). In addition, several of the ZnT zinc exporter group of genes are also upregulated in cluster 2 (figure 3I). This may be as a consequence of increased intracellular levels of Zn^2+^ and/or result from the increased expression of the zinc transporter gene regulator *MTF1* that we observed (figure 3C). Finally, *ADAMTS5* and *MMP13*, two downstream targets of *MTF1*, are also upregulated in cluster 2. (figure 3D, E)

## Discussion

In this study, we report on a clear subset of OA hip patients who undergo demethylation of the promoters of inflammatory/immunity-related genes. Demethylation is accompanied by increased expression of inflammatory factors, suggesting that cartilage inflammation may play a more integral role in the disease pathogenesis in this group of individuals. We also show that the increase in the expression of inflammatory genes correlates with the expression of zinc transporter and metalloproteinase genes. Our observations resonate with other complex diseases, where patients can present with the same disease phenotype but the underling genetic/epigenetic status differs.^14–16^

Proinflammatory cytokines such as IL6, IL1 and TNF-α are known to influence zinc homeostasis through regulating the expression of zinc transporter genes.^17^ ^18^ Intriguingly, it has been shown recently that *ZIP8* upregulation induces the expression of matrix-degrading enzymes in chondrocytes.^12^ This has led to the suggestion that zinc regulation could offer therapeutic opportunities in OA. Our data support this but very clearly highlight that this may be particularly applicable to a subgroup of OA individuals; *ZIP8* expression demonstrated very significant differential expression between the two clusters.

While we see differential methylation of several ZIP genes, it is not clear whether it is this or whether it is the increase in the expression of the inflammatory factors that is driving this increase. It may be that the inflammatory genes regulate *ZIP* gene expression via DNA methylation.

The expression correlations that we have observed between inflammatory and zinc genes are very noteworthy, but clearly the expression of other genes may also correlate. A more comprehensive analysis is therefore called for.

In this study, we focused on the inflammatory subgroups that are present within OA hip samples. Subgroups have been observed in OA knee samples, but the clustering is much more heterogeneous than that in OA hip.^5^ For this reason, we focused our study on OA hip. However, we cannot rule out that the inflammatory and zinc gene effects reported in this study occur in OA knee. A similar study in OA knee is therefore warranted.

In conclusion, we show that the cartilage methylome identifies an inflammatory-associated subgroup of OA hip individuals with concurrent differential regulation of zinc regulators. The identification of subgroups of patients has important implications for the testing and application of disease-modifying OA therapeutics, with disease interventions needing to be tailored to the underlying genetic/epigenetic profile of an individual.

## Supplementary Material

Web supplement

Web figure

Web table 1

Web table 10

Web table 11

Web table 2

Web table 3

Web table 4

Web table 5

Web table 6

Web table 7

Web table 8

Web table 9
