# Supplementary material for: Differential DNA methylation and expression of inflammatory and zinc transporter genes defines subgroups of osteoarthritic hip patients
Source: Ann Rheum Dis. 2015 Apr 8;74(9):1778–82. doi: 10.1136/annrheumdis-2014-206752 (PMC4552898; doi:10.1136/annrheumdis-2014-206752)
Supplement: Web supplement [file annrheumdis-2014-206752-s1.pdf]

SUPPLEMENTARY MATERIAL AND METHODS

Patients

Patients were ascertained and their cartilage tissue was collected as described previously [1]. Pre-operative radiographs of the joints were graded for their OA status using the Kellgren-Lawrence (KL) scoring system. All OA patients had a KL score of at least 2 and were screened to exclude OA due to trauma of other pathologies. NOF patients had no radiographic signs of OA (KL score of 0 or 1), and the cartilage was macroscopically intact with no lesions. For all OA patients non-lesional cartilage was collected from sites distal to the OA lesion. The Newcastle and North Tyneside research ethics committee granted ethical approval for the collection and informed consent was obtained from each donor.

Methylation analysis

Methylation data was acquired from our previously published study that used the Infinium HumanMethylation450 BeadChip (Illumina) [1]. Processing and normalisation of the raw methylation data was performed in R (version 3.0.1) using the Watermelon package (version 2.12) as has been previously described [2]. The R package ComBat was used to correct for batch as well chip number [3]. Gene ontology (GO) analysis of hypo and hyper methylated loci was carried out through the Gene ontology Consortium (http://geneontology.org).

To identify differentially methylated loci (DMLs) the average β value was compared between the three groups of interest (NOF, OA hip cluster 1 and OA

hip cluster 2). P values were calculated using the Kruskal-Wallis test, as has been previously reported [4-7]. A locus was deemed significantly differentially methylated if there was at least a 10% difference in methylation (between OA hip cluster 2 and both OA hip cluster 1 and NOF) and a Benjamini-Hochberg p value <0.05. When focusing on loci within or close to transcribed genes we defined promoters as the first exon (including the 5'UTR) and sequences located up to 1.5 kb upstream of the transcription start site (TSS).

RNA extraction, cDNA synthesis and real-time PCR

RNA was isolated using the RNeasy kit (Qiagen) from 250 mg of ground cartilage tissue. 500 ng of the RNA was treated with two units of Turbo DNase (Ambion), before being reverse transcribed using the SuperScript First-Strand cDNA synthesis kit (Invitrogen). Gene expression was measured by real time PCR using SyBr green chemistry (see Supplementary Table 2 for primer sequences). Expression of target genes was measured relative to the housekeeping gene 18s rRNA, and the relative expression was calculated using the $2^{-\Delta\Delta Ct}$ method with the NOF cartilage data serving as the control group.

1. Rushton MD, Reynard LN, Barter MJ, et al. Characterization of the cartilage DNA methylome in knee and hip osteoarthritis. Arthritis & rheumatology 2014;**66**:2450-2460.
2. Touleimat N, Tost J. Complete pipeline for Infinium((R)) Human Methylation 450K BeadChip data processing using subset quantile normalization for accurate DNA methylation estimation. Epigenomics 2012;**4**(3):325-41.
3. Johnson WE, Li C, Rabinovic A. Adjusting batch effects in microarray expression data using empirical Bayes methods. Biostatistics 2007;**8**(1):118-27.
4. Conway K, Edmiston SN, May R, et al. DNA methylation profiling in the Carolina Breast Cancer Study defines cancer subclasses differing in

clinicopathologic characteristics and survival. Breast cancer research : BCR 2014;**16**(5):450.
5. Renner M, Wolf T, Meyer H, et al. Integrative DNA methylation and gene expression analysis in high-grade soft tissue sarcomas. Genome biology 2013;**14**(12):r137.
6. Rijlaarsdam MA, van der Zwan YG, Dorssers LC, et al. DMRforPairs: identifying differentially methylated regions between unique samples using array based methylation profiles. BMC bioinformatics 2014;**15**:141.
7. Assenov Y, Muller F, Lutsik P, et al. Comprehensive analysis of DNA methylation data with RnBeads. Nature methods 2014;**11**(11):1138-40.
