# Supplementary material for: Differential DNA methylation and expression of inflammatory and zinc transporter genes defines subgroups of osteoarthritic hip patients
Source: Ann Rheum Dis. 2015 Apr 8;74(9):1778–82. doi: 10.1136/annrheumdis-2014-206752 (PMC4552898; doi:10.1136/annrheumdis-2014-206752)

Supplementary Figure 1. Scatter plots showing the correlation between gene expression and promoter methylation for *TNF* and *IL6*. a) The correlation between *TNF* expression and DNA methylation at six CpG sites within the *TNF* promoter. Data is also shown for all six CpG sites combined. **b)** The correlation between *IL6* expression and DNA methylation at four CpG sites within the *IL6* promoter. The combined methylation for the three probes that showed a negative correlation with gene expression (cg00087425, cg13104385 and cg05265849) are shown. r = Spearman rank coefficient, *p<0.05, **p<0.01, ***p<0.001. The exact p values for each probe are listed in Supplementary Table 8.

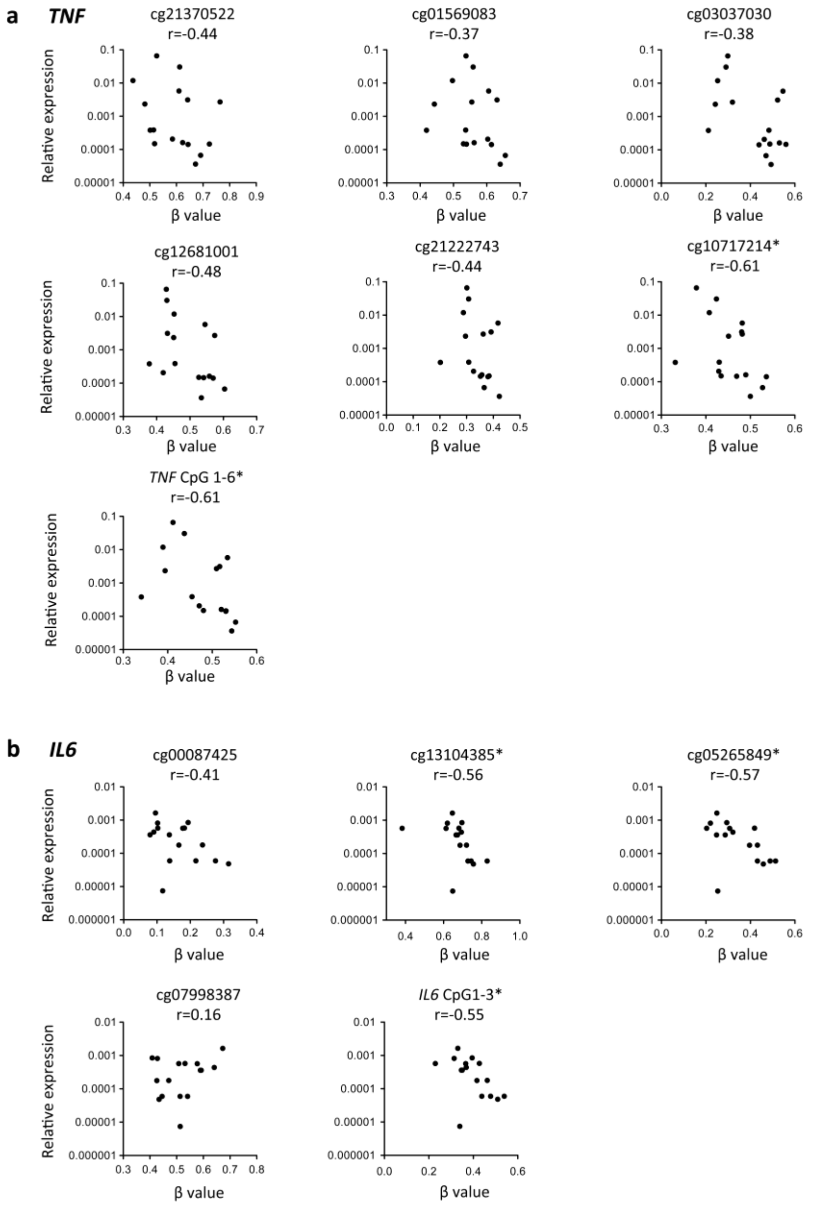

Supplementary Figure 2. Scatter plots showing the correlation between gene expression and promoter methylation for *CXCR2*, *CCL5*, *CCL2* and *IL1A*. a) The correlation between *CXCR2* expression and DNA methylation at three CpG sites within the *CXCR2* promoter. Data is also shown for all three CpG sites combined. **b)** The correlation between *CCL5* expression and DNA methylation at two CpG sites within the *CCL5* promoter. Data is also shown for both CpG sites combined. **c)** The correlation between *CCL2* expression and DNA methylation at one CpG site within the *CCL2* promoter. **d)** The correlation between *IL1A* expression and DNA methylation at one CpG site within the *IL1A* promoter. r = Spearman rank coefficient, *p<0.05, **p<0.01, ***p<0.001. The exact p values for each probe are listed in Supplementary Table 8.

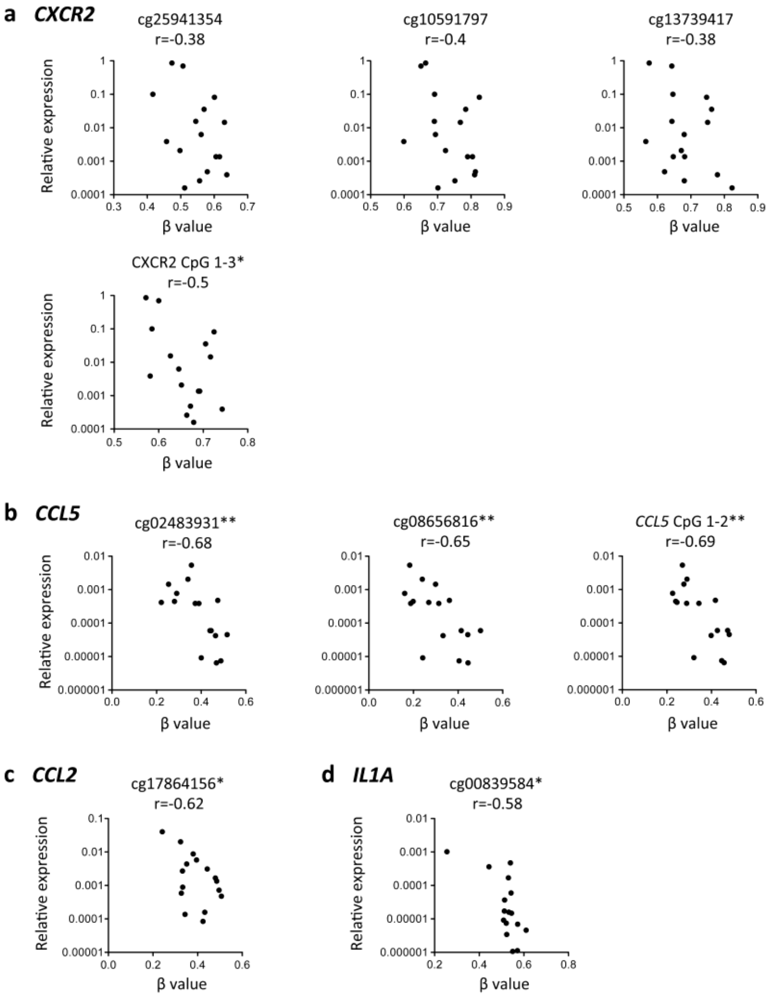

Supplementary Figure 3. Differential methylation of the zinc transporter genes *ZIP4*, *ZIP7*, *ZIP11* and *ZIP14* in OA hip cluster 2. Methylation is shown as the β value from data obtained from our previous HumanMethylation450 BeadChip analysis for 21 NOF, 11 OA hip cluster 1 and 12 OA hip cluster 2 patients. Data is shown as the mean and the standard error of the mean and statistical analysis was performed by one-way ANOVA followed by the Tukey test. ***P<0.001, **P<0.01, *P<0.05.

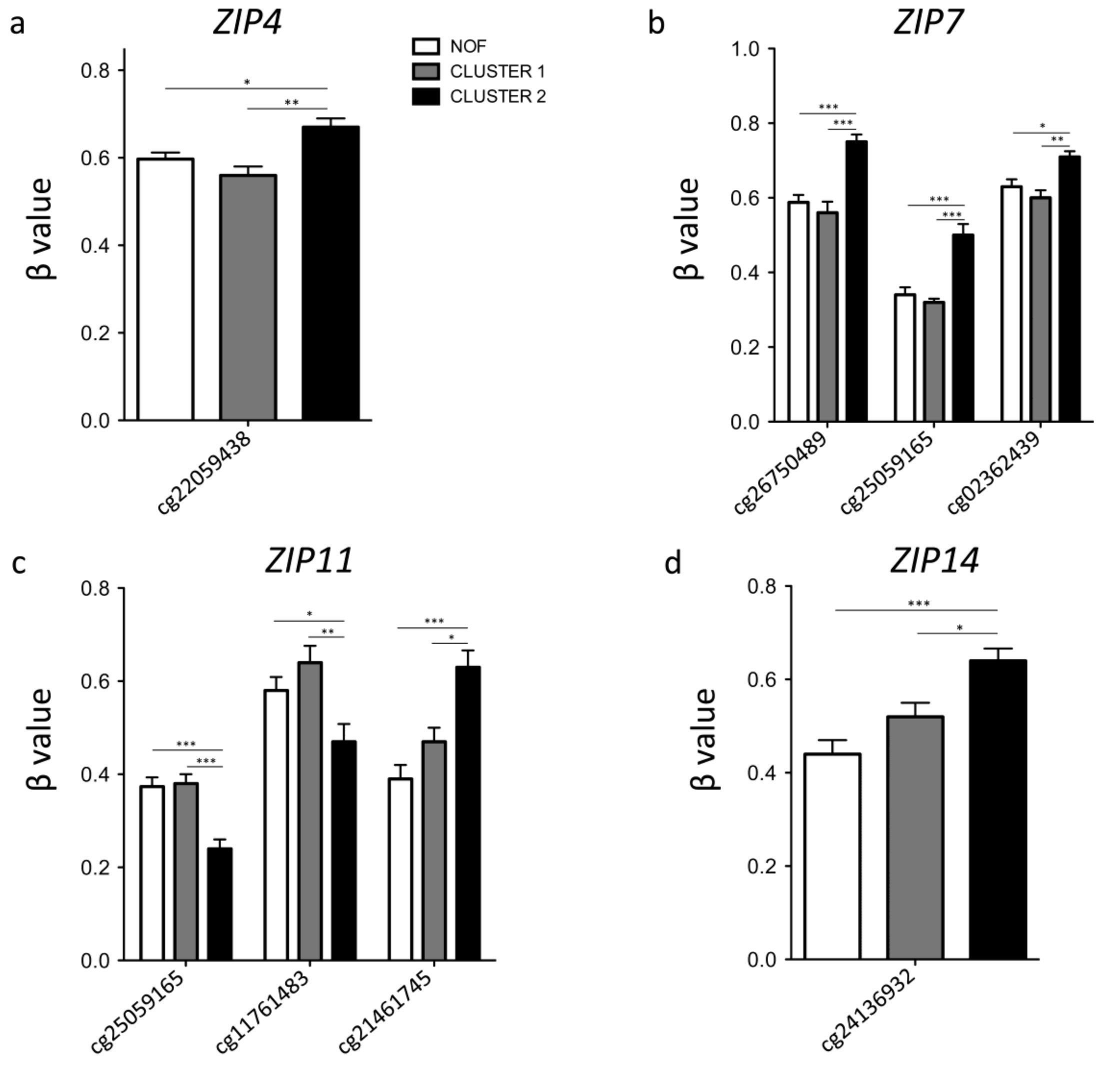

Supplementary Figure 4. Scatter plots showing the correlation between gene expression and DNA methylation within the gene bodies of *ZIP4*, *ZIP7*, *ZIP11* and *ZIP14*. a) The correlation between *ZIP4* expression and DNA methylation at one CpG site within the gene body of *ZIP4*. **b)** The correlation between *ZIP7* expression and DNA methylation at three CpG sites within the gene body of *ZIP7*. **c)** The correlation between *ZIP11* expression and DNA methylation at three CpG sites within the gene body of *ZIP11*. **d)** The correlation between *ZIP14* expression and DNA methylation at one CpG site within the gene body of *ZIP14*. r = Spearman rank coefficient, *p<0.05, **p<0.01, ***p<0.001. The exact p values for each probe are listed in Supplementary Table 10.

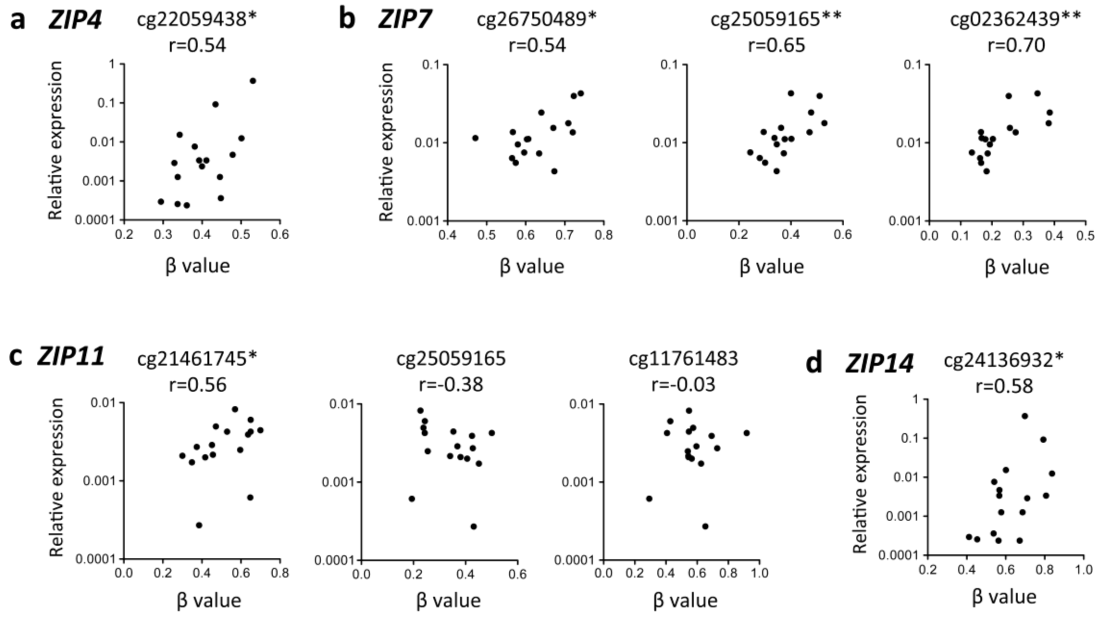

Supplementary Figure 5. Correlation between *ZIP* gene expression and the expression of *ILA*, *IL1B* and *IL6*. Data is presented as $2^{-\Delta Ct}$ (Ct Target gene – Ct average of control gene (*18s*)). r = Spearman rank coefficient, *p<0.05, **p<0.01, ***p<0.001. The exact p values for each probe are listed in Supplementary Table 11.

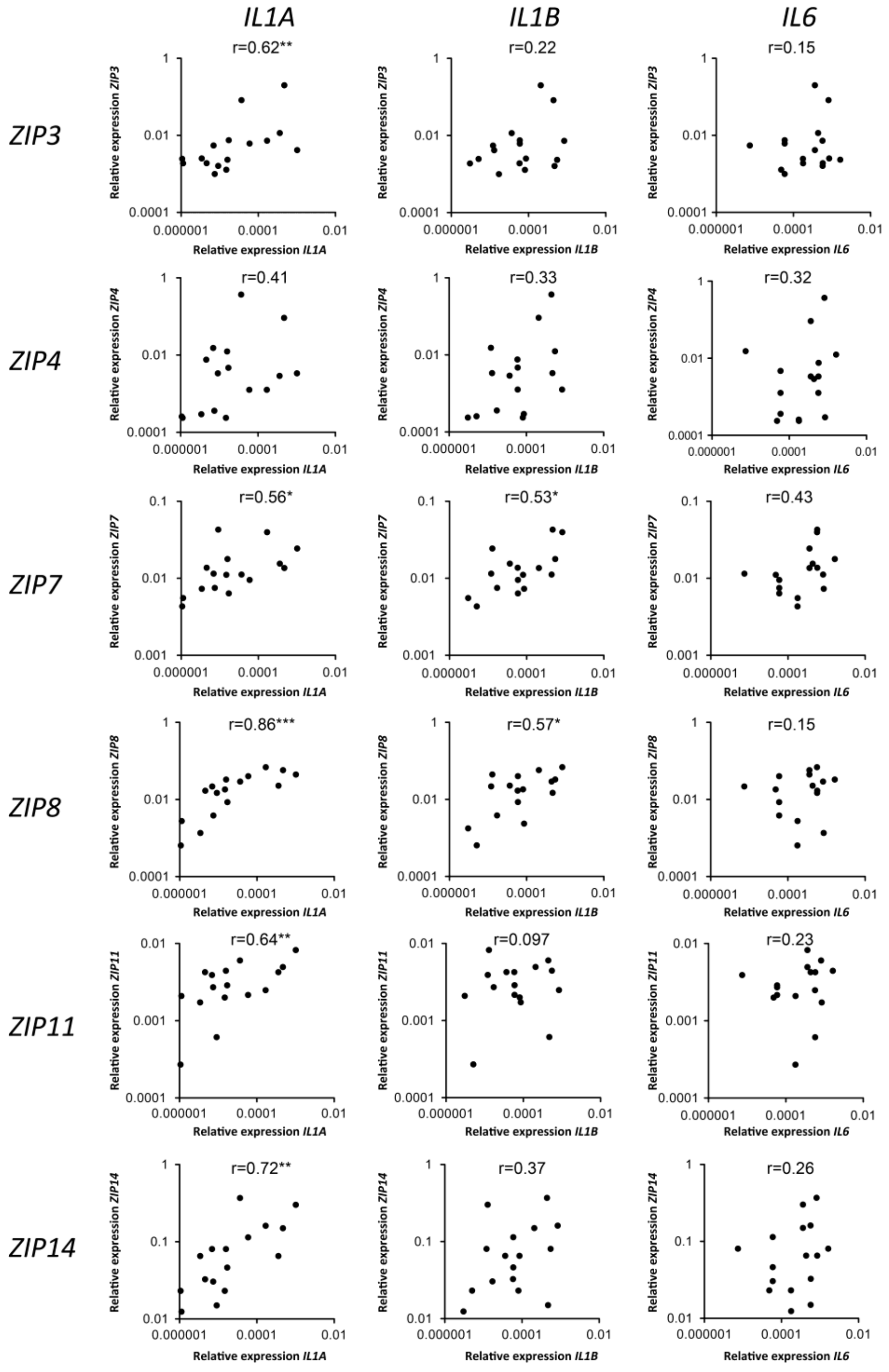

Supplementary Figure 6. Correlation between *ZIP* gene expression and the expression of *TNF*, and the expression of the four pro-inflammatory genes combined (*IL1A*, *IL1B*, *IL6* and *TNF*). Data is presented as $2^{-\Delta Ct}$ (Ct Target gene – Ct average of control gene (*18s*)). r = Spearman rank coefficient, *p<0.05, **p<0.01, ***p<0.001. The exact p values for each probe are listed in Supplementary Table 11.

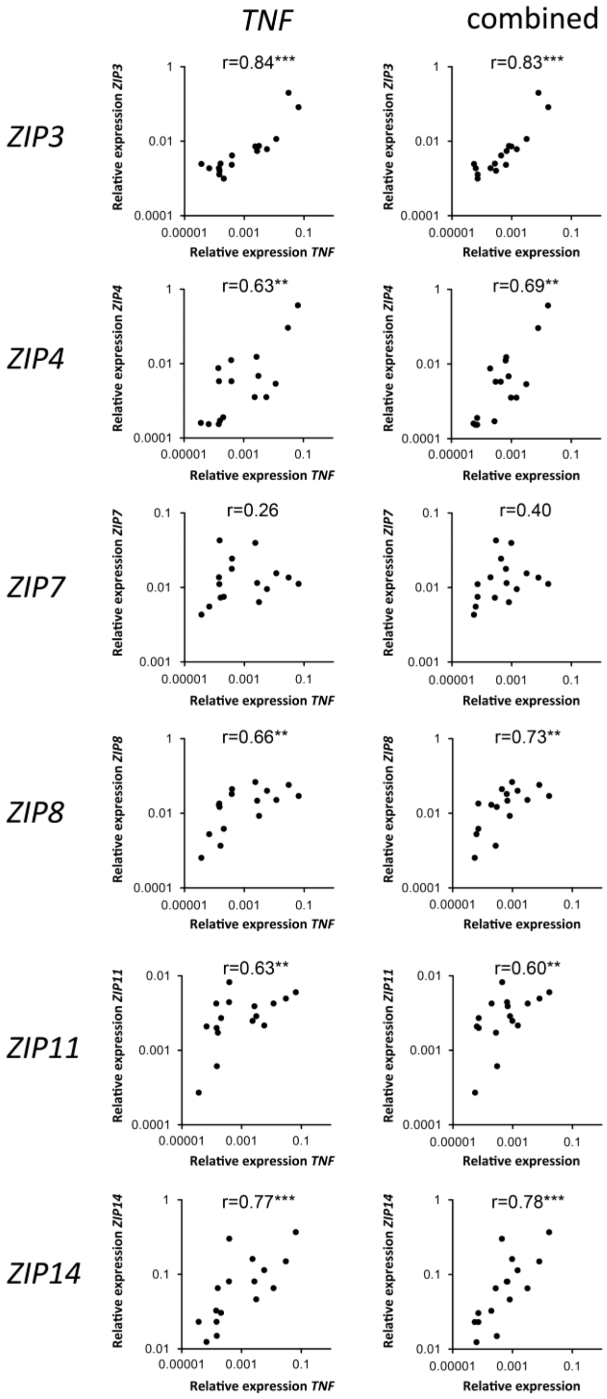

Supplement: Web figure [file annrheumdis-2014-206752-s2.pdf]
