# Supplementary material for: Differential DNA methylation and expression of inflammatory and zinc transporter genes defines subgroups of osteoarthritic hip patients
Source: Ann Rheum Dis. 2015 Apr 8;74(9):1778–82. doi: 10.1136/annrheumdis-2014-206752 (PMC4552898; doi:10.1136/annrheumdis-2014-206752)
Supplement: Web table 1 [file annrheumdis-2014-206752-s3.pdf]

Supplementary Table 1. Pateint information. OA = osteoarthritis, NOF = Neck of Femur.

Sample ID	Phenotype	Sex	Age at surgery (years)	Nucleic acid extracted
3125	OA	M	74	DNA
3417	OA	F	58	DNA
3452	OA	F	69	DNA
3727	OA	F	71	DNA + RNA
3930	OA	M	66	DNA
4263	OA	M	77	DNA
4267	OA	M	85	DNA
4290	OA	M	76	DNA + RNA
4295	OA	F	68	DNA
4337	OA	F	76	DNA + RNA
4428	OA	M	59	DNA + RNA
4479	OA	F	76	DNA
4485	OA	M	48	DNA + RNA
4486	OA	F	59	DNA + RNA
4490	OA	M	90	DNA + RNA
4491	OA	F	52	DNA + RNA
4631	OA	F	73	DNA + RNA
4663	OA	F	57	DNA
4705	OA	M	69	DNA
4713	OA	F	45	DNA
4758	OA	F	72	DNA
4791	OA	M	66	DNA + RNA
4841	OA	F	71	DNA + RNA
T005	NOF	M	69	DNA
T007	NOF	F	72	DNA
T016	NOF	F	79	DNA + RNA
T023	NOF	F	68	DNA
T028	NOF	F	86	DNA
T034	NOF	F	91	DNA
T036	NOF	F	80	DNA
T039	NOF	F	80	DNA + RNA
T059	NOF	F	82	DNA
T076	NOF	M	81	DNA
T094	NOF	F	63	DNA
T107	NOF	M	72	DNA
T117	NOF	M	85	DNA + RNA
T123	NOF	F	78	DNA
T126	NOF	M	74	DNA
T132	NOF	M	84	DNA
T138	NOF	F	93	DNA + RNA

T141	NOF	F	84	DNA
T142	NOF	F	78	DNA
T144	NOF	M	75	DNA
T145	NOF	F	83	DNA
