# Supplementary material for: Differential DNA methylation and expression of inflammatory and zinc transporter genes defines subgroups of osteoarthritic hip patients
Source: Ann Rheum Dis. 2015 Apr 8;74(9):1778–82. doi: 10.1136/annrheumdis-2014-206752 (PMC4552898; doi:10.1136/annrheumdis-2014-206752)
Supplement: Web table 10 [file annrheumdis-2014-206752-s4.pdf]

Supplementary Table 10. Correlation between gene expression and methylation within the gene bodies of *ZIP4*, *ZIP7*, *ZIP11* and *ZIP14*. Scatter plots of the correlations below are shown in Supplementary Figure 4.

**Gene**	**CpG probe ID**	**Spearman correlation**	**p value**
ZIP4	cg22059438	0.54	0.031
ZIP7	cg26750489	0.54	0.031
	cg25059165	0.65	0.0064
	cg02362439	0.70	0.0025
ZIP11	cg25067702	-0.38	0.15
	cg11761483	-0.03	0.91
	cg21461745	0.56	0.024
ZIP14	cg24136932	0.58	0.019
