# Supplementary material for: Differential DNA methylation and expression of inflammatory and zinc transporter genes defines subgroups of osteoarthritic hip patients
Source: Ann Rheum Dis. 2015 Apr 8;74(9):1778–82. doi: 10.1136/annrheumdis-2014-206752 (PMC4552898; doi:10.1136/annrheumdis-2014-206752)
Supplement: Web table 11 [file annrheumdis-2014-206752-s5.pdf]

Supplementary Table 11. Correlation between pro-inflammatory genes and ZIP genes that are differentially expressed in the OA hip clusters. The correlation is also shown for all four pro-inflammatory genes combined. Scatter plots of the correlations are shown in Supplementary Figure 5 and Supplementary Figure 6.

Pro-inflammatory gene	ZIP gene	Spearman correlation	p value
IL1A	*ZIP3*	0.62	0.009
	ZIP4	0.41	0.1
	ZIP7	0.56	0.02
	ZIP8	0.86	0.00003
	ZIP11	0.64	0.008
	ZIP14	0.72	0.002
IL1B	*ZIP3*	0.22	0.4
	ZIP4	0.33	0.2
	ZIP7	0.53	0.04
	ZIP8	0.57	0.02
	ZIP11	0.097	0.7
	ZIP14	0.37	0.2
IL6	*ZIP3*	0.15	0.6
	ZIP4	0.32	0.2
	ZIP7	0.43	0.1
	ZIP8	0.15	0.6
	ZIP11	0.23	0.4
	ZIP14	0.26	0.3
TNF	*ZIP3*	0.84	0.00001
	ZIP4	0.63	0.008
	ZIP7	0.26	0.3
	ZIP8	0.66	0.005
	ZIP11	0.63	0.009
	ZIP14	0.77	0.0005
**Combined**	*ZIP3*	0.87	0.00001
	ZIP4	0.69	0.0031
	ZIP7	0.40	0.1
	ZIP8	0.73	0.001
	ZIP11	0.60	0.01
	ZIP14	0.78	0.0003
