# Supplementary material for: Differential DNA methylation and expression of inflammatory and zinc transporter genes defines subgroups of osteoarthritic hip patients
Source: Ann Rheum Dis. 2015 Apr 8;74(9):1778–82. doi: 10.1136/annrheumdis-2014-206752 (PMC4552898; doi:10.1136/annrheumdis-2014-206752)
Supplement: Web table 2 [file annrheumdis-2014-206752-s6.pdf]

Supplementary Table 2. Primer sequences used in this study, all primer sets were used for measuring gene expression by real-time PCR using SYBR® green chemistry.

Gene	Primer sequence
CCL2	F – 5' GGTACATCCTCGACGGCATCT 3'<br>R – 5' GTGCCTCTTTGCTGCTTTCAC 3'
CCL5	F – 5' CCCTCGCTGTCATCCTCAT 3'<br>R – 5' AGTGGGCGGGCAATGTA 3'
CXCR2	F – 5' TCTGGATGCCACCGAGATTCT 3'<br>R – 5' AGTCCATGGCGAAACTTC 3'
IL6	F – 5' GGTACATCCTCGACGGCATCT 3'<br>R – 5' GTGCCTCTTTGCTGCTTTCAC 3'
IL1A	F – 5' AACCAGTGCTGCTGAAGGA 3'<br>R – 5' TTCTTAGTGCCGTGAGTTTCC 3'
IL1B	F – 5' CTGTCCTGCGTGTTGAAAGA 3'<br>R – 5' TTGGGTAATTTTTGGGATCTACA 3'
TNF	F – 5' GGAGAAGGGTGACCGACTCA 3'<br>R – 5' CTGCCCAGACTCGGCAA 3'
ZIP3	F – 5' TGGCCGAAACCATCCTCCT 3'<br>R – 5' GATCCGGCGTTGAAGGTCTC 3'
ZIP4	F – 5' ATGTCAGGAGCGGGTCTTGC 3'<br>F – 5' GCTGCTGTGCTGCTGGAAC 3'
ZIP6	F – 5' TCTCTGTCACAAATCCCCTTCA 3'<br>R – 5' GATATTGCCGTGTGGAAATTGC 3'
ZIP7	F – 5' CCACAGGCACTCACATGAAGA 3'<br>R – 5' CGTGATCGTGGGTATGTCCAT 3'
ZIP8	F – 5' TGCTACCCAAATAACCAGCTCC 3'<br>R – 5' ACAGGAATCCATATCCCCAAACT 3'
ZIP9	F – 5' TCTCTGGCTATGTTGGTGGGA 3'<br>R – 5' CCAGCACCCAAAACAGTCAC 3'
ZIP10	F – 5' ACACCAGATTCTGACTGGCTT 3'<br>R – 5' TAGGAGGGGATTCTTGTTGGC 3'
ZIP11	F – 5' TGCTGGGGACCTTCTTCAC 3'<br>R – 5' GCCAAGACTTCCATCTAAGATCC 3'
ZIP13	F – 5' TCCTGGGTTCCCTCATGGT 3'<br>F – 5' AGATGCAGAAACACATTGCCC 3'
ZIP14	F – 5' CCTGAGGCTCACGCTTCATC 3'<br>R – 5' TCACCCTCGCCATACCGAT 3'
ZNT1	F – 5' CCTCGCGTTAAGAGCACCC 3'<br>R – 5' CAATTTCAGCCCGTTGGAGTT 3'
ZNT4	F – 5' GACCTAAGCGCCATCATACTC 3'<br>F – 5' AGCTGACAAAACCTCTAAGCG 3'
ZNT5	F – 5' ACCAAACACCAGTGGATCAAAA 3'<br>R – 5' CAGCAAAGTCCTTAGTGGTCC 3'
ZNT7	F – 5' GCTTAGGCTTGATTTCCGACT 3'<br>F – 5' CCAGAACTTCCGCTCTAACATAC 3'
ZNT9	F – 5’ ACTGGAGTCAGAGCGATAAATGA 3’<br>R – 5’ GCTTCCCCAAACTTCCAAAGAT 3’
MTF1	F – 5' CGAAGGAGAAGCCATTTGAG 3'<br>R – 5' TGGCTTTTCCCCTGTATGAG 3'
MT1A	F – 5' GGCTCCTGCACCTGCACT 3'<br>F – 5' ACAGCAGCTGCACTTCTCTGA 3'
MT1G	F – 5' TCCTGCAAGTGCAAAGAGTG 3'<br>R – 5' CAGCTGCACTTCTCCGATG 3'
MT1H	F – 5' GCTCCTGCAAGTGCAAAAAG 3'<br>R – 5' CAGCAGCTGCACTTCTCTGA 3'
