# Supplementary material for: Differential DNA methylation and expression of inflammatory and zinc transporter genes defines subgroups of osteoarthritic hip patients
Source: Ann Rheum Dis. 2015 Apr 8;74(9):1778–82. doi: 10.1136/annrheumdis-2014-206752 (PMC4552898; doi:10.1136/annrheumdis-2014-206752)
Supplement: Web table 3 [file annrheumdis-2014-206752-s7.pdf]

Supplementary Table 3. List of hypomethylated DMLs in OA hip cluster 2.

Hypomethylated in OA hip cluster 2						
**Probe**	**Gene**	**CHR**	**adj.P.Val**	**mean β NOF**	**mean β cluster1**	**mean β cluster2**
cg00962147	A2BP1;A2	16	0.01146765	0.74233854	0.72978296	0.62903714
cg00134295	A2M	12	0.0212437	0.66795941	0.68725407	0.52561437
cg09866565	A4GNT	3	0.00685049	0.50443983	0.53497227	0.3295404
cg02766259	AACS	12	0.00242331	0.80836793	0.79118933	0.68056551
cg22081096	ABCA6;AE	17	0.00091597	0.73285825	0.64688145	0.52608518
cg25477497	ABCB1	7	0.01678444	0.71084022	0.80506795	0.60709541
cg00355656	ABCB10	1	0.0231425	0.73818401	0.72218061	0.60104963
cg09648454	ABCC12	16	0.00065949	0.5346487	0.36779996	0.26118054
cg07861968	ABCC12;A	16	0.00066232	0.86537473	0.80905814	0.69218839
cg02672229	ABCC2	10	0.00576931	0.81955348	0.78788013	0.65213279
cg25928474	ABCC3;AE	17	0.00073118	0.87532619	0.80644979	0.63044963
cg24260135	ABCC8	11	0.02536838	0.64618237	0.70666131	0.52930566
cg05878073	ABCD4;AE	14	0.01918451	0.31425431	0.36815468	0.19290274
cg21052932	ABHD12B	14	0.00262855	0.56837298	0.57606229	0.41496463
cg17941109	ABHD8	19	0.00073378	0.65760391	0.55598492	0.43715043
cg26334023	ABI3;GNG	17	0.00362629	0.6791491	0.67060615	0.52884351
cg03471346	ABL2;ABL	1	0.00131453	0.86957789	0.83165829	0.66986207
cg17056676	ABLIM1;A	10	0.00076471	0.52471204	0.44697031	0.32962932
cg19676285	ABLIM2;A	4	0.00072814	0.70752667	0.58643683	0.38095841
cg00245502	ABLIM3	5	0.00661882	0.51080444	0.51479898	0.38908238
cg11807006	ACAP1	17	0.00715821	0.48838767	0.46513975	0.34607663
cg16927372	ACCN1	17	0.02649037	0.62063396	0.60561146	0.49847813
cg04114655	ACCN4;A(	2	0.00555886	0.52643316	0.48929914	0.38707368
cg15736994	ACCN5	4	0.01518408	0.59169868	0.60745002	0.4871451
cg06956006	ACLY;ACL	17	0.00197515	0.89924724	0.85669951	0.73503052
cg09636163	ACOXL;A(	2	0.00745107	0.39366006	0.44664801	0.27882723
cg24187645	ACPL2;AC	3	0.00066232	0.60103443	0.5071725	0.36974085
cg18733757	ACSL5;AC	10	0.01980843	0.4488663	0.44045273	0.28329802
cg04844987	ACSL5;AC	10	0.01396719	0.46655208	0.48083693	0.323261
cg10078415	ACSM3;A(	16	0.02003709	0.33618104	0.33613916	0.20173615
cg16736889	ACSM5	16	0.00358002	0.78542874	0.75943897	0.65862684
cg08734527	ACTL8	1	0.00247433	0.65991118	0.59026685	0.46178479
cg03220543	ACTL8	1	0.00376781	0.54778592	0.56931347	0.41909398
cg02057747	ACTN1;A(	14	0.00359124	0.55809377	0.57315638	0.3770956
cg26831416	ACTN4	19	0.00260155	0.76078557	0.71403228	0.51314317
cg15211996	ACTRT2	1	0.01711555	0.69773105	0.68145669	0.54540549
cg15342452	ADAM12;	10	0.00418936	0.85824268	0.8893625	0.75380099
cg01368217	ADAM12;	10	0.00717683	0.81827033	0.78433169	0.67397272
cg04834770	ADAM12;	10	0.01237591	0.75858155	0.73220216	0.55004785
cg02235497	ADAM12;	10	0.00830789	0.85286154	0.82072073	0.70276232

cg01823925	ADAM19	5	0.00186306	0.78525944	0.75616812	0.65606074
cg08295410	ADAM19	5	0.00398606	0.8245932	0.80535189	0.6685688
cg10852045	ADAM32	8	0.01484626	0.79449217	0.80163298	0.61961288
cg01181233	ADAMDE(	8	0.00160662	0.36480768	0.36037198	0.17286623
cg27015773	ADAMTS1	19	0.0009934	0.81990435	0.72521889	0.57791549
cg07974833	ADAMTS3	4	0.02630662	0.6249669	0.56736347	0.45502511
cg03294207	ADAMTS5	21	0.0008696	0.83588401	0.74904949	0.58244801
cg00646084	ADAMTS5	21	0.01183269	0.75273061	0.67707672	0.57269736
cg14740657	ADAMTS5	21	0.00209829	0.70276404	0.61365714	0.45647284
cg21033632	ADAMTS6	5	0.00436875	0.67915265	0.62521268	0.52211085
cg16714091	ADAMTSL	9	0.04180327	0.74811422	0.77490521	0.63458961
cg22731190	ADAMTSL	9	0.00349176	0.60420712	0.5587063	0.40796127
cg02123534	ADAP1	7	0.02697984	0.72943044	0.73570539	0.54779778
cg03754605	ADARB2	10	0.00109472	0.89901895	0.86206711	0.76142546
cg08971562	ADARB2	10	0.00725225	0.39350393	0.37623206	0.25353698
cg04012681	ADARB2	10	0.00131641	0.63706114	0.61161102	0.45993247
cg02887530	ADARB2	10	0.00315788	0.46163832	0.47035666	0.31263607
cg11343713	ADCY1	7	0.00425638	0.56566348	0.57828371	0.44729569
cg16548911	ADCY7	16	0.01645625	0.81519376	0.82120963	0.66968315
cg10341242	ADCY7	16	0.00775785	0.82254901	0.81664078	0.66097843
cg07644321	ADD1;ADI	4	0.03060322	0.64536572	0.67228494	0.52365256
cg12011299	ADH4	4	0.01313275	0.55980874	0.59506924	0.36111017
cg23328194	ADRA1A;/	8	0.00066204	0.67214367	0.56299442	0.38231661
cg17979970	ADRA1B	5	0.00552473	0.63128073	0.6315038	0.52379923
cg00474091	AEBP2;AE	12	0.02156272	0.65052599	0.59120782	0.47303236
cg13670531	AFAP1L2;	10	0.04104022	0.85044936	0.84108821	0.71476917
cg22199080	AGAP2	12	0.00780052	0.29293941	0.34827719	0.16444929
cg21351392	AGPAT4	6	0.00099652	0.82965	0.80616758	0.6601434
cg01395754	AGPS	2	0.01645394	0.72657616	0.71096597	0.56752244
cg14252395	AGRN	1	0.02683244	0.68703502	0.68207552	0.57452521
cg24371033	AGXT	2	0.00214604	0.31320579	0.27108041	0.1659865
cg03822873	AHCYL2;A	7	0.00642585	0.67067578	0.72788861	0.51780815
cg25272143	AHNAK	11	0.02381973	0.32888894	0.2942107	0.18652959
cg16509239	AHNAK	11	0.0249821	0.40064422	0.37623816	0.26499996
cg11138227	AHNAK2	14	0.00593004	0.70044597	0.64392489	0.53389154
cg01513078	AHNAK2	14	0.0012071	0.5236927	0.40290125	0.2936353
cg06799735	AHNAK2	14	0.00065949	0.70805154	0.58450752	0.46528842
cg23916896	AHRR	5	0.00263648	0.72699011	0.73830569	0.48622151
cg00490406	AIM2	1	0.01974947	0.82539844	0.83550989	0.67026135
cg21052656	AIP	11	0.00911882	0.57335306	0.56620929	0.39987128
cg21747271	AIP	11	0.01361478	0.43842617	0.41682504	0.29336593
cg05254518	AKAP13;A	15	0.00661882	0.55925207	0.59198655	0.35863344
cg14003853	AKAP13;A	15	0.04406604	0.52410147	0.54337876	0.40038452
cg11314684	AKT3;AKT	1	0.01473021	0.5730223	0.61921282	0.44801005
cg06400255	ALDOA	16	0.00178025	0.84731361	0.81964794	0.59150236
cg04329347	ALDOA	16	0.04292124	0.51851787	0.56368995	0.40371265

cg18780653	ALG1L2	3	0.00552206	0.40954713	0.43956296	0.29133452
cg05977776	ALLC	2	0.002727	0.64069923	0.66450115	0.52240377
cg13306478	ALOX5AP	13	0.00073265	0.76222857	0.6614748	0.44866086
cg12466599	ALPL;ALPI	1	0.00141412	0.72601437	0.70989237	0.56324065
cg24722348	ALPL;ALPI	1	0.02846049	0.52061428	0.51725818	0.39902129
cg16413687	ALX1	12	0.00833663	0.7275089	0.70466094	0.58893105
cg03781931	ALX1	12	0.00223317	0.56202554	0.53283275	0.34341498
cg06809252	ALX3	1	0.00203482	0.60709158	0.55834132	0.45738104
cg16360777	AMBN	4	0.00159601	0.30854053	0.37290038	0.16910458
cg13003878	AMBP	9	0.03305949	0.73584023	0.77556659	0.62762532
cg27137887	AMBRA1	11	0.00215533	0.51824849	0.50987617	0.33405292
cg24374161	AMBRA1	11	0.00585298	0.51370813	0.50016637	0.25734204
cg07376232	AMICA1;/	11	0.01500741	0.59833921	0.65129238	0.49107344
cg13751113	AMICA1;/	11	0.01040861	0.6017863	0.64062162	0.47970661
cg23092449	AMPH;AN	7	0.00132756	0.43374692	0.42048468	0.272363
cg21823502	ANAPC11	17	0.00197576	0.7978189	0.74957488	0.63977931
cg09396217	ANGPT1	8	0.02252087	0.52770636	0.53969422	0.37561997
cg27616227	ANGPT1	8	0.00195832	0.63630751	0.62403998	0.40008498
cg02402368	ANGPT2;/	8	0.00499799	0.30354697	0.2923464	0.18457255
cg21193744	ANGPT2;/	8	0.00092643	0.63206501	0.50541031	0.39154431
cg08521995	ANK1;ANI	8	0.00065949	0.69047802	0.52208998	0.3362733
cg22164298	ANK2;ANI	4	0.01501672	0.79911638	0.85614143	0.65059601
cg14011971	ANK3	10	0.0249821	0.49667349	0.4752203	0.35802838
cg26110900	ANK3	10	0.00104778	0.78873934	0.7343917	0.59686937
cg23075597	ANKLE2	12	0.0068559	0.82929529	0.92275337	0.6782925
cg24065504	ANKRD22	10	0.00219431	0.61126918	0.56278678	0.41917749
cg01442620	ANKRD27	19	0.00956928	0.64511324	0.6447717	0.46015169
cg13941760	ANKRD44	2	0.01742221	0.54799324	0.63897069	0.43719627
cg00794673	ANKS1A	6	0.00288962	0.72901185	0.70559601	0.46496535
cg26401673	ANO10	3	0.00653182	0.78816654	0.82040894	0.63590771
cg02278646	ANO6;AN	12	0.01994073	0.61741678	0.63332379	0.44284391
cg20366110	ANXA13;/	8	0.0035878	0.59832847	0.64265431	0.44940748
cg19130824	ANXA5	4	0.00124521	0.77277058	0.75814399	0.65395055
cg02415661	AP1M1;A	19	0.0029234	0.69853382	0.64348231	0.4590827
cg19175742	AP3B2	15	0.00441677	0.55988023	0.55688041	0.44840773
cg23007087	AP3S2;AP	15	0.00728372	0.76980192	0.73035476	0.60803918
cg02349866	AP3S2;AP	15	0.00066204	0.81318675	0.76010607	0.50207963
cg06970472	APBB2;AF	4	0.00219711	0.3474183	0.34141218	0.21543118
cg14290210	APBB2;AF	4	0.00713936	0.80153548	0.80545052	0.63106542
cg25354657	APLP2;AP	11	0.00081925	0.87090491	0.80313488	0.68174949
cg23729763	APLP2;AP	11	0.00073118	0.68360866	0.56327358	0.35108311
cg24360617	APOBEC3.	22	0.00670577	0.37638144	0.38590549	0.2532536
cg10316474	APOBEC3	22	0.00456818	0.25302481	0.25484337	0.14853278
cg16066354	APOBEC3	22	0.00115308	0.34457145	0.29212936	0.15159788
cg11911305	APOC1P1,	19	0.01670995	0.58037434	0.60168151	0.42840831
cg26758857	APOL1;AF	22	0.00322924	0.31972347	0.34001166	0.1774538

cg07713361	APOL1;AF	22	0.00228672	0.41393056	0.32705419	0.17794996
cg09432376	APOL6	22	0.0075368	0.68559853	0.66196218	0.4822253
cg14690065	APOL6	22	0.00272772	0.62475895	0.56798288	0.41166809
cg20176648	AQP1	7	0.00339981	0.40149496	0.39359237	0.28508806
cg00622010	AQP1	7	0.00088355	0.67530026	0.66753754	0.54911524
cg09676669	AQP1	7	0.00283173	0.4199709	0.39530621	0.21826761
cg15373767	AQP1	7	0.00196226	0.51925991	0.47588288	0.28444788
cg04372674	AQP1	7	0.00245414	0.67250003	0.64007738	0.3753617
cg25075794	AQP1	7	0.0034485	0.66866969	0.65385355	0.39642765
cg25230363	AQP1	7	0.00219764	0.63803611	0.60703855	0.38902794
cg26923410	AQP1	7	0.00412512	0.35804491	0.33012648	0.21202903
cg07135629	AQP1	7	0.00073378	0.8696467	0.7859498	0.66170115
cg10132917	AQP1	7	0.00065949	0.85689903	0.76952566	0.63742642
cg18307978	AQP1	7	0.00081413	0.75957295	0.69122747	0.47524506
cg18080604	AQP1;AQ	7	0.00620309	0.34296544	0.34116162	0.20956758
cg11827925	AQP1;AQ	7	0.00534171	0.58495961	0.57662104	0.39220472
cg00729275	AQP4;C18	18	0.04018123	0.67869433	0.61523749	0.51312072
cg11752250	ARG2	14	0.02248036	0.63899333	0.60319145	0.45150599
cg03206044	ARHGAP1	4	0.00297897	0.61909333	0.56828396	0.4438549
cg01782668	ARHGAP1	4	0.00128378	0.89126896	0.81577655	0.69953867
cg24506434	ARHGAP2	10	0.00065949	0.77104008	0.60153582	0.48875261
cg22184990	ARHGAP2	5	0.00753573	0.80339074	0.82366996	0.66552426
cg14555350	ARHGAP2	5	0.00073265	0.87430358	0.82394375	0.71883971
cg20917083	ARHGDIB	12	0.01245683	0.42155855	0.44387394	0.28056728
cg11806633	ARHGEF1	1	0.01820811	0.72478026	0.75172372	0.62231194
cg07233343	ARHGEF1	1	0.00129758	0.71014415	0.62094994	0.48672483
cg22524332	ARHGEF1	1	0.00079133	0.79120551	0.67804716	0.55778508
cg02731917	ARHGEF1	1	0.00677233	0.8359148	0.80351271	0.7006396
cg11229273	ARHGEF3	3	0.00107266	0.51804069	0.33831701	0.20161285
cg21077300	ARHGEF3	3	0.01257717	0.35079681	0.35693996	0.24985282
cg25799109	ARHGEF3	3	0.00181386	0.28803525	0.27569187	0.12797056
cg14446494	ARHGEF4	2	0.00499799	0.71055104	0.69450433	0.58096843
cg18754629	ARHGEF4	2	0.00119376	0.55787378	0.5543288	0.43284049
cg18973247	ARID1A;A	1	0.00089205	0.33893777	0.25389914	0.12849562
cg24495062	ARID5B	10	0.00297897	0.61580113	0.57911121	0.46370467
cg18522931	ARID5B	10	0.036396	0.73379917	0.75494917	0.56301424
cg11287647	ARL4A;AR	7	0.00549982	0.47350179	0.4835921	0.28596015
cg05204104	ARL4C;AR	2	0.00803433	0.6817276	0.68416818	0.49903055
cg15016771	ARL4C;AR	2	0.0012838	0.2910003	0.31688033	0.17336109
cg06795960	ARL4D	17	0.01237894	0.72209082	0.70550449	0.60389457
cg04642300	ARMC2	6	0.01823439	0.40849852	0.45295615	0.26810795
cg20057782	ARMC4	10	0.02528265	0.35667758	0.36163144	0.2549997
cg01446731	ARNT2	15	0.00073118	0.69523122	0.4624446	0.3442512
cg19708986	ARNT2	15	0.00112679	0.72852597	0.69752502	0.55867845
cg26165146	ARNTL2	12	0.00661882	0.88463544	0.83005417	0.7171228
cg21756647	ARRB1;AF	11	0.00065949	0.60977999	0.48104005	0.34536438

cg00970435	ARSG	17	0.00264473	0.5774576	0.54326376	0.2991214
cg17918314	ARTN;ART	1	0.00177922	0.46912961	0.47346165	0.35701531
cg00013804	ASAP2;AS	2	0.00073118	0.86768221	0.79311448	0.61894381
cg17056069	ASAP2;AS	2	0.02450621	0.75283993	0.85152813	0.62356694
cg23902076	ASAP2;AS	2	0.00065949	0.88303947	0.7859464	0.64446558
cg18008487	ASB4;ASB	7	0.02792018	0.59288036	0.59910739	0.45455153
cg14516287	ASTN2;AS	9	0.02329073	0.60964954	0.62439877	0.45781813
cg07676329	ATCAY	19	0.00206218	0.41566121	0.41738529	0.31524648
cg03293837	ATE1;ATE	10	0.02346046	0.79332924	0.8492758	0.67023873
cg00458449	ATG4B;AT	2	0.00297897	0.7906857	0.80746984	0.58040185
cg06956052	ATP1A4	1	0.00065949	0.49734984	0.42858022	0.32422231
cg05299486	ATP6V0A:	17	0.02152957	0.49590022	0.56958252	0.38593743
cg07554478	ATP6V1A	3	0.00798815	0.75681784	0.73443375	0.59907246
cg12384004	ATP8A2	13	0.0294913	0.60441294	0.72238276	0.4998779
cg05868813	ATP8A2	13	0.00687165	0.76229098	0.76726592	0.63291307
cg20663852	ATP9A	20	0.00725225	0.54027759	0.51437498	0.32585029
cg07339236	ATP9A	20	0.01387472	0.76426001	0.81908028	0.64869167
cg22274117	ATXN1;AT	6	0.00279494	0.64274654	0.60082733	0.34874592
cg07932199	ATXN2	12	0.01096214	0.34716626	0.37196758	0.21792767
cg21557473	AUTS2;AU	7	0.01580869	0.80261497	0.85063843	0.68746372
cg04046364	AVIL	12	0.00288962	0.36770223	0.30974583	0.18050156
cg24123679	AZU1	19	0.00936413	0.42485624	0.46330612	0.31798679
cg10752406	AZU1	19	0.00820352	0.47898077	0.48342809	0.35331375
cg15610437	AZU1	19	0.04407901	0.58103259	0.5948085	0.4740462
cg26390598	B3GALT5;	21	0.02348349	0.37434796	0.41595508	0.25762816
cg22212839	B3GAT1;B	11	0.01717855	0.57735669	0.56580458	0.42599716
cg24391854	B3GAT1;B	11	0.04433808	0.72041631	0.75379019	0.61529492
cg13567349	B3GAT1;B	11	0.00244887	0.64225287	0.61299438	0.48362031
cg27121870	B3GAT1;B	11	0.00575746	0.61902033	0.60667699	0.37650055
cg25521400	B3GNT2	2	0.02691069	0.57376946	0.55302963	0.41176807
cg11115976	B3GNTL1	17	0.04884648	0.90077493	0.89826019	0.79314595
cg17486314	BACH2;BA	6	0.00176085	0.55468517	0.51751686	0.33974239
cg01303372	BAG3	10	0.03960534	0.38356404	0.38404293	0.24374911
cg01810575	BAHCC1	17	0.01860545	0.61978626	0.63949217	0.51044672
cg13739640	BAIAP2;BA	17	0.02047468	0.79993095	0.85762918	0.68958393
cg27551910	BAIAP2;BA	17	0.04006803	0.60059051	0.62166768	0.49865632
cg14374754	BARX1	9	0.00758031	0.67987545	0.62953338	0.38146624
cg22902266	BARX1	9	0.0015244	0.67080207	0.57306225	0.35686968
cg07355688	BARX2	11	0.00757575	0.38085592	0.37519335	0.27118039
cg11854007	BASE	20	0.04358513	0.42629385	0.43782976	0.29201766
cg16281534	BASP1	5	0.00197986	0.7100415	0.70906623	0.52500081
cg04536765	BAT3;BAT	6	0.00240796	0.82313743	0.7863145	0.6436974
cg27014438	BAT3;BAT	6	0.00144365	0.38885964	0.24361182	0.13900945
cg22161706	BBS9;BBS	7	0.00130049	0.90968787	0.87527196	0.75505547
cg24965479	BCAS3;BC	17	0.00742728	0.45010339	0.4096588	0.21922418
cg22214889	BCAS4;BC	20	0.0011104	0.31417514	0.29807383	0.17268931

cg24244747	BCKDHB;I	6	0.00232154	0.77285938	0.79154922	0.55916844
cg01636910	BCL10	1	0.01663906	0.731044	0.74572872	0.58203074
cg17935536	BCL11A;B	2	0.01842786	0.64823351	0.68152028	0.52156086
cg13758331	BCL2L11;I	2	0.01264372	0.46543997	0.45925785	0.29031102
cg27087650	BCL3	19	0.00490166	0.58525516	0.52759136	0.38071817
cg09643398	BCL6;BCL(	3	0.02336655	0.73405783	0.71435833	0.60338238
cg10020892	BCL9	1	0.036552	0.56427006	0.57714287	0.41608098
cg00919591	BCR;BCR	22	0.0107931	0.55798233	0.59484734	0.36985141
cg13521842	BEGAIN	14	0.00570746	0.33317837	0.33438147	0.21601451
cg09970511	BEND6	6	0.00076471	0.75749498	0.62529706	0.52076768
cg00658449	BEYLA;BE	8	0.00442269	0.47969271	0.43029853	0.31150811
cg11519176	BGLAP	1	0.01546058	0.72291843	0.74022076	0.56850256
cg23729443	BGLAP	1	0.00491882	0.41125086	0.41252251	0.25261016
cg09996284	BGLAP	1	0.0046471	0.66372156	0.64370122	0.47632143
cg08466030	BICC1	10	0.00132756	0.88158084	0.86794119	0.73072655
cg22641201	BICC1	10	0.00961487	0.47215459	0.42243313	0.26161674
cg12342675	BICC1	10	0.00366179	0.87627663	0.88737015	0.77207337
cg24890964	BLCAP;BL	20	0.00311547	0.67149194	0.64154234	0.48406738
cg08901339	BLNK;BLN	10	0.02649037	0.57977153	0.57519076	0.45137266
cg04937184	BMP4;BM	14	0.00318584	0.50743932	0.48334922	0.35528847
cg09807875	BMPER	7	0.00084118	0.53487819	0.38454412	0.25466785
cg06024391	BPGM;BP	7	0.00126466	0.84018261	0.80794268	0.65976502
cg02983911	BPI	20	0.01507976	0.79159177	0.78274223	0.66192685
cg26534847	BPI	20	0.01077982	0.53923596	0.4878851	0.30587556
cg16451371	BPIL2	22	0.01011462	0.46287895	0.50599094	0.35448586
cg16185457	BRE;BRE;I	2	0.00521564	0.87694077	0.87515857	0.72564901
cg17416644	BRSK2	11	0.01870855	0.36900284	0.34725055	0.24520113
cg20092122	BST2	19	0.00076471	0.38225158	0.28790512	0.11657735
cg03505817	BTBD7	14	0.00337719	0.83255868	0.81791866	0.68063554
cg22198603	BTBD9;BT	6	0.01206219	0.7462964	0.77163424	0.6055132
cg22690339	BTBD9;BT	6	0.03033692	0.56318691	0.49926252	0.37513944
cg12238593	BTN3A3;I	6	0.01426008	0.68678989	0.61959172	0.51452368
cg08754149	C10orf11	10	0.00122881	0.85272256	0.81916788	0.7107117
cg03936104	C10orf41;	10	0.00349377	0.66871913	0.5976756	0.39340597
cg10627429	C10orf81	10	0.00126804	0.75650154	0.69650503	0.5777335
cg11204562	C10orf81	10	0.00071094	0.89649553	0.83977099	0.71752838
cg15834395	C10orf91	10	0.01011462	0.55190781	0.59482861	0.42093795
cg08200419	C10orf91	10	0.04868581	0.69212826	0.69499452	0.56393131
cg15818008	C11orf34	11	0.01020581	0.61273988	0.64821228	0.40681857
cg12490907	C11orf41	11	0.00704684	0.56579255	0.65269438	0.41562923
cg07188648	C11orf88;	11	0.00291636	0.49438523	0.47284491	0.28548883
cg03132202	C12orf56;	12	0.00820128	0.39370859	0.43839852	0.28573997
cg02873991	C12orf77	12	0.03532942	0.55975129	0.54783606	0.3899065
cg09166410	C13orf16	13	0.00122881	0.58253756	0.5417793	0.37991532
cg08990474	C13orf35	13	0.04967107	0.73512054	0.77681049	0.62887439
cg24169915	C14orf17	14	0.00287155	0.36558335	0.41456543	0.23031298

cg18709904	C14orf18:	14	0.02712361	0.65199223	0.82601497	0.45272342
cg00859280	C14orf70	14	0.00144775	0.24547231	0.22325091	0.12320716
cg03565081	C14orf93;	14	0.04980289	0.46988978	0.5013159	0.36162026
cg08274633	C15orf53	15	0.04953247	0.78503469	0.75134842	0.63233736
cg20678353	C15orf53	15	0.04357378	0.34257015	0.31297632	0.18837123
cg12609829	C15orf53	15	0.00509504	0.80219486	0.79058	0.54791815
cg27129984	C16orf38	16	0.01994073	0.44096108	0.45899917	0.33587402
cg02319067	C16orf54	16	0.0093099	0.48255893	0.52020271	0.32217499
cg26285698	C16orf54;	16	0.01678444	0.43530529	0.43706198	0.30407442
cg09944012	C17orf10:	17	0.00908203	0.84940294	0.82213227	0.72013419
cg25490145	C17orf10:	17	0.01414127	0.77029821	0.78717225	0.667714
cg21465176	C17orf10:	17	0.02077264	0.74332464	0.76147424	0.64007843
cg21683390	C18orf1;C	18	0.03488893	0.57504033	0.56294257	0.43092066
cg27638057	C18orf45	18	0.00073118	0.74204691	0.63827619	0.48124719
cg12571879	C19orf59;	19	0.01645625	0.56960132	0.58141189	0.41429508
cg16385583	C1orf150	1	0.01672645	0.62975938	0.68858391	0.48106897
cg17518550	C1orf150	1	0.03358212	0.63560822	0.6752687	0.53141686
cg19375403	C1orf174	1	0.02231558	0.71031655	0.77593499	0.55746738
cg04486839	C1orf192	1	0.00478595	0.81759101	0.74247178	0.64138618
cg21118367	C1orf21	1	0.01467615	0.28584684	0.33497414	0.14198957
cg03320492	C1orf228	1	0.03021634	0.43762173	0.39328501	0.27533803
cg16684817	C1orf38;C	1	0.03055957	0.78114794	0.76918518	0.63206986
cg05643188	C1orf53	1	0.02389893	0.60316511	0.61359838	0.47738497
cg26788737	C1orf55	1	0.01311156	0.41485362	0.38705775	0.26323276
cg24931346	C1QB	1	0.00286125	0.67541019	0.68099469	0.57284453
cg18527716	C1QTNF1;	17	0.01980822	0.71932304	0.67772866	0.50895141
cg05003411	C1QTNF7;	4	0.00154265	0.8531175	0.83534301	0.70599176
cg15013617	C20orf14:	20	0.00100445	0.35524926	0.31724761	0.16181861
cg18157896	C20orf14:	20	0.0015244	0.44196217	0.49639206	0.23784693
cg05890956	C20orf18(	20	0.01390012	0.66534898	0.67297318	0.53534182
cg14065109	C21orf57	21	0.02111228	0.58057543	0.60872286	0.46979147
cg08416522	C21orf7	21	0.00796332	0.56415934	0.62605573	0.4385059
cg11466708	C22orf43	22	0.00943058	0.63738401	0.75453316	0.42539637
cg19942731	C22orf9;C	22	0.03866096	0.64316586	0.59905336	0.49420003
cg18565216	C2CD2;C2	21	0.00140256	0.26498695	0.29442846	0.14955845
cg26174797	C2orf53	2	0.00317466	0.57489728	0.5565974	0.4149764
cg11130441	C2orf58	2	0.03119297	0.78184269	0.7809297	0.61908723
cg04499514	C3AR1	12	0.00385922	0.56642609	0.48155176	0.32061792
cg11118413	C3orf27	3	0.00379918	0.31096593	0.33007722	0.19198627
cg20749576	C3orf27	3	0.0131276	0.39429883	0.40282656	0.28558509
cg16217908	C3orf52	3	0.01782206	0.34594592	0.32607619	0.18191383
cg10224107	C5AR1;C5	19	0.00411808	0.42290849	0.42714321	0.26335334
cg08470180	C5orf24;C	5	0.00642271	0.54874588	0.52791236	0.41044887
cg04871807	C5orf27	5	0.00252833	0.84398824	0.83243724	0.7121007
cg00287773	C5orf32	5	0.0026461	0.61892842	0.5552687	0.31944221
cg03653573	C5orf56	5	0.00379486	0.48784989	0.43836989	0.26364349

cg15601361	C6orf105;	6	0.00066204	0.58601531	0.46808177	0.32175325
cg08421910	C6orf105;	6	0.00103447	0.83619067	0.76743756	0.64851469
cg01431340	C6orf186	6	0.03585699	0.45989752	0.41114419	0.30591348
cg00239660	C6orf191	6	0.00334886	0.75689132	0.70947953	0.60812423
cg19635401	C6orf204;	6	0.00414415	0.61687233	0.61893518	0.42505585
cg18997124	C6orf27	6	0.02680411	0.74771941	0.74781773	0.63562287
cg06824394	C7orf23	7	0.02763355	0.71029068	0.69180137	0.57697549
cg18377014	C7orf50;C	7	0.04178799	0.64762124	0.67033752	0.52809461
cg09453760	C7orf65	7	0.01442631	0.37851346	0.39523311	0.26689319
cg22454022	C8orf34	8	0.01276611	0.5034807	0.53454686	0.33181557
cg15947176	C8orf46	8	0.00070134	0.54257107	0.44574833	0.31331725
cg09933836	C8orf75	8	0.00211366	0.24688185	0.36737581	0.14154295
cg06745083	CA5A	16	0.00663164	0.52298555	0.60027417	0.421673
cg21039723	CAB39;CA	2	0.0057272	0.71903287	0.71215101	0.58035683
cg11847933	CABC1	1	0.00798815	0.53727144	0.53787767	0.41098108
cg19671694	CABLES2	20	0.00820044	0.76430961	0.72842522	0.62390353
cg26224018	CACHD1	1	0.01488167	0.80358603	0.81477344	0.69919304
cg14140691	CACNA1B	9	0.00113894	0.41667337	0.39614934	0.29302745
cg14022794	CACNA1B	9	0.0102299	0.33806225	0.33929204	0.22262267
cg02468320	CACNA1C	12	0.00543797	0.65364734	0.6547712	0.37450534
cg16728539	CACNA1C	12	0.00306113	0.68848906	0.69799726	0.44679431
cg01637090	CACNA1H	16	0.001181	0.27493695	0.27188236	0.1481682
cg07890233	CACNA1H	16	0.00739749	0.7750812	0.73867151	0.58303792
cg02041885	CACNA1I;	22	0.00714351	0.52485812	0.5197878	0.40246278
cg03364832	CACNA2D	3	0.00943797	0.69458686	0.61243886	0.48997366
cg10791541	CACNA2D	3	0.00629716	0.6244516	0.70035929	0.52171501
cg25482874	CACNG6;(	19	0.00168532	0.29073246	0.29456007	0.16999881
cg19521693	CACNG8	19	0.00517725	0.47280005	0.47865937	0.35578791
cg01293740	CACNG8	19	0.00209769	0.42947405	0.42725552	0.28381314
cg03505501	CADM1;C	11	0.02450704	0.62787589	0.74865926	0.5179695
cg13716443	CADPS;CA	3	0.00206218	0.85156663	0.81286125	0.70277273
cg02753187	CALCOCO	17	0.01725665	0.61185305	0.66987702	0.49130026
cg01077501	CALD1;CA	7	0.00233439	0.8505807	0.83566009	0.72865508
cg25937598	CALHM3	10	0.04980289	0.71431057	0.72811456	0.60425314
cg26957187	CALN1;CA	7	0.00779924	0.52724049	0.54890364	0.34563557
cg12273284	CAMK1D;	10	0.00277534	0.54916517	0.49641014	0.32924321
cg20994118	CAMK1G	1	0.00854392	0.70070897	0.70101035	0.56561032
cg17541002	CAMKK2;(	12	0.00101815	0.76798101	0.73832334	0.4408899
cg02275084	CAMTA1	1	0.00073118	0.75395184	0.64972516	0.52333308
cg21583016	CAMTA1	1	0.00168675	0.87492716	0.81053994	0.65798569
cg21410633	CAP1;CAF	1	0.0437543	0.58123219	0.60501021	0.47538547
cg05820435	CAPZB	1	0.00074016	0.73792727	0.63783507	0.48234674
cg06360604	CAPZB	1	0.00065949	0.80132484	0.63356007	0.42553169
cg12424867	CAPZB	1	0.01318677	0.75123738	0.71644946	0.61581021
cg01832549	CAPZB	1	0.0035878	0.80147146	0.79217617	0.64733927
cg15136975	CAPZB	1	0.0173664	0.48886464	0.45964657	0.30245991

cg00968167	CAPZB	1	0.00372084	0.44345812	0.46594429	0.28427575
cg09077096	CARD11	7	0.01935097	0.73686867	0.72342527	0.58100677
cg13775050	CARD16;(	11	0.01365382	0.72888501	0.80756156	0.60396824
cg26818786	CARD6	5	0.03951832	0.3624055	0.40251483	0.25532542
cg15930041	CASP14	19	0.02026622	0.68065624	0.68234598	0.53916395
cg08469834	CASP4	11	0.00195644	0.21003236	0.1974186	0.09068873
cg24410214	CASP8;CA	2	0.00564684	0.29942957	0.36007504	0.18869609
cg14985891	CASQ2	1	0.01980843	0.44614833	0.4740853	0.30951617
cg09729182	CASR	3	0.01828536	0.43610878	0.40488597	0.26218961
cg10202975	CAST;CAS	5	0.04937176	0.76703065	0.77783279	0.65202322
cg05057515	CBFA2T3	16	0.00772025	0.51804378	0.56415491	0.41454635
cg07057235	CBFA2T3	16	0.02077264	0.40067603	0.41082948	0.29102224
cg23965590	CBL	11	0.00405622	0.38228651	0.38978547	0.26724229
cg01544580	CBX1	17	0.03729142	0.76742955	0.77935986	0.64715193
cg19283806	CCDC102I	18	0.00429569	0.68095441	0.68205352	0.53221077
cg21616935	CCDC11	18	0.0065693	0.62328473	0.60128934	0.45184604
cg26539468	CCDC111	4	0.00901377	0.43851622	0.46693912	0.30386558
cg07220903	CCDC12	3	0.00261671	0.59812066	0.54424589	0.31616878
cg02943305	CCDC129	7	0.00317348	0.50824763	0.51602264	0.36363929
cg03100209	CCDC155	19	0.00981397	0.33486378	0.36086506	0.21390056
cg07997596	CCDC27	1	0.01998122	0.48006359	0.49881719	0.37803204
cg02708758	CCDC33	15	0.00159601	0.88203477	0.80136192	0.69538634
cg15507901	CCDC54	3	0.02096205	0.6887436	0.77605143	0.5378439
cg12504696	CCDC60	12	0.01463726	0.73495504	0.71809122	0.60266237
cg08365738	CCDC80;(	3	0.00220808	0.70790098	0.70076806	0.39836231
cg17118262	CCL1	17	0.01294921	0.60032096	0.63744582	0.49833862
cg17864156	CCL2	17	0.0109037	0.44147689	0.44206342	0.31787408
cg17315454	CCL24	7	0.01464559	0.57487711	0.61142493	0.44547889
cg12788666	CCL24	7	0.0113009	0.59746211	0.64462699	0.49661201
cg02932854	CCL24	7	0.00188499	0.45157651	0.3937451	0.29318807
cg11303839	CCL26	7	0.00127437	0.78064139	0.7137259	0.4970382
cg18407309	CCL3	17	0.00332487	0.58449609	0.67121572	0.47037987
cg02483931	CCL5	17	0.00485896	0.44789174	0.43939225	0.30867852
cg08656816	CCL5	17	0.00076043	0.4718691	0.37994239	0.218512
cg01636591	CCL8;CCL!	17	0.0022221	0.4023632	0.39787768	0.2373668
cg03744842	CCM2;CCI	7	0.01729366	0.37509514	0.34753103	0.19892048
cg24110396	CCNY	10	0.00288962	0.74880478	0.74567262	0.58817529
cg07740894	CCNY;CCN	10	0.00187952	0.79242958	0.72216165	0.62152722
cg04110105	CCR2;CCR	3	0.02562377	0.62349814	0.60277248	0.47354973
cg23817981	CCR4	3	0.00635384	0.55624735	0.48427669	0.31220327
cg26960939	CCR7	17	0.00543797	0.53474273	0.52301578	0.38778568
cg16047279	CCR7	17	0.00068904	0.73294808	0.67981747	0.48275651
cg07248223	CCR7	17	0.00186784	0.75354648	0.72966024	0.48801937
cg18599081	CCRL2;CC	3	0.00207996	0.80123592	0.75107945	0.54248522
cg23350385	CCRL2;CC	3	0.00233734	0.65494052	0.61691372	0.3857278
cg19850333	CCRL2;CC	3	0.03477069	0.33349762	0.36226595	0.23071381

cg01606773	CD109;CD	6	0.0009502	0.68845728	0.50740889	0.39048924
cg17638468	CD200R1;	3	0.0412314	0.64049063	0.68698142	0.52297891
cg15743985	CD22	19	0.01398104	0.66165132	0.66561496	0.54994372
cg19090861	CD248	11	0.00214604	0.47372158	0.43693971	0.28053619
cg14910296	CD276;CD	15	0.00563159	0.81258976	0.74711159	0.64132218
cg13790288	CD28	2	0.00531242	0.6314714	0.65304585	0.42678112
cg04098585	CD28;CD2	2	0.01016149	0.72896284	0.75992308	0.54192717
cg03069736	CD300C;C	17	0.00606409	0.56019493	0.55751864	0.44529113
cg03533256	CD300E	17	0.03212425	0.61388565	0.5665284	0.39701891
cg14438208	CD300E;C	17	0.00479296	0.83790898	0.83075807	0.71327977
cg00873351	CD300LB	17	0.01291835	0.35143735	0.38805771	0.24495935
cg14075731	CD300LB;	17	0.01465218	0.60912997	0.630515	0.49453256
cg20293352	CD34;CD3	1	0.02595684	0.42914676	0.40669516	0.29519543
cg06601993	CD36	7	0.00685049	0.7479896	0.71896354	0.57812259
cg22908581	CD4	12	0.00674227	0.39757035	0.42158056	0.29383624
cg14082886	CD44;CD4	11	0.00100019	0.84660752	0.82115164	0.62062266
cg20868817	CD48	1	0.00697437	0.45565074	0.47707838	0.29913697
cg19408145	CD48	1	0.00768774	0.42053602	0.46652614	0.28773063
cg10516886	CD53;CD5	1	0.03471543	0.57117468	0.59803288	0.47094464
cg17240760	CD53;CD5	1	0.00535942	0.54004491	0.62277682	0.39865181
cg23903301	CD59;CD5	11	0.00065949	0.73261101	0.62806514	0.40146884
cg04188351	CD59;CD5	11	0.00737637	0.40825608	0.40172754	0.23473473
cg07440264	CD59;CD5	11	0.00073265	0.81444089	0.74622864	0.50869879
cg13014558	CD6	11	0.04126303	0.66414632	0.72068944	0.55594461
cg11848173	CD63;CD6	12	0.00276502	0.6244759	0.56797265	0.36338382
cg03677492	CD7	17	0.00225589	0.33710427	0.32196852	0.21676232
cg06692929	CD7	17	0.01294921	0.37329093	0.38458916	0.2467133
cg21108085	CD82;CD8	11	0.00334886	0.38051972	0.28852049	0.1723684
cg02945019	CD84	1	0.03502085	0.61871304	0.58206597	0.47462338
cg01878435	CD86	3	0.01561358	0.35563612	0.39730621	0.23908598
cg07341609	CDC42EP1	22	0.00357777	0.72667447	0.67046649	0.40152496
cg09220326	CDCP1;CD	3	0.00295256	0.38073371	0.34176213	0.18765659
cg02973961	CDH11	16	0.01068625	0.77807687	0.77642529	0.58668492
cg09415485	CDH13	16	0.00076471	0.60723854	0.48298518	0.35494659
cg04304802	CDH23	10	0.00855749	0.48918974	0.51062965	0.30071916
cg04005943	CDH23;CD	10	0.02054758	0.60651019	0.63532321	0.44255092
cg16325421	CDH4	20	0.00253153	0.59868964	0.55648736	0.43984498
cg02078525	CDH5	16	0.01921519	0.44023763	0.44909432	0.33671581
cg02723617	CDK5;CDK	7	0.0012504	0.40867004	0.36645473	0.26532407
cg05101437	CDK6;CDK	7	0.00113577	0.55413764	0.51952008	0.20601403
cg23342625	CDKAL1	6	0.00078196	0.88099025	0.84749215	0.73558172
cg06321045	CDSN;PSC	6	0.01660506	0.73766631	0.7467555	0.63665508
cg03545404	CDX1	5	0.00486208	0.62197308	0.59659817	0.47347875
cg23241878	CEACAM2	19	0.04639674	0.44149078	0.44858705	0.31860736
cg21529807	CEACAM4	19	0.0132124	0.37025954	0.38645908	0.26225128
cg06726231	CECR1;CE	22	0.00073118	0.5649696	0.47412501	0.34090112

cg09397359	CELSR3	3	0.02049456	0.74292029	0.76709496	0.61894038
cg13237068	CENPJ	13	0.01486263	0.79211272	0.8419998	0.64966869
cg26542660	CEP135	4	0.0070758	0.48669833	0.50838221	0.33101371
cg03447083	CES3	16	0.007634	0.35661255	0.33270997	0.23206374
cg12124018	CFB	6	0.00071044	0.72960893	0.63342106	0.53208573
cg05647929	CFC1B;CF	2	0.01856748	0.47338364	0.54084774	0.33718156
cg22092521	CFD	19	0.00722201	0.4629359	0.45498925	0.30438288
cg06633978	CFDP1	16	0.00852388	0.81572749	0.83720748	0.65477272
cg03974286	CFLAR;CF	2	0.02672791	0.46529698	0.48442548	0.3377011
cg10947827	CFLAR;CF	2	0.00775785	0.50986632	0.47736302	0.36023772
cg12944530	CFLAR;CF	2	0.02241528	0.78697526	0.84461659	0.6244754
cg23457862	CFLAR;CF	2	0.00311192	0.43206145	0.46739819	0.25345151
cg25198049	CGN	1	0.00293109	0.60425274	0.59318981	0.37846503
cg07596668	CGN	1	0.00713936	0.74191642	0.74428858	0.58312214
cg18670236	CHCHD6	3	0.00281579	0.78942874	0.7818668	0.48566654
cg25035059	CHD7	8	0.00733126	0.26797638	0.25480854	0.15327232
cg03355213	CHMP4B	20	0.00411861	0.5362709	0.67235888	0.43292872
cg11941300	CHRFAM7	15	0.0398235	0.80530545	0.81872201	0.69746251
cg04590119	CHRM2;C	7	0.01032661	0.74951441	0.717314	0.61264525
cg07157107	CHRNA6	8	0.01114245	0.39818147	0.41751697	0.27378347
cg27051318	CHRNA6	8	0.01442596	0.73221117	0.71007131	0.57643038
cg06523556	CHRNA6	8	0.01059488	0.44870015	0.41007438	0.2518158
cg26531231	CHRNA9	4	0.00077754	0.82116921	0.76593387	0.65947739
cg16861964	CHST11	12	0.0058315	0.44610494	0.39646667	0.23737362
cg00608661	CHST11	12	0.00087274	0.83627888	0.78010437	0.62203876
cg18664965	CHST13	3	0.00131453	0.53095147	0.44677513	0.30793167
cg13276580	CHST15	10	0.00317531	0.46525582	0.51019202	0.31702754
cg20388165	CHSY1	15	0.02917135	0.79800028	0.80964147	0.69687268
cg08327038	CIRH1A	16	0.00112626	0.54797343	0.45597028	0.3398472
cg02295216	CKLF;CKLI	16	0.00137441	0.48538929	0.39148717	0.20726125
cg13790426	CLCC1;CL(	1	0.00354163	0.53928747	0.56782995	0.38198093
cg08702344	CLDN12	7	0.02122541	0.73329174	0.75809817	0.60464737
cg13643452	CLEC14A	14	0.01485881	0.30467339	0.30065556	0.19692122
cg16125874	CLEC14A;	14	0.02611848	0.75692095	0.74023557	0.60320919
cg03217729	CLEC16A	16	0.03376554	0.59381923	0.63608428	0.48943682
cg08321576	CLEC2B;C	12	0.00317234	0.52457555	0.53146582	0.26767513
cg13300689	CLEC2D;C	12	0.00279059	0.2407009	0.24071826	0.12951814
cg03447908	CLEC3B	3	0.00804549	0.67983693	0.718942	0.45094702
cg18460107	CLEC4C;C	12	0.00092849	0.67525292	0.66249366	0.54303889
cg03619586	CLIC1	6	0.00405794	0.34843678	0.40229869	0.22838762
cg18434367	CLIC5;CLI(	6	0.0015244	0.77253877	0.78701521	0.6644535
cg13730618	CLIC6	21	0.02122541	0.478077	0.49982353	0.36756349
cg05239310	CLMN	14	0.00218429	0.58479712	0.53258785	0.28521166
cg02330500	CLMN	14	0.00252984	0.4808914	0.52181044	0.28581075
cg06745511	CLNK	4	0.00659076	0.34395415	0.3901613	0.1797272
cg00865429	CLNK	4	0.00086795	0.73142834	0.61378465	0.4622015

cg20143111	CLNK	4	0.02362077	0.73871149	0.76498189	0.61677799
cg00326231	CLTCL1;Cl	22	0.00243597	0.39679389	0.42090934	0.22830628
cg20471562	CNGA3;Cl	2	0.0198367	0.68014298	0.64019334	0.53998484
cg09988837	CNIH3	1	0.00908203	0.55796478	0.57148073	0.37541309
cg11812748	CNST;CNS	1	0.01857513	0.33026293	0.33759353	0.20398774
cg14131220	CNTFR;CN	9	0.00569156	0.70473114	0.67983429	0.46844575
cg02825887	CNTFR;CN	9	0.00122265	0.54313282	0.47824661	0.25352146
cg22878054	CNTN5;CN	11	0.02651696	0.74878614	0.75211697	0.64534903
cg20694241	CNTNAP3	9	0.0115206	0.53966987	0.5917219	0.43220273
cg00937175	COL18A1	21	0.00377649	0.812017	0.74028801	0.6393219
cg22809726	COL1A1	17	0.00073265	0.24799203	0.20147817	0.09102942
cg25026926	COL1A1	17	0.00072814	0.70442328	0.62087533	0.48176953
cg14700325	COL1A1	17	0.00065949	0.84869905	0.72002632	0.57511178
cg18618815	COL1A1	17	0.03450675	0.80260095	0.84365782	0.66985024
cg02186748	COL1A1	17	0.00258198	0.64073879	0.61347484	0.49608975
cg23348014	COL1A2	7	0.00492669	0.62292369	0.54796907	0.38844309
cg06299997	COL1A2	7	0.00084118	0.58437202	0.51309639	0.34267282
cg10368049	COL1A2	7	0.00230867	0.62314913	0.59604235	0.37135776
cg13079333	COL20A1	20	0.00411808	0.36900178	0.35319688	0.2206857
cg05008898	COL23A1	5	0.0102299	0.69481858	0.6891862	0.57501341
cg06485308	COL23A1	5	0.00168741	0.69987975	0.61460407	0.51417233
cg08760493	COL25A1;	4	0.00935896	0.70661928	0.67351653	0.48771816
cg10275917	COL28A1	7	0.00078196	0.60308378	0.49301873	0.35020113
cg04887675	COL4A1	13	0.02175305	0.59013401	0.57303207	0.43435123
cg22332589	COL4A2	13	0.00131674	0.78443005	0.85369225	0.63530136
cg04815713	COL4A2	13	0.00082513	0.8169724	0.77503229	0.65674258
cg18114040	COL4A2	13	0.02944061	0.76619222	0.81560231	0.63747854
cg10979567	COL6A3;C	2	0.01830967	0.78112922	0.78269358	0.6746039
cg21175685	COL8A1;N	3	0.0006776	0.79729294	0.73696433	0.59442685
cg00876141	CORIN	4	0.00883762	0.67573293	0.74417311	0.50885747
cg09182455	CORO1C	12	0.00930663	0.88914265	0.88290873	0.76059094
cg13836518	CORO2A;	9	0.00741048	0.35870262	0.33075844	0.22580048
cg08290628	CORO2B	15	0.00634238	0.83027977	0.80199744	0.67767618
cg10423607	CPA6;CPA	8	0.00652679	0.75906421	0.77169684	0.53185369
cg26426690	CPEB3	10	0.00302287	0.73710931	0.71612947	0.56669219
cg19464524	CPEB3	10	0.0033331	0.44166527	0.3817089	0.21027047
cg24238409	CPEB3	10	0.0034485	0.64998418	0.58288295	0.45180527
cg22881750	CPNE5	6	0.00149058	0.51200335	0.48318618	0.25527534
cg23756264	CPT1A;CP	11	0.03471543	0.6870948	0.75012509	0.5733755
cg00233633	CPVL;CPV	7	0.00758063	0.49398299	0.51988893	0.31690896
cg03073429	CPXM2	10	0.00732415	0.5915782	0.61151435	0.45564583
cg09709565	CREB3L1	11	0.00065949	0.72854159	0.48831049	0.32877579
cg01972879	CRHR2	7	0.03743475	0.72374828	0.71395635	0.60042054
cg10004114	CRLF1	19	0.02878476	0.78281331	0.77836372	0.63331323
cg00166343	CRLF3	17	0.01678972	0.78266895	0.80607648	0.65760295
cg16294414	CROCC	1	0.02205285	0.72469116	0.66649847	0.56170849

cg26369418	CSF2	5	0.00396568	0.42024914	0.47875559	0.27189541
cg20450123	CSGALNA	8	0.00149569	0.32373628	0.27511811	0.16353192
cg14854503	CSGALNA	8	0.0080564	0.7278286	0.71484487	0.55527596
cg06699216	CSGALNA	8	0.00523353	0.81124879	0.836456	0.66811609
cg14722693	CSGALNA	8	0.00065949	0.50590118	0.43980127	0.29009835
cg23014871	CSGALNA	10	0.00704122	0.7526066	0.70738006	0.57887872
cg09631495	CSMD1	8	0.01132073	0.89370845	0.87247603	0.73651357
cg20564969	CSMD1	8	0.00380612	0.35030258	0.345906	0.22076834
cg01136458	CSMD1	8	0.00520163	0.49947766	0.49646279	0.33556079
cg24759795	CSMD3;C:	8	0.00197986	0.39145451	0.38140137	0.18929312
cg21896766	CSMD3;C:	8	0.00171661	0.46495189	0.49406487	0.24630557
cg25581222	CSNK1G1	15	0.00121809	0.76821823	0.74775072	0.48033446
cg24168221	CST9	20	0.01162689	0.64733439	0.65876483	0.51978137
cg11897887	CST9L	20	0.03017877	0.51898681	0.73074418	0.31748232
cg18618429	CSTA	3	0.00602447	0.40653054	0.45646732	0.2565209
cg12386061	CTDSPL;C	3	0.01987695	0.30471973	0.35354826	0.20272211
cg06968859	CTNNA2;(	2	0.00246645	0.70530567	0.68843819	0.48492465
cg14698665	CTNND2	5	0.0138324	0.48627663	0.51010559	0.35119365
cg14436231	CTNS;CTN	17	0.01332867	0.3956247	0.36609165	0.25261357
cg03206537	CTSA;CTS.	20	0.00670577	0.63149721	0.6179396	0.46395909
cg01099512	CTSG	14	0.04155652	0.53387887	0.51769416	0.39039283
cg19792802	CTSW	11	0.01396719	0.50375659	0.50839515	0.35532571
cg08416661	CTSW	11	0.00756169	0.34387311	0.31956296	0.20278958
cg23679724	CTSZ	20	0.00066204	0.54990101	0.47005614	0.33279823
cg12787553	CTSZ	20	0.02125381	0.72487929	0.79201711	0.62156455
cg18563860	CTSZ	20	0.00686574	0.88705135	0.88522222	0.77303394
cg25913882	CUBN	10	0.00068904	0.87086474	0.79094467	0.68385921
cg22900075	CUEDC1	17	0.00368593	0.61448323	0.57984128	0.45563667
cg18940274	CUL1	7	0.00430896	0.9071466	0.90729625	0.79771048
cg23261443	CUX1;CU)	7	0.00159601	0.59768238	0.55972243	0.4231883
cg25113157	CUX2	12	0.00748137	0.45922377	0.47685154	0.29864848
cg08944961	CUX2	12	0.00531242	0.55925127	0.58930678	0.38206224
cg03296370	CX3CR1;C	3	0.00431388	0.31905974	0.32281174	0.19586292
cg18956547	CXCR1	2	0.00315265	0.31529558	0.29753638	0.16163571
cg25941354	CXCR2	2	0.02190777	0.58432417	0.6083344	0.4828086
cg12595667	CXCR4;CX	2	0.01093903	0.66884402	0.66509774	0.47411148
cg01257799	CXCR5	11	0.00499353	0.45738933	0.43833485	0.246397
cg04294383	CYFIP1;CY	15	0.00077582	0.91010143	0.87357533	0.76406216
cg23217940	CYFIP1;CY	15	0.00065949	0.77737033	0.66007542	0.53860856
cg24844518	CYFIP2;CY	5	0.01365337	0.47152873	0.63742048	0.33031976
cg20073007	CYP11B1;	8	0.00258141	0.36833447	0.38338769	0.2610053
cg07954324	CYP11B2	8	0.00186737	0.42518876	0.42071832	0.31789678
cg02162897	CYP1B1	2	0.00180551	0.84579382	0.80702024	0.62388312
cg20408276	CYP1B1	2	0.00132756	0.82685029	0.74285285	0.62778731
cg00565882	CYP1B1	2	0.01430792	0.49085131	0.51953163	0.32653454
cg06264984	CYP1B1	2	0.00190678	0.54582496	0.63461928	0.38198399

cg19852607	CYP2C18;	10	0.04540817	0.44564025	0.4192762	0.31832238
cg06579354	CYP2F1	19	0.0039383	0.62280924	0.6136033	0.45170709
cg17461336	CYP3A43;	7	0.00583531	0.66939792	0.65946071	0.4915224
cg21637761	CYTSB;CY	17	0.00065949	0.78598198	0.66418356	0.52121788
cg15602972	DACT3	19	0.02711474	0.64126807	0.6665247	0.50214141
cg19815574	DACT3	19	0.01191142	0.47980125	0.49439344	0.36749778
cg23976388	DAGLA	11	0.01870855	0.56762899	0.58042972	0.38057719
cg20242427	DAK	11	0.01507976	0.607377	0.56804277	0.42539229
cg25406518	DAK;DDB	11	0.00128725	0.58205257	0.52045686	0.41735522
cg21244322	DAPK2	15	0.00775785	0.79221706	0.76149033	0.52094359
cg09365002	DAXX;DA	6	0.0012838	0.83064072	0.78269357	0.63919099
cg09597022	DAXX;DA	6	0.0013522	0.81940959	0.77821079	0.62856974
cg07905975	DAXX;DA	6	0.00507632	0.73888945	0.67283703	0.52691119
cg24498636	DAXX;DA	6	0.00976408	0.56120802	0.50421604	0.36955061
cg22697517	DBF4B;DE	17	0.01860545	0.45697831	0.48096591	0.34210585
cg21572000	DCAF5	14	0.00626821	0.76356973	0.76071904	0.62200611
cg02575483	DCBLD1	6	0.01608519	0.67546752	0.69325948	0.52198319
cg08514408	DCK	4	0.00074036	0.5485202	0.4592838	0.29880117
cg23574583	DCLK1	13	0.0009934	0.77964848	0.72019838	0.58084985
cg15001032	DDC;DDC	7	0.01952894	0.36030745	0.41996503	0.25902559
cg05883128	DDX60	4	0.00334901	0.53099938	0.5539299	0.38083482
cg11721464	DEDD2	19	0.0231425	0.69025827	0.72836722	0.5144764
cg14189583	DEDD2	19	0.01864666	0.5545901	0.5754352	0.40330564
cg10076006	DEFB1	8	0.03060322	0.34260922	0.35156369	0.2165148
cg01958189	DEFB125	20	0.02328473	0.63443738	0.61697153	0.51230764
cg12807628	DEFB135	8	0.00329066	0.34451715	0.33965728	0.23526112
cg05226729	DEFB135	8	0.00227382	0.85030755	0.80963851	0.67159025
cg22743884	DEFB135	8	0.00474032	0.78374076	0.80609644	0.64791549
cg02920600	DGKI	7	0.00065949	0.73533173	0.5726098	0.36440879
cg07092212	DGKZ;DGI	11	0.00958999	0.72346978	0.71644359	0.58042022
cg01132064	DGKZ;DGI	11	0.03166005	0.69727186	0.71310571	0.58439993
cg02211741	DGKZ;DGI	11	0.02775429	0.72766835	0.74836539	0.62512997
cg10177197	DHCR24	1	0.00065949	0.88453569	0.78999081	0.61279193
cg17475467	DHCR24	1	0.00065949	0.72747816	0.55898914	0.45896345
cg13624631	DHRS7	14	0.00127238	0.29136533	0.23274168	0.10703781
cg22129276	DHRS9;DI	2	0.0138324	0.57011999	0.61419524	0.43068663
cg04097334	DIRC1	2	0.02251946	0.48658948	0.50174271	0.356841
cg07328664	DIS3L2	2	0.00073118	0.84169812	0.77794015	0.60529875
cg08467371	DIS3L2	2	0.00442269	0.48031209	0.42813781	0.32767412
cg00550040	DKFZp566	19	0.00218799	0.70720867	0.68269866	0.52313756
cg27395342	DLC1;DLC	8	0.00073118	0.48784054	0.39967409	0.24223052
cg15681239	DLEC1;DL	3	0.00310177	0.79946616	0.75795269	0.49569484
cg03439898	DLGAP2	8	0.04175282	0.81032526	0.85121986	0.68590944
cg05455971	DLGAP2	8	0.00968951	0.75955691	0.79161733	0.65236992
cg09730801	DLX4;DLX	17	0.00196226	0.66158075	0.69637198	0.54554151
cg13619074	DMRT1	9	0.01399795	0.55942524	0.56114632	0.44735261

cg14377594	DMRT2;D	9	0.00232775	0.68741532	0.68441192	0.52358071
cg12626882	DNAH9;D	17	0.00427549	0.66556294	0.63216023	0.48214924
cg07993586	DNAJB6	7	0.0023359	0.9212064	0.91725212	0.7648115
cg22913903	DNAJC22	12	0.00293735	0.41690359	0.38583749	0.26107104
cg21763058	DNAJC22	12	0.0049312	0.42787917	0.42301367	0.27673016
cg07028869	DNAJC22;	12	0.00244246	0.40997584	0.39604919	0.23414753
cg07197831	DNAJC5G	2	0.01004931	0.52833852	0.52544551	0.3771993
cg15503081	DNASE2	19	0.03129419	0.58769868	0.6045772	0.45136961
cg07802362	DNM3;DN	1	0.00077468	0.8380504	0.76183423	0.62402249
cg00050692	DNMT3A;	2	0.00561533	0.83822326	0.84527095	0.64245602
cg03752935	DOCK1	10	0.04380272	0.64476312	0.72122047	0.52685471
cg14939821	DOCK1;FA	10	0.00445086	0.6455142	0.67669641	0.42345988
cg05363955	DOCK10	2	0.00112617	0.86853997	0.80061746	0.64374314
cg25012185	DOCK10	2	0.00499353	0.69882432	0.6857322	0.5727209
cg11394600	DOCK4	7	0.028008	0.70394974	0.76107751	0.56984089
cg06870470	DOCK6	19	0.00441677	0.33707606	0.32350148	0.18158787
cg16146353	DPP10	2	0.02115736	0.70832329	0.70886599	0.58704689
cg05868547	DPP10	2	0.00958772	0.7814168	0.7871094	0.66475889
cg02926033	DPP6;DPF	7	0.00590146	0.69419786	0.70051056	0.58388847
cg26187031	DPYSL2	8	0.00065949	0.71778905	0.61989535	0.40373128
cg17759095	DST	6	0.0036184	0.75028207	0.70434671	0.51568023
cg20236089	DTNBP1;D	6	0.0012838	0.31543958	0.2542831	0.12665072
cg07946977	DUSP10;D	1	0.01860545	0.81262065	0.8431616	0.66544428
cg27449114	DUSP28	2	0.00614688	0.35614782	0.37009716	0.23452082
cg09357462	DUSP5	10	0.0008696	0.65683182	0.57021289	0.43396087
cg02927679	DUSP5	10	0.00109472	0.5729151	0.49487233	0.38170037
cg10080966	DUSP5	10	0.00074016	0.42033312	0.31440056	0.20785763
cg03290131	DUSP5	10	0.00407141	0.42517429	0.38474389	0.23782813
cg10077746	DUSP6;DU	12	0.01258987	0.45167667	0.37073953	0.26455177
cg27365701	DUSP6;DU	12	0.01956069	0.65730051	0.67457593	0.47135752
cg17740822	DUSP6;DU	12	0.00290723	0.31893907	0.31459003	0.17884799
cg05769889	DUSP6;DU	12	0.00186249	0.31968312	0.32805432	0.16990568
cg13460297	DYNC1I2	2	0.00471011	0.81454768	0.80491028	0.68969468
cg17726575	E2F6;E2F6	2	0.01678444	0.67889765	0.72451375	0.56745208
cg02930866	EAF1	3	0.00217555	0.61061816	0.60779696	0.3318631
cg21211213	EBF1	5	0.00457097	0.30965325	0.30066929	0.15813753
cg00210856	EBF1	5	0.02001663	0.78586782	0.80159828	0.62069037
cg11898646	EBF1	5	0.02743413	0.80316736	0.8151961	0.66606609
cg00594129	EBF1	5	0.01170233	0.4046281	0.38178509	0.24534808
cg07616828	EBF2	8	0.0018234	0.42831547	0.37860763	0.2733012
cg05487589	EBF2	8	0.00337574	0.59687452	0.60407407	0.34624703
cg03497907	EBF3	10	0.01966537	0.62956975	0.66857089	0.48936475
cg21005416	ECE1;ECE	1	0.00575836	0.55120602	0.48671609	0.35815199
cg13527631	ECE2	3	0.00393823	0.50966188	0.49493727	0.39351409
cg16386293	ECM1;ECI	1	0.000777	0.59579263	0.53958331	0.39986281
cg09809672	EDARADD	1	0.04228846	0.54646223	0.56010226	0.42133899

cg16264526	EDNRA;ED	4	0.00242276	0.73931522	0.65338508	0.49810132
cg10112943	EDNRA;ED	4	0.0008696	0.71277616	0.6549594	0.47327407
cg05618426	EDNRA;ED	4	0.02368354	0.44570168	0.45574663	0.3176319
cg00133624	EFCAB4B;	12	0.00112626	0.36842081	0.45783051	0.2229479
cg03224812	EFCAB4B;	12	0.0008887	0.62426529	0.46525377	0.3224099
cg25978218	EFHD2	1	0.01342353	0.84677376	0.87577401	0.70410035
cg14036402	EFHD2	1	0.00429883	0.71682149	0.78670741	0.58001526
cg18919106	EFR3A	8	0.01783758	0.37078543	0.37312764	0.26454991
cg12476044	EGFLAM;I	5	0.00177922	0.78470128	0.74991675	0.63402071
cg08810842	EGR3	8	0.00507512	0.64199942	0.79244196	0.47382409
cg05150973	EHD1	11	0.00073265	0.87480631	0.77773802	0.6551677
cg17344040	EHD1	11	0.0070502	0.58004463	0.55411681	0.39869713
cg11321083	EHD1	11	0.00398606	0.5137923	0.49240475	0.30466807
cg00648955	EHD3	2	0.0050226	0.62415955	0.57099191	0.46483949
cg10071442	EHMT2;EI	6	0.01861071	0.4587768	0.42352502	0.25003129
cg08822118	EIF4G1;EI	3	0.0012278	0.63238311	0.5926181	0.45163098
cg01440489	ELF1;ELF1	13	0.0069524	0.50600778	0.50194698	0.27545861
cg02632314	ELF1;ELF1	13	0.00897906	0.56891948	0.55584215	0.36607188
cg15050051	ELFN1	7	0.00338778	0.72818048	0.70951871	0.58477639
cg00107982	ELFN2	22	0.00129726	0.29884823	0.31298656	0.16398276
cg01291854	ELK3	12	0.00066204	0.8149677	0.69396528	0.51327842
cg26245202	ELK4;ELK4	1	0.00374779	0.62831154	0.50663323	0.38759438
cg01119452	ELMO1	7	0.00433527	0.40919775	0.49980848	0.27434451
cg08314781	EML6	2	0.00093631	0.6695751	0.70925423	0.56557598
cg19009132	EMP1	12	0.00436286	0.81048248	0.77657415	0.66058079
cg16353851	ENC1	5	0.0019386	0.44472361	0.40607837	0.23832354
cg11978634	ENOX1;EN	13	0.00065949	0.82550801	0.69537515	0.5625867
cg10332003	ENPP6	4	0.00140062	0.83932326	0.82656141	0.7011603
cg20623350	ENTHD1	22	0.04089782	0.47684314	0.50936844	0.35769913
cg21211882	EPB41L1;I	20	0.00425638	0.60133776	0.53813954	0.36313695
cg06459104	EPB41L3	18	0.03879786	0.41188847	0.42767749	0.29228836
cg13678551	EPB41L4B	9	0.00194185	0.44600637	0.3979927	0.29533345
cg03292675	EPB49;EP	8	0.00128133	0.41707676	0.43582684	0.26787178
cg10835083	EPHA4	2	0.00135325	0.76686536	0.74306799	0.57606416
cg01970336	EPS15	1	0.00167443	0.63824537	0.61799603	0.4521078
cg03507641	ERC1;ERC	12	0.01024014	0.34454371	0.31202731	0.19049806
cg03927470	ERCC4	16	0.00116059	0.89429722	0.87748837	0.75831561
cg18413706	ERG;ERG;	21	0.00344958	0.5743783	0.55442093	0.35544112
cg22709362	ERI3	1	0.00081789	0.89101771	0.8534088	0.71146052
cg13582028	ERICH1	8	0.03174689	0.53415318	0.54107161	0.40461554
cg21211780	ESPN	1	0.00180652	0.77570752	0.71172949	0.55492324
cg00917101	ESPN	1	0.00087864	0.48223129	0.48471029	0.29353567
cg04003327	ESPNL;SC	2	0.01446513	0.75445455	0.79060603	0.5691516
cg06574296	ESPNL;SC	2	0.0088828	0.74111879	0.76269878	0.59788327
cg01395667	ESPNP	1	0.00155548	0.48890002	0.40906903	0.28802688
cg22364729	ESPNP	1	0.00134844	0.7952736	0.76612004	0.66348423

cg17984400	ESPNP	1	0.03124896	0.53048247	0.50514154	0.32735925
cg03037684	ESR1;ESR1	6	0.00284687	0.49681409	0.4895599	0.34429303
cg07059469	ESR1;ESR1	6	0.00137441	0.63438135	0.63845045	0.35286663
cg21464115	ESYT3	3	0.00201167	0.67194954	0.66316191	0.54791766
cg21223094	ETV6	12	0.00565829	0.31684414	0.31436988	0.18409884
cg11655490	ETV6	12	0.00152405	0.70054332	0.69802829	0.51031703
cg05582310	EVC	4	0.00993335	0.83353815	0.8222452	0.7180203
cg20992708	EVC2;EVC	4	0.01647542	0.76780273	0.78217368	0.6273644
cg17526483	EVX1	7	0.00857669	0.74972089	0.80782018	0.63538099
cg04922154	EVX1	7	0.00841345	0.66632919	0.70876429	0.49964499
cg03056526	EVX1	7	0.01792334	0.72036781	0.71651503	0.5333839
cg13677149	EVX1	7	0.00737637	0.77323519	0.77654752	0.64809298
cg08483376	EVX1	7	0.00088355	0.53025855	0.44351967	0.23147624
cg01357429	EVX1	7	0.00467797	0.537477	0.52378596	0.36054872
cg22964918	EVX1	7	0.0048797	0.66668678	0.67004474	0.47293744
cg01544270	EXD3	9	0.04601984	0.50222636	0.50447705	0.36894893
cg26008509	EXOC3L2	19	0.00072814	0.60502587	0.57273983	0.44815791
cg10679182	EXOC4	7	0.00149248	0.43804068	0.53395433	0.2334652
cg19350870	EXOC6B	2	0.03358008	0.72766218	0.72962401	0.61745012
cg15694987	EXT1	8	0.00070305	0.56636206	0.42940742	0.31540734
cg00328720	EXTL3	8	0.00154265	0.92746195	0.90853751	0.76853341
cg22871253	EZR;EZR	6	0.01058353	0.30490407	0.36213722	0.19339359
cg18642576	FA2H	16	0.01715772	0.85150703	0.82974327	0.71071996
cg06856840	FAH	15	0.01541996	0.65071143	0.65718859	0.45288729
cg22511455	FAIM3;FA	1	0.01155671	0.32235734	0.31952573	0.21140397
cg15414028	FAM107B	10	0.00193389	0.29120979	0.26990367	0.15974691
cg14170976	FAM110A	20	0.04251752	0.65534498	0.73586423	0.55214203
cg06547285	FAM113B	12	0.00065949	0.75137917	0.63096213	0.41440076
cg25123566	FAM113B	12	0.0012504	0.67714055	0.63117211	0.32298972
cg18951135	FAM126B	2	0.04818625	0.66239167	0.66309256	0.5603947
cg17320378	FAM129C	19	0.00180652	0.56697953	0.49348521	0.35375916
cg01403030	FAM154A	9	0.00552563	0.65858991	0.72562402	0.47961341
cg02771117	FAM167A	8	0.00298303	0.35114114	0.30281708	0.16335302
cg06358322	FAM171A	10	0.02818877	0.84818424	0.84549945	0.70732039
cg13436110	FAM188B	7	0.00065949	0.87834485	0.82531924	0.62437272
cg12239580	FAM198A	3	0.001362	0.57543805	0.46706847	0.36141266
cg05006211	FAM19A5	22	0.00469519	0.61035161	0.54243846	0.41184173
cg14596108	FAM19A5	22	0.01044005	0.81676014	0.8978291	0.70983864
cg08859215	FAM20A	17	0.00121234	0.84084713	0.77687692	0.65682728
cg18069356	FAM20A	17	0.00078623	0.86880058	0.77132247	0.67029283
cg10183671	FAM20C	7	0.00954906	0.71584967	0.74206534	0.57813622
cg00167275	FAM35A;(	10	0.00450697	0.20627558	0.20308311	0.09824751
cg09054528	FAM38A	16	0.03236546	0.58261688	0.57481193	0.44592398
cg08798862	FAM38A	16	0.00079833	0.8882117	0.83160708	0.72909735
cg07091529	FAM49A	2	0.01686581	0.88136624	0.87513524	0.74965582
cg02187259	FAM49A	2	0.01055568	0.87874091	0.88372422	0.7443212

cg17485265	FAM50B	6	0.04568556	0.77697763	0.75423143	0.64396246
cg03935116	FAM60A;I	12	0.03826066	0.70698807	0.77221015	0.59442215
cg21937128	FAM63A;I	1	0.03235903	0.68365955	0.68248678	0.562816
cg27050407	FAM65B	6	0.00065949	0.82142701	0.67903784	0.52090702
cg08893087	FAM69A	1	0.00177922	0.6770526	0.64174228	0.44727928
cg16689937	FAM71D;	14	0.00077454	0.47571727	0.43878315	0.2415364
cg10624729	FAM73A	1	0.00065949	0.88124372	0.82575737	0.69586895
cg00831735	FAM83A;I	8	0.00078196	0.63583981	0.55222288	0.41418124
cg08256536	FAM83A;I	8	0.00184091	0.70830375	0.65518491	0.50237267
cg27649094	FAM83A;I	8	0.01390004	0.58395842	0.59424611	0.45579545
cg20283107	FAM91A1	8	0.00471011	0.7876937	0.78038835	0.62936559
cg22223119	FARP1	13	0.00319723	0.54925382	0.42718435	0.30750864
cg12570309	FARSA	19	0.00937895	0.47747108	0.45530878	0.34745565
cg07107113	FBLIM1	1	0.00147208	0.70971014	0.66714626	0.56652507
cg03531326	FBXO17;F	19	0.02763355	0.68818961	0.70862788	0.58185257
cg12897164	FBXO32;F	8	0.00066204	0.54638028	0.45203748	0.23106344
cg05904901	FBXO41	2	0.00548311	0.66823419	0.67992287	0.55726355
cg04973915	FBXW11;I	5	0.00214604	0.73710397	0.70497923	0.58121048
cg03192919	FBXW12;I	3	0.01272421	0.71443693	0.7916479	0.51507245
cg15565057	FCAMR;F(	1	0.01094414	0.59028136	0.61490805	0.41144992
cg09773499	FCER2	19	0.00545658	0.30145509	0.32149598	0.19884174
cg19103704	FCGBP	19	0.00183809	0.46076347	0.45171765	0.30991817
cg04384208	FCGR3A;F	1	0.01088059	0.6726408	0.65384202	0.54513454
cg26435281	FCGR3B	1	0.00438294	0.37120075	0.40496543	0.2181776
cg09340639	FCRL1;FCI	1	0.0196961	0.45651152	0.49507894	0.32049948
cg05777583	FCRL2	1	0.00099652	0.52578861	0.52495719	0.36593204
cg17134153	FCRL3	1	0.02042436	0.75311339	0.77256517	0.64190483
cg00160981	FCRLB	1	0.00913085	0.53025265	0.55191738	0.33487381
cg16174681	FER	5	0.00115308	0.68770863	0.62201517	0.34837985
cg20312012	FER1L5	2	0.02394548	0.57616214	0.51284294	0.38064037
cg20890440	FER1L6	8	0.00085205	0.80485053	0.78243172	0.62990319
cg15692360	FERMT1	20	0.01060969	0.57760676	0.59293567	0.44003317
cg13724550	FGF1;FGF	5	0.00065949	0.6082571	0.49387757	0.37003563
cg19746186	FGGY;FG(	1	0.0154863	0.82356675	0.82782092	0.64877329
cg03055021	FHAD1	1	0.00065949	0.83257155	0.7161279	0.60697776
cg00004700	FHL2;FHL	2	0.0015244	0.91516944	0.89553337	0.76736878
cg19586143	FHOD3	18	0.03451707	0.7046036	0.78154577	0.60412691
cg13447566	FIBCD1;FI	9	0.00154265	0.81454893	0.79838665	0.69398466
cg19652483	FILIP1	6	0.00101054	0.54327705	0.44189298	0.31611895
cg11795276	FLJ12825	12	0.03728999	0.4500313	0.44641223	0.33857531
cg00753676	FLJ13197	4	0.00279494	0.76872692	0.75753921	0.63250028
cg02832512	FLJ22536	6	0.00147518	0.79830155	0.73817189	0.52661496
cg05925497	FLJ32810	11	0.02362077	0.50144022	0.40545414	0.27543309
cg19025461	FLJ37453	1	0.00804549	0.74743913	0.7847745	0.57568857
cg02579959	FLJ42289;	15	0.00336646	0.55807459	0.55338048	0.39262304
cg11648730	FLJ42709;	5	0.00629918	0.69076567	0.71185222	0.58302696

cg08732466	FLJ42875;	1	0.01289231	0.80799201	0.81076234	0.70711356
cg06991974	FLJ42875;	1	0.00714351	0.57613013	0.56292529	0.34975285
cg07946633	FLJ42875;	1	0.0113406	0.53115555	0.49533026	0.35904781
cg20228731	FLJ43663;	7	0.00078729	0.86621758	0.80033869	0.68525508
cg15928106	FLJ43663;	7	0.00246645	0.78970721	0.70790689	0.55645393
cg02122540	FLJ43860	8	0.01729366	0.68288271	0.66637087	0.55274433
cg11251367	FMN2	1	0.00204505	0.24661626	0.44557345	0.08921483
cg13966628	FMN2	1	0.01789965	0.49159497	0.53167677	0.36862936
cg12805374	FMNL1	17	0.01563939	0.47961901	0.46588461	0.35037607
cg00612751	FMNL2	2	0.00615357	0.7666191	0.71810587	0.54476477
cg04070601	FMNL3;FM	12	0.00065949	0.50012345	0.43419367	0.30689446
cg02054252	FMNL3;FM	12	0.00407611	0.74357707	0.71913472	0.59193719
cg22803510	FNBP1L;F	1	0.00235711	0.64657455	0.60558789	0.47611572
cg07739841	FNDC1	6	0.00575247	0.84922921	0.78834046	0.64596476
cg11637968	FNIP2	4	0.00395998	0.72523528	0.60374623	0.49514331
cg14513071	FOXD2;FC	1	0.00160273	0.85905178	0.78642477	0.65307607
cg02490736	FOXF2	6	0.01760238	0.49673387	0.51243885	0.35110556
cg15141195	FOXF2	6	0.01672645	0.76155886	0.79537467	0.60925723
cg04261496	FOXK1	7	0.01672645	0.77619655	0.81198036	0.63246566
cg01808969	FOXK1	7	0.00138693	0.88123002	0.80425645	0.65127046
cg26958524	FOXL1	16	0.02944061	0.72127408	0.81580671	0.58039349
cg00584450	FOXL1;FO	16	0.02519732	0.61286938	0.70401699	0.46235244
cg10696351	FOXO1	13	0.01842786	0.28926986	0.31298777	0.1875835
cg02202169	FOXO1	13	0.0331983	0.49568071	0.49977507	0.38364827
cg06175008	FOXP1	3	0.00855208	0.72474152	0.81588917	0.53701806
cg17803993	FOXP1;FC	3	0.00920255	0.40546457	0.33979346	0.22970427
cg07368857	FOXP1;FC	3	0.0025887	0.87178049	0.84742708	0.71516447
cg25356639	FOXP1;FC	3	0.00554086	0.80381469	0.77538021	0.61350974
cg09611620	FOXP1;FC	3	0.00079224	0.86397298	0.82334936	0.62527626
cg13492245	FPGS;FPG	9	0.03450675	0.41983859	0.44303806	0.31048733
cg22152605	FPR1	19	0.00073547	0.70932794	0.65510871	0.49701643
cg09684112	FRMD4A	10	0.014833	0.74987947	0.75115003	0.63842574
cg23037642	FRMD4A	10	0.02999866	0.50674324	0.50809918	0.38649558
cg03355808	FRMD4A	10	0.00213077	0.50388943	0.4903739	0.36662984
cg11377213	FRMD4B	3	0.00345706	0.43653606	0.39616687	0.20320998
cg04987857	FRS2;FRS2	12	0.01927179	0.47129252	0.41818889	0.31636091
cg13672638	FSD1L;FSI	9	0.01287168	0.40942281	0.4523793	0.30181322
cg19005955	FSTL4	5	0.01495982	0.58468601	0.61154492	0.45652507
cg02544257	FUK	16	0.00065949	0.35962709	0.24638714	0.12119882
cg19348484	FURIN	15	0.01660506	0.53374554	0.54163037	0.34664128
cg13300301	FUT4;FUT	11	0.03729142	0.41192973	0.40865007	0.3041023
cg17682828	FXYD1;FX	19	0.00294511	0.61981801	0.54489482	0.38482506
cg08130572	FYN;FYN	6	0.00401824	0.65415962	0.66618574	0.42005481
cg11026604	GABRB3	15	0.02115736	0.7145215	0.71588405	0.57911156
cg03942042	GABRB3;G	15	0.00354864	0.71266899	0.69642564	0.56873185
cg10311132	GABRB3;G	15	0.00758031	0.37000582	0.43412191	0.24962293

cg24087944	GABRD	1	0.01495982	0.58571724	0.61063387	0.42471159
cg19816354	GABRG3	15	0.00286384	0.33195905	0.36607045	0.20123071
cg09205438	GABRG3	15	0.01970757	0.73751501	0.75166572	0.63260129
cg16741573	GALNT1	18	0.00798815	0.26534251	0.27490029	0.14641827
cg13579752	GALNT10	5	0.00093631	0.8718734	0.82871176	0.67457213
cg18311633	GALNT13	2	0.00597656	0.39797497	0.43135168	0.27546848
cg24308604	GALNT7	4	0.01580869	0.32508129	0.36180514	0.21976776
cg11334728	GALNT9	12	0.03738486	0.63397111	0.66054278	0.52242286
cg20076126	GALNTL1;	14	0.00074409	0.63383279	0.47490487	0.34570041
cg08202226	GATAD2B	1	0.01642344	0.59516026	0.54556352	0.41607812
cg03125765	GATSL3	22	0.00086166	0.4864048	0.41599685	0.27945908
cg19257864	GBA;GBA;	1	0.00209505	0.22006752	0.21309074	0.10588124
cg16387581	GCH1;GCI	14	0.00830789	0.7741615	0.78699023	0.63512171
cg18492943	GCK	7	0.00450197	0.68906111	0.69975428	0.53112415
cg13411789	GCNT2	6	0.01910846	0.38635959	0.41968378	0.26847287
cg05989312	GCOM1;G	15	0.01539932	0.43344811	0.38997543	0.2458252
cg04365123	GDF6	8	0.00317531	0.50678842	0.59036745	0.33689901
cg13002506	GDPD4	11	0.00203531	0.77012113	0.75099293	0.594173
cg08268692	GDPD4	11	0.00253904	0.74702621	0.68647446	0.57932436
cg16874185	GDPD5	11	0.00065949	0.89111905	0.83323972	0.70119055
cg01522592	GDPD5	11	0.00089487	0.80367556	0.76460781	0.61784648
cg09120267	GEFT;GEF	12	0.0016682	0.66809902	0.63803744	0.52801452
cg10922232	GGA3;GG	17	0.00680102	0.36241301	0.32079198	0.21769663
cg13053608	GHDC;GH	17	0.03889249	0.63342294	0.64704553	0.5211544
cg06306927	GHDC;GH	17	0.01677529	0.36036051	0.36378378	0.2498189
cg08468689	GHDC;GH	17	0.00912728	0.39727905	0.41160876	0.23897983
cg20795023	GIMAP1	7	0.00575746	0.40979315	0.40227838	0.226245
cg12210293	GIMAP1	7	0.01495982	0.55500705	0.5988186	0.41616594
cg08960815	GIMAP4	7	0.00812661	0.70230694	0.72769007	0.59493333
cg13043509	GIMAP5;(	7	0.02630407	0.36830403	0.39175244	0.26662901
cg07319528	GIMAP5;(	7	0.01379421	0.49988975	0.54070261	0.39509461
cg24249248	GIMAP5;(	7	0.01149175	0.39786828	0.44318634	0.29559062
cg15849098	GIMAP7	7	0.02205285	0.80120248	0.79790319	0.68225824
cg00051483	GIMAP7	7	0.00178025	0.41835527	0.42961111	0.1950867
cg24413842	GIMAP7	7	0.01302012	0.58966504	0.58560678	0.43807787
cg17643598	GIMAP7	7	0.00158181	0.48017994	0.49418804	0.21407614
cg25597797	GIPC3	19	0.00160273	0.38094729	0.36045365	0.23715087
cg07999953	GJB3;GJB:	1	0.00177922	0.69000943	0.62863697	0.50171749
cg03568673	GJB6;GJB(	13	0.03502867	0.7022227	0.72320983	0.59423774
cg12630277	GJD4	10	0.0156946	0.65123545	0.6552074	0.54740218
cg17215361	GLB1L2	11	0.01064552	0.82444835	0.77756526	0.67651371
cg06679990	GLI1;GLI1	12	0.00100298	0.62230547	0.59177813	0.48395255
cg13168314	GLIS1	1	0.00150566	0.45463344	0.40648468	0.24372591
cg24741609	GLIS1	1	0.0012297	0.45481856	0.41717005	0.23461788
cg14325112	GLIS3;GLI	9	0.00065949	0.76574498	0.62131397	0.44652121
cg11650648	GLP2R	17	0.00976037	0.70123265	0.67468124	0.56955323

cg22689909	GLRX;GLR	5	0.00094876	0.33604365	0.32410642	0.10240431
cg16677191	GLRX;GLR	5	0.00187198	0.70686785	0.69522524	0.3926717
cg03852144	GLRX;GLR	5	0.00104158	0.23137756	0.1893485	0.07052032
cg18131582	GLT25D2	1	0.0017637	0.72042692	0.71034082	0.49426651
cg27642528	GLUD1	10	0.01820811	0.63554105	0.63967205	0.4737773
cg18551877	GLYATL2	11	0.03009647	0.62308272	0.66823738	0.49667212
cg01708377	GMDS	6	0.0010175	0.81677593	0.77497059	0.61571004
cg27360210	GMFG	19	0.01317683	0.73949137	0.76363533	0.61190304
cg09569432	GMFG;GN	19	0.00792881	0.32870486	0.34370411	0.22463798
cg20650545	GMFG;GN	19	0.01891509	0.39739012	0.40898615	0.28677676
cg25418001	GNAI1	7	0.01884397	0.64946922	0.64784147	0.48033204
cg10309886	GNASAS	20	0.00868726	0.74289719	0.78427885	0.54375864
cg23699918	GNASAS	20	0.00628685	0.79267923	0.79357245	0.68109218
cg03030267	GNASAS;N	20	0.00198246	0.83218209	0.84661419	0.67629163
cg06736148	GNB5;GN	15	0.02766761	0.81002379	0.8041752	0.70308348
cg08038054	GNG11	7	0.00335524	0.31396558	0.29074993	0.16453973
cg17168836	GNG12	1	0.04382199	0.61640273	0.64964954	0.48933347
cg04603130	GNG7	19	0.00100445	0.23834901	0.27744335	0.11051859
cg10996111	GNL1	6	0.02168773	0.37700351	0.37972847	0.27024745
cg15058852	GNS	12	0.00205947	0.60577095	0.5082978	0.37021318
cg18963763	GOLGA7B	10	0.0012504	0.439796	0.38662309	0.28203146
cg02389590	GOLSYN;C	8	0.04868581	0.48994944	0.4909667	0.38468242
cg02357751	GP1BB;SE	22	0.01518408	0.7998773	0.81139933	0.69319915
cg16210718	GP1BB;SE	22	0.01044398	0.70900668	0.73460748	0.5779718
cg03263514	GP1BB;SE	22	0.01891633	0.77005598	0.78559171	0.62686145
cg07359545	GP1BB;SE	22	0.01497704	0.55014545	0.6416092	0.43681235
cg02565999	GPC6	13	0.00093001	0.87938449	0.86103784	0.72312519
cg09012091	GPLD1	6	0.00840516	0.7052782	0.68138109	0.4898322
cg19700872	GPR151	5	0.04358513	0.411672	0.42800068	0.2961319
cg25586410	GPR155;G	2	0.02911369	0.39975239	0.42940673	0.29150095
cg23078123	GPR177	1	0.00393823	0.70283698	0.67024046	0.54265206
cg16110541	GPR177;G	1	0.0129037	0.77509848	0.77264052	0.65095296
cg09626634	GPR183;U	13	0.00073118	0.59362576	0.47778363	0.35866682
cg05875421	GPR68	14	0.00109472	0.77616243	0.68514263	0.4332683
cg23859630	GPSM3	6	0.02389893	0.45166838	0.48593372	0.34048202
cg26170660	GPX5;GPX	6	0.02096205	0.44330671	0.51189137	0.32156341
cg12280901	GPX5;GPX	6	0.01074556	0.63658421	0.63707907	0.5251198
cg11241750	GRAMD3	5	0.04650849	0.40106049	0.40411644	0.26623108
cg03840259	GRAP2	22	0.03239984	0.68482093	0.72663096	0.58422232
cg26203383	GRAP2	22	0.00403972	0.69565603	0.71738599	0.4936983
cg19374752	GRASP	12	0.00146177	0.51218035	0.46864383	0.30630506
cg06611426	GRASP	12	0.00405622	0.50342082	0.49523911	0.34292777
cg12358041	GRB10;GI	7	0.0008696	0.56433622	0.46952868	0.3458087
cg13396607	GRIK2;GR	6	0.00861733	0.77046568	0.74891948	0.64198079
cg08106961	GRIK3	1	0.00233298	0.27017542	0.26705263	0.16218905
cg27179693	GRIK4	11	0.02614058	0.61231988	0.55929266	0.43049161

cg24030173	GRIN2D	19	0.01771787	0.65442821	0.67232888	0.53014661
cg15580458	GRK5	10	0.0097835	0.8184635	0.82552308	0.68873069
cg22834542	GRM5;GR	11	0.00886166	0.72020553	0.66446963	0.56281769
cg21032008	GRM7;GR	3	0.01561381	0.31407921	0.32107967	0.19147073
cg24294950	GRM8;GR	7	0.00223531	0.41332252	0.40287641	0.27247366
cg10469791	GRXCR1	4	0.00262855	0.58270925	0.59166475	0.42393085
cg05316065	GSDMC;G	8	0.00099274	0.29166797	0.27461978	0.17165741
cg08305799	GSR	8	0.03740936	0.6728709	0.71883244	0.54882913
cg04340502	GSTA3	6	0.01169892	0.69274772	0.72957646	0.55314252
cg27343684	GSTA3	6	0.00799281	0.74186636	0.790643	0.59357423
cg11566152	GTF2E1	3	0.00120369	0.79408101	0.73897708	0.63498925
cg18325315	GTF2IRD1	7	0.0157424	0.69226504	0.68818322	0.56939833
cg22579828	GTSF1L;G	20	0.02775429	0.5655793	0.57745524	0.44755435
cg24013213	GUCY1B2	13	0.04733286	0.73240526	0.77294703	0.61524563
cg13725803	GUCY1B2	13	0.04550442	0.47316729	0.49170636	0.35584941
cg24772388	GYPC;GYF	2	0.00243597	0.38983623	0.37928277	0.26589313
cg06745001	GYS1;GYS	19	0.00149268	0.48710964	0.42821559	0.31944822
cg18118198	GZMB	14	0.00293735	0.67215354	0.63726187	0.5190658
cg03875678	GZMB	14	0.00134844	0.34301969	0.31981402	0.1844652
cg16356013	H2AFY;H2	5	0.00070134	0.86998098	0.79532384	0.6113445
cg14418802	H6PD	1	0.00163361	0.77189114	0.74845446	0.59692309
cg03719032	HAPLN4	19	0.01116167	0.52389768	0.53254659	0.40147404
cg17484237	HAVCR2;H	5	0.02049456	0.69397415	0.73797766	0.56486301
cg15371617	HAVCR2;H	5	0.00317972	0.65279783	0.71463825	0.48269631
cg04938738	HCCA2	11	0.00913085	0.76505285	0.75665843	0.5914201
cg13376953	HCCA2	11	0.00569156	0.54014193	0.52062815	0.32632493
cg07717512	HCG22	6	0.01853332	0.65886324	0.68874711	0.51479573
cg05615856	HCG27	6	0.04213177	0.42495121	0.4066017	0.25388953
cg09470907	HCG4P6	6	0.03735894	0.49764637	0.55296918	0.34027296
cg21026460	HDAC11;H	3	0.00280852	0.88896379	0.89236618	0.77025395
cg05997414	HDAC4	2	0.01400131	0.62858733	0.63152338	0.42795434
cg09681977	HDAC4	2	0.0140889	0.58890703	0.68066367	0.4841595
cg26876664	HDAC4	2	0.00587915	0.73627854	0.78148815	0.63340489
cg00332048	HDAC9;HI	7	0.00264473	0.39716189	0.36146056	0.22804345
cg23280720	HECA	6	0.00081599	0.84190664	0.80080044	0.64106737
cg01213645	HECW1	7	0.00140256	0.87429125	0.81439488	0.70867163
cg08338478	HECW2	2	0.00616957	0.55990622	0.71087082	0.45139348
cg09907068	HECW2	2	0.00420965	0.85473677	0.82044715	0.66212746
cg11029367	HEG1	3	0.00168532	0.80202117	0.76743004	0.51623864
cg09317502	HEPACAM	7	0.01502763	0.45241327	0.49861541	0.31744774
cg02544502	HEPACAM	7	0.03954748	0.42429122	0.45962618	0.30973655
cg14091419	HERC2	15	0.02720807	0.79704363	0.77429799	0.67246093
cg24495350	HEXA	15	0.00103697	0.85190781	0.8207514	0.70704902
cg03301084	HEY1;HEY	8	0.00480767	0.44821018	0.38879018	0.26371521
cg15567428	HGF;HGF;	7	0.00257075	0.4533262	0.5132185	0.28319593
cg08449639	HIC2	22	0.01411406	0.460912	0.48526987	0.3478395

cg14277677	HIP1R	12	0.00065949	0.67006035	0.55314583	0.43265581
cg01171339	HIST1H2A	6	0.00911882	0.64005739	0.63874611	0.49309773
cg05519606	HIVEP2	6	0.00089487	0.59024512	0.44303326	0.31819946
cg17892259	HIVEP3;H	1	0.02448461	0.73456411	0.75945302	0.5709375
cg10054398	HIVEP3;H	1	0.00380612	0.77694393	0.78484729	0.59237099
cg13423554	HIVEP3;H	1	0.01488167	0.39137143	0.34252418	0.22790551
cg05595692	HK2	2	0.01817172	0.70660167	0.7515916	0.54086899
cg08039587	HLA-A	6	0.01023819	0.61970908	0.63821899	0.4761404
cg22731440	HLA-B	6	0.04638701	0.76065229	0.67699756	0.54620036
cg05281796	HLA-DOA	6	0.00617318	0.59545503	0.59263829	0.48588175
cg11451043	HLA-DPA1	6	0.00387262	0.36209366	0.47728437	0.24715171
cg12365667	HLA-DPB1	6	0.03771398	0.40241632	0.47045171	0.29254061
cg00334532	HLA-DQA1	6	0.00499353	0.82678654	0.80067079	0.69805545
cg10989981	HLA-DQB1	6	0.01891191	0.6370285	0.63545038	0.45460937
cg06499030	HLA-DQB1	6	0.00359037	0.35249456	0.35448962	0.21206405
cg25046571	HLA-G	6	0.03708299	0.60309544	0.58542461	0.29923641
cg15595495	HLA-G	6	0.03208512	0.79777695	0.80570074	0.67795463
cg18645081	HLX	1	0.01512979	0.53902898	0.5792546	0.36682087
cg25207224	HMGA1;H	6	0.03349395	0.73383898	0.7291254	0.60444084
cg02123091	HMGA2;H	12	0.00065949	0.85227524	0.74242699	0.63822142
cg02423080	HMGXB3	5	0.01678972	0.39596483	0.4160932	0.27817751
cg02753354	HMHA1	19	0.02917135	0.44733744	0.45967305	0.33796384
cg10655396	HMHA1	19	0.0106654	0.38765706	0.41368198	0.27037112
cg14478768	HMHA1	19	0.01021896	0.6040719	0.59010774	0.48432918
cg09560590	HMHB1	5	0.00503527	0.68943639	0.75513618	0.54359157
cg04225775	HMHB1;H	5	0.00434951	0.65403018	0.71178385	0.47557841
cg17573720	HOTAIR	12	0.01169892	0.43017841	0.47730912	0.27571824
cg18824990	HOTAIR	12	0.03561451	0.71239337	0.76369389	0.60511413
cg16006349	HOTAIR	12	0.00585213	0.79893975	0.84475854	0.64384193
cg08941355	HOXA1;H	7	0.0286529	0.79379967	0.81774308	0.68636621
cg20994254	HOXA11A	7	0.04928347	0.72089059	0.77132257	0.60181482
cg12147489	HOXA11A	7	0.01304683	0.4920637	0.44917127	0.29883642
cg06587155	HOXA11A	7	0.0457117	0.46047053	0.45764929	0.34836561
cg26511321	HOXA7	7	0.01092179	0.437881	0.45995443	0.30423485
cg00524443	HOXB3	17	0.00553981	0.50200171	0.48141577	0.3693323
cg10165801	HOXB3	17	0.01980843	0.56460282	0.53190528	0.41557066
cg04014328	HOXB4	17	0.00816896	0.55346557	0.51445428	0.40151253
cg10366797	HOXC4	12	0.02956308	0.80240054	0.84952624	0.66507806
cg26201952	HOXC4;H	12	0.00521564	0.60551862	0.7144427	0.48177115
cg05992786	HOXC4;H	12	0.03346287	0.7115689	0.71188462	0.57876709
cg09720701	HOXC4;H	12	0.00412385	0.47574942	0.43143852	0.30914962
cg11585893	HOXC4;H	12	0.00910614	0.58366543	0.57931585	0.3878049
cg07266404	HOXC4;H	12	0.01120433	0.57943784	0.52580484	0.42412491
cg22747076	HOXC4;H	12	0.00742728	0.70600119	0.67681675	0.54906514
cg11746813	HOXC4;H	12	0.03333586	0.60193978	0.57285071	0.43506329
cg11597131	HOXD9	2	0.01263862	0.43929064	0.47056161	0.31342937

cg19373813	HOXD9	2	0.00260525	0.3656316	0.38315227	0.20591944
cg11938718	HPCAL1;H	2	0.0331983	0.66617777	0.6390316	0.52679427
cg24715735	HPN;HPN	19	0.02944061	0.43141077	0.46772566	0.3217902
cg06151964	HPN;HPN	19	0.01838071	0.60814432	0.63401773	0.43674194
cg12183501	HPS3	3	0.02108987	0.78674289	0.83477986	0.67551081
cg16745930	HPSE2;HP	10	0.00203531	0.70433238	0.68201451	0.51941795
cg09837116	HRNBP3	17	0.00083329	0.54904883	0.54089575	0.42647238
cg01944110	HS3ST1	4	0.03301459	0.80237687	0.85534593	0.70038496
cg18192491	HS3ST5	6	0.0106258	0.71647119	0.71353561	0.59913733
cg16684691	HS6ST3	13	0.00160642	0.49475247	0.50705064	0.32040855
cg09894383	HSD17B2	16	0.02215534	0.47985754	0.5100195	0.36964667
cg24888257	HSPA1A;H	6	0.00248169	0.62854237	0.58593913	0.448353
cg15888097	HTR1E	6	0.00576163	0.39982013	0.41874686	0.2772072
cg08273233	HTR1E	6	0.00101815	0.70033201	0.66466638	0.55231862
cg08372315	HTR3A;HT	11	0.01933517	0.56559578	0.62244995	0.44700932
cg01459748	HTR3B	11	0.00667841	0.62295498	0.66618646	0.4995048
cg23791011	HTRA1	10	0.0022585	0.77979399	0.70918993	0.43192054
cg00701951	HTRA1	10	0.00177922	0.86635382	0.84116418	0.64557767
cg11157691	HTRA1	10	0.00168532	0.8288142	0.79023025	0.56404768
cg15719652	HTRA1	10	0.00097337	0.89407485	0.82468978	0.67632805
cg21184369	HTRA4;PL	8	0.00123883	0.67705583	0.75866831	0.43910348
cg21121843	HTT	4	0.00264208	0.83418437	0.79619687	0.62196748
cg12793803	ICAM2;IC	17	0.00488333	0.55410733	0.59128773	0.44180412
cg15391590	ICK;ICK	6	0.02527866	0.83439259	0.88039234	0.72796466
cg21440776	IFFO1;IFF	12	0.00520168	0.73726246	0.6669884	0.510804
cg01342196	IFI16	1	0.00199536	0.74870552	0.64492543	0.50290375
cg07463059	IFI16;IFI1	1	0.0022221	0.65360528	0.65222048	0.36087997
cg20597486	IFI16;IFI1	1	0.01196286	0.76497243	0.79384697	0.55202393
cg16341979	IFI27;IFI2	14	0.00375204	0.75307552	0.72157622	0.59177783
cg24979161	IFI27L1;IF	14	0.00112793	0.83461416	0.78492116	0.64543198
cg17980508	IFI44L	1	0.00660543	0.48289854	0.45287156	0.25907801
cg01911529	IFI6;IFI6;I	1	0.00468024	0.91296061	0.88931269	0.78249448
cg27478224	IFIT2	10	0.01089189	0.24197998	0.2364414	0.12670379
cg27027427	IFIT3;IFIT	10	0.01102429	0.31531139	0.25837206	0.12533804
cg08173915	IFNGR2	21	0.04136151	0.759595	0.79887221	0.63919286
cg19724567	IFRD1	7	0.00446647	0.60152349	0.58683145	0.41315895
cg05526364	IFT122;IF	3	0.00070134	0.73754279	0.6661746	0.47656582
cg03163545	IFT140;TN	16	0.00291147	0.70065498	0.62667674	0.40288023
cg08761208	IGDCC4	15	0.00362881	0.60311896	0.64385165	0.44681503
cg26579713	IGDCC4	15	0.00392454	0.73612366	0.72586883	0.39480082
cg25924694	IGF1;IGF1	12	0.02818766	0.38405863	0.34376534	0.23768378
cg26577252	IGF1R	15	0.00796332	0.57776682	0.62602014	0.36391827
cg27139419	IGF1R	15	0.01321271	0.84630795	0.85024629	0.74090139
cg18158670	IGF1R	15	0.00327239	0.86651897	0.80744722	0.68901609
cg19131227	IGF2AS;IN	11	0.01010631	0.76664382	0.72095873	0.59909721
cg05323345	IGF2AS;IN	11	0.01678444	0.73456382	0.7588295	0.57266768

cg05777976	IGF2AS;IN	11	0.00160583	0.52841952	0.5880099	0.28060359
cg02425416	IGF2AS;IN	11	0.0097133	0.70371454	0.71328047	0.46829933
cg12877935	IGF2AS;IN	11	0.00966232	0.42510528	0.44935406	0.28160059
cg11005826	IGF2AS;IN	11	0.01073914	0.55863723	0.61688269	0.41233051
cg07060794	IGF2BP3	7	0.02432105	0.81988382	0.82204632	0.64794412
cg17209188	IGF2BP3	7	0.01045915	0.81465867	0.8012984	0.66442403
cg21025494	IGF2BP3	7	0.00486472	0.66812688	0.68974407	0.4909545
cg25754852	IGF2R	6	0.00093055	0.74974129	0.6860781	0.5831953
cg17980404	IGFBP4	17	0.00160451	0.74234146	0.62013235	0.40287844
cg06559575	IGFBP6	12	0.00068904	0.42963972	0.33191804	0.16343395
cg10923036	IGFBP7	4	0.00073265	0.49818055	0.43398496	0.27149433
cg15696506	IGFBP7	4	0.00526523	0.26994584	0.29100885	0.16143764
cg13840174	IGSF21	1	0.00247271	0.66326108	0.6636477	0.50958842
cg05468843	IL10RA;IL1	11	0.01093065	0.50106625	0.50057972	0.37817245
cg09577367	IL12RB1;I	19	0.0212437	0.51227392	0.52426352	0.3826732
cg01574571	IL12RB1;I	19	0.01857513	0.59384552	0.62257987	0.46814935
cg12123019	IL12RB1;I	19	0.00735604	0.60428359	0.6428988	0.5008307
cg16324121	IL17RE;IL1	3	0.03170823	0.67539013	0.69040085	0.51304565
cg00090674	IL17REL	22	0.00521564	0.78973076	0.78498253	0.68376532
cg01522296	IL17REL	22	0.00816896	0.7775404	0.79315608	0.6676618
cg12380764	IL19	1	0.01678972	0.56870211	0.58117806	0.42468987
cg00839584	IL1A;IL1A	2	0.03875896	0.55372142	0.57520626	0.44968683
cg02229946	IL1F7;IL1F	2	0.00828598	0.66574606	0.67391814	0.54523019
cg25065535	IL2	4	0.04742879	0.46472291	0.49449196	0.3492085
cg02656594	IL21R	16	0.03110358	0.57411412	0.57922667	0.44204848
cg05814654	IL21R;IL21	16	0.03699135	0.43024951	0.46734056	0.31881219
cg23507945	IL22RA2;I	6	0.01094414	0.82083662	0.82493485	0.65907444
cg22788465	IL3	5	0.00276855	0.51815882	0.51884424	0.35037231
cg05265849	IL6	7	0.02923471	0.36163016	0.36146418	0.25427524
cg07363330	IL31RA	5	0.00779924	0.60928126	0.57982479	0.38911772
cg04245540	IL31RA	5	0.00283173	0.29147213	0.24094326	0.12672558
cg16649560	IL4R;IL4R	16	0.00078819	0.52884707	0.44731078	0.24702329
cg04312209	IL7R	5	0.00507632	0.4835313	0.41358329	0.30527718
cg24833674	ILDR2	1	0.00662513	0.80697818	0.85675802	0.69833869
cg08065733	INHBB	2	0.02103823	0.40822907	0.42197274	0.29987411
cg06760238	INPP5A	10	0.0212437	0.63798543	0.65165146	0.43478539
cg03438101	INPP5A	10	0.03136149	0.61898507	0.63934991	0.49474098
cg07185664	INPP5K;IN	17	0.00901377	0.71493214	0.74267857	0.55499517
cg04057455	INS-IGF2;I	11	0.00295889	0.75298544	0.72559207	0.6175132
cg02742555	INSC;INSC	11	0.00206218	0.50823292	0.53406353	0.29245586
cg08129092	INTS3	1	0.0051141	0.38757918	0.40226025	0.26003963
cg16932472	IPO5;IPO5	13	0.00202182	0.55707247	0.54146656	0.40743254
cg22677858	IPO9	1	0.00101054	0.37047819	0.35593543	0.18868518
cg26915799	IPO9	1	0.02075286	0.80217804	0.79426286	0.66825516
cg18527651	IRAK2	3	0.04357378	0.71650606	0.73914304	0.59854023
cg16478536	IRF4	6	0.00806542	0.32958627	0.35617177	0.21974569

cg07955474	IRF8	16	0.00186737	0.68138434	0.68070318	0.55520548
cg04599946	IRF8	16	0.01447617	0.35570023	0.34238613	0.23696992
cg25312054	IRS2	13	0.00164959	0.86465668	0.76724318	0.64732668
cg01901262	IRX5	16	0.00163822	0.30004225	0.31143214	0.1970704
cg10824810	IRX5	16	0.00218291	0.75367507	0.70712562	0.49930716
cg02198701	IRX6	16	0.01164124	0.53711213	0.55864864	0.42123924
cg26293019	ISL1	5	0.00184545	0.53918132	0.51646783	0.31698869
cg24488317	ISM1	20	0.00066204	0.72046531	0.62021046	0.48800082
cg19185637	ITGA11	15	0.00113844	0.79685151	0.68480329	0.57168203
cg09828265	ITGA9	3	0.01021212	0.45549812	0.43509346	0.29596454
cg08821998	ITGA9	3	0.00380612	0.72959609	0.61260387	0.48900566
cg02225720	ITGAE	17	0.00129639	0.57084273	0.48840171	0.23159898
cg22699620	ITGB2;ITG	21	0.00131453	0.35787468	0.29930823	0.18953859
cg10753610	ITGB3	17	0.00798815	0.46914373	0.50455465	0.25277953
cg00871980	ITGB5	3	0.00341932	0.53089654	0.47998877	0.31421686
cg00171092	ITGB5	3	0.00921039	0.58105966	0.61786862	0.44166313
cg26689077	ITGB7	12	0.00200529	0.41579249	0.34062001	0.22807463
cg11838152	ITGBL1	13	0.00225672	0.80917572	0.81202292	0.5707773
cg24628310	ITIH1;ITIH	3	0.01096214	0.55430116	0.55776029	0.42898141
cg05480883	ITIH5;ITIH	10	0.00105291	0.29059657	0.22751066	0.11738832
cg08972954	ITK	5	0.04671332	0.52335519	0.57569895	0.38576024
cg10094191	ITLN1	1	0.00487618	0.67556016	0.68942443	0.56552978
cg14181956	ITPK1;ITP	14	0.04646611	0.73553542	0.76787401	0.63246349
cg22999830	ITPK1;ITP	14	0.03119297	0.82110082	0.84718371	0.70582428
cg11600734	ITPR1;ITP	3	0.02053379	0.74164143	0.75867648	0.55931005
cg02799411	ITPR1;ITP	3	0.03359449	0.76540617	0.77822659	0.64593993
cg05795849	ITPR1;ITP	3	0.0097133	0.37112661	0.36376901	0.19619129
cg25552843	ITPRIP	10	0.00591291	0.73388612	0.75238137	0.59012853
cg19640166	ITPRIP	10	0.0037901	0.81424425	0.81730617	0.65978532
cg16636756	ITPRIP	10	0.01946986	0.71904547	0.75196893	0.58295664
cg22888181	ITPRIP	10	0.00122881	0.72580898	0.66255889	0.39214589
cg24199006	ITPRIP	10	0.00195	0.61259203	0.52329474	0.33927433
cg05335315	JAG2;JAG	14	0.00281199	0.38107218	0.41403106	0.22702205
cg13250024	JAKMIP3	10	0.02474533	0.86615359	0.8690834	0.7542007
cg13623749	JARID2	6	0.02271157	0.69529006	0.6835812	0.51783824
cg26797722	JARID2	6	0.00066232	0.80016356	0.74840859	0.54623165
cg12230983	JARID2	6	0.02009829	0.79743114	0.84112914	0.69581673
cg01675238	JMJD7-PL	15	0.00085205	0.48931006	0.35091917	0.2070137
cg12024811	KALRN;KA	3	0.00126466	0.80426377	0.73452043	0.59179513
cg10464130	KALRN;KA	3	0.00073118	0.60667726	0.50337328	0.38314277
cg25250013	KANK2;KA	19	0.00077468	0.56337696	0.44333784	0.29810179
cg20916427	KATNAL2	18	0.02368354	0.71351848	0.74055946	0.57152735
cg13925011	KCNA3	1	0.01645394	0.65745653	0.66691083	0.52580497
cg04063166	KCNA4	11	0.00252115	0.51381201	0.59422503	0.38692143
cg03553947	KCNA4	11	0.01064534	0.7345547	0.71604588	0.61214174
cg25508319	KCNJ5	11	0.02332665	0.72885799	0.74075968	0.50682135

cg16739441	KCNJ8	12	0.01775699	0.3109984	0.33708338	0.20073503
cg07699454	KCNK17;K	6	0.00109306	0.81721192	0.78726131	0.65913199
cg01627597	KCNK3	2	0.00092849	0.4923467	0.46067257	0.32522795
cg23107301	KCNMA1;	10	0.00629716	0.69630588	0.68256298	0.5570724
cg26615853	KCNMA1;	10	0.00103895	0.89784652	0.88923792	0.76416552
cg22646937	KCNMB1;	5	0.00184569	0.51927202	0.49223777	0.32279827
cg04534404	KCNMB2;	3	0.0347271	0.78147232	0.7662987	0.66113137
cg05343739	KCNN3;K(	1	0.0019957	0.43387239	0.33160577	0.2256952
cg10503610	KCNN3;K(	1	0.00951557	0.66715671	0.63472554	0.5146332
cg08895013	KCNQ1	11	0.02987215	0.646089	0.67558179	0.54598008
cg18729298	KCNQ1;K(	11	0.00806542	0.49332259	0.48727504	0.37901649
cg07824422	KCNQ1;K(	11	0.00325324	0.5156602	0.57033779	0.35046451
cg16083838	KCNQ1DN	11	0.01666517	0.74063119	0.75564457	0.52754383
cg22675922	KCNQ1DN	11	0.02700619	0.64020414	0.70182109	0.53579626
cg01923099	KCNQ1DN	11	0.00359037	0.54348705	0.54582535	0.33185467
cg07439128	KCNQ1DN	11	0.01124644	0.66780443	0.67321147	0.45796252
cg05656180	KCNQ1DN	11	0.00302287	0.38584438	0.3415671	0.24078292
cg15746253	KCNQ3	8	0.0048146	0.76677935	0.78989411	0.62476678
cg16856453	KCNS1	20	0.00381594	0.44169595	0.42666403	0.2765973
cg13730736	KCNT1	9	0.0019957	0.46651642	0.46774918	0.32030599
cg24193140	KCTD16	5	0.00101054	0.76609966	0.6855268	0.58551314
cg09185884	KCTD2	17	0.0025513	0.39534072	0.306848	0.18029433
cg09175485	KDM2A;K	11	0.0047315	0.56067896	0.49342953	0.3115756
cg17416338	KDM4B	19	0.03699135	0.62471307	0.67772175	0.44528515
cg14498745	KEL	7	0.0135118	0.7301407	0.70699345	0.59749623
cg21031917	KHDRBS2	6	0.00236052	0.51146949	0.4641626	0.33567427
cg24471894	KIAA0020	9	0.00211366	0.43574232	0.38916641	0.20334028
cg16051718	KIAA0232	4	0.03599253	0.6601945	0.72335125	0.54410536
cg01557792	KIAA0247	14	0.01966195	0.65294351	0.67480514	0.52384381
cg26311501	KIAA0831	14	0.02683244	0.39120544	0.35560122	0.25129799
cg16328023	KIAA0895	7	0.00197515	0.41412966	0.39098755	0.22043911
cg02927252	KIAA1026	1	0.01216006	0.77888596	0.81599392	0.67434585
cg00101629	KIAA1026	1	0.00578816	0.74687817	0.66335771	0.53963368
cg16689761	KIAA1147	7	0.02488364	0.78150901	0.78187349	0.64484012
cg21282282	KIAA1324	1	0.00997311	0.48848141	0.53265453	0.29926269
cg01827195	KIAA1486	2	0.00667685	0.34367693	0.35897195	0.18703057
cg22417613	KIAA1549	7	0.02377716	0.79541728	0.80523365	0.65872424
cg08908131	KIAA1598	10	0.00141699	0.65371589	0.54788353	0.41938125
cg11165619	KIAA1644	22	0.00070678	0.80005055	0.71743758	0.59193083
cg02687592	KIAA1644	22	0.0033048	0.82705139	0.79725121	0.69178774
cg10759286	KIAA1751	1	0.02729523	0.44004771	0.46111765	0.33715044
cg08420066	KIAA1949	6	0.00861733	0.40533307	0.38354927	0.26655626
cg20690667	KIAA1949	6	0.0088759	0.40761779	0.39574501	0.24599962
cg24967096	KIAA1949	6	0.00336153	0.28339932	0.27401097	0.16218379
cg05098566	KIAA1949	6	0.00899594	0.65493394	0.61665975	0.47583357
cg15768443	KIAA1949	6	0.01068625	0.51234607	0.50385297	0.3772673

cg08887400	KIAA1949	6	0.00783809	0.3884505	0.35596619	0.22960721
cg03953626	KIAA1949	6	0.01291157	0.4589786	0.44657561	0.30749936
cg18586886	KIAA1949	6	0.01026108	0.50681496	0.4813915	0.31969025
cg18427856	KIAA1949	6	0.00408071	0.54034742	0.46714611	0.2827865
cg01413582	KIAA1949	6	0.00213077	0.32025887	0.27250905	0.16705629
cg00278359	KIAA1949	6	0.00171661	0.45871275	0.35378832	0.22321493
cg12036303	KIAA1949	6	0.00140274	0.68246232	0.60828261	0.37506826
cg00010853	KIAA1949	6	0.00178025	0.62168735	0.62765171	0.4082539
cg11554650	KIAA1949	6	0.00130462	0.7391302	0.66333643	0.52296211
cg12105190	KIAA1949	6	0.00100376	0.64443593	0.57302349	0.3627053
cg25980484	KIAA1949	6	0.00463727	0.36126457	0.33316476	0.23202179
cg03052182	KIAA1949	6	0.00302211	0.38195854	0.3479097	0.23298688
cg01662942	KIF1A	2	0.00523353	0.81219626	0.74221449	0.61592784
cg11802797	KIF1B	1	0.00429569	0.5720761	0.58603416	0.35264813
cg21477075	KIF25;KIF:	6	0.0021933	0.86271515	0.86398449	0.73664803
cg18234115	KIF26B	1	0.02630407	0.73659934	0.75559529	0.62086815
cg15358723	KIF26B	1	0.00343258	0.79790268	0.80970549	0.67512441
cg01078772	KIF3B	20	0.0008887	0.77617634	0.69458952	0.58641371
cg17226602	KIF4B	5	0.00564684	0.3606003	0.40039882	0.24610173
cg16861598	KIR2DL3;I	19	0.00631167	0.49158642	0.5183163	0.36639523
cg01819568	KIT;KIT	4	0.00144775	0.75825298	0.74738377	0.61950873
cg01308409	KL	13	0.01237771	0.66116328	0.73870588	0.55114969
cg06724409	KLF6;KLF6	10	0.00065949	0.8620331	0.77282008	0.64322774
cg21777553	KLF7	2	0.00703053	0.28914501	0.35410969	0.18774345
cg09606470	KLHL6	3	0.01665552	0.5177352	0.58457993	0.35512506
cg20659064	KLK10	19	0.01237894	0.37007161	0.36895516	0.23452832
cg08375658	KLK12;KLI	19	0.00953692	0.31985649	0.32820012	0.21900794
cg07798605	KLK9	19	0.01073914	0.57860584	0.55911594	0.44914644
cg15368872	KLRK1	12	0.03038061	0.46219349	0.49693453	0.35494947
cg11150901	KMO	1	0.00388546	0.40210383	0.51843317	0.24412988
cg27277403	KMO	1	0.00204611	0.27297835	0.29307855	0.1393513
cg04880751	KMO	1	0.0039229	0.68627691	0.65947342	0.42723885
cg20378687	KPNA4	3	0.02892693	0.54742742	0.62146372	0.4424896
cg03348792	KRT1	12	0.01481204	0.72456823	0.73109727	0.62258697
cg11471401	KRT6A	12	0.00638555	0.61370897	0.57671433	0.43469284
cg11051139	KRT80;KR	12	0.00173053	0.82089547	0.76890517	0.56201164
cg07925587	KRT80;KR	12	0.00183657	0.69124673	0.60723747	0.3590119
cg23878404	KRTAP1-5	17	0.00758031	0.59262762	0.58132709	0.44179463
cg14588606	KRTAP1-5	17	0.0012838	0.84284629	0.80838878	0.67135879
cg17903229	KRTAP10-	21	0.00117894	0.62058976	0.58633444	0.48358835
cg00275828	KRTAP2-4	17	0.00294138	0.77861591	0.77125345	0.6398423
cg23058745	KRTAP4-5	17	0.00252124	0.7778533	0.78817143	0.66661039
cg23718736	L3MBTL4	18	0.02530038	0.60126741	0.59860491	0.465121
cg22545168	LAIR1;LAI	19	0.02027046	0.54717511	0.52431117	0.42407585
cg21878746	LAIR1;LAI	19	0.02145574	0.53169691	0.58290014	0.42243193
cg13031029	LAMB1	7	0.04680546	0.63201759	0.75979881	0.45309023

cg15600176	LAMB1	7	0.00124243	0.86141936	0.82568032	0.72013196
cg03041510	LAMB1	7	0.00220808	0.66033598	0.66418317	0.4643557
cg07168232	LAMB3;LA	1	0.0008696	0.59371349	0.45277016	0.25204515
cg22809683	LAMC1	1	0.00200529	0.85387152	0.79157173	0.68247929
cg12689670	LAMC1	1	0.00351539	0.77703253	0.78567762	0.51616104
cg13766560	LAMC3	9	0.01580869	0.74400879	0.74793851	0.62630273
cg18030943	LAMP3	3	0.00081498	0.64660066	0.59852479	0.46298634
cg17418085	LAPTM5	1	0.01183547	0.73507928	0.71044627	0.52878216
cg04760493	LARP1B;L	4	0.01633983	0.45492475	0.50146271	0.3205396
cg03134947	LAT;LAT;L	16	0.03209984	0.49718339	0.50455581	0.38887468
cg18793512	LBH	2	0.00134068	0.84597508	0.82100047	0.63152095
cg10991454	LCLAT1;LC	2	0.00170786	0.79876008	0.7510454	0.5430074
cg23469878	LCN6	9	0.02087024	0.65373907	0.73672586	0.54582148
cg23205648	LCP1	13	0.00567752	0.4882437	0.56313324	0.38167867
cg25827710	LCP1	13	0.001223	0.26336038	0.28666139	0.12634204
cg08670465	LCP2	5	0.0048797	0.40735487	0.54194252	0.26001901
cg09451413	LCP2;LCP2	5	0.02802634	0.62662668	0.60427158	0.49958093
cg09672233	LCP2;LCP2	5	0.02115736	0.64807808	0.66617675	0.51969196
cg08262002	LDB2;LDB	4	0.00697437	0.54095721	0.55920373	0.42193868
cg27450010	LDB2;LDB	4	0.00447146	0.8234389	0.80569279	0.69162509
cg11821245	LDHC;LDH	11	0.01807343	0.42558703	0.49910672	0.27474025
cg06180729	LDLRAD3	11	0.00065949	0.90288059	0.86066427	0.74344289
cg18140889	LELP1	1	0.00068904	0.46179538	0.50951807	0.32507226
cg15466952	LEPR;LEPI	1	0.00151239	0.59649268	0.54313884	0.34572886
cg13439241	LGALS12;	11	0.00395839	0.32062618	0.34136591	0.14265439
cg11183156	LGALS12;	11	0.00279455	0.33421951	0.32103035	0.16710636
cg27376024	LGALS12;	11	0.00168532	0.2405602	0.24943433	0.11421902
cg26651950	LGALS2	22	0.0212437	0.60102979	0.53583239	0.40271602
cg04927537	LGALS3BI	17	0.00431388	0.69429775	0.69747531	0.57945598
cg14870271	LGALS3BI	17	0.00481061	0.64712361	0.64007889	0.49421641
cg24387542	LGALS9B	17	0.02089413	0.4067516	0.43203461	0.2930652
cg13597417	LGALS9C	17	0.01530568	0.41306754	0.44326639	0.28180735
cg02300379	LGI1	10	0.00262173	0.41884634	0.45408173	0.28286322
cg04468092	LGI2	4	0.00639169	0.87655314	0.88437048	0.76473211
cg11683364	LGR6;LGR	1	0.03497365	0.60844981	0.60104015	0.44308153
cg26658897	LHFPL2	5	0.00390072	0.78309096	0.74337524	0.60752432
cg16326367	LHFPL2	5	0.00072814	0.78985227	0.67428979	0.5068485
cg14305801	LHX2	9	0.04650849	0.64701738	0.67087966	0.47672518
cg06626184	LHX2	9	0.00425638	0.62278558	0.69799154	0.43740793
cg16353006	LHX9	1	0.00702537	0.44528096	0.41317134	0.25598366
cg05128414	LHX9	1	0.00279494	0.32289679	0.31576176	0.20969983
cg08463788	LHX9;LHX	1	0.01040141	0.60898948	0.56268731	0.45467255
cg08610862	LHX9;LHX	1	0.02313299	0.61684916	0.58636781	0.4834213
cg05920909	LHX9;LHX	1	0.00989476	0.61472751	0.58418085	0.43231563
cg25384595	LILRA1;LIL	19	0.04825245	0.60497639	0.65746272	0.48581301
cg19216475	LILRA2;LIL	19	0.00613201	0.32345945	0.35725179	0.19105866

cg26649140	LILRB1	19	0.00077468	0.27153198	0.31825481	0.16233788
cg05365532	LILRB1	19	0.00082157	0.57585145	0.54028727	0.39718416
cg03372852	LILRP2	19	0.00947108	0.40289655	0.45055995	0.29400341
cg25437886	LIMD1	3	0.01390012	0.5991029	0.59178002	0.35742054
cg05310309	LIMS1	2	0.00857669	0.77097547	0.74458665	0.59001428
cg03555157	LIMS2;LIM	2	0.00516729	0.43239102	0.41004292	0.30495319
cg04947052	LIMS2;LIM	2	0.00168532	0.84615307	0.80615123	0.7011208
cg01761729	LIMS2;LIM	2	0.00065949	0.52468624	0.42220161	0.28398631
cg17728974	LIN28B	6	0.00655853	0.40692074	0.4389373	0.24730012
cg19721087	LIN28B	6	0.04280685	0.59470952	0.54588604	0.44437271
cg08382737	LIN7B	19	0.03824432	0.342687	0.32842583	0.22612202
cg09797463	LIPA;LIPA	10	0.01296171	0.78917078	0.7604425	0.63150776
cg12581682	LIPC	15	0.00065949	0.88650569	0.82239514	0.71425823
cg08767044	LITAF;LITA	16	0.00806542	0.75532725	0.76393542	0.54794108
cg26083045	LMCD1	3	0.02658897	0.54848449	0.52039	0.40217138
cg05989795	LMF1	16	0.04596111	0.63826345	0.62204506	0.50244775
cg17453456	LMNA;LM	1	0.00131641	0.23737618	0.22096722	0.11962453
cg27182012	LMNA;LM	1	0.00280066	0.85221762	0.85155892	0.7176782
cg08586426	LMO2	11	0.00213077	0.75594378	0.73506672	0.493089
cg04757411	LMO7	13	0.00123845	0.85158294	0.83181277	0.64465916
cg26400954	LMO7	13	0.00101151	0.71499636	0.62825743	0.47503879
cg18833788	LMTK3	19	0.01457962	0.5116214	0.51422978	0.40301876
cg14325930	LMX1B	9	0.01428062	0.67255826	0.73720435	0.52239461
cg18560915	LNX1	4	0.00146177	0.77338084	0.71894534	0.5584645
cg23543318	LOC10013	4	0.0101333	0.74944754	0.81699768	0.61671923
cg23127323	LOC10013	4	0.01083395	0.40459458	0.4317092	0.29041907
cg18120259	LOC10013	6	0.00736725	0.83961729	0.85621993	0.68568865
cg20874785	LOC10018	2	0.00958999	0.57774729	0.6187259	0.43139707
cg00230120	LOC10029	12	0.00129639	0.88313403	0.8532236	0.72422982
cg24951335	LOC10029	12	0.0261269	0.65107459	0.61748077	0.47748051
cg18071202	LOC10029	12	0.02665468	0.69939025	0.69288723	0.54518383
cg14475966	LOC14584	15	0.003299	0.77057612	0.76213611	0.60968902
cg00213301	LOC14584	15	0.0103127	0.64529228	0.62465269	0.4850171
cg14891209	LOC14584	15	0.00201346	0.27087938	0.28079441	0.1463277
cg12309653	LOC14584	15	0.00317466	0.48476276	0.4844662	0.29805612
cg00216138	LOC14584	15	0.00176484	0.54392107	0.56346692	0.26722653
cg06451900	LOC14584	15	0.00111281	0.75599327	0.6990499	0.47049889
cg24956533	LOC14584	15	0.00283173	0.84114693	0.79906348	0.61416629
cg22444584	LOC14584	15	0.00334886	0.6620054	0.62373208	0.43492644
cg11965311	LOC14584	15	0.00237687	0.86856851	0.80545802	0.62601812
cg05429448	LOC15222	3	0.00152273	0.8206719	0.80783922	0.51971705
cg12963656	LOC15222	3	0.00472204	0.80303031	0.79967425	0.54737384
cg15046507	LOC22112	11	0.0038151	0.23511329	0.23578829	0.1324463
cg22093306	LOC25455	15	0.01240534	0.42219966	0.46810191	0.27626114
cg15129183	LOC25735	5	0.0048909	0.7743971	0.78296554	0.52060559
cg09802818	LOC28340	12	0.00913085	0.6193411	0.64125333	0.46331686

cg00220661	LOC2842[illegible]	18	0.01015119	0.31644203	0.31993609	0.20051023
cg01723706	LOC2854[illegible]	4	0.00548311	0.53238555	0.56619518	0.40935759
cg26701510	LOC2858[illegible]	6	0.02524768	0.57373755	0.6591501	0.3854722
cg12035144	LOC2858[illegible]	6	0.00675316	0.37327059	0.39784835	0.20570111
cg01751584	LOC2863[illegible]	9	0.00206218	0.82594085	0.7815495	0.64072637
cg21242356	LOC4042[illegible]	17	0.01817172	0.37953377	0.39910791	0.27949114
cg17062109	LOC4042[illegible]	17	0.00833663	0.39300422	0.35246064	0.24493007
cg22583148	LOC4042[illegible]	17	0.01622063	0.50032129	0.47998712	0.37021367
cg15908709	LOC4042[illegible]	17	0.0024386	0.46452065	0.42531897	0.27048325
cg17528189	LOC4400[illegible]	11	0.01317469	0.2894296	0.29172002	0.16696938
cg01821850	LOC4409[illegible]	2	0.00297171	0.65075864	0.65665719	0.51665434
cg05146756	LOC5542[illegible]	9	0.00964959	0.33868296	0.34493748	0.19259547
cg01878406	LOC6466[illegible]	1	0.00624726	0.32833251	0.33086767	0.21923116
cg23590187	LOC6467[illegible]	7	0.00840556	0.40727132	0.37488788	0.23625272
cg13346869	LOC7280[illegible]	8	0.03358008	0.40736589	0.40394409	0.28034231
cg20414364	LOC7286[illegible]	5	0.0122155	0.64404602	0.69065125	0.45069535
cg09048205	LOC7286[illegible]	5	0.01447617	0.76499283	0.76605627	0.59107179
cg02927821	LOC7307[illegible]	17	0.03384214	0.52339618	0.49909706	0.3949096
cg05825085	LOC7322[illegible]	16	0.036396	0.76987379	0.77653109	0.66318503
cg15111469	LOX	5	0.01710021	0.70696855	0.66475461	0.55877788
cg23345292	LOXHD1;L[illegible]	18	0.00174903	0.58021633	0.56918003	0.46596055
cg19877248	LOXL2	8	0.02025944	0.76639438	0.74015128	0.62605653
cg14429427	LPAR1;LP[illegible]	9	0.04388834	0.38595107	0.38185728	0.27759966
cg12646794	LPHN3	4	0.00723854	0.41981983	0.49512471	0.29704754
cg16225703	LPHN3	4	0.00506149	0.76026029	0.77835868	0.64110018
cg12297546	LPP;LPP	3	0.00081925	0.86338713	0.84256767	0.72740766
cg04773529	LPP;LPP;L	3	0.00405622	0.35388469	0.41315854	0.19710087
cg00902153	LPP;LPP;L	3	0.00186404	0.48188115	0.36895243	0.23459298
cg04103706	LPP;LPP;L	3	0.01574127	0.91625979	0.90900511	0.77071638
cg02011953	LPPR1	9	0.00614688	0.47680808	0.55628007	0.37222308
cg02044895	LPXN;LPX	11	0.01061523	0.33879515	0.27052786	0.16341601
cg20294319	LRCH1;LR	13	0.01502763	0.49350061	0.46727277	0.31938621
cg18005280	LRIG1	3	0.00503285	0.72638403	0.67911734	0.55881456
cg24112628	LRP11	6	0.01698967	0.83313214	0.84123476	0.72082091
cg03252605	LRP1B	2	0.0059385	0.39425609	0.41013412	0.27848876
cg16671674	LRP1B	2	0.00183351	0.65206237	0.6598797	0.52446719
cg15664504	LRP1B	2	0.03564926	0.65412788	0.67311811	0.48594697
cg03678566	LRP5	11	0.00112679	0.82840045	0.77433508	0.62908984
cg26838900	LRRC15;L[illegible]	3	0.00362629	0.80373417	0.79633378	0.69296402
cg09670263	LRRC2;LR[illegible]	3	0.00065949	0.88290375	0.7593238	0.61664449
cg05076755	LRRC32;L[illegible]	11	0.00457701	0.70694331	0.68539676	0.43577574
cg27022853	LRRC33	3	0.04063967	0.66203899	0.6658002	0.55659906
cg15819128	LRRFIP1;L	2	0.00763406	0.48496125	0.43000142	0.23445428
cg12283393	LSP1	11	0.01581441	0.73370475	0.75134526	0.62697632
cg09761080	LST1;LST1	6	0.01688348	0.53591913	0.5653522	0.43338542
cg16056219	LTBP2	14	0.00168532	0.50775679	0.35677128	0.24963129

cg26011692	LTBP2	14	0.0122204	0.91382412	0.91644961	0.76744236
cg14986500	LTBP2	14	0.00846831	0.70649055	0.71296075	0.57247538
cg14340889	LTBP2	14	0.00077454	0.63907877	0.5561509	0.43062689
cg03491087	LTBP2	14	0.0012838	0.69132115	0.6177145	0.44104513
cg18865207	LY9;LY9	1	0.00239883	0.58927868	0.61775087	0.42805594
cg10316357	LY9;LY9	1	0.0042005	0.2607422	0.26774439	0.14982943
cg13213009	LY96	8	0.01687692	0.63218903	0.61887418	0.4488194
cg23732024	LY96	8	0.00666111	0.87969117	0.86358376	0.73768627
cg08806632	LYL1	19	0.02445578	0.56595961	0.60284587	0.41571932
cg05095252	LYPD6	2	0.04358513	0.6642786	0.67570494	0.52890635
cg22367631	MACF1	1	0.00385922	0.56825384	0.55103462	0.31053864
cg26864766	MACF1;M	1	0.00506968	0.70391133	0.65957234	0.51189616
cg00502509	MAD1L1;I	7	0.00065949	0.9144899	0.86905492	0.74340661
cg21728101	MAD1L1;I	7	0.00705117	0.66894838	0.71752465	0.49584251
cg10030633	MAD1L1;I	7	0.03599253	0.85446029	0.77102122	0.65836785
cg27109748	MAD1L1;I	7	0.00065949	0.82342601	0.74966462	0.60784069
cg00575665	MAFB;MA	20	0.00349176	0.5255617	0.42047375	0.29484407
cg01403010	MAFF;MA	22	0.04610222	0.73476953	0.75210859	0.57996527
cg05593887	MAGI2	7	0.04832512	0.7123082	0.69045598	0.44626022
cg14649449	MAMDC2	9	0.00073265	0.67817247	0.57801778	0.37595974
cg04677410	MAML2	11	0.04870447	0.83064122	0.80150189	0.6919768
cg15521790	MAML2	11	0.00239822	0.47796427	0.37462924	0.2422067
cg01093786	MAML2	11	0.00392454	0.29205989	0.24708013	0.13330103
cg23206160	MAML3	4	0.00219764	0.39332712	0.36293514	0.18555697
cg03597159	MAML3	4	0.00075518	0.71249549	0.61612308	0.45880213
cg08880153	MAP1A	15	0.00092595	0.80300756	0.72917053	0.59949221
cg15076869	MAP2K2	19	0.014833	0.54462309	0.52956973	0.38948185
cg14573876	MAP2K2	19	0.00408071	0.65261586	0.60325819	0.36932551
cg00146864	MAP3K14	17	0.00768024	0.6084765	0.5811205	0.44773075
cg19982668	MAP3K3;I	17	0.00615082	0.76866605	0.79363329	0.61230987
cg07474842	MAP3K5	6	0.00387438	0.83219982	0.82405598	0.59620024
cg21778987	MAP3K5	6	0.00218837	0.61189556	0.62880821	0.3163445
cg09331409	MAP6;MA	11	0.00597821	0.29075379	0.25425229	0.14300659
cg26946769	MAPK4	18	0.02252087	0.62416148	0.63744721	0.50161566
cg14321936	MAPKAPK	3	0.00665442	0.7231191	0.66776873	0.5660381
cg16805068	MAPRE1	20	0.00127437	0.83847416	0.79859288	0.67539301
cg07017164	MAPT;MA	17	0.03110358	0.79241005	0.78522026	0.67394331
cg15498306	MARCH1;	4	0.00674191	0.60041005	0.59259089	0.40584326
cg14026106	MARCH8;	10	0.00932052	0.676441	0.63434949	0.5301955
cg11009736	MARCO	2	0.04889422	0.63125155	0.64560776	0.52401692
cg02224164	MARS	12	0.00128812	0.67358934	0.54919416	0.42804705
cg13305823	MAS1L	6	0.00535942	0.45880528	0.53398629	0.33640629
cg25818109	MASP1	3	0.00206218	0.58697094	0.53220655	0.3851442
cg25391820	MATR3;SI	5	0.03931081	0.39061763	0.40101125	0.28439984
cg08535978	MBNL1;M	3	0.01317683	0.42670113	0.42295811	0.29103709
cg24446429	MBP;MBI	18	0.00176816	0.79448176	0.74024068	0.53340654

cg16762684	MBP;MBI	18	0.00749864	0.38169145	0.35457993	0.21062895
cg27262850	MBP;MBI	18	0.03686125	0.72663094	0.72827544	0.60079499
cg24853975	MCC	5	0.00521564	0.65197445	0.62041792	0.47323548
cg06062378	MCC;MC(	5	0.00111524	0.77353528	0.72503245	0.46546844
cg08213909	MCC;MC(	5	0.00065949	0.84341221	0.74674764	0.57299861
cg11521563	MCF2L;M	13	0.01009622	0.64154842	0.61437102	0.50051307
cg12560772	MCHR2;M	6	0.00539114	0.56542882	0.61426915	0.45177371
cg06778183	MCTP1;M	5	0.00751061	0.68070109	0.7013914	0.46529012
cg01278975	MDGA1	6	0.00179217	0.8270529	0.88995951	0.49604896
cg19503462	MDGA2	14	0.01095648	0.34041436	0.33271263	0.1797042
cg25956089	MECOM;I	3	0.0355165	0.47882432	0.47880802	0.31739439
cg10371155	MED1	17	0.00405622	0.79732773	0.79736461	0.62238452
cg05689413	MED20	6	0.00925322	0.43983489	0.39770646	0.26329936
cg03301498	MED29	19	0.00140256	0.77986787	0.72777111	0.52163003
cg05554494	MEFV	16	0.01248948	0.73692586	0.7707355	0.60001806
cg03919650	MEFV	16	0.00145334	0.40069137	0.39532365	0.20546887
cg26846864	MEFV	16	0.00073118	0.67470952	0.60717293	0.44447225
cg09187007	MEGF6	1	0.00209505	0.33759556	0.35407959	0.22340482
cg09425926	MEST	7	0.02001663	0.68775984	0.68589399	0.56732676
cg24197567	MET;MET	7	0.00073265	0.57168016	0.52408161	0.35849286
cg11056055	MET;MET	7	0.00075494	0.7741613	0.71235172	0.59267914
cg23992886	MET;MET	7	0.01946986	0.81945418	0.809594	0.70093143
cg15224459	METRNL	17	0.00317348	0.61521241	0.61999438	0.35101289
cg02633149	METRNL	17	0.00154265	0.78801029	0.72461389	0.57325676
cg12666827	METRNL	17	0.02846049	0.76288924	0.72615767	0.58796521
cg09808606	METTL9;I	16	0.04470828	0.67853696	0.71438659	0.53945014
cg07917978	METTL9;I	16	0.0045798	0.40570831	0.40307296	0.26771955
cg12695612	MFAP2;M	1	0.00065949	0.88510259	0.79012787	0.61914402
cg17744878	MFAP2;M	1	0.00206867	0.71653349	0.65219488	0.46775342
cg19306948	MFAP2;M	1	0.00206218	0.72506015	0.67906802	0.55827706
cg10201923	MFAP2;M	1	0.00201167	0.93495918	0.92833974	0.82407831
cg08477744	MFAP2;M	1	0.00074801	0.89669452	0.82300604	0.71633111
cg07086226	MFSD7	4	0.00068904	0.63294558	0.43828614	0.30533557
cg01149192	MGAT1;M	5	0.03609869	0.70152255	0.69295598	0.55662569
cg04212021	MGAT1;M	5	0.01321271	0.78951096	0.80558321	0.62589701
cg26050975	MGAT4B;	5	0.01729033	0.6990971	0.63715187	0.47091451
cg05514299	MGAT5B;	17	0.00539114	0.44829312	0.47036989	0.26160066
cg21868063	MGAT5B;	17	0.00344865	0.53038133	0.55881222	0.37902238
cg06975633	MGC2738	1	0.00503375	0.56040577	0.53715382	0.41863874
cg03148889	MGC2738	1	0.00670817	0.45438154	0.46474012	0.31606617
cg10435609	MGC2738	1	0.00073547	0.85775074	0.77408014	0.62871202
cg24113496	MGC295(	5	0.01396719	0.50395915	0.50475929	0.35582482
cg23370946	MGC458(	4	0.019985	0.61517752	0.67838396	0.50687444
cg16027775	MGLL;MG	3	0.00077468	0.64587779	0.53376646	0.29024118
cg22575127	MGMT	10	0.00081925	0.89947349	0.84894437	0.72419445
cg03821121	MICAL2	11	0.00766486	0.72216153	0.65195628	0.46145422

cg03078520	MICAL3;M	22	0.02122541	0.72854755	0.75394144	0.60556538
cg00044354	MIER1;W	1	0.008246	0.3977376	0.37550232	0.26683243
cg24199384	MIPEP	13	0.00232326	0.72716043	0.6346251	0.51040214
cg26614229	MIR1182;	1	0.00380612	0.30149272	0.30059379	0.17532655
cg22492271	MIR1200;	7	0.00118039	0.78938901	0.74669409	0.58579891
cg05076730	MIR1207;	8	0.01997904	0.8100912	0.83496492	0.70125299
cg00444740	MIR1208	8	0.01530568	0.8402745	0.86275628	0.72798779
cg07018107	MIR1208	8	0.00174783	0.87227184	0.82636605	0.64540606
cg27292264	MIR1280;	3	0.00141699	0.34052974	0.34810755	0.23345794
cg00477302	MIR182	7	0.00532167	0.36152493	0.35755778	0.22589082
cg23955417	MIR1976;	1	0.00936413	0.45064434	0.4852691	0.33961533
cg02515217	MIR21	17	0.0012504	0.78925828	0.69991917	0.54570447
cg15759721	MIR21	17	0.00595714	0.70963496	0.67801889	0.4678874
cg03184776	MIR2117	17	0.00104452	0.49040521	0.43297842	0.26761067
cg25687874	MIR2117	17	0.00065949	0.58777195	0.51698579	0.36332966
cg16506910	MIR30A	6	0.00186249	0.73673274	0.73804003	0.60040986
cg10583119	MIR518D	19	0.00317234	0.35285926	0.33890038	0.23195486
cg17670263	MIR520C	19	0.00371278	0.36695103	0.35502356	0.23561222
cg26664528	MIR548H	15	0.00073118	0.81537129	0.7008088	0.54610629
cg20587874	MIR548N	2	0.00127427	0.53034081	0.49244019	0.27997929
cg01706263	MIR598;X	8	0.00159552	0.40289897	0.47034003	0.25696748
cg18496212	MITF	3	0.01874852	0.82219284	0.8450142	0.72102375
cg04811592	MITF;MIT	3	0.00081046	0.62542091	0.55321762	0.4445108
cg15571405	MKLN1	7	0.00095411	0.20829835	0.21085987	0.10028416
cg03677952	MLC1;ML	22	0.01546058	0.56430509	0.58229296	0.41174943
cg08660915	MLC1;ML	22	0.00716301	0.40970245	0.44907779	0.29450127
cg14271023	MLPH;ML	2	0.00276855	0.87876709	0.91001272	0.72605008
cg22729539	MLXIP	12	0.03811638	0.81726051	0.84514268	0.68458652
cg17865265	MMP19	12	0.00363129	0.67269231	0.69445368	0.50456171
cg03509949	MMP19	12	0.00997311	0.39278747	0.37195401	0.25454613
cg07436701	MMRN2;S	10	0.00315764	0.79136181	0.79837719	0.65507033
cg05304729	MNDA	1	0.00857669	0.57367505	0.5888867	0.46388639
cg21876918	MOBKL2A	19	0.02002988	0.72172398	0.74816772	0.57806694
cg24938761	MOBKL2A	19	0.00223182	0.75818393	0.69708576	0.50654441
cg13648550	MOBKL2E	9	0.01608519	0.68327779	0.70511695	0.55931215
cg14643264	MOBKL2E	9	0.01506509	0.73695036	0.70297976	0.55634382
cg11621113	MORG1;M	19	0.00430406	0.33746953	0.34791775	0.22689592
cg00032879	MORN1	1	0.00953692	0.75160624	0.76774998	0.59768422
cg23110109	MORN3	12	0.01645625	0.29978916	0.29241505	0.18630373
cg01422009	MPG	16	0.00126879	0.56919077	0.44150036	0.33412269
cg18936620	MPL	1	0.00163336	0.68309639	0.65409071	0.43846718
cg04988978	MPO	17	0.00359274	0.34918433	0.39202584	0.23702016
cg17327331	MPP5	14	0.00074016	0.60765291	0.48964625	0.34686604
cg14599440	MPPE1	18	0.01390004	0.53065588	0.53239265	0.37173355
cg08591668	MRE11A;I	11	0.01488914	0.52833133	0.5314166	0.36676408
cg13062935	MRGPRX2	11	0.02568798	0.57427782	0.64742143	0.4708839

cg26371345	MRPL14	6	0.00501826	0.43438718	0.49681059	0.32310021
cg01445659	MRPL34;/	19	0.03905874	0.44728012	0.44857694	0.34457347
cg23924737	MRPS23	17	0.00802646	0.693967	0.66692794	0.51790254
cg00673646	MS4A6A;I	11	0.01009622	0.48602193	0.53694864	0.31417672
cg16773768	MSRA;MS	8	0.00066204	0.78875608	0.68821202	0.5245629
cg00791074	MTHFD1L	6	0.00073378	0.71476896	0.59499009	0.32974505
cg20694147	MTHFD2;	2	0.00077013	0.35368454	0.27544413	0.13173265
cg05959989	MTL5;MT	11	0.02817065	0.65224972	0.6553287	0.53001267
cg10995381	MTRR;MT	5	0.00119228	0.85115412	0.82826693	0.70556689
cg05656374	MX2	21	0.00548311	0.61935841	0.68111131	0.43941431
cg25368647	MXD3;M›	5	0.00171661	0.86602411	0.8170258	0.61639455
cg14606858	MYH16	7	0.01233628	0.50206983	0.51414338	0.34058387
cg02558132	MYLK;MY	3	0.00628685	0.79519737	0.77749024	0.67351258
cg19611163	MYLK;MY	3	0.00168532	0.78744044	0.75446761	0.61742917
cg12235788	MYLK;MY	3	0.01915914	0.73428666	0.73567092	0.58274311
cg16402006	MYLK4	6	0.02336655	0.86052575	0.84985909	0.7338176
cg15453396	MYLK4	6	0.00134295	0.79857057	0.80807325	0.61466471
ch.5.432310I	MYO10	5	0.00806395	0.21701477	0.21403821	0.11295214
cg06742628	MYO10	5	0.00160732	0.62302742	0.57046052	0.45763479
cg20748397	MYO15A	17	0.00097487	0.83687495	0.79899724	0.69430932
cg02920604	MYO1E	15	0.00077183	0.3249656	0.25553475	0.14209273
cg24128316	MYOM1;I	18	0.01098413	0.3712795	0.43977423	0.26554769
cg20103825	MYOM2	8	0.00123203	0.29289438	0.28114015	0.14384489
cg10215032	MYT1L	2	0.00747631	0.3483833	0.34092682	0.23837056
cg09716613	N4BP2L1;	13	0.01231302	0.57879246	0.60442073	0.38545456
cg18700744	NAA25	12	0.00071044	0.72419605	0.57598804	0.43085123
cg25024321	NALCN	13	0.0190357	0.75307059	0.7758376	0.65214422
cg06934394	NALCN	13	0.00212575	0.5197309	0.39278482	0.28655484
cg11147919	NALCN	13	0.03346286	0.55870353	0.57565544	0.45652116
cg24324984	NANOS2	19	0.00753326	0.52855368	0.52085898	0.41540764
cg26609120	NAPSB	19	0.00197576	0.54015152	0.56583161	0.42438516
cg15474337	NAPSB	19	0.013012	0.72372655	0.73799655	0.592552
cg19323289	NAV2	11	0.00337598	0.74197632	0.74794587	0.60881631
cg02849401	NAV2	11	0.00126059	0.88664269	0.82414231	0.64964171
cg14534803	NAV2	11	0.000682	0.80298305	0.67109159	0.54941147
cg19141316	NAV2;NA'	11	0.00201017	0.9181361	0.89682212	0.77859866
cg02959112	NBL1;NBL	1	0.00099274	0.82131804	0.72449975	0.61189565
cg10024909	NBLA003(	4	0.00635299	0.57063924	0.66294291	0.43295744
cg15234400	NBLA003(	4	0.00840303	0.73513679	0.79325144	0.56167988
cg26805882	NBLA003(	4	0.00787909	0.8152664	0.85562163	0.69456473
cg21383280	NBLA003(	4	0.00679356	0.49156235	0.53781492	0.31227738
cg21727486	NCAM1;N	11	0.02411591	0.53594642	0.59843789	0.43065621
cg03147185	NCAPH	2	0.00146869	0.57911773	0.5636349	0.41851156
cg02532700	NCF4;NCI	22	0.02956308	0.63308433	0.68121195	0.52288113
cg14416623	NCKAP5;I	2	0.01373528	0.5364141	0.50659593	0.31623103
cg16328548	NCKAP5;I	2	0.02417619	0.70233341	0.71090671	0.59890378

cg04804139	NCOR2;N	12	0.00079224	0.89264059	0.74464026	0.60730096
cg23484380	NCR3;NCI	6	0.00429038	0.34949987	0.37111829	0.23603996
cg08610982	NCR3;NCI	6	0.00369078	0.44667595	0.44567127	0.24367091
cg13910377	NCRNA00	21	0.00944446	0.64625546	0.64176115	0.53807751
cg04436964	NDUFS2;A	1	0.00092849	0.65029329	0.58335052	0.46692639
cg08850169	NDUFS2;N	1	0.02658729	0.40222908	0.39087641	0.28810901
cg08580187	NECAB2	16	0.00667685	0.66716896	0.67772295	0.4134033
cg13822256	NEFL	8	0.00494816	0.57871718	0.59786294	0.41002813
cg13008174	NEIL2;NEI	8	0.00803433	0.51421563	0.50048425	0.33898183
cg17280514	NEU4;NEU	2	0.00093851	0.67271671	0.58953471	0.4636089
cg00351443	NEURL	10	0.03615697	0.72981485	0.73616517	0.61190606
cg18636003	NEURL	10	0.04194736	0.63404786	0.68278609	0.50325047
cg20806345	NEUROD1	2	0.00319532	0.29965828	0.36047324	0.1791862
cg05109049	NF1;EVI2I	17	0.00694471	0.77084202	0.74172284	0.57107976
cg10186605	NF1;NF1	17	0.00160273	0.76528114	0.69138046	0.59097422
cg08915824	NFASC;NF	1	0.00755255	0.62936011	0.65023681	0.46601087
cg21964564	NFASC;NF	1	0.00504019	0.35798276	0.33335202	0.19249642
cg27050612	NFE2L1	17	0.01880817	0.38462328	0.39389102	0.27766316
cg12510708	NFE2L3	7	0.00552473	0.65108253	0.64074395	0.44861888
cg16303048	NID2	14	0.00572805	0.46547231	0.36325428	0.22950269
cg11087503	NID2	14	0.00122358	0.78871014	0.72662559	0.60893573
cg24575128	NISCH	3	0.00539186	0.53659218	0.47656118	0.33095925
cg12300018	NKAIN3	8	0.00262173	0.65408663	0.65898414	0.54609251
cg06874016	NKIRAS2;I	17	0.00262173	0.27016983	0.29650156	0.15663052
cg11330740	NKX2-5;N	5	0.03251446	0.52010523	0.55432546	0.36330951
cg18017926	NKX2-5;N	5	0.01137235	0.39286715	0.40986608	0.28404988
cg07383092	NKX2-5;N	5	0.01038907	0.43533684	0.44563624	0.28670253
cg01040654	NKX2-5;N	5	0.03547817	0.65440181	0.66973136	0.51415717
cg06374325	NKX6-1	4	0.00520163	0.5508383	0.53496009	0.38333688
cg24845848	NKX6-1	4	0.01342353	0.30850714	0.32601431	0.18328887
cg18297736	NKX6-1	4	0.00174783	0.37547959	0.39529683	0.16133006
cg22256677	NKX6-1	4	0.00083214	0.30920692	0.39458941	0.15860159
cg25830182	NKX6-1	4	0.0010638	0.29221345	0.32114001	0.14879777
cg02489958	NKX6-1	4	0.00176816	0.42195326	0.45765637	0.19540419
cg27406678	NKX6-1	4	0.00496156	0.38263829	0.40937685	0.19002688
cg22598426	NKX6-1	4	0.00112617	0.33490635	0.39945035	0.18738166
cg15704699	NLK	17	0.00089487	0.303211	0.3286446	0.14623414
cg22171142	NLRC3	16	0.03249565	0.60579637	0.62956405	0.50272581
cg24307499	NLRP2	19	0.00999778	0.70497108	0.74214067	0.60388658
cg13101705	NMNAT2;	1	0.00197986	0.49280083	0.49414371	0.33888761
cg07429087	NMUR2	5	0.01528948	0.63471052	0.65832788	0.53226109
cg19071544	NOD1	7	0.03352901	0.75802731	0.77327463	0.63158175
cg01243823	NOD2	16	0.03358212	0.39210091	0.4011104	0.26317148
cg03250947	NOTCH4	6	0.00440148	0.67280381	0.63892295	0.50747326
cg22008551	NOVA2	19	0.01581441	0.59551388	0.57426636	0.47238978
cg05797854	NOX3	6	0.00513872	0.64376339	0.65237945	0.4464034

cg06979108	NOX4	11	0.01734283	0.59706997	0.58118842	0.46830866
cg21609584	NPAS2	2	0.00488665	0.76690443	0.69835951	0.59036712
cg06850660	NPFF	12	0.0019386	0.37865051	0.34561927	0.21075544
cg26647771	NPR3	5	0.00065949	0.68991509	0.56392416	0.41924889
cg01335126	NPW	16	0.00456803	0.46282084	0.45703138	0.3310791
cg09636661	NQO1;NC	16	0.00563553	0.32516558	0.29485485	0.17581161
cg09244872	NR1H4	12	0.00200023	0.55943775	0.56614255	0.45446194
cg08969950	NR2F1	5	0.01887078	0.59916595	0.61646231	0.40887407
cg26593946	NR2F2	15	0.0048909	0.28724871	0.32155607	0.14409494
cg05108467	NR2F2	15	0.00207334	0.20344191	0.21233432	0.09093829
cg01213447	NR2F2	15	0.0017637	0.55841012	0.62938784	0.39051217
cg20754348	NR2F2;NF	15	0.00227742	0.34478809	0.32305769	0.18367398
cg27236629	NR2F2;NF	15	0.00281906	0.50074322	0.64562774	0.37861765
cg23964820	NRG1	8	0.01207774	0.3639441	0.27359417	0.16414843
cg27133711	NRG1	8	0.00967401	0.50504757	0.55162414	0.29127073
cg17519477	NRG3;NR	10	0.00255413	0.7416265	0.67994366	0.54665467
cg06850159	NRG3;NR	10	0.00670817	0.42917704	0.49758898	0.2892594
cg27270412	NRP1;NRI	10	0.0063644	0.86879775	0.83098701	0.68586277
cg19731541	NRP2;NRI	2	0.00115308	0.69039616	0.65979851	0.46175196
cg12884814	NRXN1;NI	2	0.01653772	0.609993	0.6113118	0.50520812
cg10917619	NRXN1;NI	2	0.01580869	0.68647891	0.69218938	0.57900042
cg08623810	NRXN2;NI	11	0.00077454	0.78902664	0.63817743	0.39415374
cg14185201	NRXN3	14	0.00177039	0.33412724	0.33605357	0.20656704
cg10991579	NRXN3	14	0.01479951	0.32833463	0.32570616	0.20415735
cg10037913	NSMCE1	16	0.01788219	0.60218966	0.64030695	0.48218404
cg16396933	NT5C2;NT	10	0.03645999	0.56508409	0.55538816	0.45045936
cg06004033	NT5DC3	12	0.0021194	0.39865922	0.41842131	0.28686247
cg00925339	NT5E	6	0.00543186	0.52451088	0.48106222	0.3661907
cg12445309	NTM	11	0.0007901	0.54512983	0.4885718	0.33677004
cg26567736	NTM	11	0.00160662	0.44339304	0.44009892	0.31646284
cg16763885	NTM;NTM	11	0.00299824	0.25358476	0.26358519	0.12967581
cg15370309	NTN1	17	0.00124266	0.65336292	0.52683281	0.38862605
cg25699759	NTRK3;NT	15	0.00074801	0.83327634	0.79379211	0.68270995
cg03287527	NUBP1	16	0.00068904	0.75970792	0.68190924	0.57836191
cg17467898	NUDT1;N	7	0.00485228	0.51331966	0.49609943	0.37332319
cg14530679	NUDT9;N	4	0.04489794	0.45382453	0.47180366	0.32774401
cg23878206	NUP62;AT	19	0.00262867	0.89220128	0.86631902	0.75019261
cg08496737	NXF1;NXF	11	0.00375204	0.45331602	0.44977994	0.34910366
cg25505880	NXF1;NXF	11	0.0037804	0.6383535	0.56307443	0.41066401
cg22237425	NXN	17	0.00543186	0.66003562	0.56300653	0.38937308
cg08510234	NXN	17	0.00087577	0.84140558	0.7770986	0.54793023
cg13383734	NXN	17	0.00077582	0.88931922	0.84124924	0.607718
cg21971800	NXN	17	0.01396719	0.48291943	0.48300408	0.34890571
cg03836586	NXPH1	7	0.00247433	0.20355058	0.24543731	0.09341015
cg12502577	NXPH4	12	0.03346287	0.61778787	0.62171749	0.50175773
cg19789466	OAS1;OAS	12	0.04164301	0.75340441	0.76391067	0.6511328

cg04708790	OAS1;OAS	12	0.00298225	0.63610654	0.64118625	0.48719003
cg19371652	OAS2;OAS	12	0.01130868	0.67791879	0.69242202	0.57671928
cg12560128	OAS2;OAS	12	0.00289141	0.67256823	0.65369612	0.42288247
cg27147785	OAS2;OAS	12	0.00114056	0.3801667	0.32680758	0.17739882
cg20870559	OAS2;OAS	12	0.00499723	0.36708634	0.38698012	0.24667258
cg05048523	OBSCN;OI	1	0.00247655	0.86497645	0.85130363	0.73999064
cg03419458	OBSCN;OI	1	0.02794807	0.66794681	0.71534545	0.55436213
cg10948657	OCA2	15	0.04093366	0.84611315	0.80567342	0.68864117
cg24943998	OCA2	15	0.01355936	0.53201263	0.57147016	0.41953102
cg15399926	OCA2	15	0.01074556	0.7745636	0.76250497	0.64744473
cg09966895	ODZ4	11	0.00065949	0.5289542	0.36403069	0.13661835
cg11983942	OPCML	11	0.00118326	0.59760763	0.57814308	0.47028182
cg01875106	OPCML;O	11	0.008876	0.38991934	0.42696551	0.25726426
cg17786206	OPRD1	1	0.00182779	0.33448024	0.37943698	0.22239341
cg10278025	OR12D3	6	0.00791399	0.49898741	0.52123781	0.37862546
cg03019219	OR14J1	6	0.00360641	0.40663567	0.45079916	0.28320141
cg03529662	OR1A1	17	0.02581149	0.37433031	0.36280407	0.2581992
cg03208902	OR1F2P	16	0.0059385	0.50457733	0.55312419	0.31776419
cg05009707	OR2H2	6	0.00538453	0.4678645	0.4676148	0.32406913
cg11246007	OR2H2	6	0.00714351	0.6353632	0.63424784	0.53028965
cg07483650	OR2T4	1	0.00357923	0.58773246	0.58598474	0.46002105
cg05282518	OR4K2	14	0.00401281	0.34007143	0.40317637	0.2332716
cg21052687	OR5D14	11	0.00725151	0.54086412	0.56447658	0.40712097
cg12750151	OR5D18	11	0.00142756	0.46595298	0.51695132	0.35574084
cg18157275	OR5M10;	11	0.00589429	0.51738388	0.55181351	0.38395724
cg10906826	OR6B3	2	0.00177922	0.49877688	0.49481796	0.3836102
cg19364530	OR6F1	1	0.0011414	0.44604524	0.42489899	0.31117783
cg20778958	OR6P1	1	0.00704684	0.54692155	0.60152557	0.38755738
cg04948906	OR8B12	11	0.0091896	0.68089434	0.732089	0.55754479
cg12453675	OR8G1	11	0.00227742	0.22510761	0.22680886	0.10525857
cg27191606	OR8H2	11	0.01366893	0.37050516	0.40331444	0.24090743
cg20886721	OR8U8	11	0.01423002	0.45456956	0.49393348	0.33763264
cg11052535	OR8U8;O	11	0.00184462	0.24566824	0.26741587	0.13773073
cg12011522	OSBPL6;C	2	0.01321271	0.42886427	0.44832495	0.32255739
cg05539509	OSM	22	0.00852388	0.56132228	0.57422091	0.45120825
cg26701390	OSR1	2	0.03895844	0.71801887	0.74573241	0.59952216
cg11943209	OSR1	2	0.04296377	0.61196746	0.580596	0.4577553
cg02172492	OSR1	2	0.01695226	0.71130434	0.78258545	0.5086925
cg22478121	OSR1	2	0.00654053	0.35796962	0.40389183	0.25727318
cg27305303	OTOF	2	0.02991399	0.78109002	0.79388713	0.67576979
cg10465028	OTUB1;O	11	0.00614688	0.4493548	0.43792525	0.29952891
cg20687940	OTUB1;O	11	0.00279729	0.40231373	0.39707923	0.27086783
cg04276301	OTX1	2	0.03533354	0.60939685	0.66157804	0.44330715
cg15745401	P2RY6;P2	11	0.00491697	0.59261319	0.59501991	0.42256237
cg00277342	P2RY6;P2	11	0.01148808	0.82612904	0.8291673	0.71881101
cg02652891	PACRG;PA	6	0.03934159	0.40527489	0.43611111	0.29947565

cg02940070	PACSIN2	22	0.03502867	0.80488838	0.79089663	0.50736336
cg03513388	PADI3	1	0.00835301	0.45259682	0.42529154	0.3203412
cg23228178	PADI4	1	0.00178025	0.58633916	0.5307729	0.40630258
cg16091981	PADI4	1	0.00904361	0.43657139	0.45814874	0.29979232
cg00665650	PAIP2;PAI	5	0.03969411	0.42016686	0.45456015	0.31218865
cg13866253	PAK1;PAK	11	0.00177192	0.90285084	0.8914397	0.79043125
cg24784416	PALLD;PA	4	0.0069951	0.83123586	0.79573069	0.66283741
cg05259872	PALLD;PA	4	0.01614766	0.76405134	0.81351997	0.58486414
cg03822639	PALLD;PA	4	0.04127719	0.78420846	0.81488477	0.66368551
cg08947774	PALLD;PA	4	0.00614711	0.76819164	0.7517786	0.55542478
cg24101009	PANX1	11	0.00073265	0.67670503	0.56157949	0.43197605
cg15542639	PANX1	11	0.00097053	0.8921455	0.84003283	0.73377038
cg00860359	PANX2;PA	22	0.02196399	0.61890507	0.59624099	0.47130236
cg18563886	PAOX;PA(	10	0.03758612	0.57678259	0.64655855	0.4601285
cg00744494	PAPLN	14	0.03213335	0.6216934	0.68873874	0.50109958
cg14094498	PAPPA	9	0.00084594	0.73587326	0.73582303	0.5867832
cg10447404	PARD3	10	0.0198367	0.50460143	0.46378178	0.36151339
cg19017553	PARD3	10	0.00194312	0.84694011	0.78777795	0.6655625
cg06271128	PARD3	10	0.0075368	0.33705872	0.316709	0.19130163
cg23270841	PARD3B;F	2	0.0190357	0.58026598	0.62426674	0.47998229
cg13072214	PARP10	8	0.03169761	0.67236222	0.65892298	0.55803235
cg02346790	PARP15	3	0.00855208	0.46714906	0.54486289	0.34449438
cg20765408	PARP4	13	0.00261671	0.37191975	0.32467972	0.20761342
cg05014660	PARVG;PA	22	0.01598777	0.38285046	0.41212581	0.25492209
cg11163555	PATZ1;PA	22	0.00912728	0.36086494	0.30239734	0.19152587
cg20735988	PBLD;PBL	10	0.02078916	0.33376537	0.31776653	0.20152313
cg26558862	PBRM1;PI	3	0.00066232	0.81397649	0.7268054	0.61642299
cg20765522	PBX2	6	0.01562094	0.56473198	0.56521341	0.46316134
cg27149567	PCBP3;PC	21	0.00511478	0.57238436	0.49732988	0.34153262
cg15901722	PCDH24	5	0.01234308	0.25473948	0.25920877	0.14919585
cg14369318	PCDH7	4	0.0167445	0.60477002	0.61897202	0.43630427
cg10707081	PCDH9;PC	13	0.00303952	0.50147144	0.59855381	0.36450367
cg14205864	PCID2;PCI	13	0.00065949	0.81450117	0.68387945	0.47981716
cg01966277	PCLO	7	0.00431388	0.60792935	0.60777414	0.38743772
cg21234538	PCNT	21	0.00209654	0.89712444	0.89061251	0.69636826
cg07869023	PCSK2	20	0.00228672	0.62463385	0.68713869	0.47814275
cg14453145	PDCD1	2	0.00074801	0.60700313	0.52627138	0.3942086
cg17457379	PDE4DIP	1	0.0025887	0.69841843	0.66543612	0.55796293
cg23933216	PDE4DIP	1	0.0129037	0.42932324	0.42442597	0.32313456
cg10505902	PDE4DIP;I	1	0.00071094	0.75657883	0.70794378	0.59584744
cg11164441	PDE6B;PD	4	0.00107905	0.80817801	0.80252423	0.63432252
cg12546582	PDE7B	6	0.00327239	0.85031818	0.82168939	0.70287089
cg24240349	PDGFA;PD	7	0.00156458	0.79500947	0.71429286	0.60405583
cg20070933	PDGFA;PD	7	0.00092849	0.93078717	0.87317417	0.76525179
cg16375048	PDGFC	4	0.00522751	0.74157015	0.71131874	0.58472548
cg18688704	PDGFC	4	0.03302702	0.38050174	0.42215696	0.25446601

cg03020863	PDGFD;PI	11	0.00248997	0.60905696	0.601946	0.40933681
cg20449614	PDLIM2;P	8	0.03916836	0.74839102	0.77967118	0.63410284
cg02484352	PDLIM4;P	5	0.00065949	0.6720797	0.54219012	0.4187731
cg02383173	PDLIM5;P	4	0.04275864	0.61621508	0.64378632	0.47952431
cg20210051	PDZRN4;I	12	0.00412385	0.55902577	0.53239899	0.38046332
cg04368094	PELI2	14	0.04153301	0.45680362	0.45913298	0.35554389
cg04397884	PELI2	14	0.02002893	0.31970779	0.31279582	0.20996845
cg05014727	PFKFB3	10	0.00230932	0.77791291	0.73003577	0.52960605
cg08994060	PFKFB3	10	0.00249039	0.78608076	0.74331247	0.57666517
cg26262157	PFKFB3	10	0.00317432	0.74974397	0.71207288	0.49620119
cg10517290	PGS1	17	0.00894518	0.71468444	0.70987788	0.54752983
cg21216268	PHC3	3	0.00967401	0.7523979	0.74639278	0.64513904
cg21328779	PHLDB2;F	3	0.00142669	0.26531953	0.28063349	0.12451775
cg16978268	PHLPP1	18	0.03849619	0.37747392	0.33899836	0.22670634
cg02960777	PHTF2;PH	7	0.0019957	0.86481438	0.82966313	0.70886741
cg09304624	PI15	8	0.00812661	0.59267077	0.60755168	0.42815598
cg00961792	PIGR	1	0.00144775	0.59079461	0.58690114	0.48292886
cg21723559	PIGT	20	0.0012838	0.81719282	0.76464762	0.65888701
cg04287259	PIK3CD	1	0.00566397	0.80146019	0.82278386	0.69930699
cg07805542	PIK3CD	1	0.00679225	0.35158199	0.33034314	0.18185622
cg04610450	PIK3R2	19	0.01773077	0.75707281	0.78205035	0.65499566
cg08697847	PIK3R6	17	0.03153468	0.45430855	0.51560754	0.34900123
cg24092340	PIK3R6;PI	17	0.03699135	0.68559421	0.72049816	0.58137368
cg09209669	PIK3R6;PI	17	0.00356659	0.39309857	0.47232666	0.29114487
cg11969526	PIKFYVE;F	2	0.04942609	0.77077078	0.78881012	0.67030268
cg14062643	PIP5KL1;F	9	0.00065949	0.77704003	0.54755399	0.43548877
cg01768814	PITPNC1;I	17	0.03053716	0.3395183	0.33161495	0.19150992
cg00396667	PITX1	5	0.02818766	0.68289335	0.64414216	0.51521344
cg19304088	PITX2;PIT.	4	0.00097618	0.38830745	0.35862105	0.14658473
cg09781994	PITX2;PIT.	4	0.00183659	0.562013	0.52793231	0.32857708
cg13891220	PITX2;PIT.	4	0.0008708	0.3096692	0.39681078	0.1610883
cg21299542	PITX2;PIT.	4	0.00112235	0.2347877	0.24772615	0.12258724
cg03184290	PITX2;PIT.	4	0.00262147	0.54553067	0.51084564	0.31204983
cg26831119	PITX2;PIT.	4	0.00689691	0.51869661	0.53830217	0.31048188
cg01951086	PITX2;PIT.	4	0.00597821	0.46139688	0.44205677	0.25039692
cg02725370	PITX2;PIT.	4	0.00220808	0.42290875	0.42053921	0.20964348
cg21029045	PITX2;PIT.	4	0.00614711	0.24580707	0.26557685	0.12641169
cg26708319	PITX2;PIT.	4	0.00144772	0.44600076	0.44699544	0.2261066
cg03943773	PITX2;PIT.	4	0.00126466	0.27669286	0.34842048	0.15057719
cg24925400	PITX2;PIT.	4	0.00217555	0.41818305	0.4059569	0.20304859
cg09854515	PITX2;PIT.	4	0.01422483	0.6324812	0.66325797	0.42999911
cg19829847	PITX2;PIT.	4	0.01235814	0.6683175	0.67196263	0.48716407
cg06842954	PITX2;PIT.	4	0.00205127	0.62742674	0.61694351	0.46687514
cg13385016	PITX2;PIT.	4	0.02505273	0.68473913	0.67269696	0.54736984
cg13736376	PITX2;PIT.	4	0.00231646	0.30524715	0.31177577	0.1837677
cg20291436	PITX2;PIT.	4	0.00327239	0.45785196	0.48168492	0.26841466

cg03972665	PITX2;PIT	4	0.00262855	0.63554802	0.64664836	0.4676108
cg08979895	PITX2;PIT	4	0.03314909	0.51551706	0.53151706	0.36420906
cg10055501	PITX2;PIT	4	0.00260155	0.71549236	0.70247614	0.53410086
cg21726372	PITX2;PIT	4	0.00206218	0.70520046	0.67360364	0.479284
cg03144922	PITX2;PIT	4	0.00159552	0.74746572	0.73822659	0.5846897
cg27398720	PITX2;PIT	4	0.0039383	0.53194417	0.58967303	0.35109594
cg22914729	PITX2;PIT	4	0.00314361	0.44549644	0.50510539	0.26596403
cg01371072	PITX2;PIT	4	0.00503319	0.40539642	0.41530378	0.24569048
cg17507671	PITX2;PIT	4	0.00486472	0.28068829	0.30243505	0.17719793
cg05030680	PITX2;PIT	4	0.00648443	0.46116085	0.45011728	0.33321342
cg16184803	PITX2;PIT	4	0.00186737	0.29588998	0.3111806	0.18181663
cg22717014	PITX2;PIT	4	0.00429383	0.51042695	0.48968255	0.35149869
cg05835105	PITX2;PIT	4	0.00349009	0.38582784	0.3630821	0.20230751
cg14308375	PITX2;PIT	4	0.00183809	0.33218246	0.35999535	0.19142693
cg17929238	PITX2;PIT	4	0.02568798	0.51891235	0.58085871	0.3767503
cg03594078	PIWIL2;PI	8	0.04178799	0.75542286	0.77469338	0.62134913
cg12858593	PIWIL2;PI	8	0.00429038	0.64480283	0.58019455	0.40231261
cg00163702	PKD1L2;P	16	0.00403008	0.76372271	0.7820004	0.65722366
cg09270285	PKNOX2	11	0.00554761	0.48591744	0.49422072	0.32971722
cg22193393	PLA2G4E	15	0.0269694	0.60608334	0.63666199	0.47117543
cg18981758	PLA2G6;P	22	0.00081925	0.78034892	0.69908065	0.5844309
cg09920804	PLAT;PLA	8	0.00109472	0.79054776	0.71060147	0.53027849
cg21931938	PLCB2	15	0.0035378	0.36511999	0.40750958	0.22794249
cg24624873	PLCE1;PL	10	0.00244246	0.60684582	0.66188813	0.46853252
cg16503724	PLCL2;PL	3	0.00284268	0.46149644	0.4315222	0.30067028
cg21535931	PLEC1;PL	8	0.01540791	0.78710831	0.75442334	0.59561787
cg20784950	PLEC1;PL	8	0.02995719	0.62225335	0.6180579	0.45283798
cg22258045	PLEC1;PL	8	0.01488914	0.73749262	0.73450049	0.62324364
cg02861056	PLEK;PLE	2	0.00287155	0.67335092	0.63867553	0.50763305
cg13060970	PLEK;PLE	2	0.00180518	0.69315673	0.65270155	0.53274352
cg04872689	PLEK;PLE	2	0.00303201	0.70120982	0.6561953	0.46162183
cg10812236	PLEK;PLE	2	0.00367708	0.71189908	0.68397101	0.46021285
cg02556345	PLEKHA1;	10	0.00148902	0.66091974	0.57909694	0.42885452
cg01073837	PLEKHA2	8	0.00280849	0.39349029	0.455464	0.26490026
cg01767200	PLEKHG4	5	0.01083395	0.86849829	0.86686083	0.75641235
cg07533239	PLEKHG6;	12	0.00157573	0.68918472	0.65506219	0.52349596
cg11524454	PLEKHG7	12	0.00104452	0.62881542	0.58007842	0.39139452
cg00186909	PLEKHG7	12	0.00989055	0.6838955	0.74871192	0.54356733
cg05939149	PLEKHH2	2	0.00358325	0.86182377	0.83631606	0.7275461
cg05652225	PLXDC1	17	0.00146929	0.51883958	0.4706007	0.35542896
cg02736746	PLXNA2	1	0.00971058	0.5105028	0.50649338	0.35911959
cg16850690	PLXND1	3	0.0127705	0.76615982	0.75604867	0.59389426
cg01117384	PMEPA1;	20	0.00126804	0.49532758	0.43789317	0.28631802
cg00587342	PMM1	22	0.00263672	0.70015141	0.62257845	0.49722514
cg12894984	PNMT	17	0.00220808	0.56660222	0.55635113	0.42138851
cg22027204	PNPLA1;P	6	0.00242331	0.64282646	0.61322262	0.39534057

cg17921363	POFUT2;F	21	0.03301459	0.75378499	0.69684818	0.5852519
cg05673882	POLK	5	0.00115897	0.51613573	0.46344993	0.24520064
cg00201870	POM121L	7	0.00435264	0.54870717	0.56790844	0.42945459
cg21862081	POMT2	14	0.00109472	0.77777389	0.73932552	0.63080262
cg10324703	POU6F2;F	7	0.0048909	0.4278685	0.42310611	0.32008819
cg07676920	PPARG;PF	3	0.01398164	0.82471081	0.78448063	0.68119807
cg20872072	PPM1G;P	2	0.00295711	0.78903519	0.75399416	0.65117006
cg20793532	PPP2R2C;	4	0.01608519	0.45048462	0.42400625	0.28988013
cg22325673	PPP2R2C;	4	0.00232154	0.57664664	0.57260328	0.46634353
cg20910008	PPP3CC	8	0.00546612	0.85883478	0.86242542	0.69637199
cg08752433	PPTC7	12	0.01296171	0.82934643	0.8371621	0.70646812
cg23878241	PPTC7	12	0.00742222	0.63503114	0.63038801	0.49411371
cg27382409	PPYR1	10	0.00152405	0.50196343	0.4764207	0.36353891
cg01556246	PRAGMIN	8	0.00314361	0.44120451	0.45894439	0.26865039
cg27345534	PRB1;PRB	12	0.00908203	0.76060902	0.74557889	0.64178558
cg08369079	PRDM1;P	6	0.02679222	0.63718566	0.65691168	0.49815679
cg08381918	PRDM15;	21	0.01183594	0.80356374	0.87771255	0.70132756
cg04852443	PRDM16;	1	0.01317683	0.63588268	0.64240248	0.48363555
cg09321238	PRDM16;	1	0.01237894	0.7119099	0.72783467	0.49893011
cg01104489	PRDM16;	1	0.01864666	0.69086762	0.72632518	0.51104719
cg01448098	PRDM16;	1	0.02792018	0.68418652	0.71520522	0.5402479
cg20699701	PRDM16;	1	0.03722047	0.61143814	0.63431342	0.50880523
cg18759102	PRDM16;	1	0.01123239	0.7191506	0.792186	0.60614312
cg06099439	PRDM16;	1	0.00704342	0.87126556	0.85461876	0.74314817
cg03527274	PRDM16;	1	0.00441129	0.74880072	0.71917458	0.58097177
cg03082523	PRDM16;	1	0.00233439	0.87969412	0.88150116	0.76392872
cg27187555	PRDM16;	1	0.01073914	0.66668249	0.63253036	0.51038922
cg01085125	PRDM6	5	0.01935097	0.68227237	0.66946865	0.4436029
cg04503968	PRDM6	5	0.00303542	0.72992054	0.71684243	0.49082441
cg02300584	PRDM6	5	0.02521286	0.56797995	0.58361717	0.41096544
cg05569742	PRDM6	5	0.02763355	0.58685857	0.60743045	0.39914958
cg07741162	PRDM6	5	0.00539114	0.66555588	0.72172033	0.43069326
cg03729337	PRDM6	5	0.01526804	0.7151257	0.77596994	0.56033627
cg02564291	PRDM6	5	0.02087024	0.65104212	0.69068826	0.48722488
cg17786697	PRDM6	5	0.00582851	0.64332595	0.66056256	0.42567797
cg03776662	PRDM6	5	0.01064534	0.32399029	0.33620755	0.2238315
cg07799386	PRDM6	5	0.0029375	0.41962913	0.43837379	0.29552047
cg05668996	PRDM6	5	0.00891192	0.52543137	0.64156472	0.41733463
cg15262954	PRIC285;F	20	0.01789965	0.69402761	0.7449294	0.57065809
cg20584157	PRICKLE2	3	0.00264208	0.7367312	0.68405043	0.53326166
cg03567939	PRKAG2;F	7	0.00065949	0.80688996	0.70033042	0.57853583
cg06728793	PRKAR1B;	7	0.0033331	0.77332638	0.74649075	0.57652563
cg12304937	PRKAR1B;	7	0.00134844	0.75420168	0.72736355	0.61936062
cg04721825	PRKCA	17	0.01471848	0.70002345	0.75642827	0.57442115
cg03543319	PROKR1	2	0.0154705	0.47007232	0.50708351	0.32260502
cg17626960	PRRT1	6	0.04839114	0.57504635	0.65156125	0.46026472

cg02897989	PRRX1;PR	1	0.02878476	0.5305222	0.56337835	0.42723014
cg25181170	PRTFDC1	10	0.00348701	0.82683107	0.88817701	0.71420539
cg20633213	PRTFDC1	10	0.00245414	0.86023255	0.83766551	0.71377737
cg17004025	PRTN3	19	0.03657758	0.50282193	0.48643338	0.38416981
cg13914324	PRUNE2	9	0.02944061	0.46073466	0.49407313	0.33630732
cg07526408	PSG10	19	0.04399614	0.36843111	0.3608506	0.23918463
cg16197388	PSG3	19	0.01760238	0.4419504	0.46118417	0.32157338
cg11706729	PSMB8;PS	6	0.01499384	0.74380283	0.73250291	0.5763667
cg02567488	PSMB9;PS	6	0.0026334	0.4135236	0.42128451	0.2334967
cg18369034	PTGDR	14	0.00073547	0.71205732	0.58773081	0.36403176
cg23022053	PTGDR	14	0.01268304	0.72996203	0.62028031	0.36405342
cg01091117	PTGER4	5	0.01158752	0.61768557	0.62968191	0.43838002
cg16060930	PTGFRN	1	0.00810421	0.74897317	0.68332049	0.32454319
cg15430294	PTGIR	19	0.01678972	0.63986039	0.67096228	0.52080401
cg11641102	PTK2B;PT	8	0.00198593	0.55530141	0.48224221	0.3017133
cg21578195	PTK6	20	0.02970062	0.57291558	0.57090591	0.45300368
cg25143652	PTK6;PTK	20	0.00689691	0.66836685	0.68231881	0.47099144
cg20386404	PTPN14	1	0.00213077	0.46718738	0.43333415	0.24188129
cg15702949	PTPN18;P	2	0.02103552	0.52768808	0.52882597	0.42647209
cg07111834	PTPN22;P	1	0.04433808	0.73907995	0.76678834	0.62567758
cg09887955	PTPN7;PT	1	0.00658287	0.53463717	0.57874623	0.42265368
cg00238353	PTPRE	10	0.00882698	0.41647183	0.32454706	0.22338099
cg07053114	PTPRE	10	0.00581095	0.57791578	0.56885353	0.42283845
cg07986469	PTPRE	10	0.00190306	0.5887042	0.53707034	0.35607634
cg03900492	PTPRN2;P	7	0.00084179	0.55999299	0.58812908	0.40232911
cg04996300	PTPRN2;P	7	0.00441677	0.58759431	0.56011579	0.42282245
cg22128328	PTPRN2;P	7	0.00271748	0.54084291	0.49396859	0.37227356
cg25910519	PTPRN2;P	7	0.01174797	0.45094549	0.43257909	0.31530863
cg03232056	PTPRN2;P	7	0.01093065	0.42286764	0.44823845	0.30165996
cg14468481	PTPRN2;P	7	0.00349009	0.55918117	0.53388803	0.35986844
cg24842334	PTPRN2;P	7	0.02291377	0.48435138	0.51810349	0.37010982
cg14582691	PTPRN2;P	7	0.01530568	0.71656711	0.73725191	0.57596557
cg17429772	PTPRN2;P	7	0.0026461	0.26992465	0.26337748	0.15796699
cg22337920	PTPRN2;P	7	0.00220808	0.51502596	0.49953336	0.39693698
cg12434889	PTPRN2;P	7	0.00629716	0.39488973	0.37746303	0.26479943
cg02100997	PTPRU;PT	1	0.01471848	0.45209188	0.51917459	0.34571415
cg07856761	PVALB	22	0.01767742	0.44735816	0.44921001	0.32676777
cg23898497	PVT1	8	0.02895902	0.61807019	0.58897458	0.48514843
cg26753608	PVT1	8	0.00092387	0.86067737	0.79345426	0.67270761
cg03402443	PWRN1	15	0.02009425	0.69008463	0.68057836	0.57703816
cg08216099	PXDN	2	0.00066232	0.76245067	0.60752914	0.39400701
cg08293102	PYGL;PYG	14	0.01329117	0.64903545	0.67238655	0.53150454
cg05732883	PYROXD2	10	0.00971274	0.80707372	0.82306341	0.6947203
cg26712743	PYROXD2	10	0.00173154	0.57171597	0.5669787	0.41621781
cg17960615	RAB31	18	0.00229687	0.75185997	0.73315032	0.48810171
cg14066471	RAB37	17	0.00594191	0.31521377	0.29578619	0.179134

cg22213445	RAB7A	3	0.0439181	0.65302136	0.66747795	0.53527403
cg10777887	RAB8B	15	0.00840111	0.77410119	0.77806659	0.6430727
cg02747950	RAB8B	15	0.02377716	0.7320101	0.74670616	0.60535679
cg25603108	RAD51L1;	14	0.00597656	0.42271126	0.3603137	0.23439398
cg08297686	RAD51L1;	14	0.00346969	0.71773613	0.7224274	0.50739825
cg14260083	RADIL	7	0.01093065	0.68891098	0.68231204	0.57033401
cg14083146	RALGAPA	20	0.00476313	0.55600252	0.58598141	0.39882793
cg08622666	RANBP9	6	0.00205947	0.81866056	0.80253868	0.68977426
cg06205333	RAP1A;RA	1	0.00969263	0.71361962	0.68402163	0.50234696
cg18553223	RAP1GAP	17	0.02086555	0.58993907	0.65381179	0.45929516
cg09981407	RAP1GAP	17	0.03389831	0.46477537	0.50043223	0.35271862
cg13784312	RAPGEF1	9	0.02447745	0.76667701	0.77419666	0.58908689
cg13655250	RAPGEF1;	9	0.00637687	0.6326555	0.61645989	0.46883233
cg22900476	RAPGEF4	2	0.00336153	0.77104588	0.78074518	0.57550274
cg11094248	RARA;RAI	17	0.02507257	0.75975343	0.77231133	0.61730559
cg07944862	RARA;RAI	17	0.04373093	0.70784114	0.69670443	0.5954753
cg14986890	RARRES1;	3	0.00160273	0.56700896	0.40105044	0.22346944
cg04402088	RASA3	13	0.00065949	0.86809453	0.79264078	0.67381541
cg10976218	RASA3	13	0.0037804	0.72431869	0.67053654	0.49406711
cg11288831	RASA3	13	0.00826879	0.75981498	0.76039301	0.53182961
cg15972148	RASA3	13	0.00412512	0.84875499	0.82732851	0.68262246
cg16046375	RASA3	13	0.00368022	0.76041573	0.7154932	0.423456
cg01811815	RASA3	13	0.00947108	0.74647489	0.79541852	0.57399068
cg05504085	RASAL3	19	0.00576931	0.45071897	0.42575496	0.30419199
cg04707603	RASGRP3,	2	0.02317852	0.69957431	0.69484817	0.57918234
cg01932734	RASSF1;R.	3	0.00859639	0.50125062	0.59142529	0.37926642
cg12691994	RASSF5;R.	1	0.02766761	0.71637454	0.72662773	0.61002518
cg10992219	RB1CC1;R	8	0.00113577	0.76836338	0.7546596	0.53292197
cg15168816	RBM24;RI	6	0.00242331	0.40613645	0.39339081	0.26503785
cg14466942	RBM24;RI	6	0.00185385	0.65933208	0.6612608	0.50755679
cg02066331	RBM24;RI	6	0.00758031	0.5334861	0.53303184	0.41057786
cg00137234	RBM46	4	0.01946986	0.43261144	0.46728661	0.31071442
cg22062068	RBMXL2	11	0.01653351	0.60817802	0.6163024	0.45588893
cg02837128	RBPMS;RI	8	0.02002893	0.45624339	0.46406965	0.33234865
cg02552250	RCBTB2	13	0.01332118	0.59635073	0.64087277	0.48624115
cg09829263	RCBTB2	13	0.00961487	0.58244547	0.65295922	0.4502562
cg11093142	RCBTB2	13	0.00369903	0.79722288	0.82930461	0.58955127
cg15359390	REG1P	2	0.00081789	0.6686578	0.64589434	0.53174631
cg15692837	REG3A;RE	2	0.00168532	0.49606112	0.53284006	0.36818407
cg07184423	REPS1;RE	6	0.03302702	0.68851715	0.73222338	0.57653972
cg02098619	RERE;RER	1	0.01414127	0.60243576	0.64157979	0.48787817
cg20316440	RERE;RER	1	0.03954748	0.52659856	0.53777138	0.40650625
cg10464462	RFFL	17	0.01730009	0.61428901	0.66608839	0.49790368
cg11184109	RFT1	3	0.00513612	0.59418498	0.53336149	0.35996669
cg13026795	RFTN1	3	0.00685368	0.51529296	0.55020574	0.37106945
cg00506299	RFTN1	3	0.02568798	0.86525863	0.84714983	0.73903898

cg04381957	RFTN1	3	0.00279686	0.68864843	0.72708453	0.45993289
cg00259834	RFTN1	3	0.00367708	0.59331786	0.58553404	0.33298749
cg05991009	RFX8	2	0.00741553	0.82135756	0.85057938	0.68153282
cg15611912	RGS10	10	0.00227382	0.58712575	0.54100683	0.35283851
cg18944752	RGS12;RG	4	0.00103895	0.41658825	0.29535629	0.18862914
cg25461508	RGS14	5	0.02991399	0.33010848	0.33780273	0.21627619
cg24028809	RGS17	6	0.01040141	0.70531849	0.69883743	0.45973363
cg10299383	RGS20;RG	8	0.00134295	0.66853241	0.65593129	0.50082114
cg11993173	RGS20;RG	8	0.02944061	0.51901153	0.53158018	0.34111377
cg13604445	RGS3;RGS	9	0.00306113	0.44138396	0.42055116	0.22439844
cg18515872	RGS6	14	0.00572656	0.62020292	0.58249106	0.39172924
cg21899942	RGS6	14	0.01959183	0.69928016	0.70413799	0.54739969
cg05111779	RGS6	14	0.00224325	0.62681605	0.55561651	0.41593811
cg25329734	RGS9;RGS	17	0.00355472	0.24112518	0.23923106	0.12947829
cg24497361	RHOG	11	0.00663932	0.51147222	0.50254023	0.38200469
cg07768107	RHOH	4	0.00703549	0.63781931	0.69302401	0.49863043
cg16086237	RIMBP2	12	0.0019386	0.50629359	0.49390028	0.38355626
cg23373401	RIMBP2	12	0.00401824	0.60102089	0.61575093	0.48883603
cg05939560	RIMBP2	12	0.00186404	0.49087814	0.50381834	0.35289737
cg00009196	RIN2	20	0.00386127	0.52145108	0.58243365	0.35711374
cg02902617	RIN3	14	0.00429038	0.87304626	0.86959272	0.75390197
ch.14.14889{	RIN3	14	0.04006803	0.42301545	0.44905051	0.31201634
cg10197238	RNASE1;R	14	0.00575836	0.60169267	0.65803701	0.48976187
cg12940993	RNASE1;R	14	0.00272772	0.499697	0.53375849	0.35898835
cg18294707	RNASE2	14	0.00553815	0.40275044	0.41315459	0.26959761
cg07525077	RNASE3	14	0.03954748	0.74588418	0.74882568	0.61217724
cg00502926	RNASE7	14	0.03547817	0.33974243	0.33791231	0.20799762
cg01715025	RNF115	1	0.00806395	0.73263114	0.6661746	0.55795443
cg03191504	RNF149	2	0.00134295	0.83768754	0.73088863	0.6229763
cg06418113	RNF165	18	0.0172362	0.58540332	0.57724419	0.42127233
cg06788514	RNF166	16	0.01571591	0.54399805	0.5235326	0.42040203
cg16789707	RNF166	16	0.00857641	0.44270174	0.45163372	0.31152734
cg23146741	RNF170;R	8	0.01771787	0.84940358	0.85662292	0.72938319
cg24820936	RNF19A;R	8	0.00367963	0.56482657	0.58066006	0.37712825
cg11519930	RNF220	1	0.03349395	0.54775431	0.54952838	0.43269239
cg01942845	RNU5E;RN	5	0.01021897	0.27146518	0.31843832	0.15825063
cg03577157	RNU5E;SS	5	0.01632034	0.49875899	0.42499405	0.29617173
cg03572680	ROBO4	11	0.00230614	0.58577677	0.49921336	0.39826331
cg07906432	ROR1;ROI	1	0.00894518	0.72758002	0.70199711	0.59661297
cg14244439	ROR2	9	0.00415941	0.80020667	0.817619	0.65494878
cg01400040	RORA	15	0.00947108	0.90829537	0.87212582	0.77098815
cg18216249	RPAP3;RP	12	0.00853767	0.39993191	0.48497459	0.2990903
cg20491963	RPL31P11	1	0.00997311	0.73645744	0.75588953	0.63084832
cg13271206	RPS3	11	0.00805596	0.88771359	0.87929992	0.77031823
cg16490823	RPS6KA2	6	0.00298225	0.23864256	0.24073924	0.13699967
cg03849851	RPS6KA2;	6	0.00065949	0.85439538	0.75890855	0.60148837

cg23002590	RPS6KA2;	6	0.00830789	0.60637925	0.66386871	0.41191311
cg24667756	RPTOR;RF	17	0.00209538	0.69003222	0.62874493	0.48081985
cg22335826	RREB1;RR	6	0.001879	0.4676709	0.45347094	0.29308877
cg26932790	RRN3P1	16	0.00065949	0.4627941	0.32875347	0.21833948
cg12132508	RRP15	1	0.00070285	0.75201602	0.67106687	0.48260915
cg10959651	RSAD2	2	0.0301561	0.55063598	0.59010153	0.44671457
cg24249648	RSPH3	6	0.00065949	0.87701059	0.77183796	0.56880077
cg05879505	RTN4RL1	17	0.02047468	0.47899501	0.54248399	0.35987704
cg15091747	RUNX1	21	0.00157573	0.63110807	0.55411876	0.3789232
cg05973398	RUNX1	21	0.01678444	0.49586371	0.43465596	0.31265253
cg19836199	RUNX1	21	0.0037395	0.59761127	0.63725166	0.37510255
cg08443845	RUNX1	21	0.00260306	0.56523266	0.48684445	0.31684499
cg01519261	RUNX1;RU	21	0.00156239	0.69162447	0.60631068	0.37018977
cg04915566	RUNX1;RU	21	0.00127437	0.71818363	0.61043267	0.30000007
cg13030790	RUNX1;RU	21	0.00081789	0.35121355	0.23945248	0.10613821
cg04554131	RUNX3	1	0.03853559	0.613131	0.66835719	0.46759167
cg09993145	RUNX3	1	0.00113883	0.77542805	0.78673372	0.6321794
cg13461622	RUNX3;RU	1	0.0067146	0.81815956	0.80763628	0.70290044
cg14236758	RXRA	9	0.02196399	0.69968195	0.78467551	0.5301501
cg10774802	RYR1;RYR	19	0.00872502	0.68251824	0.73740201	0.56807383
cg03181524	RYR1;RYR	19	0.02332665	0.70980271	0.72571869	0.54602877
cg01411912	S100A4;S	1	0.00091908	0.72109898	0.63224482	0.50156555
cg24691453	S100A4;S	1	0.00132036	0.42855654	0.37573269	0.18792233
cg13091627	S100A4;S	1	0.00186737	0.57045957	0.53716261	0.33563628
cg21217846	S100A5	1	0.00079224	0.85181444	0.76415087	0.60684673
cg26303281	S100A5	1	0.00147518	0.4087255	0.35684232	0.23938775
cg03514239	S100A9	1	0.02800741	0.58989628	0.63046347	0.48531259
cg14742715	SAA1;SAA	11	0.01465773	0.48444297	0.54969414	0.35374768
cg15484375	SAA1;SAA	11	0.00176161	0.53115695	0.52904678	0.29000213
cg12907644	SAA2;SAA	11	0.00328917	0.60888528	0.63063369	0.43380092
cg09314984	SAFB	19	0.0301561	0.33918168	0.36064742	0.23616971
cg02507889	SALL3	18	0.01111549	0.73966856	0.77259103	0.61081362
cg17018082	SAMD12;S	8	0.00639169	0.74200126	0.70420814	0.58512242
cg20466954	SAMD13;S	1	0.00619019	0.24941762	0.29258506	0.14891021
cg16554153	SAMD9	7	0.02856411	0.59999027	0.54081353	0.35968802
cg08986767	SAMD9;SA	7	0.00313278	0.33556313	0.37493457	0.17208537
cg04554576	SATB2	2	0.02657818	0.62986468	0.62973838	0.48462075
cg06876053	SBF2	11	0.00748334	0.48556463	0.47543338	0.21662212
cg14068721	SBK2	19	0.01561142	0.65067477	0.66655964	0.51648197
cg17297634	SCARA5	8	0.00216509	0.46945497	0.45378874	0.34491428
cg03349134	SCGN	6	0.002776	0.41089384	0.44404074	0.24179459
cg18751231	SCHIP1	3	0.01670995	0.71656906	0.72140925	0.59749924
cg20314918	SCN2A;SC	2	0.00185385	0.71555628	0.72042976	0.59322902
cg09680149	SCNN1A	12	0.00085355	0.42952477	0.37573315	0.23061545
cg20366549	SCNN1A;S	12	0.03160477	0.5060367	0.52337455	0.37031766
cg03033182	SCOC	4	0.00136593	0.58509994	0.48009606	0.33175352

cg05648303	SCOC;SCC	4	0.00314361	0.64763316	0.63302004	0.44965877
cg11183307	SCRT2	20	0.01687995	0.59053701	0.68567497	0.44349042
cg26736232	SCT	11	0.00614688	0.7896703	0.77545673	0.58200977
cg10944063	SCTR	2	0.01672645	0.59466313	0.61279858	0.48572407
cg01530803	SDC4P	22	0.00669348	0.75938012	0.73220801	0.60168352
cg06968788	SDC4P	22	0.00065949	0.81321299	0.74011525	0.62351503
cg00295780	SDCCAG8	1	0.01164121	0.60609337	0.53847826	0.42531943
cg11859398	SDK1	7	0.04627729	0.65137616	0.63831184	0.5066845
cg27005946	SDK1	7	0.00907575	0.42297128	0.4900743	0.31944018
cg17756105	SDK1	7	0.00674191	0.38226623	0.3604258	0.24775939
cg16250868	SDK1	7	0.00362881	0.50784031	0.44059997	0.26770489
cg25274735	SDK1	7	0.00313278	0.6329878	0.63405727	0.50941921
cg04004830	SDPR	2	0.04671332	0.66550005	0.70678101	0.55630166
cg16790374	SDR16C5	8	0.0012838	0.65890082	0.68391613	0.55247793
cg05149586	SDSL	12	0.00422127	0.35182524	0.37129099	0.20365503
cg25750901	SEC14L3	22	0.00486472	0.39258641	0.40843633	0.28282788
cg13855364	SEC16A	9	0.00144767	0.88602668	0.88046063	0.7455955
cg16640599	SEC24D	4	0.00457113	0.7995553	0.761428	0.58823763
cg09043518	SECTM1	17	0.01088059	0.78354351	0.77033226	0.62261039
cg09461545	SEL1L3	4	0.00648443	0.53669399	0.51422404	0.3950791
cg19853494	SELL	1	0.01446542	0.4623828	0.51185531	0.31592168
cg25165932	SELPLG	12	0.00648443	0.40260068	0.42721367	0.26009142
cg22328644	SELPLG	12	0.01905936	0.6608504	0.6988497	0.55890764
cg01477253	SEMA4F	2	0.00073118	0.9083583	0.84642634	0.71772942
cg17679604	SEMA6A	5	0.00279494	0.43136254	0.36808209	0.18199231
cg20015689	SEMG1	20	0.03699135	0.61156859	0.62032134	0.50189239
cg05474605	SEPP1;SEI	5	0.00160273	0.4407861	0.4175165	0.19200226
cg00934987	SEPT4;SEI	17	0.00219764	0.52282545	0.53392299	0.32484368
cg14773822	SEPT9;SEI	17	0.00935236	0.65281584	0.53709971	0.43009693
cg16293484	SEPT9;SEI	17	0.01714791	0.57563708	0.53604115	0.42299814
cg22056755	SERAC1	6	0.00487618	0.80257316	0.82568485	0.693117
cg18939081	SERINC2	1	0.00228407	0.75809842	0.71993455	0.61960624
cg13357229	SFRP2	4	0.03349395	0.50631591	0.49609611	0.33109484
cg00705808	SFRP2	4	0.02633873	0.37228393	0.41421781	0.26170124
cg21630608	SFRP2	4	0.01807343	0.51517049	0.60622846	0.40603077
cg04959480	SFRP2	4	0.00548311	0.73068518	0.78037268	0.62337112
cg20054157	SFTA1P	10	0.01806244	0.50027218	0.38121696	0.2770363
cg20393620	SGK1	6	0.00798815	0.26597787	0.28193169	0.14399573
cg04905719	SGK1	6	0.00225589	0.51608756	0.51721265	0.26646988
cg09404376	SGK1;SGK	6	0.00144352	0.45395661	0.43087992	0.23483101
cg02904344	SGK1;SGK	6	0.00242331	0.74840429	0.74560651	0.5168093
cg26501657	SH2B3	12	0.00277425	0.75301534	0.74333716	0.61648861
cg27003165	SH2D1B	1	0.00141191	0.47258097	0.48138354	0.34089361
cg20839149	SH2D2A;N	1	0.00112849	0.23827607	0.23095343	0.10282656
cg06888746	SH3PXD2A	10	0.00195549	0.7368909	0.67880874	0.41207983
cg23786152	SH3PXD2A	10	0.00099363	0.69636915	0.58216092	0.4533611

cg16572224	SH3PXD2I	5	0.00094183	0.80142739	0.7554539	0.59848181
cg19027424	SH3PXD2I	5	0.01310065	0.77088759	0.79674911	0.59075392
cg18135683	SH3PXD2I	5	0.00263954	0.65465406	0.61159831	0.4658646
cg09178384	SH3RF1	4	0.00291542	0.46127065	0.47532004	0.32705717
cg16635948	SH3RF3	2	0.0086408	0.79523843	0.78036443	0.5984554
cg26645082	SHANK2	11	0.00218063	0.56706169	0.56669912	0.27510696
cg04404381	SHANK2	11	0.00347082	0.54864003	0.56617581	0.33604958
cg13813366	SHISA3	4	0.01980843	0.82730361	0.84606935	0.6811133
cg07631533	SHISA9	16	0.00864149	0.61002114	0.61969652	0.50233043
cg03222834	SHISA9	16	0.00435264	0.72330368	0.70960211	0.58245032
cg19162496	SHROOM.	4	0.00521564	0.6138162	0.62079909	0.46764061
cg20311846	SHROOM.	4	0.00283173	0.32354753	0.35660471	0.15723192
cg11615509	SHROOM.	4	0.00228407	0.5619556	0.51550337	0.3620977
cg04503593	SHROOM.	4	0.00101054	0.83458016	0.79431235	0.65623606
cg04461123	SHROOM.	4	0.01160447	0.8490456	0.85170178	0.69719281
cg27417997	SHROOM.	4	0.00228672	0.54862728	0.51130441	0.36676136
cg27086014	SIGLEC12,	19	0.01356792	0.61273092	0.68689691	0.50552232
cg08861404	SIGLECP3	19	0.00445086	0.45597947	0.46918963	0.3347476
cg21982437	SIGLECP3	19	0.00375204	0.35011367	0.38231686	0.21989461
cg17610755	SIK1	21	0.0009078	0.35165642	0.33029814	0.19310659
cg09458566	SIK3	11	0.0135118	0.67668729	0.65164669	0.52097925
cg00407329	SIM1	6	0.04685951	0.42058552	0.40244864	0.29767902
cg06246999	SIM1	6	0.00768774	0.38448957	0.43222692	0.27212079
cg27248980	SIM2;SIM	21	0.02362077	0.60997445	0.60774554	0.41164295
cg22711869	SIM2;SIM	21	0.03889955	0.77637821	0.82888298	0.6685555
cg22289831	SIM2;SIM	21	0.02888438	0.58533878	0.61773028	0.4514566
cg21697851	SIM2;SIM	21	0.01465218	0.69645254	0.71789542	0.59592804
cg08066129	SIM2;SIM	21	0.00277351	0.60033157	0.69011516	0.4112218
cg19498960	SIM2;SIM	21	0.00238358	0.51385432	0.59564682	0.30329659
cg07615087	SIM2;SIM	21	0.00418817	0.70926305	0.76748049	0.44667345
cg10456990	SIM2;SIM	21	0.00359124	0.66608292	0.69045185	0.35565513
cg02701080	SIM2;SIM	21	0.03753328	0.68575742	0.71021421	0.50978077
cg00698204	SIM2;SIM	21	0.00542585	0.67175405	0.80419954	0.44522877
cg15750546	SIM2;SIM	21	0.02041679	0.70145276	0.69109091	0.48166372
cg01090834	SIM2;SIM	21	0.02511511	0.70935043	0.75910221	0.57162643
cg11803771	SIPA1;SIP.	11	0.03173228	0.76735903	0.77930095	0.60458263
cg04466898	SIPA1;SIP.	11	0.036552	0.68355896	0.73294368	0.54707032
cg24757937	SIX1	14	0.01863458	0.74740353	0.7547448	0.60046364
cg10397765	SIX1	14	0.03148743	0.4253611	0.41927213	0.25520409
cg23209353	SKA3;SKA	13	0.04237386	0.91011158	0.8625557	0.73710308
cg26017930	SKI	1	0.01782206	0.45043153	0.46429833	0.34690323
cg22845912	SLA;TG;SL	8	0.00102765	0.71891146	0.62163003	0.51870701
cg03841065	SLA2;SLA2	20	0.00615082	0.49249582	0.53920805	0.39244048
cg07598052	SLA2;SLA2	20	0.00659076	0.52078154	0.55144778	0.39817179
cg08495185	SLA2;SLA2	20	0.00591291	0.31042689	0.31792818	0.18729962
cg01710351	SLAMF1	1	0.03068939	0.72898304	0.77009274	0.62153484

cg04275881	SLAMF8	1	0.0018139	0.46113816	0.44466393	0.24885989
cg07625783	SLAMF8;S	1	0.0013706	0.59800567	0.53219521	0.31948103
cg21301505	SLAMF8;S	1	0.0025513	0.62889716	0.56918428	0.42316176
cg06764092	SLAMF8;S	1	0.00134295	0.6378973	0.62889169	0.42599933
cg26417912	SLAMF9;S	1	0.00079512	0.74607018	0.67847587	0.57521387
cg11742202	SLC17A5	6	0.00197538	0.51033625	0.60621972	0.38827015
cg13844463	SLC1A2	11	0.01585898	0.597148	0.54443583	0.44039959
cg08258650	SLC1A2	11	0.03174292	0.64111156	0.64916896	0.44711387
cg07254421	SLC1A3;SI	5	0.00660273	0.60215533	0.49976598	0.34697808
cg01823958	SLC1A7	1	0.02445578	0.55471824	0.5237467	0.3744105
cg00532936	SLC1A7	1	0.0013661	0.68765105	0.64559988	0.48502673
cg18105725	SLC22A1;S	6	0.01678972	0.55833255	0.6026825	0.42641038
cg18458509	SLC22A18	11	0.00413669	0.56672656	0.54944582	0.35576658
cg23190089	SLC22A18	11	0.00521564	0.26886311	0.25547738	0.14940776
cg19040266	SLC22A5	5	0.00688172	0.44725281	0.39631834	0.29501912
cg16146033	SLC22A8	11	0.00820507	0.28329592	0.31565696	0.18300128
cg19811934	SLC24A4;S	14	0.00126059	0.62582287	0.6095859	0.38565627
cg11107966	SLC24A4;S	14	0.00755255	0.43316034	0.43684691	0.25165271
cg23558213	SLC25A17	22	0.00855208	0.36613283	0.34454674	0.22018241
cg11088051	SLC25A3;S	12	0.0214306	0.44004646	0.50127727	0.33901673
cg07659624	SLC25A37	8	0.00065949	0.78280352	0.68366521	0.55077667
cg14646244	SLC26A4;I	7	0.00233544	0.8229487	0.72276333	0.59364883
cg05028948	SLC27A2;S	15	0.00320053	0.79735233	0.80633666	0.66838759
cg17217665	SLC29A1;S	6	0.00175545	0.42170667	0.50494085	0.26151216
cg25602242	SLC2A5	1	0.00898993	0.7122506	0.7227426	0.57503833
cg13410614	SLC2A6;SI	9	0.04024645	0.82752148	0.87751852	0.70915295
cg25788793	SLC2A9	4	0.00912728	0.36601757	0.38411951	0.24354358
cg03048902	SLC35D3	6	0.01768314	0.69437859	0.67156339	0.54445314
cg27391816	SLC35E2	1	0.02393802	0.61684296	0.60098315	0.4395543
cg25067702	SLC39A11	17	0.00805596	0.37351554	0.38461918	0.243144
cg11761483	SLC39A11	17	0.04013158	0.57740106	0.63931915	0.46567616
cg04204452	SLC43A2	17	0.01891899	0.69050649	0.76451085	0.52860385
cg06121226	SLC4A4;SI	4	0.00209789	0.59237443	0.5971757	0.35781396
cg01629545	SLC6A18	5	0.02096691	0.65971358	0.66727532	0.55065501
cg07010687	SLC6A19	5	0.00218837	0.75152994	0.71179095	0.61170355
cg24756227	SLC6A3	5	0.00279686	0.33560559	0.35274916	0.21003075
cg15155209	SLC6A6;SI	3	0.00073265	0.61181284	0.46226054	0.30710111
cg10586672	SLC6A6;SI	3	0.00100759	0.79087957	0.72465616	0.58500384
cg15454698	SLC6A6;SI	3	0.00315265	0.57768802	0.56327817	0.32091683
cg03987842	SLC7A6;SI	16	0.00092849	0.75938835	0.58707707	0.46462654
cg19015847	SLC8A1;SI	2	0.0012504	0.840105	0.77528543	0.64070182
cg08780166	SLC8A1;SI	2	0.00065949	0.52016267	0.40716976	0.30426794
cg13408795	SLC9A9	3	0.00438997	0.72849726	0.6846996	0.50037244
cg22260869	SLCO3A1;	15	0.0067125	0.68099026	0.7678637	0.54418408
cg02107844	SLCO3A1;	15	0.00379918	0.50715534	0.50726361	0.26472884
cg27132471	SLCO3A1;	15	0.00073547	0.2921373	0.21099356	0.09120508

cg23480341	SLPI	20	0.00317466	0.29261604	0.2412063	0.12273999
cg12966875	SLPI	20	0.00211366	0.58366214	0.57308537	0.30977275
cg25594736	SMAD3	15	0.00822746	0.59034315	0.60396935	0.43469672
cg10017293	SMAGP;D	12	0.01890283	0.66334342	0.67030203	0.51251788
cg26061357	SMARCD3	7	0.00606409	0.73406361	0.72709396	0.53422196
cg13783238	SMC4;MII	3	0.04676265	0.65568382	0.62315863	0.52024576
cg02858512	SMG6;SM	17	0.00210972	0.70738625	0.69280451	0.48094536
cg15816464	SMG6;SM	17	0.02521286	0.57099593	0.58169009	0.45722862
cg07058377	SMG6;SM	17	0.00931986	0.44221044	0.43712146	0.28802174
cg23403980	SMG6;SM	17	0.00755255	0.53766572	0.53827183	0.33302578
cg26927232	SMG6;SM	17	0.00113577	0.89568892	0.85527351	0.74461287
cg05509228	SMOC2;SI	6	0.0284419	0.46795515	0.64627985	0.26615799
cg25025181	SMYD3;SI	1	0.01137235	0.83340137	0.86010824	0.70016201
cg01966878	SNCA;SNC	4	0.0286529	0.53478032	0.58901995	0.41660769
cg11308170	SNORA1;S	11	0.02146686	0.68777601	0.72710272	0.56412975
cg16490124	SNORA14	1	0.00986941	0.15555893	0.21989254	0.03831878
cg04195863	SNRPN;SN	15	0.02541872	0.76536647	0.74519681	0.64259386
cg22208536	SNX25	4	0.00336153	0.51285116	0.44142695	0.27928617
cg20465954	SOAT2	12	0.01113996	0.49834552	0.54598583	0.38243546
cg27312979	SORBS1;S	10	0.00280849	0.81667838	0.81611149	0.67337541
cg20365580	SORBS3	8	0.00152405	0.71200715	0.71429928	0.60897285
cg22357679	SORCS2	4	0.01580869	0.4827201	0.50729918	0.37516514
cg05573434	SORCS2	4	0.03157474	0.64059059	0.65106062	0.52092379
cg00387964	SORCS2	4	0.00588728	0.79457956	0.78200532	0.54785231
cg14239983	SORL1	11	0.00090291	0.37152759	0.30951198	0.19817909
cg05075579	SORL1	11	0.00097337	0.77644257	0.74368813	0.5379294
cg17510957	SORL1	11	0.00216103	0.78522987	0.73258811	0.54160087
cg11287987	SOST	17	0.00131453	0.36906848	0.33636432	0.22118029
cg10825530	SOX6;SOX	11	0.0080564	0.79203428	0.78006859	0.64185614
cg11606261	SP1;SP1	12	0.00323074	0.63780847	0.5921547	0.38335041
cg24044052	SP140L	2	0.01317683	0.43134902	0.44524431	0.3191189
cg25095994	SP140L	2	0.00392454	0.4006901	0.4397168	0.24472706
cg13278004	SP140L	2	0.00330705	0.26055929	0.24193387	0.12297408
cg08974244	SP6	17	0.0118868	0.42715797	0.46625941	0.27278164
cg05730975	SPAG16	2	0.00074016	0.62123027	0.54113224	0.37862936
cg16760223	SPATA16	3	0.01891899	0.6273852	0.59757116	0.45131702
cg14669524	SPATS1	6	0.03571113	0.5441202	0.51514315	0.383042
cg14774117	SPATS1	6	0.00931986	0.52877075	0.5114148	0.28187058
cg06712013	SPATS2	12	0.0122594	0.65897137	0.71409148	0.50751485
cg03394909	SPEG	2	0.00065949	0.67442069	0.57008234	0.42212923
cg23101680	SPERT	13	0.00705117	0.62850467	0.61801778	0.43261154
cg20734569	SPINK8	3	0.01206219	0.34691493	0.38645825	0.23649134
cg07856138	SPN;SPN	16	0.04243997	0.5444355	0.55395893	0.44139342
cg23083984	SPN;SPN	16	0.03630626	0.47306372	0.49233597	0.36458471
cg06101324	SPRR1A	1	0.00181386	0.39089596	0.43557287	0.24406838
cg00891995	SPRR2C	1	0.01631013	0.49246852	0.47525896	0.3449643

cg02713162	SPRY4;SPI	5	0.00540086	0.5973982	0.64783736	0.40055966
cg07200877	SPRYD5	11	0.00112679	0.42292055	0.3945553	0.28932505
cg11622008	SPSB4	3	0.02817065	0.61013688	0.61961811	0.43820334
cg22730004	SPTA1	1	0.02435054	0.63975587	0.68636285	0.50237271
cg24544082	SPTBN1	2	0.00853827	0.83884067	0.83363864	0.70804181
cg01626885	SQRDL	15	0.00958999	0.56456559	0.56615422	0.4142074
cg19701531	SRCRB4D;	7	0.00186737	0.4262497	0.38297388	0.27874789
cg27407935	SREBF1;SI	17	0.01318677	0.37478196	0.33948709	0.23570282
cg16928487	SREBF1;SI	17	0.01820811	0.45671785	0.4491205	0.32819806
cg01668352	SRGAP1	12	0.00094119	0.88198287	0.85488851	0.68050651
cg05733104	SRGAP3;S	3	0.00917702	0.83448857	0.83909997	0.67251838
cg09895325	SRRM1	1	0.01666517	0.44238874	0.49617796	0.34160295
cg19963589	SSBP3;SSI	1	0.02586964	0.6546404	0.71069516	0.4574465
cg01996567	SSPO	7	0.01771787	0.45351912	0.45660243	0.3136166
cg24119990	SST	3	0.02078916	0.68258209	0.67012336	0.56216917
cg09586924	ST14	11	0.00154265	0.42789364	0.39097547	0.18578017
cg22110158	ST14	11	0.00065949	0.7870969	0.67644879	0.47450805
cg24969820	ST3GAL6	3	0.00964959	0.51766704	0.53796666	0.37503163
cg19019345	ST5	11	0.00670817	0.74056855	0.72515034	0.61682012
cg08726522	ST5;ST5;S	11	0.0020588	0.35218032	0.29421787	0.15089064
cg23195547	ST5;ST5;S	11	0.00140062	0.70177241	0.62970406	0.50614729
cg26550194	ST6GALN/	17	0.01580869	0.6288803	0.63122142	0.51641135
cg07168526	ST7;ST7	7	0.00429569	0.80816634	0.83416842	0.63707594
cg14871870	ST8SIA1	12	0.00798815	0.67865438	0.70311234	0.52262088
cg02369052	ST8SIA2	15	0.0048909	0.74378549	0.74040457	0.64017577
cg24112733	STARD3N	7	0.00486472	0.42153391	0.4355557	0.22342346
cg05487134	STAT3;ST/	17	0.04937176	0.80106052	0.74980837	0.63342387
cg24718015	STAT3;ST/	17	0.00339981	0.65045574	0.64481548	0.38963256
cg17833746	STAT3;ST/	17	0.00280852	0.87825912	0.86093109	0.73550285
cg16777510	STAT5A	17	0.0012838	0.46491927	0.4642974	0.25647412
cg25440680	STBD1	4	0.01220473	0.49161619	0.46949226	0.32399809
cg07719679	STEAP4	7	0.00073265	0.2174215	0.1910768	0.09048181
cg14143752	STIM1	11	0.00551198	0.80330995	0.81150586	0.64697848
cg14462124	STK17B	2	0.00855749	0.78045642	0.83320154	0.58107513
cg07804470	STK24	13	0.01809124	0.86122357	0.8668913	0.7000018
cg02767093	STK24;STI	13	0.02974791	0.47917385	0.43994989	0.30828692
cg07496457	STOX2	4	0.00174783	0.83470348	0.80044882	0.7002264
cg12253931	STX18	4	0.00290723	0.78767799	0.72951067	0.61849933
cg01712428	STX1A;ST	7	0.00099274	0.49077751	0.39323922	0.22744375
cg22472023	SULT2A1;	19	0.00131453	0.36857327	0.32500617	0.22154944
cg10262032	SULT2B1;	19	0.00539451	0.49594265	0.4865406	0.36642344
cg01946401	SUPT3H;S	6	0.00289141	0.79707642	0.77725057	0.64620319
cg03940484	SV2B;SV2	15	0.00296278	0.35831163	0.39675969	0.21597547
cg25661049	SV2C	5	0.00812456	0.56847981	0.62262742	0.43016112
cg00593008	SV2C	5	0.01469567	0.61654449	0.60442423	0.45929918
cg05814312	SVIL;SVIL	10	0.00822746	0.40471135	0.42174781	0.25383317

cg06537575	SVOPL;SV	7	0.01948191	0.72043239	0.70605827	0.59109352
cg09976669	SWAP70	11	0.02763355	0.56620182	0.52809301	0.40408859
cg03310376	SYN2	3	0.03710592	0.44172831	0.47157923	0.29364072
cg20476019	SYN3;SYN	22	0.00309052	0.7070024	0.69534389	0.44945322
cg05342835	SYNC;SYN	1	0.01564242	0.69430894	0.70785564	0.54435028
cg07586863	SYNE1;SY	6	0.03369957	0.69563423	0.68196932	0.55442402
cg14079545	SYNGAP1	6	0.036552	0.6380888	0.67731282	0.4920433
cg03467087	SYNJ2	6	0.03129757	0.69078558	0.68284127	0.57770355
cg12002139	SYNJ2	6	0.00091059	0.86358144	0.78272595	0.53122493
cg11569478	SYNPO2;S	4	0.00070305	0.75051996	0.66087632	0.55606157
cg20515580	SYT12	11	0.00352201	0.38334225	0.35310515	0.20761998
cg15957341	SYT9	11	0.00554206	0.42240979	0.46481877	0.2843125
cg21672572	SYT9	11	0.02630407	0.40051714	0.43503613	0.27582006
cg15731317	SYTL2;SYT	11	0.01391836	0.46928774	0.41371129	0.30116904
cg11065271	SYTL3	6	0.00177922	0.74223831	0.69203328	0.52683302
cg12691330	SYTL3	6	0.00568196	0.85201522	0.833449	0.69985022
cg03003434	SYTL3	6	0.00499723	0.8047841	0.83716547	0.58981836
cg00370106	SYTL3;SYT	6	0.04178177	0.4946923	0.50391351	0.33527655
cg10657228	T-SP1	8	0.02377716	0.58223569	0.58275677	0.44627549
cg14284618	TACC1	8	0.00810401	0.29393015	0.30856592	0.16147864
cg15217044	TAGAP;TA	6	0.00458992	0.42525444	0.43078607	0.29082884
cg05262335	TAGAP;TA	6	0.02417619	0.51104856	0.5506413	0.37498926
cg22339338	TAGLN2	1	0.01471045	0.2868606	0.30050818	0.18326772
cg15641364	TAGLN2	1	0.01019308	0.68490841	0.69051672	0.54892306
cg25709789	TAOK1	17	0.00197403	0.7860293	0.77252302	0.66678475
cg17626301	TAP1	6	0.00144751	0.79704617	0.77837206	0.65343785
cg10666909	TAP1	6	0.01714132	0.55061692	0.5649913	0.42859107
cg05592483	TAP2;TAP	6	0.04622903	0.41952599	0.41768581	0.31518804
cg24131538	TAPBPL	12	0.01302012	0.5313464	0.54912806	0.3745299
cg00862408	TBC1D10	11	0.00276502	0.51268604	0.60593631	0.38633736
cg15437231	TBC1D15;	12	0.00065949	0.63719699	0.53611585	0.40977345
cg02348119	TBC1D16	17	0.00743666	0.80515453	0.82537145	0.69163453
cg19016694	TBCD	17	0.002727	0.78141493	0.7124344	0.59153933
cg03930153	TBL1XR1	3	0.03889249	0.30795454	0.30895605	0.18485581
cg04254487	TBPL1	6	0.0120439	0.73564837	0.7510382	0.54165224
cg14557487	TBR1	2	0.00631385	0.40564579	0.48460496	0.29601076
cg04679902	TBX1;TBX	22	0.02729523	0.54601312	0.62059933	0.43022446
cg11631427	TBX2	17	0.01123326	0.76026378	0.75988619	0.5175277
cg03753391	TBX2	17	0.04296377	0.63629608	0.62075454	0.44755089
cg01913455	TBX2	17	0.02054758	0.400958	0.42433463	0.28135768
cg10308654	TBX2	17	0.00670817	0.65887072	0.69275133	0.36788353
cg04794141	TC2N	14	0.0230167	0.29982052	0.32513324	0.18540184
cg03683087	TCF7L2;TC	10	0.00935896	0.32663543	0.25066623	0.13941124
cg05402891	TCL1B;TC	14	0.01485881	0.46606844	0.46259745	0.3370222
cg03852551	TCP10L2	6	0.00541343	0.33255055	0.29317881	0.18950015
cg23346134	TCTA	3	0.00346969	0.3615191	0.35833887	0.20193013

cg09861057	TDRD10;T	1	0.00080601	0.56902385	0.46101529	0.3276698
cg02097120	TDRD10;T	1	0.00065949	0.32411244	0.25199214	0.13840913
cg21198586	TEAD1	11	0.00065949	0.51496178	0.40382865	0.28321464
cg12399536	TEAD1	11	0.04407901	0.56610757	0.56733266	0.44977359
cg17249224	TERT;TERT	5	0.0249821	0.7124919	0.67511563	0.56914906
cg06768361	TESC;TESC	12	0.00427027	0.816233	0.8157478	0.69918622
cg00804338	TFDP1;TF	13	0.00197986	0.20361828	0.20443832	0.08286429
cg08053935	TFEB;TFEI	6	0.01438148	0.46993611	0.45463202	0.30986601
cg15339605	TFEC;TFE(	7	0.00102765	0.47791503	0.46239408	0.16206472
cg11947187	TG	8	0.00225183	0.62611431	0.52406953	0.37159785
cg11883141	TG;SLA;SL	8	0.00241892	0.70093958	0.63611271	0.47268444
cg08911152	TG;SLA;SL	8	0.02614058	0.35054744	0.38893459	0.24478381
cg15735736	TGFB1	19	0.00211122	0.42149837	0.33992122	0.21707099
cg09417692	TGFBR2;T	3	0.01284723	0.6227593	0.62213058	0.38243734
cg02061431	TGFBR3	1	0.01390012	0.4998714	0.51832205	0.34080249
cg00857921	TGFBR3	1	0.00499412	0.60870123	0.55781115	0.41592441
cg25769732	TGFBR3	1	0.01064552	0.38593874	0.3376501	0.23151229
cg04204669	TH;TH;TH	11	0.00590146	0.70480783	0.70477876	0.59472439
cg08685909	TH1L	20	0.00252483	0.43551464	0.42153892	0.30103142
cg23105419	THADA;TH	2	0.00075254	0.89022167	0.82096495	0.5837116
cg13869942	THEM4	1	0.02995719	0.55141653	0.56508934	0.43911079
cg11344950	THEM5;TI	1	0.00622238	0.72543866	0.75486378	0.62319022
cg19763108	THRB;THF	3	0.0033048	0.499674	0.41213781	0.30686252
cg00466492	TIMM44;(	19	0.00183809	0.62983268	0.58197351	0.45949237
cg14255824	TJP2;TJP2	9	0.00105674	0.80454355	0.76846247	0.62219186
cg02934719	TK2	16	0.00155292	0.8355086	0.77076397	0.63727886
cg10472651	TK2	16	0.00523248	0.6073392	0.58645383	0.44057554
cg13544946	TLN1	9	0.02935375	0.43220598	0.38777683	0.27874582
cg25840354	TLR10;TLI	4	0.00912728	0.39247369	0.38869417	0.22017821
cg23039250	TLR5	1	0.01771787	0.4857475	0.47975921	0.33766265
cg01883662	TM4SF19	3	0.00429569	0.69881893	0.70859956	0.57930565
cg21845080	TM4SF19	3	0.00704609	0.5881633	0.58338188	0.47261598
cg21090033	TM4SF19	3	0.00152405	0.78087641	0.78009496	0.61948916
cg15044270	TM4SF19	3	0.0037342	0.38632901	0.38711957	0.22686818
cg13314965	TM4SF19	3	0.0013522	0.79795618	0.7863301	0.60124832
cg23250593	TM4SF19	3	0.00885213	0.61389036	0.6072979	0.50205853
cg02641539	TM7SF4	8	0.00958999	0.35550192	0.33366656	0.21670978
cg03190661	TMC8;TM	17	0.02700247	0.67844337	0.63923619	0.4905552
cg19056418	TMC8;TM	17	0.036552	0.60268975	0.59444491	0.47767824
cg06507678	TMED7-TI	5	0.00598432	0.77417964	0.70931461	0.59884102
cg05384697	TMEM13.	12	0.02072204	0.81086021	0.76794336	0.66362725
cg21363348	TMEM14(	7	0.00119385	0.26379601	0.22366971	0.10869893
cg09414827	TMEM14(	7	0.00160451	0.28097769	0.26095952	0.1103835
cg27641628	TMEM14(	7	0.002727	0.46355135	0.44274448	0.22751652
cg08016257	TMEM14(	7	0.00944446	0.66746675	0.7019125	0.50056343
cg07522970	TMEM14(	7	0.00670577	0.55705427	0.57170611	0.34372667

cg06456031	TMEM14(	7	0.00317432	0.50063093	0.49644655	0.28011951
cg07972322	TMEM14(	7	0.00334886	0.37910192	0.33892227	0.18167848
cg15139596	TMEM14!	19	0.00600204	0.58506786	0.54115246	0.41437341
cg16911583	TMEM14!	19	0.04648826	0.71368728	0.73905058	0.59783811
cg04232128	TMEM17:	5	0.00129758	0.39805332	0.32289531	0.19676638
cg18396403	TMEM18	22	0.02346046	0.65615656	0.70160684	0.55476312
cg25921357	TMEM19(	19	0.03524554	0.43510872	0.43585613	0.32654956
cg26906737	TMEM20(	1	0.00806542	0.69427623	0.6939745	0.58199645
cg06776976	TMEM20(	1	0.02883885	0.54959626	0.59914175	0.44330105
cg20068209	TMEM30/	6	0.00950809	0.43512432	0.5043052	0.30153465
cg18282388	TMEM45I	11	0.01580766	0.68397008	0.66538735	0.52400666
cg24174557	TMEM49	17	0.02987993	0.647217	0.65775384	0.50023972
cg01409343	TMEM49	17	0.00115895	0.60435646	0.50083771	0.37935156
cg02782634	TMEM49	17	0.00160033	0.54607985	0.45154007	0.31859975
cg19897979	TMEM50/	1	0.00066204	0.87336072	0.78222552	0.64560368
cg23313885	TMEM71;	8	0.00263672	0.47543339	0.40627091	0.3002369
cg18973101	TMEM79;	1	0.00375204	0.29893833	0.27592152	0.17023964
cg05954120	TMEM79;	1	0.01559842	0.61809121	0.59503024	0.46820392
cg14087413	TMEM8B;	9	0.01203452	0.45916811	0.4197203	0.30972833
cg13736811	TMEM91	19	0.02241786	0.71066898	0.74309096	0.52881151
cg12955084	TMEM91;	19	0.00447146	0.78480866	0.76305718	0.65598918
cg00488740	TMIGD2;T	19	0.00670817	0.7989869	0.80205061	0.68651491
cg27144670	TMOD3	15	0.00322924	0.77306567	0.79076612	0.60498696
cg10973881	TMOD4	1	0.02595684	0.41601001	0.4467884	0.30063731
cg17318719	TMPRSS4	11	0.00368022	0.50816558	0.45056683	0.28444118
cg21370522	TNF	6	0.02099588	0.58644753	0.64447656	0.46644174
cg03037030	TNF	6	0.01078115	0.44363764	0.45566858	0.27477745
cg01569083	TNF	6	0.03060322	0.55579862	0.58213908	0.45848254
cg09918674	TNFAIP8L	19	0.03756327	0.36956301	0.38077352	0.26339905
cg02368508	TNFRSF17	16	0.0048797	0.75359403	0.75126033	0.63732333
cg20820107	TNFRSF8;	1	0.00244246	0.68421816	0.71033865	0.4447524
cg09247175	TNFRSF8;	1	0.00586773	0.51426213	0.50083461	0.3497939
cg14153654	TNFRSF9	1	0.00327239	0.5395548	0.46343874	0.3266607
cg18025409	TNFRSF9	1	0.00930715	0.42927346	0.36347854	0.24832433
cg01462856	TNFSF13E	13	0.00838913	0.35483215	0.40273513	0.20941061
cg10362335	TNFSF14;	19	0.00868726	0.59154919	0.64762268	0.45266603
cg26315984	TNFSF4	1	0.02995719	0.62969343	0.69371341	0.50087831
cg10861599	TNFSF4	1	0.0052016	0.64173411	0.66828428	0.48415227
cg05687083	TNRC18	7	0.00379918	0.83039846	0.87290957	0.63289892
cg02481714	TNRC6B	22	0.00551242	0.83128542	0.79917991	0.66378182
cg12338137	TNS1	2	0.00165724	0.84409045	0.77902416	0.59674269
cg01188191	TNXB	6	0.01158752	0.74126934	0.75749191	0.60394261
cg03376089	TOM1L2;	17	0.00065949	0.84803499	0.7793225	0.55719587
cg13473434	TOMM40	1	0.00134295	0.27472382	0.24503635	0.13435851
cg21388339	TP73;TP7	1	0.02774083	0.41949457	0.50063958	0.24983985
cg12205230	TPM3	1	0.01334584	0.53612693	0.59506556	0.42992956

cg09856467	TPST2;TPS	22	0.00749271	0.69041461	0.68231378	0.4453973
cg16704530	TRAF3IP2	6	0.00073265	0.83996799	0.76232806	0.6368191
cg11756029	TRAFD1;T	12	0.0065693	0.56211143	0.45191965	0.33656338
cg22211507	TRAT1	3	0.00694471	0.64279935	0.66613397	0.52303806
cg21328082	TREM1	6	0.00128725	0.62673138	0.58246141	0.31363494
cg10981439	TREM1;TF	6	0.0015244	0.70864534	0.69730005	0.40111094
cg25473438	TREML2	6	0.00855749	0.52914418	0.55346413	0.42896179
cg05264377	TRERF1	6	0.0007088	0.61190946	0.52727376	0.40764241
cg09103187	TRERF1	6	0.00073118	0.57782494	0.40456497	0.27087251
cg16597079	TRERF1	6	0.00628266	0.61654312	0.62617733	0.50001159
cg09804858	TRERF1	6	0.00796638	0.79561567	0.77710363	0.6256895
cg15120497	TRH	3	0.01884096	0.74481183	0.71676497	0.61237237
cg14741870	TRIM26	6	0.00078729	0.7913114	0.69501835	0.53453756
cg01505556	TRIM26	6	0.00066232	0.56232059	0.43494566	0.28981879
cg14606478	TRIM26	6	0.00112679	0.58213873	0.51024331	0.36858405
cg01591152	TRIM29	11	0.00069165	0.61883006	0.50055362	0.35883777
cg17048834	TRIM31	6	0.00186404	0.55700039	0.62793644	0.45421706
cg01190484	TRIM40;T	6	0.03956605	0.45670616	0.46889816	0.31540948
cg06365108	TRIM40;T	6	0.00514311	0.54324711	0.6050867	0.42110769
cg26700215	TRIM55;T	8	0.00403008	0.50590069	0.54654721	0.32259937
cg22107533	TRIM69;T	15	0.00828598	0.66006462	0.67733069	0.53348945
cg19200592	TRIM71	3	0.00805241	0.53899684	0.58049056	0.39611663
cg21531038	TRIO	5	0.00159601	0.79640092	0.77758878	0.62116436
cg04646987	TRIO	5	0.01531619	0.83019376	0.79057589	0.68794482
cg11421768	TRIP13	5	0.00070134	0.85759299	0.79652324	0.66552862
cg17510385	TRIP13	5	0.00097509	0.84138073	0.76964031	0.65656601
cg17206393	TRPC4AP;	20	0.00629918	0.63060547	0.65913934	0.42310297
cg17406359	TRPM2	21	0.03749859	0.59981789	0.6161993	0.4942459
cg08191854	TRPM2	21	0.03960534	0.60637818	0.62204552	0.50501964
cg26719625	TRPV2	17	0.00092387	0.82549481	0.79435924	0.59668989
cg09093656	TRPV2	17	0.00165724	0.74476011	0.69208902	0.45971677
cg10122294	TSNAX-DI	1	0.00093001	0.39132522	0.34650597	0.23832866
cg04250837	TSNAX-DI	1	0.01820811	0.69555269	0.70472764	0.58602043
cg05946353	TSPAN10	17	0.00073118	0.77529766	0.72688219	0.57966482
cg09690632	TSPAN10	17	0.0009934	0.82330709	0.80007663	0.6877979
cg00509207	TSPAN11	12	0.01561358	0.71738304	0.69202524	0.5878175
cg01481646	TSPAN14;	10	0.01268304	0.79115001	0.79950697	0.67157306
cg08560387	TSPAN14;	10	0.03452515	0.76658777	0.81076913	0.64302874
cg24328142	TSPAN15	10	0.00066232	0.83299395	0.77604752	0.58260973
cg18247223	TSPAN18	11	0.00065949	0.78993237	0.6498869	0.5416388
cg11958128	TSPAN9;T	12	0.01980843	0.65484893	0.73242034	0.47786657
cg15852320	TSPAN9;T	12	0.01476513	0.71222831	0.73315191	0.54113749
cg14340336	TTC25	17	0.01044398	0.46381934	0.4946332	0.32658976
cg14766231	TTC32	2	0.00938113	0.73733238	0.74173046	0.57035454
cg19319037	TTF2	1	0.00970413	0.57750307	0.53553308	0.39292314
cg18788205	TTPAL;TTI	20	0.03060322	0.75946726	0.76452516	0.64449891

cg26282283	TTYH3	7	0.02548815	0.47107578	0.42562631	0.30793998
cg19050555	TUBA1C	12	0.00362629	0.84550544	0.84216413	0.6417141
cg01108243	TUBB1	20	0.01980843	0.64631995	0.66332944	0.54401648
cg13756273	TUBB1;TU	20	0.00065949	0.87490254	0.79134357	0.64070578
cg19244428	TUBB2B	6	0.0048909	0.58810645	0.58232304	0.40936729
cg02113368	TUBGCP2	10	0.00132175	0.91737953	0.88687798	0.77486003
cg01280180	TXNDC6	3	0.01438148	0.72205739	0.69147455	0.56116629
cg14557690	UAP1L1	9	0.00186737	0.68732717	0.62291745	0.48128485
cg07450210	UBAC2;UI	13	0.00708587	0.71435805	0.73647788	0.47515991
cg05257202	UBAC2;UI	13	0.00686574	0.649849	0.63033753	0.39771795
cg12578486	UBAC2;UI	13	0.00693355	0.66497415	0.64644138	0.39205801
cg24058145	UBD	6	0.01010631	0.79307342	0.82393816	0.69086788
cg11554335	UBE2L6	11	0.00065949	0.55049641	0.44358895	0.26031186
cg27429749	UBE2L6;U	11	0.00561885	0.49611385	0.53306561	0.31102679
cg04346683	UBE2Q2;U	15	0.02252087	0.52757989	0.54438347	0.40493354
cg17295666	UBOX5;FA	20	0.02634735	0.45343985	0.45123293	0.35025815
cg24247482	UBTF;UBT	17	0.03437206	0.60771383	0.59406283	0.4751521
cg10135708	UCN2	3	0.0235449	0.73204319	0.72530992	0.59960935
cg14699903	UCN2	3	0.0019194	0.69693659	0.68751872	0.5681963
cg22145666	UGT1A10	2	0.00799281	0.60773906	0.64604954	0.49002454
cg06635797	UHRF1;UI	19	0.00186784	0.35584901	0.32230425	0.17914857
cg20724032	ULK4	3	0.00151202	0.49597255	0.40036388	0.2775946
cg02527375	ULK4	3	0.00074036	0.89902362	0.84099228	0.7190831
cg16413715	UNC5A	5	0.00938113	0.63147734	0.64059329	0.51235638
cg20434422	UOX	1	0.00084118	0.84763846	0.80893676	0.64956922
cg04585822	USH1G	17	0.04667899	0.72183569	0.77187369	0.61439457
cg23982392	UST	6	0.00170239	0.7736754	0.70838669	0.59939072
cg09594210	UST	6	0.02658729	0.28633835	0.28230148	0.18070836
cg11274172	UVRAG	11	0.00247655	0.78873751	0.78581561	0.54779327
cg17567602	UXS1	2	0.02156272	0.79471424	0.75318218	0.63111798
cg06710195	VAV3	1	0.00092849	0.6243389	0.58799396	0.42213955
cg25763716	VCAM1;V	1	0.00275808	0.52982633	0.56192472	0.31531094
cg04295372	VCAM1;V	1	0.00552473	0.6781523	0.70675044	0.51691656
cg14260889	VIM	10	0.00143276	0.83910477	0.80619641	0.6775806
cg12595461	VIT	2	0.01158752	0.5017437	0.47797814	0.30420783
cg00852964	VNN1	6	0.03696736	0.49209597	0.55388558	0.37827816
cg23654401	VOPP1	7	0.00177922	0.67937962	0.64042781	0.40290375
cg14844130	VPREB1	22	0.01273892	0.37163155	0.39613847	0.22783988
cg03763796	VPREB1	22	0.00383289	0.59274919	0.60009622	0.43981868
cg25330366	VPREB1	22	0.00277567	0.34687652	0.35729096	0.19649333
cg18441959	VPREB1	22	0.00429038	0.29849869	0.29950878	0.19634324
cg21689902	VWA5A;V	11	0.00183809	0.55861959	0.50856943	0.29087519
cg25884442	VWF	12	0.00093631	0.50431226	0.46233769	0.31231066
cg18898325	WBP2NL	22	0.00720289	0.78700328	0.76390267	0.63279504
cg00187692	WDFY2	13	0.0019386	0.6113951	0.53070018	0.37479503
cg20469502	WDR25;W	14	0.00065949	0.90745825	0.82928931	0.72055262

cg09755594	WDR63	1	0.02195382	0.50309595	0.54114987	0.3766673
cg22822803	WDR8	1	0.04388834	0.53554831	0.50826255	0.39752144
cg09433910	WDR81;W	17	0.00092661	0.54261686	0.40855839	0.23265122
cg10080732	WDR81;W	17	0.00104158	0.37446991	0.31517956	0.1680857
cg11783356	WFDC10E	20	0.04901873	0.61496268	0.61487838	0.51438443
cg20485165	WFDC12	20	0.00132756	0.64908783	0.65783054	0.54837293
cg07982740	WFDC3	20	0.00279442	0.74710069	0.69609802	0.5820904
cg15558558	WHAMM	15	0.00201167	0.47580101	0.42298507	0.28173283
cg15058645	WIPF1	2	0.00967401	0.57749622	0.59361161	0.37457796
cg19275653	WIPF1	2	0.00519569	0.53382733	0.55670448	0.36883948
cg17165848	WIPF1;WI	2	0.00478782	0.45783071	0.34352708	0.23834357
cg27117828	WIPF1;WI	2	0.00575178	0.62865028	0.63061813	0.41914975
cg03983223	WIPF1;WI	2	0.01160447	0.53585028	0.55294523	0.37032263
cg03562120	WISP2;WI	20	0.00177039	0.76562631	0.69687291	0.59143328
cg04154465	WNT2B	1	0.02122541	0.61777776	0.65370589	0.51662535
cg15237742	WNT3	17	0.00701189	0.42352098	0.43287588	0.28552086
cg24114556	WNT3	17	0.00429038	0.42170681	0.48172647	0.3141834
cg26121234	WNT7A	3	0.00094876	0.37859965	0.34052604	0.23127273
cg02179982	WNT7B	22	0.00283173	0.76413297	0.69769915	0.52219635
cg07777652	WRB	21	0.0113406	0.66369454	0.64386929	0.43756378
cg24115019	WWC1;W	5	0.00107365	0.52491058	0.4968166	0.35598904
cg18484958	WWTR1;\	3	0.00065949	0.78612313	0.6956307	0.53763693
cg10353683	XCR1;XCR	3	0.00539186	0.62392785	0.58292865	0.41215007
cg24394891	XPNPEP1;	10	0.0140881	0.72847625	0.70802406	0.56416332
cg11845168	XPO4	13	0.0040421	0.6145897	0.61473252	0.37632387
cg26730763	XPO6	16	0.00471011	0.47103032	0.52546361	0.35874395
cg02889774	XPO7;XPC	8	0.04390149	0.72667242	0.73601409	0.58288098
cg07140459	XRN1;XRN	3	0.00264473	0.51481089	0.47107177	0.30672194
cg15389191	XRRA1	11	0.00600779	0.78018828	0.76841602	0.64947733
cg09745987	XYLT1	16	0.00228672	0.36745443	0.37293779	0.20957832
cg08908247	YIF1A	11	0.01067425	0.57762786	0.61772842	0.46499896
cg24040043	YOD1	1	0.04006803	0.57248101	0.53515174	0.42044496
cg19743666	YPEL5;YPI	2	0.00703549	0.61849022	0.62885778	0.47708857
cg00004667	ZBTB17	1	0.01653772	0.62957316	0.64108232	0.43318173
cg03559942	ZBTB3	11	0.01198766	0.43234239	0.485468	0.3307733
cg09231418	ZBTB32	19	0.04941317	0.52271232	0.48507127	0.38470174
cg03025465	ZBTB38	3	0.00779924	0.79592253	0.80332005	0.6331774
cg19680850	ZC3H12A	1	0.00344865	0.23264255	0.24492854	0.10511729
cg00859858	ZC3H12A	1	0.00139783	0.83616357	0.78821337	0.62063787
cg00073460	ZC3H12D	6	0.00168532	0.38243632	0.31380768	0.18408261
cg06762457	ZC3H12D	6	0.00329066	0.55893773	0.52785101	0.37295179
cg14030904	ZC3H12D	6	0.00522751	0.82910228	0.80193836	0.67641953
cg20995564	ZEB2;ZEB	2	0.00555806	0.91338083	0.90800505	0.80665069
cg23095192	ZEB2;ZEB	2	0.00121234	0.39983863	0.45185717	0.29316723
cg26608667	ZFAND2A	7	0.00575746	0.55264324	0.53637173	0.36017216
cg05972185	ZFHX3;ZFI	16	0.00334886	0.56877749	0.55421877	0.45304638

cg10298741	ZFHX3;ZF	16	0.00068904	0.65744845	0.57796645	0.45993343
cg07176589	ZFHX4;LO	8	0.03878779	0.72639099	0.75744369	0.62529058
cg10174063	ZFP57	6	0.0359373	0.5446455	0.61854822	0.35958729
cg11814087	ZFR	5	0.02049456	0.86957963	0.8862461	0.75397393
cg26306154	ZFYVE20	3	0.00100011	0.89758711	0.8575974	0.75308392
cg04884025	ZFYVE9;ZI	1	0.00065949	0.67919443	0.54122994	0.41704203
cg26224785	ZIC4;ZIC4	3	0.01841074	0.51744149	0.60002604	0.40688187
cg02872693	ZNF133;Z	20	0.00079883	0.8948957	0.82413196	0.71403634
cg10668363	ZNF175	19	0.03038061	0.8404941	0.81304614	0.7128689
cg27474223	ZNF19	16	0.00097618	0.788144	0.75257477	0.56359945
cg01855245	ZNF19	16	0.00186737	0.7934311	0.75744524	0.62424407
cg18507146	ZNF236	18	0.01506509	0.35824232	0.37727899	0.25198857
cg27118035	ZNF267	16	0.00341932	0.30541304	0.25939301	0.14309781
cg14690467	ZNF282	7	0.0008947	0.62517803	0.53806507	0.42256394
cg02278959	ZNF286B;	17	0.0047737	0.75244191	0.72202843	0.58512929
cg15394911	ZNF331;Z	19	0.00882698	0.84649601	0.82703592	0.7180913
cg13733708	ZNF335	20	0.02447745	0.5790189	0.54758024	0.42526153
cg23786747	ZNF365;Z	10	0.04639674	0.45727875	0.46948883	0.31635227
cg03861065	ZNF365;Z	10	0.00126535	0.87539161	0.83725003	0.70824461
cg13823492	ZNF365;Z	10	0.01526804	0.7437883	0.79165349	0.62882016
cg09287086	ZNF365;Z	10	0.00806542	0.845203	0.83875454	0.73818226
cg21330896	ZNF395	8	0.00206218	0.27482299	0.27371796	0.16926608
cg07787851	ZNF474	5	0.00081925	0.90144184	0.86361796	0.76225881
cg21213593	ZNF608	5	0.00930663	0.7960168	0.78410319	0.58852184
cg16558770	ZNF710	15	0.0091896	0.78466584	0.83702755	0.6389602
cg09939948	ZNF710	15	0.00337229	0.30886627	0.31226608	0.15869221
cg02443306		11	0.03170823	0.42598012	0.38285436	0.25877884
cg10308673		11	0.03173228	0.46033992	0.4708791	0.34562525
cg20312077		8	0.00073265	0.68028043	0.57449652	0.44104887
cg20223392		8	0.00152023	0.77809051	0.68915606	0.57264062
cg10717610		2	0.04382199	0.59588737	0.61098507	0.49433477
cg05464506		2	0.01145563	0.62851283	0.58408518	0.42709435
cg02924234		2	0.00190848	0.76555856	0.68393534	0.50759631
cg11539857		6	0.00065949	0.88047015	0.78185095	0.66620136
cg08800670		6	0.00338706	0.71958688	0.67728119	0.5382111
cg12686441		8	0.00178025	0.55724292	0.56036148	0.26641234
cg09533087		8	0.00451843	0.83668935	0.8313462	0.7279536
cg09757158		2	0.00220808	0.70444162	0.71283121	0.57274476
cg19485103		6	0.00559757	0.42946757	0.50591856	0.30455213
cg10454879		8	0.04357378	0.78626855	0.77266418	0.65904013
cg01119145		20	0.0013706	0.63093187	0.60069782	0.49717118
cg20435594		11	0.02195328	0.54040498	0.54299039	0.42030288
cg26201596		10	0.0066172	0.70020361	0.69127587	0.56798136
cg07178994		8	0.00338778	0.60967728	0.63900442	0.46352337
cg06623778		1	0.01760238	0.56729551	0.58387739	0.42384477
cg15415194		8	0.00294138	0.70395258	0.68407085	0.57536869

cg08212438	16	0.0050226	0.82215369	0.77779939	0.67082352
cg00687073	8	0.02075001	0.59580811	0.55977362	0.45354713
cg08945340	16	0.01862437	0.65453468	0.60020033	0.49990848
cg07191524	16	0.00480767	0.74316743	0.7030021	0.56155991
cg17224892	17	0.00637687	0.46383273	0.4404995	0.31635046
cg01952913	8	0.01704845	0.50951253	0.63305688	0.40235766
cg08474826	1	0.00105291	0.82444622	0.7557467	0.52537421
cg01577678	16	0.00140274	0.81032586	0.74159892	0.59844523
cg01619416	16	0.00314038	0.68294444	0.59070419	0.47268483
cg01830154	17	0.00563159	0.48404765	0.41116552	0.30049119
cg02296931	1	0.00997964	0.66540222	0.66100451	0.51814566
cg26999501	8	0.02758055	0.86761111	0.88090951	0.75571678
cg11629812	5	0.03533922	0.57751556	0.58591972	0.46029288
cg24699005	19	0.00802316	0.67207034	0.66278436	0.47910295
cg27094323	7	0.0156946	0.46742442	0.46495998	0.35590471
cg12165219	8	0.01080128	0.8095763	0.73495272	0.62812037
cg21146170	8	0.00236052	0.73350548	0.68764283	0.5780817
cg20309975	8	0.00302287	0.65820469	0.59680576	0.4915372
cg00320597	8	0.03302702	0.7379026	0.7710726	0.61795469
cg12246922	7	0.00587243	0.72734303	0.66988373	0.55223875
cg04095327	5	0.021196	0.40211037	0.43558522	0.29506298
cg15579822	6	0.028548	0.73704967	0.73382634	0.61504165
cg16442298	8	0.00242331	0.60306911	0.57997795	0.46637719
cg08525429	7	0.02708404	0.49334176	0.47633626	0.36902514
cg22013019	6	0.00133856	0.7184448	0.6924575	0.51633536
cg11234688	2	0.00920255	0.68319151	0.6017409	0.44571209
cg10397082	2	0.0156653	0.49336088	0.4609572	0.32092954
cg17159058	8	0.00207771	0.29188528	0.24816249	0.11104196
cg03503942	20	0.01485881	0.57458794	0.57524114	0.34866629
cg08692111	19	0.02655172	0.41089744	0.40650506	0.27692748
cg17681527	7	0.00437662	0.34705179	0.41378226	0.20260838
cg13827584	7	0.02414194	0.33214136	0.30865113	0.20274931
cg15684661	5	0.00147442	0.57533045	0.52569252	0.42183355
cg05693204	5	0.00628685	0.61707352	0.60568619	0.48948385
cg21707089	8	0.00097509	0.4887266	0.43647168	0.30868372
cg03660096	8	0.0048909	0.81561707	0.78071699	0.64380211
cg19462210	11	0.02511511	0.71562977	0.75147213	0.58847235
cg18143317	11	0.00729536	0.62542026	0.60100446	0.49931366
cg14553377	11	0.0235449	0.54013225	0.53633481	0.43141625
cg03024791	11	0.00097509	0.51808049	0.48770683	0.35271484
cg09476073	11	0.01423002	0.52182257	0.56475255	0.39480406
cg26986989	5	0.00183809	0.69176087	0.67905363	0.56165825
cg13183298	1	0.00994104	0.69231031	0.65706638	0.55132325
cg00055986	5	0.0154863	0.55977013	0.59155928	0.43212108
cg25794147	7	0.00725151	0.34005816	0.36609732	0.2250106
cg21957306	2	0.03124896	0.67438184	0.66832756	0.53930488

cg07611933	16	0.00128265	0.78935103	0.71302332	0.58108697
cg15030449	1	0.00294202	0.48102589	0.5037364	0.3136555
cg12635314	10	0.02852565	0.7639305	0.81028679	0.65608791
cg21943117	1	0.00407141	0.72447036	0.66142083	0.50828577
cg25720825	1	0.01809124	0.6273956	0.61259588	0.48738453
cg13402298	1	0.00192049	0.8652803	0.88382565	0.70560949
cg04921646	1	0.0017241	0.89307944	0.87916796	0.73243516
cg10943263	6	0.02350362	0.27807347	0.34847963	0.17348968
cg01977798	1	0.00219711	0.86854153	0.85182324	0.70969464
cg00050294	1	0.00122881	0.85788534	0.80276571	0.62451557
cg02589685	11	0.02818766	0.72209354	0.71488484	0.60850331
cg08306076	6	0.01111549	0.78830729	0.76879875	0.6258436
cg01462537	10	0.00380612	0.81182617	0.79525	0.69508558
cg07295362	2	0.00878866	0.63096305	0.68303877	0.47537395
cg24882709	10	0.00093631	0.84550225	0.80639675	0.66177993
cg02621151	5	0.02514684	0.36700447	0.44327794	0.25990971
cg25555753	19	0.00670577	0.58455208	0.60587543	0.37822746
cg16428517	16	0.00065949	0.74344629	0.65471334	0.54804221
cg03247944	12	0.02310662	0.46561321	0.50664013	0.36327199
cg15805490	19	0.01661249	0.69609653	0.67370168	0.56468546
cg16704703	10	0.00099274	0.66055054	0.59218539	0.33355045
cg08698943	10	0.0012278	0.60065075	0.52241607	0.2673149
cg11624328	5	0.01574127	0.65401249	0.67848896	0.52163112
cg24996440	2	0.00180881	0.39111873	0.35319415	0.25042779
cg06637330	5	0.01157637	0.47086133	0.48296571	0.36481155
cg25354587	12	0.00225672	0.40371655	0.37452522	0.23776266
cg13413719	6	0.00961487	0.79187737	0.7711846	0.56965804
cg00520406	6	0.00403008	0.72775692	0.7712094	0.55896418
cg12048965	10	0.00173751	0.82295242	0.79221694	0.58288825
cg11964099	17	0.0036184	0.63087282	0.59687169	0.4427555
cg03076094	16	0.02514684	0.54131506	0.54874775	0.41861205
cg16747928	6	0.00065949	0.70354998	0.56950429	0.45369414
cg05148180	10	0.0120439	0.66549839	0.66230576	0.5167674
cg21995261	11	0.00323713	0.50719087	0.58575129	0.40477025
cg02849956	19	0.00154265	0.4713037	0.364136	0.2151023
cg27661394	4	0.00065949	0.71507564	0.57209086	0.40927397
cg19619817	10	0.03541631	0.80814633	0.81182544	0.67849537
cg01832712	3	0.00362881	0.4541846	0.48517436	0.26799101
ch.4.194519R	4	0.02080485	0.31241411	0.32469461	0.18133851
cg00874032	3	0.00065949	0.54817416	0.43170576	0.3243168
cg00512484	18	0.02013944	0.60955792	0.63448222	0.50131207
cg15613991	7	0.01150507	0.53985705	0.49031676	0.39000971
cg13067635	1	0.00703549	0.70625489	0.74488325	0.56943509
cg19500334	1	0.00931986	0.3519897	0.34462053	0.23918813
cg05586863	16	0.00804549	0.75576584	0.74712041	0.62516349
cg05144620	17	0.00085205	0.49495043	0.45853525	0.32121864

cg10898182	5	0.00184921	0.76025826	0.75607574	0.62383492
cg15222329	5	0.00186737	0.39942611	0.39716795	0.28301603
cg15463580	19	0.00223531	0.6054535	0.56973672	0.45934553
cg19726711	10	0.00101151	0.55874284	0.47483972	0.36480035
cg10601624	12	0.00073378	0.5828343	0.54873672	0.34937983
cg11404945	1	0.00597821	0.6394587	0.59596966	0.48863548
cg07142009	17	0.00503319	0.65377062	0.69307236	0.43239192
cg21922810	1	0.04280685	0.36314681	0.34482493	0.23774066
cg10721538	17	0.0452911	0.48434893	0.5225808	0.36510331
cg08090407	7	0.00246888	0.21977131	0.22826796	0.11486962
cg27594683	6	0.00814151	0.32924791	0.32723971	0.21475536
cg16799831	6	0.00745657	0.42999564	0.42663201	0.28013085
cg03263730	1	0.0338225	0.77375972	0.81538557	0.67337267
cg22229039	1	0.01870855	0.77563902	0.79148542	0.61861346
cg02312715	5	0.00429038	0.84019853	0.82764003	0.71575741
cg26118643	1	0.00091059	0.71521327	0.67307908	0.53293839
cg08375396	11	0.00081925	0.84912424	0.78506376	0.67000643
cg13014219	4	0.03718903	0.36734797	0.3627556	0.25911939
cg06322557	3	0.00753326	0.66656343	0.68743659	0.50018957
cg14366742	4	0.01030631	0.40806229	0.41756946	0.27915927
cg08894532	19	0.01925109	0.56595468	0.5670759	0.4410549
cg08015107	11	0.02004883	0.53301362	0.5649469	0.40386484
cg18100045	3	0.00083228	0.59956827	0.49324904	0.3270282
cg00034336	2	0.00783897	0.64979537	0.64605467	0.47364069
cg15074524	2	0.00733126	0.67997403	0.68986568	0.44329002
cg15981982	2	0.00930663	0.76459558	0.75605313	0.57627539
cg03614721	5	0.00335524	0.45406229	0.44777898	0.27521994
cg00184457	8	0.01574127	0.70083645	0.76252594	0.54078534
cg05405914	16	0.01890283	0.43814501	0.43247736	0.32136645
cg08943045	1	0.01764264	0.58866368	0.54821769	0.40001609
cg10377510	1	0.02360575	0.47730454	0.49685208	0.32540294
cg22373770	1	0.00419259	0.36936302	0.26966303	0.15831908
cg16102739	1	0.04265641	0.61613211	0.57540415	0.44862437
cg13693328	1	0.00230968	0.49274618	0.53290769	0.29321849
cg05933510	1	0.00648185	0.80489674	0.79712838	0.5951371
cg22730655	1	0.00453677	0.52459757	0.52344887	0.37244468
cg01246390	12	0.00486472	0.84285132	0.86170188	0.72898564
cg27511894	1	0.00436875	0.62120927	0.61602578	0.4320176
cg01450566	20	0.00280561	0.66100692	0.65471958	0.51546692
cg11018892	6	0.00127427	0.79401727	0.77078263	0.63714793
cg13468767	17	0.00851187	0.37686628	0.29302083	0.18009515
cg11374933	8	0.00297992	0.47086694	0.47257921	0.33132532
cg03959186	2	0.0111106	0.39401262	0.37575867	0.22039399
cg18320356	12	0.00623289	0.69830718	0.66572956	0.42700918
cg13716880	2	0.00391578	0.74264741	0.73337129	0.6034245
cg07353006	8	0.02389893	0.43864204	0.46037408	0.32813552

cg24134018	18	0.04180327	0.62120517	0.56570768	0.42094941
cg05940672	2	0.01098413	0.57853031	0.65070063	0.4652947
cg06828143	5	0.04649715	0.41268628	0.44404116	0.30109874
cg20052760	6	0.00855749	0.44025917	0.49124049	0.32084433
cg24505713	11	0.01546656	0.35993484	0.35239471	0.24169135
cg10466548	16	0.00291147	0.43361188	0.41747653	0.22350652
cg04224025	1	0.01096356	0.72514862	0.77302331	0.62083712
cg23919830	21	0.01729366	0.57024472	0.60091812	0.46539964
cg01303055	11	0.01193746	0.53803906	0.49319244	0.38469861
cg08493063	16	0.01677529	0.55284013	0.56385833	0.41487272
cg09528494	8	0.00177922	0.58161756	0.54911743	0.34668866
cg04136456	1	0.00290723	0.40803867	0.42366951	0.22158654
cg06946797	16	0.00381415	0.44205812	0.44731613	0.26457696
cg15952840	8	0.01575731	0.56790967	0.55157778	0.44693735
cg01510278	16	0.00318264	0.55405794	0.43314533	0.24802085
cg09232555	8	0.00618952	0.78136258	0.80454713	0.67017709
cg16251016	19	0.0006776	0.63345451	0.56554858	0.43015417
cg03052004	6	0.01465218	0.85180157	0.86276312	0.74549322
cg07593977	8	0.00758031	0.76653754	0.74490103	0.52674847
cg07283896	6	0.00396544	0.53233308	0.57157899	0.28998337
cg26394636	10	0.0470817	0.72413414	0.81539562	0.62128115
cg22877366	1	0.00283769	0.50569397	0.48735999	0.36205374
cg19800032	7	0.03308492	0.50732703	0.50510006	0.39648613
cg04399631	3	0.00065949	0.76661482	0.68052378	0.49237629
cg15930777	6	0.00167382	0.76212092	0.77842359	0.66049352
cg25476130	8	0.00925322	0.63046577	0.59318439	0.46866946
cg03309393	6	0.01206219	0.78695892	0.81079202	0.6661623
cg16474118	2	0.00076471	0.84072386	0.73164821	0.50088569
cg04137004	11	0.0008386	0.84061977	0.78785059	0.62640164
cg04057599	11	0.00073118	0.87729776	0.82326506	0.67859023
cg01702055	6	0.00639169	0.62820382	0.58854324	0.41101988
cg07417348	12	0.00160583	0.29170051	0.32227759	0.14290996
cg06857289	4	0.0015667	0.38124065	0.47864244	0.27598043
cg18120446	4	0.00949739	0.64990616	0.75042611	0.47298328
cg00339682	4	0.00395839	0.70435265	0.73050566	0.42922334
cg00964997	4	0.01435007	0.76957051	0.84720557	0.57508081
cg22560214	4	0.0157632	0.73512533	0.81242244	0.57150283
cg06635722	4	0.01162689	0.57251415	0.64119401	0.45692237
cg05710540	4	0.00736725	0.64103509	0.65503587	0.53048112
cg19019849	4	0.02633873	0.61351786	0.65583619	0.51268812
cg14144025	4	0.00364684	0.35368331	0.42256349	0.25291954
cg02373784	4	0.0336255	0.66215744	0.72964289	0.53532189
cg10134799	6	0.0008696	0.69346137	0.63397108	0.4420081
cg17593342	6	0.00555868	0.6802823	0.67500722	0.54697558
cg02849507	16	0.00441677	0.67399339	0.65140697	0.44664927
cg01612366	4	0.00756169	0.27738064	0.27591938	0.15400253

cg26508928	18	0.02672791	0.64918736	0.65600021	0.54061963
cg21649089	17	0.00354864	0.4418972	0.42368549	0.30301118
cg10519417	17	0.00223317	0.7258834	0.68259312	0.48272859
cg09584358	17	0.00755255	0.5346157	0.52482208	0.33003738
cg15893295	6	0.00445086	0.74079476	0.70228846	0.4981656
cg12197573	18	0.00175728	0.51423388	0.48559065	0.34472918
cg26104072	5	0.02763008	0.45396862	0.50866683	0.35361093
cg22164009	6	0.01687692	0.49803044	0.48346122	0.34055514
cg08136020	6	0.00627871	0.68303959	0.6580781	0.44478449
cg18112794	21	0.00223317	0.2838576	0.26697753	0.16325402
cg18489994	4	0.00180551	0.46583946	0.38776233	0.20023914
cg25827670	17	0.00067859	0.69118531	0.5863973	0.40255551
cg09663343	11	0.01309153	0.78125529	0.75265615	0.61867207
cg03176917	19	0.00128812	0.43147745	0.41612848	0.14354481
cg23104436	11	0.00132036	0.89291046	0.84068529	0.72660942
cg13911697	11	0.00074016	0.85822441	0.83722922	0.73013804
cg26250400	19	0.00197007	0.3528754	0.34982768	0.24411632
cg27584135	3	0.0051502	0.64797669	0.69591983	0.42650446
cg11997536	19	0.00103895	0.42049375	0.43057735	0.26581731
cg07812957	19	0.00280852	0.66628414	0.57175959	0.45061346
cg11492723	3	0.00878866	0.34553865	0.36636963	0.22484725
cg20405742	6	0.00217555	0.74503601	0.65416672	0.51277853
cg02292809	6	0.00333361	0.47398215	0.58975597	0.35608386
cg26906217	11	0.04433808	0.59170309	0.6117786	0.42625298
cg04456219	7	0.0292294	0.58082032	0.52187961	0.37989367
cg08939394	1	0.00164877	0.63938432	0.62188267	0.39759251
cg22146612	6	0.007634	0.75437247	0.73296572	0.62381474
cg12512771	7	0.02080485	0.70380134	0.73508634	0.50565669
cg22012281	22	0.00486472	0.58357496	0.57606675	0.4148116
cg06937927	8	0.0011206	0.6289096	0.61797064	0.48551821
cg04139043	1	0.00066232	0.71718307	0.61494912	0.44872896
cg18890112	1	0.01891509	0.54060046	0.55886353	0.42474387
cg17109725	1	0.00855749	0.44934903	0.45408434	0.32368313
cg24095864	22	0.00299125	0.39455763	0.40405258	0.27321964
cg26220594	1	0.01657372	0.36859499	0.40307783	0.23152757
cg11630152	7	0.00362881	0.82585096	0.72849153	0.62007156
cg06615816	3	0.00349009	0.49673574	0.49185031	0.3763952
cg15509286	3	0.00333721	0.6826706	0.66595866	0.50557564
cg12253071	4	0.00572656	0.74795768	0.70319432	0.59288753
cg07866909	2	0.00065949	0.77439834	0.66664743	0.50129707
cg20082877	18	0.00144767	0.63473194	0.62295237	0.34835534
cg14799457	18	0.01015464	0.5866579	0.53924357	0.42624919
cg23614811	17	0.00947108	0.43019341	0.41346425	0.22995618
cg26727231	8	0.00073547	0.63978616	0.56416748	0.45144845
cg09772075	8	0.00506299	0.64380373	0.62544455	0.4677142
cg09539496	8	0.00155548	0.71291366	0.69288794	0.55309213

cg18014547	2	0.00320302	0.8616179	0.82981493	0.71377814
cg23047322	17	0.00263648	0.75631874	0.73740713	0.63662168
cg06195193	13	0.002776	0.26416468	0.28130045	0.1580562
cg27282397	2	0.00092783	0.86496377	0.80297673	0.64271522
cg10149296	2	0.00178025	0.82260071	0.75751693	0.57451136
cg04526480	1	0.00174783	0.47097901	0.40465692	0.30305128
cg23489384	14	0.01398885	0.41370431	0.49054428	0.19138756
cg01298102	16	0.00857669	0.6592922	0.63750806	0.50664278
cg13048903	13	0.0067125	0.68007506	0.75210301	0.53986578
cg00580562	10	0.04980289	0.49776228	0.51357116	0.36085546
cg26562263	19	0.03368408	0.67007696	0.74313696	0.56465074
cg04526361	7	0.00575863	0.46855528	0.54944566	0.36733068
cg13819609	9	0.00491697	0.82831588	0.83944788	0.70781185
cg08243401	13	0.00159552	0.78845312	0.77395073	0.62904971
cg03499161	12	0.00380612	0.40725539	0.38977552	0.23167442
cg01164574	21	0.00167443	0.50184781	0.45574932	0.29116283
cg03720745	8	0.0013706	0.54545767	0.50115361	0.38191976
cg11260715	7	0.01463726	0.43194783	0.44320712	0.28091829
cg23261107	7	0.01457962	0.79746352	0.81776102	0.69401997
cg17722435	7	0.00627871	0.793261	0.7584339	0.63896944
cg04819760	10	0.00547327	0.37411824	0.38293825	0.21695754
cg25324047	10	0.02496653	0.73566496	0.77339233	0.62144574
cg17077610	10	0.00501214	0.57323678	0.6195407	0.33629959
cg15772157	10	0.02229636	0.7501667	0.75871467	0.54762831
cg15732079	7	0.00065949	0.73788416	0.57178701	0.4171243
cg10193711	14	0.0135118	0.5796739	0.54150474	0.35954094
cg13716585	14	0.01158752	0.70473555	0.70804345	0.53769991
cg26118358	14	0.00270636	0.6883165	0.59581589	0.35012565
cg05068848	4	0.01019185	0.82565115	0.82717627	0.62842971
cg00045910	10	0.00387834	0.26030111	0.30456461	0.15665625
cg04349084	8	0.00595714	0.69665007	0.67665791	0.4538444
cg14893576	2	0.02794807	0.39288954	0.3845999	0.25430802
cg04427254	19	0.00151706	0.31951464	0.29846028	0.16079061
cg16152753	10	0.01452282	0.60716891	0.59319362	0.41896142
cg12228534	19	0.00582851	0.63348015	0.64211314	0.50361723
cg01364674	1	0.00103808	0.61275037	0.55151513	0.45111595
cg07447769	13	0.03660254	0.65193137	0.63011829	0.51355425
cg04104789	15	0.01991471	0.63390652	0.65890723	0.52829861
cg24911283	4	0.02151984	0.75996375	0.7326381	0.61953134
cg12378001	7	0.00065949	0.85611354	0.77477612	0.62028521
cg02322889	4	0.00065949	0.78370003	0.70766147	0.58824791
cg18565795	10	0.01376061	0.75913511	0.71575226	0.61196565
cg12899747	3	0.00855749	0.64356231	0.64621115	0.43950098
cg00150785	20	0.00081046	0.67736303	0.56908476	0.39023311
cg10097313	14	0.00065949	0.78411621	0.70298227	0.4949627
cg14830371	8	0.00107365	0.29302051	0.21905682	0.11230261

cg17896243	8	0.01235814	0.46200999	0.44407497	0.29011006
cg03002688	4	0.00309052	0.71247749	0.66888493	0.44952852
cg21544415	12	0.0048909	0.5736952	0.49415889	0.37794169
cg10780778	6	0.01971434	0.58307089	0.61808445	0.45741002
cg23756173	17	0.01463726	0.45127279	0.43487307	0.30648864
cg27134139	10	0.00369078	0.36316075	0.36160494	0.24564528
cg23221603	2	0.01095648	0.69247796	0.71211358	0.54489901
cg09654261	11	0.00065949	0.74455242	0.63417491	0.48232854
cg09395805	6	0.04649715	0.58547361	0.62500384	0.46426095
cg17642941	7	0.00903599	0.79903835	0.87278086	0.64440707
cg06982190	7	0.03523391	0.58704043	0.65534438	0.45063297
cg26533311	6	0.01456284	0.45027952	0.42446574	0.28293717
cg16211174	7	0.00897906	0.63515346	0.62314197	0.49773217
cg17694351	7	0.02979258	0.66611512	0.66244741	0.42402412
cg02063817	7	0.02763008	0.69258691	0.74969476	0.56813161
cg21252523	7	0.02078916	0.73328363	0.72524221	0.5871032
cg05057910	7	0.00757575	0.58351174	0.57348286	0.42329416
cg04004787	7	0.00551198	0.80487728	0.82449405	0.56275693
cg23346622	7	0.00228505	0.31784024	0.28928337	0.18181205
cg23998071	7	0.00702677	0.4480308	0.43870159	0.27433534
cg09678539	2	0.01769764	0.60211802	0.5930299	0.46412
cg03402926	11	0.00095394	0.57465343	0.57043663	0.43350035
cg21437157	8	0.00450197	0.4430854	0.46073237	0.24687617
cg04522310	16	0.00211366	0.7182449	0.69608159	0.46034032
cg03834116	2	0.01678444	0.58437176	0.5344052	0.42907265
cg12143499	12	0.01296171	0.69880406	0.70928655	0.49579158
cg15921887	10	0.00218799	0.53312139	0.46680501	0.36115046
cg17284116	11	0.02008355	0.67941399	0.64398723	0.54043438
cg01037082	22	0.04248615	0.70159792	0.74429295	0.55251585
cg22500261	22	0.00323448	0.6838412	0.66984238	0.33496064
cg17955599	22	0.00964959	0.66032471	0.65591128	0.52094443
cg13873324	1	0.00422127	0.48151804	0.47774388	0.30276363
cg13215862	15	0.0088828	0.45350579	0.47901506	0.33001688
cg25653641	6	0.0122594	0.63128776	0.61135865	0.48269973
cg27535677	6	0.03934159	0.72053649	0.74090064	0.592411
cg22452837	6	0.01029727	0.30633891	0.30650977	0.17278309
cg10582608	6	0.03686125	0.36826558	0.33081643	0.22931986
cg00204512	16	0.02016391	0.58879681	0.52417378	0.42089516
cg01116161	5	0.0249821	0.90762939	0.90182725	0.77842198
cg27198040	12	0.00878866	0.78118672	0.75292617	0.6209335
cg22863700	5	0.03832563	0.69194419	0.63275843	0.51704882
cg04926881	7	0.00100759	0.53486094	0.48622874	0.22173728
cg24561305	13	0.00553815	0.41365428	0.41238524	0.28078071
cg10368834	16	0.00722201	0.64643775	0.64139389	0.53110975
cg21831467	13	0.02389893	0.73570209	0.76002334	0.63203005
cg04745064	6	0.00195644	0.77667487	0.7443352	0.64072216

cg11638399	8	0.03366673	0.67782166	0.67434799	0.51311629
cg15717275	22	0.00857669	0.61674559	0.61657598	0.42939077
cg25699073	6	0.03452515	0.881593	0.8854617	0.77572235
cg06032337	6	0.00429038	0.63530038	0.58549288	0.23741592
cg20228636	6	0.02003709	0.78035259	0.77605319	0.5494641
cg11383134	6	0.01430792	0.80944121	0.85078536	0.65509324
cg03449857	6	0.0157632	0.77504251	0.77088912	0.49539941
cg15570656	6	0.02641471	0.79017031	0.7951158	0.54415016
cg02157626	6	0.00659132	0.77779618	0.79098544	0.5154296
cg13835168	6	0.03541631	0.67284324	0.64970872	0.40682267
cg08041448	6	0.04407901	0.62640207	0.6403526	0.41053073
cg24100841	6	0.00763406	0.75869924	0.77041731	0.50300068
cg19636627	6	0.03587201	0.81067065	0.83361469	0.57566458
cg06458771	6	0.03914927	0.58367943	0.65059569	0.38039731
cg06503981	6	0.00560808	0.84688189	0.79873518	0.60666677
cg15967278	8	0.01787304	0.66773627	0.70450617	0.54227993
cg20907614	8	0.01540791	0.81611183	0.76855461	0.66494565
cg00578614	6	0.0044883	0.52039988	0.57943676	0.33575857
cg09558425	10	0.00074381	0.6719681	0.58963582	0.4738622
cg06480353	7	0.00065949	0.53731677	0.38377243	0.25480201
cg00344308	3	0.0071433	0.75706766	0.75572152	0.54195719
cg27268352	10	0.00754448	0.77794989	0.84260736	0.63664247
cg02640104	13	0.00339981	0.5576056	0.55890187	0.26575457
cg16783186	2	0.00144772	0.66111788	0.61181192	0.38603478
cg10577819	2	0.00066232	0.85915822	0.76388014	0.62326136
cg11564601	22	0.02002893	0.41273498	0.41111999	0.23989808
cg14129735	6	0.01481543	0.67493423	0.72162989	0.52376562
cg20873416	6	0.02087024	0.76307795	0.78408666	0.63419518
cg10784519	6	0.00530406	0.41760288	0.41788956	0.23072908
cg03813377	6	0.0147912	0.35097149	0.33925532	0.22275712
cg07082447	11	0.03783756	0.67077745	0.68213956	0.5348759
cg11781335	10	0.01657372	0.6401527	0.70333199	0.53107085
cg14189441	10	0.00068471	0.80290559	0.73591223	0.570632
cg01511901	13	0.00139627	0.26609049	0.24150728	0.13051862
cg18890461	6	0.0097133	0.30841292	0.30541077	0.18991821
cg05775862	10	0.00398606	0.25849256	0.2423733	0.11534422
cg13426642	12	0.00220808	0.72748283	0.70766426	0.55201973
cg03127349	10	0.00065949	0.58063212	0.44615906	0.24968258
cg07851290	2	0.01631013	0.39400456	0.3859055	0.27415775
cg02914097	3	0.00228672	0.48011654	0.42318494	0.2453472
cg01076704	20	0.01484626	0.67792625	0.71418058	0.52896627
cg00516092	20	0.01390151	0.34239069	0.32819258	0.20375634
cg18016304	15	0.01512979	0.62223069	0.637204	0.52024949
cg06618134	5	0.03896039	0.47395474	0.39720488	0.29069883
cg06434490	6	0.00444287	0.79657398	0.79206443	0.65423803
cg19410609	2	0.00351772	0.8159067	0.86235303	0.65590113

cg00960906	5	0.00131467	0.49056747	0.39817635	0.2872343
cg09372486	19	0.04615769	0.55647964	0.55202929	0.43115409
cg07096763	1	0.0156946	0.44670821	0.45086189	0.30120388
cg20465333	22	0.02802634	0.48415384	0.49419608	0.37924533
cg15025379	11	0.00065949	0.63713401	0.54828668	0.37761412
cg05935961	11	0.0013706	0.55478935	0.52510467	0.42017249
cg19802929	14	0.01694602	0.6966157	0.70533104	0.53432119
cg17942618	1	0.00186737	0.31416565	0.31211825	0.19317053
cg00119778	6	0.01877442	0.23253312	0.23921813	0.10178313
cg12362478	7	0.01677529	0.47216013	0.50465116	0.3625508
cg10334416	17	0.00273943	0.83587219	0.810915	0.68242033
cg01987353	17	0.00563693	0.76640637	0.80812661	0.57116376
cg07007382	6	0.01020581	0.34165149	0.38512021	0.21968559
cg07056079	6	0.01782206	0.26398739	0.25918524	0.1522272
cg02273797	1	0.02343917	0.47468681	0.48883869	0.36394015
cg14232108	9	0.00369903	0.59624107	0.63428536	0.36033755
cg23138250	5	0.00178025	0.53028301	0.45303112	0.34845231
cg25898192	15	0.00344958	0.30487988	0.2306965	0.1071693
cg06223926	10	0.00072814	0.72101426	0.62668229	0.39863674
cg16031846	8	0.00349377	0.49822498	0.5231728	0.39134798
cg11760395	10	0.00082513	0.70009781	0.58961807	0.44241787
cg08365609	19	0.00947108	0.78965516	0.73135937	0.58168748
cg12016405	6	0.002727	0.80488945	0.78415022	0.65996334
cg15626881	7	0.03934159	0.62214063	0.62708857	0.51821112
cg05512561	13	0.00263491	0.49719265	0.47200953	0.33515475
cg03116182	11	0.01473127	0.38394092	0.41330493	0.27045435
cg08854586	13	0.00128725	0.51025437	0.46081412	0.35267046
cg04762129	6	0.02895902	0.49983328	0.48430705	0.36265748
cg20170223	20	0.0156698	0.5662969	0.5856014	0.45974747
cg26550214	5	0.00233439	0.27353663	0.22922834	0.12301145
cg23201812	11	0.00170239	0.2929566	0.23348139	0.10220502
cg06287548	21	0.01820811	0.59954794	0.64016775	0.46199002
cg00974255	17	0.00280852	0.67781994	0.68684718	0.55042168
cg20814718	8	0.00076471	0.55040764	0.58304425	0.41532433
cg15481493	11	0.01317469	0.82385447	0.83077002	0.69416399
cg07979348	20	0.0006736	0.62301357	0.51279485	0.4109111
cg12837463	7	0.01670995	0.67553501	0.70487917	0.54000924
cg23155333	19	0.01665552	0.5987986	0.63487834	0.47967076
cg17998530	22	0.0042395	0.35890414	0.35431193	0.25150943
cg17367078	22	0.00925322	0.47447119	0.44930225	0.34795452
cg18388547	1	0.00174783	0.49942818	0.50723229	0.29451552
cg19530168	11	0.00197639	0.62497757	0.54155044	0.38001392
cg21913652	11	0.01178392	0.78154453	0.79258016	0.63258582
cg04244152	10	0.00073547	0.83496341	0.76229619	0.63846422
cg08272572	19	0.02086555	0.50659093	0.55138505	0.40311762
cg01166876	2	0.00732415	0.70653396	0.72054685	0.60233194

cg03214130	7	0.00380612	0.43179276	0.44082236	0.24974244
cg16569309	19	0.02512074	0.82347334	0.8298727	0.58783222
cg03149958	6	0.02595684	0.69152994	0.67819776	0.57686845
cg18814699	7	0.00506656	0.79572051	0.74826995	0.63651109
cg14420953	6	0.00092849	0.67074617	0.60553314	0.48700304
cg13057055	17	0.02496653	0.46568227	0.46483271	0.34975191
cg21172011	21	0.02614058	0.76993423	0.8376607	0.65291785
cg05389236	6	0.00202314	0.7033758	0.67048828	0.50108665
cg03796381	15	0.00372084	0.66226422	0.64682457	0.42757717
cg26027052	6	0.03934159	0.26024689	0.29902272	0.15673883
cg13017983	8	0.00150566	0.32865844	0.27667938	0.15449905
cg14214856	10	0.00376781	0.43051981	0.45697573	0.28786922
cg10587854	3	0.03528716	0.39266457	0.44764953	0.27099895
cg06971224	10	0.02757672	0.63252737	0.54353532	0.30492773
cg20745381	1	0.0103161	0.3125792	0.30678189	0.2039843
cg04640920	15	0.01096175	0.82449574	0.8270662	0.71225074
cg22567585	15	0.0062619	0.40660813	0.4269752	0.26151918
cg21952052	1	0.01236318	0.29615875	0.30106502	0.18753689
cg02184226	17	0.0017447	0.76609448	0.69734203	0.56745864
cg03623982	6	0.00194569	0.27898341	0.26274182	0.13993698
cg10327067	8	0.00068559	0.43408955	0.32099297	0.17790176
cg15134714	8	0.01842703	0.5115632	0.47373042	0.35789223
cg03097134	6	0.00079224	0.56646495	0.46445481	0.33189703
cg23521140	2	0.00230737	0.42599477	0.38083169	0.25486605
cg02944084	17	0.00182655	0.5084155	0.51243897	0.36184073
cg09063683	5	0.00938113	0.35704513	0.36292937	0.2462521
cg18207999	17	0.00280561	0.52178961	0.52665175	0.27676107
cg16163382	2	0.00713358	0.76220982	0.78978065	0.53540726
cg19501909	22	0.00243597	0.28021714	0.25723891	0.15240503
cg00760950	13	0.00109306	0.78716654	0.76358559	0.61614384
cg08064488	21	0.00918003	0.67034296	0.76227118	0.55872729
cg24459147	21	0.00202174	0.46269521	0.56341185	0.28306847
cg09408917	21	0.01262374	0.62130435	0.69213921	0.40740113
cg06368184	21	0.00805241	0.63310147	0.72094443	0.39328867
cg02730156	21	0.00648231	0.40337586	0.4262434	0.25686257
cg16404784	14	0.00450697	0.53057715	0.52030342	0.3826109
cg07989851	15	0.02457877	0.69369153	0.74160805	0.57793802
cg04316353	6	0.04013049	0.68927875	0.72560833	0.58225435
cg19634252	4	0.00352201	0.53628245	0.49281705	0.38200634
cg05191655	4	0.0157424	0.48692988	0.50507246	0.32411863
cg03906681	15	0.0078763	0.7230758	0.69314618	0.53377705
cg24802813	7	0.00426883	0.46464979	0.49406096	0.29476408
cg16703420	14	0.00958397	0.44655419	0.37011489	0.26409964
cg08986840	8	0.00622936	0.32212329	0.30252991	0.15600877
cg11422851	8	0.00066204	0.90914419	0.82233642	0.69744528
cg08037478	12	0.01137235	0.65752403	0.64898477	0.54020364

cg27109912	17	0.00072814	0.64404203	0.51475324	0.36327566
cg26300517	4	0.00065949	0.90570827	0.84445691	0.73152072
cg22588983	5	0.00107266	0.8076217	0.73186197	0.61494712
cg27638284	20	0.0156946	0.54524345	0.55560293	0.31614006
cg14118997	14	0.008876	0.32955604	0.36335363	0.22817692
cg21949194	2	0.00595346	0.70025296	0.74264635	0.56056841
cg18275589	5	0.01284723	0.369284	0.34471401	0.21879105
cg26494138	2	0.02577962	0.64768014	0.71029932	0.53728766
cg05005073	1	0.00295889	0.83233011	0.80154218	0.68162603
cg25119946	17	0.00270728	0.89469712	0.87524723	0.74544435
cg03453870	15	0.00074381	0.86607419	0.80602629	0.63374494
cg09290175	22	0.00116059	0.93886566	0.913445	0.78379465
cg25673591	17	0.00212345	0.61133565	0.55277714	0.41910944
cg04804321	8	0.00798815	0.78679355	0.79776986	0.67312372
cg09126279	4	0.03068939	0.67341625	0.73442213	0.52801722
cg19684783	5	0.02944061	0.72952842	0.74245725	0.62718021
cg14955976	11	0.0011469	0.2912029	0.31320839	0.16685621
cg07138070	7	0.02240881	0.42314703	0.41113433	0.30235006
cg06581409	19	0.00092849	0.64454593	0.55030019	0.41314054
cg02624905	6	0.00858831	0.75221768	0.82622666	0.54881995
cg24369185	6	0.0281492	0.78660538	0.79224532	0.68524629
cg21052660	6	0.04791139	0.69301578	0.74855432	0.47993981
cg17609158	6	0.02368354	0.70721657	0.7511086	0.48952379
cg10274208	13	0.01718572	0.7486055	0.7406769	0.59886663
cg26325867	6	0.00201167	0.50914152	0.49217973	0.28993088
cg08655662	6	0.00081925	0.68774522	0.64785366	0.50458378
cg20263901	6	0.00084118	0.43207028	0.37834302	0.25495469
cg05234552	6	0.00147559	0.59372484	0.49420063	0.29755182
cg20893203	6	0.00525399	0.49429712	0.46604512	0.24379415
cg06484100	17	0.00103447	0.53633762	0.44300828	0.32240986
cg02420480	7	0.00405622	0.76152889	0.75136669	0.49910486
cg24202916	20	0.00132036	0.56847421	0.58613458	0.46702022
cg24484352	17	0.01954678	0.66757662	0.76331102	0.54764276
cg13057506	15	0.00335524	0.56263688	0.56278945	0.38990732
cg04783450	6	0.00071044	0.763751	0.65629821	0.48308346
cg09244312	13	0.00073118	0.81804523	0.72174477	0.50673956
cg16230352	8	0.02828378	0.6558536	0.67487091	0.52894367
cg21293933	20	0.00622936	0.54995867	0.53494105	0.42950922
cg17367816	10	0.01616965	0.33199321	0.32900112	0.22820123
cg07339138	3	0.0009934	0.82065546	0.78863594	0.63323243
cg07749597	5	0.01244196	0.76718447	0.76389052	0.61528566
cg14697657	5	0.00212575	0.23076515	0.24700102	0.12548469
cg18739675	2	0.02190777	0.80166804	0.85429411	0.70043746
cg21567649	2	0.01245683	0.41269683	0.42941292	0.30349263
cg18257574	2	0.0008947	0.44607594	0.37903798	0.20240758
cg14718363	2	0.02122541	0.60115559	0.61496796	0.45769958

cg14675881	2	0.01212811	0.68554059	0.77412887	0.5805051
cg00568910	1	0.02917135	0.73736424	0.75493664	0.59313427
cg20788482	22	0.03257065	0.63365108	0.62800313	0.49898389
cg03143046	6	0.00908203	0.39944996	0.32622271	0.21844046
cg16540590	5	0.01640621	0.7292954	0.70834732	0.60190557
cg15142335	1	0.00354163	0.78606136	0.78405566	0.57113152
cg18403852	21	0.00136593	0.70137526	0.63038638	0.52462258
cg12670123	10	0.01021896	0.41086399	0.50094849	0.28665086
cg07121693	10	0.00218799	0.48073165	0.46325461	0.33745308
cg26606789	21	0.00461206	0.26032269	0.27576909	0.15348158
cg02698668	19	0.00100759	0.39649571	0.29987587	0.16626302
cg02059176	2	0.00275824	0.7157742	0.7118549	0.43726427
cg02068486	6	0.01310065	0.73975265	0.71603866	0.61009744
cg01482969	11	0.03891003	0.33785341	0.38180281	0.23336315
cg13979708	6	0.03857163	0.66805263	0.68106662	0.54193827
cg04781339	21	0.01334584	0.47265498	0.52127319	0.3472987
cg18703515	10	0.00106594	0.80080198	0.77995576	0.67251394
cg12111500	21	0.02310662	0.77970303	0.73232842	0.59888067
cg13413982	13	0.00971274	0.87541116	0.8746338	0.76617444
cg00362657	21	0.0423854	0.54982742	0.59110854	0.42609375
cg26203328	15	0.00174123	0.40483633	0.41058659	0.19818347
cg15006828	20	0.00174783	0.39564856	0.37789015	0.24117233
cg07780762	1	0.00073378	0.52411545	0.47749168	0.33163776
cg22912834	2	0.01607325	0.74743623	0.83691954	0.62306518
cg10068996	2	0.02284505	0.8096165	0.84393278	0.69633824
cg13207733	7	0.0058315	0.57672963	0.56392232	0.39745603
cg10281509	2	0.01678444	0.5369822	0.58918522	0.39948093
cg06798115	3	0.00344958	0.59796953	0.5532349	0.4221789
cg21981144	19	0.00068904	0.42655227	0.37126338	0.26488959
cg26587014	19	0.00708587	0.67793527	0.67137293	0.56681074
cg05168229	13	0.00216509	0.46047859	0.48044263	0.27014528
cg13496662	19	0.02473841	0.56706194	0.55281328	0.43730004
cg06739525	2	0.00299664	0.87854844	0.86312274	0.75511692
cg05091585	13	0.00142756	0.57622914	0.50972544	0.40191596
cg13076829	18	0.00073265	0.56041986	0.42199555	0.32150452
cg01632562	6	0.02215534	0.77588627	0.7761938	0.64119356
cg23425762	18	0.00501039	0.6460605	0.53031858	0.40591557
cg15835542	6	0.02002988	0.24883892	0.24306992	0.13975066
cg26904260	11	0.03692847	0.43622981	0.44359752	0.31434926
cg17527177	17	0.03346286	0.68472576	0.72332618	0.55677954
cg10521147	13	0.00655853	0.34635932	0.37703677	0.22362202
cg19061690	17	0.01038666	0.35840972	0.39995148	0.24074404
cg24954967	3	0.01011462	0.83500919	0.84043663	0.6805665
cg02352281	3	0.0012504	0.74644739	0.72309209	0.48156379
cg27609217	11	0.03342972	0.64129917	0.63881283	0.45946027
cg01514353	22	0.00857669	0.43717657	0.46193193	0.31770776

cg19916740	22	0.01794611	0.63382513	0.62115046	0.49469186
cg01993552	19	0.00073918	0.70087966	0.57353681	0.45626725
cg25255850	17	0.0051141	0.49523086	0.50404779	0.33974114
cg05322273	1	0.00937465	0.59917603	0.72497903	0.4858465
cg14686297	22	0.00751269	0.63053123	0.63327778	0.44357207
cg25636466	6	0.03778772	0.66450791	0.69915761	0.53390779
cg22005990	17	0.01096356	0.52692828	0.51323637	0.35842316
cg21082028	17	0.00297384	0.75862888	0.73169634	0.58138115
cg02311193	17	0.00516729	0.68680368	0.63601059	0.42464925
cg03666350	2	0.00065949	0.70574866	0.52357892	0.40867901
cg22266211	17	0.00499799	0.30175808	0.28801959	0.18763556
cg12131828	17	0.001181	0.27393407	0.26672906	0.14853045
cg03064100	2	0.01668465	0.66678791	0.63028343	0.52179268
cg16097858	2	0.0191912	0.56027118	0.62541142	0.40431772
cg08381504	3	0.02870038	0.7598738	0.79789601	0.60551807
cg09571950	3	0.04213177	0.75048352	0.78742414	0.60294039
cg15321306	12	0.00092661	0.9018846	0.87140452	0.70973589
cg18645361	20	0.00100011	0.8895346	0.84706753	0.7458039
cg25983901	7	0.00155276	0.75413198	0.65985652	0.52631775
cg17411016	2	0.00142756	0.47937457	0.41808225	0.2489875
cg13097800	14	0.00118952	0.59529433	0.61771573	0.47599566
cg03273315	8	0.0036184	0.47234171	0.4448262	0.31807649
cg09455881	3	0.03208512	0.63623901	0.70292754	0.51069175
cg27305917	21	0.01666517	0.58165413	0.56961112	0.46186878
cg10511890	11	0.00806542	0.70462675	0.722534	0.55051924
cg13912307	11	0.00248044	0.67314722	0.65853767	0.50772346
cg25439798	21	0.00065949	0.90755023	0.81929824	0.68112149
cg15317049	21	0.00079512	0.78777643	0.73970937	0.49132443
cg12457659	21	0.00073378	0.64024193	0.5468442	0.36837146
cg03147815	21	0.01357235	0.72646499	0.70499297	0.59424152
cg22786667	7	0.02310117	0.78196026	0.80307665	0.62650253
cg01423916	11	0.00758361	0.72382138	0.74463577	0.56883357
cg15657641	11	0.00332266	0.36539274	0.36711001	0.21920314
cg22538557	19	0.01313275	0.61062454	0.65498432	0.50092705
cg09140531	6	0.00518187	0.67825976	0.73738429	0.51281549
cg14398113	15	0.0355165	0.75708989	0.77593642	0.63012297
cg18655633	19	0.01074556	0.74600173	0.73332395	0.61337498
cg14241836	7	0.00083771	0.88654214	0.86034094	0.65058264
cg23981172	14	0.03021634	0.40813971	0.38990626	0.289421
cg02134839	17	0.00238047	0.72188968	0.69054034	0.54830301
cg21286117	17	0.00066232	0.85407003	0.77717764	0.66785189
cg25616869	3	0.00065949	0.61430802	0.4746052	0.36720886
cg09889228	16	0.00502478	0.60897477	0.63689824	0.44010623
cg08871545	1	0.00299125	0.56939204	0.63629809	0.38863728
cg13772700	22	0.00112793	0.53582553	0.49218972	0.38887359
cg04149776	22	0.0157632	0.72442413	0.72711213	0.60525406

cg09341796	22	0.00216343	0.62880973	0.58674011	0.48622619
cg00253248	17	0.03743475	0.66180351	0.68702109	0.54729311
cg21393124	17	0.00147559	0.70765712	0.64722642	0.52092947
cg01955492	6	0.01223489	0.39999754	0.41111367	0.2833285
cg15471388	14	0.00581095	0.83005774	0.8248807	0.71944352
cg23391288	13	0.00074016	0.89537003	0.85445115	0.68186656
cg03421236	16	0.04976146	0.49886044	0.5241701	0.39813662
cg07586760	22	0.00414201	0.6078794	0.57690073	0.46631523
cg27166177	22	0.00103895	0.40232893	0.40868401	0.26053065
cg10828561	8	0.00077454	0.71983946	0.66420365	0.52599046
cg11905324	8	0.00067859	0.90481832	0.84766349	0.723377
cg23462772	10	0.00947108	0.57887461	0.55819974	0.32191936
cg05729745	22	0.00218799	0.60988914	0.59411198	0.48199164
cg00657780	16	0.00066204	0.78395489	0.70016694	0.49796202
cg26706803	16	0.0097835	0.29929713	0.2760085	0.1668045
cg20031845	7	0.00911882	0.74197817	0.78442225	0.55955703
cg19604907	12	0.00403008	0.32627847	0.28518354	0.17607623
cg10238080	12	0.03276765	0.65625644	0.66082121	0.52747085
cg26277754	5	0.02326722	0.57019243	0.52488191	0.39718984
cg24549912	5	0.00262867	0.50553609	0.47198541	0.27769656
cg09524946	13	0.00670577	0.29442201	0.29522951	0.17889184
cg07121900	13	0.04648826	0.47236614	0.47733242	0.36767158
cg01955153	16	0.03744929	0.61947211	0.63691646	0.51927958
cg18530299	8	0.00755255	0.40828114	0.40019252	0.27135593
cg07807757	7	0.00219431	0.75674611	0.69394856	0.49471606
cg03663556	7	0.00471011	0.44140785	0.40641357	0.20862287
cg21140898	1	0.01630619	0.55296424	0.55381973	0.43983538
cg08981669	7	0.00489082	0.67013924	0.74667845	0.54586685
cg21814178	12	0.00356822	0.45069399	0.46218044	0.25805779
cg00092400	12	0.02250121	0.72183706	0.73047079	0.53572055
cg22540135	19	0.01613093	0.69877936	0.68884983	0.48706299
cg02964087	16	0.00961487	0.61285941	0.58687819	0.48639693
cg24674269	13	0.01011462	0.73639899	0.76903725	0.5709099
cg11551296	20	0.01423002	0.32975133	0.3707685	0.20058748
cg09225373	6	0.0042005	0.33976164	0.37243334	0.2110764
cg23591955	14	0.0097835	0.46076685	0.45235915	0.29722485
cg17403995	1	0.01210192	0.72209525	0.71854543	0.61407899
cg12071775	14	0.0011159	0.38382774	0.35460121	0.19880704
cg15964159	13	0.00527173	0.81905911	0.8098243	0.69908195
cg00063982	1	0.0015244	0.89511493	0.87423533	0.73444193
cg04373760	16	0.04904378	0.38242667	0.40054489	0.26904969
cg04071967	17	0.00639169	0.51713458	0.5489558	0.36551391
cg12008054	13	0.02002893	0.73549076	0.70887168	0.60875112
cg06049381	17	0.03358212	0.34546552	0.34898584	0.23235552
cg07559178	14	0.00074381	0.73407195	0.61568393	0.46427941
cg11721349	6	0.00081789	0.4014543	0.29342157	0.15573252

cg12790424	14	0.00168532	0.70455887	0.68210246	0.48275936
cg12534147	5	0.00306394	0.56488451	0.50161417	0.27969155
cg16523850	6	0.02530038	0.47781843	0.40698109	0.30694959
cg22021794	5	0.00065949	0.84252915	0.69472317	0.55029285
cg10809166	12	0.01807343	0.63599787	0.67833213	0.49120739
cg07972716	20	0.00240097	0.50104476	0.43793425	0.32754276
cg10129391	4	0.00910614	0.54735776	0.54854447	0.35496168
cg14430614	13	0.00139783	0.82429269	0.78857768	0.68427667
cg04231042	4	0.00092849	0.89325289	0.8373002	0.73604254
cg00187120	7	0.01109894	0.54646899	0.51426088	0.41358652
cg22341513	18	0.0020873	0.52852863	0.52312597	0.3821626
cg02388378	16	0.00384078	0.74871133	0.74412944	0.57880894
cg01961105	16	0.00514	0.78786143	0.77647253	0.6097175
cg07505018	16	0.00207334	0.70370275	0.6677371	0.51494856
cg07455757	16	0.00315261	0.69592465	0.66308215	0.53062991
cg06993307	16	0.00334886	0.49229175	0.44621578	0.24400504
cg27327911	16	0.00218597	0.28675155	0.27922241	0.17352119
cg15916160	16	0.03100155	0.54382391	0.56878834	0.36204853
cg08454455	16	0.00315265	0.63555733	0.61138718	0.48203539
cg01047631	16	0.00207996	0.68300258	0.66140552	0.46409229
cg13744172	16	0.00168732	0.69492583	0.66904608	0.30877303
cg00747944	16	0.00243597	0.66402869	0.62596903	0.34726276
cg10214640	16	0.00171661	0.81938831	0.76065321	0.546938
cg06138439	16	0.00275412	0.7263199	0.69797246	0.44661548
cg16387593	16	0.00454901	0.73454631	0.71281442	0.49245516
cg07271394	16	0.0048897	0.50703481	0.52134214	0.36291079
cg08732659	16	0.00576931	0.67307838	0.68257659	0.56584258
cg27577928	4	0.01463398	0.71878218	0.82023475	0.55586998
cg11332163	4	0.02244742	0.69901202	0.79634857	0.5520282
cg03709475	5	0.00320723	0.45400591	0.43864004	0.2352011
cg05616589	6	0.00110772	0.64070802	0.68458938	0.43193031
cg21620078	5	0.00186306	0.72212216	0.68562119	0.57844101
cg15742777	2	0.00487756	0.48560289	0.51886055	0.27133647
cg05403316	2	0.00711398	0.71389637	0.70470183	0.50182878
cg14553705	5	0.04292124	0.58908551	0.64837495	0.47081633
cg05267204	2	0.00967401	0.43666216	0.46892417	0.29318928
cg25750209	2	0.00092849	0.84533335	0.80253194	0.68543246
cg02670637	1	0.00957502	0.71239735	0.6628502	0.53497683
cg25537245	17	0.00332266	0.53903907	0.54376385	0.35770932
cg06551697	17	0.00830906	0.81993103	0.82861892	0.68765057
cg23408558	5	0.00341332	0.34591508	0.41784556	0.22925176
cg16426215	16	0.02911369	0.52641974	0.55185146	0.4070417
cg06821992	5	0.00679223	0.48792571	0.61214184	0.38212327
cg13670898	14	0.00262147	0.65173398	0.62286891	0.47066796
cg08415582	8	0.00520958	0.64289233	0.64478032	0.53823826
cg26236329	11	0.00779924	0.75226354	0.75654954	0.50730219

cg06627087	19	0.02562377	0.78594458	0.85384979	0.63604886
cg07980854	20	0.00082513	0.58176034	0.50910042	0.32593895
cg06815411	4	0.00305182	0.23838102	0.28472766	0.13448588
cg11895596	3	0.0009078	0.80736549	0.76148731	0.64143963
cg03500164	17	0.00886166	0.83051626	0.8158308	0.70541082
cg11718312	6	0.01430792	0.37933478	0.39178914	0.27752286
cg09726198	3	0.00341932	0.52202641	0.46109959	0.32764408
cg23634401	15	0.04997855	0.54534278	0.59184193	0.44436006
cg11661534	3	0.00687165	0.34737878	0.32974303	0.21732759
cg24499677	17	0.00217555	0.69549714	0.64497164	0.5397636
cg10416784	2	0.00156239	0.53551121	0.48059211	0.35376943
cg01360605	20	0.01249982	0.44705043	0.44506483	0.30743332
cg12207248	3	0.02078916	0.56803628	0.56928928	0.43942688
cg13432945	1	0.0009028	0.68973566	0.66823324	0.48796663
cg01556275	1	0.00169556	0.84387477	0.78402033	0.6807496
cg11586930	1	0.00074016	0.83688099	0.75829893	0.62776694
cg02691192	17	0.00372456	0.51352273	0.49718806	0.27668025
cg25766247	8	0.00364279	0.7834151	0.78580885	0.53313598
cg21493768	14	0.00223213	0.60980397	0.66393098	0.39476717
cg05995607	11	0.00211742	0.31745022	0.37431897	0.18320713
cg26915329	13	0.00155166	0.4588738	0.46031407	0.31208467
cg10589330	15	0.00153162	0.79536093	0.7619264	0.65186297
cg07486821	16	0.00192049	0.61523898	0.58178629	0.46883909
cg26124526	5	0.00462871	0.65091575	0.63055968	0.49799947
cg26930006	14	0.00548311	0.35391433	0.39229066	0.22187516
cg20321319	8	0.00669355	0.41876286	0.41319876	0.31273512
cg03032497	14	0.00937895	0.5788785	0.57771937	0.44328443
cg08082810	14	0.03190657	0.75718918	0.77586858	0.63061811
cg19610529	14	0.01064534	0.72289003	0.72771041	0.60236739
cg02320543	14	0.00664361	0.42924944	0.44811995	0.26770582
cg18103859	14	0.00822746	0.66512768	0.69524449	0.49675648
cg26236177	14	0.00803433	0.527201	0.55381961	0.32729026
cg12818596	14	0.02944061	0.49220583	0.55143714	0.38542264
cg21177821	14	0.04319217	0.7059969	0.71077407	0.58059098
cg04166812	14	0.00323448	0.77204485	0.78994129	0.56676015
cg10858480	14	0.01015119	0.723774	0.72388075	0.61724875
cg15540602	14	0.01145563	0.65592197	0.63451284	0.43598668
cg05854694	14	0.01495982	0.74628436	0.77279208	0.61216786
cg23512231	17	0.01501879	0.63067937	0.56897821	0.46659733
cg24753988	14	0.00168532	0.66953428	0.63020457	0.48876671
cg14272084	14	0.00184462	0.59785894	0.55450017	0.39261936
cg06011086	8	0.00540086	0.581363	0.61694657	0.41637839
cg17922998	8	0.00219764	0.79470402	0.7654948	0.50081272
cg12171761	8	0.01026031	0.59193348	0.59920577	0.48575132
cg01279413	20	0.01059488	0.47444913	0.45061735	0.3335918
cg19532942	14	0.000777	0.61750512	0.49205638	0.37056093

cg18728780	14	0.00531242	0.544971	0.56385771	0.37428205
cg11633080	7	0.01237894	0.67116008	0.67132269	0.56172194
cg06520521	11	0.00195549	0.27448195	0.24611487	0.13945012
cg01719405	14	0.00353532	0.63911732	0.62736659	0.40899762
cg21568661	15	0.01580869	0.4822723	0.4896593	0.28793299
cg10600730	20	0.00076471	0.77265906	0.67791771	0.52933764
cg18995788	8	0.00102765	0.83003963	0.80575216	0.65317243
cg15835664	1	0.00065949	0.58746879	0.4609275	0.32606754
cg19169932	15	0.00223317	0.81828606	0.81494148	0.64882774
cg22005336	5	0.02835259	0.38501893	0.41112524	0.27023716
cg04770088	17	0.0169634	0.65481927	0.70133679	0.54431338
cg17415265	17	0.0035378	0.61407045	0.66656714	0.43247453
cg03741862	17	0.00798815	0.29190186	0.33320319	0.16641443
cg06178669	11	0.01744696	0.56438213	0.56983172	0.43444171
cg19064304	15	0.00844494	0.75675991	0.72421811	0.62188097
cg13617812	15	0.01154548	0.55213061	0.54505199	0.42212014
cg26971423	15	0.00299664	0.76767424	0.67752417	0.54022218
cg22046201	1	0.00857669	0.63185148	0.61728075	0.40540579
cg04462931	7	0.01373528	0.81632374	0.82041405	0.54471688
cg07264203	3	0.00774408	0.73392642	0.81312879	0.62859423
cg14016418	2	0.00514311	0.40149617	0.33311151	0.2198505
cg24710503	11	0.00184462	0.83393654	0.80279765	0.66639699
cg18926409	15	0.00623289	0.75918458	0.7409886	0.56497249
cg07076836	15	0.01317021	0.80117866	0.7850651	0.64661884
cg20889322	11	0.00336153	0.46359805	0.4681863	0.30139284
cg07029024	11	0.02075286	0.58392671	0.55681617	0.4210208
cg05471881	13	0.00129968	0.6300973	0.66724021	0.4903671
cg07735220	2	0.0103127	0.3572173	0.3470316	0.22322903
cg18129996	7	0.00101054	0.90354108	0.88885834	0.76465361
cg21221767	14	0.00551242	0.76929918	0.72744887	0.57648847
cg16204717	12	0.0026461	0.46877899	0.4532738	0.30347322
cg23207542	2	0.00520163	0.63992959	0.63485403	0.51891472
cg00802386	7	0.00186737	0.87310642	0.84696224	0.73693747
cg04111102	1	0.00725151	0.64326463	0.69178085	0.5005676
cg08098420	16	0.00666111	0.38401632	0.33335338	0.22758478
cg12078913	13	0.00521564	0.54413919	0.54652052	0.44166927
cg27381488	16	0.00285259	0.38779768	0.37558465	0.21782359
cg08237521	16	0.00160451	0.81290607	0.81415904	0.70530655
cg10723431	14	0.01891102	0.66800781	0.69029343	0.5443852
cg07143815	8	0.00485266	0.32396821	0.34508592	0.21225324
cg16663891	3	0.00563693	0.63771392	0.68650585	0.38401826
cg09693588	3	0.00099274	0.85502151	0.82281593	0.71385565
cg05068866	3	0.00614711	0.77917262	0.76560041	0.61307387
cg14630001	3	0.00181303	0.58344926	0.60568336	0.46279781
cg13851508	2	0.00589429	0.82848797	0.7803218	0.67039805
cg10552446	2	0.00319723	0.61874783	0.69111282	0.44405863

cg08217163	2	0.00255186	0.7111275	0.7033405	0.55693363
cg11241663	2	0.00857669	0.57347737	0.59723084	0.39911714
cg01020413	2	0.00209427	0.7147919	0.74271611	0.55498445
cg01721569	2	0.00276743	0.68207189	0.69956565	0.51793609
cg02676167	2	0.00296953	0.62851487	0.68446011	0.40580945
cg08850641	17	0.00674191	0.40924534	0.36889424	0.26497287
cg23089445	8	0.00696373	0.52217413	0.53948849	0.40632861
cg19111408	15	0.0099603	0.49966761	0.53432065	0.37756853
cg19440115	15	0.00065949	0.68891268	0.60091766	0.49069367
cg18762500	6	0.00184894	0.50935215	0.53522833	0.38741334
cg04033732	16	0.00243597	0.30978487	0.29173964	0.18428936
cg00176043	1	0.00414415	0.43947895	0.37849135	0.26247585
cg26426334	1	0.00126804	0.56963232	0.47960136	0.34339384
cg03188000	12	0.01032661	0.6468426	0.61580501	0.45272896
cg19476334	12	0.00079512	0.80929294	0.78394683	0.64627421
cg22540040	12	0.03710592	0.48673234	0.52310299	0.38275569
cg20972199	11	0.00670817	0.65389956	0.64070077	0.45525072
cg17418276	11	0.00129968	0.68282011	0.64156978	0.41528819
cg24292665	11	0.0035878	0.43657317	0.43431678	0.26680914
cg24610561	11	0.03377212	0.744068	0.8024347	0.64296642
cg19616339	2	0.00181052	0.72059466	0.66946801	0.49787579
cg24178897	14	0.00081925	0.6080414	0.55189967	0.33899464
cg26540315	11	0.00830789	0.74323835	0.77013181	0.61326047
cg17064090	11	0.02252087	0.53237957	0.55124858	0.40918489
cg04718050	14	0.0013706	0.57189903	0.54033965	0.33237997
cg10791884	8	0.0016785	0.84098389	0.85099808	0.71682576
cg08422793	15	0.00796332	0.69555826	0.71103702	0.45046621
cg20175702	15	0.04006803	0.39135581	0.35302501	0.22125696
cg15705813	2	0.03354729	0.52868219	0.52899455	0.37682817
cg26248066	11	0.01541137	0.65506408	0.57076695	0.45283871
cg24680415	17	0.00076471	0.83881521	0.74445298	0.64096471
cg03261725	17	0.00177922	0.72504582	0.69992955	0.55977727
cg24796998	17	0.00480767	0.35995391	0.32564227	0.2139173
cg09362047	17	0.00073118	0.8982764	0.81465629	0.69429476
cg01957897	15	0.00065949	0.74359222	0.60116057	0.47028937
cg27492584	17	0.00947108	0.51460961	0.44094354	0.29091189
cg26962618	15	0.00252483	0.75368622	0.72703638	0.5625639
cg14434062	15	0.00315582	0.70296894	0.66168399	0.37318525
cg16973527	15	0.00221513	0.62153265	0.54833536	0.2829208
cg13427748	15	0.00384078	0.57719084	0.56158318	0.34532827
cg03278514	15	0.00107365	0.80234361	0.71704207	0.45742894
cg24405567	15	0.00631167	0.76228239	0.79707381	0.55615732
cg03108347	11	0.03608849	0.77223628	0.81233669	0.66978015
cg04380269	2	0.01488167	0.65931538	0.65699365	0.53656025
cg20391220	15	0.01910705	0.78810226	0.80787902	0.68059537
cg23153661	2	0.01562094	0.57676811	0.56431835	0.44560668

cg23883632	14	0.00315582	0.70016638	0.66486513	0.40898781
cg24611351	2	0.01558325	0.55413951	0.55565016	0.44424379
cg20928238	14	0.00971058	0.46621373	0.47671891	0.36358187
cg01816783	11	0.00112038	0.42079517	0.46520916	0.28727924
cg27409484	6	0.00930663	0.37056738	0.4068087	0.2303323
cg09478002	2	0.00077739	0.38105983	0.29254201	0.17323992
cg02001991	4	0.00220384	0.58605763	0.60582706	0.3242069
cg02235918	6	0.00112152	0.82798164	0.7283427	0.56466999
cg26094232	11	0.0062669	0.27564337	0.28074643	0.16110322
cg00206507	6	0.00335524	0.65417833	0.69634885	0.48970418
cg06498232	3	0.00279686	0.24182039	0.255768	0.13839388
cg10598503	3	0.00201346	0.78827423	0.69875824	0.55386405
cg09488090	16	0.00295711	0.83516637	0.80861238	0.68836031
cg14260169	10	0.00297228	0.38097114	0.44085921	0.2318742
cg05607079	3	0.00073547	0.89562422	0.85029084	0.74404085
cg25016544	5	0.0131276	0.34807192	0.34710982	0.23458903
cg01768201	5	0.00629935	0.57750989	0.48530647	0.35508536
cg06422883	5	0.01789337	0.6587877	0.62942513	0.44156328
cg01240795	5	0.02041679	0.75101831	0.75746534	0.57838881
cg12656681	5	0.01670995	0.57679216	0.5902765	0.35598012
cg06159353	5	0.03528716	0.62886179	0.69905676	0.43316253
cg09553242	5	0.04378149	0.70600597	0.77328531	0.57579646
cg09835740	5	0.01356792	0.60209097	0.69845921	0.44734234
cg16667631	5	0.01234856	0.75558499	0.82402191	0.62329679
cg25113089	5	0.01097293	0.41320296	0.48023739	0.29620355
cg11176481	5	0.00703549	0.49637783	0.40946045	0.26815496
cg01558931	5	0.00768024	0.50272006	0.44310564	0.27660925
cg17598999	5	0.01618273	0.44014493	0.48954758	0.33593154
cg00462107	5	0.00631385	0.45182289	0.5284063	0.33609482
cg21046148	5	0.00853629	0.73500335	0.70464192	0.58375502
cg01025233	5	0.0103161	0.73300525	0.7728665	0.48496838
cg17573301	5	0.02823023	0.70387128	0.72990184	0.52082569
cg15499368	5	0.00509326	0.6257137	0.71159008	0.35413538
cg23440636	5	0.01485881	0.67367604	0.79244726	0.5043521
cg09160020	5	0.02457467	0.71983505	0.85167604	0.61845737
cg06647928	5	0.00262173	0.85051753	0.83960517	0.66540045
cg01117339	11	0.00146203	0.79305916	0.69997523	0.50442005
cg02608831	8	0.00085355	0.73048287	0.68084547	0.49169677
cg12032650	7	0.03472106	0.55944292	0.57219119	0.44142587
cg23361127	5	0.00866121	0.83817226	0.87711625	0.70255849
cg07904073	10	0.01110735	0.63502392	0.62593523	0.45293997
cg20544372	7	0.03736046	0.44557566	0.44326158	0.31196232
cg05207637	16	0.00463443	0.53633399	0.56741474	0.41252588
cg14508696	15	0.01163705	0.61391082	0.62405242	0.39744804
cg20116800	17	0.02385226	0.68821474	0.65886951	0.54858103
cg07343174	18	0.02151248	0.7131955	0.73628888	0.60353021

cg14747132	12	0.02343917	0.65092757	0.65351463	0.51205065
cg14343713	11	0.0043837	0.57659718	0.5691767	0.43396531
cg22780193	13	0.02483202	0.78698198	0.72732617	0.60357324
cg10900663	14	0.00182631	0.48266737	0.36431286	0.2517717
cg05796504	16	0.00806542	0.52231802	0.52378106	0.41613299
cg13308743	17	0.04482831	0.56821529	0.51873228	0.39384887
cg04184716	14	0.00988742	0.7067291	0.74279033	0.50339286
cg11597902	17	0.00248015	0.83904076	0.83563985	0.69515064
cg05850870	2	0.00086498	0.89228933	0.84431516	0.73274856
cg08858426	17	0.00117894	0.79107392	0.72697441	0.54972404
cg17472111	11	0.00243597	0.33294545	0.35875872	0.16972089
cg25738326	15	0.00403008	0.42312044	0.33952127	0.21915745
cg06825878	7	0.02009829	0.57725329	0.62037193	0.47532056
cg18720803	4	0.00456818	0.71089302	0.70031628	0.48399722
cg06829584	10	0.00279494	0.85450824	0.8305468	0.65367615
cg02138953	10	0.00081789	0.76945927	0.69518633	0.49007174
cg06677367	11	0.00065949	0.76085394	0.62879767	0.50624563
cg05209996	11	0.00754215	0.33730322	0.35877582	0.21433003
cg07770402	18	0.01203452	0.78781739	0.7597307	0.63016002
cg07817608	12	0.00100445	0.78697288	0.67014975	0.52244138
cg05762612	18	0.00436502	0.51981001	0.53241635	0.39580568
cg26556489	18	0.01141835	0.69545691	0.71048964	0.54797282
cg18537464	18	0.02735855	0.6376456	0.6342185	0.49045147
cg21348406	17	0.01294921	0.68803236	0.70641045	0.52009207
cg03884860	18	0.03824432	0.66725985	0.64798276	0.54631977
cg03411579	12	0.00081599	0.55994323	0.49574443	0.33153883
cg02072834	14	0.04180327	0.57174447	0.57134047	0.46016325
cg04797957	17	0.0286529	0.23976009	0.27704044	0.13964946
cg19413066	17	0.01373528	0.38570951	0.37905278	0.27213489
cg04759376	10	0.03738486	0.51937703	0.55743996	0.39828511
cg00901878	13	0.00197189	0.79437583	0.75524711	0.64462075
cg18413218	14	0.00372084	0.60393961	0.60409599	0.30789754
cg00213052	18	0.00398122	0.53905146	0.55257576	0.3100106
cg14453935	18	0.04580358	0.71289115	0.63544952	0.53360712
cg18817955	14	0.00910491	0.78246274	0.76092439	0.64227083
cg08324862	12	0.01245683	0.66113253	0.68298711	0.5577988
cg06822193	15	0.01145563	0.46790195	0.43635714	0.3174203
cg10259521	15	0.0122204	0.63959231	0.62097652	0.50505806
cg14579864	9	0.02911369	0.53906747	0.57772552	0.40211579
cg00835745	15	0.00505751	0.64566152	0.68266216	0.40880538
cg18659867	10	0.00420965	0.73031075	0.7008174	0.46675485
cg07013698	17	0.01957672	0.31458211	0.30962264	0.18752689
cg25175654	17	0.00245827	0.45239508	0.41549636	0.31306425
ch.10.80061816F	10	0.01690382	0.35617671	0.33388807	0.20866019
cg05317600	13	0.00099274	0.83758784	0.81050992	0.62127478
cg23346544	8	0.03931081	0.64895888	0.67107538	0.53580805

cg27449200	15	0.00194981	0.64580169	0.58806281	0.41919092
cg01940753	15	0.00440271	0.68087468	0.77151895	0.41750585
cg08783317	11	0.02009425	0.8177104	0.79928143	0.69798137
cg09533845	8	0.01317683	0.41846686	0.42671269	0.28623231
cg00602295	16	0.00485228	0.51891033	0.47872757	0.28647113
cg22812413	15	0.00796638	0.58103962	0.59882168	0.38996528
cg21807262	14	0.00065949	0.78273635	0.71405611	0.58513463
cg09318283	8	0.00752854	0.81143347	0.78944366	0.67135478
ch.3.82259654F	3	0.00100019	0.31408511	0.23969276	0.1185741
cg16534399	16	0.00509326	0.49877468	0.51083369	0.28290227
cg17391194	11	0.00208359	0.82130724	0.81769002	0.68605529
cg14212012	3	0.00150566	0.30342817	0.31399096	0.13692474
cg11125758	14	0.01866928	0.84701136	0.82969636	0.71489162
cg04528720	16	0.00084118	0.77689091	0.70639238	0.59461871
cg25132782	15	0.03699135	0.70607577	0.74990869	0.58202655
cg17765025	2	0.00521564	0.80103556	0.79785207	0.65084684
cg11955727	2	0.00854173	0.82374251	0.84444553	0.67938887
cg07726287	16	0.00768774	0.58639512	0.57750121	0.4510464
cg26645655	3	0.01734283	0.7528361	0.751351	0.63922202
cg01722566	16	0.0135118	0.7593114	0.71966372	0.58766183
cg15034300	2	0.00597656	0.31960756	0.32998741	0.21298524
cg16700163	16	0.00302287	0.69335604	0.62736297	0.36444975
cg02975243	16	0.00368022	0.76949379	0.74401984	0.63750667
cg12656085	2	0.01802754	0.55677583	0.59098138	0.3778667
cg02803141	16	0.00091059	0.88975616	0.85120119	0.69971657
cg02201914	4	0.00263648	0.49651974	0.50795801	0.27825387
cg23793104	4	0.0035878	0.68783749	0.68497156	0.40416061
cg03475509	4	0.01580766	0.66328694	0.66454086	0.46732944
cg03988540	4	0.00461358	0.63009986	0.59985518	0.39767311
cg22122321	4	0.00229687	0.75938446	0.7350839	0.53483763
cg13996610	4	0.00329066	0.79898114	0.73953656	0.62853756
cg01227239	4	0.01015703	0.45204036	0.48013789	0.30526783
cg08474859	4	0.00168532	0.57203028	0.54096829	0.34249896
cg00316839	4	0.0058315	0.61721451	0.58684758	0.42883585
cg22869030	4	0.00342408	0.48571412	0.47290029	0.30177841
cg07673740	4	0.00689691	0.53807013	0.56728748	0.30031373
cg11857320	4	0.00291825	0.56936915	0.5583047	0.3484213
cg04184019	4	0.00606663	0.39842831	0.38492976	0.23592169
cg03998835	6	0.02115579	0.52818119	0.51540143	0.36127732
cg10893095	6	0.0023961	0.53676212	0.5394764	0.32168694
cg09708616	6	0.00342811	0.38686791	0.40737621	0.228902
cg26299756	6	0.00648231	0.40317677	0.43891718	0.2587094
cg25560696	6	0.01670995	0.74369891	0.80183551	0.55165455
cg09832245	16	0.04010261	0.71293546	0.76385673	0.61233296
cg13536080	11	0.01042401	0.53476963	0.45242542	0.3349987
cg13718418	12	0.01580869	0.6542979	0.66159774	0.55197695

cg16596317	1	0.00236052	0.67739384	0.61876652	0.37794552
cg05792169	15	0.00233298	0.50241252	0.46301141	0.34339448
cg08428292	16	0.00141131	0.83737839	0.82664396	0.7081815
cg18802138	16	0.01887509	0.64047368	0.66134121	0.45116545
cg00335219	16	0.01432832	0.52775492	0.56509304	0.39355828
cg18019509	6	0.0037342	0.80157223	0.75576805	0.64201915
cg02502233	6	0.00520163	0.29634238	0.30068472	0.17156104
cg02637247	16	0.00273355	0.78278235	0.75824437	0.65322147
cg05626644	16	0.00117325	0.78507439	0.63997667	0.51925076
cg10332116	16	0.003807	0.39533093	0.40551996	0.2413191
cg17989763	10	0.00252483	0.80952953	0.82888367	0.68415819
cg16622061	16	0.0062271	0.70056142	0.68766599	0.51226273
cg01831404	5	0.02122541	0.81650741	0.80293932	0.70010659
cg24215812	10	0.02677197	0.5409437	0.54080363	0.4074868
cg09338148	16	0.00302287	0.37681615	0.30228667	0.17337825
cg04843568	5	0.01095648	0.32254823	0.364324	0.22030514
cg09247170	14	0.00244246	0.35476783	0.40840169	0.19855496
cg04041159	16	0.01390151	0.58285977	0.55651719	0.44906285
cg24406240	11	0.00878866	0.59486027	0.62192466	0.43278364
cg01413671	16	0.00144989	0.83223211	0.76941719	0.6569727
cg00318897	16	0.00118031	0.72124904	0.69417914	0.59158119
cg27464846	16	0.00198797	0.8393912	0.79737966	0.69057933
cg21494379	5	0.01376061	0.78922336	0.76409795	0.59772346
cg25414639	15	0.0023187	0.87731014	0.85473664	0.74781388
cg23598010	12	0.00065949	0.69372531	0.56468559	0.41728506
cg10172979	14	0.02627541	0.64875527	0.64593343	0.53061053
cg06531870	13	0.01946986	0.51507282	0.53108671	0.38457632
cg06183244	6	0.00463727	0.69550995	0.64802288	0.54675547
cg12177851	15	0.0194986	0.75338687	0.74033455	0.58514493
cg02984092	15	0.00547629	0.72645672	0.76531325	0.53240631
cg10513595	5	0.02720807	0.7816981	0.77472337	0.65724776
cg15547143	10	0.00459107	0.81710228	0.81035174	0.70094754
cg00172872	12	0.00113577	0.87611829	0.83658056	0.64172965
cg09884419	15	0.00099274	0.52512639	0.42678661	0.19862252
cg16846514	11	0.0440672	0.76145421	0.77339954	0.63948216
cg25430838	10	0.03204666	0.72099133	0.77124433	0.60646588
cg20801645	15	0.01887078	0.59136831	0.59546949	0.45592133
cg17083685	13	0.01092901	0.60968277	0.57629004	0.40364336
cg10255237	12	0.02382799	0.80515456	0.81926323	0.64252099
cg03618113	12	0.0065693	0.6920949	0.68430019	0.50331847
cg25946790	14	0.01264372	0.44188424	0.43185538	0.28317317
cg13410764	9	0.00087343	0.62956867	0.55103927	0.3609615
cg08447373	5	0.00119385	0.52458301	0.42502087	0.28137416
cg26404226	10	0.0012838	0.73817208	0.71396852	0.54750256
cg16598679	14	0.00081413	0.68303953	0.65035229	0.39810157
cg21946631	7	0.00149569	0.69839115	0.60431581	0.46050103

cg11465213	15	0.02338172	0.60550519	0.65388178	0.45103124
cg07786355	10	0.00168532	0.85192407	0.80740592	0.67976013
cg10108710	14	0.000777	0.89507278	0.8130878	0.7124052
cg00460983	10	0.00569156	0.75842901	0.79054336	0.6061606
cg07453778	6	0.00840828	0.4820262	0.5459665	0.3451237
cg16440978	14	0.00065949	0.62248664	0.35143491	0.1709459
cg10850823	5	0.02680775	0.59196952	0.61898503	0.46020862
cg14785064	2	0.03358212	0.52828417	0.55181991	0.41205417
cg04510459	1	0.00161582	0.63298318	0.61196809	0.41204235
cg04239548	1	0.00072814	0.85923948	0.78141871	0.67071722
cg25765315	12	0.00065949	0.83798587	0.74310323	0.49463258
cg18263166	7	0.0016785	0.50339661	0.46612245	0.22194804
cg11284842	5	0.02743413	0.53467912	0.513398	0.40933045
cg19429051	5	0.03274494	0.6770141	0.66170344	0.53509187
cg07533422	5	0.00873119	0.45977469	0.42914446	0.3289983
cg22658758	5	0.01260286	0.41727585	0.40238306	0.27423598
cg11670789	15	0.00245827	0.70948601	0.70592274	0.51813092
cg14358570	14	0.00639646	0.51016369	0.56615032	0.37777085
cg08584947	8	0.00146869	0.84591229	0.82012692	0.70842936
cg09564361	8	0.00207996	0.51683805	0.50538733	0.24334602
cg14488466	9	0.00084118	0.51916163	0.417196	0.17641044
cg21241675	9	0.0120439	0.62790126	0.6622103	0.4497792
cg13823964	9	0.00457701	0.53033257	0.51417931	0.3170883
cg02922879	1	0.00387834	0.46368457	0.40665691	0.29082037
cg16060189	10	0.02118965	0.62824936	0.67861283	0.52199532
cg25483123	12	0.0016785	0.81489053	0.77326448	0.6192296
cg12999267	12	0.00670577	0.32324789	0.32528163	0.20016265
cg08257009	14	0.00375204	0.70629954	0.66435027	0.50076636
cg27295434	1	0.0011159	0.87136251	0.85254166	0.72575594
cg07144720	3	0.01183547	0.49323834	0.4632694	0.34454717
cg00778190	1	0.01393327	0.60250467	0.63444714	0.42769756
cg24941342	11	0.00554761	0.79981713	0.81433624	0.61760193
cg09051342	8	0.00066232	0.68195716	0.54005978	0.3816988
cg25969107	15	0.00639697	0.55295912	0.61852162	0.3491385
cg06788267	1	0.0026101	0.72271517	0.72666219	0.44229566
cg18808466	13	0.00917702	0.81812214	0.83521764	0.70399034
cg06388350	4	0.00213077	0.37508395	0.34690554	0.16971019
cg20068510	15	0.00073118	0.89114825	0.83655198	0.72107556
cg25668093	15	0.01259427	0.74741574	0.82829831	0.61814712
cg12434362	5	0.01426642	0.75140493	0.77780226	0.65098947
cg23464619	13	0.03940048	0.53768261	0.60926873	0.42613978
cg13681496	9	0.0293309	0.4427162	0.44395159	0.33990698
cg04203453	8	0.01015703	0.38283179	0.4010005	0.25675566
cg16099687	12	0.01291157	0.72178081	0.72069495	0.51791828
cg23314893	5	0.00779924	0.54898821	0.57852903	0.36917332
cg07959469	15	0.03174945	0.72331508	0.74833817	0.61370138

cg17454263	15	0.00252984	0.64204477	0.68079941	0.43083495
cg05289920	15	0.03933893	0.78666108	0.79594942	0.64743378
cg00033666	15	0.0133181	0.77195499	0.82325945	0.66139233
cg17140992	15	0.01529615	0.60127496	0.62779795	0.43543851
cg27336587	15	0.01317469	0.77317833	0.79153723	0.66203738
cg15281710	15	0.01342633	0.81190477	0.82767789	0.68707888
cg21834679	15	0.01336583	0.73507664	0.78115016	0.61386401
cg01223086	15	0.00667367	0.57503957	0.62035653	0.39812042
cg25428740	2	0.00485896	0.41173286	0.42555254	0.25483201
cg06837325	5	0.00366179	0.82277872	0.83947109	0.72113027
cg27423669	13	0.01373528	0.60744746	0.5735406	0.46080249
cg04230579	11	0.01678972	0.88677777	0.89589099	0.78122174
cg21653474	6	0.00065949	0.57756916	0.48550376	0.36735006
cg10419556	8	0.01241725	0.49136406	0.45223722	0.33880362
cg18643563	14	0.00971274	0.54854499	0.54347777	0.39498177
cg14378231	1	0.00144359	0.5859378	0.47073971	0.32519277
cg03848421	10	0.0157632	0.40626211	0.40650071	0.27035974
cg12593746	1	0.00128378	0.4474163	0.4401457	0.1675325
cg27076113	6	0.00338778	0.27118552	0.30068472	0.16677556
cg03593014	10	0.01183547	0.58188137	0.56200234	0.37107375
cg03580065	10	0.03174689	0.60782714	0.61500251	0.49914566
cg15583285	6	0.00221105	0.35462303	0.3324677	0.19729645
cg02756683	10	0.04046492	0.73353224	0.74612897	0.59211946
cg17150809	6	0.0107931	0.80040764	0.82145956	0.69132828
cg13522075	9	0.0198367	0.52800049	0.45547756	0.35503727
cg06257058	7	0.01725665	0.54337615	0.54425312	0.4351882
cg20617957	13	0.00694471	0.41411022	0.43689035	0.2543211
cg20012607	13	0.01388202	0.33025023	0.38395727	0.2223052
cg04362790	14	0.00065949	0.66825736	0.42347734	0.30566584
cg07485423	4	0.00132756	0.49423327	0.41546085	0.24162378
cg15457481	4	0.00065949	0.8256089	0.73872025	0.5383072
cg05494668	11	0.00109839	0.72495192	0.64417684	0.52148999
cg04230891	6	0.00263672	0.42001258	0.45733487	0.26014397
cg01889956	6	0.00066232	0.46593328	0.33129669	0.20335221
cg10123952	3	0.03409516	0.69340865	0.67069559	0.55473435
cg01905090	1	0.00262173	0.82261825	0.81602153	0.69619154
cg14327296	14	0.00768774	0.3605661	0.38950272	0.25483863
cg02954563	14	0.00629716	0.44064617	0.43514199	0.30133572
cg03185794	13	0.00081928	0.78622218	0.72711196	0.53773727
cg26063904	1	0.01026117	0.79402606	0.79945204	0.6911889
cg14918548	2	0.028548	0.44463553	0.48045042	0.34055846
cg08702879	10	0.00189637	0.7079791	0.66484444	0.55050463
cg03312117	2	0.00631832	0.6671445	0.55608635	0.43106472
cg22909709	8	0.00805596	0.72843317	0.7295014	0.60037766
cg13106758	15	0.00211366	0.65025667	0.58735198	0.39612502
cg19009471	14	0.00520958	0.71523698	0.6694165	0.56770915

cg11667061	14	0.00537181	0.5758616	0.4504435	0.34354286
cg13758186	2	0.00155292	0.55305898	0.48056723	0.36805892
cg17654419	2	0.0008696	0.7926773	0.74169878	0.59134732
cg01771065	14	0.00664725	0.7689666	0.76311398	0.60448947
cg22202381	13	0.00504019	0.74284951	0.72259769	0.60405443
cg15840891	2	0.00474032	0.50516426	0.38122404	0.24406404
cg08461576	4	0.01365382	0.73115725	0.72469882	0.56304523
cg22022821	11	0.00078196	0.8797925	0.84692207	0.73239838
cg02775243	10	0.04127719	0.65667599	0.69163267	0.52700837
cg19928294	6	0.04832512	0.47646655	0.49418443	0.34977362
cg15128147	2	0.01711555	0.57821411	0.58367363	0.43249028
cg26269286	14	0.00396452	0.37341239	0.36489439	0.22706658
cg19589396	8	0.00112679	0.76589889	0.73959108	0.58107708
cg02987523	13	0.00758361	0.83974701	0.84129041	0.72402989
cg04481603	10	0.01268304	0.5534908	0.53453246	0.42470832
cg12950030	14	0.00135325	0.4469135	0.42681472	0.29514351
cg03022263	14	0.00127427	0.65036835	0.62221257	0.51208064
cg21833076	11	0.00648231	0.53167352	0.58114963	0.38410407
cg02495743	11	0.0009264	0.33321289	0.26666195	0.12449603
cg17812664	10	0.04866142	0.73372955	0.72850685	0.59103717
cg16759813	2	0.01398885	0.49367784	0.51361066	0.38994113
cg22865501	2	0.01579113	0.85429165	0.86160481	0.75284844
cg05979457	8	0.00616656	0.50169921	0.43625191	0.23709013
cg00998372	2	0.0037756	0.56471657	0.42274112	0.30786525
cg22928386	2	0.0157632	0.69079534	0.61820812	0.4756679
cg07316846	2	0.00437887	0.75433468	0.69813114	0.57962387
cg13077865	8	0.00714351	0.8352399	0.8358694	0.71858366
cg20788020	8	0.00069166	0.74188072	0.59579869	0.46694921
cg11111372	7	0.00283173	0.48160817	0.45014369	0.30940068
cg13155421	3	0.00071044	0.80180352	0.72635012	0.58932623
cg00100420	4	0.00168849	0.32234202	0.32860665	0.12699711
cg17947364	6	0.00065949	0.90308208	0.84444781	0.68932503
cg09477232	7	0.00279494	0.78181482	0.7668425	0.61203018
cg21557087	2	0.00073118	0.84894449	0.80161232	0.63984224
cg00139681	7	0.00405622	0.51411975	0.51665078	0.31110147
cg14387626	14	0.00820507	0.55402642	0.59548871	0.42453357
cg03875496	6	0.00127427	0.53747222	0.60139782	0.28646771
cg00688840	14	0.03174689	0.36455157	0.36619979	0.23857931
cg27576694	7	0.00193378	0.48457586	0.56604826	0.30678126
cg04686230	6	0.00481427	0.44020358	0.41905772	0.26752215
ch.2.105901354F	2	0.00092643	0.38898446	0.333906	0.17624217
cg17666418	6	0.01995197	0.68398996	0.69690293	0.49616481
cg06775939	14	0.0102143	0.39361036	0.45116256	0.28298154
cg17494199	13	0.00796638	0.74106563	0.7955771	0.61647113
cg04103847	10	0.03152563	0.69989609	0.70518476	0.59660804
cg07368069	12	0.00320053	0.2797448	0.29397578	0.16803577

cg16479476	6	0.00065949	0.83461875	0.69976574	0.5627134
cg08950364	1	0.02445578	0.59210697	0.66083207	0.40524267
cg21186098	8	0.00244246	0.45643245	0.52727348	0.28362254
cg00951857	6	0.02004883	0.58567253	0.64809078	0.46206979
cg08707476	1	0.01052554	0.70565918	0.72501003	0.59498224
cg01950077	6	0.04423676	0.66703941	0.64091465	0.52726219
cg04064998	11	0.02833301	0.4158116	0.4125672	0.24663828
cg12894371	6	0.0066172	0.5948488	0.59319975	0.45320458
cg20608895	13	0.01024091	0.66310564	0.62544149	0.46921278
cg02489769	12	0.00628685	0.76435951	0.76258819	0.64726052
cg00556100	1	0.00516729	0.78338151	0.72786266	0.59478529
cg05132925	1	0.00314361	0.64547348	0.63615516	0.46365507
cg01733904	13	0.00417561	0.76640873	0.79691335	0.58881051
cg21197796	9	0.00077468	0.68381846	0.59255306	0.47381069
cg22006640	4	0.00084594	0.32567853	0.32375123	0.20758251
cg13891768	4	0.00958999	0.31001335	0.28048854	0.17171969
cg04676482	4	0.00227742	0.46507358	0.46404703	0.33999927
cg05492170	4	0.00227742	0.35419758	0.37021147	0.19248075
cg11761728	4	0.00570747	0.42503646	0.46671877	0.31925416
cg19849728	4	0.00124853	0.26632085	0.27935632	0.12489094
cg23646776	4	0.00093631	0.2305769	0.24367099	0.09936137
cg07790170	4	0.00182655	0.32886786	0.34929728	0.13367709
cg03722396	4	0.00290589	0.41289876	0.45608017	0.20594349
cg16041686	4	0.00177922	0.36901379	0.37035498	0.22086263
cg01733176	4	0.01975439	0.29848151	0.31348277	0.18941256
cg17242937	4	0.00160662	0.49460315	0.50704009	0.28119037
cg16933229	4	0.00334886	0.52025757	0.49058987	0.31165167
cg03133735	4	0.00330119	0.48140917	0.51103693	0.27985468
cg11671598	4	0.00456344	0.57311205	0.62128611	0.45586078
cg13993197	1	0.00081925	0.58427579	0.51212152	0.38122556
cg03171003	2	0.00196334	0.34529339	0.34008372	0.20701086
cg24617444	10	0.01309134	0.42871651	0.450334	0.29289295
cg03548415	11	0.02445578	0.40479649	0.38217396	0.26918736
cg00035969	1	0.02157743	0.68927029	0.71521467	0.56829074
cg01269299	10	0.00557338	0.79577188	0.77168227	0.6619866
cg22976832	10	0.00314623	0.25023525	0.27294461	0.13737497
cg02655361	3	0.00161582	0.77258401	0.73951017	0.57428656
cg06685282	3	0.01944702	0.41386268	0.50588757	0.29850364
cg09662304	13	0.01463398	0.58242986	0.66075147	0.47391088
cg26117369	13	0.01616965	0.57049684	0.57945132	0.41918617
cg22378095	6	0.00103895	0.68423294	0.61851985	0.49154934
cg03705366	5	0.02944061	0.61255515	0.63132493	0.51085453
cg20948409	3	0.01096214	0.4908929	0.49214338	0.38459987
cg19186056	11	0.0439181	0.72429697	0.79596316	0.62342548
cg11612799	3	0.04565804	0.65622047	0.62490501	0.48916736
cg05117638	6	0.01776628	0.85146193	0.86891683	0.71279559

cg00066854	10	0.02146686	0.79732376	0.84306673	0.637913
cg24103651	10	0.00804549	0.80673066	0.81963882	0.61092699
cg09440866	12	0.01484626	0.36911651	0.3641265	0.23858675
cg15274309	11	0.00089205	0.87126913	0.84268628	0.72738244
cg04452203	4	0.01465773	0.29458679	0.27747824	0.16318081
cg00437124	6	0.00430896	0.58538908	0.64075746	0.4069687
cg03972891	2	0.00085355	0.86179226	0.83180866	0.72009056
cg03254336	10	0.00065949	0.45727325	0.30827208	0.18653551
cg11611580	6	0.01040141	0.50266409	0.48119391	0.37112132
cg10233830	12	0.00539451	0.43500669	0.3999641	0.22412925
cg24116380	5	0.0011414	0.79505176	0.7814884	0.64895317
cg02637031	5	0.00248044	0.47249286	0.45377861	0.31722601
cg02136690	13	0.00499353	0.35427558	0.34633374	0.21885151
cg23739746	13	0.00585298	0.42027067	0.41531121	0.26364145
cg25888386	10	0.00816896	0.70520937	0.77445999	0.56834995
cg20799176	11	0.00826879	0.43238042	0.41028576	0.26023441
cg18408273	4	0.0101333	0.56217099	0.63249313	0.45726631
cg14855679	4	0.00329066	0.54091382	0.61928601	0.42738802
cg25747670	1	0.00096242	0.73236375	0.68262949	0.49803798
cg16550264	12	0.00486966	0.55192874	0.55534248	0.38673046
cg16893861	7	0.02457497	0.62904665	0.61964993	0.50547898
cg21584846	1	0.01183547	0.76253787	0.76042005	0.55857723
cg19442415	11	0.0035878	0.33214707	0.33610589	0.23061102
cg12136387	12	0.0051099	0.31225398	0.28676291	0.18528334
cg13017929	7	0.00085355	0.73453816	0.63065399	0.52099
cg00960147	1	0.00159601	0.51543983	0.4603386	0.27603496
cg14113958	9	0.01768314	0.77662787	0.76403672	0.62715842
cg22795331	6	0.00179209	0.85857683	0.82245311	0.71289104
cg13838276	6	0.03020815	0.29555716	0.33684634	0.18156971
cg11958234	1	0.00066232	0.90499492	0.84534239	0.70948294
cg07301829	8	0.01015464	0.71134398	0.67980558	0.54279571
cg25916172	10	0.00111057	0.5310044	0.52254138	0.42240276
cg21308365	6	0.00224846	0.57901354	0.49696797	0.27804349
cg06169746	3	0.00327239	0.76674087	0.74849655	0.58128598
cg04214938	2	0.0039383	0.62671184	0.58366984	0.46401389
cg12482501	10	0.0352175	0.73104317	0.70794969	0.59286181
cg21301805	1	0.00277682	0.43901956	0.43892098	0.25152992
cg26570165	1	0.00646891	0.64375803	0.60691203	0.41819047
cg00667647	1	0.01997754	0.73546375	0.68699165	0.52031497
cg26856924	1	0.01452282	0.69913318	0.68336021	0.46121046
cg12659883	1	0.01679702	0.66050397	0.63031995	0.43039101
cg02063759	1	0.01515174	0.66620722	0.62135444	0.42818596
cg01439876	1	0.04388834	0.67622823	0.67403988	0.54890855
cg17479943	1	0.00427027	0.69119794	0.686648	0.49601438
cg24053992	1	0.00441677	0.74730461	0.71362465	0.59928797
cg01994190	2	0.0063774	0.42953887	0.47317416	0.30891847

cg07080244	11	0.01467615	0.5170099	0.49546707	0.36373574
cg07081245	11	0.00066232	0.67471348	0.52268118	0.40906767
cg19318537	11	0.00311025	0.3854662	0.38181755	0.24509117
cg26123824	10	0.00065949	0.73493078	0.6303359	0.46978967
cg26671477	1	0.03342972	0.79196445	0.82579367	0.65208857
cg02601475	5	0.02089413	0.69894952	0.69780894	0.53022636
cg24298578	6	0.01806244	0.76596007	0.68979368	0.58925815
cg26196626	8	0.00110703	0.52841594	0.43894989	0.26726222
cg04844574	12	0.00357923	0.23997792	0.2433746	0.10654095
cg13988455	10	0.00098208	0.65930909	0.53487697	0.41303044
cg01434160	5	0.00166476	0.48457313	0.5015849	0.23963944
cg18829827	5	0.00620953	0.68487512	0.70031689	0.57027474
cg04285666	5	0.00458992	0.73775969	0.74166243	0.50908423
cg14698448	5	0.01935097	0.70872585	0.69763294	0.57576572
cg01250603	10	0.01643293	0.73217213	0.8001986	0.59782623
cg01829632	5	0.01390012	0.60561389	0.62115575	0.49647631
cg27365991	5	0.0010058	0.63621564	0.61306756	0.37028864
cg07874386	5	0.00349176	0.72035219	0.69861712	0.57176522
cg15083851	7	0.01580038	0.56277309	0.53938573	0.41213866
cg24623760	12	0.00156239	0.85355601	0.835624	0.64539451
cg14250450	10	0.02522711	0.5652636	0.58379111	0.45151704
cg08639279	11	0.00113577	0.88792036	0.86352054	0.72989508
cg10840277	8	0.00971058	0.77004221	0.79407866	0.63156874
cg03129324	10	0.00606663	0.60898808	0.59867811	0.35546904
cg15624719	4	0.00438281	0.60624245	0.5151428	0.40144136
cg14397231	4	0.04698917	0.37271383	0.39550239	0.27024978
cg07243762	12	0.00117201	0.72744475	0.64963602	0.53481192
cg14503489	10	0.02358538	0.59075179	0.60241382	0.48358491
cg26780125	8	0.0107931	0.63184814	0.67238479	0.44565467
cg20945764	3	0.00901377	0.53482283	0.57089771	0.4188187
cg16657152	8	0.00849487	0.46326272	0.51840932	0.2998597
cg26821681	8	0.00287555	0.48414	0.47464003	0.25608279
cg02612397	6	0.01614766	0.46314844	0.5092237	0.35317768
cg16945415	3	0.00967401	0.58416944	0.58446096	0.41347863
cg17389956	12	0.00243597	0.32206584	0.29343255	0.17944683
cg08913523	8	0.00118039	0.65626017	0.55988584	0.31859393
cg17534999	11	0.03515143	0.61474163	0.61036988	0.4802079
cg16652303	11	0.01317021	0.60631944	0.61758598	0.41901386
cg17273911	11	0.01689306	0.48105422	0.50985762	0.36075932
cg17888985	10	0.00375204	0.3638789	0.34213982	0.20672151
cg20143985	10	0.01145563	0.47554952	0.44624317	0.34443746
cg19526685	8	0.01933502	0.53204622	0.56401675	0.33868087
cg14213105	9	0.02917135	0.67270024	0.68363302	0.52160246
cg20708282	5	0.00715821	0.75732254	0.7317392	0.57615508
cg12495090	2	0.03946158	0.36315869	0.35067559	0.22833453
cg16723381	2	0.01616965	0.45276914	0.51434086	0.34901674

cg16578085	4	0.00084118	0.71792112	0.65219393	0.39978787
cg27228272	2	0.00578063	0.55594673	0.50077426	0.39664061
cg01495299	8	0.02492359	0.72326121	0.76172106	0.58595439
cg18334977	3	0.00142756	0.77168753	0.74505352	0.56165564
cg17709804	11	0.04539328	0.73034354	0.74409995	0.62664564
cg25607177	11	0.03710592	0.68230413	0.70558966	0.55067213
cg07318609	10	0.025223	0.50696595	0.57581314	0.40537964
cg22950831	10	0.00073118	0.87594239	0.73970112	0.59886534
cg17588094	8	0.00381594	0.8037784	0.80272477	0.66458509
cg22881265	3	0.03659438	0.47145826	0.48219233	0.34783346
cg20475082	2	0.01235814	0.61352193	0.61013195	0.4560283
cg15331705	8	0.00763406	0.46502318	0.44563979	0.24904889
cg04795044	3	0.02715108	0.87259983	0.85230725	0.75147469
cg23229770	2	0.00857669	0.63707057	0.6615475	0.46230605
cg04998379	4	0.01506509	0.66780406	0.71334709	0.5255353
cg17477578	8	0.00079512	0.68811645	0.53754439	0.35699097
cg16434510	8	0.03052319	0.73096985	0.68864995	0.56782074
cg09187338	7	0.01130304	0.56309377	0.53927769	0.39994933
cg00228735	7	0.03009647	0.62108489	0.65895548	0.51124038
cg21211664	9	0.02190777	0.72446774	0.7596721	0.59863359
cg21551253	12	0.00538904	0.59432883	0.62195889	0.48089516
cg11225089	5	0.00223182	0.73217028	0.68095785	0.52472408
cg01645729	2	0.00233734	0.43806699	0.45876962	0.20765743
cg13363575	2	0.0108906	0.42407712	0.68107603	0.57250692
cg05189730	10	0.00281199	0.62713708	0.63922153	0.49917906
cg11161597	12	0.00428303	0.64911464	0.65184217	0.51304803
cg11367260	10	0.03477069	0.6136375	0.636625	0.50593418
cg01577029	12	0.00233298	0.64060804	0.57394607	0.47271347
cg08634598	12	0.03929291	0.54179256	0.50966546	0.40671266
cg00792107	12	0.02549971	0.4860534	0.50492136	0.38302095
cg07095347	6	0.00796332	0.49125188	0.48525609	0.30850497
cg06455180	10	0.01485881	0.66173173	0.65911541	0.55838408
cg01342115	3	0.03488893	0.40444067	0.39710838	0.29628111
cg24686551	3	0.00703549	0.77107953	0.74599563	0.52964033
cg05810177	7	0.00355472	0.76958029	0.76478348	0.62241742
cg16347123	7	0.00247734	0.36453677	0.34911507	0.23516502
cg19549902	7	0.00219711	0.87317128	0.88439827	0.74047306
cg16408820	8	0.00070678	0.90032007	0.83002407	0.71074551
cg14031473	9	0.01580766	0.51759286	0.52700244	0.37423645
cg07243202	8	0.0008947	0.61079537	0.5220957	0.31853464
cg01830023	5	0.00358374	0.57656964	0.56931054	0.34039688
cg15128679	5	0.00143343	0.26166431	0.23467089	0.1267986
cg22192489	5	0.00127413	0.60005367	0.55071517	0.26613957
cg14671011	5	0.00177922	0.775684	0.7427566	0.54402423
cg02104392	2	0.00081789	0.84037707	0.79890275	0.65548542
cg24822529	5	0.00065949	0.544361	0.33594126	0.210375

cg02395454	11	0.00290723	0.40355086	0.42461763	0.27721898
cg18765405	5	0.00100561	0.76248377	0.70580663	0.54080227
cg22716280	10	0.0048909	0.54517671	0.53702087	0.43401102
cg01384319	2	0.00772025	0.71105593	0.76286612	0.59663733
cg26922854	6	0.02106058	0.58965999	0.55981838	0.44838267
cg16792071	10	0.00109306	0.48788535	0.46385251	0.35968894
cg02314201	10	0.00345314	0.64792625	0.77998557	0.52618499
cg08267250	10	0.00104158	0.30170073	0.27280444	0.13909775
cg03183618	2	0.00101054	0.71980343	0.64245981	0.52784393
cg02142926	6	0.00207423	0.573971	0.50480824	0.33472154
cg16001689	6	0.00513872	0.58355662	0.55407557	0.32776323
cg17051543	10	0.03434255	0.52018698	0.55064894	0.40312645
cg09932730	7	0.01340049	0.44370521	0.40103525	0.28355845
cg18914751	5	0.00165724	0.85108693	0.79443333	0.69060169
cg01185573	3	0.00372084	0.64631031	0.64142023	0.53260499
cg13375466	8	0.01494119	0.45623676	0.66840176	0.56777576
cg07217824	7	0.00425638	0.54622375	0.62067011	0.35314547
cg25398315	8	0.00336153	0.52853751	0.54312817	0.40298738
cg19350115	6	0.01398104	0.8174851	0.78451389	0.6726589
cg00688895	7	0.00857662	0.37335012	0.37361947	0.26749354
cg11678250	7	0.01019185	0.69811674	0.69131135	0.52802164
cg13638282	9	0.00065949	0.82422769	0.6974666	0.55227864
cg00927256	2	0.00552206	0.71554125	0.72388146	0.58067417
cg25388800	5	0.00405622	0.51679208	0.48348516	0.21519368
cg20470975	6	0.01760238	0.70674542	0.73565522	0.60302788
cg11013714	3	0.00066232	0.82131192	0.74343506	0.63712097
cg13688783	9	0.00769304	0.50792011	0.52446481	0.40720884
cg08832234	8	0.00109888	0.62061966	0.63165943	0.5088875
cg27200630	3	0.01975439	0.46214357	0.4604085	0.31687788
cg15649384	2	0.02214521	0.70965345	0.71912423	0.59516909
cg07708521	5	0.00217555	0.84152513	0.74913111	0.63058834
cg22642485	5	0.01096175	0.55631458	0.52969953	0.33774226
cg07285237	6	0.00065949	0.72045721	0.6166704	0.36548106
cg14223856	9	0.00076471	0.75249404	0.66337094	0.5380302
cg14462040	9	0.04256022	0.65243994	0.67831728	0.53926266
cg08719642	9	0.01468727	0.43883707	0.47590576	0.33810738
cg19987129	6	0.04539328	0.72354147	0.71827436	0.60613236
cg05219445	6	0.02155924	0.50052283	0.55638044	0.37865368
cg14534848	9	0.00485228	0.24292866	0.25296105	0.1326648
cg15812586	7	0.00113577	0.73457983	0.64033561	0.35819939
cg13306815	6	0.00065949	0.86387318	0.76436334	0.65279505
cg12895857	6	0.00081599	0.80184411	0.71613398	0.60580577
cg23500924	5	0.04902903	0.39321959	0.38958409	0.27679994
cg26994377	4	0.00150458	0.34798043	0.27935558	0.15885382
cg14699112	4	0.00173267	0.80460489	0.8573036	0.69742986
cg05979766	5	0.00112828	0.43103048	0.32015624	0.15458774

cg22929787	5	0.00156239	0.4844725	0.42425352	0.25953288
cg21550107	5	0.00828598	0.66437655	0.67256896	0.53479527
cg09599062	3	0.01057629	0.65165316	0.63753856	0.48110386
cg22822656	8	0.01384716	0.34701616	0.35213954	0.2037131
cg07271186	7	0.01378835	0.4122685	0.42128573	0.28970843
cg11324467	8	0.00805596	0.64906959	0.58500394	0.46177672
cg26283879	8	0.04319217	0.69444411	0.69373326	0.54544844
cg20203768	8	0.00125711	0.49946311	0.50450994	0.39941168
cg21200085	1	0.02743413	0.38127029	0.40139264	0.27754057
cg19145082	3	0.00130462	0.34586848	0.2976081	0.18126085
cg17796982	8	0.01890283	0.71483107	0.71899508	0.58269502
cg10755035	8	0.01317683	0.38059492	0.38109501	0.26753814
cg18198461	8	0.00119376	0.40202414	0.39872666	0.2714438
cg12192282	2	0.00132036	0.66040055	0.62357875	0.38786872
cg04035553	8	0.00287555	0.48623054	0.4502742	0.28067296
cg01771479	8	0.01825754	0.5881837	0.62565005	0.47719373
cg17779002	8	0.01305145	0.6342185	0.64707907	0.4759964
cg04021592	2	0.01907154	0.37062956	0.33307634	0.21662237
cg19266329	1	0.00248044	0.57058519	0.54278907	0.37358383
cg02463513	3	0.0011104	0.91171013	0.88450895	0.77551185
cg26905281	8	0.02169667	0.43545328	0.44740734	0.32817711
cg20724214	3	0.02122541	0.32868799	0.33588173	0.22290906
cg25503888	3	0.02463599	0.40871309	0.44497695	0.30849002
cg09120877	1	0.00670577	0.52245889	0.5571225	0.38520474
cg20168495	4	0.00211366	0.82957524	0.80061501	0.65723678
cg01903799	4	0.00591291	0.67787369	0.68469493	0.50890737
cg16247269	5	0.00318676	0.61033723	0.60233527	0.49521388
cg03772020	6	0.00194312	0.78549837	0.7714611	0.41131684
cg09714315	2	0.03265907	0.68525304	0.75743788	0.56778157
cg11603406	7	0.00913085	0.57511915	0.57734064	0.47411277
cg26613811	7	0.00593649	0.43703517	0.51086591	0.28011757
cg01195545	7	0.01736212	0.6493477	0.67559716	0.48340661
cg12810503	7	0.02521286	0.57428751	0.59658093	0.44242365
cg00252472	6	0.03221861	0.40910944	0.44305805	0.27793251
cg24085232	5	0.00663444	0.63218031	0.64984758	0.53032284
cg04134096	3	0.00067859	0.75270036	0.67976993	0.56023396
cg07201475	2	0.00832752	0.64428432	0.55951323	0.41530339
cg08529744	1	0.00098204	0.6327911	0.58636129	0.46103296
cg07092896	2	0.00471011	0.57401881	0.62309128	0.45261804
cg08737338	2	0.0042395	0.72993318	0.72534342	0.58295415
cg07089056	3	0.00548311	0.35846759	0.38480864	0.24052549
cg10132704	7	0.00593649	0.7309637	0.77055964	0.61243342
cg21502476	3	0.04243997	0.4747159	0.48138372	0.33888265
cg26875626	4	0.04879812	0.77334248	0.81628622	0.66526639
cg00399683	7	0.01580766	0.82717859	0.83125223	0.68949332
cg05662444	5	0.01506509	0.59527833	0.65654945	0.46100344

cg16295461	4	0.00352201	0.63990314	0.59079011	0.46240251
cg00786237	3	0.00468024	0.63295188	0.59645679	0.48159605
cg03252770	4	0.00283173	0.50517779	0.4612502	0.27647177
cg00168634	4	0.01093903	0.44124714	0.41918623	0.2728709
cg08894020	5	0.00651383	0.57345993	0.65715413	0.46076332
cg25102742	7	0.02830718	0.63442371	0.66285076	0.4963754
cg00096536	4	0.02233892	0.43114943	0.40534259	0.25341198
cg19748027	5	0.00579556	0.70718375	0.75465011	0.56984174
cg06361606	1	0.00075494	0.4716409	0.35375382	0.16549667
cg03831405	7	0.00912728	0.69708962	0.68572347	0.57631584
cg23697406	7	0.00139783	0.85008741	0.80672153	0.68007925
cg03573168	7	0.01276611	0.49474883	0.50031257	0.39416319
cg20685713	1	0.00183809	0.61404937	0.58270965	0.43811552
cg25343618	1	0.00512138	0.37999247	0.29080326	0.18978631
cg05612608	3	0.01956069	0.72410065	0.72906967	0.59017735
cg00345744	7	0.03241445	0.51467383	0.49355758	0.36535602
cg15688980	3	0.0008696	0.82354236	0.76545762	0.66411474
cg26448394	4	0.01031494	0.49349406	0.48799803	0.31510246
cg25024214	1	0.00118326	0.75051533	0.74523821	0.62188845
cg25028404	1	0.00444287	0.6769938	0.6694228	0.50417775
cg25252017	6	0.00117894	0.86087875	0.83794039	0.73090951
cg11763066	6	0.00749271	0.3785448	0.35061827	0.21760342
cg16223546	5	0.00260155	0.66062307	0.69598776	0.41687887
cg01068621	1	0.00109306	0.88235635	0.86313643	0.73624921
cg04500909	5	0.00075494	0.879225	0.81870809	0.68141942
cg00975624	3	0.00094083	0.66346253	0.59138944	0.34339672
cg14217495	1	0.01314425	0.58808641	0.60745539	0.43260736
cg17033891	3	0.00170239	0.68322077	0.67112319	0.36479158
cg05581878	3	0.00132756	0.72721162	0.6014133	0.45201231
cg02682520	5	0.00608343	0.29884575	0.33364214	0.17481647
cg13423054	3	0.0011159	0.82417068	0.7730298	0.65980508
cg21832142	3	0.00072814	0.47199109	0.35628294	0.2206262
cg21758140	2	0.01126796	0.45685957	0.48072642	0.32497265
cg24525138	3	0.01783758	0.59936236	0.6026519	0.48180137
cg25831439	2	0.01884397	0.61230237	0.63191815	0.43639749
cg11141696	1	0.00189637	0.86721404	0.86771915	0.75277729
cg27227317	2	0.00078196	0.60921632	0.56946812	0.3542779
cg12341429	2	0.00065949	0.69224418	0.53788002	0.43379885
cg02104138	5	0.03954748	0.67276405	0.69380328	0.52403949
cg26338202	6	0.04291975	0.58614118	0.54993656	0.40876028
cg20867674	6	0.00912728	0.54998498	0.54836421	0.39254504
cg08510178	6	0.00670817	0.48823545	0.50906003	0.34919234
cg16721489	2	0.00159601	0.64428151	0.60837503	0.46320511
cg06500090	6	0.01332867	0.73622956	0.72200931	0.58221113
cg00386035	6	0.02042436	0.8297058	0.84061598	0.7013696
cg00121876	6	0.00383861	0.85424369	0.83057962	0.68687382

cg27325820	5	0.0103956	0.75022822	0.68844915	0.5448872
cg16383005	6	0.00213077	0.61208514	0.58233767	0.47521791
cg26954951	6	0.01206219	0.66635799	0.676543	0.50161171
cg19445758	6	0.00844494	0.62041429	0.60163189	0.43079537
cg11942594	3	0.0132124	0.796172	0.82188759	0.64384548
cg04728978	2	0.00849487	0.67475565	0.72539157	0.57038556
cg20432671	1	0.01783758	0.52985161	0.52455513	0.37402043
cg12708109	1	0.00780052	0.50193406	0.55738568	0.38292969
cg01813280	6	0.0118001	0.70108734	0.78276471	0.53931845
cg08406698	6	0.048539	0.67758195	0.65514284	0.54009166
cg21002542	6	0.01344244	0.7398416	0.70593874	0.59455097
cg24104268	6	0.0102099	0.50231119	0.53506809	0.38334142
cg14580747	3	0.03204666	0.42973526	0.46510856	0.28884183
cg10703884	3	0.00930663	0.37885879	0.43694149	0.25311231
cg22453826	6	0.00065949	0.70057729	0.60032922	0.49378493
cg06853836	6	0.00106261	0.60390927	0.52754536	0.41663465
cg24939196	6	0.00104158	0.57555821	0.53414116	0.3067785
cg08866665	6	0.00367708	0.51648609	0.49221766	0.32785842
cg23143090	6	0.00155548	0.56666647	0.58563082	0.36189369
cg22006060	4	0.00146376	0.3182171	0.30020005	0.12918092
cg04398893	6	0.00077468	0.87602033	0.81255095	0.69404062
cg05935904	6	0.01019185	0.55251938	0.55044136	0.35406627
cg01766850	6	0.02435054	0.5333833	0.57399908	0.36837356
cg02360002	6	0.00855749	0.80985181	0.81428665	0.68655258
cg12439157	1	0.03860148	0.80403632	0.80139954	0.69523505
cg03292222	5	0.02616513	0.40604039	0.4684287	0.30398644
cg01423389	3	0.00113188	0.65972702	0.67093939	0.54603136
cg13945880	5	0.00065949	0.86061181	0.8003082	0.6597249
cg05468458	5	0.00077361	0.80749763	0.7358378	0.59974532
cg20952257	5	0.02357584	0.44567045	0.39542582	0.28311496
cg24114813	5	0.00857076	0.77583212	0.79190972	0.62675969
cg01905967	5	0.0012838	0.58431525	0.54041054	0.35211096
cg24465329	2	0.00642316	0.58106615	0.53836243	0.43094475
cg11079129	5	0.02099588	0.39450731	0.4266913	0.29033278
cg05234035	5	0.01050457	0.63720268	0.67018703	0.52006541
cg13313423	5	0.02658729	0.72231141	0.71398753	0.58259125
cg00321614	5	0.01533394	0.37713497	0.40971035	0.26339087
cg26473548	5	0.00112679	0.44603854	0.41879409	0.26896703
cg14746387	5	0.01492665	0.81456803	0.8591458	0.70906096
cg18234224	1	0.00083771	0.90455712	0.87768793	0.71797255
cg26355232	2	0.01359116	0.72875858	0.72790872	0.62509779
cg05871756	5	0.00260155	0.53050769	0.46883935	0.31330197
cg23091549	1	0.00181052	0.76218097	0.69894551	0.59644958
cg11470941	4	0.03832563	0.63004013	0.61699778	0.47000955
cg02307239	4	0.01807343	0.3981008	0.40579802	0.29637868
cg04840702	4	0.01422483	0.77702115	0.79499752	0.66928409

cg13401079	4	0.00381021	0.47674274	0.48152627	0.3078207
cg24530432	4	0.0113406	0.61982547	0.61269922	0.50405247
cg00070383	4	0.00576931	0.56395412	0.67834903	0.46079997
cg05191919	5	0.01183547	0.38929118	0.3504646	0.21684116
cg26113233	5	0.00065949	0.71145727	0.61379271	0.45762365
cg19807286	5	0.00186784	0.84408818	0.81699895	0.69334706
cg10256255	2	0.00129726	0.8694677	0.88540625	0.7420845
cg06609094	4	0.02466633	0.35271869	0.41282204	0.24923684
cg07240834	4	0.00913085	0.26625712	0.27700129	0.16306063
cg19867579	2	0.0009934	0.63548832	0.51986698	0.33590458
cg18526008	3	0.00973492	0.47042208	0.51048584	0.30244801
cg18475969	3	0.01276611	0.31367135	0.33096222	0.20857653
cg13401658	2	0.00420024	0.84826139	0.8053365	0.68223
cg07484739	2	0.00065949	0.76857874	0.5877745	0.38722572
cg06065019	2	0.00065949	0.41998578	0.31816852	0.1931315
cg24045357	2	0.00076043	0.62908407	0.56538647	0.31282857
cg10606620	4	0.00117894	0.86245911	0.82067038	0.69827701
cg09264231	1	0.02514684	0.61990706	0.63578471	0.44253002
cg16346032	1	0.02265546	0.7047913	0.692595	0.52700625
cg08943293	1	0.02711474	0.72740621	0.73567405	0.58200823
cg04446653	3	0.00244887	0.36219139	0.41701954	0.20284027
cg17126947	4	0.00255774	0.79793006	0.78737804	0.60907185
cg23170646	4	0.01293322	0.46161981	0.49611815	0.31471509
cg13612936	2	0.00441677	0.31917227	0.34430655	0.21686706
cg22293458	3	0.00531964	0.47896587	0.54372919	0.33604172
cg04657325	4	0.01665976	0.23785773	0.24869204	0.13657243
cg18518916	4	0.03020815	0.70611797	0.75346565	0.59769139
cg14054279	3	0.00065949	0.74479604	0.63688412	0.49212074
cg20965866	4	0.00325324	0.8580232	0.85991458	0.74786932
cg04156889	3	0.00260306	0.27585925	0.25259265	0.13709018
cg10168494	3	0.01870855	0.67127261	0.68283961	0.52442087
cg13427361	4	0.0160141	0.67502343	0.64695318	0.47115088
cg03466587	3	0.00243686	0.3848916	0.2879519	0.1775529
cg12307200	3	0.00182941	0.55413772	0.43316208	0.32696538
cg04810745	2	0.0011159	0.84741863	0.75192332	0.62725659
cg16652387	4	0.03960534	0.74611591	0.73273389	0.59792492
cg27621721	3	0.00065949	0.66569452	0.51540173	0.33460096
cg25568241	3	0.00655853	0.64849762	0.58185446	0.48118847
cg12591668	1	0.00237543	0.80076991	0.7326863	0.57758452
cg07779313	2	0.02265145	0.56113438	0.52725536	0.42114173
cg23542902	3	0.00076912	0.41328339	0.31850527	0.17743866
cg23192683	3	0.03087305	0.68894262	0.67396058	0.54768553
cg13905238	3	0.00726745	0.51310001	0.53138721	0.34330906
cg14653136	1	0.00318831	0.37402319	0.43252264	0.24341373
cg00151251	2	0.0122594	0.4142203	0.45888147	0.30143913
cg09517106	1	0.01488167	0.45699468	0.45801312	0.28181032

cg26257082	1	0.01580766	0.30386558	0.30758397	0.15657457
cg27058077	1	0.00686574	0.64852363	0.63667851	0.48587843
cg15647364	1	0.0438072	0.46123595	0.50076977	0.33858749
cg12082516	1	0.00462871	0.67445877	0.64289772	0.51704015
cg22534374	1	0.00068904	0.80346787	0.65386327	0.44790417
cg00387090	1	0.01533364	0.68188914	0.676107	0.47264041
cg17836014	2	0.02348349	0.78247864	0.75732275	0.654301
cg06215984	1	0.00857662	0.47904959	0.48214907	0.28935356
cg12801030	1	0.01096356	0.6716114	0.66933691	0.50354757
cg11786870	1	0.0074197	0.7204002	0.76306632	0.53488739
cg02736228	1	0.01866928	0.6259207	0.6119299	0.50812863
cg09030187	1	0.0012504	0.65230652	0.59611953	0.44003228
cg16308270	1	0.00085355	0.46272164	0.38062993	0.24067775
cg13052638	1	0.00065949	0.71872949	0.59460272	0.40979903
cg02338840	2	0.00183659	0.78997479	0.78366784	0.62870954
cg05956608	1	0.03120023	0.6241422	0.57593696	0.46162468
cg13857933	1	0.0084821	0.4158013	0.42346427	0.23919734
cg15749661	2	0.0046036	0.73362187	0.69154817	0.59103864
cg10440877	2	0.00520958	0.65352628	0.70178502	0.51199405
cg19479609	2	0.00857076	0.47668799	0.49739006	0.31855144
cg22787719	1	0.02215556	0.56792682	0.56024381	0.42686374
cg12697139	1	0.00279455	0.5766262	0.66313216	0.42122573
cg21393051	1	0.00730525	0.45889882	0.51498966	0.31690144
cg26035071	1	0.01009664	0.70664252	0.63449381	0.39619414
cg00054639	2	0.00304655	0.2996284	0.30867616	0.17854007
cg06660522	1	0.02466633	0.75821637	0.82916017	0.5972318
cg26192309	1	0.00094183	0.80023748	0.76611968	0.64698112
cg07356549	1	0.00594191	0.5484628	0.49718207	0.3009263
cg25467833	1	0.00127427	0.51637368	0.35633344	0.23956267
cg01966974	1	0.01889561	0.67826151	0.71190359	0.56895107
cg24486958	1	0.00068904	0.62518726	0.5267744	0.35326926
cg16242653	1	0.02252189	0.35143062	0.35229283	0.25000276
cg06873024	1	0.00414415	0.7441427	0.76894461	0.51950186
cg12436772	2	0.00670577	0.77814176	0.79311895	0.61758657
cg03165356	2	0.00384818	0.55663841	0.47812147	0.30782094
cg03834467	2	0.00353204	0.83005183	0.78137157	0.67978393
cg07181395	2	0.01183547	0.41717635	0.3982636	0.29209323
cg15559833	2	0.02087024	0.42518351	0.34341353	0.23566415
cg00132972	2	0.01674385	0.61372798	0.59413228	0.44823557
cg16651768	1	0.00065949	0.71844826	0.57534577	0.45027867
cg14101940	2	0.00514	0.6727196	0.64959861	0.54832368
cg06755427	2	0.00168532	0.60690809	0.57239677	0.41912107
cg00583068	1	0.00670577	0.76545457	0.73798464	0.57881329
cg06092869	1	0.00085355	0.34189575	0.3113995	0.19909523
cg09501717	2	0.00065949	0.86060007	0.7906922	0.66853227
cg25101764	2	0.0066172	0.82533069	0.81551207	0.66651385

cg02932364	1	0.00170239	0.67442163	0.61486216	0.47322337
cg17165759	1	0.00202174	0.73371367	0.62720615	0.51830589
cg07970146	2	0.00716301	0.66269122	0.70399697	0.51885735
cg01432692	1	0.00081789	0.88325329	0.84496701	0.67828903
cg04031093	1	0.00179741	0.54924108	0.52007084	0.27378149
cg13058623	1	0.02096205	0.41884003	0.37535302	0.23395645
cg03262802	2	0.0008696	0.84017713	0.77700403	0.63449861
cg10714509	1	0.00546217	0.55726153	0.60940947	0.4164925
cg10881749	1	0.00705117	0.63517949	0.6498834	0.47665564
cg13777730	1	0.02115579	0.66126978	0.67612481	0.45383911
cg26314722	1	0.00316303	0.78322873	0.74845327	0.54919492
cg25341313	1	0.00377723	0.46901876	0.44567268	0.28337824
cg20155035	1	0.01896129	0.84144667	0.82231819	0.71547498
cg05926640	1	0.00849487	0.76169719	0.79449278	0.54183663
cg18530568	2	0.00792881	0.68440914	0.71287457	0.488095
cg14127046	2	0.00806395	0.86531522	0.8593835	0.74841051
cg15867307	2	0.00065949	0.65040274	0.48922227	0.36665785
cg22304705	2	0.00074016	0.90941801	0.82960617	0.71093372
cg04838191	2	0.00109606	0.8967991	0.88803525	0.78344244
cg03190769	2	0.01296171	0.33238991	0.33591288	0.20758652
cg18141622	2	0.04320111	0.64998185	0.65829567	0.50668251
cg07480446	2	0.00132756	0.83999984	0.79400939	0.68918471
cg06761220	2	0.00065949	0.74542607	0.65310873	0.50307269
cg16640358	2	0.01583141	0.46309049	0.46396065	0.32544082
cg08454015	1	0.0012504	0.81931197	0.75364721	0.57833876
cg15808795	2	0.00183809	0.84666793	0.77961212	0.67719184
cg24232510	2	0.00065949	0.82346865	0.69081075	0.53529571
cg11792671	2	0.04716956	0.56381264	0.6014022	0.44977643
cg00248242	2	0.00763406	0.41710732	0.37894839	0.24775425
cg09499095	2	0.02980647	0.31744361	0.34476574	0.21626457
cg24980709	2	0.00073118	0.72658548	0.623728	0.48497303
cg16110704	1	0.00141131	0.4395331	0.46013174	0.29584876
cg11370814	2	0.00290723	0.60699352	0.51683303	0.38116522
cg00967073	1	0.00699558	0.36298956	0.35405819	0.20737106
cg25286679	1	0.0024732	0.6196357	0.55178091	0.43932562
cg02148612	1	0.00904235	0.50252899	0.55318789	0.38506508
