# Supplementary material for: Differential DNA methylation and expression of inflammatory and zinc transporter genes defines subgroups of osteoarthritic hip patients
Source: Ann Rheum Dis. 2015 Apr 8;74(9):1778–82. doi: 10.1136/annrheumdis-2014-206752 (PMC4552898; doi:10.1136/annrheumdis-2014-206752)
Supplement: Web table 4 [file annrheumdis-2014-206752-s8.pdf]

Supplementary Table 4. List of hypermethylated DMLs in OA hip cluster 2.

Hypermethylated in OA hip cluster 2						
**Probe**	**Gene**	**CHR**	**adj.P val**	**mean β NOF**	**mean β cluster 1**	**mean β cluster 2**
cg04006565	A2BP1;A2	16	0.00626821	0.47431908	0.479589673	0.642442681
cg25710107	A2LD1	13	0.00168532	0.33288494	0.374014983	0.578197911
cg13776095	A2LD1;A2	13	0.00117377	0.50176139	0.550007564	0.687663724
cg07439546	ABAT;AB	16	0.00179741	0.29083781	0.311020576	0.458620744
cg06400857	ABCA2;AB	9	0.00220808	0.25097072	0.239785499	0.361063742
cg14151317	ABCA2;AB	9	0.00253175	0.44913082	0.351588157	0.627657431
cg01807748	ABCA4	1	0.00430465	0.12171182	0.095298452	0.237625043
cg07372195	ABCA5;AB	17	0.00100054	0.60985001	0.634147407	0.815047177
cg21660392	ABCA8	17	0.0106258	0.37389018	0.392276928	0.551651702
cg09330923	ABCB8	7	0.00233298	0.6146422	0.602164431	0.737506678
cg18473642	ABCB9;AB	12	0.0022873	0.41460194	0.376045799	0.51984814
cg15656257	ABCB9;AB	12	0.04629574	0.19542502	0.192828921	0.301544032
cg06503456	ABCC1;AB	16	0.00452348	0.22993631	0.19001339	0.432957911
cg00241432	ABCC4;AB	13	0.00255831	0.41502837	0.459382599	0.673754491
cg10311020	ABI1;ABI1	10	0.00624726	0.43681294	0.444030625	0.623454638
cg15895268	ABL2;ABL	1	0.03502085	0.36345723	0.400702722	0.533283983
cg05733497	ABLIM2;A	4	0.00074801	0.59717169	0.623059961	0.730911679
cg25046274	ABLIM2;A	4	0.01193746	0.39120043	0.456623031	0.570713125
cg23401459	ABR;ABR	17	0.00209427	0.74901353	0.740475717	0.863741606
cg14458783	ABR;ABR	17	0.0041734	0.31256713	0.276698327	0.480803417
cg08458121	ABR;ABR	17	0.00663444	0.30773286	0.28113315	0.456752602
cg04318619	ABR;ABR	17	0.02519732	0.27075289	0.198636984	0.388786052
cg10803453	ABR;ABR;	17	0.00161582	0.45903084	0.504755155	0.616332616
cg24439623	ABR;ABR;	17	0.03783756	0.24631824	0.239817707	0.346392892
cg10996148	ABR;ABR;	17	0.00703549	0.29234679	0.252763607	0.436511606
cg10370025	ABR;ABR;	17	0.00119385	0.19654698	0.191876886	0.405409516
cg05633070	ABR;ABR;	17	0.00113577	0.43764949	0.500183456	0.635224609
cg24210280	ABR;ABR;	17	0.0118868	0.4151251	0.444276558	0.581012483
cg08663298	ABTB2	11	0.00666067	0.57149452	0.569228596	0.694329452
cg04954111	ABTB2	11	0.00590146	0.13602838	0.131413307	0.273571754
cg01369908	ABTB2	11	0.00798569	0.50584096	0.521953811	0.630356627
cg10548708	ACAA1;A(	3	0.00189609	0.68879915	0.677483658	0.792547428
cg01760189	ACACA;A(	17	0.00146869	0.38671456	0.422787447	0.652892815
cg16822666	ACACA;A(	17	0.00197576	0.22423123	0.217440055	0.387433272
cg10458080	ACAD10;A	12	0.04937176	0.32166355	0.340068012	0.482784595
cg08931456	ACAP2	3	0.00151706	0.44890784	0.478527606	0.640595932
cg03003335	ACAP3	1	0.01891633	0.32665189	0.339944598	0.496450584
cg19414302	ACAP3	1	0.0011571	0.39103345	0.44676133	0.605158088
cg22675767	ACAP3	1	0.00081413	0.24611481	0.282856019	0.396556841
cg17952826	ACAT2	6	0.02271157	0.17076527	0.174765354	0.279813274

cg17475583	ACBD6	1	0.02195382	0.20356383	0.147010428	0.36632378
cg26142972	ACCN4;A(	2	0.0016785	0.18841554	0.192895747	0.325596534
cg08172947	ACO2	22	0.04194736	0.50552433	0.489875723	0.615021861
cg18180407	ACOT11;/	1	0.0027878	0.25948009	0.249029831	0.443106385
cg02328793	ACOT7;A(	1	0.00623418	0.45723242	0.485290487	0.588484351
cg11654006	ACOT7;A(	1	0.00579556	0.24866996	0.242056701	0.454879089
cg26570804	ACOT7;A(	1	0.00403008	0.26511856	0.225585899	0.474054079
cg24597207	ACOX3;A(	4	0.0008696	0.56365579	0.602891423	0.765804851
cg08529267	ACOX3;A(	4	0.00329066	0.43727762	0.444647776	0.605820299
cg08206079	ACOX3;A(	4	0.0107931	0.49566756	0.476363589	0.617814288
cg09133485	ACOX3;A(	4	0.00691903	0.39401324	0.357550903	0.52813479
cg26869512	ACOX3;A(	4	0.00108946	0.41736766	0.473968293	0.713911833
cg17949316	ACOX3;A(	4	0.00297992	0.50695213	0.516659213	0.66195499
cg22002216	ACSF2	17	0.00171175	0.39987338	0.44725825	0.554362095
cg18038894	ACSF2	17	0.00065949	0.23175642	0.33994089	0.47075532
cg14906909	ACSS3	12	0.02762555	0.36736656	0.346378885	0.485190117
cg24474409	ACTR3C;L	7	0.02970062	0.38330218	0.371219335	0.487090783
cg01234517	ACTR3C;L	7	0.0075368	0.35742407	0.382205895	0.503570247
cg00653455	ACTR8	3	0.01219095	0.45890384	0.421109665	0.630707403
cg09648467	ADAM19	5	0.00179679	0.08985551	0.097661256	0.202328751
cg26523264	ADAMTS1	19	0.01970757	0.62438541	0.590341956	0.727330451
cg12845177	ADAMTS1	19	0.00659227	0.5127066	0.558057188	0.728060898
cg06982745	ADAMTS1	10	0.02763558	0.4787342	0.442309782	0.587130829
cg24368226	ADAMTS1	15	0.00065949	0.13514056	0.222565196	0.336850547
cg21901223	ADAMTS2	5	0.00532167	0.29697458	0.317618668	0.423338082
cg17212277	ADAMTS2	5	0.00193482	0.47283625	0.510772593	0.643883224
cg27000228	ADAMTS2	5	0.00118031	0.35625467	0.381162116	0.50911567
cg10077410	ADAMTS7	15	0.00403008	0.43227751	0.422394827	0.547948853
cg13468759	ADAMTSI	9	0.00247655	0.35462637	0.383492193	0.625939662
cg18127823	ADAP1	7	0.00575746	0.30623693	0.316107973	0.448741814
cg08944086	ADARB2	10	0.00193482	0.17670048	0.206495553	0.337100592
cg26361041	ADARB2	10	0.03453375	0.31125603	0.325814823	0.455421018
cg16990474	ADARB2	10	0.04470828	0.39395499	0.382104961	0.509431607
cg01294521	ADAT3;S(	19	0.00359124	0.38206408	0.376304021	0.545951858
cg19254118	ADAT3;S(	19	0.00104158	0.48646211	0.533645247	0.709495296
cg01397065	ADAT3;S(	19	0.00074016	0.26318299	0.310493645	0.506799547
cg20243900	ADAT3;S(	19	0.00367708	0.45167639	0.506757401	0.647974588
cg06887224	ADCY2	5	0.0107931	0.2060656	0.199256403	0.359900527
cg14418176	ADCY3	2	0.00263648	0.16494108	0.161735216	0.300419424
cg10639428	ADCY3	2	0.00376781	0.21348698	0.214416875	0.319325831
cg08266286	ADCY3;AI	2	0.00130383	0.13629563	0.140262983	0.258479181
cg00061520	ADCY9	16	0.00317432	0.33613984	0.425810547	0.612680811
cg00162104	ADD1;AD	4	0.0048909	0.42286633	0.408163358	0.580778814
cg03532879	ADIG	20	0.00170786	0.49221002	0.548147855	0.674087416
cg14271690	ADIG	20	0.0035878	0.22381879	0.207642708	0.349902153
cg27392775	ADIPOR1;	1	0.01137235	0.5448716	0.550956877	0.657024066

cg19678564	ADORA1;.	1	0.00569156	0.2867513	0.255587336	0.409052091
cg21083556	ADSSL1	14	0.00137603	0.34614598	0.338189043	0.541120111
cg04522949	ADSSL1;A	14	0.00103808	0.41734188	0.422467327	0.527206746
cg07406289	AFAP1;AF	4	0.01554011	0.21885486	0.20396861	0.323090303
cg01592387	AFAP1;AF	4	0.04902903	0.45680027	0.44783309	0.595755582
cg07109358	AFAP1;AF	4	0.0013661	0.32822314	0.333502128	0.454977487
cg20147046	AFAP1;AF	4	0.00220808	0.32416674	0.325091882	0.430607149
cg02465859	AFAP1L2;	10	0.0440672	0.2279353	0.20445031	0.331319433
cg17875845	AFF1;AFF	4	0.00159552	0.35390918	0.363106317	0.596788874
cg25350306	AFF3;AFF	2	0.0020873	0.19727137	0.194383734	0.378405967
cg01649623	AFF3;AFF	2	0.01158752	0.15457897	0.157165943	0.268626968
cg20965469	AFF3;AFF	2	0.00126337	0.6522135	0.594538709	0.764409552
cg10140126	AFF3;AFF	2	0.00873782	0.4355034	0.46855813	0.5703987
cg01254170	AGAP1;A(	2	0.00702677	0.34882386	0.355082443	0.532010575
cg07164047	AGAP1;A(	2	0.00295373	0.43692769	0.507297076	0.608369535
cg23467747	AGAP1;A(	2	0.00971274	0.65477814	0.657172515	0.773901919
cg16823042	AGAP2;L(	12	0.01160447	0.22718557	0.227164092	0.330707673
cg23387569	AGAP2;L(	12	0.01943805	0.2430325	0.205642887	0.352166743
cg14845962	AGAP2;L(	12	0.02651696	0.14596633	0.118760865	0.249894937
cg25417842	AGAP3	7	0.01430511	0.3835687	0.360226204	0.494625465
cg11817411	AGAP3;A(	7	0.01956069	0.49564516	0.488449233	0.596778766
cg08321684	AGER;AG	6	0.00352201	0.30886728	0.324428858	0.487094553
cg05914981	AGER;AG	6	0.00264473	0.27241413	0.28075241	0.400561081
cg01331461	AGPAT3;/	21	0.0020588	0.18500002	0.176866375	0.291815562
cg19057779	AGPAT3;/	21	0.02038239	0.51471466	0.544061861	0.665288293
cg12859101	AGPAT6	8	0.00211914	0.43930381	0.460160385	0.617324583
cg18272461	AGRN	1	0.00597656	0.31104854	0.302956775	0.413403873
cg06712559	AGRN	1	0.00073118	0.04484806	0.075698228	0.184536791
cg08057869	AGXT2L1;	4	0.00227327	0.26504484	0.232774081	0.437697496
cg07181257	AHDC1	1	0.00174123	0.33454677	0.330926763	0.503660927
cg11480534	AHNAK	11	0.02993615	0.24802165	0.233203556	0.377137385
cg02283949	AIG1	6	0.01333054	0.21643728	0.156926737	0.346298545
cg06483820	AKAP1	17	0.00129758	0.47648293	0.507897091	0.687727987
cg17133734	AKAP13;/	15	0.00953692	0.24949722	0.231183094	0.41935841
cg13263323	AKAP13;/	15	0.00802462	0.28126747	0.176191389	0.392542719
cg13430173	AKAP2;Ak	9	0.01372997	0.20419759	0.207027256	0.35161357
cg25862117	AKAP7	6	0.00144772	0.1493247	0.168075515	0.304056295
cg09794444	AKD1	6	0.00430465	0.63404581	0.599673574	0.755800833
cg07160992	AKT1;AKT	14	0.02917135	0.36018246	0.376288983	0.494826167
cg04820159	AKT1;AKT	14	0.00113577	0.13127518	0.134877689	0.239139646
cg14274357	AKT1;AKT	14	0.00248015	0.32783549	0.328090865	0.475331321
cg11973514	AKT3;AKT	1	0.00677233	0.74858696	0.76353049	0.864929607
cg27451508	ALDH3A1	17	0.00156869	0.59863901	0.614095601	0.743496873
cg12491555	ALDH8A1	6	0.00702785	0.21955669	0.277693469	0.412773725
cg02693227	ALG1L2	3	0.04738917	0.38063404	0.436739922	0.589300554
cg18007837	ALOX12P:	17	0.01020581	0.16706025	0.147756283	0.268473457

cg11027217	ALPK1;AL	4	0.00429038	0.36073078	0.352744458	0.463030119
cg00031779	ALPK2	18	0.00377723	0.2802751	0.304473127	0.471545452
cg27555092	ALPK2	18	0.00167382	0.11260307	0.115922818	0.256429764
cg07910680	ALPK2	18	0.00150566	0.1881335	0.196809555	0.35500887
cg27614534	ALPK2	18	0.00083551	0.53006778	0.597630803	0.705922948
cg25407077	ALPK2	18	0.00359691	0.52369163	0.546767583	0.679108671
cg00894103	ALPK3	15	0.02494466	0.41245538	0.368210799	0.524273571
cg26893816	AMACR;A	5	0.00841172	0.20543367	0.201080871	0.329506243
cg20406747	AMHR2;A	12	0.00173053	0.20035447	0.220584731	0.402236679
cg15616310	AMOTL1	11	0.01766834	0.1346282	0.122836382	0.250505882
cg01636354	AMOTL2	3	0.00170786	0.1474386	0.154290998	0.262206068
cg08173606	AMOTL2	3	0.00263648	0.11090519	0.110089821	0.214979483
cg27565899	AMPD2;A	1	0.00067897	0.12132633	0.163183198	0.274609372
cg25772143	AMPD2;A	1	0.00126059	0.25555033	0.263572413	0.370015452
cg24435741	AMPD3	11	0.04237386	0.27593316	0.308846999	0.451458343
cg22308970	AMPH;AN	7	0.0019141	0.44619189	0.508261063	0.626251593
cg06618764	AMZ1	7	0.00138618	0.33502458	0.307201085	0.454788245
cg06703803	ANAPC7	12	0.00230116	0.26735007	0.237933728	0.474194342
cg25116615	ANGPT1	8	0.00401281	0.44022858	0.472999569	0.596406127
cg05234958	ANK1;AN	8	0.00291825	0.57489521	0.576168584	0.705812896
cg13555290	ANK2	4	0.01807343	0.35959126	0.298144317	0.488359502
cg07677598	ANK2	4	0.00169362	0.41341817	0.416779394	0.648634251
cg17665652	ANK2;AN	4	0.00536676	0.14657919	0.088652319	0.281993403
cg01497527	ANKDD1A	15	0.00295889	0.38004964	0.381103565	0.495874985
cg20647656	ANKMY1;	2	0.0011414	0.55779107	0.586876316	0.710741774
cg25810938	ANKMY1;	2	0.01580869	0.11872078	0.099191157	0.223079874
cg09420319	ANKRD11	16	0.00362881	0.26024458	0.258298372	0.46913423
cg05071195	ANKRD11	16	0.01148808	0.22593474	0.19974317	0.339444524
cg08420923	ANKRD11	16	0.00703053	0.31167492	0.283201685	0.44772242
cg06567829	ANKRD11	16	0.01957672	0.35290488	0.377232366	0.510419103
cg00770754	ANKRD11	16	0.00597656	0.48942384	0.482733671	0.615285587
cg03163759	ANKRD11	16	0.00488538	0.25646517	0.239689578	0.359577999
cg00488747	ANKRD11	16	0.005559	0.5895862	0.572321832	0.738114792
cg09486407	ANKRD11	16	0.00104158	0.33491258	0.392935736	0.627910442
cg22889914	ANKRD2;A	10	0.0009601	0.37827404	0.432321734	0.594284167
cg02950892	ANKRD2;A	10	0.00143276	0.28085281	0.329073709	0.454929307
cg10343327	ANKRD2;A	10	0.00107365	0.38594537	0.442830957	0.604168478
cg09114211	ANKRD23	2	0.00181052	0.5486812	0.550928061	0.654084962
cg25612428	ANKRD33	5	0.00451843	0.40992018	0.363849008	0.523009518
cg07683388	ANKRD44	2	0.00520163	0.24484314	0.226524173	0.351574017
cg04869949	ANKRD44	2	0.00218291	0.56154468	0.589290187	0.719778428
cg16501561	ANKRD55	5	0.02830078	0.19330233	0.181088302	0.344522675
cg03613003	ANO1;AN	11	0.00137194	0.23184181	0.260994012	0.468232626
cg15767218	ANO1;AN	11	0.00582851	0.25531961	0.242380151	0.435337078
cg13767135	ANO1;AN	11	0.02541872	0.20014836	0.197777027	0.303493989
cg04146573	ANO1;AN	11	0.00184462	0.1170902	0.129705153	0.232446283

cg02179492	ANO1;AN	11	0.00236311	0.55923158	0.5800274	0.692074326
cg20883194	ANO6;AN	12	0.00236311	0.11514724	0.143584812	0.294313331
cg01410615	ANO7	2	0.00185385	0.14787749	0.134583518	0.317418362
cg25605313	ANO8	19	0.0219159	0.15217096	0.141229264	0.258967636
cg12526923	ANO8	19	0.00327469	0.21518987	0.279558738	0.396096697
cg24192660	ANO8	19	0.0012838	0.1503785	0.226541437	0.33977312
cg24530041	ANPEP	15	0.00329066	0.34096255	0.378953865	0.52154459
cg13042288	ANPEP	15	0.00295373	0.54065733	0.628151485	0.74524392
cg20836795	ANTXR1;/	2	0.01238447	0.22716713	0.186255296	0.348802464
cg03164800	ANTXR2	4	0.00403972	0.42160051	0.503162576	0.621412907
cg14818154	ANTXR2	4	0.02496653	0.29182747	0.328205541	0.443857217
cg16326777	ANTXR2	4	0.02326197	0.49544596	0.581225433	0.704829727
cg11900509	ANXA11;/	10	0.00357923	0.17234089	0.172539447	0.309605664
cg03730709	ANXA11;/	10	0.00205947	0.20397622	0.209791409	0.363691822
cg15892650	ANXA6;AI	5	0.00092643	0.28124575	0.340923551	0.44812577
cg20437604	ANXA9;AI	1	0.00450697	0.30851807	0.317842803	0.468412792
cg14707353	AOX1	2	0.00768774	0.60671928	0.628730565	0.765492456
cg03832197	AOX2P	2	0.0154863	0.3785106	0.303871633	0.482432403
cg21185674	AP2A2	11	0.01236318	0.39723264	0.392350101	0.530651659
cg27205902	AP2A2	11	0.00073118	0.2524728	0.338129611	0.648988778
cg14389139	AP2A2	11	0.00293735	0.46545141	0.542748115	0.656463148
cg02570643	AP3B1	5	0.0031673	0.56725267	0.538482395	0.676397735
cg17656426	AP3D1;AI	19	0.0044883	0.56235792	0.532949994	0.694105154
cg13917002	APBA1	9	0.00627871	0.17940317	0.19428843	0.321414947
cg27216937	APBB1;AI	11	0.01396719	0.52037509	0.518164404	0.633411735
cg23774717	APBB1IP	10	0.01574127	0.21724528	0.201904371	0.319960619
cg23877017	APBB2;AI	4	0.00539186	0.20559539	0.234656065	0.4091651
cg26865481	APBB2;AI	4	0.00569156	0.66447101	0.691986786	0.79961044
cg25611723	APCDD1	18	0.0130683	0.28653776	0.28800269	0.395397779
cg04229487	APCDD1	18	0.04615769	0.57231424	0.571108118	0.677588549
cg17548735	APOBEC2	6	0.00133251	0.26928762	0.276852222	0.491249016
cg24424889	APOBEC3	22	0.00146267	0.35210367	0.424193444	0.557600024
cg05502439	AQP6	12	0.00590146	0.22404266	0.212250295	0.340921986
cg27321325	ARAP1;AI	11	0.02110372	0.33012523	0.316653233	0.440398095
cg00487506	ARC	8	0.00629716	0.43180986	0.44163637	0.565411083
cg16178743	ARG1;ME	6	0.01934763	0.40144182	0.443021948	0.582289789
cg14780276	ARHGAP1	4	0.01629158	0.31803683	0.308340935	0.449391153
cg01165159	ARHGAP1	4	0.00222822	0.23488168	0.248974103	0.401515396
cg01381498	ARHGAP1	4	0.00537181	0.25028371	0.318069303	0.431436572
cg26282731	ARHGAP1	4	0.00073118	0.11274845	0.168179148	0.337985491
cg17600630	ARHGAP1	2	0.00329066	0.20172652	0.24454285	0.354449059
cg14575356	ARHGAP1	6	0.00065949	0.11485598	0.210275097	0.324349936
cg03930313	ARHGAP2	17	0.01700751	0.2081501	0.180006245	0.314685792
cg20028019	ARHGAP2	17	0.0023961	0.48242998	0.435805574	0.588491903
cg23659289	ARHGAP2	17	0.00258198	0.11346491	0.10366473	0.231817066
cg27244773	ARHGAP2	17	0.0070644	0.33614275	0.276756353	0.46006096

cg10094238	ARHGAP2	17	0.00211366	0.30391855	0.270238759	0.41385653
cg10236857	ARHGDIA	17	0.00132756	0.31532103	0.331556412	0.446512174
cg26074051	ARHGDIA	17	0.00391963	0.74408322	0.675045167	0.879244623
cg11326429	ARHGDIA	17	0.00758031	0.24197933	0.236791954	0.347121533
cg05503433	ARHGEF1	19	0.00097592	0.35032179	0.382699586	0.485841843
cg11492536	ARHGEF1	8	0.02214521	0.53659569	0.571928568	0.676896432
cg22223001	ARHGEF1	8	0.00213077	0.49821928	0.549024906	0.714265045
cg15414698	ARHGEF1	8	0.00802646	0.57189766	0.599459108	0.703517784
cg03705571	ARHGEF1	8	0.0011122	0.21818179	0.274290178	0.465992408
cg08381013	ARHGEF1	8	0.00312811	0.58455354	0.596693646	0.708990175
cg20149022	ARHGEF1	8	0.00081599	0.22487794	0.326063832	0.476594293
cg19351604	ARHGEF1	8	0.02680411	0.48929629	0.51675308	0.632184166
cg01607321	ARHGEF1	8	0.00753326	0.31578218	0.310996309	0.447430441
cg07694735	ARHGEF1	8	0.00165917	0.33449357	0.349110749	0.583414735
cg06807837	ARHGEF1	8	0.00085934	0.48107451	0.52044579	0.734257151
cg01824603	ARHGEF1	8	0.0011571	0.34365494	0.36763851	0.607699784
cg09915232	ARHGEF1	8	0.00113577	0.56307553	0.5673839	0.672582387
cg23780635	ARHGEF1	8	0.00491697	0.33125883	0.312139368	0.47639498
cg21161403	ARHGEF1	8	0.00084118	0.25365228	0.26941543	0.380833571
cg20690085	ARHGEF1	8	0.02549119	0.2418051	0.212204799	0.380790109
cg20294080	ARHGEF1	1	0.01384716	0.2598926	0.248425602	0.393079139
cg24371534	ARHGEF1	1	0.00534446	0.34095296	0.359092949	0.481802272
cg20430773	ARHGEF1	1	0.00745107	0.11961934	0.100005571	0.227700035
cg03887218	ARHGEF1	1	0.00456378	0.29612857	0.253110571	0.427369659
cg05722035	ARHGEF3	3	0.04457786	0.39799079	0.325292162	0.503472707
cg05400732	ARHGEF3	3	0.00317348	0.39972056	0.258158879	0.509655689
cg04606397	ARID1B;A	6	0.02041679	0.45194351	0.478191029	0.582316113
cg00877708	ARID1B;A	6	0.00486477	0.4977107	0.583407711	0.68933205
cg02671826	ARID1B;A	6	0.00262867	0.65422082	0.687375658	0.801494918
cg03561416	ARID1B;A	6	0.00106137	0.17771841	0.20455331	0.352086998
cg14987175	ARID1B;A	6	0.00287532	0.30559492	0.28894233	0.43645133
cg26758131	ARID5B	10	0.02802634	0.47903663	0.479894944	0.581242673
cg10845391	ARL10	5	0.00179217	0.18018183	0.202147477	0.369380305
cg02437766	ARL15	5	0.02987215	0.2022441	0.217603836	0.329284275
cg21467371	ARL16	17	0.00165724	0.31513076	0.294583252	0.41557237
cg19140602	ARMC4	10	0.00705647	0.42943707	0.33336312	0.532416961
cg13004609	ARMC9	2	0.01630619	0.4735212	0.504443141	0.614189817
cg10435282	ARNTL;AF	11	0.00137603	0.44552086	0.445407607	0.633714171
cg19536902	ARNTL;AF	11	0.02368354	0.68282886	0.667830105	0.783460733
cg11699265	ARPC1B	7	0.00259677	0.19769045	0.18314652	0.313869775
cg21852346	ARPP-21;.	3	0.00197986	0.5042788	0.500214173	0.684675254
cg17456851	ARSB	5	0.00180881	0.48888191	0.443463248	0.72086476
cg15922611	ARSJ	4	0.00889507	0.20781649	0.208070757	0.364694258
cg13258774	ASAP2;AS	2	0.00375919	0.12446059	0.109311021	0.242331369
cg05027258	ASAP2;AS	2	0.03358212	0.71872037	0.725841638	0.855796401
cg24580199	ASAP3;AS	1	0.0284419	0.28902436	0.247271153	0.408181141

cg24654025	ASB13;AS	10	0.02549971	0.42336964	0.407279224	0.541126245
cg08240466	ASIP	20	0.00429163	0.34040819	0.424153393	0.560179984
cg10584100	ASPH;ASF	8	0.02995719	0.68655209	0.69896984	0.80952479
cg22949575	ASPSCR1	17	0.0013706	0.49043883	0.533060227	0.719664601
cg04784499	ASPSCR1	17	0.00220808	0.43388534	0.436240156	0.566552083
cg11780549	ASPSCR1	17	0.00310177	0.29577915	0.312375067	0.45078566
cg02647835	ASPSCR1	17	0.00220808	0.33121729	0.343578452	0.497013677
cg08311321	ASPSCR1	17	0.00431293	0.25586778	0.250704109	0.445107395
cg14419393	ASPSCR1	17	0.00449495	0.33589012	0.347271529	0.563100767
cg09925620	ASPSCR1	17	0.00072011	0.61654354	0.677242313	0.796659019
cg01410415	ASPSCR1	17	0.00320723	0.26063364	0.258685496	0.418321439
cg26955287	ASPSCR1	17	0.00181052	0.34253324	0.359649237	0.627359286
cg15438211	ASPSCR1	17	0.00089116	0.19898025	0.206106511	0.353365429
cg06483978	ASPSCR1	17	0.00081925	0.27726427	0.276460453	0.424089348
cg23928512	ASPSCR1	17	0.00314361	0.42976096	0.490238518	0.605567086
cg12374579	ASPSCR1	17	0.00174903	0.31415258	0.336708695	0.562811905
cg14010829	ASS1;ASS	9	0.00283173	0.26385537	0.291684128	0.475945274
cg14053997	ASS1;ASS	9	0.00126466	0.10600713	0.117836937	0.218292474
cg13792444	ASTN2;AS	9	0.00167382	0.23629071	0.244748551	0.456947626
cg20363931	ATE1;ATE	10	0.00420965	0.19126904	0.190916113	0.347366674
cg10064029	ATF6	1	0.01622063	0.21328242	0.253919044	0.401948284
cg13074795	ATF7;ATF	12	0.02505273	0.19106502	0.207305302	0.33832156
cg07238630	ATG10;AT	5	0.00142458	0.51859431	0.51354734	0.708942513
cg22214498	ATG2A	11	0.00318676	0.56729744	0.536214411	0.6844242
cg16721106	ATG4B;AT	2	0.00255774	0.66034655	0.661429166	0.797980637
cg00977707	ATG7;ATC	3	0.00899313	0.44640743	0.507612446	0.623118991
cg24434988	ATHL1	11	0.00840111	0.44832969	0.446584207	0.616253786
cg12139707	ATM;ATM	11	0.00107365	0.61657538	0.692166866	0.800197667
cg05471373	ATM;ATM	11	0.00080814	0.56099667	0.662900403	0.789150609
cg20839205	ATOH8	2	0.00096621	0.14101108	0.183951736	0.362211268
cg18256640	ATP10A	15	0.00520958	0.2053301	0.198189136	0.37595412
cg06206670	ATP11A;A	13	0.00335524	0.46561056	0.484475872	0.669918127
cg14737571	ATP11A;A	13	0.00539451	0.46215513	0.494313824	0.621740924
cg10883123	ATP11A;A	13	0.00357848	0.36626881	0.383549974	0.554017844
cg14649073	ATP11A;A	13	0.03270435	0.37639319	0.35474604	0.487068919
cg14381255	ATP11A;A	13	0.00349176	0.27277839	0.299040839	0.496925412
cg02873074	ATP11A;A	13	0.00191447	0.30187577	0.348064025	0.478923524
cg19970086	ATP11A;A	13	0.00334886	0.50309803	0.546826453	0.678592843
cg13858127	ATP11A;A	13	0.00777499	0.51748639	0.555344567	0.684393153
cg11840467	ATP11A;A	13	0.00213077	0.30601802	0.326796197	0.462663562
cg11462099	ATP11A;A	13	0.00521564	0.11529656	0.114246978	0.223807865
cg17434062	ATP13A2;	1	0.01533394	0.30288527	0.281070925	0.426983941
cg04403423	ATP1A1;A	1	0.01466514	0.44875622	0.491049782	0.610690359
cg01448881	ATP1A2	1	0.00624726	0.3600669	0.357832888	0.492011241
cg26009832	ATP1B1;A	1	0.02388672	0.28155206	0.265834426	0.439054738
cg10095954	ATP2A3;A	17	0.01293322	0.29200043	0.263925854	0.418077733

cg03956243	ATP5G2;A	12	0.00376781	0.16491072	0.158670052	0.298820575
cg05883442	ATP6V0D	16	0.00077468	0.21643318	0.254822479	0.377170948
cg02286270	ATP8A2	13	0.0015244	0.50300126	0.543998524	0.702365513
cg17488500	ATP8B2;A	1	0.01585745	0.20743973	0.162182297	0.307846564
cg22288309	ATP8B4	15	0.02681286	0.4392286	0.444217628	0.636937744
cg07111441	ATPAF1;A	1	0.02649037	0.31390655	0.328243604	0.471306176
cg19566879	ATXN1;AT	6	0.00432931	0.41141508	0.380559927	0.562300786
cg26843612	ATXN1;AT	6	0.01007472	0.35481977	0.394263286	0.564397489
cg16280399	ATXN1;AT	6	0.00868726	0.27931643	0.354338613	0.518087251
cg08045301	ATXN1L;A	16	0.00921039	0.4144134	0.440211609	0.541353583
cg19053223	ATXN7L1	7	0.00248015	0.30138931	0.337798196	0.439910076
cg03619256	ATXN7L1;	7	0.00961538	0.22074647	0.21627135	0.326947454
cg09965388	ATXN7L3;	17	0.01073324	0.35509749	0.33551643	0.550240144
cg04601228	AUTS2;AU	7	0.00207648	0.32533613	0.275186396	0.55238131
cg19793499	AUTS2;AU	7	0.00476313	0.25086702	0.237593488	0.383764351
cg08474953	AUTS2;AU	7	0.00073265	0.62409182	0.660873528	0.801967669
cg14818546	AUTS2;AU	7	0.01006689	0.23326723	0.255997979	0.377221601
cg00012698	AVEN	15	0.02087024	0.37136123	0.391944096	0.511142647
cg24403487	AZI1;AZI1	17	0.00143053	0.50767028	0.512717435	0.621151115
cg15564207	AZI2;AZI2	3	0.0135118	0.60757855	0.580689893	0.729376994
cg12624040	B3GALT1;	2	0.04659965	0.73781612	0.674637179	0.867470719
cg02413326	B3GALT5;	21	0.00934957	0.62251658	0.644933843	0.750019195
cg05532325	B3GNT7	2	0.01096356	0.54981608	0.506677473	0.7126752
cg03037437	B3GNT7	2	0.00093925	0.30322175	0.351991476	0.485182131
cg11472422	B3GNTL1	17	0.0019386	0.20365323	0.216542094	0.454753766
cg21688152	B3GNTL1	17	0.00820352	0.20597482	0.193265061	0.34305524
cg03591954	B3GNTL1	17	0.00223317	0.19513267	0.211004537	0.327883581
cg07200616	B3GNTL1	17	0.00574659	0.16183679	0.131822908	0.266499624
cg24962672	B3GNTL1	17	0.00238047	0.33200961	0.372476411	0.563816335
cg19877683	B3GNTL1	17	0.01653406	0.35842987	0.424813014	0.540842551
cg13858803	B4GALT1	9	0.00953692	0.21942917	0.1909923	0.404693986
cg16883145	B9D2;TGF	19	0.00126059	0.57527004	0.606886321	0.724810144
cg11523350	BACE1;BA	11	0.02658897	0.32078974	0.281416949	0.427454885
cg01923020	BACE1;BA	11	0.00405051	0.41800959	0.414199157	0.543246615
cg09790829	BACH2;BA	6	0.00414201	0.26019304	0.278277028	0.401551487
cg15380603	BACH2;BA	6	0.01859349	0.39227929	0.428732282	0.551352419
cg07016556	BAHCC1	17	0.01009622	0.18888029	0.160963844	0.29795423
cg17624073	BAHCC1	17	0.00168532	0.18939303	0.159253142	0.303796598
cg25348105	BAHCC1	17	0.01572147	0.27857509	0.272890286	0.383134434
cg23532322	BAIAP2;B	17	0.0023207	0.31546829	0.354363355	0.498026634
cg11620135	BAIAP2;B	17	0.00244246	0.16867903	0.155264834	0.32609673
cg12475142	BAIAP2;B	17	0.00513872	0.23709104	0.261167736	0.396105859
cg22184944	BAIAP2;B	17	0.03409516	0.30229043	0.30335213	0.404868105
cg03060228	BAIAP2;B	17	0.00183351	0.73841373	0.726480768	0.84155264
cg12349181	BAIAP2;B	17	0.00313278	0.47762466	0.465986357	0.613530632
cg09840386	BAIAP2;B	17	0.00126816	0.20865437	0.228703346	0.440476866

cg06374794	BANP;BAI	16	0.04261712	0.40297242	0.359090468	0.524934607
cg26979299	BANP;BAI	16	0.0019469	0.39454924	0.484518999	0.603332603
cg09870004	BANP;BAI	16	0.00126059	0.20451936	0.221541224	0.38193049
cg27058702	BANP;BAI	16	0.00431293	0.29097519	0.281723283	0.470144779
cg04081145	BANP;BAI	16	0.00184468	0.583442	0.593451495	0.705937835
cg01256999	BANP;BAI	16	0.00103895	0.62321521	0.663512571	0.791169431
cg05762553	BANP;BAI	16	0.00431388	0.32062071	0.300031638	0.503587778
cg06969933	BANP;BAI	16	0.00400629	0.16997071	0.159503805	0.270060693
cg15647614	BAT1;BAT	6	0.0039229	0.55032302	0.587600833	0.706457919
cg10660666	BAT2L1	9	0.01021297	0.50806064	0.549899067	0.684186579
cg09144098	BBS2	16	0.02987844	0.26215135	0.281215461	0.427019651
cg05891572	BBS9;BBS	7	0.03453375	0.32528376	0.339842004	0.538018469
cg13619623	BBS9;BBS	7	0.04180327	0.40273507	0.425518477	0.545890804
cg20299670	BCAN	1	0.00482325	0.6217093	0.437903494	0.722227259
cg08284564	BCAR1;BC	16	0.01059488	0.47648508	0.513048024	0.62551519
cg06888900	BCAR1;BC	16	0.00253378	0.28460252	0.267922718	0.433907214
cg02853019	BCAR1;BC	16	0.01206679	0.22736829	0.197816339	0.333337596
cg17192381	BCL2	18	0.00290119	0.46224841	0.518521561	0.629580572
cg26157948	BCL3	19	0.02630407	0.44851581	0.423989512	0.584688106
cg26811705	BCL9L;BC	11	0.00137133	0.15346837	0.150853079	0.281998497
cg20088756	BEGAIN;E	14	0.00342408	0.44200316	0.463305703	0.653581519
cg12025832	BEGAIN;E	14	0.00177922	0.19587407	0.19723925	0.33099146
cg19883293	BEGAIN;E	14	0.00255831	0.13819812	0.143143412	0.25071173
cg01963573	BEND3	6	0.00144772	0.35672357	0.384200884	0.551810113
cg08811259	BEND6	6	0.02205285	0.18774743	0.196753297	0.321203092
cg11562596	BEST1;BE	11	0.00497255	0.32580952	0.346703733	0.483526368
cg17277939	BEST3;BE	12	0.01446513	0.48074913	0.457876315	0.627206755
cg13533456	BET3L	6	0.00338706	0.31450461	0.445427315	0.585694596
cg18815565	BFSP1	20	0.02870038	0.33604247	0.295920121	0.45746022
cg26344982	BHLHE40	3	0.00339981	0.27395423	0.250281068	0.41353329
cg18768347	BHLHE40	3	0.0059385	0.24869517	0.216553561	0.383470128
cg25629905	BHLHE41	12	0.00716301	0.2940989	0.243655122	0.448694119
cg24791156	BIN3	8	0.00341796	0.38254582	0.343179518	0.509831404
cg15270350	BIRC5;BIF	17	0.0019906	0.16995448	0.176003513	0.343697197
cg11971852	BIRC6	2	0.02833301	0.16767518	0.156700072	0.279046126
cg14579118	BLVRA	7	0.00085355	0.19633133	0.24489028	0.419564959
cg26665274	BMP2	20	0.0010778	0.31766555	0.276852283	0.563504426
cg05923197	BMP4;BM	14	0.00380612	0.41380867	0.41913471	0.53521378
cg15476602	BMPR1A	10	0.00186737	0.32348786	0.311834874	0.478920121
cg23210521	BMPR1B	4	0.00074016	0.34210406	0.53558042	0.641443337
cg06147895	BNIP3	10	0.00124152	0.18893177	0.210763384	0.372371805
cg02365648	BNIP3	10	0.00075702	0.15957234	0.229999097	0.427260915
cg22987267	BNIP3L	8	0.00568196	0.32108084	0.367398425	0.531488768
cg25132931	BOC	3	0.0017637	0.37398848	0.43896533	0.607420306
cg02858625	BOC	3	0.00733126	0.29470723	0.319277047	0.559005972
cg13071869	BOC	3	0.00291147	0.36889758	0.466991683	0.647050234

cg15111495	BOC	3	0.00100376	0.24979574	0.289249009	0.47097434
cg05122957	BOC	3	0.00579203	0.17408963	0.155211668	0.29326428
cg13272743	BOC	3	0.00177922	0.50510802	0.5642257	0.698497617
cg11882488	BOC	3	0.00729536	0.60541878	0.661419494	0.788371137
cg01908177	BOC	3	0.00303542	0.49156995	0.494919548	0.625545363
cg25958911	BOP1	8	0.03875896	0.64663725	0.597026309	0.749698074
cg06354558	BOP1	8	0.00734129	0.79286733	0.781736489	0.896216846
cg01294253	BRD3	9	0.00104452	0.20173615	0.228334829	0.37272679
cg03657791	BRD4	19	0.00280262	0.59476781	0.627748653	0.756179057
cg10827768	BRE;BRE;I	2	0.04901873	0.4402088	0.449176126	0.558039523
cg15161050	BRI3BP	12	0.01183088	0.53118277	0.568641879	0.682827417
cg24351452	BRSK1	19	0.00377206	0.50449955	0.483936056	0.6697867
cg10002860	BRSK2	11	0.01317683	0.35306534	0.346677525	0.483019408
cg19977004	BRSK2	11	0.00256738	0.46128443	0.447989388	0.59024764
cg24206507	BRSK2	11	0.00463097	0.50205282	0.489416715	0.681992277
cg21757872	BTBD11;E	12	0.01086076	0.1768174	0.154792694	0.297824242
cg11841741	BTBD11;E	12	0.00319532	0.14239908	0.206819246	0.322119829
cg27384342	BTBD12	16	0.02438027	0.18885371	0.202392504	0.318490528
cg06094113	BTBD18	11	0.00352383	0.29762298	0.264073173	0.443523721
cg06766016	BTBD19	1	0.03923812	0.46148283	0.469087696	0.588605055
cg25688060	BTBD3	20	0.00747148	0.47002113	0.527888179	0.640825944
cg13442386	BTBD9;BT	6	0.00588734	0.50393083	0.508651456	0.674142807
cg11914795	C10orf10	10	0.01091243	0.25126921	0.188677724	0.351633616
cg01140247	C10orf11	10	0.01387172	0.29383321	0.312067119	0.437268191
cg14207539	C10orf11	10	0.00149857	0.44729269	0.547169625	0.698111699
cg23264016	C10orf11	10	0.00094083	0.29542507	0.345011014	0.490923579
cg23966017	C10orf11	10	0.03212706	0.26595883	0.284897297	0.4349449
cg09411597	C10orf18	10	0.01206219	0.29456137	0.331659133	0.47713978
cg17502312	C10orf18	10	0.00539114	0.23451213	0.213362792	0.400916814
cg01291665	C10orf47	10	0.02182683	0.38939914	0.39673868	0.535028011
cg08571020	C10orf62	10	0.00117736	0.65890276	0.669943516	0.809167634
cg10775039	C10orf71	10	0.00480767	0.25335897	0.300751018	0.468309309
cg18895336	C10orf72	10	0.00065949	0.33596759	0.411101641	0.564566682
cg07260003	C10orf72	10	0.00065949	0.12494458	0.16518496	0.274867178
cg18286127	C10orf84	10	0.00114256	0.21840522	0.255899157	0.485081426
cg01743835	C11orf34	11	0.02846049	0.55089476	0.59750187	0.745883595
cg06579738	C11orf92	11	0.0196961	0.48388905	0.458540509	0.612737404
cg27475923	C11orf95	11	0.00232154	0.32108408	0.275342752	0.442502074
cg04686545	C12orf23	12	0.03009647	0.30258894	0.267619256	0.431582106
cg10082647	C12orf23	12	0.00798815	0.27154819	0.267863017	0.376053786
cg00019678	C12orf34	12	0.00316372	0.15249521	0.137910049	0.298910036
cg19879537	C12orf34	12	0.00419259	0.52570478	0.597828609	0.708447535
cg03061518	C12orf65	12	0.00436875	0.22064791	0.177121622	0.330182461
cg11727592	C13orf18	13	0.00359037	0.41505107	0.426803172	0.534151729
cg10664305	C13orf30	13	0.021196	0.54382314	0.575428319	0.678843885
cg17923947	C14orf13	14	0.04745403	0.50433864	0.482212823	0.604662162

cg10297617	C14orf15	14	0.01026031	0.11221884	0.100782637	0.216082635
cg00780666	C14orf17	14	0.02878476	0.41728157	0.423115097	0.578186608
cg08258506	C14orf43	14	0.01154548	0.22606213	0.235784728	0.392371239
cg24128590	C14orf43	14	0.00353178	0.27760037	0.25981595	0.471657647
cg23938483	C14orf43	14	0.00202314	0.46776393	0.478342782	0.672380534
cg21032266	C14orf43	14	0.00327239	0.38432867	0.437587142	0.607490217
cg01024668	C15orf28	15	0.0062271	0.56894803	0.591484968	0.704301819
cg03536657	C15orf28	15	0.00073118	0.40512059	0.494745643	0.622336476
cg27211935	C15orf39	15	0.00664361	0.59764565	0.580830036	0.743919956
cg20602300	C15orf57	15	0.00068904	0.34532637	0.482397392	0.686891839
cg17140476	C15orf57	15	0.00242276	0.30901974	0.373191765	0.557398295
cg03015880	C16orf13	16	0.0135118	0.47387783	0.500296101	0.624411007
cg02642565	C16orf5	16	0.00661882	0.12165337	0.110152459	0.228706801
cg05396425	C16orf5	16	0.04797329	0.41849951	0.455645839	0.56841487
cg08496964	C16orf68	16	0.00464252	0.4964174	0.514552405	0.638547849
cg26923629	C16orf71	16	0.00810401	0.22784134	0.191044024	0.379793856
cg04558424	C16orf74	16	0.00263371	0.34005064	0.320339775	0.488791952
cg26967566	C16orf74	16	0.00133856	0.33568864	0.362146239	0.53224501
cg25657222	C17orf10	17	0.00692459	0.57409402	0.611323061	0.741205603
cg11494841	C17orf62	17	0.00329066	0.2944389	0.247080663	0.400845762
cg09913183	C17orf65	17	0.02077264	0.69436488	0.655479847	0.81248549
cg25309567	C17orf70	17	0.00087274	0.31681071	0.356093901	0.529063779
cg02698731	C17orf70	17	0.00141131	0.43665875	0.449278455	0.567089851
cg26421997	C17orf79	17	0.00312077	0.56018668	0.540972317	0.663209959
cg21737421	C18orf1;C	18	0.00442574	0.57700982	0.488035559	0.691478241
cg23508887	C18orf1;C	18	0.00232775	0.09083004	0.080601558	0.210708107
cg24780966	C18orf1;C	18	0.00195644	0.32407006	0.359777844	0.59294717
cg27107110	C18orf1;C	18	0.00359124	0.45127452	0.456078708	0.570200815
cg23357435	C19orf26	19	0.00097337	0.55320967	0.554145809	0.736897912
cg07946961	C19orf34	19	0.01189791	0.3548933	0.33734668	0.483436699
cg27345346	C19orf55	19	0.01572147	0.39034695	0.354739147	0.504158815
cg17344770	C19orf71	19	0.0006736	0.22754975	0.31530102	0.54036976
cg07720789	C1orf105	1	0.01268304	0.49883583	0.509332093	0.614152227
cg17131183	C1orf113	1	0.0080158	0.28360197	0.281063225	0.438205099
cg15926004	C1orf133	1	0.00745107	0.1590898	0.126801101	0.260396628
cg23812948	C1orf159	1	0.04937176	0.43889327	0.40595543	0.61359143
cg01722868	C1orf161	1	0.00742222	0.40306695	0.419353717	0.61955772
cg14853231	C1orf174	1	0.0102761	0.35265961	0.358177207	0.470192975
cg27446570	C1orf174	1	0.00715821	0.25839005	0.221886049	0.424737009
cg15324917	C1orf198	1	0.00215533	0.13611575	0.139055743	0.309208358
cg21777154	C1orf21	1	0.00100561	0.43630886	0.481370281	0.687192835
cg11855643	C1orf21	1	0.00074409	0.43589492	0.51099984	0.652832727
cg07582892	C1orf21	1	0.00287532	0.22472455	0.314376568	0.485101196
cg15644413	C1orf216	1	0.00066232	0.36334619	0.412450725	0.586557141
cg21582311	C1orf96	1	0.00381415	0.26094342	0.233768653	0.494260552
cg04243822	C1QTNF1	17	0.03249565	0.1524468	0.133737523	0.290079492

cg02282892	C1QTNF3	5	0.04085317	0.17052556	0.174713882	0.312599967
cg03934354	C1QTNF3	5	0.00357923	0.15131913	0.143545901	0.29502997
cg18356785	C1QTNF4	11	0.01042463	0.25129637	0.198678866	0.352261676
cg09250458	C1QTNF8	16	0.01116167	0.14997565	0.138025418	0.261025758
cg02726898	C1QTNF8	16	0.02252189	0.24946966	0.243613438	0.367572203
cg03904630	C20orf11	20	0.01523475	0.62011228	0.635738484	0.75775647
cg16125816	C20orf11	20	0.00575178	0.61107723	0.653492353	0.782260664
cg02182210	C20orf16	20	0.00198606	0.24046378	0.212307732	0.372899145
cg09203029	C20orf20	20	0.00201167	0.16918912	0.147650667	0.427842403
cg23736024	C21orf12	21	0.00556434	0.59302785	0.554505938	0.714035738
cg10273072	C21orf34	21	0.02935375	0.27954522	0.333538344	0.44714156
cg06684407	C21orf67	21	0.00227742	0.27691821	0.284200082	0.472511533
cg19902195	C21orf67	21	0.00271346	0.38673754	0.394025637	0.602445292
cg10007534	C21orf70	21	0.00704342	0.32890167	0.370588446	0.520094249
cg05054998	C21orf70	21	0.0293309	0.26668922	0.258834647	0.391983935
cg06787912	C21orf82	21	0.00953692	0.28007974	0.269305144	0.478096519
cg12003230	C21orf84	21	0.00645854	0.34535701	0.319477461	0.533666305
cg01169075	C2orf34	2	0.00838112	0.55011783	0.554339558	0.660453427
cg22948077	C2orf40	2	0.00600204	0.41080714	0.47232772	0.603017112
cg02061967	C2orf48	2	0.00456818	0.54018872	0.56886574	0.671628573
cg25819429	C2orf48	2	0.0046471	0.30987168	0.325178274	0.450876732
cg09994035	C2orf54;	2	0.00293735	0.34423872	0.316602541	0.489635725
cg12789935	C2orf54;	2	0.00081925	0.20765937	0.204811632	0.381258334
cg16171392	C2orf54;	2	0.00067897	0.52740499	0.574225467	0.72722594
cg08517455	C2orf55	2	0.00552473	0.51628302	0.525939204	0.654169636
cg10481134	C2orf58	2	0.04601984	0.31094464	0.282654215	0.464831952
cg04276057	C2orf82	2	0.0019386	0.17838869	0.188485248	0.299965849
cg13047596	C2orf82;	2	0.00258141	0.4037787	0.419426364	0.532085446
cg06436504	C3orf26;	3	0.00637687	0.49481111	0.516104103	0.645944487
cg00447769	C3orf62	3	0.048539	0.66058801	0.670443161	0.78801851
cg22690884	C3orf74	3	0.00088355	0.45432523	0.499606875	0.648207133
cg26044340	C4BPA	1	0.02995719	0.51865569	0.441940392	0.626324945
cg26430305	C4BPA	1	0.02541872	0.18296157	0.194859109	0.312502166
cg14740771	C4BPB;C4	1	0.008246	0.19774815	0.187159931	0.351579584
cg06005199	C4orf23	4	0.00076471	0.39640787	0.480248855	0.706908383
cg11036672	C4orf23	4	0.00083329	0.17590111	0.210201903	0.379862475
cg09774649	C4orf23	4	0.00159903	0.12614285	0.134863514	0.275944455
cg15466157	C4orf44;	4	0.000693	0.67908639	0.744417402	0.873302041
cg23210109	C4orf44;	4	0.00367311	0.3760931	0.338108186	0.53767038
cg01242977	C5orf13;	5	0.00294138	0.49700718	0.505918166	0.643497693
cg08927631	C5orf33;	5	0.00178771	0.26808115	0.360727783	0.494216911
cg13011452	C6orf136	6	0.00351539	0.31393886	0.312432053	0.424445606
cg19117070	C6orf145	6	0.00402744	0.54398932	0.54062056	0.68507535
cg07241170	C6orf145	6	0.0122594	0.27165002	0.222773015	0.384525896
cg00786256	C6orf147	6	0.01096356	0.73996493	0.708582419	0.844904526
cg24756966	C6orf154	6	0.0017228	0.53308302	0.551180666	0.669168784

cg11523799	C6orf154;	6	0.00180881	0.47466023	0.451914759	0.635118518
cg25658884	C6orf163;	6	0.04685951	0.29207385	0.304561539	0.4065904
cg17767198	C6orf170	6	0.00118031	0.67917036	0.736350131	0.868039768
cg16672203	C6orf27	6	0.00079512	0.40104492	0.518784428	0.63782232
cg21032074	C6orf94	6	0.00186737	0.21118781	0.195664748	0.485243592
cg21863721	C6orf94;L	6	0.00314038	0.65529985	0.680477515	0.800418481
cg04099158	C7orf10	7	0.03585699	0.19358991	0.162310848	0.340694558
cg24921614	C7orf20	7	0.00137524	0.42855846	0.422802729	0.539142247
cg25795026	C7orf20	7	0.00207648	0.26969806	0.212534144	0.37372099
cg11177223	C7orf20	7	0.00488333	0.3425397	0.310897183	0.507794634
cg11360546	C7orf50;C	7	0.00065949	0.35075487	0.462992405	0.630675806
cg02332902	C7orf50;C	7	0.00075144	0.79121245	0.82021996	0.9210842
cg09526129	C7orf50;C	7	0.00090417	0.58705952	0.608900686	0.856285905
cg01343703	C7orf50;C	7	0.00255186	0.53899045	0.565349042	0.715688115
cg04498913	C7orf50;C	7	0.00100054	0.39621437	0.401751356	0.526037195
cg21255128	C7orf50;C	7	0.00195613	0.45485123	0.390508002	0.601708505
cg08081346	C7orf50;C	7	0.001445	0.43334014	0.43077755	0.606432553
cg00364696	C7orf50;C	7	0.00870675	0.46541023	0.448096224	0.627333066
cg08626653	C7orf51	7	0.00103895	0.23916619	0.258354491	0.412694386
cg05686445	C7orf54;S	7	0.01820811	0.1717131	0.175073155	0.319898684
cg24777710	C7orf54;S	7	0.00164959	0.346334	0.453178453	0.622688568
cg11869184	C7orf61	7	0.00126235	0.61545462	0.632073826	0.75574769
cg01814565	C8orf34	8	0.03889249	0.32924667	0.341816051	0.483952355
cg13402635	C9orf3	9	0.00457044	0.1606299	0.128729926	0.30342932
cg21189849	C9orf3	9	0.00160732	0.35317532	0.396878303	0.512303556
cg14503441	C9orf3	9	0.00126816	0.58780574	0.578905364	0.725010419
cg00542880	C9orf3	9	0.00335524	0.4295827	0.430696252	0.611742653
cg14375632	C9orf3	9	0.02830078	0.52886428	0.527785444	0.63260571
cg13853813	C9orf3	9	0.0097835	0.20122929	0.202175432	0.358158356
cg14601621	C9orf3	9	0.00137723	0.30806657	0.310986588	0.45075324
cg13972491	C9orf3	9	0.00081711	0.25975829	0.274499042	0.422003832
cg23598352	C9orf46	9	0.01640621	0.30252004	0.342569507	0.442951704
cg06788565	C9orf69	9	0.00405622	0.24277292	0.221302477	0.383183876
cg14224203	C9orf86;C	9	0.00126535	0.15763515	0.189086767	0.293851099
cg06557985	CA10;CA1	17	0.00804549	0.57791424	0.568707053	0.727473027
cg02190618	CA5A	16	0.03956605	0.44463887	0.471055767	0.583589736
cg06948222	CA5A	16	0.01512979	0.40490186	0.379875642	0.524808188
cg25919221	CA6	1	0.00349176	0.29157237	0.368278274	0.541714223
cg04874511	CAB39L;C	13	0.00387834	0.54378956	0.500834712	0.69784639
cg27440150	CABIN1	22	0.00124625	0.48531917	0.478858518	0.656111599
cg18793086	CABLES1;	18	0.02199601	0.24685436	0.181643828	0.36088971
cg19075717	CACNA1G	17	0.00193169	0.31220706	0.376475676	0.485795731
cg14585054	CACNB1;C	17	0.00643366	0.22110254	0.177616853	0.380377771
cg15828524	CACNB3	12	0.01269426	0.54729232	0.507332924	0.65800406
cg16922523	CADM1;C	11	0.00488665	0.26709057	0.370002376	0.499600975
cg11425149	CALD1;CA	7	0.00967401	0.19609011	0.18020382	0.381609477

cg25418947	CALD1;CA	7	0.00449495	0.39999986	0.455961087	0.587701304
cg09707038	CALM2	2	0.0086408	0.46128252	0.496773655	0.613170214
cg19513232	CAMK2A;	5	0.0022221	0.28517745	0.29252432	0.431403838
cg05379597	CAMK2G;	10	0.00073378	0.12196488	0.150408421	0.260104096
cg25834768	CAMKK1;	17	0.01890283	0.27416661	0.269878597	0.417338255
cg01643090	CAMTA1	1	0.00375204	0.56818179	0.571381178	0.721602342
cg01912298	CAMTA2;	17	0.03524554	0.26916741	0.249317258	0.386896593
cg27090078	CAPG	2	0.00112793	0.43672196	0.464076059	0.63598403
cg06970290	CAPN1	11	0.00263491	0.46223366	0.48605351	0.657845767
cg09620688	CAPN1	11	0.01216006	0.33376025	0.296995512	0.435633972
cg04962005	CAPN1	11	0.00541575	0.28829744	0.263717846	0.448836536
cg01712700	CAPN10;C	2	0.00204904	0.24510817	0.238544484	0.406022812
cg10078335	CAPN10;C	2	0.00134844	0.3930997	0.408540374	0.527040654
cg14086599	CAPN10;C	2	0.00178025	0.2021347	0.19161523	0.367407405
cg16615776	CAPN10;C	2	0.00090002	0.27386015	0.299319003	0.572994062
cg22428691	CAPN2;CA	1	0.00070134	0.48836337	0.538182097	0.652605398
cg04652208	CAPN8	1	0.00296695	0.3813739	0.391682313	0.64775092
cg19807694	CAPN8	1	0.02438027	0.24963669	0.278497443	0.423983648
cg15119640	CAPN8	1	0.03270435	0.48031943	0.480853377	0.59378236
cg27235315	CAPN8	1	0.01289231	0.47103427	0.522446565	0.644209441
cg13545089	CAPS;CAF	19	0.00119385	0.45005878	0.446461707	0.620288465
cg07535740	CAPS;CAF	19	0.00401824	0.27438791	0.242891788	0.39299633
cg12763668	CAPZB	1	0.013012	0.19750957	0.166351005	0.32978508
cg17786902	CAPZB	1	0.01751915	0.26019615	0.230638275	0.369444047
cg03534655	CARD11	7	0.02289623	0.58440106	0.605715233	0.708345743
cg14035368	CARD9;CA	9	0.00806542	0.29531507	0.265668539	0.406268532
cg21664614	CARKD	13	0.00177922	0.46296319	0.460138877	0.594799691
cg02922734	CARKD	13	0.01183117	0.53050028	0.567486365	0.691162817
cg25709335	CARKD	13	0.00637687	0.47437052	0.518893545	0.643550871
cg17155330	CARKD	13	0.0154863	0.63354306	0.61303881	0.768837605
cg02321885	CARM1	19	0.00925638	0.35046984	0.336843073	0.491571384
cg08862742	CARS;CAF	11	0.01164121	0.24116908	0.224238699	0.424379765
cg14312661	CARS;CAF	11	0.00854392	0.14092511	0.129796834	0.283870263
cg10993865	CARS2	13	0.00674191	0.66579118	0.665219714	0.802841168
cg15597662	CASC3	17	0.02551242	0.40259779	0.360647692	0.519004609
cg25525709	CASKIN2;	17	0.00152405	0.68377014	0.679866614	0.82144906
cg05446253	CASKIN2;	17	0.00317348	0.2979832	0.284220984	0.487720067
cg09457470	CASKIN2;	17	0.00401094	0.47290001	0.437662535	0.613832625
cg09662798	CASKIN2;	17	0.00194569	0.25227287	0.255466435	0.42576153
cg19405868	CASP1;CA	11	0.00126535	0.62391413	0.691028629	0.836921129
cg01128042	CASP7;CA	10	0.02766761	0.25239554	0.250217077	0.484788843
cg26194092	CASP9;CA	1	0.0097835	0.54592323	0.563991266	0.669523349
cg15064681	CASQ2	1	0.00320302	0.43416429	0.524927337	0.675186055
cg17158913	CASZ1;CA	1	0.00395839	0.32685486	0.308949423	0.431278509
cg03845745	CBR4	4	0.02001663	0.42747071	0.432061753	0.57966549
cg09257178	CBWD2;L	2	0.00168849	0.4092642	0.407714624	0.577013572

cg05738585	CBX6	22	0.00337131	0.33647542	0.324378318	0.455217205
cg12580580	CCDC109.	10	0.00905411	0.40905724	0.397126146	0.530395195
cg18251984	CCDC144	17	0.0181634	0.53842393	0.530628023	0.672984836
cg21646082	CCDC21	1	0.01666517	0.32411234	0.32723708	0.435389598
cg11134246	CCDC24	1	0.00194312	0.35838561	0.378752789	0.531352491
cg13389211	CCDC40	17	0.00242555	0.30140587	0.320500909	0.524218486
cg23197939	CCDC40	17	0.00303201	0.54947082	0.610406411	0.771749319
cg15394558	CCDC42;C	17	0.01206219	0.37254062	0.422353346	0.532954585
cg22110888	CCDC57	17	0.00225052	0.50446295	0.48031743	0.632306604
cg12879038	CCDC57	17	0.0167445	0.32159635	0.319179897	0.481713529
cg26507988	CCDC57	17	0.01487501	0.54115397	0.532706923	0.668054445
cg02967812	CCDC57	17	0.00886166	0.4785976	0.446323891	0.604884608
cg11935831	CCDC57	17	0.00280849	0.36959766	0.368857575	0.488566041
cg17751591	CCDC57	17	0.00127437	0.51660205	0.463208043	0.635909673
cg12113740	CCDC68;C	18	0.02512074	0.41319804	0.402673499	0.557519703
cg11657323	CCDC88B	11	0.00184403	0.18452789	0.168498628	0.317965295
cg15175716	CCDC88B	11	0.00405622	0.32293458	0.31824077	0.43972049
cg05233289	CCDC88B	11	0.00547327	0.34529312	0.332817462	0.482715588
cg00156230	CCM2;CC	7	0.00971274	0.1522073	0.13007092	0.277589138
cg09637363	CCND1	11	0.00151706	0.26826899	0.264952781	0.372649275
cg06539449	CCND1	11	0.00116059	0.29196314	0.296423912	0.532439744
cg04717045	CCND1	11	0.00220693	0.40032586	0.407466577	0.619007153
cg12263469	CCND1	11	0.00183659	0.50590609	0.490545075	0.625761243
cg11802013	CCND1	11	0.01717855	0.54194939	0.557432401	0.658686713
cg12594237	CCND2	12	0.0009934	0.61761727	0.666383523	0.828692178
cg00417288	CCND2	12	0.0010753	0.53285088	0.544416155	0.796746885
cg02950701	CCNY;CCN	10	0.00804549	0.27901245	0.253315368	0.433218099
cg09033997	CCR9;CCF	3	0.00452348	0.74506465	0.737917197	0.857349781
cg24508095	CD151;CD	11	0.00357923	0.48006077	0.481968734	0.600003547
cg08614201	CD160	1	0.00096946	0.51782185	0.564334845	0.713779384
cg15166089	CD164L2	1	0.00757332	0.49323671	0.492219417	0.594935571
cg07458509	CD320;CD	19	0.02630662	0.26244861	0.2381221	0.373436844
cg17393635	CD37;CD3	19	0.00129968	0.3168772	0.321756223	0.442798806
cg06448249	CD79A;CD	19	0.0373326	0.40635201	0.435450253	0.544559511
cg07813265	CDC14A;C	1	0.01838071	0.36849492	0.351922057	0.472100317
cg04715649	CDC42BP	14	0.00736725	0.17618942	0.16001853	0.286921159
cg14267099	CDC42BP	11	0.02001663	0.47336934	0.42778081	0.585975893
cg27613998	CDC42EP:	2	0.01653351	0.36603105	0.368647696	0.520659469
cg05236720	CDC42EP:	2	0.0042711	0.37012051	0.360762402	0.539796695
cg15703377	CDC42EP4	17	0.00106734	0.57553163	0.533214314	0.751583154
cg27065254	CDC42EP4	17	0.00125711	0.31046466	0.289018049	0.468181433
cg24527183	CDC42SE:	5	0.01124375	0.50610534	0.557405849	0.673695292
cg08271366	CDH13	16	0.01163705	0.14784627	0.132976588	0.279556773
cg01093138	CDH13	16	0.01760022	0.46769728	0.4777301	0.641546844
cg05323683	CDH17;CD	8	0.00756169	0.14975368	0.129632961	0.292079003
cg13714026	CDH23;CD	10	0.00459107	0.18276063	0.178947476	0.343125876

cg19615295	CDH26;CI	20	0.00306113	0.24833768	0.223565094	0.441057944
cg05376601	CDH3	16	0.00753069	0.50162837	0.537507792	0.661143684
cg05086443	CDH5	16	0.00188718	0.45738698	0.48853791	0.594368044
cg01814495	CDIPT	16	0.00546217	0.44349157	0.406054566	0.56819096
cg14139051	CDK14	7	0.02555878	0.3514343	0.273751133	0.452232056
cg24060173	CDK18;CI	1	0.00218799	0.19892795	0.203785911	0.303985059
cg01720722	CDK18;CI	1	0.00110703	0.1840805	0.200982428	0.331109723
cg13189671	CDK2AP1	12	0.00318268	0.6924246	0.692975641	0.793183291
cg14948795	CDK2AP1	12	0.00710215	0.27012834	0.242554285	0.384008761
cg04270264	CDK5RAP	20	0.01838071	0.25704993	0.260762687	0.412907235
cg17405178	CDKAL1	6	0.02670405	0.239977	0.287110346	0.39272284
cg05426966	CDKL2	4	0.02129269	0.2865727	0.263419624	0.396714893
cg02953912	CDKN1C;(	11	0.00936285	0.5701751	0.497148041	0.677049247
cg23419490	CDKN1C;(	11	0.00439411	0.56047999	0.496777186	0.701288016
cg01838596	CDYL;CDY	6	0.00236882	0.3551105	0.30347485	0.52946996
cg04148753	CDYL;CDY	6	0.00252833	0.45551501	0.483877502	0.650623758
cg01510990	CDYL;CDY	6	0.03236546	0.26155698	0.25275075	0.362538861
cg04963575	CELA1	12	0.00701189	0.53273719	0.525934448	0.634040392
cg19437325	CELSR1	22	0.02568798	0.15791792	0.17631828	0.278596425
cg26456021	CEMP1;A	16	0.028548	0.56547469	0.581710053	0.704386785
cg22595230	CENPB	20	0.02251946	0.20632685	0.21075881	0.31395214
cg05922384	CFH	1	0.00283173	0.660745	0.625364737	0.81501263
cg16767221	CGNL1	15	0.00131453	0.32788682	0.412522356	0.612962059
cg07981370	CHAC1;CH	15	0.00704609	0.64927575	0.641068993	0.753264273
cg22608534	CHADL	22	0.00146177	0.32164673	0.338175382	0.501892871
cg16195245	CHADL	22	0.00327239	0.16081254	0.156752967	0.261693435
cg27570892	CHCHD4;(	3	0.00273355	0.45504046	0.476336955	0.606993187
cg04031656	CHD9	16	0.00165917	0.16794604	0.167058504	0.279282033
cg27567495	CHDH	3	0.00649	0.22023958	0.224298725	0.350405629
cg04071104	CHDH	3	0.00459107	0.1560914	0.145102981	0.306118485
cg21232015	CHFR;CHI	12	0.00336646	0.21603936	0.213076968	0.393332421
cg18451156	CHFR;CHI	12	0.00429038	0.30354449	0.305222104	0.47551704
cg20066677	CHFR;CHI	12	0.01465773	0.37057456	0.380972553	0.533448467
cg24532476	CHFR;CHI	12	0.01788219	0.34794729	0.323767084	0.484646581
cg04002957	CHID1;CH	11	0.01414127	0.74952414	0.745466059	0.875038613
cg13318256	CHKA;CHI	11	0.00499723	0.2382817	0.212250327	0.404066503
cg16087630	CHKA;CHI	11	0.00077468	0.52691608	0.612906388	0.717674682
cg08723459	CHMP1A;	16	0.00151706	0.60452583	0.580920504	0.731025712
cg01497921	CHMP1A;	16	0.01111549	0.42683078	0.41020181	0.560449403
cg25706861	CHPF	2	0.02389893	0.64467922	0.644951695	0.76554619
cg18406852	CHST10	2	0.00898993	0.19407378	0.191641673	0.34214234
cg11117177	CHST11	12	0.00471011	0.32946955	0.354583149	0.499051187
cg16255541	CHST12	7	0.00285469	0.68760101	0.687934537	0.801495179
cg19698242	CHST3	10	0.00211366	0.14841173	0.152594043	0.305144974
cg14496909	CHSY1	15	0.00217021	0.50757077	0.52179951	0.676085196
cg13902101	CHSY1	15	0.00521564	0.42157541	0.421179985	0.636902815

cg24927646	CHSY1	15	0.02103552	0.22928027	0.244599359	0.406197014
cg08080395	CILP	15	0.02252189	0.61247983	0.627160712	0.736900855
cg23323879	CILP	15	0.01861071	0.31543542	0.348220956	0.467548266
cg13356370	CILP	15	0.01206219	0.31122074	0.359948693	0.495567972
cg25896785	CILP	15	0.01828536	0.59867051	0.617835316	0.727077335
cg26635955	CILP2	19	0.00237009	0.14055594	0.15729962	0.270066605
cg03186620	CILP2	19	0.00591291	0.25107169	0.242770076	0.385506966
cg03339668	CIT	12	0.00799281	0.26282448	0.209032983	0.391560074
cg26097051	CIT	12	0.01665552	0.27647429	0.237665609	0.380183394
cg07180571	CLASP1;C	2	0.04937176	0.28310535	0.2507386	0.402489992
cg10950615	CLASP1;C	2	0.00103697	0.63761246	0.689961284	0.80244674
cg16591681	CLCNKA;(	1	0.0012026	0.27799879	0.301018824	0.437940004
cg09808075	CLCNKB	1	0.01502763	0.30226234	0.260432849	0.459397966
cg09413450	CLCNKB	1	0.00569156	0.2036163	0.209917027	0.320652856
cg21660130	CLCNKB	1	0.03760037	0.31579187	0.269996035	0.488093131
cg11744304	CLDN15	7	0.0063774	0.30790289	0.264253557	0.410886817
cg02512860	CLDN15	7	0.00131467	0.37274315	0.395841276	0.510978254
cg27507254	CLDN19;(	1	0.01446513	0.34836238	0.328231413	0.450522233
cg08082763	CLEC16A	16	0.00128133	0.25847999	0.280352715	0.42610675
cg00027155	CLEC16A	16	0.02009829	0.51195306	0.460204019	0.664082192
cg26597734	CLEC16A	16	0.00116579	0.13564465	0.153492695	0.338024408
cg08166431	CLEC16A	16	0.01705178	0.39247368	0.416161303	0.519443813
cg06636831	CLEC3B	3	0.00515676	0.22395724	0.220549989	0.338154452
cg13423383	CLIC3	9	0.01248948	0.19585096	0.169737648	0.299941511
cg17878351	CLIC5	6	0.02388672	0.6347493	0.634212372	0.739666714
cg03058520	CLK3;CLK	15	0.00318676	0.56966747	0.540857881	0.691894063
cg17808195	CLRN1;CL	3	0.01750308	0.43146487	0.478216997	0.594993655
cg19981828	CLRN1;CL	3	0.01272761	0.32717421	0.344194255	0.459467186
cg12182284	CLTB;CLT	5	0.00704609	0.40546052	0.372124632	0.546778079
cg24911827	CLTCL1;C	22	0.00260155	0.27545802	0.296430929	0.408203294
cg03129774	CLUAP1	16	0.00285259	0.12925798	0.132075366	0.34185977
cg07751214	CLUAP1;(	16	0.00597821	0.44627787	0.508916798	0.623269187
cg04963697	CLUAP1;(	16	0.04030438	0.16065139	0.133455463	0.262164919
cg27300028	CLYBL	13	0.02016391	0.52379113	0.560999501	0.684634939
cg24852561	CLYBL	13	0.00176484	0.50071587	0.488739214	0.652848963
cg23284609	CMBL	5	0.00183809	0.32742278	0.377453619	0.515120649
cg05705335	CMIP	16	0.00654696	0.31838435	0.278997035	0.420012908
cg16353318	CMIP;CM	16	0.00066232	0.34762364	0.431372714	0.577492543
cg01761552	CMIP;CM	16	0.01825754	0.49362146	0.465643266	0.638516261
cg03122201	CMIP;CM	16	0.00775785	0.24811584	0.201136488	0.383355181
cg06896529	CMIP;CM	16	0.01817641	0.28876916	0.229208165	0.393470326
cg09963814	CMIP;CM	16	0.003807	0.25042237	0.21231073	0.363284125
cg08682723	CMIP;CM	16	0.00382061	0.3063223	0.297534044	0.442169093
cg02053054	CMTM1;(	16	0.02753865	0.55501427	0.438862648	0.66077393
cg05404009	CMTM4;(	16	0.00756169	0.17742999	0.133303891	0.283558024
cg06715352	CMTM4;(	16	0.00174928	0.58177583	0.672266146	0.781926878

cg22843111	CMYA5	5	0.02841543	0.2182324	0.161191787	0.355625228
cg05226518	CNIH	14	0.00209505	0.35210222	0.362763675	0.601489397
cg27269110	CNKSR3	6	0.00119385	0.36852215	0.421689508	0.640403627
cg16175941	CNKSR3	6	0.00184921	0.2326764	0.28977731	0.466569994
cg13777287	CNKSR3	6	0.02794807	0.24912201	0.256974302	0.362056468
cg12448539	CNN3	1	0.00352201	0.34509817	0.380445417	0.578802737
cg19164761	CNN3	1	0.00958772	0.24515899	0.248306499	0.400081699
cg26096304	CNOT6	5	0.02794807	0.40824857	0.424355829	0.550569608
cg16563470	CNP	17	0.01430792	0.17327772	0.154374511	0.280483677
cg21692620	CNTNAP1	17	0.00293735	0.11778556	0.141919096	0.248252416
cg07871971	CNTNAP1	17	0.00941939	0.52067527	0.498827234	0.642839042
cg10524687	COBL	7	0.01677529	0.55648267	0.560497138	0.686398996
cg04723449	COG7	16	0.00122881	0.41392572	0.459534729	0.602558067
cg02890435	COL11A2	6	0.00081413	0.32242304	0.354197366	0.462568164
cg26867987	COL11A2	6	0.00436875	0.20757598	0.200170969	0.327744253
cg04249605	COL11A2	6	0.00131641	0.27105776	0.295729339	0.467515632
cg12881453	COL11A2	6	0.00175728	0.16449203	0.171670568	0.30510631
cg08077736	COL11A2	6	0.00124152	0.20383727	0.233035324	0.389364517
cg19887293	COL11A2	6	0.00155292	0.31471809	0.361504692	0.478815782
cg17790159	COL11A2	6	0.00233298	0.18351899	0.20518166	0.315553532
cg02667414	COL11A2	6	0.00074016	0.56736933	0.609466963	0.715835528
cg03500646	COL11A2	6	0.00296695	0.22258792	0.230591908	0.36468008
cg25482983	COL11A2	6	0.00777499	0.25311678	0.274459214	0.395608086
cg22748722	COL11A2	6	0.00069166	0.43115949	0.499373401	0.675299966
cg21205776	COL11A2	6	0.00065949	0.29968003	0.405146404	0.601654109
cg00514307	COL11A2	6	0.00234919	0.55277917	0.56239716	0.711208133
cg15173443	COL11A2	6	0.00065949	0.37914343	0.456355913	0.649702046
cg23912266	COL11A2	6	0.0009934	0.342315	0.425257478	0.541027922
cg01320908	COL11A2	6	0.00129968	0.55421663	0.597286199	0.744931375
cg00920162	COL11A2	6	0.00177694	0.3226058	0.377561023	0.547160474
cg18384402	COL11A2	6	0.00142756	0.51443716	0.523630816	0.671611501
cg22361816	COL11A2	6	0.00065949	0.37464191	0.46649034	0.568569478
cg00936309	COL11A2	6	0.00076471	0.33802436	0.427604173	0.531184534
cg15818109	COL11A2	6	0.00065949	0.23836774	0.354486794	0.540993045
cg00866054	COL11A2	6	0.00134532	0.25440029	0.284691869	0.416285111
cg05738403	COL11A2	6	0.00077183	0.32321323	0.354752832	0.484891375
cg16139380	COL11A2	6	0.00078819	0.37667749	0.441734135	0.573356738
cg20427378	COL11A2	6	0.00065949	0.22358249	0.289310594	0.391410294
cg12228461	COL11A2	6	0.00227382	0.28081166	0.311119827	0.496062845
cg17636806	COL11A2	6	0.00108672	0.33205829	0.376204742	0.48441119
cg27590742	COL11A2	6	0.00088728	0.66205741	0.699797364	0.818722911
cg12312338	COL11A2	6	0.00431388	0.33421136	0.327172763	0.4410494
cg08733307	COL11A2	6	0.00745134	0.20624434	0.183714558	0.329625583
cg14683730	COL11A2	6	0.00159903	0.17090595	0.187678077	0.368447891
cg12947757	COL11A2	6	0.00068904	0.30393093	0.347111776	0.517593088
cg26322749	COL11A2	6	0.00074016	0.41751152	0.491789366	0.691565446

cg18375610	COL11A2	6	0.00065949	0.43667206	0.525677317	0.700421003
cg22161893	COL11A2	6	0.00070285	0.3822725	0.484239417	0.667705476
cg12061370	COL11A2	6	0.00107089	0.26183752	0.335802872	0.510108123
cg24311693	COL11A2	6	0.00126816	0.25535762	0.278167592	0.527983076
cg09261794	COL11A2	6	0.0012504	0.17011996	0.238773093	0.399383734
cg18624108	COL11A2	6	0.00065949	0.50878378	0.564396212	0.691719642
cg10736955	COL11A2	6	0.00074801	0.40568241	0.509282203	0.704196677
cg14466923	COL11A2	6	0.00142756	0.31116894	0.348532149	0.473603976
cg01412022	COL11A2	6	0.00149569	0.2149322	0.239002186	0.448089331
cg02230225	COL11A2	6	0.00098847	0.3643795	0.427500625	0.644226451
cg07457375	COL11A2	6	0.00131453	0.1338013	0.145865811	0.315279105
cg13617209	COL11A2	6	0.00153162	0.20818605	0.260423315	0.429684276
cg03260211	COL11A2	6	0.00092849	0.279749	0.337904098	0.479920885
cg20269729	COL11A2	6	0.000777	0.29522659	0.372239779	0.566994594
cg26981781	COL11A2	6	0.00074801	0.3092619	0.35618754	0.525106002
cg09502339	COL11A2	6	0.00112679	0.25441634	0.329982749	0.495486461
cg00501482	COL11A2	6	0.0020498	0.3607626	0.485198715	0.58857367
cg15239679	COL11A2	6	0.00646891	0.20036313	0.206283414	0.344015463
cg01194674	COL11A2	6	0.00240796	0.16533001	0.180129112	0.333682432
cg20798604	COL11A2	6	0.00078196	0.21569234	0.296676371	0.430900014
cg17596257	COL11A2	6	0.00352201	0.3810836	0.477383614	0.593663845
cg04653627	COL11A2	6	0.00144989	0.47674044	0.548846111	0.654625181
cg18671115	COL11A2	6	0.00104158	0.26565772	0.358068944	0.568416809
cg04182805	COL11A2	6	0.00111057	0.43578625	0.476654745	0.660715866
cg01806857	COL11A2	6	0.00147518	0.18613224	0.219611986	0.37186747
cg24864639	COL11A2	6	0.00068904	0.25538975	0.313218622	0.449612298
cg17591573	COL11A2	6	0.00240535	0.21584743	0.217322949	0.444483663
cg03790047	COL11A2	6	0.00079512	0.37195145	0.442477033	0.621520307
cg21509966	COL11A2	6	0.00248997	0.30928658	0.336994811	0.518932706
cg23704085	COL11A2	6	0.0013522	0.14655169	0.184515674	0.356122497
cg11835806	COL11A2	6	0.00081789	0.24944368	0.297676546	0.493797823
cg01680268	COL11A2	6	0.00179679	0.16241714	0.171054193	0.35794721
cg00505762	COL11A2	6	0.00207996	0.17869348	0.188975589	0.347181819
cg23231727	COL11A2	6	0.00126804	0.13627571	0.157682688	0.259970303
cg22501449	COL11A2	6	0.00077468	0.18111971	0.231444269	0.404461693
cg03783258	COL11A2	6	0.00117602	0.17645487	0.210172813	0.375650905
cg11411509	COL11A2	6	0.00878866	0.17264505	0.172898008	0.359805551
cg19628686	COL11A2	6	0.00917376	0.19700322	0.211731299	0.364875453
cg12472351	COL11A2	6	0.00660543	0.13580206	0.139122887	0.250161861
cg15089846	COL12A1	6	0.00388546	0.64498422	0.661466718	0.775695825
cg20436086	COL13A1	10	0.0018139	0.21174131	0.232758004	0.424755164
cg24488229	COL16A1	1	0.00262147	0.21622937	0.228695766	0.342394658
cg08930131	COL23A1	5	0.04063967	0.2408224	0.257278882	0.364429036
cg04011940	COL25A1	4	0.04706253	0.26166853	0.26944818	0.408328566
cg03758241	COL27A1	9	0.00193378	0.16912999	0.158978511	0.304264016
cg04382108	COL27A1	9	0.00427027	0.2288486	0.2095251	0.332456949

cg13427810	COL27A1	9	0.00161582	0.33775733	0.408366975	0.640910324
cg14031246	COL27A1	9	0.00175545	0.5926804	0.568498429	0.697720503
cg15108410	COL2A1;C	12	0.00302211	0.22995589	0.245166646	0.387649026
cg15465823	COL2A1;C	12	0.00644172	0.17315113	0.140043358	0.322818153
cg08715466	COL2A1;C	12	0.00131453	0.23422582	0.269931999	0.452844337
cg10502244	COL2A1;C	12	0.00314038	0.20966422	0.217125499	0.338733519
cg01906695	COL2A1;C	12	0.00281543	0.22681578	0.253368334	0.36614509
cg08234256	COL4A1	13	0.00894518	0.20262014	0.201556956	0.3262388
cg00050792	COL4A1	13	0.00565829	0.1855926	0.184257114	0.305058349
cg13618741	COL5A1	9	0.01060969	0.24830214	0.239427538	0.371782826
cg13567205	COL5A1	9	0.00548311	0.53778712	0.51749637	0.641646076
cg22247792	COL6A2;C	21	0.0017241	0.4443887	0.480733147	0.660354032
cg20502977	COL6A3;C	2	0.02443534	0.38090934	0.310852486	0.533479046
cg03878567	COL9A1;C	6	0.00478553	0.30038852	0.285650779	0.533087464
cg10210510	COL9A2	1	0.00532167	0.29705515	0.359997861	0.470966133
cg03059921	COL9A3	20	0.00109888	0.27974865	0.295686395	0.449659539
cg07305723	COL9A3	20	0.00189637	0.44362576	0.459735766	0.569501409
cg06598544	COL9A3	20	0.00358374	0.34232745	0.35179317	0.546339595
cg25597307	COLQ;CO	3	0.02728203	0.29155714	0.317373521	0.419389408
cg20950633	COLQ;CO	3	0.00539114	0.60407499	0.58990966	0.71796491
cg24888814	COPZ1	12	0.00568196	0.34501138	0.364732566	0.546959855
cg18957463	CORIN	4	0.00821138	0.15012454	0.10459919	0.263001741
cg12449974	CORO2B	15	0.00126208	0.24435464	0.226009923	0.565817899
cg01678309	CORO2B	15	0.00587243	0.19359447	0.192371964	0.310293205
cg02351655	CORO7	16	0.01342353	0.27789312	0.263612117	0.40046182
cg09069446	CORO7	16	0.00182121	0.35930345	0.368332259	0.469551526
cg20163085	CORT;API	1	0.02175305	0.57396972	0.581375548	0.683802022
cg24876069	CP	3	0.01738159	0.44273202	0.491901649	0.620601622
cg03951662	CPD	17	0.0103161	0.50267655	0.511832293	0.677756203
cg11043571	CPE	4	0.00894518	0.17778525	0.168055525	0.302082819
cg01708636	CPE	4	0.00626125	0.24704402	0.283077888	0.443444074
cg23427348	CPE	4	0.01585898	0.33327181	0.434819053	0.556794984
cg15341575	CPEB3	10	0.00911882	0.188087	0.184675183	0.354358696
cg16710448	CPSF3	2	0.01598777	0.58836293	0.555281756	0.703945658
cg03101058	CPSF4L	17	0.00139627	0.3647549	0.401732806	0.570820534
cg15211864	CPT1A;CF	11	0.01096356	0.22733213	0.203491596	0.391716302
cg04732324	CPT1A;CF	11	0.00184894	0.59247329	0.634071006	0.750945708
cg26906629	CPT1A;CF	11	0.00207771	0.41685032	0.435140292	0.596288948
cg20285002	CPT1A;CF	11	0.00218837	0.68720371	0.694954601	0.795077676
cg23729283	CPVL;CPV	7	0.00666111	0.43876363	0.492206442	0.635650036
cg19573230	CREB3L2	7	0.00127413	0.43233394	0.471778715	0.615563027
cg13016916	CREB3L2	7	0.03618565	0.17746054	0.194737511	0.296828922
cg13495373	CREB5;CF	7	0.00617894	0.34254815	0.375349911	0.50013264
cg07042532	CREB5;CF	7	0.00217021	0.39670956	0.407182087	0.551842442
cg14862787	CREB5;CF	7	0.0048909	0.57385679	0.566105162	0.692235317
cg12552104	CREB5;CF	7	0.00181052	0.26713612	0.293568726	0.483504717

cg20315150	CREB5;CR	7	0.00687165	0.44665745	0.452808283	0.632057621
cg22681186	CREB5;CR	7	0.01934763	0.42285467	0.448964606	0.589800888
cg20306842	CREB5;CR	7	0.00997311	0.16542385	0.177663599	0.307277654
cg09450153	CREB5;CR	7	0.0039383	0.23783731	0.265356331	0.381512368
cg21159940	CREB5;CR	7	0.00420024	0.21314888	0.198235343	0.39198291
cg19588408	CREB5;CR	7	0.0063774	0.31769741	0.268601681	0.47728336
cg19559984	CREB5;CR	7	0.01608519	0.30648015	0.279827654	0.441159955
cg04704487	CRISPLD2	16	0.00316669	0.1884569	0.175978987	0.367969173
cg09967633	CRISPLD2	16	0.00172495	0.38223642	0.430934396	0.626231264
cg13407975	CRLF1	19	0.00077582	0.0717728	0.112319367	0.221643428
cg09721078	CRLF3	17	0.0261165	0.54826416	0.568878343	0.697804499
cg14973240	CROCC	1	0.00905238	0.14206805	0.094494239	0.265777836
cg10558598	CROCCL1	1	0.0026668	0.29568072	0.261896219	0.407964721
cg25680629	CRTAP	3	0.01595775	0.15666047	0.127416171	0.263638876
cg07015183	CRTC1;CR	19	0.00379797	0.55397183	0.504528088	0.685145366
cg02961385	CRTC1;CR	19	0.00769304	0.26111586	0.231904376	0.429880932
cg01874562	CRTC1;CR	19	0.00186737	0.15416369	0.150225283	0.273876813
cg03073714	CRY1	12	0.03879786	0.47214615	0.463274184	0.6290148
cg08688043	CRYBA1	17	0.00379918	0.40335454	0.409275777	0.559917716
cg25221625	CRYBB3	22	0.00143965	0.56091982	0.561117567	0.687829221
cg03244494	CRYL1	13	0.01693242	0.3389497	0.431198468	0.534203057
cg24580724	CRYZ;CRY	1	0.01219095	0.28971169	0.235898526	0.425965931
cg18636739	CSDA;CSD	12	0.03477069	0.17324699	0.145375992	0.274061418
cg20704330	CSF2	5	0.00344347	0.53388013	0.509112099	0.652049075
cg00158770	CSGALNA	8	0.00077468	0.33571845	0.397007528	0.54844068
cg07169233	CSGALNA	8	0.00298275	0.36494621	0.468932679	0.611648315
cg12059540	CSGALNA	8	0.00425638	0.23296353	0.258246691	0.400384442
cg01129958	CSGALNA	8	0.00748334	0.38165481	0.384249231	0.590258615
cg06129556	CSNK1G1	15	0.00426288	0.32823725	0.334965793	0.587445036
cg06470424	CSNK1G2	19	0.00983482	0.26088584	0.222908334	0.376220795
cg05729827	CSPG4	15	0.00262388	0.46397274	0.456434608	0.580787682
cg05208483	CSPG4	15	0.00220808	0.45210462	0.420217633	0.606017692
cg06734519	CSPG4	15	0.00103895	0.55688347	0.567099478	0.739215628
cg21460582	CSPG4	15	0.00315764	0.11753816	0.11725306	0.232201135
cg03238581	CSPG4	15	0.00396544	0.09903118	0.0839921	0.229009462
cg14029169	CST3	20	0.00084118	0.55952114	0.646992	0.789923767
cg12525219	CTBP1;CT	4	0.00165724	0.60784579	0.590274726	0.709838957
cg09758273	CTBP1;CT	4	0.00429038	0.54501004	0.541387331	0.64501247
cg21373263	CTBP2;CT	10	0.03003784	0.41303969	0.478642943	0.60409638
cg17523415	CTBP2;CT	10	0.00330705	0.56524773	0.587607574	0.709457441
cg06240209	CTBP2;CT	10	0.00065949	0.55892212	0.723288333	0.825651386
cg07237540	CTDP1;CT	18	0.0034485	0.43980972	0.490463234	0.631236745
cg07753598	CTDSP1;C	2	0.00149569	0.49696769	0.411636824	0.653154766
cg03228931	CTDSP2	12	0.00809353	0.54674549	0.519450412	0.661951324
cg27606822	CTDSP2;N	12	0.00150566	0.62142544	0.613260384	0.735588179
cg13333722	CTDSPL;C	3	0.00131641	0.62399569	0.63742124	0.768907878

cg08171483	CTDSPL;C	3	0.0040421	0.12633301	0.117946114	0.237711776
cg05740575	CTHRC1	8	0.00430896	0.17404497	0.146047032	0.351373029
cg01156295	CTNNA1;I	5	0.01236318	0.36570642	0.31202839	0.527692615
cg11677683	CTNNA1;I	5	0.02108987	0.2274988	0.165265609	0.333640371
cg27004195	CTNNA2;(	2	0.01776628	0.24625083	0.261396247	0.383981526
cg12232373	CTNS;CTN	17	0.00452348	0.58293058	0.575495056	0.683966506
cg16118839	CTSC;CTS	11	0.00339981	0.39744581	0.482139688	0.607620007
cg08914150	CTTN;CTT	11	0.00173084	0.57784635	0.652963578	0.787963885
cg22720428	CTTN;CTT	11	0.00099363	0.26000364	0.363865586	0.512711005
cg02011981	CTTN;CTT	11	0.00597821	0.54787407	0.60705682	0.734782582
cg13096351	CTTN;CTT	11	0.03960534	0.1996842	0.169986282	0.310216297
cg12030523	CTXN3;CT	5	0.02111416	0.38531018	0.431321808	0.536790774
cg08581745	CUBN	10	0.00248015	0.17242828	0.158689083	0.36392348
cg06352464	CUGBP2;(	10	0.00405622	0.22684014	0.199458744	0.479698928
cg11086547	CUGBP2;(	10	0.02856411	0.28438467	0.333420645	0.434100057
cg20080282	CUL5	11	0.00334886	0.15940657	0.160883578	0.311425659
cg20567148	CUX1;CU)	7	0.00630045	0.30002299	0.210990121	0.440058654
cg24420432	CUX1;CU)	7	0.00471011	0.23856703	0.188481155	0.423927023
cg14466759	CUX1;CU)	7	0.00218799	0.22119723	0.223848237	0.48920694
cg26570844	CUX1;CU)	7	0.00118031	0.18462378	0.211562246	0.492550478
cg24183566	CUX1;CU)	7	0.02577962	0.24695751	0.200384172	0.359161426
cg02911387	CUX1;CU)	7	0.00089741	0.21285913	0.266418894	0.408212456
cg17472610	CUX1;CU)	7	0.00215533	0.22455299	0.238760973	0.364339862
cg07266412	CUX1;CU)	7	0.00247734	0.27371591	0.301920218	0.492604022
cg17713751	CUX1;CU)	7	0.00187837	0.44346045	0.423783099	0.586006478
cg12349350	CUX1;CU)	7	0.00169556	0.26157564	0.320247342	0.550803271
cg14203426	CUX1;CU)	7	0.00367708	0.37410037	0.407138687	0.560933892
cg18483404	CUZD1	10	0.00134844	0.25140381	0.285806473	0.466985769
cg05724197	CX3CL1	16	0.00669541	0.37258384	0.366076398	0.481895994
cg22908000	CXCR7	2	0.02720807	0.20489032	0.192410713	0.311783944
cg13707793	CXXC5	5	0.00466622	0.18240861	0.160542447	0.297754555
cg17306279	CXXC5	5	0.00367963	0.36252019	0.334137394	0.472833543
cg23167035	CXXC5	5	0.00219711	0.39261904	0.390085234	0.529546223
cg22741735	CXXC5	5	0.00513872	0.22277782	0.217637829	0.337447501
cg05227215	CXXC5	5	0.00555868	0.22783191	0.214251348	0.378362309
cg14876315	CXXC5	5	0.0009934	0.56227492	0.519843431	0.714024254
cg06905511	CXXC5	5	0.00206218	0.18703036	0.188351188	0.355033101
cg15290965	CYB5R2	11	0.00209505	0.18887607	0.179152314	0.336418905
cg02603251	CYFIP2;CY	5	0.0026958	0.34304349	0.362947886	0.518266891
cg04790755	CYHR1;CY	8	0.0065693	0.32669948	0.350429343	0.466029279
cg21181051	CYP20A1	2	0.00568196	0.26571171	0.307289411	0.478450354
cg20822767	CYP20A1	2	0.03960534	0.48004039	0.485785217	0.631594968
cg14577373	CYP20A1	2	0.00886166	0.17397854	0.188350592	0.315997286
cg04586286	CYP26B1	2	0.00152405	0.73314642	0.73180959	0.847280979
cg01959421	CYP26B1	2	0.00128725	0.13625342	0.14534416	0.340712324
cg00999152	CYP26B1	2	0.0082702	0.33850775	0.287734869	0.492931932

cg14447606	CYP26B1	2	0.0017637	0.09476764	0.11186234	0.21755167
cg09233204	CYP26C1	10	0.01160447	0.25248106	0.260309513	0.400852038
cg11234637	CYP26C1	10	0.0034485	0.31603835	0.333240327	0.44994767
cg04564481	CYP26C1	10	0.0020873	0.2591481	0.264922946	0.379661049
cg25083618	CYP26C1	10	0.00297171	0.21890693	0.212937026	0.327883939
cg20293609	CYR61	1	0.01725665	0.19876417	0.173708482	0.324492587
cg13562102	CYTH3	7	0.00554206	0.50341851	0.476232754	0.608280636
cg08687948	CYTH3	7	0.00261671	0.24367309	0.226777152	0.424959925
cg08893109	CYTH3	7	0.00160732	0.64177939	0.676303067	0.816841495
cg20492034	CYTH3	7	0.00134844	0.2428435	0.275461349	0.449397262
cg21045385	CYTSB;CY	17	0.01033353	0.47838538	0.514628536	0.645054678
cg05643545	CYTSB;CY	17	0.01980843	0.24367524	0.189177308	0.423201793
cg17370412	CYTSB;CY	17	0.03615697	0.20596721	0.204665257	0.323806713
cg20290367	CYTSB;CY	17	0.0103127	0.22730312	0.207215081	0.392055365
cg09818912	CYTSB;CY	17	0.01539932	0.32277832	0.357339658	0.524750802
cg17365725	D2HGDH	2	0.00388546	0.51761743	0.491262262	0.642245627
cg27351239	DAAM2	6	0.00337568	0.13802604	0.152053576	0.252650298
cg05609656	DAAM2	6	0.00193169	0.33321809	0.452192262	0.602604593
cg08465708	DAB1	1	0.03657758	0.57398779	0.609472066	0.722198777
cg14190522	DAB2IP	9	0.00302211	0.11514724	0.13226934	0.272725322
cg08906015	DACT3	19	0.00243597	0.19413698	0.157527197	0.373832347
cg06454084	DACT3	19	0.00661882	0.27766288	0.287574703	0.512956871
cg12472022	DAGLA	11	0.00845805	0.14449775	0.133068338	0.246909792
cg19633004	DAP	5	0.00594191	0.21260547	0.208175764	0.390022641
cg05473987	DAP	5	0.00076471	0.30959435	0.402606393	0.55283537
cg18801045	DAP	5	0.00109472	0.20586813	0.259833079	0.497735475
cg24754277	DAPK1	9	0.00223531	0.19197954	0.232804387	0.348276851
cg16447058	DAPK2	15	0.00878866	0.22401666	0.194035486	0.345364678
cg11775215	DAPK2	15	0.01507708	0.44384491	0.506424885	0.609479306
cg02872476	DBNDD1	16	0.0100567	0.36828686	0.313563893	0.511011769
cg26730025	DCAKD;D	17	0.01320626	0.1150828	0.115115057	0.217554969
cg16177647	DCAKD;D	17	0.00132036	0.34259924	0.374654993	0.613673645
cg12226735	DCBLD2	3	0.00469934	0.49586509	0.480978979	0.637423375
cg23173301	DCHS1	11	0.00367708	0.33313836	0.306216809	0.491690006
cg09605634	DCLK1	13	0.00440148	0.17646206	0.150417886	0.307782807
cg11325273	DCLK1	13	0.01435007	0.17298868	0.224434463	0.352131431
cg22806908	DCP1A	3	0.0012281	0.36703745	0.392166673	0.646724588
cg07039975	DCTN1;D(	2	0.0182655	0.31947507	0.311490956	0.440397791
cg20202112	DCTN1;D(	2	0.00857669	0.29268419	0.275763709	0.415760052
cg21238257	DCUN1D2	13	0.00334886	0.67573244	0.703196495	0.816848218
cg01145245	DCUN1D3	16	0.00989055	0.1458617	0.113635857	0.248588262
cg01820584	DCUN1D4	4	0.00092643	0.27174402	0.369721147	0.644850388
cg25095032	DDAH2	6	0.00334886	0.27374086	0.239611487	0.379815426
cg04074004	DDAH2	6	0.00737637	0.31995881	0.321363932	0.44442713
cg10771650	DDR1	6	0.00297143	0.35587136	0.35235087	0.54482447
cg19215110	DDR1;DD	6	0.01748472	0.25804948	0.23987511	0.379893566

cg16707952	DDR1;DD	6	0.00335022	0.44056608	0.497142751	0.634779233
cg12768993	DDX10	11	0.00783744	0.66119304	0.646014432	0.782259869
cg07194374	DDX24	14	0.00132036	0.40881473	0.462581589	0.641754288
cg12499311	DDX46	5	0.03931081	0.1674144	0.135440355	0.275774837
cg13569700	DEAF1	11	0.00367963	0.68229042	0.672017033	0.789853302
cg09375756	DEAF1	11	0.00369903	0.39946234	0.375262027	0.595887531
cg21156386	DEAF1	11	0.00329066	0.25604775	0.302552282	0.464528781
cg26894841	DEF8;DEF	16	0.03533938	0.41366376	0.40850249	0.523228503
cg14798653	DENND1C	19	0.00466622	0.28661886	0.271721394	0.395437266
cg08379738	DENND1C	19	0.02232449	0.33201591	0.300701675	0.468471949
cg08319291	DENND2A	7	0.00085355	0.59287188	0.60075195	0.702839334
cg07580128	DENND3	8	0.00184462	0.63640095	0.681097981	0.798815508
cg21442881	DENND3	8	0.00065949	0.21287069	0.351217216	0.537794669
cg02919712	DEPDC6	8	0.01059488	0.29242704	0.340261017	0.466236617
cg12744321	DFFB	1	0.01653406	0.43297309	0.41643697	0.538195566
cg17790129	DFNA5;DI	7	0.00798815	0.16438626	0.141659445	0.299361104
cg25577212	DFNA5;DI	7	0.00655853	0.16619857	0.160132705	0.292578948
cg13826055	DFNB31;I	9	0.00152405	0.44472118	0.458419175	0.736589891
cg23224619	DGKD;DG	2	0.02205285	0.34503295	0.351303715	0.482371161
cg25107522	DGKD;DG	2	0.00620309	0.16655459	0.127273948	0.273319467
cg08051105	DGKD;DG	2	0.00290605	0.46383916	0.473188241	0.579780017
cg15815084	DGKE	17	0.00138788	0.20797433	0.275189837	0.399415069
cg22718636	DGKQ	4	0.03515143	0.1866143	0.185840418	0.288005405
cg10523319	DHRS3	1	0.00076471	0.57015005	0.634998757	0.786236161
cg16432031	DHRS3	1	0.00485228	0.47433594	0.50127345	0.610456441
cg00352872	DHRS7C	17	0.00758031	0.15781421	0.154666724	0.282177084
cg14777519	DHX30;DI	3	0.00552473	0.3219801	0.314924573	0.474776929
cg20592691	DIAPH3;D	13	0.02833129	0.44295771	0.425027964	0.601776073
cg23899649	DICER1;D	14	0.00779924	0.63399621	0.61885261	0.765883363
cg10948630	DICER1;D	14	0.00192049	0.59459332	0.653954789	0.801618489
cg23488578	DICER1;D	14	0.00614688	0.15828085	0.145593354	0.260672086
cg13387972	DIP2C	10	0.00263125	0.53026159	0.535773593	0.651284888
cg17242812	DIP2C	10	0.02823023	0.65935981	0.594130693	0.824559144
cg25301103	DIP2C	10	0.0012838	0.4888182	0.60408024	0.813490187
cg11423998	DIP2C	10	0.00097509	0.50918532	0.594489491	0.780931381
cg09782637	DIP2C	10	0.00227742	0.43639176	0.521357628	0.729256294
cg13477178	DIP2C	10	0.0012504	0.16477255	0.203040301	0.366621301
cg20251943	DIP2C	10	0.00168532	0.24974176	0.285560142	0.428568211
cg15584954	DIP2C	10	0.01373528	0.53137363	0.565362502	0.674279194
cg04806237	DIP2C	10	0.00341932	0.62739918	0.701357609	0.860837383
cg08306029	DIP2C	10	0.00117894	0.57385464	0.638815593	0.771287215
cg05005968	DIP2C	10	0.00726745	0.13027951	0.10985874	0.251395737
cg20517784	DIP2C	10	0.00224923	0.49633925	0.480662804	0.716623346
cg17608529	DIP2C	10	0.00567752	0.3649349	0.315462136	0.493274401
cg26406407	DIP2C	10	0.00722201	0.45714615	0.465389313	0.573062351
cg04248461	DIP2C	10	0.00314361	0.21050461	0.182939712	0.313997962

cg24461627	DIP2C	10	0.036552	0.39607971	0.411603922	0.540272292
cg00590260	DIP2C	10	0.00488333	0.22092949	0.189141952	0.348734412
cg12531838	DIP2C	10	0.00953692	0.19090346	0.169706021	0.307762115
cg17905091	DIP2C	10	0.00844494	0.41173964	0.446150842	0.613821305
cg06492521	DIP2C	10	0.0231425	0.43692249	0.42345554	0.588883514
cg17861458	DIP2C	10	0.00446647	0.50259361	0.521011119	0.644105084
cg26511507	DIP2C	10	0.00176085	0.14403839	0.158775313	0.307552658
cg06156195	DIP2C	10	0.00319723	0.54450334	0.604143402	0.719611258
cg10441401	DIP2C	10	0.00225052	0.21905477	0.22599385	0.385400778
cg11081275	DIP2C	10	0.00555638	0.27333866	0.236077484	0.41872938
cg09961254	DIP2C	10	0.00852838	0.27253103	0.249591515	0.412590265
cg27159421	DIP2C	10	0.00247655	0.54835363	0.550330375	0.708438791
cg26782150	DIP2C	10	0.01177946	0.24905776	0.199621536	0.397786148
cg08784934	DIP2C	10	0.00103895	0.42894028	0.415214002	0.589873451
cg11468148	DIP2C	10	0.00714351	0.26786443	0.266595351	0.404926643
cg08079763	DIP2C	10	0.00065949	0.20450994	0.270471372	0.389136055
cg04720116	DIP2C	10	0.00337719	0.45526937	0.486945171	0.663813612
cg25297146	DIP2C	10	0.00074409	0.75863202	0.796543821	0.901099183
cg10494981	DIP2C	10	0.00838577	0.63230088	0.696629322	0.796855529
cg15713378	DIP2C;C1	10	0.00233734	0.45060537	0.453690117	0.598698984
cg13660126	DIP2C;C1	10	0.01847369	0.48539659	0.48231296	0.624489605
cg15516835	DIP2C;C1	10	0.00579556	0.17057806	0.151804677	0.288299426
cg17198320	DIP2C;C1	10	0.02630407	0.34199329	0.373431382	0.516808124
cg07469445	DIP2C;C1	10	0.02002229	0.20026534	0.21531231	0.339225421
cg16682227	DIRAS3	1	0.00664361	0.36120055	0.330019038	0.519835929
cg03803789	DIXDC1;D	11	0.00538904	0.21986211	0.158773045	0.357322816
cg18962750	DIXDC1;D	11	0.00213077	0.56166623	0.599134747	0.724930229
cg26148020	DLC1	8	0.02212773	0.22054979	0.194128343	0.36400123
cg16749785	DLC1;DLC	8	0.01443228	0.47778702	0.529144746	0.668878883
cg20607577	DLC1;DLC	8	0.03898484	0.41906895	0.449347266	0.578237512
cg17989952	DLC1;DLC	8	0.00796638	0.47667885	0.429794617	0.644867759
cg20733077	DLEU2	13	0.04190693	0.37833329	0.417897508	0.543779479
cg01404873	DLEU2	13	0.04172015	0.34933795	0.400197686	0.519747035
cg13489576	DLG1;DLC	3	0.00449495	0.61894662	0.638510577	0.750197453
cg24182581	DLG2;DLC	11	0.00433996	0.32086438	0.36828333	0.526569202
cg27328839	DLG5	10	0.00263491	0.61276183	0.642563291	0.763670657
cg23042540	DLGAP2	8	0.02047468	0.28154795	0.227901947	0.38589503
cg06432036	DLL1	6	0.00297078	0.59067675	0.621718017	0.726779463
cg14157570	DLL4	15	0.00390603	0.21799201	0.189129435	0.416482206
cg00204249	DNAH17	17	0.0024796	0.44372366	0.44531759	0.625787234
cg05361750	DNAH17	17	0.00644172	0.66269363	0.65472295	0.771183962
cg10161638	DNAJB6	7	0.00152273	0.32816639	0.34847944	0.499669695
cg16081609	DNAJC1	10	0.03174689	0.43332891	0.430018177	0.545681622
cg26544725	DNAJC1	10	0.02888438	0.7505124	0.727818198	0.852410473
cg25700533	DNAJC17,	15	0.00070678	0.0746193	0.095908638	0.254083547
cg11174089	DNAJC3	13	0.00384078	0.54634841	0.579648827	0.752344258

cg09764984	DNASE1	16	0.00367708	0.18503276	0.169594979	0.373521477
cg27552378	DNASE1	16	0.00248015	0.24400335	0.233296927	0.382191017
cg23009818	DNMT3A;	2	0.001223	0.20286479	0.236334925	0.339120425
cg20702417	DNMT3A;	2	0.00173053	0.49903571	0.522503134	0.624211092
cg06748978	DNMT3A;	2	0.00219764	0.31674545	0.351171366	0.523124031
cg27482168	DNPEP	2	0.03288793	0.36496765	0.355651441	0.476287065
cg26717418	DOCK1	10	0.00804549	0.47191312	0.443669627	0.584856616
cg02262162	DOCK3	3	0.01748472	0.65883735	0.593987201	0.799777489
cg05046083	DOCK4	7	0.00620216	0.2328488	0.302568886	0.429268226
cg16797901	DOCK4	7	0.00901377	0.16218202	0.142036109	0.271932602
cg11800635	DOK1	2	0.00077739	0.16762431	0.236444707	0.337581188
cg07280936	DOK7;DO	4	0.00491697	0.19533998	0.178423478	0.316220613
cg21725158	DOT1L	19	0.00679946	0.43462966	0.419040127	0.563125241
cg14293102	DOT1L	19	0.0007901	0.52872404	0.577232828	0.757674292
cg09350880	DPF3	14	0.01173837	0.3041515	0.275615263	0.408673777
cg01901381	DPF3	14	0.00103447	0.44797906	0.49127242	0.624300878
cg06776201	DPF3	14	0.03327853	0.24741386	0.232144473	0.365953828
cg23057232	DPYD	1	0.01083301	0.20980129	0.230482604	0.393432918
cg16649728	DRAP1;C1	11	0.00349176	0.28672318	0.27726874	0.397764768
cg14603539	DSCAM	21	0.00591291	0.29854688	0.297893028	0.427158348
cg22778797	DSCR8;DS	21	0.00543797	0.6242728	0.628607611	0.729230968
cg12561125	DTX3;GEF	12	0.01580038	0.33854448	0.329748695	0.458759069
cg03913136	DUSP14	17	0.01144141	0.73743837	0.732392868	0.860404406
cg14689456	DYSF;DYS	2	0.00595714	0.53155818	0.576159273	0.698144251
cg01171786	DYSF;DYS	2	0.04152323	0.30800034	0.289381792	0.445733713
cg12245706	DYSF;DYS	2	0.00077468	0.26413114	0.338194604	0.591180372
cg14196395	DYSF;DYS	2	0.00132175	0.27829333	0.371516551	0.574447821
cg22480875	E2F7	12	0.00358002	0.57713453	0.617985163	0.734846806
cg09172244	EBF1	5	0.00247734	0.30194028	0.325593847	0.545794077
cg11690724	EBF1	5	0.00065949	0.41900127	0.56525764	0.719251137
cg27417526	EBF3	10	0.00735709	0.59826264	0.551212681	0.715732024
cg03774288	EBF3	10	0.00574743	0.32391745	0.30916034	0.486154949
cg20161275	EBF3	10	0.01590217	0.30809237	0.254548894	0.431609379
cg16093296	EBF3	10	0.00356189	0.61269355	0.702655508	0.830390582
cg23250262	EBF3	10	0.02833301	0.26499661	0.257599531	0.403799897
cg06344553	EBF3	10	0.02414194	0.44910179	0.47283187	0.620171292
cg11834658	ECE1;ECE	1	0.00141131	0.48323097	0.50294609	0.639546025
cg13562122	ECE1;ECE	1	0.00405794	0.41009503	0.353152728	0.535784293
cg13138089	ECEL1P2	2	0.01155848	0.42866742	0.407064003	0.532090655
cg24767131	ECEL1P2	2	0.00624349	0.32738604	0.287918188	0.429627035
cg21221557	EDIL3	5	0.00279455	0.5932348	0.536986888	0.743271602
cg16306043	EDIL3	5	0.00917376	0.22263546	0.22593078	0.391974135
cg05423018	EEPD1	7	0.00084118	0.24180284	0.257235804	0.386627289
cg18454133	EFS;EFS	14	0.02247384	0.27118409	0.224389969	0.398907969
cg16681436	EGFL8	6	0.00109472	0.19193016	0.197733589	0.341627701
cg00359087	EGFL8	6	0.00258141	0.45412953	0.422962806	0.568168194

cg06154903	EHD1	11	0.00302287	0.22921905	0.26957604	0.382872677
cg23121787	EHD4	15	0.01336583	0.39432277	0.373435668	0.520855105
cg21195984	EHMT1	9	0.00296383	0.33926618	0.263954222	0.452306414
cg14064959	EHMT1	9	0.00229687	0.52081355	0.489443929	0.711106755
cg19268388	EHMT2;E	6	0.03585699	0.73775941	0.738016023	0.841672233
cg24493649	EHMT2;SI	6	0.01162689	0.22611525	0.20226952	0.373058245
cg19770715	EHMT2;SI	6	0.00073118	0.25911054	0.296483087	0.480470166
cg19539385	EIF2AK1;E	7	0.01749142	0.22362705	0.238005897	0.360512351
cg25922279	EIF2C2;EI	8	0.0008696	0.48628425	0.480528596	0.667039647
cg24251448	EIF2C2;EI	8	0.00113577	0.56101064	0.552431979	0.740481071
cg03172341	EIF2C2;EI	8	0.00126466	0.60227813	0.640469815	0.750716352
cg13582226	EIF2C2;EI	8	0.00079224	0.4698037	0.498543112	0.644904904
cg02911270	EIF2C2;EI	8	0.0107931	0.52603802	0.507145209	0.651743305
cg15936375	EIF2C2;EI	8	0.00120369	0.5028474	0.478649157	0.619613906
cg21301240	EIF3H	8	0.00997527	0.56289236	0.525024734	0.708741773
cg17887364	EIF4EBP1	8	0.00521564	0.2437122	0.226327841	0.430178158
cg17868307	EIF4H;EIF	7	0.00232775	0.47381864	0.560017587	0.677924599
cg18429196	EIF4H;EIF	7	0.0034485	0.37924594	0.389944032	0.546929955
cg12691679	ELF1	13	0.00240097	0.32366048	0.418810137	0.541290803
cg03940407	ELF2	4	0.01698967	0.43635191	0.472467695	0.638802601
cg05653018	ELF3;ELF3	1	0.00288962	0.13150955	0.110678085	0.239149167
cg16986578	ELF3;ELF3	1	0.00295889	0.15550822	0.171250294	0.301562678
cg07897871	ELF3;ELF3	1	0.00079224	0.45630565	0.498352401	0.706092931
cg02076020	ELF3;ELF3	1	0.00120195	0.27318857	0.314887429	0.500566345
cg26328757	ELF3;ELF3	1	0.0016378	0.44491595	0.486289428	0.628513129
cg12967164	ELFN1	7	0.0047737	0.29255997	0.306501335	0.48376761
cg26287309	ELFN2	22	0.00316916	0.37767945	0.378617858	0.485003001
cg08721338	ELMO3	16	0.01405386	0.23495305	0.235856199	0.367727894
cg09777237	ELN;ELN;I	7	0.00513872	0.47789724	0.479781453	0.629303107
cg15983182	ELN;ELN;I	7	0.00343258	0.14611847	0.119723413	0.271947881
cg25729687	ELN;ELN;I	7	0.00725225	0.13727785	0.128838986	0.287212386
cg17363943	ELOVL6;E	4	0.01025339	0.23486205	0.206752695	0.372976772
cg13352306	EML3;RO	11	0.00160583	0.27208989	0.264349465	0.428544044
cg14055970	ENG;ENG	9	0.01026108	0.46854496	0.499223844	0.6488223
cg26938599	ENKUR	10	0.02025944	0.31019719	0.38136868	0.482270761
cg13777984	ENOX1;EI	13	0.00359691	0.15505146	0.174568159	0.372147601
cg00049892	ENPP1	6	0.01052586	0.40923572	0.398613096	0.604372688
cg18796704	ENPP1	6	0.0084821	0.16075901	0.137232605	0.282014098
cg19270309	ENPP7	17	0.00112915	0.24156687	0.294179413	0.413782496
cg02148800	EP400	12	0.00485228	0.4458515	0.479304736	0.585332862
cg05629774	EP400	12	0.00851187	0.46424085	0.463638928	0.595001257
cg09075307	EP400	12	0.00317466	0.64650417	0.646141739	0.75034233
cg27007356	EP400	12	0.0013706	0.56569535	0.585367912	0.700706032
cg12608132	EPAS1	2	0.00318268	0.28005568	0.248465608	0.396549227
cg13997788	EPAS1	2	0.00453882	0.32669539	0.298905516	0.452121182
cg10507988	EPAS1	2	0.02530958	0.43991323	0.442774212	0.550650445

cg22423984	EPB41L1	20	0.01751915	0.18668305	0.138863266	0.287663674
cg14090400	EPB41L3	18	0.00262388	0.57892633	0.637593678	0.797017511
cg23205365	EPB41L5	2	0.00556434	0.2030137	0.150382111	0.33139034
cg16678564	EPB49;EP	8	0.00095459	0.39996769	0.398759676	0.572415277
cg16448847	EPHB1	3	0.00344958	0.18472792	0.212305863	0.333442679
cg02974898	EPHB2;EF	1	0.00546217	0.14361959	0.111620633	0.277242392
cg18156963	EPHB2;EF	1	0.03515606	0.24041064	0.211959233	0.375945048
cg13694876	EPHB3	3	0.00359753	0.41907234	0.467238202	0.618302541
cg20438472	EPHB3	3	0.00405622	0.60708836	0.608426386	0.716840423
cg15334250	EPHB3	3	0.01006689	0.33918855	0.388006359	0.506152664
cg22493616	EPHB4	7	0.00411612	0.48539637	0.464505595	0.588322242
cg22303758	EPHX2	8	0.0033048	0.26642508	0.353705369	0.486401339
cg17742418	EPN2;EPN	17	0.00152023	0.6687704	0.657247727	0.792827851
cg14173476	EPN2;EPN	17	0.00186737	0.25071513	0.24313733	0.423663164
cg25673598	EPN2;EPN	17	0.00075989	0.12023639	0.157424745	0.290038162
cg11414610	EPS15L1	19	0.00176085	0.32268283	0.42375856	0.561898057
cg03332546	EPS15L1	19	0.00123203	0.09525794	0.113553374	0.252861546
cg16597993	EPS15L1	19	0.00190848	0.22046217	0.215630568	0.3659165
cg17649115	EPS8L2	11	0.00183809	0.76161169	0.78044218	0.882201846
cg04587886	EPS8L2	11	0.00116732	0.59968524	0.596351136	0.715171315
cg06378010	ERC1;ERC	12	0.01502763	0.22833921	0.230374662	0.362222
cg21578987	ERG;ERG	21	0.01896129	0.23230868	0.250371055	0.413530711
cg14536431	ERG;ERG	21	0.00866121	0.1357675	0.110203976	0.260479854
cg15833565	ERI3	1	0.00144775	0.19667403	0.203714745	0.35821208
cg02650323	ERLIN2;EI	8	0.01860545	0.20985545	0.2041499	0.330243904
cg06193232	ERN1	17	0.00327469	0.21738805	0.217397059	0.393740973
cg15676711	ERRFI1	1	0.00524148	0.41587042	0.458065636	0.57324352
cg06159484	ERRFI1	1	0.00822746	0.39756473	0.367000199	0.549662532
cg25338972	ESR1	6	0.01417701	0.14833967	0.105863917	0.251159138
cg07455133	ESR1;ESR	6	0.01546656	0.21071595	0.195259946	0.340647757
cg00944421	ESRP2	16	0.01487618	0.23309211	0.217961553	0.360079981
cg05212892	ESYT2	7	0.00334886	0.35202088	0.309717422	0.548298379
cg16354756	ETV3;CYC	1	0.008246	0.50528885	0.518878677	0.628003701
cg03609488	ETV6	12	0.00258141	0.1145828	0.114562099	0.215693847
cg26889659	EXOC2	6	0.00810421	0.20371682	0.169182616	0.326752644
cg08460580	EXOC2	6	0.00878866	0.63002266	0.609228951	0.730821328
cg00449767	EXOC3L2	19	0.00542561	0.29501282	0.288431755	0.403845403
cg01565314	EXOC3L2	19	0.00113574	0.1815062	0.178838059	0.286335597
cg09450024	EXOC3L2	19	0.01114245	0.18715235	0.163082638	0.306202739
cg08882547	EXOC3L2	19	0.00566397	0.21374254	0.178773246	0.325001752
cg13654884	EXOC6	10	0.00233298	0.54582131	0.598684953	0.706137943
cg13314863	EXOC6;EX	10	0.00514	0.5787022	0.601435455	0.718645402
cg24892992	EXT1	8	0.00140256	0.40481521	0.472401889	0.645376715
cg22271457	EXT1	8	0.00075963	0.59799563	0.637467846	0.812889845
cg03599855	EXT1	8	0.0169634	0.22490095	0.259558538	0.383208306
cg04395788	EXT1	8	0.00523471	0.30109784	0.388911322	0.507394602

cg00332126	EXT2;EXT	11	0.02944061	0.26575105	0.232199907	0.372418772
cg07551677	EXTL3	8	0.00168532	0.62261161	0.675354383	0.811693896
cg00550493	EYS	6	0.01164166	0.21173293	0.182088074	0.342603733
cg18868540	F2	11	0.00294138	0.32857307	0.348015358	0.536570571
cg26667735	FAAH	1	0.00065949	0.54504911	0.647730929	0.777249942
cg07168328	FAAH	1	0.00196477	0.28213078	0.299901475	0.419018343
cg05009047	FAM100A	16	0.01154548	0.62580573	0.662601893	0.765441334
cg05712979	FAM101A	12	0.00097618	0.46402588	0.508158003	0.621751782
cg18672389	FAM101A	12	0.00148491	0.28943683	0.314280591	0.570134002
cg14273093	FAM101A	12	0.00248044	0.24911783	0.293006797	0.47769815
cg13761284	FAM102A	9	0.00158181	0.36073755	0.369337808	0.506505723
cg13692655	FAM102A	9	0.00403008	0.34014629	0.342319675	0.467048603
cg12354318	FAM107B	10	0.00521564	0.25357701	0.252452033	0.375126424
cg08608727	FAM109A	12	0.00065949	0.51280017	0.567846275	0.674322591
cg01285862	FAM114A	5	0.02289835	0.16168282	0.153981671	0.277622795
cg21184699	FAM120A	9	0.00095989	0.65539741	0.670168612	0.813889018
cg06381463	FAM125B	9	0.00405622	0.45786831	0.410393732	0.640561952
cg11970163	FAM129A	1	0.02822075	0.47205387	0.426901748	0.580640391
cg25182523	FAM129A	1	0.00075421	0.28195328	0.352231633	0.483169312
cg06712722	FAM129B	9	0.00488333	0.43384006	0.467999032	0.593954111
cg14408339	FAM129B	9	0.00184545	0.24394679	0.248408691	0.350798676
cg21169267	FAM129B	9	0.00672923	0.41557513	0.366497975	0.524685171
cg17156402	FAM131C	1	0.00077454	0.50035037	0.531334546	0.675000668
cg13782092	FAM13A;	4	0.0075862	0.55977831	0.50069908	0.688977354
cg04860977	FAM155A	13	0.00287555	0.31807879	0.355170165	0.584299405
cg03520938	FAM160B	8	0.00272772	0.23898138	0.241648811	0.351239924
cg06551905	FAM167A	8	0.00802316	0.14121831	0.13285282	0.243124077
cg16495818	FAM168A	11	0.00751061	0.19593099	0.227806936	0.332932999
cg11452043	FAM168B	2	0.00216893	0.46893816	0.402550204	0.593194032
cg01245667	FAM169B	15	0.02346046	0.32561449	0.267486038	0.443710818
cg12771178	FAM170B	10	0.00211366	0.18305291	0.166105537	0.286564537
cg16512990	FAM170B	10	0.01068625	0.40919673	0.41823701	0.539030098
cg27406618	FAM170B	10	0.00327239	0.24318453	0.244772941	0.397089275
cg01948367	FAM172A	5	0.0266726	0.62479095	0.626504846	0.729539707
cg22810403	FAM176B	1	0.00653182	0.68192119	0.683759514	0.802244744
cg18665384	FAM177B	1	0.00967401	0.31537408	0.292332578	0.45595163
cg26108017	FAM178B	2	0.00245827	0.4504408	0.445537399	0.558609051
cg05368724	FAM178B	2	0.00092758	0.33924749	0.350512842	0.514810403
cg05557262	FAM180A	7	0.00917562	0.63896027	0.649110817	0.758255499
cg00005619	FAM180B	11	0.00111524	0.23630761	0.254118952	0.410204979
cg24657419	FAM184A	6	0.0048909	0.60019885	0.567565373	0.701189994
cg03309770	FAM18A;	16	0.00405622	0.15322671	0.150191751	0.256384699
cg26952618	FAM18A;	16	0.00702677	0.1513523	0.152195299	0.263057741
cg12801917	FAM18B2	17	0.00233298	0.25489605	0.247891794	0.38436045
cg25790133	FAM193A	4	0.00432931	0.32022207	0.276448679	0.542642719
cg05083414	FAM193A	4	0.00524121	0.26959737	0.228330729	0.372729042

cg05949640	FAM193A	4	0.00735604	0.33473534	0.302246812	0.487817963
cg26981170	FAM193A	4	0.02248239	0.29072947	0.244982212	0.398081337
cg20163033	FAM193A	4	0.00168532	0.65278754	0.617873536	0.762741663
cg05883695	FAM198A	3	0.00271218	0.19516549	0.215397096	0.319674752
cg16716189	FAM19A3	1	0.00610999	0.32763507	0.307120544	0.43079441
cg19579080	FAM20A	17	0.00500442	0.18859177	0.223948427	0.371638996
cg05031931	FAM20C	7	0.00758361	0.16841662	0.159130942	0.305508967
cg00308560	FAM20C	7	0.00169362	0.24020398	0.205094697	0.348698678
cg23782833	FAM20C	7	0.00213077	0.24660386	0.276615393	0.430486335
cg18234533	FAM20C	7	0.0018139	0.34628942	0.382472854	0.524852811
cg19619414	FAM20C	7	0.0012026	0.15593465	0.157199661	0.261955553
cg12748511	FAM20C	7	0.00568798	0.17127432	0.140622306	0.283483391
cg25929664	FAM20C	7	0.00160273	0.18501268	0.198479159	0.39789555
cg27640234	FAM20C	7	0.00286125	0.48903785	0.473818762	0.644925947
cg11200568	FAM20C	7	0.00735709	0.31217708	0.2716649	0.41547243
cg19476741	FAM20C	7	0.00232775	0.18778747	0.189127659	0.330772736
cg08616667	FAM20C	7	0.00209789	0.16530785	0.15288364	0.280804038
cg04256674	FAM20C	7	0.01891102	0.67494183	0.666363176	0.807716361
cg24286414	FAM20C	7	0.00130462	0.48333364	0.519455506	0.681937378
cg08728302	FAM20C	7	0.00185795	0.30310503	0.301340629	0.566906985
cg24248680	FAM24B	10	0.01638971	0.52824749	0.550909237	0.660011552
cg15683166	FAM24B	10	0.01580766	0.35883421	0.29407452	0.48369997
cg06751612	FAM38A	16	0.00330705	0.46444573	0.436144783	0.57716158
cg06855137	FAM38A	16	0.00090315	0.2214813	0.24950843	0.351546172
cg27539806	FAM38A	16	0.00142756	0.40504833	0.440829572	0.633330389
cg16641055	FAM38A	16	0.0035878	0.29401831	0.295879064	0.399487942
cg00626702	FAM53A	4	0.01842786	0.20766646	0.18235227	0.321606337
cg26725552	FAM53B	10	0.02382799	0.67939653	0.68255888	0.82876271
cg19975346	FAM53B	10	0.00065949	0.16614349	0.277683078	0.480968835
cg23088510	FAM53B	10	0.01416646	0.28254428	0.338069262	0.49961553
cg00557402	FAM55D;	11	0.00620216	0.42562158	0.505981235	0.629566712
cg22223402	FAM55D;	11	0.02985248	0.4248458	0.395971815	0.53053556
cg01045946	FAM63A;	1	0.0062271	0.43310375	0.430062596	0.553151541
cg01872947	FAM65A	16	0.00349009	0.20567111	0.208359093	0.351457423
cg19619331	FAM71F2	7	0.00841172	0.18331464	0.211408171	0.338397708
cg22248750	FAM82A1	2	0.01130304	0.23292651	0.156828999	0.355622567
cg00846121	FAM83E	19	0.00567904	0.23124402	0.208228322	0.358064043
cg13791971	FANCC	9	0.00283173	0.28157551	0.325848271	0.453112891
cg17380943	FARP1	13	0.00582397	0.43463193	0.454562697	0.608024581
cg15168958	FARP1	13	0.00248015	0.35713039	0.350734089	0.550796844
cg20750642	FARP1	13	0.00785654	0.20615199	0.159242241	0.33887884
cg11829616	FARP1	13	0.01698967	0.44886467	0.47449877	0.588531766
cg16450249	FARP1;FA	13	0.00624349	0.17817478	0.145770528	0.281344382
cg19832331	FARP1;FA	13	0.02075286	0.62131224	0.671936806	0.785714156
cg06450397	FAS;FAS;F	10	0.00079883	0.38982478	0.46543024	0.675172523
cg20656964	FAT1	4	0.00314038	0.50549028	0.556350823	0.670082483

cg01880399	FAT1	4	0.00520163	0.30767991	0.350981756	0.508414412
cg27661793	FAT1	4	0.01098413	0.22076871	0.174105649	0.343577434
cg18183817	FBLN2;FB	3	0.00077361	0.64585865	0.726174971	0.838170444
cg00498816	FBLN2;FB	3	0.00176085	0.66564029	0.722674738	0.823178074
cg04956944	FBLN7;FB	2	0.00109472	0.39679521	0.405853367	0.621726591
cg14549078	FBN1	15	0.02336655	0.18456977	0.146958246	0.291897661
cg20664313	FBRSL1	12	0.00068904	0.36227636	0.405162135	0.548240528
cg19510206	FBRSL1	12	0.00702537	0.51521585	0.543723776	0.680190913
cg00960772	FBRSL1	12	0.00569156	0.30474963	0.334521147	0.462378864
cg24462656	FBRSL1	12	0.0122594	0.22314107	0.1515971	0.339808983
cg24621258	FBRSL1	12	0.01817641	0.26090651	0.183049102	0.398646748
cg25750363	FBRSL1	12	0.00720967	0.24924558	0.209817427	0.477369425
cg03378525	FBXL18	7	0.00243597	0.46959031	0.484062497	0.6046818
cg01149929	FBXL7	5	0.02489039	0.54387446	0.549849745	0.656761617
cg02160692	FBXO11;F	2	0.01164121	0.72010235	0.724444778	0.826079354
cg24878594	FBXO2	1	0.0127705	0.15831874	0.159870027	0.263304775
cg19256813	FBXO21;F	12	0.0166015	0.19201998	0.137141153	0.292635187
cg26829322	FBXO44;F	1	0.0107931	0.35406263	0.324483221	0.504916339
cg05796704	FBXO44;F	1	0.00316372	0.148357	0.142120621	0.28955898
cg05540569	FBXO47	17	0.03488234	0.14626014	0.170933493	0.272609558
cg10523001	FBXW11;I	5	0.01011462	0.33385318	0.30759575	0.521733917
cg23985408	FBXW11;I	5	0.00295889	0.27216457	0.329822679	0.493010525
cg27371456	FBXW4	10	0.01023819	0.17333591	0.145433623	0.324167454
cg08361180	FBXW4	10	0.00417561	0.14333967	0.129296576	0.311508086
cg17860133	FBXW8;FI	12	0.00281579	0.5753524	0.598699235	0.737722481
cg07050611	FBXW8;FI	12	0.00207724	0.37528505	0.446450574	0.609450067
cg22942846	FCGR2B;F	1	0.00336153	0.3102445	0.277223916	0.460543683
cg26549860	FCHSD2	11	0.0047315	0.29924332	0.373520322	0.516416553
cg24496021	FCHSD2	11	0.02980647	0.50021384	0.493603353	0.636560435
cg17467108	FCHSD2	11	0.00426883	0.54033309	0.522168684	0.666961467
cg20577477	FDFT1	8	0.00648185	0.49531319	0.509392103	0.671325447
cg06979924	FDPS;FDP	1	0.00852388	0.22235997	0.217459574	0.338885091
cg26827653	FEZ1;FEZ1	11	0.01554011	0.2539522	0.242850003	0.36581039
cg07541160	FGD2	6	0.00211914	0.16011	0.159659258	0.263972032
cg09718347	FGD5	3	0.00147518	0.38001378	0.486561443	0.626615521
cg05173159	FGD6	12	0.00387229	0.51229544	0.531616967	0.641055251
cg20684197	FGF1;FGF	5	0.00228672	0.21026805	0.278177466	0.481931265
cg21619138	FGF1;FGF	5	0.01506509	0.18250347	0.11847097	0.286526294
cg02406096	FGF1;FGF	5	0.01672645	0.24656328	0.210524157	0.349616352
cg19576738	FGF2	4	0.00187339	0.10964145	0.148349059	0.30160565
cg14703482	FGF2	4	0.00539451	0.13844226	0.129403522	0.282080564
cg26955980	FGF9	13	0.0103161	0.5657343	0.568986755	0.696172156
cg17091967	FGFBP1	4	0.00426288	0.32630568	0.345276719	0.493127945
cg27653103	FGFR1;FG	8	0.00211366	0.12743128	0.13324096	0.315515142
cg03552039	FGFR2;FG	10	0.00280561	0.31102986	0.308420755	0.479575948
cg13734061	FGFR2;FG	10	0.00426288	0.52926998	0.560277079	0.707521043

cg25294906	FGFR2;FG	10	0.0019906	0.36037644	0.383639391	0.583976636
cg10280007	FGFR2;FG	10	0.00897515	0.22825629	0.264021413	0.427183739
cg12226006	FGFR2;FG	10	0.00582851	0.30715631	0.28165689	0.537967993
cg14834653	FGFR2;FG	10	0.00353204	0.19701865	0.244367388	0.40455322
cg20277356	FGFR2;FG	10	0.00873782	0.24942825	0.292639244	0.463133155
cg13175830	FGFR2;FG	10	0.00349176	0.12259456	0.129054092	0.272102537
cg17337672	FGFR2;FG	10	0.00485228	0.33601035	0.359488494	0.476548591
cg14856220	FGFR2;FG	10	0.04333813	0.25527151	0.295561348	0.413173777
cg10362842	FGFR2;FG	10	0.0011159	0.29877151	0.360084233	0.482854011
cg10314760	FGFR2;FG	10	0.01841511	0.29137351	0.326791383	0.515027376
cg17997363	FGFR2;FG	10	0.00521564	0.2068611	0.21383423	0.36636255
cg10788901	FGFR2;FG	10	0.00153118	0.19714808	0.226472556	0.326833591
cg00730832	FGFR2;FG	10	0.00806395	0.18836316	0.183020849	0.368721597
cg08145949	FGFR3;FG	4	0.00079224	0.27339587	0.307746731	0.519137059
cg03629948	FGFR3;FG	4	0.02987993	0.51528344	0.527692481	0.630248714
cg02137691	FGFR3;FG	4	0.00576163	0.40388646	0.39322634	0.509529379
cg15697036	FGFR3;FG	4	0.001181	0.38708799	0.394862515	0.572102254
cg21311834	FGFR3;FG	4	0.00162163	0.207539	0.270391062	0.524423389
cg18229134	FGFR4;FG	5	0.00336153	0.4542154	0.459465593	0.619991912
cg04849878	FGFR4;FG	5	0.01686581	0.38559152	0.398049443	0.518048016
cg00160522	FGFRL1;F	4	0.00096621	0.46221315	0.501527495	0.614522597
cg06839424	FGFRL1;F	4	0.00073118	0.37123585	0.416035663	0.526904042
cg04145890	FGFRL1;F	4	0.00567904	0.19477523	0.183277373	0.294971646
cg18699025	FGFRL1;F	4	0.01358672	0.23200959	0.198839947	0.344916052
cg07727358	FGFRL1;F	4	0.00127764	0.39827888	0.428811239	0.548549231
cg02826685	FGFRL1;F	4	0.00126804	0.58569898	0.585143276	0.764428359
cg08521859	FGFRL1;F	4	0.00458661	0.19757872	0.184090625	0.328854732
cg14724492	FGFRL1;F	4	0.00167382	0.43838852	0.45699041	0.59858251
cg02014217	FGFRL1;F	4	0.00207888	0.497238	0.495164831	0.611317204
cg11411884	FGFRL1;F	4	0.00233734	0.54130349	0.577861474	0.715180913
cg14869372	FGFRL1;F	4	0.00089116	0.39497644	0.421046108	0.639883865
cg13184177	FGR;FGR;	1	0.02376522	0.528425	0.520875301	0.632445631
cg23167456	FGR;FGR;	1	0.00283769	0.62947252	0.653117712	0.766865183
cg14160480	FHAD1	1	0.01471045	0.14156162	0.115186776	0.259545481
cg11815980	FHIT;FHIT	3	0.00224923	0.41531545	0.494290643	0.629795439
cg08239282	FHL2	2	0.01645625	0.18073442	0.179542552	0.290443933
cg19226017	FKBP5;LO	6	0.00376781	0.27992924	0.30998469	0.492609832
cg07702311	FLI1;FLI1	11	0.00705117	0.4427231	0.469030185	0.6244091
cg27390009	FLII	17	0.00387834	0.31933969	0.312026132	0.43563576
cg01048349	FLJ10357	14	0.01342633	0.32216539	0.32629105	0.459788
cg22800743	FLJ22536	6	0.00934957	0.18167175	0.189419544	0.345785686
cg26926497	FLJ22536	6	0.00396452	0.15160002	0.151100177	0.320015577
cg08373323	FLJ32810	11	0.00168532	0.2103749	0.24966732	0.47646614
cg14192935	FLJ35024	9	0.00302287	0.142449	0.146859278	0.265347296
cg14761417	FLJ43663;	7	0.00383939	0.40265527	0.366402339	0.574378238
cg03243768	FLJ46111	11	0.00246645	0.72749169	0.768153071	0.899138127

cg05116002	FLNB;FLN	3	0.00171175	0.18721765	0.231930444	0.374795731
cg01869548	FLYWCH2	16	0.00629918	0.47509221	0.455998629	0.601739254
cg17802486	FMN1	15	0.00561885	0.16371089	0.118040634	0.329396102
cg13619277	FMN1	15	0.01278183	0.15927433	0.205465044	0.339773655
cg19735250	FMNL1	17	0.00555917	0.25837956	0.259566593	0.442614318
cg21617353	FMNL1	17	0.0154705	0.35155984	0.304842577	0.496985682
cg20408776	FMNL1	17	0.01502763	0.32147739	0.256765904	0.444013156
cg26207245	FMNL2	2	0.02115736	0.47602376	0.476600369	0.66086607
cg15705810	FMNL2	2	0.00509504	0.17758903	0.183330052	0.384660776
cg06343233	FMNL2	2	0.00247433	0.3525843	0.313427024	0.554454264
cg11173609	FMNL2	2	0.04220346	0.39505642	0.380576952	0.544408393
cg21860045	FMNL2	2	0.00359124	0.56160996	0.567317392	0.748192058
cg16034517	FMO1	1	0.02025944	0.17218815	0.124638065	0.294274235
cg12495731	FN3K	17	0.00106082	0.42474149	0.45865325	0.706246962
cg08495617	FNDC3B;F	3	0.00958999	0.39868364	0.435534073	0.584543614
cg12202228	FNDC3B;F	3	0.02469417	0.39180105	0.346709575	0.531387132
cg00000109	FNDC3B;F	3	0.00816438	0.26673485	0.299894091	0.481738716
cg26334671	FNDC3B;F	3	0.00855749	0.30623459	0.336775521	0.511804276
cg00360324	FNDC4	2	0.0011122	0.62842031	0.690261241	0.805231688
cg26161895	FNIP2	4	0.01895346	0.3558055	0.420944466	0.562096574
cg11244091	FOXA3	19	0.00710861	0.37187579	0.393407065	0.513056832
cg23318967	FOXK1	7	0.00763406	0.39886978	0.386488854	0.524106585
cg22581896	FOXK1	7	0.00684483	0.45218602	0.484948319	0.622076934
cg11931953	FOXK1	7	0.00393302	0.64683787	0.629490034	0.8069238
cg05066096	FOXK1	7	0.03533922	0.49362566	0.480633461	0.605401054
cg06114605	FOXK1	7	0.00376892	0.40301424	0.459204867	0.619903088
cg25330264	FOXK1	7	0.00186758	0.33328559	0.431133804	0.545071
cg05237718	FOXK1	7	0.00177922	0.42864882	0.424674119	0.573575613
cg06114987	FOXL1	16	0.00186737	0.59617659	0.591707706	0.758610266
cg14113778	FOXN3	14	0.00089116	0.71222589	0.728366604	0.853351239
cg18359596	FOXN3;FC	14	0.00677233	0.57835263	0.602948254	0.743500967
cg01838853	FOXN4	12	0.00349176	0.23144957	0.225855719	0.378572402
cg07580479	FOXO3;FC	6	0.01426008	0.53488486	0.604829806	0.709801413
cg19484481	FOXO3;FC	6	0.00130049	0.30233662	0.371856092	0.62991317
cg15963983	FOXP1	3	0.04902903	0.51323915	0.505421927	0.633203612
cg00201568	FOXP1	3	0.02115579	0.51427527	0.528050657	0.658047918
cg03504834	FOXP2;FC	7	0.0261269	0.25906692	0.293626803	0.402385408
cg18871253	FOXP2;FC	7	0.02394548	0.23088073	0.206347596	0.348346773
cg11177740	FOXP4;FC	6	0.00213077	0.21463753	0.217256467	0.344591181
cg10719970	FOXP4;FC	6	0.00845805	0.40684614	0.402577392	0.508363748
cg03127034	FOXP4;FC	6	0.00092643	0.56155366	0.562372778	0.692632335
cg07588430	FOXP4;FC	6	0.00112793	0.51374314	0.538314757	0.693627028
cg23867085	FOXP4;FC	6	0.00232154	0.2248223	0.221059242	0.371840182
cg17631429	FOXS1	20	0.00968951	0.62827886	0.570536276	0.785671592
cg15237820	FRAS1;FR	4	0.00554206	0.47446441	0.42442033	0.642780529
cg13426351	FREQ	9	0.0061767	0.42921633	0.429500943	0.577469212

cg06127202	FRK	6	0.00194185	0.20605175	0.229480167	0.379878218
cg24008634	FRMD4A	10	0.02385226	0.47299573	0.494081558	0.601579177
cg11813497	FRMD4A	10	0.01645625	0.30331296	0.249348335	0.421683638
cg05104995	FRMPD2;	10	0.00405622	0.50110833	0.500303888	0.626377183
cg25274185	FRY	13	0.00620216	0.22807015	0.303997494	0.409062785
cg16995086	FRY	13	0.03241445	0.19780414	0.221234369	0.335775431
cg09188385	FSCN1	7	0.00233298	0.19308862	0.188800412	0.383808214
cg13087761	FSCN1	7	0.00208745	0.1877258	0.172459575	0.422168029
cg06969129	FSCN1	7	0.00893433	0.305781	0.288804988	0.424087052
cg01512532	FSCN2;FS	17	0.02634735	0.28635078	0.235650401	0.400135042
cg26166170	FSIP1	15	0.00368022	0.53148895	0.566025738	0.67684673
cg12075901	FURIN	15	0.0011206	0.67703085	0.697468249	0.846808658
cg00758797	FURIN	15	0.01205623	0.38455046	0.443401298	0.549744779
cg14231966	FURIN	15	0.00219476	0.33993468	0.345480811	0.461451336
cg24256772	FUT4	11	0.00357923	0.53024541	0.535838863	0.670161809
cg08863777	FUT4	11	0.00086791	0.19544058	0.215406045	0.334137215
cg18554976	FXYD1;FX	19	0.00270728	0.35778305	0.310333774	0.477663411
cg22335223	FXYD2	11	0.00066232	0.29604415	0.354028707	0.468482738
cg00480115	FXYD3;FX	19	0.00554761	0.23063072	0.220122729	0.433443251
cg21304163	FXYD3;FX	19	0.0026334	0.64361765	0.655879862	0.757187693
cg14848289	FXYD6;FX	11	0.01189791	0.2948122	0.252446547	0.422170606
cg00161459	FZD6;FZD	8	0.00648443	0.51514576	0.556299003	0.70912619
cg02906915	FZD8;FZD	10	0.00704751	0.15733755	0.127608848	0.287514306
cg03009240	FZR1	19	0.00607259	0.22876829	0.180249976	0.36273897
cg14142817	GAB1;GA	4	0.00103001	0.63570834	0.667969195	0.804179349
cg25742761	GAB1;GA	4	0.00852172	0.50344202	0.541888247	0.683714452
cg03286735	GAK	4	0.01638971	0.36768709	0.406530755	0.540384228
cg10961323	GALE;GAI	1	0.01966537	0.24078719	0.223893776	0.348169936
cg21523719	GALE;GAI	1	0.00961487	0.32920955	0.327358678	0.476677429
cg08466889	GALNS	16	0.00444845	0.51394951	0.454657338	0.622721485
cg09308897	GALNS	16	0.00197986	0.36817469	0.360916429	0.522294054
cg08894593	GALNS	16	0.02568798	0.15763427	0.141246047	0.283567115
cg06062279	GALNS	16	0.0022998	0.52773442	0.534277217	0.635438282
cg14968553	GALNT10	5	0.00247655	0.49984154	0.493617449	0.664429098
cg00563858	GALNT10	5	0.00209262	0.32783142	0.324758954	0.558349408
cg00566187	GALNT2	1	0.02524768	0.42849796	0.481479177	0.623568166
cg26525772	GALNT2	1	0.0174639	0.31889254	0.349794577	0.526737436
cg16698674	GALNT2	1	0.0058315	0.29888934	0.327221306	0.496186803
cg15817769	GALNT2	1	0.00284268	0.20937662	0.253909312	0.443981842
cg16246661	GALNT2	1	0.03209984	0.38264293	0.409759963	0.574710804
cg12526197	GALNT2	1	0.00184569	0.2155049	0.235589596	0.349696867
cg17954142	GALNT2	1	0.0011143	0.32567498	0.37575141	0.509787118
cg15438014	GALNTL2	3	0.02003709	0.61458479	0.609853511	0.731544864
cg03299336	GALNTL4	11	0.00464252	0.53785084	0.5397074	0.651375472
cg15094908	GALNTL4	11	0.00275412	0.16181515	0.156890749	0.338945908
cg23678274	GALNTL4	11	0.0047737	0.10830331	0.108167931	0.240873742

cg05527856	GALNTL4	11	0.00151139	0.20192495	0.214092489	0.408186985
cg21089050	GAS7	17	0.00100561	0.6419203	0.671338096	0.814398028
cg10296867	GAS7	17	0.00067859	0.60118773	0.695152691	0.814052308
cg10237765	GAS7;GAS	17	0.00539186	0.37868542	0.412169928	0.527703039
cg10089865	GATA3;G/	10	0.00512138	0.59425255	0.538675365	0.747913203
cg03935183	GATA3;G/	10	0.01884096	0.52220283	0.571014111	0.706588771
cg25209111	GBF1	10	0.04622903	0.29851985	0.287504915	0.408975579
cg12105183	GBP6	1	0.00283173	0.44375499	0.419723265	0.594751543
cg02868790	GCLC	6	0.0019906	0.30559208	0.343488429	0.564536964
cg20546778	GDAP1;G	8	0.02853243	0.28231911	0.248870828	0.393296118
cg19239052	GDF10	10	0.00102816	0.23254842	0.237037444	0.343426397
cg12433084	GDF10	10	0.00314038	0.18833924	0.18716451	0.324866542
cg13565300	GDF10	10	0.00485228	0.18593301	0.181751239	0.302079824
cg17417496	GDF5	20	0.00081925	0.65810869	0.661323093	0.78548998
cg08062713	GDF5	20	0.0046677	0.632859	0.615852453	0.827195156
cg05567631	GDPD5	11	0.01124644	0.43003646	0.404010115	0.543615134
cg22610645	GEFT;GEF	12	0.00775531	0.27359265	0.259781973	0.377477454
cg18383668	GEMIN7;(	19	0.0021194	0.25084889	0.21761679	0.370236798
cg01769243	GFAP;GF/	17	0.00324717	0.33224513	0.308647858	0.442510445
cg03383006	GFAP;GF/	17	0.00553815	0.38490138	0.377086701	0.535108773
cg09639715	GFAP;GF/	17	0.00556197	0.21391275	0.199438027	0.322148462
cg00318322	GFOD2;G	16	0.01612589	0.55919255	0.535833891	0.664124448
cg14476487	GFPT1	2	0.04382199	0.62431394	0.652456395	0.759425112
cg06845268	GFPT1	2	0.01004931	0.52201235	0.536520165	0.679327794
cg26338723	GFRA1;GI	10	0.00180551	0.38715607	0.419108886	0.562373757
cg20626616	GHRL;GH	3	0.01760238	0.44889654	0.435042894	0.61027374
cg11030744	GHRL;GH	3	0.00882698	0.50538567	0.453728674	0.677323036
cg08489410	GHRL;GH	3	0.00388142	0.46038601	0.433520701	0.660634818
cg07871024	GHRL;GH	3	0.0037901	0.63150795	0.635936721	0.754410373
cg14528319	GIPC1;GII	19	0.00563693	0.19624934	0.169799851	0.297945647
cg17337670	GIT2;GIT2	12	0.00186737	0.50144193	0.557308678	0.657520938
cg03650119	GJC2	1	0.01471045	0.31613395	0.305986612	0.450935862
cg09257635	GJC2	1	0.00597656	0.20638682	0.189540621	0.325241089
cg14773228	GLI2	2	0.00195549	0.6039942	0.619533437	0.774570293
cg21133992	GLI2	2	0.00219764	0.23296943	0.229170065	0.368240183
cg11791452	GLI2	2	0.00111281	0.16402142	0.18586955	0.330712
cg01791238	GLI2	2	0.00147208	0.20028318	0.236654558	0.424068497
cg02774705	GLI2	2	0.00123883	0.41482711	0.399104888	0.676062537
cg10120778	GLI3	7	0.00971128	0.42943411	0.377543146	0.595131759
cg09008589	GLI3	7	0.01026031	0.16604354	0.14631066	0.319486836
cg15078211	GLI3	7	0.00857641	0.28405668	0.280119272	0.496739381
cg22857963	GLI4	8	0.00269197	0.17646621	0.132964905	0.303459994
cg14825413	GLI4	8	0.00901377	0.24216452	0.192302592	0.363011736
cg27311970	GLIS1	1	0.01678972	0.25986182	0.227106115	0.408045487
cg05040219	GLIS1	1	0.03236546	0.2456179	0.235167519	0.350103725
cg08602528	GLIS2	16	0.00377206	0.35934591	0.352161739	0.468572781

cg04694492	GLIS2	16	0.0039985	0.29869225	0.278706117	0.46488659
cg05052407	GLIS2	16	0.00175728	0.32770037	0.299755895	0.485752254
cg14590902	GLT1D1	12	0.0154863	0.55285667	0.565397313	0.694998252
cg26736878	GLT25D1	19	0.00339981	0.35232583	0.360988947	0.520568417
cg25605307	GLT25D2	1	0.00556621	0.29143868	0.345490872	0.495353493
cg03704673	GLTPD2;G	17	0.00186404	0.29277806	0.323245025	0.491730327
cg07515565	GMDS	6	0.01361135	0.28680886	0.231104148	0.391762189
cg11786839	GMDS	6	0.00148102	0.50016898	0.47660881	0.617260424
cg13394216	GMDS	6	0.00190306	0.23977888	0.27616782	0.434124169
cg10707788	GMDS	6	0.00471011	0.35378115	0.378388541	0.504893015
cg01465824	GMDS	6	0.01206219	0.17676853	0.160719746	0.332069762
cg14358088	GMDS	6	0.03645999	0.17459203	0.19611376	0.332946702
cg06007850	GMDS	6	0.02326197	0.2977772	0.350310663	0.466433291
cg16762849	GMDS	6	0.00117965	0.2513828	0.267369486	0.512268645
cg08840441	GMIP	19	0.00134295	0.32425477	0.334445344	0.504735662
cg07899459	GMIP	19	0.00674227	0.29092036	0.245500708	0.432401608
cg14694205	GMPS	3	0.02241528	0.30745833	0.401169803	0.503004735
cg24366168	GNA11	19	0.00137603	0.31822505	0.320054099	0.471240358
cg05308829	GNA12	7	0.00449258	0.58532019	0.63905364	0.779161696
cg06173857	GNA12	7	0.00100019	0.6700184	0.697228581	0.856170573
cg02046881	GNA12	7	0.00682394	0.64559588	0.653339379	0.777109061
cg21028562	GNAI2;GN	3	0.00997527	0.31631181	0.296755488	0.422776139
cg08644463	GNAI3	1	0.0137817	0.42701423	0.395912093	0.542506515
cg04037585	GNAO1;G	16	0.00684483	0.40411809	0.429264746	0.554505733
cg17658854	GNAS;GN	20	0.00975114	0.49171068	0.470472244	0.597224757
cg21971807	GNAS;GN	20	0.02757672	0.32398516	0.340963365	0.47509542
cg11021321	GNAS;GN	20	0.01678972	0.2611458	0.256528015	0.377182754
cg13680388	GNAS;GN	20	0.0283256	0.35970601	0.364535669	0.473585152
cg24714094	GNAZ;RTI	22	0.00446647	0.28742677	0.298151848	0.454045882
cg00808175	GNB3	12	0.0012838	0.6160416	0.621987839	0.741520402
cg16433737	GNB3	12	0.00131641	0.28202413	0.293555685	0.488467325
cg08407014	GNG7	19	0.028548	0.21778842	0.18683988	0.328616952
cg27176392	GNG7	19	0.0071227	0.65980394	0.660766152	0.77947786
cg04267184	GNRH2;G	20	0.00576931	0.15371438	0.129896519	0.27984912
cg08436467	GNRH2;G	20	0.0338225	0.50310358	0.4942576	0.610949735
cg08424446	GOLGA3	12	0.00208616	0.6583636	0.657527847	0.784283654
cg05614952	GOLGA3	12	0.00263371	0.5997674	0.591562879	0.727806493
cg16120883	GOLGA3	12	0.00685049	0.48470557	0.466852947	0.687300177
cg17346500	GOLIM4	3	0.00248997	0.34633201	0.380212489	0.613788834
cg14574047	GOLM1;G	9	0.03434255	0.54253706	0.530179732	0.64601624
cg00782690	GORASP2	2	0.01137235	0.38525613	0.380914777	0.502301194
cg15011026	GPATCH2	1	0.03513796	0.19181252	0.1532746	0.321786925
cg05087722	GPC1	2	0.00073118	0.54153618	0.556244388	0.704507821
cg20094506	GPC1	2	0.0008696	0.44473806	0.46987728	0.588053817
cg17676994	GPC1	2	0.00088355	0.38085109	0.425850885	0.533888403
cg06047020	GPC6	13	0.0010144	0.23317339	0.27326359	0.448880456

cg11772527	GPC6	13	0.00168532	0.23185062	0.280258707	0.545237302
cg26671050	GPC6	13	0.00590538	0.56402284	0.564518788	0.715415397
cg15318697	GPD1L	3	0.00178025	0.30634281	0.280773284	0.527514007
cg25679431	GPD1L	3	0.00097487	0.4488806	0.423489755	0.692523136
cg07537750	GPHA2	11	0.00150566	0.42592624	0.467891037	0.577382901
cg13393408	GPR107;(	9	0.00233439	0.4312883	0.464502268	0.655186102
cg16204524	GPR113	2	0.02128084	0.38043193	0.444726305	0.549845548
cg12869334	GPR124	8	0.00754388	0.2729186	0.247830483	0.416576227
cg19861632	GPR125	4	0.00716301	0.27489793	0.280820873	0.388336171
cg26126417	GPR126;(	6	0.01102429	0.21808518	0.231117038	0.360774629
cg01008088	GPR133	12	0.01064552	0.42042869	0.348462126	0.595129079
cg12528056	GPR44	11	0.00448016	0.36760789	0.342531861	0.589023579
cg08343644	GPR56;GI	16	0.02351176	0.2765489	0.268924042	0.383632276
cg19592277	GPR75;LC	2	0.01815312	0.2797091	0.211571473	0.38653703
cg10124812	GPR75;LC	2	0.01390004	0.29841617	0.288739104	0.400815911
cg11939825	GPR75;LC	2	0.03856073	0.41397011	0.392441684	0.521355898
cg09167413	GPR98;GI	5	0.00312077	0.52316152	0.521054543	0.680941269
cg18421360	GPR98;GI	5	0.01077982	0.3781523	0.437543228	0.602754706
cg19212391	GPRC5C;(	17	0.00117602	0.60437201	0.623723798	0.77611627
cg14471064	GPSM1	9	0.00149248	0.30299847	0.313503228	0.476473946
cg14213590	GPSM1	9	0.00073265	0.1109024	0.17245017	0.344436377
cg13799504	GPSM1	9	0.00382061	0.20388395	0.221352234	0.353224776
cg22984256	GPSM1	9	0.0011414	0.19997291	0.204772675	0.305951951
cg21195231	GPSM1	9	0.01126909	0.20631274	0.176485283	0.318035795
cg14934821	GPSM1;G	9	0.00168532	0.22813732	0.222474293	0.367064432
cg04305913	GPSM1;G	9	0.00112793	0.20074163	0.208454112	0.317253682
cg01393841	GPSM1;G	9	0.00608343	0.45829365	0.412201937	0.611339932
cg14040131	GPSM1;G	9	0.00099274	0.22908856	0.234106957	0.43720324
cg14271150	GPSM1;G	9	0.00128812	0.38284738	0.390465048	0.549257739
cg13705014	GPSM1;G	9	0.00118326	0.3827226	0.375534285	0.524403605
cg14489965	GPSM1;G	9	0.00076043	0.36320197	0.411814887	0.626804664
cg14203108	GPSM1;G	9	0.00283173	0.3059525	0.334562137	0.436699782
cg00280345	GPT	8	0.00610693	0.18677226	0.17608514	0.301799913
cg07658280	GPT	8	0.00341517	0.32766491	0.353094942	0.543328946
cg05241828	GPT	8	0.00701189	0.25594284	0.236182569	0.446315297
cg09957864	GPT	8	0.0069524	0.25613763	0.260016966	0.467556614
cg25600446	GPT	8	0.01096214	0.25250924	0.250193599	0.376575283
cg15983520	GPT	8	0.00181696	0.38349489	0.410904721	0.60792318
cg05380921	GPT2;GPT	16	0.00168532	0.1926149	0.234287244	0.380163289
cg26155983	GPX2	14	0.00385922	0.52622214	0.552702074	0.702900167
cg06733347	GRAMD2	15	0.00262867	0.39746227	0.405043364	0.547056918
cg25356006	GRAMD2	15	0.02103823	0.21462615	0.196601623	0.326287196
cg21811450	GRAMD4	22	0.0122594	0.36838481	0.338968061	0.478850667
cg04955856	GRAMD4	22	0.00456803	0.45788089	0.397914333	0.563922435
cg09150232	GRB10	7	0.01530568	0.35453211	0.350270321	0.512454473
cg18047172	GRB10;GI	7	0.00332795	0.20680153	0.205603569	0.34628148

cg02787989	GREB1;GI	2	0.00337574	0.37010507	0.382418786	0.505351695
cg14605150	GREM1	15	0.00339981	0.37230753	0.460379198	0.618615013
cg13651876	GRHPR	9	0.00335524	0.19320469	0.16528087	0.413907786
cg07633435	GRIA1;GF	5	0.00261671	0.34851007	0.333022059	0.537649348
cg19670474	GRID2	4	0.00507632	0.58326304	0.566655366	0.699795686
cg17897352	GRK5	10	0.00092849	0.35611793	0.388757573	0.520628616
cg10399824	GRK5	10	0.00684483	0.59021954	0.622112184	0.737456344
cg13739115	GRK5	10	0.01206679	0.17719183	0.138707985	0.312726657
cg26759179	GRK5	10	0.02087024	0.19262639	0.192386275	0.293215178
cg18001722	GRK5	10	0.01447617	0.30381114	0.320982174	0.555862803
cg16395614	GRK5	10	0.01207774	0.57692538	0.590484204	0.716126825
cg00522048	GRK5	10	0.00066232	0.29165962	0.400981802	0.530923237
cg23537932	GRK5	10	0.00070285	0.47619639	0.574291068	0.722456222
cg24807198	GRK5	10	0.0103127	0.26899199	0.251423686	0.374866728
cg00993388	GRLF1	19	0.00937895	0.43650793	0.464701841	0.60200821
cg25520422	GRM5;GF	11	0.03169761	0.42271709	0.453863436	0.610717667
cg04020590	GRTP1	13	0.00130049	0.28866171	0.340416404	0.518270643
cg01695643	GSC	14	0.00218429	0.21008667	0.332727698	0.472963811
cg23074992	GSC	14	0.00138239	0.16121402	0.269648903	0.398887854
cg13702181	GSTZ1;GS	14	0.00190848	0.24920852	0.253403903	0.436159029
cg05738924	GTDC1;GT	2	0.02304757	0.17707262	0.183860115	0.329664576
cg03598457	GTF3C1	16	0.00109794	0.16187164	0.168877875	0.325153797
cg27372015	GTF3C1	16	0.00158389	0.18363106	0.20830584	0.409372809
cg19202813	GULP1	2	0.01158752	0.44003651	0.449646474	0.630351244
cg03392710	GULP1	2	0.00209505	0.72219287	0.693117506	0.826565012
cg16335858	GYLTL1B	11	0.00065949	0.24024783	0.302058389	0.455586631
cg09025327	H6PD	1	0.00586842	0.18003786	0.200729979	0.320209603
cg04566694	H6PD	1	0.00551242	0.43376366	0.431143172	0.620762704
cg16256342	H6PD	1	0.01789965	0.58556153	0.562181965	0.686716187
cg01566965	HAND2	4	0.00189892	0.15923418	0.24982108	0.369962618
cg27218796	HAND2	4	0.00223317	0.16792847	0.246123807	0.417292441
cg08297751	HAND2	4	0.0032263	0.17809025	0.227754117	0.373361625
cg25325949	HAPLN1	5	0.00403008	0.30184819	0.333854675	0.512373022
cg01045835	HAPLN1	5	0.03129757	0.2521126	0.263641531	0.418077015
cg22006672	HAPLN1	5	0.00233544	0.4699018	0.473781284	0.640720924
cg18156471	HAPLN1	5	0.01109894	0.13014474	0.134392251	0.254120272
cg08317426	HAPLN3	15	0.00594682	0.42919482	0.45160988	0.576423524
cg13420004	HAPLN3	15	0.0070644	0.23328172	0.226562191	0.38900145
cg12828294	HAS2	8	0.0074902	0.39668211	0.473200496	0.627249881
cg20737909	HCCA2	11	0.00158181	0.24437408	0.26415245	0.365113811
cg15470102	HCCA2	11	0.00130219	0.25673	0.286239734	0.455280516
cg03565674	HCCA2	11	0.00077739	0.20875241	0.241014967	0.409043041
cg22979615	HCCA2	11	0.00111057	0.50548656	0.50623229	0.675597688
cg26892308	HCCA2	11	0.0009502	0.43145657	0.434338953	0.537911123
cg24803059	HCCA2	11	0.00113577	0.50661829	0.523364924	0.739052637
cg09947611	HCCA2	11	0.03110358	0.35120308	0.352790594	0.484307506

cg21051815	HCCA2	11	0.00367311	0.26808066	0.240839386	0.413237771
cg25050723	HCCA2;CT	11	0.00141699	0.25709197	0.280917157	0.505137268
cg13978447	HCCA2;LO	11	0.00453677	0.47704831	0.5072426	0.652775574
cg10900641	HCCA2;LO	11	0.00173267	0.28751462	0.264901546	0.428059701
cg08966208	HCCA2;LO	11	0.00741048	0.12891479	0.140821196	0.247702624
cg03934392	HCCA2;LO	11	0.00425638	0.24942228	0.251900881	0.398439245
cg23598212	HCCA2;LO	11	0.00405622	0.24271575	0.242509446	0.349020454
cg13337307	HCRT	17	0.00905411	0.38237775	0.376837404	0.493771167
cg25893831	HDAC1	1	0.01750762	0.407979	0.515331543	0.617815458
cg05446471	HDAC11;I	3	0.00283173	0.23017084	0.230552531	0.367909269
cg06568490	HDAC11;I	3	0.00383289	0.24394795	0.254212388	0.414900853
cg17410431	HDAC4	2	0.00155292	0.10030948	0.111172305	0.24410382
cg03475776	HDAC4	2	0.00092022	0.2611874	0.312223585	0.41398363
cg23870168	HDAC4	2	0.00263648	0.1738454	0.168354385	0.290584711
cg20924425	HDAC4	2	0.00209829	0.32146811	0.289073614	0.549925695
cg09664216	HDAC4	2	0.00359124	0.62714412	0.618841794	0.744122517
cg06855182	HDAC4	2	0.00229687	0.22104283	0.228989508	0.501921177
cg04869380	HDAC4	2	0.00380612	0.21965449	0.232948675	0.401171364
cg19770748	HDAC4	2	0.00258198	0.42851606	0.420688409	0.542844319
cg01641368	HDAC4	2	0.00186784	0.43137981	0.454773323	0.555903078
cg05684907	HDAC9;H	7	0.00851187	0.37173924	0.383270369	0.596373327
cg21464220	HDLBP	2	0.00575746	0.37143093	0.325323949	0.529867637
cg18969029	HDLBP;HI	2	0.0194986	0.5888214	0.609777165	0.717957232
cg09386050	HEATR2	7	0.00221513	0.4722828	0.403388376	0.594726086
cg01743658	HEATR5A	14	0.00322149	0.36656965	0.406720575	0.529428591
cg07570069	HEATR5A	14	0.0020873	0.3761701	0.37732543	0.548271881
cg00213745	HEG1	3	0.00293735	0.53077573	0.556784557	0.670436073
cg14706455	HEG1	3	0.00126466	0.21439308	0.195180865	0.347100673
cg10839322	HERC2	15	0.00101054	0.38056757	0.545871675	0.66690165
cg11774232	HERC2	15	0.00518187	0.53720008	0.500425081	0.64683551
cg20695534	HHAT;HH	1	0.03524554	0.32002452	0.313900173	0.444266932
cg08240383	HHAT;HH	1	0.01047255	0.15124301	0.132084288	0.276168073
cg01123449	HHIPL1	14	0.00414415	0.19833312	0.191080986	0.395757948
cg26025557	HIGD1A;H	3	0.00184462	0.55090489	0.631323079	0.773468353
cg16272084	HIP1	7	0.00144385	0.30336069	0.326725894	0.529186625
cg02025034	HIP1R	12	0.00294138	0.10312939	0.109468487	0.215858095
cg10803309	HIPK3;HIP	11	0.000777	0.73473387	0.764803643	0.868299688
cg03234702	HIST1H3E	6	0.02555878	0.30134476	0.231147593	0.411606047
cg03450107	HIVEP3;H	1	0.00137182	0.63408054	0.652531358	0.800214528
cg27169610	HIVEP3;H	1	0.00108946	0.51270343	0.520005943	0.722949832
cg00542261	HK1;HK1;	10	0.00117377	0.14480673	0.158672603	0.387743145
cg20520115	HK2	2	0.00065949	0.21534389	0.273399406	0.379395423
cg10850215	HLA-DPB1	6	0.00233439	0.20970388	0.152553264	0.335461785
cg20223237	HLA-DPB1	6	0.01783758	0.21318474	0.157377403	0.316094848
cg26228476	HLA-DQB	6	0.04317828	0.44460426	0.413788978	0.553707856
cg17316649	HLA-DRB1	6	0.00855749	0.17036706	0.14492977	0.298779308

cg06032479	HLA-DRB1	6	0.00423803	0.31885251	0.254914637	0.441746489
cg27159583	HLCS	21	0.00866099	0.15475867	0.13308687	0.280984403
cg09515921	HLX	1	0.01039075	0.4796285	0.536957198	0.646064712
cg26440142	HLX	1	0.00976037	0.24448049	0.268563777	0.381965098
cg08655206	HLX	1	0.00233734	0.30387247	0.306217159	0.433819771
cg14531576	HMCN1;N	1	0.00168532	0.16486134	0.196805314	0.354561999
cg09996240	HMG20B	19	0.04716956	0.74754662	0.725906269	0.849926271
cg07355069	HMGCS1;	5	0.0026334	0.45970518	0.514652546	0.65148767
cg13976683	HMGCS2;	1	0.0106258	0.20436479	0.21002219	0.312325985
cg09828010	HMP19	5	0.0015244	0.66250258	0.621858906	0.794418889
cg14818176	HN1;HN1	17	0.04196515	0.49919013	0.447787746	0.629651685
cg19142553	HNRNPA2	7	0.00202182	0.27697206	0.293195475	0.398781168
cg17002899	HNRNPC;	14	0.00218429	0.24635385	0.261717111	0.396762627
cg19817912	HOXA3	7	0.00118326	0.55217198	0.58183545	0.770178085
cg24469729	HOXA3;H	7	0.00081789	0.15286345	0.171654396	0.299586832
cg07094847	HOXA3;H	7	0.00275824	0.45579219	0.497190152	0.678553247
cg12538674	HOXA3;H	7	0.00160273	0.41648008	0.517922968	0.629615101
cg24389585	HOXA5	7	0.00353532	0.39201001	0.419238025	0.628538212
cg05349837	HOXC4	12	0.00330705	0.15625742	0.15025447	0.291132244
cg14345676	HRH2;HR	5	0.01235814	0.22538804	0.227272619	0.388751577
cg24366429	HRNBP3	17	0.00628685	0.27945879	0.277525144	0.387524683
cg24895178	HS3ST3B1	17	0.015222	0.63492356	0.600254745	0.748860715
cg17863312	HS3ST3B1	17	0.03166005	0.56284235	0.555702416	0.6708369
cg11416620	HS6ST1	2	0.00065949	0.33174554	0.381831265	0.547101108
cg27405010	HSBP1	16	0.00161871	0.38562684	0.372454703	0.580458538
cg05315365	HSD17B2	16	0.00359753	0.25500547	0.29515757	0.419653848
cg15854333	HSD17B8	6	0.00652679	0.20476668	0.189186473	0.330236379
cg22896793	HSPA12B	20	0.0023961	0.31223399	0.316541694	0.427635297
cg13320181	HSPB7	1	0.00425638	0.15574982	0.148338872	0.258492363
cg21945949	HSPC159	2	0.002727	0.24481726	0.244502906	0.485821147
cg12636882	HTT	4	0.00436875	0.54851888	0.557994744	0.663739116
cg11173579	HUNK	21	0.03856073	0.25374955	0.228064436	0.36477693
cg08898569	HYLS1;HY	11	0.01477927	0.29129059	0.340699677	0.461127134
cg11962566	HYOU1;H	11	0.00521564	0.24291754	0.242401741	0.361089682
cg21901386	ICAM3	19	0.00525399	0.65193937	0.6606444	0.769711438
cg03065308	IFNGR1	6	0.00159903	0.21491809	0.245052277	0.416001453
cg02102832	IFT140	16	0.00225589	0.50667503	0.53836451	0.740869714
cg08006309	IFT140;TN	16	0.02135859	0.46630586	0.48250469	0.629120883
cg02307681	IFT140;TN	16	0.00474032	0.22457559	0.194241478	0.339694469
cg08880369	IFT140;TN	16	0.00109399	0.49239306	0.46536765	0.61900497
cg07994487	IFT140;TN	16	0.00092056	0.49571079	0.449758456	0.716799057
cg03204177	IFT81;IFT8	12	0.00285259	0.58122168	0.592696794	0.719635772
cg12544411	IGDCC3	15	0.01095648	0.51608323	0.523436749	0.663020263
cg24676120	IGF1;IGF1	12	0.0122594	0.2057463	0.195822513	0.357186664
cg20388729	IGF1R	15	0.00127427	0.53575747	0.585359971	0.738713187
cg02350767	IGF1R	15	0.0021194	0.56523009	0.672997447	0.776748815

cg03795574	IGF1R	15	0.00855749	0.42413629	0.522498248	0.636167502
cg12402183	IGF1R	15	0.00073118	0.1694267	0.250464695	0.463350809
cg05075416	IGF1R	15	0.00525399	0.45663169	0.456120899	0.602123656
cg20966754	IGF2BP1;I	17	0.01093065	0.20102945	0.185665658	0.323629144
cg12754982	IGF2R	6	0.00149088	0.45612058	0.47382497	0.5943362
cg15096505	IL10	1	0.0018234	0.15522726	0.171250984	0.336308626
cg27292389	IL15RA;IL	10	0.00168532	0.49383637	0.537856084	0.726622865
cg25580782	IL15RA;IL	10	0.00371278	0.21287206	0.22489225	0.408842343
cg05042034	IL16	15	0.01154548	0.37712138	0.42127211	0.550857658
cg14899065	IL16;IL16	15	0.02559062	0.2574201	0.302751093	0.46761329
cg14898127	IL16;IL16;	15	0.01910705	0.59234431	0.546857335	0.699777228
cg05860978	IL17B	5	0.00164763	0.31950662	0.359658262	0.480543388
cg03876697	IL17B	5	0.0009934	0.41706518	0.436266587	0.555109636
cg24394667	IL1RL2	2	0.00165724	0.37407082	0.425423794	0.586466018
cg12377972	IL4;IL4;IL4	5	0.01123326	0.59231322	0.607688673	0.71420414
cg07998387	IL6	7	0.0059365	0.43154544	0.435907629	0.57733547
cg05893902	IMPG2	3	0.0460244	0.51224706	0.506714527	0.665824755
cg26122413	INF2;INF2	14	0.00173267	0.4864541	0.391353911	0.657261931
cg05132222	INO80	15	0.00288962	0.27551825	0.299060247	0.450137189
cg16469046	INPP4A;IN	2	0.01092901	0.27127871	0.325070058	0.476312647
cg18455616	INPP4A;IN	2	0.02025944	0.33271721	0.371659721	0.475355805
cg01169610	INPP4A;IN	2	0.0051754	0.476646	0.447406865	0.618780521
cg10773526	INPP4B;IN	4	0.00141131	0.58622487	0.600800549	0.729150705
cg12501287	INPP5A	10	0.04006803	0.45854761	0.577763999	0.765673635
cg07249433	INPP5A	10	0.02957954	0.41471099	0.402416559	0.537741802
cg02836764	INPP5A	10	0.00156239	0.57978233	0.67977027	0.787663235
cg18121190	INPP5A	10	0.00207081	0.44616819	0.448058982	0.574157523
cg02668055	INPP5A	10	0.00218799	0.21975004	0.234271701	0.353100095
cg12449716	INPP5A	10	0.00073378	0.44789551	0.516242657	0.703257021
cg05736824	INPP5A	10	0.02878476	0.68287956	0.73351676	0.838883653
cg21140843	INPP5K;IN	17	0.0016682	0.21252131	0.232199773	0.416910071
cg20720680	INPPL1	11	0.0048909	0.26614348	0.255372787	0.406832741
cg06399933	INSC	11	0.01276611	0.19890864	0.147593391	0.305708711
cg06049367	INSC;INSC	11	0.00113577	0.21956523	0.259737935	0.452133085
cg20523653	INSC;INSC	11	0.00420965	0.14611621	0.149450466	0.286717569
cg09779027	INSR;INSR	19	0.00830906	0.19257784	0.187010017	0.323586773
cg16926712	INTS9;INT	8	0.00279455	0.1420104	0.125992328	0.24564112
cg13577194	IPPK	9	0.00118326	0.5110092	0.422185484	0.672266898
cg14255700	IPPK	9	0.00140443	0.27430685	0.224751447	0.49567257
cg04919592	IQCE;IQCE	7	0.00104452	0.23595052	0.292616637	0.408020521
cg16686273	IQCE;IQCE	7	0.00679946	0.28496525	0.264251332	0.476215356
cg08494871	IQCK	16	0.00729536	0.24445121	0.269894183	0.379522528
cg18459152	IQGAP2	5	0.00276743	0.48202317	0.457455686	0.678924692
cg02109605	IQSEC1	3	0.00302287	0.12498083	0.117057435	0.23496604
cg24380053	IQSEC1	3	0.00220808	0.21488885	0.203778805	0.438188081
cg16192821	IQSEC1	3	0.00328917	0.15291255	0.135662701	0.263959292

cg18000764	IQSEC1;IC	3	0.00524492	0.6471606	0.627707213	0.749272188
cg17554647	IQSEC1;IC	3	0.00139627	0.12162884	0.128276349	0.299597415
cg22016995	IRF7;IRF7	11	0.0052016	0.31332115	0.297364082	0.422514837
cg00305996	IRS1	2	0.00298049	0.14756126	0.14476566	0.29203113
cg00912573	IRS1	2	0.00217555	0.28045647	0.301818176	0.469058946
cg14283647	IRS1	2	0.00092643	0.25261948	0.302050023	0.501956643
cg04129548	IRS1	2	0.00114056	0.21661866	0.262802396	0.399165652
cg13008631	IRS1	2	0.00120091	0.27524807	0.335793147	0.458023933
cg06462663	ISYNA1;IS	19	0.02957954	0.75688406	0.754291476	0.858095327
cg24935345	ITGA1	5	0.00798815	0.57010686	0.548923575	0.730223604
cg15638366	ITGA6;ITC	2	0.03908908	0.19656017	0.229814127	0.349879527
cg03013554	ITGB2;ITC	21	0.0071433	0.43716849	0.436649174	0.564571319
cg05742728	ITGB2;ITC	21	0.00504019	0.54887883	0.603821093	0.734656806
cg13613634	ITGB6	2	0.01163705	0.65703592	0.658020418	0.782783683
cg15889057	ITGB7	12	0.00854289	0.53652917	0.506189751	0.65015763
cg07464094	ITIH3	3	0.00107365	0.43534979	0.44568419	0.554490316
cg18994033	ITM2C;ITI	2	0.00190678	0.15793364	0.210529935	0.311009547
cg16426321	ITPK1;ITP	14	0.00243597	0.53399911	0.575539169	0.695875171
cg09837298	ITPK1;ITP	14	0.00158181	0.5910912	0.615612221	0.75146574
cg05139187	ITSN2;ITS	2	0.00202314	0.21590409	0.219014336	0.390309538
cg26005086	JAG1	20	0.00195	0.35394776	0.312455479	0.536574271
cg12075498	JAK1	1	0.0191912	0.28636459	0.319709778	0.462707083
cg05796838	JAK3	19	0.00295991	0.2842239	0.211943806	0.425304279
cg06493612	JARID2	6	0.00233734	0.31790906	0.323106758	0.490592209
cg22982811	JAZF1	7	0.0103161	0.36645818	0.352592148	0.569869871
cg17702455	JAZF1	7	0.0191912	0.21866967	0.197102842	0.370880098
cg22377814	JAZF1	7	0.01149175	0.46188367	0.496634712	0.629874597
cg17052756	JAZF1	7	0.00083228	0.50121591	0.548987778	0.676676182
cg07128233	JMJD1C	10	0.00144359	0.60884741	0.609390048	0.761249946
cg19495650	JMJD1C	10	0.00803433	0.24358371	0.273387245	0.380228776
cg13038025	JMJD7-PL	15	0.00152988	0.45997687	0.42510346	0.587724956
cg00409260	JRK;JRK	8	0.01661249	0.20919625	0.233012002	0.341304699
cg06690085	KALRN;KA	3	0.00073118	0.0764574	0.110487889	0.212790083
cg14688451	KALRN;KA	3	0.00244246	0.24959324	0.210645774	0.417726514
cg07949011	KANK4	1	0.01630619	0.19692544	0.183383432	0.308548388
cg06133771	KATNB1	16	0.00747631	0.38312593	0.424000122	0.561246201
cg25731943	KCNAB1;I	3	0.00154265	0.15767177	0.154693962	0.290892926
cg15731035	KCNAB2;I	1	0.01135963	0.49884191	0.534312048	0.6397901
cg13377102	KCNAB3	17	0.01076929	0.32522993	0.286022139	0.44262788
cg21017569	KCNAB3	17	0.02143961	0.330596	0.311034263	0.475987452
cg13407335	KCNAB3	17	0.04052436	0.25165561	0.200847156	0.357810695
cg01323777	KCNAB3	17	0.02417619	0.21691678	0.185655089	0.325764704
cg24006352	KCND3;KC	1	0.0225065	0.26733257	0.299107767	0.43324458
cg26736645	KCNE1;KC	21	0.00401283	0.55383962	0.548372565	0.698063352
cg05591210	KCNE3	11	0.00649	0.47572712	0.502739214	0.632644345
cg15510325	KCNH2;KC	7	0.00472916	0.65043732	0.639268448	0.758920864

cg00948250	KCNJ10	1	0.04649715	0.32945538	0.327716843	0.45784089
cg03746851	KCNJ12	17	0.00566397	0.32979469	0.350347463	0.464540632
cg10222534	KCNJ14;K	19	0.00890116	0.2675394	0.192926011	0.404846062
cg21388745	KCNK5	6	0.00160732	0.27657768	0.365282861	0.542658111
cg19427642	KCNMA1;	10	0.00626821	0.38487915	0.384418607	0.578685753
cg07210187	KCNMA1;	10	0.00968951	0.30765385	0.269955583	0.453196918
cg07029975	KCNMA1;	10	0.02897628	0.48573955	0.476693975	0.625945306
cg12614113	KCNMA1;	10	0.00488723	0.36767821	0.376396286	0.593885197
cg24529771	KCNMB3;	3	0.01921828	0.3253716	0.264870893	0.431850561
cg13396821	KCNN3;K(	1	0.00247251	0.20661065	0.202948753	0.389248042
cg26504422	KCNN4	19	0.00169037	0.31156892	0.323341774	0.615301682
cg22904711	KCNN4	19	0.00158899	0.19262973	0.200714334	0.350442983
cg19388050	KCNQ1;K(	11	0.00336646	0.6316296	0.607822292	0.73319745
cg06960356	KCNQ1;K(	11	0.00140062	0.31176051	0.299640985	0.444014861
cg24725201	KCNQ1;K(	11	0.0012838	0.57810987	0.580649373	0.687314185
cg15686157	KCNQ1;K(	11	0.002153	0.2499726	0.288569895	0.389038204
cg04907802	KCNQ1;K(	11	0.00141191	0.49317578	0.548078574	0.754287441
cg08376310	KCNQ1;K(	11	0.00319723	0.2214569	0.246198752	0.391866027
cg13880539	KCNQ1;K(	11	0.01068658	0.23746367	0.190844323	0.365981207
cg15861694	KCNQ1;K(	11	0.003299	0.50222641	0.509572201	0.61153742
cg19672982	KCNQ1;K(	11	0.00263491	0.42066899	0.432320563	0.56768448
cg14245619	KCNQ2;K(	20	0.00986554	0.41085848	0.391705016	0.529367134
cg07338584	KCNU1	8	0.01390151	0.21754803	0.226273057	0.344875776
cg10646179	KCTD16	5	0.04127719	0.27875104	0.313757864	0.4525372
cg02969426	KCTD4;GT	13	0.00065949	0.21360523	0.349596109	0.455025975
cg01080424	KDELR2;K	7	0.00250718	0.26379125	0.191436229	0.428205492
cg01640635	KDELR3;K	22	0.0091896	0.2162057	0.173490871	0.327137327
cg21166985	KDM2B;K	12	0.00315788	0.22025977	0.199664298	0.385215577
cg06646494	KDM2B;K	12	0.00125104	0.40374206	0.403020892	0.507740764
cg06024930	KDM2B;K	12	0.00317234	0.32825816	0.341851418	0.469219094
cg11724984	KDM2B;K	12	0.00067554	0.53619615	0.573885493	0.717207412
cg16062733	KDM2B;K	12	0.00118039	0.22986308	0.298425237	0.472581333
cg25482146	KDM2B;K	12	0.00099652	0.28256568	0.279294996	0.448318863
cg00946598	KDM4A	1	0.00262173	0.44442997	0.437209448	0.588599357
cg02577963	KDM4B	19	0.00112679	0.38725658	0.387312457	0.548707944
cg03358468	KDM4B	19	0.00388972	0.35213902	0.464744555	0.629443123
cg22079102	KDM4B	19	0.00341932	0.37520312	0.419027611	0.540808514
cg20098875	KDM4B	19	0.00864245	0.6051626	0.564332329	0.729374877
cg15705062	KDM4B	19	0.00133856	0.67313552	0.658068095	0.797822144
cg03310779	KDM4B	19	0.00299024	0.57688679	0.550529874	0.69723205
cg02842850	KDM4B	19	0.00524492	0.30296598	0.280614368	0.442532019
cg10821320	KDM4B	19	0.00666067	0.57816917	0.584824692	0.719753171
cg14438634	KDM4C;K	9	0.01980843	0.69224776	0.676919718	0.807476634
cg01586432	KEAP1;KE	19	0.01075636	0.19632227	0.183037671	0.372342754
cg05441854	KIAA0182	16	0.00116059	0.31986054	0.36640869	0.473178994
cg05245094	KIAA0182	16	0.00420965	0.61667876	0.655240735	0.764738102

cg00168118	KIAA0182	16	0.00280849	0.29831985	0.300690821	0.444211698
cg26657404	KIAA0182	16	0.00186737	0.32237852	0.313139583	0.538420347
cg08899895	KIAA0182	16	0.00183351	0.19816254	0.191193664	0.300883268
cg22455529	KIAA0195	17	0.00129968	0.27908977	0.302136419	0.50030741
cg27089714	KIAA0196	8	0.04813395	0.50088573	0.47796775	0.606621113
cg25289457	KIAA0240	6	0.00584178	0.26802445	0.204983973	0.405229804
cg03505866	KIAA0247	14	0.00144775	0.27485193	0.271715178	0.46797677
cg27260684	KIAA0513	16	0.02595684	0.54734802	0.588844697	0.697121346
cg06407371	KIAA0513	16	0.00657278	0.26352419	0.306969741	0.43500771
cg18618674	KIAA0528	12	0.00126804	0.83318762	0.85560071	0.968053306
cg27498545	KIAA0802	18	0.00233476	0.46482041	0.528371299	0.673480333
cg17226446	KIAA0922	4	0.00070134	0.20245833	0.291000571	0.470428288
cg15733729	KIAA1026	1	0.00074381	0.42299336	0.540951103	0.675542913
cg12380772	KIAA1161	9	0.00113577	0.16795945	0.213787476	0.329672599
cg22192454	KIAA1267	17	0.00428303	0.58386058	0.616026665	0.735488684
cg13732302	KIAA1267	17	0.00144365	0.3985657	0.468628537	0.589082512
cg17239876	KIAA1274	10	0.0118868	0.25239703	0.265598894	0.427024079
cg24775327	KIAA1274	10	0.01378915	0.30701178	0.300113731	0.499992144
cg18295923	KIAA1409	14	0.0108413	0.4794472	0.464065558	0.587379571
cg19603836	KIAA1462	10	0.00303542	0.48464284	0.505791992	0.67041616
cg13661330	KIAA1462	10	0.00295256	0.5012803	0.494282356	0.638393146
cg11197101	KIAA1522	1	0.00840111	0.08852181	0.090650529	0.198755389
cg16007395	KIAA1522	1	0.00113577	0.50813486	0.539997776	0.679501243
cg12478578	KIAA1522	1	0.00205947	0.41785485	0.450534529	0.660063198
cg09804503	KIAA1522	1	0.04315457	0.31402241	0.334253375	0.44644518
cg04892182	KIAA1522	1	0.00426288	0.24032538	0.250037183	0.437263972
cg00528191	KIAA1522	1	0.00556574	0.44191514	0.412988308	0.552557431
cg04173688	KIAA1530	4	0.00745107	0.68521487	0.661332802	0.801501824
cg21182616	KIAA1530	4	0.04151822	0.49831433	0.422141552	0.617886053
cg00395625	KIAA1549	7	0.01860545	0.18381679	0.165767127	0.329201979
cg09510085	KIAA1609	16	0.02729523	0.5109042	0.447768613	0.625342451
cg04132007	KIAA1688	8	0.00620309	0.68110373	0.692851979	0.818892756
cg26426395	KIDINS22	2	0.036552	0.2174721	0.182067396	0.367141089
cg23977002	KIDINS22	2	0.00087577	0.56008494	0.585517233	0.784452308
cg09888753	KIF13B	8	0.0011206	0.49792336	0.512948598	0.690733505
cg17876641	KIF21B	1	0.00097946	0.48904693	0.495642879	0.628549586
cg04257913	KIF26A	14	0.02775429	0.45714435	0.430665668	0.575296125
cg26186408	KIF2A;KIF	5	0.01782206	0.51379696	0.563493966	0.667289631
cg24588468	KIF3C	2	0.0472398	0.79914366	0.553662666	0.688600562
cg23186534	KIF5A	12	0.03795947	0.46385718	0.475840926	0.603549934
cg11597157	KIF7	15	0.00288962	0.62400337	0.603298285	0.737030297
cg02161701	KIF7	15	0.00166784	0.46892731	0.499084789	0.672864376
cg00917156	KIFC3;KIF	16	0.00327239	0.27596451	0.291952475	0.432975313
cg04445570	KIRREL3;K	11	0.00380612	0.55512678	0.59062784	0.710028186
cg07140250	KLC1;KLC	14	0.02001759	0.4635219	0.454685485	0.579506291
cg05647197	KLF11	2	0.02001759	0.34289519	0.344302266	0.47216532

cg00540067	KLF15	3	0.00798815	0.18302563	0.185575606	0.289406092
cg02668248	KLF2	19	0.00270297	0.27265528	0.213841674	0.378146977
cg12251895	KLF7	2	0.01783758	0.29670628	0.287468396	0.435156107
cg01304993	KLHDC4	16	0.00442346	0.44523474	0.489873036	0.596271406
cg11705699	KLHDC4	16	0.01039926	0.68151484	0.72287405	0.82792313
cg09821701	KLHDC4	16	0.00275824	0.14697107	0.163964679	0.271604096
cg08356614	KLHDC5	12	0.00713936	0.34659803	0.343674635	0.478033811
cg09502111	KLHDC8A	1	0.00124243	0.55831991	0.58287508	0.746282837
cg00058923	KLHDC9;K	1	0.00298275	0.49386289	0.487239455	0.64784746
cg04359978	KLHL17	1	0.00113844	0.23364111	0.23494831	0.351513794
cg26635367	KLHL2;KL	4	0.00183809	0.22595457	0.200820155	0.422464868
cg01120851	KLHL21	1	0.00272772	0.70346673	0.726233022	0.836043105
cg06371489	KLHL25	15	0.0048909	0.50338025	0.506910729	0.632819196
cg24215531	KLHL26	19	0.00147208	0.25540679	0.278320116	0.379893243
cg27297315	KLHL29	2	0.00435059	0.34881603	0.399305541	0.511655282
cg03445244	KLHL29	2	0.00152273	0.28078626	0.317757462	0.531067232
cg00854172	KLHL29	2	0.00218837	0.14149144	0.130906503	0.325613733
cg14551881	KLHL29	2	0.00107365	0.18644866	0.219598696	0.355767316
cg06676049	KLHL29	2	0.00548794	0.26444715	0.266046398	0.393778337
cg17245125	KLHL29	2	0.00247655	0.29636489	0.29771829	0.402591008
cg00746446	KLHL29	2	0.00245827	0.50979998	0.530643738	0.691632092
cg10982443	KLHL33	14	0.01372997	0.20608477	0.160581882	0.318875363
cg03946012	KLKBL4	16	0.03035371	0.24635472	0.264579035	0.365631504
cg03404742	KLKBL4;C	16	0.01535397	0.37724694	0.325178359	0.485878971
cg15241779	KLRD1;KL	12	0.00168532	0.53065105	0.601130057	0.754957696
cg01940139	KLRD1;KL	12	0.00554761	0.30191686	0.310345857	0.534697202
cg04468334	KRBA1	7	0.00073265	0.42745886	0.473750471	0.609220155
cg21336116	KRT23;KR	17	0.01399795	0.19166283	0.163779944	0.354946709
cg24340657	KRT24	17	0.01405386	0.31106406	0.314493617	0.418084666
cg05138546	KRT36	17	0.02414773	0.65278893	0.542029788	0.767100372
cg05762338	KRTAP1-5	17	0.00574659	0.38403405	0.360024399	0.563922643
cg15322542	KRTAP17-	17	0.0181634	0.22837019	0.214370469	0.330715369
cg12188197	KRTAP2-4	17	0.02601271	0.33868571	0.331224378	0.462774153
cg17158414	KRTCAP3;	2	0.02089413	0.33692245	0.332594581	0.458439905
cg02592271	KRTCAP3;	2	0.00441677	0.29378426	0.316752371	0.427852284
cg20102877	KRTCAP3;	2	0.00317338	0.30271201	0.315172502	0.456711176
cg18428193	KRTCAP3;	2	0.00953692	0.29583562	0.299175734	0.403685435
cg19826115	KYNU;KYI	2	0.04769493	0.34772309	0.39942327	0.529641951
cg19421125	LAG3	12	0.00097337	0.08664671	0.116223143	0.228857433
cg10191002	LAG3	12	0.00144772	0.1779051	0.226492988	0.347556189
cg23613317	LARP1	5	0.0058315	0.13939488	0.131063688	0.244303568
cg02639667	LARP1	5	0.00245827	0.3293151	0.313980552	0.49183881
cg16645907	LARP1	5	0.00150566	0.174986	0.188533783	0.31666753
cg03519907	LARP1	5	0.00279775	0.14481219	0.158688648	0.273345415
cg13689073	LARP1	5	0.00262867	0.12441693	0.136586979	0.318634934
cg04180113	LARP4B	10	0.03209984	0.53994607	0.562037111	0.663561691

cg05380759	LARS2	3	0.00066232	0.10508438	0.160306129	0.278094402
cg01716603	LASP1	17	0.02155924	0.34664773	0.30186037	0.458405254
cg26803993	LASS6	2	0.00841172	0.59209391	0.577856487	0.721068299
cg09045552	LASS6	2	0.00065949	0.60635796	0.701246008	0.803602819
cg27053177	LASS6	2	0.00527173	0.22177585	0.220359766	0.507155997
cg27409974	LATS1	6	0.00186737	0.43960252	0.439997888	0.597592744
cg26525870	LAYN	11	0.00248026	0.47525564	0.472369605	0.643495415
cg19534945	LCN12	9	0.03359449	0.3928184	0.408924629	0.541855489
cg17607330	LDHA;LDH	11	0.00422785	0.36116922	0.413758497	0.524809497
cg19002462	LDLRAD3	11	0.02188973	0.14854454	0.148372056	0.258023467
cg15108490	LDLRAD3	11	0.00317234	0.20203848	0.209681139	0.353249352
cg16943934	LDLRAD3	11	0.036396	0.40692342	0.400930552	0.537094335
cg26597485	LDLRAD3	11	0.00196334	0.15724047	0.155594266	0.37057755
cg06359931	LDLRAP1	1	0.00510852	0.37113167	0.321106232	0.487454593
cg12565679	LECT2	5	0.01045915	0.2316458	0.246632889	0.447867267
cg03607891	LEPR;LEPI	1	0.03409516	0.37150613	0.394359359	0.501762677
cg18968278	LETM1	4	0.02773862	0.34451427	0.374962516	0.49892946
cg16500874	LETM1	4	0.00187837	0.53674409	0.501454127	0.672544531
cg08824221	LGR4	11	0.01311783	0.19184522	0.1631954	0.322141886
cg16964946	LGR4	11	0.04790527	0.36891111	0.352620585	0.516356669
cg23586835	LGR4	11	0.01613643	0.67267755	0.690356592	0.794857603
cg17707870	LHFP	13	0.00480767	0.05756945	0.078102053	0.244286213
cg03499943	LHPP;LHP	10	0.00126804	0.38109664	0.396223941	0.55086183
cg09690597	LHPP;LHP	10	0.00461558	0.23124937	0.178686044	0.343342968
cg26284388	LHPP;LHP	10	0.02514684	0.34420875	0.3288319	0.525491235
cg17995557	LHPP;LHP	10	0.00280852	0.19817578	0.187563254	0.459288411
cg04949429	LHPP;LHP	10	0.01729366	0.2433768	0.261738586	0.411401864
cg21390852	LHPP;LHP	10	0.0108413	0.20763774	0.190683219	0.309802093
cg18593374	LHPP;LHP	10	0.01342633	0.26374904	0.244987061	0.386579015
cg14496753	LHX6;LHX	9	0.00820507	0.2669558	0.217753614	0.379436747
cg14476745	LHX6;LHX	9	0.01616965	0.25735063	0.226982337	0.360274378
cg01578293	LIMCH1;L	4	0.00335972	0.51586914	0.571102963	0.701248505
cg23291145	LIMCH1;L	4	0.00241261	0.30973709	0.267931997	0.449770634
cg23825522	LIMD1	3	0.00298225	0.14222465	0.117122926	0.340191459
cg00225067	LIMD1	3	0.00485896	0.13968534	0.142904446	0.307833202
cg01942372	LIMS1	2	0.00224325	0.41722446	0.500123693	0.642054591
cg23528975	LIMS2;GP	2	0.00499353	0.6281791	0.648174785	0.777442141
cg18044111	LIMS2;LIN	2	0.00184403	0.2846615	0.280488821	0.404959781
cg21869609	LINGO3	19	0.00796638	0.25073848	0.211797905	0.375293541
cg09370594	LINGO3	19	0.00287058	0.38189367	0.364640301	0.484322955
cg09610311	LIPC	15	0.00570747	0.38512244	0.348047492	0.490079865
cg01891736	LITAF;LIT/	16	0.00739749	0.2718769	0.294417858	0.419301526
cg06461408	LLGL2;LL(	17	0.01661249	0.46399376	0.469422128	0.591927251
cg26289155	LLGL2;LL(	17	0.01074953	0.56091012	0.53274702	0.674223265
cg08972916	LLGL2;LL(	17	0.01039075	0.3528465	0.399731915	0.532980639
cg01707127	LMF1	16	0.00624955	0.19031227	0.183500535	0.302987149

cg10244976	LMF1	16	0.00252124	0.22176222	0.211700591	0.348061739
cg16515546	LMF1	16	0.001223	0.4229095	0.457919244	0.63003543
cg17408637	LMO2;LM	11	0.0086408	0.28897932	0.306424385	0.432639293
cg06529818	LOC1001	1	0.00432931	0.23165567	0.210125763	0.358602519
cg01624637	LOC1001	16	0.00255186	0.4380171	0.465357934	0.626754448
cg03579904	LOC1001	16	0.00228672	0.54388977	0.580642723	0.687473332
cg06908618	LOC1001	16	0.00414415	0.27907058	0.286228562	0.487837228
cg03059126	LOC1001	1	0.01332118	0.48415037	0.425815598	0.621949039
cg02694925	LOC1001	18	0.04046963	0.44070809	0.345110344	0.581786625
cg21987921	LOC1001	4	0.00143965	0.5130319	0.514092522	0.666708505
cg02272576	LOC1001	6	0.01980822	0.20398225	0.259168895	0.371625367
cg09248429	LOC1001	11	0.00218429	0.19303828	0.205205034	0.381801233
cg04859117	LOC1001	11	0.00161073	0.33016197	0.375162135	0.588022622
cg18716210	LOC1001	11	0.00275824	0.17481252	0.170471092	0.289212602
cg20494635	LOC1001	11	0.00206218	0.17322025	0.181052083	0.293265545
cg24143374	LOC1001	1	0.00211122	0.33313154	0.379226274	0.503302924
cg04517258	LOC1001	8	0.00140256	0.19528547	0.206688409	0.414087753
cg18034368	LOC1001	10	0.00735604	0.51601468	0.507259185	0.698235601
cg03141090	LOC1001	10	0.00244246	0.74999556	0.749166665	0.855843759
cg23697796	LOC1001	10	0.00814151	0.09256729	0.096832255	0.212673755
cg19192065	LOC1001	10	0.00151706	0.08770043	0.095956011	0.208655175
cg00526835	LOC1001	10	0.00427027	0.17344937	0.14762735	0.367217791
cg03184472	LOC1002	2	0.045184	0.3222152	0.288249377	0.44371279
cg25308803	LOC1002	2	0.00520958	0.19801925	0.19267986	0.338418592
cg03846641	LOC1002	2	0.0031673	0.18047143	0.156221964	0.301130932
cg08390901	LOC1002	2	0.00314038	0.56184857	0.562079843	0.706316081
cg25265234	LOC1003	3	0.01268304	0.33017707	0.391467334	0.494120307
cg14892517	LOC1003	2	0.00669348	0.5159675	0.562904433	0.674380427
cg08611810	LOC1003	2	0.00541343	0.35679616	0.292233407	0.460396051
cg13356253	LOC1003	2	0.02584059	0.38293138	0.337866459	0.494773634
cg00735454	LOC1218	13	0.00375919	0.23250088	0.21643871	0.435798249
cg03826194	LOC1218	13	0.01580038	0.24041425	0.245433255	0.440896259
cg09287356	LOC1219	13	0.00913085	0.43789091	0.438287755	0.588937695
cg21247923	LOC1219	13	0.00074801	0.64498258	0.60270337	0.790848929
cg27053629	LOC1454	14	0.00412512	0.51118418	0.569709312	0.705397032
cg22793142	LOC1486	1	0.00099274	0.48259037	0.528155402	0.663962905
cg20426361	LOC1506	2	0.00931288	0.32142927	0.270170584	0.474171005
cg02969706	LOC1506	2	0.03956605	0.44674403	0.475478374	0.596170341
cg08348461	LOC1511	2	0.00334886	0.65884404	0.661429147	0.784460462
cg17028595	LOC2830	10	0.00527173	0.22680629	0.244401884	0.382802136
cg07580867	LOC2830	10	0.00211122	0.31282955	0.38222542	0.54263408
cg24288715	LOC2857	6	0.00161073	0.49153285	0.524425413	0.678550073
cg05885137	LOC2859	7	0.01598314	0.33422853	0.373362548	0.509522552
cg16415646	LOC2859	7	0.00353865	0.22884042	0.165486248	0.39737646
cg20487548	LOC3395	20	0.00165724	0.55433386	0.596267647	0.701178422
cg06811361	LOC3999	11	0.00456818	0.48149561	0.454780676	0.727578867

cg00927666	LOC40264	7	0.02050584	0.23321622	0.187472624	0.386567786
cg14286546	LOC4401	9	0.02120024	0.58346737	0.603153819	0.753104842
cg00060374	LOC44186	1	0.00556434	0.26739521	0.22099244	0.372167915
cg18233411	LOC5528	12	0.01541996	0.57902032	0.584063223	0.691821584
cg02536995	LOC5531	6	0.00336646	0.23057229	0.224996627	0.385546753
cg00842231	LOC5590	19	0.00094083	0.51578511	0.579591391	0.686022032
cg04946709	LOC64464	16	0.0266726	0.60142894	0.582551071	0.753693289
cg13159985	LOC6469	13	0.00067553	0.70345314	0.747523873	0.858288372
cg20148389	LOC6469	13	0.00182941	0.49531864	0.519909599	0.702526619
cg08569979	LOC6469	13	0.00143343	0.38359173	0.41204659	0.586548002
cg11510891	LOC6469	13	0.00085355	0.21155166	0.23212278	0.365089165
cg13750802	LOC6469	13	0.00314361	0.36718818	0.413776101	0.582472621
cg16314851	LOC72380	7	0.00323713	0.30395004	0.328176791	0.561041337
cg09205190	LOC7283	17	0.00488723	0.20081961	0.1823748	0.346086085
cg09603824	LOH3CR2	3	0.02169667	0.25816747	0.289303798	0.423612159
cg26476173	LONP2	16	0.03749859	0.23461746	0.229844188	0.362231041
cg09916599	LOXL1	15	0.00160451	0.53053598	0.482014869	0.654495153
cg06768079	LOXL2	8	0.00128378	0.41057337	0.429004258	0.66033163
cg04259752	LOXL2	8	0.00232775	0.2240964	0.222766903	0.347981723
cg00326251	LOXL2	8	0.00066232	0.28313289	0.343906001	0.457635924
cg21422605	LPAR3	1	0.00073118	0.32595844	0.375472966	0.517180682
cg00281273	LPCAT1	5	0.00090315	0.18438202	0.241108551	0.377680084
cg20962974	LPCAT1	5	0.00157567	0.54733591	0.554699217	0.665360863
cg21656685	LPIN2;LO	18	0.04896636	0.43127341	0.420581565	0.54223547
cg15052335	LPIN2;LPI	18	0.00468024	0.32684579	0.391405466	0.524078582
cg16224163	LPP	3	0.0437543	0.23613134	0.232860262	0.365815388
cg05048043	LPP;LPP;L	3	0.02544422	0.26873247	0.299457833	0.415774158
cg22486630	LPPR2;LP	19	0.00359691	0.46188385	0.476502045	0.628100694
cg18365406	LPPR2;LP	19	0.00309973	0.29458435	0.302780694	0.407851965
cg08326019	LPPR2;LP	19	0.00334886	0.33005961	0.334252859	0.50854481
cg03729251	LRBA	4	0.02013944	0.23867823	0.18770743	0.345127215
cg26053346	LRIG1	3	0.01438148	0.23660137	0.245234438	0.400405964
cg05543520	LRIG1	3	0.00090291	0.12444689	0.14919522	0.28571915
cg11198596	LRIG1	3	0.00065949	0.09928535	0.145936119	0.307183266
cg22459236	LRIG3;LRI	12	0.00192049	0.47304281	0.45649994	0.694707
cg14621254	LRP1	12	0.00291542	0.25685257	0.232149476	0.389452648
cg12832726	LRP5	11	0.00081789	0.10519808	0.157763167	0.258606656
cg25781963	LRP5	11	0.02362077	0.41839574	0.419974974	0.574006728
cg16409259	LRP5	11	0.00180518	0.24135998	0.290474771	0.493408456
cg13738327	LRP5	11	0.00203531	0.30257116	0.332706809	0.569602067
cg08648997	LRP5	11	0.01305145	0.21585579	0.212769239	0.352177298
cg05754939	LRP5	11	0.00174783	0.23597554	0.241650874	0.402080412
cg11950754	LRP8;LRP	1	0.00079224	0.26008524	0.29646627	0.546420359
cg04483460	LRP8;LRP	1	0.00315365	0.15456359	0.143095602	0.305159444
cg20389822	LRPAP1	4	0.00606409	0.50597307	0.491420571	0.607974799
cg20437668	LRPAP1	4	0.00245414	0.66219548	0.682755703	0.795961466

cg16465012	LRPAP1	4	0.00104158	0.58890357	0.61582592	0.730036173
cg12174175	LRRC1	6	0.02720807	0.30517081	0.340996688	0.441161652
cg19091930	LRRC14B	5	0.03352901	0.35721102	0.350562835	0.457343828
cg01239922	LRRC14B	5	0.00904361	0.1895034	0.180519677	0.304728858
cg26692873	LRRC16A	6	0.0043837	0.54727826	0.558519348	0.715474845
cg16875182	LRRC2;TD	3	0.00099274	0.2988039	0.360464193	0.479251016
cg24602245	LRRC47	1	0.00555917	0.44007642	0.445113435	0.611529798
cg03153397	LRRC48;L	17	0.00953692	0.33965791	0.370590295	0.49528698
cg07566790	LRRC48;L	17	0.00279775	0.25674533	0.280934859	0.426182792
cg21198455	LRRC8A;L	9	0.00117201	0.41877419	0.436557141	0.593265714
cg14206615	LRRC8A;L	9	0.0023323	0.66402073	0.673816153	0.784885666
cg04550950	LRRC8D;L	1	0.00196226	0.14001456	0.154021405	0.2548949
cg17603219	LRRC8D;L	1	0.01098413	0.30063963	0.323488212	0.496108865
cg27373390	LRRFIP2;L	3	0.01479868	0.30024972	0.266455184	0.442746081
cg24305555	LRRFIP2;L	3	0.00558836	0.60488376	0.550948722	0.741878323
cg07165120	LRRK1	15	0.01088059	0.23804767	0.289393479	0.477677716
cg11408395	LRRK1	15	0.00112679	0.64351206	0.660632813	0.803093171
cg27313674	LRRN4	20	0.00299664	0.37418106	0.386311402	0.536272127
cg08963013	LRRTM4	2	0.0190231	0.69252146	0.543114406	0.800190401
cg23352483	LSAMP	3	0.03758612	0.30882151	0.261984542	0.410941653
cg03538814	LSM14A;L	19	0.01170233	0.34690761	0.368055652	0.490023923
cg09245582	LSM4	19	0.0011159	0.38404571	0.407807921	0.647077641
cg05409391	LSP1	11	0.00195	0.77058989	0.756689796	0.875378877
cg05003723	LSP1	11	0.00223531	0.66455771	0.696119321	0.797705206
cg09989681	LSP1;LSP1	11	0.00228505	0.29052023	0.272070146	0.393187606
cg16903735	LSP1;LSP1	11	0.00082808	0.17466674	0.185424661	0.333065183
cg26897904	LSP1;LSP1	11	0.00466622	0.22267724	0.204669192	0.337067077
cg15079934	LSP1;LSP1	11	0.01058353	0.33856315	0.29990652	0.445530684
cg07164388	LTB4R2;L	14	0.01060969	0.56945193	0.561339517	0.67731093
cg18658787	LTBP1;LT	2	0.03358212	0.28679766	0.286952751	0.432471718
cg27317046	LTBP2	14	0.00628266	0.21774333	0.205836519	0.426000122
cg23272978	LTBP3;LT	11	0.00132756	0.48819838	0.532530035	0.654285407
cg22046408	LTBP4;LT	19	0.00295889	0.106322	0.13006754	0.251874649
cg01564640	LTBP4;LT	19	0.00416013	0.35521084	0.316410731	0.480067728
cg26504110	LTBP4;LT	19	0.01093065	0.17736087	0.138042664	0.297535442
cg22706992	LVRN	5	0.00736892	0.42481146	0.325569417	0.542019166
cg18805892	LY9;LY9	1	0.04560477	0.19533145	0.158710739	0.313357904
cg08090164	LYNX1;LY	8	0.00521564	0.20217148	0.206735122	0.36648122
cg11812233	LYPD1;LY	2	0.00704342	0.16805601	0.177419523	0.314795222
cg19753174	LYPLAL1	1	0.00113577	0.20408435	0.233676371	0.43449353
cg02357637	LYSMD4	15	0.00109306	0.19873069	0.225761815	0.375497318
cg14085715	MACC1	7	0.00663932	0.34246125	0.36236386	0.55047878
cg09230154	MACF1;M	1	0.01120712	0.15314545	0.20317288	0.3142901
cg09705456	MACROD	11	0.00088355	0.55802368	0.591964239	0.734318081
cg02374207	MACROD	11	0.00075494	0.14997442	0.164378615	0.312346235
cg04683210	MACROD	11	0.00113577	0.33094364	0.383692304	0.631966014

cg19324462	MACROD	11	0.00099652	0.35492085	0.408035479	0.597486437
cg15769472	MACROD	11	0.00208359	0.17245712	0.170299547	0.288999298
cg16519399	MACROD	11	0.00137728	0.13696213	0.138321154	0.248618866
cg22011888	MACROD	11	0.00160451	0.44219203	0.441826663	0.583500918
cg22963979	MAD1L1;	7	0.00177922	0.20968842	0.246407019	0.441843874
cg00860321	MAD1L1;	7	0.00330705	0.23174316	0.244823932	0.365538781
cg08513622	MAD1L1;	7	0.02595684	0.18878053	0.205037294	0.306765751
cg06624731	MAD1L1;	7	0.0094452	0.35227507	0.36843293	0.507084148
cg02421172	MAD1L1;	7	0.0086963	0.2583233	0.201489598	0.421088618
cg00749118	MAD1L1;	7	0.01766687	0.45214944	0.321544318	0.591958876
cg11852646	MAD1L1;	7	0.00119385	0.48262249	0.491429998	0.657285818
cg17551891	MAD1L1;	7	0.00181052	0.64052452	0.695060281	0.802431929
cg03075889	MAD1L1;	7	0.04937176	0.32569885	0.506986448	0.624891083
cg06994282	MAD1L1;	7	0.00065949	0.61934103	0.701774401	0.804984148
cg05533001	MAD1L1;	7	0.00095227	0.41600053	0.414490776	0.551381775
cg05434287	MAD1L1;	7	0.04489794	0.50730917	0.47145677	0.626040192
cg09118048	MAD1L1;	7	0.00233734	0.18709192	0.201627403	0.315573075
cg10534659	MAD1L1;	7	0.00634238	0.18405733	0.163754144	0.37962143
cg05985070	MAD1L1;	7	0.00352201	0.22127159	0.22505459	0.436900346
cg17498476	MAD1L1;	7	0.00129758	0.18062329	0.185560837	0.293624502
cg15225688	MAD1L1;	7	0.00195	0.19772252	0.229474175	0.334208535
cg24295561	MAD1L1;	7	0.00478595	0.38975348	0.437599389	0.552566895
cg17103856	MAD1L1;	7	0.0037804	0.45469871	0.525807646	0.657436645
cg21004924	MAD1L1;	7	0.00302287	0.4638076	0.535969103	0.668297452
cg09075743	MAD1L1;	7	0.01744696	0.48671977	0.49747727	0.611065336
cg12964697	MAD1L1;	7	0.00079018	0.59179764	0.631613453	0.732746652
cg18319104	MAD1L1;	7	0.01191142	0.64106614	0.626962568	0.749677928
cg04596071	MAD1L1;	7	0.00486966	0.27298727	0.271466253	0.416506057
cg27238358	MAD1L1;	7	0.00091163	0.55011311	0.60690346	0.709308051
cg16222367	MAD1L1;	7	0.00157286	0.34801474	0.39740594	0.599041351
cg04863758	MAEA;MA	4	0.00967401	0.17137117	0.163380456	0.286873452
cg03207121	MAFK	7	0.00913085	0.32049497	0.318304589	0.438545128
cg00650672	MAGI1;M	3	0.02289623	0.4086156	0.447743378	0.564141105
cg25972943	MAGI2	7	0.01387172	0.25815515	0.282028526	0.411567326
cg19755714	MAML3	4	0.01506509	0.42861039	0.477609584	0.592965453
cg03127558	MAMSTR	19	0.0084821	0.47336681	0.481332712	0.620479199
cg10404009	MAMSTR	19	0.00710689	0.12960208	0.1301861	0.253484794
cg24661274	MAP1B	5	0.00120091	0.37839282	0.436490678	0.628467364
cg04589021	MAP2K3;	17	0.00158722	0.22148916	0.207877885	0.400800145
cg26779305	MAP2K4;	17	0.0017385	0.16482878	0.18025973	0.360952114
cg04798016	MAP3K11	11	0.01134767	0.35105967	0.30627514	0.466768289
cg26038649	MAP3K6	1	0.0009934	0.25318406	0.268524964	0.409779038
cg02634454	MAP4;MA	3	0.0026461	0.47553763	0.524908398	0.642411838
cg04388901	MAP4;MA	3	0.0190357	0.57030621	0.559185335	0.704507843
cg18489755	MAP6D1	3	0.0025935	0.15271563	0.190355982	0.328444963
cg22117438	MAPK10;	4	0.00267111	0.6885931	0.699643545	0.83076714

cg16054907	MAPK11	22	0.00608941	0.24123768	0.223232968	0.353935163
cg04059696	MAPRE3	2	0.00109306	0.31249182	0.35119445	0.456166099
cg06982473	MARK2;M	11	0.0017731	0.25223624	0.253732547	0.400911487
cg09034795	MARK2;M	11	0.00220808	0.21534297	0.206329291	0.402514128
cg10689689	MARK2;M	11	0.00156239	0.16993238	0.175306371	0.302560404
cg21609804	MARK2;M	11	0.00412385	0.20730069	0.166876519	0.315377405
cg18140824	MASP1;M	3	0.00197403	0.340518	0.357921456	0.581315179
cg17462107	MAST4;M	5	0.00260306	0.20904389	0.216716226	0.391208023
cg04348183	MATN1	1	0.00188718	0.60147977	0.578049644	0.738389075
cg17461278	MATN1	1	0.00306113	0.35450653	0.366345503	0.532692378
cg14183455	MATN1	1	0.00094183	0.62067483	0.628779305	0.763938761
cg24531825	MATN1;M	1	0.0023187	0.50267099	0.524916826	0.656319266
cg21745307	MATN4;R	20	0.00097509	0.38383602	0.4510172	0.633733184
cg12429065	MB	22	0.00142756	0.23039129	0.275954377	0.478545109
cg02312594	MBD3	19	0.02995719	0.56671364	0.523371179	0.677665528
cg25006077	MBNL1;M	3	0.00093078	0.09387596	0.160964283	0.281819189
cg06155414	MBNL2;M	13	0.04270907	0.4643524	0.46570065	0.594521834
cg11862665	MBNL2;M	13	0.0010175	0.43566331	0.510489856	0.662977271
cg15683856	MBNL2;M	13	0.02743413	0.5335701	0.548559943	0.65968125
cg15676677	MCC;MCC	5	0.02389893	0.12506514	0.112359724	0.251061413
cg02481669	MCCD1	6	0.00896808	0.46484439	0.437567816	0.586102703
cg21609154	MCF2L	13	0.01038208	0.32489269	0.296693364	0.430052035
cg21847368	MCF2L;M	13	0.03474198	0.30462177	0.338977823	0.445051889
cg15730074	MCF2L;M	13	0.00564684	0.55453439	0.625793829	0.746498324
cg16277139	MCF2L;M	13	0.00539451	0.51417517	0.530943221	0.694084957
cg20732304	MCF2L;M	13	0.01289854	0.33522834	0.30976385	0.448074451
cg06264060	MCHR1	22	0.00841345	0.19755166	0.157061848	0.302890189
cg02621376	MCOLN2	1	0.00779924	0.15248972	0.140575143	0.253877719
cg19511748	MCTP1;M	5	0.00758361	0.54888942	0.543084726	0.676197826
cg14558568	MDFI	6	0.00198593	0.2088418	0.222692266	0.328358409
cg23671901	MDFI	6	0.00089487	0.30486855	0.336552497	0.444120284
cg12328429	ME3;ME3	11	0.00139627	0.41455452	0.479928577	0.645674492
cg09416908	ME3;ME3	11	0.01342633	0.35875459	0.404052616	0.535995499
cg01384290	MECOM	3	0.0075495	0.50456872	0.497127506	0.639658476
cg15877399	MED13L	12	0.02195382	0.49549078	0.450967212	0.644400343
cg05133323	MED13L	12	0.00468549	0.55711937	0.602248314	0.703508202
cg15412772	MED24;M	17	0.00065949	0.11095947	0.169256204	0.320017496
cg14300812	MED27	9	0.00485896	0.72375072	0.737865719	0.841774641
cg16126137	MEG8	14	0.00142756	0.1086797	0.12195472	0.246869226
cg26450275	MEGF10	5	0.00422345	0.45837258	0.421515028	0.653110064
cg22760690	MEGF11	15	0.00629716	0.47579707	0.385400643	0.591357659
cg06511312	MEGF6	1	0.00576931	0.32060309	0.270156613	0.463583719
cg27450668	MEGF6	1	0.00744839	0.291611	0.228303816	0.411747931
cg20826162	MEGF6	1	0.01643293	0.19399036	0.153774933	0.294560819
cg18553657	MEGF8	19	0.00180551	0.48556339	0.513427608	0.615135718
cg17945976	MEIS1	2	0.00129758	0.05083793	0.111953611	0.213900799

cg04288999	MEIS1	2	0.00158297	0.0620715	0.124638951	0.235335722
cg05877497	MEIS1	2	0.00099274	0.09037555	0.185163114	0.322046755
cg26537478	MEIS1	2	0.00124625	0.10471389	0.182579139	0.308280441
cg01271812	MEIS1	2	0.00092849	0.12152126	0.200428252	0.34250914
cg09535924	MEIS1	2	0.00290119	0.21351855	0.254396314	0.430181838
cg12082609	MEIS1	2	0.00108543	0.11694856	0.180240855	0.31279964
cg09550083	MEIS1	2	0.00543186	0.20326956	0.281835155	0.466206605
cg06994420	MEIS1	2	0.00679225	0.34799333	0.361752471	0.51181836
cg10464312	MEIS1	2	0.00092643	0.16638842	0.23363133	0.35970276
cg14775296	MEIS1	2	0.00868337	0.15652235	0.173530081	0.274211567
cg06833110	MEIS1	2	0.0012898	0.08766502	0.165928578	0.295682849
cg08238215	MEIS1	2	0.00165724	0.11727227	0.173164519	0.276947711
cg04339553	MEIS2;MI	15	0.00741048	0.32305691	0.356474439	0.554186126
cg25447144	MEIS2;MI	15	0.00417366	0.52816652	0.568558864	0.687494609
cg25221496	MEIS2;MI	15	0.01060969	0.52892686	0.511287246	0.744210341
cg21177426	MEIS2;MI	15	0.00073118	0.22398474	0.314809153	0.593249761
cg15902390	MEIS2;MI	15	0.00147518	0.12724435	0.199224315	0.32105838
cg26708220	MEIS2;MI	15	0.00132935	0.15358077	0.226855099	0.364011561
cg19010490	MEIS2;MI	15	0.00183659	0.26239887	0.299844513	0.504621991
cg02377544	MEIS2;MI	15	0.00106594	0.29605495	0.325224127	0.491436476
cg13181164	MEIS2;MI	15	0.00108543	0.09173825	0.170071018	0.277677909
cg26585007	MERTK	2	0.00253153	0.53741429	0.572092129	0.718691203
cg13831540	MESTIT1;	7	0.00339981	0.25930412	0.229038587	0.410977869
cg03260530	METRNL	17	0.00180551	0.27027774	0.309752284	0.483834188
cg16002136	METRNL	17	0.00103509	0.61017328	0.604608902	0.765319336
cg22846776	METT10D	17	0.00337229	0.54965827	0.548600144	0.669003504
cg24035107	METT10D	17	0.00753069	0.13709549	0.140486193	0.302116902
cg02148547	METT11D	14	0.02990873	0.33211392	0.360470801	0.489751611
cg16424082	METTL7A	12	0.00488538	0.17821389	0.152073175	0.317449018
cg11717597	MEX3A	1	0.00081928	0.18575256	0.240812255	0.369782675
cg26396535	MEX3A	1	0.01102429	0.51433592	0.551968338	0.687081961
cg02895724	MEX3C	18	0.0252514	0.62275013	0.619290862	0.739187618
cg21304454	MFAP2;M	1	0.00228672	0.2261318	0.227361186	0.403200577
cg01627785	MFI2;MFI	3	0.00393586	0.3300956	0.367671462	0.477296041
cg00637433	MFI2;MFI	3	0.00113894	0.46124805	0.463312053	0.718448064
cg03784611	MFI2;MFI	3	0.00893433	0.3214221	0.311072572	0.467972479
cg21856334	MFI2;MFI	3	0.00233734	0.3695846	0.378332117	0.513561682
cg04498801	MFSD10;I	4	0.00352201	0.4735168	0.50707579	0.638208322
cg16924045	MFSD6	2	0.00588734	0.25020484	0.236001629	0.476168597
cg19391560	MFSD6L	17	0.00131453	0.2190094	0.233324595	0.338102476
cg18432414	MFSD6L;I	17	0.00287532	0.27179957	0.246642864	0.376022938
cg11685316	MFSD6L;I	17	0.00395854	0.19643247	0.16016295	0.318651593
cg07382437	MGC1443	12	0.00252984	0.43352711	0.497619134	0.605764697
cg11842367	MGC2738	1	0.00223531	0.13107746	0.149301985	0.28208684
cg18297349	MGC2950	5	0.00552473	0.4125082	0.430578012	0.540538942
cg18811130	MGMT	10	0.02774083	0.52341294	0.562304738	0.672777241

cg25974017	MGMT	10	0.00917603	0.18517448	0.138171793	0.286361481
cg13171643	MGMT	10	0.00488538	0.34766935	0.35013835	0.527596897
cg18502933	MGMT	10	0.00796332	0.53327049	0.53768568	0.654163322
cg08883995	MGMT	10	0.01698967	0.27559271	0.293212378	0.437511592
cg02750154	MGMT	10	0.02291377	0.41340491	0.393929618	0.52567726
cg25145165	MGMT	10	0.00258198	0.39695393	0.435870759	0.553164934
cg08147187	MGRN1;N	16	0.00747631	0.65868012	0.631390035	0.775887476
cg01156249	MGRN1;N	16	0.00433996	0.30752328	0.359832426	0.484294733
cg27436118	MGRN1;N	16	0.00111057	0.13263378	0.145008965	0.276063977
cg00736299	MGRN1;N	16	0.00073118	0.21572325	0.176815175	0.348409425
cg02074956	MGRN1;N	16	0.00137728	0.2321141	0.233319077	0.346907429
cg10505257	MGRN1;N	16	0.00084371	0.23683886	0.224677322	0.476441958
cg07812289	MGRN1;N	16	0.0019386	0.50137708	0.486787448	0.63886171
cg17456675	MIB2;MIE	1	0.0023961	0.40369694	0.408673415	0.562439325
cg17529716	MICA	6	0.03969411	0.67424204	0.649198005	0.790634288
cg09210225	MICA	6	0.00602378	0.49598379	0.49961586	0.643558142
cg23080546	MICA	6	0.00286384	0.24917703	0.212903098	0.395725847
cg15242630	MICAL3;N	22	0.01440243	0.15921192	0.13883148	0.283123039
cg02759751	MICALL2	7	0.00113577	0.53654341	0.544077533	0.736791693
cg05242402	MICALL2	7	0.00092643	0.51804011	0.514747543	0.658051821
cg11251690	MICALL2	7	0.00127403	0.52311146	0.532267655	0.67277185
cg02492818	MICALL2	7	0.00131453	0.45497043	0.452847115	0.672287985
cg22972565	MICB	6	0.00953692	0.50220961	0.520461145	0.64283228
cg14773523	MINA;MI	3	0.01690382	0.42110045	0.377786687	0.532013354
cg17649671	MINPP1	10	0.00174721	0.12148466	0.099364953	0.254536948
cg27621931	MIPEP	13	0.01235814	0.16627493	0.171949018	0.29823909
cg18222371	MIR101-1	1	0.00208745	0.63485955	0.621162221	0.736755004
cg20841588	MIR1246	2	0.01934763	0.4651153	0.494611083	0.607162884
cg03417340	MIR140;V	16	0.00081928	0.12131667	0.144095615	0.309338925
cg00699693	MIR140;V	16	0.00165724	0.19979266	0.235706781	0.427788649
cg07281938	MIR140;V	16	0.00078196	0.22234595	0.29547023	0.483609516
cg03848856	MIR140;V	16	0.00233734	0.13655203	0.130833074	0.281603534
cg03549146	MIR140;V	16	0.00075421	0.11019514	0.172161773	0.28773027
cg00158530	MIR140;V	16	0.0011345	0.10406504	0.115213482	0.278912628
cg08209934	MIR140;V	16	0.00427549	0.12782688	0.14231915	0.268482883
cg26514117	MIR181D	19	0.00183809	0.52944203	0.518540833	0.672610028
cg26754262	MIR2052	8	0.00802316	0.09543997	0.101596226	0.202343699
cg02268620	MIR24-1;	9	0.00089174	0.61155547	0.644628043	0.810039488
cg15076217	MIR29C	1	0.00091042	0.52750881	0.521952809	0.663760552
cg02875879	MIR302A	4	0.02817047	0.44213175	0.426732885	0.556410131
cg04390865	MIR320B	1	0.03538972	0.19566471	0.164570732	0.315679281
cg20632224	MIR320B	1	0.02003709	0.1423178	0.139224289	0.265462413
cg18163364	MIR377	14	0.0103161	0.53189865	0.518476236	0.650776176
cg11134801	MIR377	14	0.01678972	0.31687821	0.300901294	0.430746942
cg10006887	MIR377	14	0.00912728	0.26480093	0.246211038	0.377846759
cg04522625	MIR548A	3	0.0013706	0.44330283	0.495598706	0.649081847

cg18697991	MIR548A	3	0.00935896	0.15478531	0.174299386	0.308568771
cg23036340	MIR548H	15	0.00964959	0.26337669	0.252268418	0.39839288
cg09031823	MIR575;S	4	0.02265546	0.23299323	0.204130118	0.348759574
cg20206204	MIR614	12	0.01006689	0.50432372	0.501686507	0.626617393
cg05346831	MIR614	12	0.03453375	0.42929897	0.440287844	0.543925613
cg18156845	MIR744;N	17	0.00124243	0.34138845	0.471644581	0.637123421
cg06549228	MIR758;N	14	0.00133856	0.56355897	0.584499178	0.695508215
cg22030962	MIR874;K	5	0.0055506	0.42304662	0.421166062	0.597395384
cg00926267	MKL1	22	0.00197789	0.35878365	0.36349	0.527794481
cg00434573	MKNK2;M	19	0.00079512	0.25538106	0.308892353	0.422176613
cg07723431	MKNK2;M	19	0.00105733	0.37527093	0.341872407	0.504238699
cg07746514	MLEC	12	0.0042054	0.49177545	0.552354327	0.684243654
cg11872373	MLL3	7	0.00467005	0.25154127	0.2070103	0.387597641
cg25076197	MLL5;ML	7	0.00921039	0.2855326	0.358407289	0.493051782
cg01969558	MLLT1	19	0.01233628	0.57842222	0.585836685	0.692955567
cg22501243	MLXIP	12	0.00479693	0.20091331	0.151934483	0.345591731
cg14589810	MMP28	17	0.00536676	0.3869223	0.431665171	0.537563147
cg00187327	MN1	22	0.01021297	0.2613643	0.29284399	0.441757611
cg22708961	MORN1	1	0.00630644	0.28193503	0.24095587	0.38402416
cg08868601	MORN1	1	0.00106082	0.38439164	0.399147219	0.587200242
cg16600733	MORN1	1	0.00645854	0.17717326	0.151711667	0.301042344
cg05787839	MORN1	1	0.00113894	0.23217184	0.26767395	0.38517623
cg09811127	MORN1;L	1	0.00147518	0.45124987	0.520844043	0.632202039
cg20120371	MPP2	17	0.01473127	0.27455238	0.246998648	0.413839042
cg15406585	MPP7	10	0.00073547	0.58897539	0.636564856	0.775032885
cg14133850	MPP7	10	0.00471011	0.38926677	0.493710006	0.629547587
cg16527477	MPP7	10	0.00659076	0.24243007	0.302760933	0.403911257
cg09850478	MPPED1	22	0.00702941	0.22436042	0.195032573	0.361817425
cg20545941	MPPED1	22	0.00917702	0.22608965	0.20157969	0.352197092
cg03289072	MRC2	17	0.00355421	0.56870627	0.36105388	0.46169792
cg16405908	MRGPRE	11	0.00675316	0.29192821	0.259432782	0.419291809
cg25807487	MRGPRF;	11	0.01810942	0.24595467	0.263962531	0.392641591
cg18850728	MRO;MR	18	0.01092179	0.25421155	0.235508649	0.373372685
cg11375053	MRPL17	11	0.02595684	0.46579887	0.420515092	0.57481742
cg02928928	MRPL23	11	0.00119376	0.1857221	0.190255883	0.338558549
cg01530482	MRPL23	11	0.01687692	0.5599964	0.602830332	0.711265507
cg17519037	MRPL45	17	0.00186737	0.26160267	0.26055145	0.36342832
cg06995967	MRPS16;T	10	0.00655853	0.23517385	0.230910284	0.406373688
cg04955683	MRPS6	21	0.00569156	0.18997754	0.155279153	0.312543367
cg02556430	MRPS6;SI	21	0.00197986	0.38215262	0.37822478	0.625230416
cg21291385	MRPS6;SI	21	0.00100561	0.07939634	0.098375608	0.207739137
cg08298091	MRVI1;M	11	0.00319723	0.16409993	0.143162858	0.285795013
cg00510149	MRVI1;M	11	0.00079883	0.4020394	0.512293605	0.652437271
cg22762189	MSGN1	2	0.01543666	0.41269714	0.42661413	0.549063134
cg22149471	MSI2	17	0.00359691	0.25076941	0.261165208	0.494216669
cg18875496	MSI2;MSI	17	0.03227846	0.28320738	0.323932505	0.430151313

cg13654836	MSI2;MSI	17	0.00073118	0.19905919	0.264971347	0.427424422
cg07159758	MSI2;MSI	17	0.00248169	0.21602417	0.241922909	0.46318551
cg08677210	MSI2;MSI	17	0.02203216	0.37528694	0.4200692	0.531048237
cg08788712	MSI2;MSI	17	0.0283256	0.53040963	0.536603578	0.66817475
cg17045539	MSI2;MSI	17	0.00100115	0.56425615	0.600689371	0.717589895
cg01030886	MSRB3;M	12	0.00381594	0.63828254	0.661676193	0.797262435
cg21538208	MSX1	4	0.00081599	0.21293271	0.297077029	0.429251542
cg09748975	MSX1	4	0.00279442	0.36678568	0.414866942	0.554881399
cg19001023	MTA1	14	0.00297992	0.45983485	0.461746361	0.684438709
cg18238340	MTA1	14	0.0106654	0.36664041	0.342991929	0.503141779
cg24183741	MTA1	14	0.00153162	0.59332071	0.607119806	0.759265247
cg27055366	MTA1	14	0.00197007	0.25326875	0.246362444	0.378267137
cg18920088	MTHFD1L	6	0.00094083	0.25121632	0.266721491	0.55364392
cg02285003	MTMR12	5	0.00857076	0.23929667	0.220336984	0.412252064
cg06740866	MTMR12	5	0.00937895	0.66460856	0.685865192	0.792022487
cg24683185	MTMR15	15	0.00457113	0.30195821	0.351026011	0.510296828
cg14256977	MTMR2;M	11	0.00589356	0.17600055	0.150680441	0.279555259
cg06094043	MTMR6	13	0.02336655	0.41112455	0.430921248	0.545035524
cg07122170	MTOR;AN	1	0.04030438	0.2840728	0.251382261	0.426911124
cg23629187	MTSS1	8	0.0457117	0.58904782	0.543485715	0.689984255
cg27038717	MTSS1	8	0.02773862	0.42139298	0.451431233	0.553275558
cg08996445	MTSS1L	16	0.013012	0.24765026	0.2144585	0.386371398
cg00147286	MTSS1L	16	0.00126059	0.52262119	0.541979513	0.672750819
cg07128900	MTSS1L	16	0.00098577	0.54793018	0.563175503	0.742050031
cg19075081	MTSS1L	16	0.00463097	0.36145877	0.347958504	0.518843609
cg07813065	MTSS1L	16	0.00115277	0.35517392	0.368036193	0.54501736
cg01304894	MTSS1L	16	0.00110296	0.30310513	0.310538886	0.496538371
cg04898035	MTUS1;M	8	0.02149232	0.48554882	0.524263556	0.663202655
cg02698708	MTUS1;M	8	0.00349176	0.35012246	0.364923571	0.488592892
cg25566714	MUM1;M	19	0.00073378	0.7151523	0.767141544	0.907735745
cg13937462	MUPCDH	11	0.01773077	0.4273026	0.402921282	0.52755281
cg14161107	MUSK;MU	9	0.02096205	0.38753339	0.342379067	0.521571135
cg04863197	MYADML	17	0.00893433	0.30197173	0.292522607	0.425721082
cg13189020	MYBBP1A	17	0.00174903	0.25094679	0.230305292	0.392278724
cg15598069	MYH10	17	0.03708299	0.57359476	0.591661699	0.72509924
cg25930644	MYH10	17	0.00173053	0.35074843	0.400208406	0.536164624
cg01828111	MYH11;N	16	0.00539963	0.60117873	0.631157593	0.742855474
cg26749543	MYH9	22	0.00182655	0.27639863	0.262243454	0.427058636
cg24899871	MYL10	7	0.00591291	0.32236222	0.320228692	0.474894525
cg10123201	MYL3	3	0.00140256	0.34835026	0.386216809	0.545996303
cg08936706	MYL3	3	0.00576931	0.3537987	0.34291195	0.484549363
cg19007948	MYLK4	6	0.00274286	0.1948652	0.189976795	0.394264274
cg05713044	MYO10	5	0.00787909	0.16171012	0.159247407	0.310807037
cg26264133	MYO18A;	17	0.00295909	0.54111078	0.430772912	0.65951688
cg20029153	MYO18A;	17	0.00073118	0.18183788	0.245721285	0.380855418
cg03134230	MYO1B;M	2	0.02355792	0.42681987	0.368650379	0.544774967

cg22987448	MYO1F	19	0.00551458	0.23389124	0.183400157	0.355578877
cg13523014	MYO1F	19	0.00186737	0.6350455	0.61168425	0.797113811
cg19089201	MYO1G	7	0.00858426	0.31051078	0.283152342	0.42096809
cg22132788	MYO1G	7	0.00213825	0.32813822	0.297378646	0.485303422
cg01379730	MYO3B;M	2	0.00214604	0.61108148	0.637607739	0.758916054
cg14100211	MYO9A;S	15	0.00079512	0.42349016	0.469867372	0.672856278
cg16526705	MYO9B;M	19	0.00344865	0.59443935	0.591780822	0.704844572
cg01410279	MYOC	1	0.01880235	0.25990282	0.276736098	0.466026018
cg00806505	MYOC	1	0.00670577	0.3992237	0.38937236	0.553433709
cg04852893	MYOCD;M	17	0.00948686	0.29235715	0.282095952	0.473545544
cg23636406	MYOCD;M	17	0.00480767	0.27649504	0.27028868	0.45780897
cg17800426	MYOZ3;M	5	0.01040861	0.15893575	0.15289048	0.282920095
cg13214422	MYOZ3;M	5	0.00643366	0.58946263	0.604777391	0.727455681
cg08549216	MYST1;M	16	0.00353146	0.61292707	0.614746697	0.74057624
cg15370732	MYST2	17	0.00202314	0.57667292	0.589649166	0.756669761
cg11849327	MYST4	10	0.00465176	0.34414188	0.40161709	0.545138941
cg03852659	MYT1L	2	0.02385226	0.18357857	0.165522149	0.296687812
cg02493986	NACC2	9	0.00337598	0.36242075	0.345851532	0.626189751
cg14152854	NACC2	9	0.00553981	0.54403439	0.569897903	0.694926998
cg23954731	NAT14	19	0.0042005	0.33510752	0.323009432	0.436501765
cg19827875	NAV1	1	0.00368543	0.32155407	0.305304698	0.455218985
cg03400139	NAV1	1	0.0135118	0.32262453	0.303683525	0.42701314
cg05695876	NAV1	1	0.00180422	0.1986412	0.168920427	0.342276847
cg14969261	NAV2	11	0.0009934	0.30014249	0.346066041	0.55846542
cg00491064	NAV2	11	0.00205947	0.18610628	0.202206216	0.351609835
cg18009574	NAV2;NA	11	0.00354346	0.25201242	0.263620321	0.421773662
cg09571376	NAV2;NA	11	0.03576735	0.41389709	0.449669993	0.562485937
cg23371436	NAV2;NA	11	0.00156458	0.28982525	0.35327647	0.495006052
cg01816186	NBAS	2	0.00084118	0.32590699	0.38325487	0.587740699
cg09936572	NBPF1	1	0.00911882	0.31934439	0.330320546	0.470529219
cg19865916	NCDN;NC	1	0.00078196	0.39371064	0.459026638	0.640051082
cg19758142	NCEH1;N	3	0.00663444	0.4433776	0.414609053	0.66499863
cg03729042	NCF2;NCF	1	0.00521564	0.28055686	0.331150474	0.465533983
cg22687475	NCK2;NCK	2	0.00252124	0.72347419	0.711275094	0.833769628
cg10998894	NCLN	19	0.03190657	0.51706171	0.555009042	0.655648036
cg27015161	NCOA1;N	2	0.00298275	0.23619995	0.217769826	0.468142453
cg08325898	NCOA2	8	0.00947108	0.37169213	0.393664238	0.535444124
cg13335567	NCOA2	8	0.01040141	0.17845942	0.203619137	0.34250726
cg04544533	NCOR2;N	12	0.02551242	0.58532545	0.535500887	0.695622188
cg11197258	NCOR2;N	12	0.00156869	0.22163822	0.243471703	0.518315686
cg10283674	NCOR2;N	12	0.00239775	0.45452204	0.42178142	0.5629661
cg10082088	NCOR2;N	12	0.00342408	0.2889593	0.301503265	0.449982246
cg10849160	NCOR2;N	12	0.00561885	0.18266623	0.160712161	0.285480002
cg08276543	NCOR2;N	12	0.00147873	0.41105097	0.392549832	0.520902039
cg24432389	NCOR2;N	12	0.00259677	0.33735852	0.33172038	0.446921376
cg17429587	NCOR2;N	12	0.00309287	0.51551425	0.497729713	0.665322555

cg09831875	NCOR2;N	12	0.02562377	0.23107625	0.199411282	0.335848985
cg03406367	NCOR2;N	12	0.00971274	0.147289	0.134757468	0.26577669
cg19341309	NCOR2;N	12	0.00115308	0.18055621	0.210552646	0.392281136
cg22611259	NCOR2;N	12	0.00065949	0.67472938	0.733648563	0.838664674
cg20494738	NCOR2;N	12	0.00073265	0.58507911	0.672878164	0.84905526
cg27044649	NCOR2;N	12	0.00141573	0.29225961	0.371830231	0.562363572
cg10996589	NCOR2;N	12	0.0011122	0.29099306	0.333682059	0.553381413
cg04579183	NCRNA0C	15	0.00133856	0.40680716	0.486341692	0.734612571
cg10859327	NCRNA0C	10	0.0034485	0.21303711	0.234395098	0.505511319
cg03938110	NCRNA0C	21	0.00411808	0.22939447	0.231337607	0.393120961
cg02532853	NCRNA0C	21	0.00751269	0.32306559	0.309613541	0.494939671
cg13049432	NCRNA0C	21	0.00352201	0.47869634	0.52099784	0.64836898
cg17611512	NCRNA0C	21	0.00910457	0.2636446	0.257142821	0.38930862
cg25623524	NCRNA0C	19	0.00882698	0.19910476	0.152312742	0.307379096
cg02396982	NDE1;ND	16	0.00312811	0.52143644	0.526178412	0.657812719
cg10078898	NDRG2;N	14	0.00208072	0.15569243	0.157127905	0.261544377
cg04359602	NDRG2;N	14	0.01713917	0.17845695	0.18566723	0.323161525
cg08895618	NDST1	5	0.00591073	0.13581164	0.127302414	0.255074693
cg16674484	NDST1	5	0.00219431	0.19853721	0.263245815	0.392014695
cg02633363	NDST1	5	0.02833129	0.35111475	0.351701532	0.457369747
cg15337815	NDST1	5	0.00341932	0.4523429	0.475431341	0.577492718
cg08733553	NDUFAB1	16	0.00332487	0.63173053	0.607348426	0.745685142
cg05774231	NDUFB4;I	3	0.03354729	0.36121769	0.409338294	0.516888803
cg00525496	NEDD9;N	6	0.0261625	0.48737361	0.471864053	0.592697093
cg13974765	NEK6;NEI	9	0.00317466	0.2456938	0.239651504	0.360016194
cg19624630	NEK7	1	0.02348349	0.32807329	0.36950938	0.511539535
cg24429037	NEU4;NEI	2	0.00228672	0.44604817	0.506503322	0.636268872
cg10504150	NEU4;NEI	2	0.00123203	0.50692593	0.581744535	0.686943876
cg18095041	NEU4;NEI	2	0.00081413	0.33592161	0.372258028	0.542157004
cg16660312	NEU4;NEI	2	0.00081925	0.26872332	0.347630729	0.492636612
cg11706790	NEU4;NEI	2	0.0011571	0.37709327	0.466335244	0.640009499
cg20791593	NEU4;NEI	2	0.00312811	0.22126685	0.242557347	0.413007704
cg02358362	NEURL1B	5	0.00625772	0.19016264	0.154941521	0.291656835
cg01691332	NEURL1B	5	0.00201346	0.23129162	0.203447164	0.414469921
cg17505852	NF2;NF2;	22	0.0212437	0.22108776	0.162277135	0.334628315
cg13098379	NFASC;NI	1	0.00283173	0.47608362	0.508462609	0.671867209
cg27426044	NFASC;NI	1	0.00160583	0.31386235	0.351870948	0.467783669
cg04110750	NFAT5;NI	16	0.00322924	0.25645164	0.286338334	0.523741124
cg23240477	NFATC1;I	18	0.00131453	0.18152323	0.198998476	0.368276221
cg16536399	NFATC1;I	18	0.00087274	0.2725976	0.338837487	0.482775402
cg22532194	NFATC1;I	18	0.00993335	0.49634073	0.495859792	0.617394565
cg11321921	NFATC1;I	18	0.03947516	0.60034927	0.518524555	0.729310247
cg05718035	NFATC1;I	18	0.00107365	0.29599856	0.333239096	0.485648889
cg14715383	NFATC1;I	18	0.00184894	0.30227776	0.338128486	0.555210318
cg15517343	NFATC1;I	18	0.00177192	0.41848687	0.444757032	0.601818219
cg18590092	NFATC1;I	18	0.00332266	0.26515818	0.269361963	0.413158139

cg05859578	NFATC1;N	18	0.00540896	0.25345265	0.260941619	0.422814276
cg27624471	NFATC1;N	18	0.01083301	0.33129405	0.370217153	0.472643857
cg16182267	NFATC1;N	18	0.00126235	0.38413659	0.392329572	0.494409814
cg13731636	NFATC1;N	18	0.00403328	0.43539176	0.43884785	0.624323817
cg15784814	NFATC1;N	18	0.04508458	0.55309599	0.58641876	0.719755186
cg15908877	NFATC1;N	18	0.00202314	0.57769775	0.580927474	0.695144712
cg05264214	NFATC1;N	18	0.00233544	0.25419243	0.245280442	0.469604366
cg07342752	NFATC1;N	18	0.00073265	0.39801286	0.477499809	0.699279977
cg10357989	NFATC1;N	18	0.00228672	0.38218992	0.393442704	0.525316095
cg25546405	NFATC1;N	18	0.00400629	0.15232604	0.145509396	0.316251142
cg27019645	NFATC1;N	18	0.00629918	0.25534754	0.188581451	0.368641091
cg15532942	NFATC1;N	18	0.00152273	0.14979256	0.15533231	0.27601178
cg09773897	NFIC	19	0.00167443	0.38613031	0.424285431	0.569236936
cg04340258	NFIC;NFIC	19	0.00088355	0.2297972	0.25371862	0.360783208
cg15658793	NFIC;NFIC	19	0.00123203	0.16228763	0.172748873	0.312835834
cg22315974	NFIC;NFIC	19	0.00547327	0.14278119	0.127421715	0.24338189
cg16588649	NFIC;NFIC	19	0.00109472	0.37878491	0.375071202	0.557567599
cg22824635	NFIX	19	0.00405051	0.31972011	0.330207277	0.55610512
cg09394128	NFIX	19	0.00402744	0.39699154	0.393632522	0.512068389
cg12522311	NFIX	19	0.01016149	0.16820396	0.156058307	0.269395044
cg15783941	NFIX	19	0.00227382	0.22703836	0.235919374	0.386947482
cg06623668	NFIX	19	0.008246	0.43586427	0.456487179	0.615930988
cg13649253	NFIX	19	0.00203482	0.26082772	0.307965376	0.474980428
cg12499092	NFYB	12	0.00197576	0.69823732	0.668373222	0.824641671
cg12421282	NFYC;NFY	1	0.02157743	0.3727106	0.358802146	0.537369439
cg11135080	NFYC;NFY	1	0.00293735	0.40830246	0.455218782	0.598168614
cg17048169	NGEF;NG	2	0.00165917	0.18998134	0.190163413	0.407589288
cg26178217	NGEF;NG	2	0.00099274	0.57378366	0.616706651	0.722576615
cg20388707	NGEF;NG	2	0.00093925	0.31178778	0.327186199	0.441744257
cg24367967	NGEF;NG	2	0.00627871	0.41558074	0.432306405	0.534069491
cg20539430	NGEF;NG	2	0.00521564	0.56454141	0.599237976	0.708600411
cg26152051	NHEJ1	2	0.00112679	0.4842212	0.561405418	0.728752803
cg08106288	NHEJ1	2	0.02155924	0.13989346	0.114204073	0.242876396
cg24324628	NHSL1	6	0.03038061	0.26950495	0.218028594	0.377313293
cg03379797	NHSL1	6	0.00499408	0.20220557	0.150259693	0.325016557
cg21730858	NHSL1;NH	6	0.0138324	0.32762437	0.361807095	0.478682148
cg16449219	NIN;NIN;N	14	0.03124896	0.1543222	0.144459254	0.289661597
cg04155718	NISCH	3	0.0051141	0.69079209	0.672744725	0.811054388
cg16750777	NKD1	16	0.00218799	0.27207964	0.288844647	0.40195762
cg04685962	NKD1	16	0.00666111	0.54668334	0.577859034	0.682583789
cg01827248	NKD1	16	0.0023323	0.29282047	0.297798053	0.488627772
cg02015823	NKD1	16	0.02717186	0.27740338	0.27346458	0.403294522
cg05779675	NKD1	16	0.00369903	0.34873387	0.338673558	0.455842217
cg09624807	NLRC5	16	0.00073118	0.34167032	0.38816907	0.616972979
cg07121721	NLRP1	17	0.00152405	0.30722855	0.199882436	0.450734859
cg19972788	NLRP1;LC	17	0.004607	0.39858331	0.415687842	0.558904733

cg06877848	NME2;NN	17	0.03160477	0.58989092	0.596594007	0.714989205
cg27233027	NME7;NN	1	0.02680775	0.55324682	0.503948721	0.678024672
cg02016985	NMRAL1	16	0.00218837	0.51654969	0.520993444	0.644672683
cg02268561	NMT2	10	0.02846049	0.3042408	0.341525625	0.446225883
cg20299802	NOS1AP;I	1	0.01284723	0.3014978	0.309845392	0.439485223
cg06301529	NOS2;NO	17	0.02794807	0.18065145	0.136711059	0.288794541
cg21292909	NOSIP	19	0.01580869	0.5198696	0.516358095	0.665238162
cg04355077	NOSIP	19	0.01767742	0.69004414	0.685450699	0.792190073
cg26274166	NOSIP	19	0.00904361	0.38278493	0.376668397	0.53526432
cg03348937	NOTCH1	9	0.04957184	0.4159798	0.479304815	0.58115383
cg13613245	NOTCH1	9	0.01960841	0.42147189	0.51194757	0.617259955
cg14528979	NOTCH1	9	0.00391578	0.28669548	0.386287543	0.594191042
cg14205001	NOTCH1	9	0.00073118	0.57865772	0.612967607	0.776764441
cg05019807	NOTCH1	9	0.00146869	0.67747588	0.707769617	0.831740399
cg14494733	NOTCH1	9	0.00083771	0.31739747	0.341165859	0.687331683
cg26779406	NOTCH1	9	0.00132756	0.34564157	0.361995708	0.478054275
cg21234123	NOTCH1	9	0.00251187	0.66619118	0.681182524	0.790001411
cg08828723	NOTCH4	6	0.01670995	0.26303903	0.253482059	0.384112684
cg08801479	NOTCH4	6	0.02748288	0.1837936	0.163868734	0.304552164
cg07012823	NOX3	6	0.02902686	0.13358266	0.142465253	0.246155882
cg21765730	NOX3	6	0.00211366	0.137505	0.138515527	0.324227773
cg02156899	NOXO1;N	16	0.00693622	0.26248164	0.246866448	0.375679028
cg26047938	NPAS2	2	0.01891633	0.24763485	0.22992615	0.401382052
cg10147348	NPAS2	2	0.00971128	0.60320944	0.584039058	0.703728408
cg02094363	NPAS3;NI	14	0.00670577	0.35603697	0.356538526	0.509923347
cg13496915	NPAS3;NI	14	0.00518902	0.24122739	0.224745904	0.46196791
cg13019512	NPM1;NF	5	0.00806542	0.36622613	0.362893556	0.50049032
cg04865220	NPTN;NP	15	0.00265516	0.18450219	0.199475228	0.354293129
cg19996221	NPTN;NP	15	0.02075286	0.35629953	0.38524774	0.503558957
cg22082015	NR1D1	17	0.00103895	0.34665124	0.441022415	0.554328785
cg24975211	NR1D1;TI	17	0.00414415	0.14919473	0.136984719	0.261420272
cg18713088	NR4A1;NI	12	0.0051141	0.53900287	0.534529763	0.673851972
cg26933107	NR4A1;NI	12	0.00252984	0.43451422	0.399150984	0.546808647
cg17841545	NRAP;NR	10	0.00473034	0.37064741	0.469638517	0.578097754
cg13216112	NRBF2	10	0.00394226	0.21914812	0.246236214	0.39225124
cg01540911	NRF1;NRI	7	0.04794324	0.44598898	0.435057803	0.581882593
cg20712980	NRF1;NRI	7	0.00219137	0.25303373	0.314755083	0.419456288
cg02359746	NRG1;NR	8	0.01543114	0.22293089	0.244853513	0.362744705
cg24724630	NRG2;NR	5	0.00375959	0.35772413	0.315832692	0.460063984
cg01068601	NRN1L	16	0.00754388	0.30235845	0.262197	0.438616552
cg21660452	NRXN2;N	11	0.0057439	0.42452118	0.504053248	0.647532206
cg18121224	NSD1;NSI	5	0.0103127	0.17161393	0.149667044	0.301186682
cg14950344	NSF	17	0.03999097	0.21619868	0.224972735	0.327393716
cg12034199	NSUN5B;	7	0.00181621	0.48867782	0.480793677	0.589557843
cg02344784	NT5DC3	12	0.00556197	0.27574918	0.277522288	0.442633836
cg02042623	NT5DC3	12	0.01011462	0.17216191	0.143346251	0.294744012

cg09989847	NT5E	6	0.03173228	0.25982731	0.271719669	0.375228487
cg12620806	NTF3;NTF	12	0.00570746	0.42014549	0.484487141	0.645518619
cg11647944	NTF3;NTF	12	0.01144141	0.23181436	0.213029144	0.362380039
cg23405457	NTM	11	0.00257729	0.55919418	0.56719193	0.715124348
cg11754374	NTM	11	0.00126059	0.75257097	0.756111208	0.883470835
cg18377376	NTM	11	0.00198688	0.49299412	0.47853404	0.678106908
cg20827960	NTM;NTM	11	0.00619019	0.66612854	0.604841086	0.773961864
cg16996632	NTM;NTM	11	0.00753069	0.5529928	0.507776888	0.663942178
cg07114544	NTN1	17	0.00354864	0.18853884	0.184765088	0.385258007
cg19441674	NTN1	17	0.00704609	0.42216375	0.403714123	0.531564023
cg14447193	NTRK2;NT	9	0.00337598	0.16428772	0.153094349	0.292508151
cg02926307	NUAK1	12	0.00138618	0.19221924	0.221714984	0.394744686
cg07508429	NUDCD3	7	0.00971256	0.36766859	0.332923929	0.523966027
cg24829430	NUDCD3	7	0.00323448	0.58850799	0.59804901	0.714577172
cg16251232	NUDCD3	7	0.00675316	0.49896891	0.460504464	0.629130201
cg02151120	NUMA1	11	0.00513872	0.24799222	0.236350856	0.357788862
cg07999415	NUMA1	11	0.02010471	0.30243878	0.280642802	0.41708966
cg14705010	NUMA1	11	0.00515613	0.26658727	0.213574167	0.423142278
cg20887442	NUMA1	11	0.00157286	0.20851127	0.196462402	0.388312307
cg11196788	NUMA1	11	0.00476313	0.30586084	0.281832681	0.414392668
cg03352427	NUP210L	1	0.01336583	0.33938533	0.30073822	0.459020489
cg10093067	NUP98;N	11	0.04228846	0.41100372	0.369763811	0.547643205
cg21035374	NWD1;NV	19	0.01486263	0.44588499	0.423523427	0.556350991
cg24101979	NXN	17	0.00295889	0.34860914	0.39887749	0.530930281
cg23768572	NXN	17	0.00467797	0.14373554	0.143878487	0.254421623
cg02273477	NXN	17	0.02075001	0.40930766	0.452225193	0.574947733
cg19669385	NXN	17	0.01146765	0.2906041	0.338275179	0.495057283
cg20633370	NXN	17	0.00194981	0.4383108	0.506878731	0.650694732
cg14136893	NXN	17	0.01450286	0.27746772	0.263441847	0.382103888
cg05794117	OBSCN;O	1	0.00705117	0.25942405	0.272075325	0.393018647
cg23644589	OBSCN;O	1	0.00936695	0.75458573	0.686766052	0.872332843
cg18494421	OBSCN;O	1	0.00295623	0.30599335	0.26792818	0.420252048
cg05521175	OBSL1	2	0.02389088	0.26653114	0.265284314	0.416193957
cg14037346	ODF2	9	0.00426883	0.19926876	0.16638848	0.321101021
cg06608378	ODF3L1	15	0.00228672	0.57409947	0.585020527	0.70489127
cg11097249	ODZ2	5	0.01093065	0.28661412	0.294015027	0.479818762
cg00192966	ODZ2	5	0.03095964	0.26950965	0.233015511	0.373044314
cg12025243	ODZ4	11	0.00160583	0.43883547	0.415174044	0.565955877
cg16013670	OPN3	1	0.00585587	0.4343737	0.445851997	0.580304428
cg09970593	OPRD1	1	0.00311192	0.303085	0.277790533	0.457348839
cg22539670	OSBPL10	3	0.01708682	0.44423985	0.420459196	0.595934336
cg09641663	OSBPL10	3	0.0156698	0.30578543	0.29936465	0.465047194
cg26949612	OSBPL6	2	0.01880235	0.51989561	0.541918051	0.658809815
cg03739877	P11;P11	12	0.00089487	0.48319449	0.469877192	0.604020951
cg10287137	P2RY2;P2	11	0.00113577	0.2032046	0.198302723	0.305530857
cg08695113	P4HA1;P4	10	0.01616965	0.16089872	0.129218582	0.317744953

cg17054691	P4HB	17	0.0022985	0.30144042	0.297240717	0.408295659
cg14492337	P4HB	17	0.00172495	0.14310103	0.146328797	0.261527299
cg20869369	PABPC1L	20	0.00556434	0.41832005	0.385536746	0.552259486
cg08999742	PABPN1L	16	0.00561885	0.43072841	0.466324596	0.568844366
cg09428340	PACRG;P/	6	0.00230768	0.35444061	0.395269969	0.531079125
cg26344026	PACS1	11	0.00173353	0.43156995	0.415595001	0.620957335
cg16162970	PACS2;PA	14	0.00144385	0.15577282	0.176089821	0.278234951
cg16783764	PACS2;PA	14	0.00094183	0.61440098	0.618108704	0.726985204
cg18374755	PACS2;PA	14	0.00494219	0.48238475	0.45785818	0.598694729
cg14063129	PACS2;PA	14	0.00174721	0.11563139	0.126117569	0.250176549
cg01052428	PACS2;PA	14	0.00090315	0.22229624	0.243439628	0.348156621
cg21170836	PACS2;PA	14	0.00198593	0.47261233	0.483932171	0.59812483
cg07521618	PACS2;PA	14	0.00092387	0.2770181	0.30408509	0.483573596
cg15483436	PACS2;PA	14	0.00153162	0.50542274	0.513020304	0.627355474
cg12158535	PACS2;PA	14	0.00146869	0.21565089	0.226998245	0.339033338
cg12397564	PAFAH2	1	0.00822746	0.16044948	0.128631281	0.30888424
cg17263916	PAIP2;PA	5	0.00591073	0.47778669	0.48532271	0.625315503
cg14069287	PALLD;PA	4	0.00689691	0.22711149	0.221343563	0.339973879
cg21204870	PALM2;P/	9	0.00277534	0.53524004	0.585295806	0.75092361
cg17407629	PAM;PAN	5	0.01398104	0.35240442	0.356858878	0.508260663
cg22911687	PAM;PAN	5	0.00109306	0.64821996	0.561283408	0.807392938
cg20655220	PAM;PAN	5	0.03793002	0.48209643	0.443276605	0.628058299
cg12617066	PANK2;M	20	0.02277323	0.25405066	0.279395659	0.400681095
cg15007472	PANX1	11	0.01674385	0.52804638	0.539424694	0.661093373
cg16805189	PANX1	11	0.0033331	0.52656117	0.567620374	0.678520303
cg21894762	PAPLN	14	0.00232775	0.31180326	0.313105965	0.503568424
cg17691988	PAPSS1	4	0.02041679	0.27817674	0.23621349	0.387622547
cg26541780	PAQR6;P/	1	0.01820811	0.25910187	0.227511773	0.360967749
cg14150803	PARK7;PA	1	0.00575746	0.24035348	0.239194261	0.349598866
cg00734683	PARP3;PA	3	0.00087864	0.64175256	0.673506197	0.784980604
cg13518121	PARVA	11	0.00911882	0.24959089	0.22028023	0.483281147
cg16634971	PARVA	11	0.00758031	0.26185921	0.249650595	0.452068906
cg08647724	PARVA	11	0.00804549	0.18597131	0.179048767	0.29411986
cg15123662	PARVA	11	0.00155548	0.30159541	0.281082177	0.427451911
cg13959831	PAX5	9	0.02562377	0.18043297	0.154143539	0.29515627
cg14293548	PAX6;PA>	11	0.01783758	0.59982208	0.594067927	0.707110952
cg05923681	PBRM1;P	3	0.00195549	0.54166513	0.593895059	0.720924371
cg15937861	PC;PC;PC	11	0.00263648	0.27256935	0.262043118	0.471811574
cg15784784	PC;PC;PC	11	0.00205127	0.60823074	0.600196498	0.719767676
cg06467504	PC;PC;PC	11	0.01678972	0.28021398	0.279304009	0.394207927
cg23022313	PC;PC;PC	11	0.00937895	0.1983714	0.197770058	0.311829148
cg11194398	PC;PC;PC	11	0.01159878	0.1990814	0.204734232	0.36927043
cg06022884	PCCB	3	0.0026628	0.49915902	0.526062369	0.680084779
cg00160440	PCDH10	4	0.0008947	0.24091029	0.343208832	0.544199432
cg18869485	PCDHGA4	5	0.00873119	0.50900068	0.568989256	0.677508222
cg03415916	PCDHGA4	5	0.02957954	0.50422089	0.537742791	0.650780837

cg16056164	PCNXL2	1	0.00382061	0.1386362	0.142469327	0.278291727
cg04105230	PCSK6;PC	15	0.03157474	0.62188426	0.631757078	0.755256528
cg19316405	PDDC1	11	0.00197403	0.25541398	0.25188094	0.48479607
cg10856894	PDE10A;F	6	0.03468727	0.37745569	0.27234599	0.497078277
cg05034374	PDE11A;F	2	0.00521564	0.24886823	0.226812312	0.475701284
cg05691871	PDE4D;PI	5	0.00376781	0.69325603	0.705143897	0.82344057
cg25317315	PDE4DIP;	1	0.00202182	0.27235754	0.298621544	0.452124486
cg10872815	PDE7B	6	0.00725225	0.29415901	0.269895635	0.440175775
cg22835523	PDE8A;PI	15	0.00276855	0.62055539	0.62789854	0.728067873
cg08835985	PDF;COG	16	0.0018345	0.30154531	0.241104012	0.476041143
cg23496331	PDGFRB	5	0.01113996	0.37005203	0.334217972	0.482222402
cg07576186	PDHB	3	0.00483111	0.33898761	0.448938306	0.591231008
cg10093934	PDK1	2	0.00384818	0.34111576	0.398761103	0.570627536
cg20714347	PDLIM1	10	0.00353204	0.31195608	0.353241031	0.508377109
cg07262247	PDLIM4;F	5	0.01823439	0.28161111	0.252372408	0.381886599
cg23976221	PDLIM5;F	4	0.00408296	0.22750042	0.185327708	0.355679732
cg27650985	PDLIM7;F	5	0.00653182	0.50664004	0.497711577	0.614175693
cg17037976	PDLIM7;F	5	0.00911882	0.29395912	0.283426211	0.399165038
cg09972454	PDXDC1	16	0.00661882	0.35176714	0.349054863	0.481296778
cg14906976	PDXK	21	0.00243695	0.215175	0.176241541	0.368404196
cg11565786	PDZD2	5	0.00169556	0.63390002	0.685811593	0.797725214
cg18410110	PDZD2	5	0.00233284	0.20465168	0.226178303	0.35773222
cg02529095	PDZD7	10	0.00658735	0.43925975	0.446272215	0.590977703
cg05593775	PDZD7	10	0.00381594	0.14822021	0.147950341	0.26815728
cg09437460	PDZD8	10	0.02895902	0.35441716	0.39065424	0.529117706
cg13019092	PDZK1	1	0.00130383	0.27498037	0.324121773	0.501182343
cg10321723	PDZK1	1	0.00318268	0.20399091	0.218758958	0.31936744
cg07810156	PDZK1IP1	1	0.00307424	0.27373598	0.233808114	0.420160309
cg25688523	PDZRN3	3	0.00142706	0.3049934	0.360115165	0.514184732
cg13050115	PDZRN3	3	0.002727	0.411668	0.44617683	0.680758373
cg12584826	PDZRN3	3	0.00401281	0.36962821	0.411500958	0.545962957
cg01826056	PDZRN4;F	12	0.0070644	0.51212936	0.509113617	0.612290019
cg15534461	PEBP1	12	0.00499723	0.41353266	0.39731249	0.548054453
cg13521002	PEBP4	8	0.00134844	0.37451433	0.428421394	0.59606439
cg21932360	PEBP4	8	0.00958999	0.49319333	0.505065549	0.610937192
cg18050520	PEBP4	8	0.02494466	0.2117841	0.156805795	0.315337768
cg25392325	PEMT;PEI	17	0.00279729	0.2679462	0.261616004	0.456660865
cg26177311	PEMT;PEI	17	0.00414415	0.12575103	0.132108654	0.26215135
cg12308675	PER2	2	0.00154265	0.46594959	0.440624328	0.694211365
cg22879834	PER2	2	0.00291825	0.37632687	0.38331856	0.567581488
cg07719617	PER2	2	0.00308097	0.31100487	0.318630315	0.484287343
cg25514503	PER3	1	0.02878476	0.31059517	0.316045463	0.435110485
cg09327610	PER3	1	0.00401796	0.29128929	0.366731184	0.557062773
cg25821223	PERP	6	0.0011159	0.35801045	0.466091102	0.600662571
cg24524895	PEX11A	15	0.01966537	0.33270703	0.296202893	0.486509555
cg08745329	PFKFB3	10	0.0042395	0.31041389	0.305146129	0.462151724

cg08500510	PFKFB3	10	0.00197986	0.16419783	0.146413568	0.418310149
cg03124510	PFKFB3	10	0.00216509	0.19630667	0.170171772	0.437060448
cg08289056	PFKFB3	10	0.00076471	0.43397789	0.571524984	0.685712307
cg13782176	PFKFB3;P	10	0.00129758	0.47608834	0.491146141	0.654833203
cg19084628	PFKFB3;P	10	0.00219711	0.2942995	0.31680953	0.496312468
cg07102808	PFKFB3;P	10	0.0011104	0.22843727	0.2650601	0.439311846
cg00373371	PFKFB3;P	10	0.00303542	0.42936273	0.445897803	0.551233471
cg08568670	PFKFB4	3	0.00756169	0.39451735	0.479667431	0.624391731
cg09274658	PFKL;PFKI	21	0.00141699	0.23002701	0.250918305	0.363901937
cg04643920	PFKL;PFKI	21	0.00271218	0.22336071	0.240893012	0.354909292
cg16324184	PFKP	10	0.00514	0.64816976	0.628990798	0.748326651
cg12265908	PFKP	10	0.00855749	0.31087303	0.308630813	0.463500558
cg16963373	PFKP	10	0.01237003	0.38088193	0.374820063	0.487826166
cg12303582	PFKP	10	0.00403008	0.21725749	0.222279717	0.355163734
cg01287088	PFN3	5	0.01236318	0.2763359	0.249480364	0.383663286
cg05888583	PGBD5	1	0.00422785	0.31832786	0.270630172	0.42632247
cg27224809	PGBD5	1	0.00328556	0.38949409	0.334499753	0.536282911
cg04026948	PGCP	8	0.00539963	0.18340458	0.228525939	0.422991354
cg22624827	PGM1	1	0.02499377	0.28013945	0.329434387	0.454870732
cg04839673	PGM1	1	0.00099274	0.40945676	0.483349122	0.67379782
cg03891302	PHACTR1	6	0.00253062	0.57778173	0.621260487	0.778637328
cg17586302	PHACTR2	6	0.00279494	0.13313328	0.135275744	0.240655197
cg08482167	PHACTR2	6	0.00436875	0.22145938	0.233538192	0.46499451
cg06953325	PHC2	1	0.01964774	0.54359153	0.547240689	0.649275247
cg24691891	PHC2	1	0.00556197	0.19243322	0.189076637	0.36055468
cg00582531	PHF15	5	0.00279997	0.57827886	0.58596408	0.702342991
cg13471712	PHF21A;F	11	0.00078196	0.44391158	0.495755753	0.601918286
cg16220788	PHF21A;F	11	0.00494232	0.19034045	0.227225799	0.425082452
cg06763568	PHGDH	1	0.00457305	0.33471691	0.321997836	0.470263699
cg19818271	PHTF2;PH	7	0.01179846	0.17063933	0.200648708	0.30609934
cg05036615	PHTF2;PH	7	0.02944061	0.30987862	0.298990945	0.425909564
cg13613439	PHYHD1;I	9	0.01317021	0.39682716	0.392304458	0.554915135
cg12083852	PI4KB	1	0.00198593	0.31700827	0.330891147	0.467773513
cg07926644	PIAS4	19	0.00854289	0.35263707	0.311137846	0.488753523
cg24200175	PICALM;F	11	0.02496653	0.25355896	0.231520728	0.414655562
cg16760049	PICK1;PIC	22	0.00539451	0.51088129	0.530721404	0.64600851
cg24859236	PIK3CD	1	0.00209789	0.1865202	0.182068953	0.322623543
cg17725019	PIK3IP1;P	22	0.00263125	0.25866266	0.257314234	0.486167813
cg01394255	PIK3R5;PI	17	0.00344865	0.53253156	0.517188647	0.655960468
cg17609282	PION	7	0.01174492	0.7254037	0.713230822	0.841508818
cg19397801	PIP4K2A	10	0.0039383	0.3072699	0.423782936	0.635067629
cg11327029	PIP4K2A	10	0.00104452	0.31845253	0.411117566	0.689157304
cg03771739	PIP4K2A	10	0.00065949	0.31568576	0.549544671	0.666393312
cg13995193	PIP5K1C	19	0.00167255	0.40719291	0.421034374	0.566402969
cg19841005	PIP5K1C	19	0.00116286	0.17341787	0.195167435	0.304455898
cg12148647	PIP5K1C	19	0.00159949	0.16499905	0.222843799	0.326743826

cg04965671	PISD	22	0.00878866	0.31715319	0.29456732	0.507672549
cg07284476	PITPNC1;	17	0.00312811	0.63478825	0.683704098	0.793597652
cg13037550	PITPNM2	12	0.00510852	0.56070016	0.596481425	0.730141897
cg05399718	PITPNM2	12	0.00107365	0.2294389	0.233951393	0.437858512
cg24389239	PITPNM3	17	0.0050337	0.63537686	0.638069103	0.739546836
cg12020444	PIWIL1	12	0.01163705	0.55977986	0.552121021	0.665597529
cg05598886	PKD1;MIF	16	0.00334886	0.66584018	0.619236882	0.777053275
cg19949931	PKD1;MIF	16	0.01529615	0.42536629	0.410424817	0.575208339
cg07568117	PKD1;PKD	16	0.00152023	0.65789048	0.662027642	0.799895514
cg27227497	PKD1;PKD	16	0.00663932	0.22965886	0.225130511	0.402850473
cg03494430	PKD1;PKD	16	0.00439624	0.2446409	0.224595102	0.402178719
cg10731848	PKD1L1	7	0.0048909	0.21386324	0.198536174	0.439008455
cg27618305	PKD1L1	7	0.00883809	0.17293915	0.144952707	0.275760363
cg02735620	PKD2	4	0.00422345	0.50014615	0.467195093	0.680091015
cg12078253	PKDCC	2	0.03749859	0.30381156	0.292356844	0.438229257
cg07565018	PKM2;PKI	15	0.00667367	0.22774041	0.17158805	0.393862835
cg02742281	PKMYT1;I	16	0.00200001	0.34616179	0.288075241	0.516250389
cg01243773	PKNOX2	11	0.00855749	0.223106	0.193431427	0.381699865
cg15727708	PKNOX2	11	0.00152273	0.54528092	0.53681024	0.666030394
cg13168187	PKP4;PKP	2	0.00092985	0.31689471	0.395685867	0.618668568
cg13211559	PLA2G2A;	1	0.00218837	0.40829731	0.3500685	0.524550903
cg02203881	PLA2G4D	15	0.02151984	0.3179537	0.318725066	0.424111928
cg21776667	PLAGL1;P	6	0.03502867	0.25393475	0.289131118	0.391239562
cg23120601	PLCB2	15	0.00262867	0.25685909	0.217044086	0.383657656
cg09233429	PLCB2	15	0.00352383	0.29113978	0.243194734	0.393099804
cg20484832	PLCB4;PL(	20	0.00546612	0.74512567	0.754557303	0.85808337
cg13506485	PLCE1	10	0.03384214	0.18926019	0.180072129	0.298613377
cg27638309	PLCE1;PL(	10	0.03817436	0.3586983	0.256839845	0.4612736
cg00940248	PLCG2	16	0.04459242	0.3004198	0.252689195	0.416584092
cg21767191	PLCL2;PL(	3	0.00714809	0.69867485	0.675681297	0.801195145
cg02681842	PLEC1;PLI	8	0.00896808	0.17916161	0.161418998	0.307432251
cg17082405	PLEC1;PLI	8	0.00219764	0.12828809	0.107241766	0.27504109
cg20680163	PLEC1;PLI	8	0.01329117	0.17463804	0.13829554	0.286742881
cg05141333	PLEC1;PLI	8	0.00204964	0.47587437	0.500845831	0.605922104
cg07598407	PLEC1;PLI	8	0.00574743	0.30687035	0.302054703	0.429847256
cg03537810	PLEC1;PLI	8	0.00252124	0.37113607	0.35973539	0.478405535
cg07132086	PLEKHA2	8	0.03352901	0.26792644	0.252716884	0.421187179
cg19755459	PLEKHA5;	12	0.01751915	0.31584021	0.342242348	0.461258614
cg11131599	PLEKHA6	1	0.00081925	0.41017906	0.543973333	0.656519764
cg15306794	PLEKHA7	11	0.01195883	0.36340497	0.387714877	0.559520805
cg22309568	PLEKHF1	19	0.02595684	0.3104935	0.272173199	0.424298708
cg08445687	PLEKHF1	19	0.00076471	0.15823672	0.181954744	0.360122004
cg12063688	PLEKHG3	14	0.00218597	0.15966075	0.157151528	0.305834483
cg07942500	PLEKHG3	14	0.00170239	0.15934798	0.161074867	0.299126107
cg11802553	PLEKHG3	14	0.00207771	0.38589228	0.401196975	0.560121296
cg06426253	PLEKHG4;	16	0.00147208	0.63637366	0.650201652	0.772648486

cg23462129	PLEKHG4I	5	0.01206219	0.57367696	0.600161738	0.703377547
cg02815014	PLEKHM3	2	0.0012785	0.60210687	0.574943944	0.752165013
cg26232945	PLEKHM3	2	0.00602378	0.19997147	0.199062062	0.397541064
cg18778727	PLSCR1	3	0.02389893	0.3798257	0.443208312	0.548441267
cg18686270	PLSCR1	3	0.02089413	0.67588044	0.668926508	0.777860169
cg03631457	PLXDC2	10	0.00169556	0.43996504	0.491903459	0.654060119
cg12723985	PLXDC2	10	0.00802316	0.40589773	0.417531912	0.570530693
cg11546777	PLXDC2	10	0.00365165	0.33107413	0.435060059	0.572083115
cg24161018	PLXNA4	7	0.0008696	0.41034353	0.4732683	0.678617292
cg11602148	PLXNB1;P	3	0.00157567	0.47005319	0.485333	0.718416488
cg16357930	PLXNB1;P	3	0.00165724	0.35892793	0.362771234	0.52992137
cg09837648	PLXNB1;P	3	0.00068904	0.25299715	0.345595089	0.528274906
cg10698910	PLXNB2	22	0.00667841	0.39791394	0.395628888	0.506304751
cg27206976	PLXNB2	22	0.00119385	0.26974409	0.312784895	0.441530627
cg12349580	PLXNC1	12	0.00262388	0.39495637	0.401570617	0.563781651
cg07078269	PLXND1	3	0.00092849	0.36251665	0.429292206	0.640906836
cg27353085	PLXND1	3	0.00248169	0.26668655	0.305425649	0.507995167
cg05490233	PLXND1	3	0.00319723	0.41284198	0.424450437	0.554708843
cg07908223	PMEPA1;	20	0.00214604	0.31898343	0.359490519	0.563801308
cg05355067	PMEPA1;	20	0.00186474	0.21083678	0.200422197	0.367920957
cg00177431	PMEPA1;	20	0.02152265	0.19173718	0.192245742	0.344068144
cg11858564	PNMA1;C	14	0.03514972	0.65483702	0.65110734	0.773903665
cg27178772	PNRC1	6	0.00213077	0.33826783	0.361167848	0.525919548
cg14522034	POFUT2;F	21	0.00384078	0.18686557	0.165085771	0.288149678
cg23694811	POGK	1	0.00935896	0.37553578	0.411571093	0.521324273
cg01444413	POGZ;PO	1	0.01221904	0.56340531	0.576868145	0.711136176
cg07274204	POL3S	16	0.00243678	0.44577186	0.356723983	0.55781987
cg27197524	POLE	12	0.00141191	0.59537273	0.573109459	0.791924877
cg20361429	POLE	12	0.00103895	0.58010467	0.577145015	0.788364809
cg24940701	POLR2E	19	0.00147208	0.69702939	0.705912752	0.826903448
cg13859108	POM121C	7	0.00425007	0.29207384	0.238163917	0.451867694
cg04089800	POMGNT	1	0.02681286	0.16495654	0.140397363	0.269454438
cg04270402	PON2;PO	7	0.01698967	0.4288896	0.444595681	0.551467248
cg18397073	POU2F2	19	0.01479868	0.23669632	0.19363645	0.356100917
cg11682697	PPAP2B;F	1	0.00140062	0.09190934	0.099673994	0.204540578
cg15896446	PPAPDC1	10	0.00804549	0.4587011	0.454502863	0.591512439
cg13436417	PPAPDC1	10	0.00556197	0.26882197	0.26895388	0.378386249
cg21286173	PPAPDC1	8	0.00219764	0.21590282	0.235057563	0.45774463
cg22185879	PPDPF	20	0.00317466	0.22719266	0.227818805	0.360176448
cg12652780	PPFIA1;PI	11	0.00337131	0.25788714	0.254866696	0.396784828
cg10952234	PPFIA4;PI	1	0.00091059	0.3562958	0.405181238	0.541010339
cg24000087	PPFIA4;PI	1	0.00142706	0.30367631	0.341204831	0.488771923
cg04375036	PPP1CC	12	0.00336646	0.29010803	0.286511935	0.435310041
cg03596877	PPP1R10	6	0.01110735	0.39541063	0.422234512	0.526719462
cg26058884	PPP1R10	6	0.00921039	0.2609847	0.195670436	0.382092016
cg08067969	PPP1R13I	14	0.00398122	0.35813309	0.343913599	0.552368814

cg06398396	PPP1R16/	8	0.00075458	0.31573184	0.349764972	0.456351339
cg18354248	PPP1R3C	10	0.02735855	0.16887801	0.149007129	0.289598932
cg24288527	PPP1R3C	10	0.00579556	0.20827181	0.193432928	0.327713156
cg25409308	PPP2R1B	11	0.00295256	0.47306315	0.526506624	0.642384014
cg03723716	PPP2R1B;	11	0.04180327	0.18702022	0.166455899	0.304016729
cg14823825	PPP2R2A	8	0.03223944	0.43203244	0.470999725	0.585050597
cg02265810	PPP2R2D.	10	0.00206218	0.44989177	0.466309723	0.707712407
cg15444217	PPP2R2D.	10	0.01561358	0.59774031	0.630647351	0.738774817
cg15102070	PPP2R2D.	10	0.00094183	0.53809551	0.569570413	0.689566048
cg03072003	PPP2R2D.	10	0.00318584	0.24211949	0.247496757	0.385664482
cg13845634	PPY	17	0.00449495	0.50629849	0.53079741	0.637803029
cg02710481	PQLC1;PC	18	0.00317183	0.27307374	0.188890139	0.384985307
cg13448468	PRDM11	11	0.00245827	0.23283614	0.252239415	0.397384571
cg00328729	PRDM11	11	0.01907089	0.23217792	0.239771495	0.35771602
cg15619820	PRDM16;	1	0.00274286	0.24555427	0.248760022	0.431272407
cg22720392	PRDM16;	1	0.00177922	0.19662409	0.214895627	0.382281094
cg25153629	PRDM16;	1	0.00220808	0.42401515	0.425034656	0.622875977
cg22497969	PRDM16;	1	0.00130383	0.13217033	0.13533609	0.344132447
cg04855369	PRDM16;	1	0.00279686	0.26833497	0.242617888	0.416142978
cg11392550	PRDM16;	1	0.00273943	0.22272221	0.252265905	0.431395446
cg19263228	PRDM16;	1	0.00338778	0.1799868	0.191017376	0.311898095
cg17601209	PRDM16;	1	0.00550622	0.30678688	0.347511675	0.499855026
cg26520908	PRDM16;	1	0.0073123	0.22352269	0.229780556	0.341875688
cg00894041	PRDM16;	1	0.00889507	0.22062033	0.207519807	0.337676619
cg11138362	PRDM16;	1	0.00068904	0.13893301	0.213123209	0.315540995
cg14712575	PRDM16;	1	0.00154265	0.45760175	0.474631007	0.622488911
cg06911744	PRDM16;	1	0.00154265	0.2714533	0.33592814	0.516129845
cg26938014	PRDM6	5	0.00993101	0.46294232	0.494507751	0.630038558
cg19644991	PRDM6	5	0.00387834	0.43229943	0.519059169	0.660759528
cg03311556	PRDM8	4	0.00087298	0.28760765	0.390318091	0.59491434
cg01789499	PRDM8	4	0.0019386	0.16841359	0.174711258	0.295759229
cg09595050	PRDM8	4	0.00079883	0.33829421	0.379605577	0.493513814
cg06440348	PRDM8	4	0.00249039	0.32392139	0.349747107	0.507810077
cg05452645	PRDM8;P	4	0.00066232	0.21910546	0.296982233	0.434586395
cg00138041	PRDM8;P	4	0.00065949	0.20963815	0.272097207	0.414311555
cg19409579	PRDM8;P	4	0.00246645	0.11280971	0.125756515	0.243362741
cg02732915	PRDM8;P	4	0.01021212	0.4344348	0.521964113	0.675941789
cg02941135	PRDM8;P	4	0.0113009	0.50255521	0.603894373	0.715398421
cg10880006	PRDM8;P	4	0.00774063	0.18512407	0.199156643	0.336097301
cg05522011	PRDM8;P	4	0.00148902	0.34693957	0.420288285	0.570366342
cg06989253	PRDX1;PF	1	0.00425638	0.18376734	0.162556456	0.291540125
cg05889294	PRELP;PR	1	0.0113406	0.1891543	0.19338481	0.308097813
cg19752143	PREPL;PR	2	0.04791139	0.36894911	0.342176215	0.493961965
cg00555268	PRIC285	20	0.01580766	0.29425016	0.287504979	0.410823293
cg11409510	PRICKLE1	12	0.01026108	0.58777416	0.630143193	0.744475134
cg03190018	PRKAG2;F	7	0.00141191	0.3104136	0.28958745	0.537073995

cg22719878	PRKAG2;F	7	0.00107365	0.39221776	0.443136984	0.59194616
cg27577993	PRKAG2;F	7	0.0135118	0.22883494	0.191502401	0.411887185
cg17814717	PRKAG2;F	7	0.01151933	0.29538013	0.285041718	0.418821452
cg26482626	PRKAG2;F	7	0.00099274	0.37190729	0.437755584	0.573664925
cg26284782	PRKAG2;F	7	0.00073547	0.17538171	0.225424488	0.411983904
cg09397579	PRKAG2;F	7	0.00183351	0.21108391	0.223452253	0.344958793
cg11315991	PRKAR1B	7	0.00366802	0.37491434	0.418739229	0.593977227
cg06878741	PRKAR1B	7	0.00226669	0.33319304	0.341783759	0.50821507
cg18241094	PRKAR1B	7	0.00444287	0.22970653	0.228926145	0.364664237
cg04666346	PRKAR1B	7	0.01677529	0.2093989	0.189431001	0.328569983
cg21150288	PRKAR1B	7	0.00521564	0.5190511	0.534672325	0.674955802
cg04191989	PRKAR1B	7	0.01276611	0.27647838	0.242729782	0.416634895
cg06413196	PRKAR1B	7	0.01725665	0.18873383	0.160737765	0.302106178
cg03719152	PRKAR1B	7	0.01291157	0.19392599	0.172723761	0.344223533
cg01462184	PRKAR1B	7	0.0048909	0.12986675	0.132595569	0.233508947
cg10127554	PRKAR1B	7	0.00077468	0.45370671	0.520132129	0.644729953
cg16581738	PRKAR1B	7	0.00081789	0.43164228	0.506441531	0.734248361
cg27418434	PRKAR1B	7	0.0013661	0.31440448	0.318191584	0.479706094
cg01477379	PRKCA	17	0.01299988	0.21112688	0.204513691	0.356670481
cg14121185	PRKCA	17	0.00820044	0.20246892	0.245380902	0.35015529
cg19325477	PRKCA	17	0.01032661	0.64244054	0.555896822	0.761329276
cg02699518	PRKCA	17	0.00322924	0.45834938	0.448074093	0.655376849
cg24171047	PRKCA	17	0.01463726	0.61121595	0.633358494	0.74244023
cg09655520	PRKCA	17	0.00099274	0.45902831	0.471141908	0.716420846
cg22632947	PRKCA	17	0.01373528	0.38434757	0.416428209	0.523605425
cg10460643	PRKCD;PF	3	0.00073118	0.34756875	0.405972766	0.560176752
cg15035324	PRKCE	2	0.01705178	0.34124382	0.376793351	0.519388301
cg24545602	PRKCE	2	0.03502867	0.33808639	0.270710098	0.458272459
cg00409434	PRKCE	2	0.01145563	0.39913565	0.421069438	0.589380749
cg15505219	PRKCH	14	0.01309134	0.41689646	0.388100556	0.572149409
cg24447775	PRKCZ	1	0.00701147	0.42366148	0.439503601	0.542046968
cg06176608	PRKCZ;PR	1	0.01281774	0.38672253	0.374868102	0.49035992
cg00409816	PRKCZ;PR	1	0.00137043	0.5927351	0.582889724	0.760170047
cg00981070	PRKCZ;PR	1	0.01317683	0.32416111	0.322425806	0.442855657
cg09179248	PRKCZ;PR	1	0.00756169	0.24516892	0.24206268	0.387204803
cg07836663	PRKCZ;PR	1	0.00129968	0.36766224	0.362363309	0.487550276
cg00866690	PRKCZ;PR	1	0.0026668	0.32608066	0.313071442	0.468195638
cg22128985	PRKCZ;PR	1	0.00207771	0.33911093	0.362404639	0.542414036
cg24578937	PRKCZ;PR	1	0.00081599	0.11253055	0.158545526	0.258917627
cg02840585	PRKCZ;PR	1	0.00066232	0.39752583	0.482415382	0.679636204
cg17156349	PRKCZ;PR	1	0.00334886	0.26224464	0.298603203	0.474418424
cg25727019	PRKCZ;PR	1	0.00197487	0.3392053	0.384374023	0.546663122
cg22338356	PRKDC;PF	8	0.01842786	0.23146844	0.18284814	0.351879947
cg15121267	PRL;PRL	6	0.00097053	0.31582314	0.350266628	0.507657042
cg11705975	PRLHR	10	0.00329066	0.21846566	0.188153001	0.329081745
cg01456572	PRM3	16	0.00284268	0.29988504	0.320323998	0.441728512

cg08358964	PRM3	16	0.00953692	0.19856679	0.198526535	0.322439327
cg09196146	PRO1768	14	0.00911311	0.3196743	0.375414624	0.486943608
cg25734864	PROZ	13	0.01148808	0.55750442	0.536726222	0.662695634
cg23800587	PROZ	13	0.00563693	0.54873601	0.539398062	0.659742268
cg12459923	PRPF6	20	0.00175545	0.37549093	0.303068003	0.594347925
cg11167993	PRPF6	20	0.00220808	0.64924629	0.497833241	0.778975871
cg12155028	PRR16	5	0.00320159	0.48088702	0.41392421	0.584644723
cg26995244	PRR5;PRF	22	0.00227382	0.09569346	0.120131801	0.22023228
cg08123207	PRRT1	6	0.00139627	0.24134134	0.218229229	0.391513648
cg19227031	PRRT1	6	0.00368543	0.30637332	0.28192279	0.417271434
cg22849665	PRRT1	6	0.01026031	0.12982147	0.132306691	0.236693376
cg16040614	PRRT1	6	0.00458159	0.36368323	0.35100708	0.505249287
cg20266593	PRRT1	6	0.00428303	0.29355714	0.262977966	0.491955847
cg01943851	PRRT2	16	0.00297078	0.7319372	0.705855769	0.841807793
cg20094462	PRTN3	19	0.00264473	0.11055604	0.098459507	0.2131193
cg13579473	PSD3	8	0.02215556	0.43512121	0.45027069	0.606240872
cg24804106	PSD3	8	0.01616965	0.68730656	0.686601075	0.789447463
cg09894683	PSD3;PSD	8	0.01970757	0.22187403	0.237053752	0.392884082
cg03172765	PSMD1	2	0.01884035	0.55946503	0.511118549	0.704458519
cg05989984	PSMD9;W	12	0.00904235	0.5686919	0.576318076	0.742988895
cg15077193	PTBP1;PT	19	0.00997311	0.14832143	0.133077092	0.266465814
cg02086742	PTBP1;PT	19	0.00377759	0.61374906	0.621010641	0.806853005
cg17357561	PTBP1;PT	19	0.0048909	0.30522735	0.288025208	0.478570604
cg01025800	PTBP1;PT	19	0.00130383	0.48328449	0.54221961	0.687329371
cg16656493	PTCRA	6	0.01240534	0.40982162	0.385316041	0.520566646
cg14914552	PTDSS1	8	0.00240535	0.50278069	0.530883536	0.701140275
cg09267188	PTDSS1	8	0.00756169	0.32999322	0.398391345	0.499523041
cg24862548	PTDSS1	8	0.00513872	0.52865769	0.523684208	0.660657929
cg08251761	PTDSS2	11	0.00457044	0.42521552	0.400851079	0.562603494
cg15751198	PTDSS2	11	0.0156698	0.68980199	0.683444971	0.792637078
cg10575846	PTDSS2	11	0.00124243	0.44534192	0.449835409	0.651332168
cg13712952	PTENP1	9	0.01064534	0.4762364	0.524781747	0.655169999
cg12603671	PTGER3;F	1	0.01910705	0.16283783	0.133618407	0.273774016
cg01311102	PTGES2;P	9	0.03100155	0.48844515	0.481673368	0.593152693
cg04041000	PTH1R	3	0.00219711	0.45288121	0.40032123	0.632351844
cg18707417	PTH1R	3	0.00429038	0.30691498	0.306071012	0.466984752
cg00658161	PTK2B;PT	8	0.00799281	0.42632906	0.402632839	0.533880926
cg02454815	PTOV1	19	0.01365382	0.70215965	0.708620795	0.814290357
cg07268431	PTPN23	3	0.00430896	0.15045305	0.153731991	0.326039845
cg27234747	PTPN4	2	0.01237894	0.32858534	0.313776544	0.502230417
cg04025127	PTPRF;PT	1	0.00361412	0.42623243	0.395645193	0.53713789
cg13235366	PTPRF;PT	1	0.00725763	0.17539815	0.151728587	0.293171655
cg18022607	PTPRG	3	0.00384078	0.59289751	0.643779459	0.743782543
cg27118533	PTPRK;PT	6	0.00515613	0.15620813	0.126930984	0.284431261
cg21323106	PTPRN2;F	7	0.00401824	0.42950021	0.389587993	0.56880896
cg02505956	PTPRN2;F	7	0.00359753	0.39589414	0.310873713	0.533224569

cg27572053	PTPRN2;F	7	0.04054798	0.30266805	0.333703312	0.447692089
cg15085205	PTPRR	12	0.01426642	0.38733595	0.351534425	0.509979202
cg13896328	PTPRU;PT	1	0.00186737	0.20209829	0.204670107	0.358407298
cg26369180	PUSL1	1	0.00784239	0.56599236	0.554233525	0.666431759
cg25328384	PVR;PVR;	19	0.03274223	0.24169137	0.214725412	0.366965129
cg20847701	PVT1	8	0.0009934	0.11534492	0.124775328	0.316498264
cg07733247	PWWP2B	10	0.00309973	0.53611542	0.533616834	0.651940262
cg14230647	PXDN	2	0.01148808	0.54305969	0.54291169	0.649980026
cg20311673	PXDN	2	0.00306982	0.25704537	0.243673408	0.401588444
cg21514857	PXDNL	8	0.02346534	0.23823987	0.246269369	0.375494219
cg09849967	QARS	3	0.00629716	0.62735002	0.602019695	0.747376713
cg13991698	QSOX1;Q	1	0.00160732	0.567837	0.554394607	0.701696465
cg23626387	QSOX1;Q	1	0.00323646	0.17684374	0.16284118	0.306942497
cg10023454	QSOX1;Q	1	0.01373528	0.52691661	0.5625597	0.688660731
cg02363202	R3HDM2	12	0.00262147	0.38073935	0.368658877	0.500873233
cg04685747	RAB11B	19	0.0252514	0.30429489	0.303858466	0.412356292
cg09962377	RAB11FIP	16	0.00299664	0.17417553	0.171440615	0.338253442
cg09408143	RAB11FIP	16	0.0061024	0.32280835	0.379446076	0.480767643
cg05000339	RAB11FIP	17	0.00092661	0.23233434	0.271379628	0.421587582
cg25709050	RAB11FIP	17	0.00126804	0.22190902	0.271543699	0.397373952
cg24174232	RAB11FIP	17	0.00114883	0.15352986	0.184673406	0.329664783
cg01433190	RAB11FIP	2	0.0156698	0.4295882	0.434151177	0.588134112
cg26881535	RAB3GAP	1	0.02507458	0.27769462	0.23753048	0.474178851
cg06755297	RAB3GAP	1	0.00670817	0.65916575	0.661286778	0.785435358
cg03349407	RAB3GAP	1	0.00367708	0.26571347	0.292262972	0.49249542
cg18573251	RAB3IL1	11	0.00630644	0.25923978	0.222829985	0.432969032
cg03828193	RAB40B	17	0.0103161	0.37643699	0.366510226	0.504278166
cg06714655	RAB40C	16	0.00170239	0.62121985	0.646265424	0.76354759
cg07597022	RAB40C	16	0.00197786	0.49323528	0.49249184	0.666782642
cg08524190	RAB43	3	0.02628532	0.25357966	0.224585364	0.365399365
cg06459724	RAB7L1;R	1	0.00670577	0.17188148	0.153515186	0.299736898
cg10098464	RAB7L1;R	1	0.00893394	0.41691976	0.398288876	0.55427565
cg12019814	RAD21	8	0.00461206	0.61937907	0.580005258	0.736872175
cg01197302	RADIL	7	0.00703549	0.2453173	0.19491437	0.378177887
cg09856107	RAF1	3	0.03674299	0.19704966	0.214268454	0.343957878
cg03461781	RAGE	14	0.04897117	0.41751726	0.40595649	0.540519759
cg19147912	RAI1	17	0.00088355	0.29166078	0.288524012	0.498822429
cg03791861	RAI1	17	0.00077183	0.44349559	0.490684057	0.661997417
cg06775073	RAI1	17	0.00151094	0.23487381	0.231452576	0.350568186
cg02147681	RAI1	17	0.00993335	0.13568826	0.143848894	0.248864376
cg19447962	RAI1	17	0.00065949	0.18586097	0.27117583	0.486592446
cg06685437	RAI1	17	0.00588106	0.17649019	0.16216358	0.300632092
cg13658093	RAI1	17	0.04180327	0.60688405	0.629294579	0.7312017
cg07505049	RAI14;RA	5	0.00134844	0.55296103	0.616747219	0.734135683
cg01363736	RALGAPB	20	0.00095963	0.5509638	0.580974463	0.80358574
cg14586546	RALGDS	9	0.00170239	0.218641	0.228775783	0.372111886

cg14541011	RALGDS;I	9	0.0012838	0.14481244	0.153350927	0.260157941
cg07213181	RAMP1	2	0.02649781	0.18639509	0.225537695	0.332224832
cg14819160	RAMP1	2	0.00143537	0.18492725	0.262858473	0.365953513
cg10846682	RAMP1	2	0.00472614	0.36568476	0.31380795	0.494610208
cg02909097	RAP1GAP	17	0.00100445	0.2056647	0.238404033	0.412614914
cg15070283	RAP1GAP	17	0.00455939	0.4569044	0.451763671	0.560217428
cg19458020	RARA;RAI	17	0.013012	0.1515971	0.133089507	0.255310771
cg26878655	RARA;RAI	17	0.00079224	0.48469424	0.544933311	0.685877235
cg21746001	RARG;RAI	12	0.02003709	0.51286878	0.510488332	0.636300425
cg10592478	RARG;RAI	12	0.01366893	0.28395047	0.209811068	0.415619246
cg08977270	RARRES1;	3	0.00065949	0.44598518	0.530100469	0.637267172
cg21107044	RASA3	13	0.00349176	0.31562581	0.333888241	0.511373339
cg00502575	RASA3	13	0.00100445	0.34308524	0.399696999	0.608368589
cg15082473	RASA3	13	0.01183547	0.30947736	0.332164265	0.480786974
cg06560223	RASA3	13	0.01767742	0.39175922	0.380398476	0.499656644
cg18013730	RASA3	13	0.00218799	0.33070381	0.269707606	0.485743585
cg05187651	RASA3	13	0.00183809	0.29245672	0.280922086	0.62587442
cg04493793	RASGRF2	5	0.00226669	0.61766672	0.624945489	0.73244675
cg15787807	RASGRP2	11	0.01447617	0.27487736	0.295955511	0.411895496
cg17735563	RASGRP3	2	0.00186737	0.57309736	0.602543666	0.728089526
cg21226754	RBM22	5	0.01281774	0.59997109	0.612288904	0.729578774
cg08161081	RBM39;R	20	0.01487501	0.28672442	0.283229681	0.407476921
cg27052183	RBM4	11	0.02120024	0.40809686	0.429939669	0.542711704
cg22109787	RBM43	2	0.04395526	0.25211084	0.207570032	0.357686159
cg11657849	RBM4B	11	0.02990975	0.36779607	0.404833153	0.524300014
cg08127080	RBMS1;R	2	0.01585745	0.40112897	0.340556999	0.535835449
cg02048613	RBMS1;R	2	0.02489039	0.31166856	0.302587834	0.447385549
cg25834692	RBMS3;R	3	0.0048909	0.65201163	0.634405396	0.788734184
cg08667600	RBMS3;R	3	0.00227742	0.37546177	0.376854545	0.666994086
cg22494026	RBMS3;R	3	0.00544367	0.2765817	0.211641174	0.429384499
cg22992279	RBPJ;RBP	4	0.00083228	0.3084568	0.406893165	0.559619411
cg17487741	RCBTB1	13	0.00754388	0.20140484	0.16125204	0.326755875
cg16550606	RCBTB1	13	0.00280849	0.30215641	0.250377825	0.418248164
cg13471374	RCL1	9	0.00671027	0.57660996	0.537439397	0.691420214
cg03907174	RCN3	19	0.00838913	0.33637804	0.310928452	0.521070184
cg22619810	RCOR2	11	0.00643366	0.68228033	0.697034908	0.824119827
cg10823566	RCSD1	1	0.00099652	0.20627784	0.23869318	0.418204878
cg09354263	RECQL5;L	17	0.00093925	0.25573589	0.264400033	0.470070636
cg08907956	REEP3	10	0.00218799	0.46184506	0.378662384	0.592211756
cg11937768	REEP3	10	0.00359753	0.25132966	0.317109353	0.528875554
cg14396758	RELL1;REI	4	0.01690382	0.489621	0.438373379	0.5938969
cg10630085	RELT;REL	11	0.01910846	0.43220751	0.392332515	0.572926887
cg27235337	RER1	1	0.00230634	0.22535402	0.17522316	0.398212349
cg25722041	RERE;RER	1	0.00094083	0.36264754	0.403155042	0.564637912
cg19400316	RERE;RER	1	0.01398885	0.34253753	0.32968836	0.531157608
cg13400249	RERE;RER	1	0.03349395	0.31989841	0.318101774	0.4546657

cg02780029	RET;RET	10	0.00160273	0.37334243	0.39889375	0.554464296
cg23527387	REV1;REV	2	0.01408624	0.17583817	0.229688689	0.349968216
cg15952465	RFPL4B	6	0.00490166	0.5191791	0.568296824	0.692350834
cg24250363	RFPL4B	6	0.00233734	0.25539319	0.242748233	0.402826227
cg04212651	RFPL4B	6	0.00113577	0.45644319	0.520953633	0.731601166
cg12728543	RFWD2;R	1	0.00660543	0.59681626	0.646737898	0.784695811
cg07205154	RFWD2;R	1	0.00186404	0.52780288	0.545504935	0.709805214
cg08675117	RFX2;RFX	19	0.00666067	0.1552694	0.154804567	0.268652283
cg25741259	RFX2;RFX	19	0.00383861	0.50700048	0.546786611	0.653127948
cg24937727	RGL3;RGL	19	0.0212437	0.13198896	0.125471695	0.236209567
cg14819229	RGMA;RG	15	0.00615082	0.59924072	0.570361815	0.773074614
cg05342906	RGMA;RG	15	0.00449302	0.61648231	0.605524691	0.728906
cg03462901	RGMA;RG	15	0.00073547	0.26304847	0.304182994	0.505824552
cg26905931	RGMA;RG	15	0.00067897	0.18460092	0.220188913	0.451637294
cg24569831	RGMA;RG	15	0.00127403	0.21989848	0.234835138	0.384421954
cg03971808	RGS12;RG	4	0.00065949	0.61158768	0.657215441	0.785137487
cg13702185	RGS12;RG	4	0.01159097	0.24263717	0.196163294	0.360638017
cg10954765	RGS12;RG	4	0.00857623	0.6338807	0.623852158	0.761842257
cg16324409	RGS12;RG	4	0.00075989	0.12237756	0.161849226	0.280843698
cg08403043	RGS12;RG	4	0.00113577	0.32139835	0.330721788	0.523998424
cg21836117	RGS6	14	0.00245414	0.36976299	0.385714222	0.500095799
cg24928726	RGS8;RGS	1	0.00311025	0.18122729	0.229917289	0.350167918
cg00179196	RHBDF1	16	0.00218597	0.34887575	0.351882847	0.465947964
cg07405083	RHBDF1	16	0.0009078	0.18548561	0.210944123	0.385962686
cg15120848	RHBDL2	1	0.0109037	0.58490201	0.589849702	0.703334295
cg20333869	RHEB	7	0.02120024	0.38872933	0.412044522	0.563031614
cg18237616	RHEB	7	0.01538119	0.25812684	0.223886027	0.388183495
cg20171453	RHOH	4	0.0130683	0.40532292	0.4157571	0.529808119
cg10331518	RHOT2	16	0.03129419	0.70927426	0.702749665	0.819024889
cg03054541	RIC3;RIC3	11	0.00233544	0.43312701	0.434811288	0.639477544
cg00219210	RICH2	17	0.01281867	0.33492671	0.392100386	0.547325295
cg18393905	RICTOR	5	0.00131796	0.66787305	0.587068529	0.812364116
cg22107662	RIMBP3	22	0.00103447	0.26633994	0.294267867	0.480233727
cg08591048	RIMBP3C	22	0.00154265	0.26947579	0.290912192	0.412848543
cg27036054	RIMS1;RII	6	0.00319723	0.69944583	0.672063078	0.84328404
cg01288337	RIN3	14	0.00073118	0.1725844	0.260446767	0.382068908
cg15789055	RING1	6	0.00855749	0.56371231	0.567461275	0.667969791
cg13796295	RIPK3	14	0.00796332	0.44342975	0.4453608	0.563609189
cg20989926	RLBP1	15	0.00365562	0.35008704	0.400698533	0.519303461
cg12903024	RLBP1	15	0.00755255	0.27558901	0.270041247	0.433251348
cg10173075	RLBP1;RL	15	0.00188718	0.34518961	0.387029467	0.531380631
cg00452882	RLTPR	16	0.01366893	0.40099946	0.37677707	0.531920327
cg10665490	RMND5A	2	0.00591291	0.35354402	0.369613605	0.520468015
cg06033764	RNF138;F	18	0.00279455	0.18386639	0.180799755	0.28949422
cg08058160	RNF144A	2	0.01980822	0.39044739	0.433531572	0.578596985
cg17918184	RNF144A	2	0.04084205	0.58498899	0.618460969	0.727214815

cg17589449	RNF185;F	22	0.01678972	0.23022792	0.20740006	0.330798713
cg22163472	RNF19B;F	1	0.00147518	0.56363733	0.6153886	0.741028922
cg15106452	RNF208	9	0.00758031	0.52672884	0.53083726	0.692664106
cg11606607	RNF213;F	17	0.01970757	0.63933537	0.62834071	0.788074666
cg09860921	RNF220	1	0.00331728	0.50017576	0.4899494	0.616411655
cg20748242	RNF44	5	0.00209505	0.15084352	0.140525744	0.31469909
cg19311055	RNF5P1	8	0.00194981	0.36316343	0.436375667	0.631617672
cg03163812	RNF5P1	8	0.00084118	0.20658015	0.279800695	0.550298748
cg19488149	RNGTT	6	0.01073914	0.61006539	0.64268852	0.753900772
cg26204913	RNH1;RN	11	0.0016549	0.53163443	0.516895822	0.688218491
cg24933651	ROBO2;R	3	0.00177694	0.69981188	0.766210068	0.868066302
cg14216252	ROR1;RO	1	0.00092643	0.28309303	0.291768449	0.501609708
cg19267457	ROR1;RO	1	0.00154265	0.1641922	0.171837428	0.319822197
cg20629316	ROR1;RO	1	0.01358672	0.75028686	0.750380103	0.855961554
cg14044388	ROR2	9	0.00283173	0.34379837	0.385545473	0.499295655
cg00937392	ROR2	9	0.00459107	0.46199558	0.456805786	0.589237228
cg08106764	RORA	15	0.01235814	0.34221403	0.388910394	0.498653481
cg22387253	RORA	15	0.00262173	0.47981513	0.499261343	0.610060638
cg08099431	RORA	15	0.03981181	0.37840466	0.292156503	0.533062205
cg03952578	RORA	15	0.00142756	0.61944752	0.646287067	0.764072625
cg09026994	RPH3AL	17	0.00132036	0.17249262	0.184179498	0.315814229
cg12742645	RPH3AL	17	0.02418358	0.46979684	0.42505848	0.611241961
cg22251117	RPH3AL	17	0.02078916	0.42358955	0.443380117	0.579158372
cg11407989	RPL13AP1	7	0.0081506	0.35158547	0.385512377	0.505429847
cg05181927	RPS24;RP	10	0.02381973	0.33758576	0.324639383	0.50208452
cg16361947	RPSA;RPS	3	0.00172495	0.19058202	0.195950945	0.43869287
cg20462129	RPTOR;RF	17	0.00211914	0.64600203	0.66022928	0.77218435
cg15815120	RPTOR;RF	17	0.00375204	0.60543035	0.621237475	0.749881096
cg12434898	RPTOR;RF	17	0.00327239	0.26693736	0.291818444	0.444558658
cg01911440	RPTOR;RF	17	0.02252087	0.3685837	0.364493107	0.506806678
cg14647957	RPTOR;RF	17	0.00097592	0.65356565	0.700680046	0.802720596
cg01886663	RPTOR;RF	17	0.00070678	0.33852372	0.394128751	0.535514239
cg24327522	RPTOR;RF	17	0.03856073	0.38928452	0.367861154	0.496485379
cg06053702	RPTOR;RF	17	0.00103895	0.57351706	0.576202774	0.682691554
cg02864619	RPTOR;RF	17	0.00136593	0.46050331	0.422109897	0.653587307
cg09361653	RPTOR;RF	17	0.00249952	0.44414582	0.449470462	0.561758471
cg12632411	RRBP1;RF	20	0.00954417	0.22462895	0.177830766	0.364083374
cg26161708	RRBP1;RF	20	0.00969263	0.1567002	0.119693174	0.267110255
cg01358966	RRN3P2	16	0.00215126	0.41171103	0.465254907	0.604028851
cg24517066	RSAD1	17	0.00629918	0.12269856	0.11777421	0.25049507
cg07112562	RSU1;RSU	10	0.02199481	0.58050308	0.56190824	0.683697829
cg11994630	RTKN;RTK	2	0.00320053	0.36047069	0.357134839	0.467633547
cg00326131	RTKN;RTK	2	0.00702941	0.17555475	0.150618308	0.290104666
cg18280126	RTN4RL2	11	0.01783758	0.44601204	0.437939834	0.561042028
cg02767634	RUFY1;RU	5	0.00741048	0.30715485	0.317988984	0.510086026
cg04194479	RUNDC2C	16	0.00119385	0.30683009	0.348672626	0.586552248

cg08254842	RUNDC2C	16	0.01744696	0.22747891	0.223845471	0.355458751
cg04110902	RUNX2;RU	6	0.03680055	0.54073498	0.544712696	0.659290249
cg05162523	RUNX3;RU	1	0.00376781	0.21935408	0.241784343	0.35587027
cg04875697	RXRA	9	0.00497255	0.39771309	0.418879046	0.598851214
cg02319187	RXRA	9	0.00099274	0.41409802	0.473190101	0.6972652
cg14545975	RXRA	9	0.00065949	0.31906315	0.446339868	0.618868959
cg14344989	RXRA	9	0.00220808	0.17218197	0.173166747	0.370198822
cg13425135	RXRA	9	0.00089205	0.37764328	0.433428961	0.557947684
cg04966574	RXRB	6	0.00092912	0.31668152	0.34178986	0.463294803
cg14263924	RXRB	6	0.00556197	0.58268209	0.581330373	0.702362828
cg06370855	RXRB	6	0.00316916	0.22397666	0.218148469	0.358433763
cg19033438	RXRB	6	0.01766834	0.53709649	0.546035276	0.669440875
cg07516307	RXRB	6	0.00146869	0.59133067	0.618617643	0.729520672
cg11284973	RXRB	6	0.00255774	0.24439438	0.246044202	0.428695587
cg08092515	RYK;RYK	3	0.0070502	0.5505005	0.58361277	0.713993595
cg00299070	RYR2	1	0.00089487	0.153893	0.189791782	0.335135716
cg16888559	RYR3	15	0.04742879	0.47045579	0.462067559	0.57528372
cg17080740	S100A1;S	1	0.00107001	0.19821817	0.233707056	0.370001896
cg14467840	S100A13;	1	0.0009078	0.1312236	0.158761362	0.333719087
cg10963218	S100A13;	1	0.00162163	0.18545618	0.248721913	0.430618859
cg15559456	S100A13;	1	0.00081789	0.13159529	0.153088318	0.313595511
cg20847746	S100A13;	1	0.00910491	0.19215074	0.198247422	0.360397589
cg10224088	S100A13;	1	0.01842703	0.21264263	0.205372454	0.353643107
cg11064537	S100B	21	0.02586844	0.16741603	0.140901202	0.299804535
cg04353438	SAFB2	19	0.00232154	0.21186893	0.234679215	0.395893289
cg04312620	SALL4	20	0.00597821	0.32052689	0.329274845	0.463854686
cg14160518	SALL4;SAI	20	0.01787304	0.35051143	0.312051425	0.467360003
cg13856810	SAMD11	1	0.01195883	0.37880109	0.343667544	0.490526477
cg20074307	SAMD4A;	14	0.04407901	0.31858554	0.315888787	0.451746065
cg10888242	SAMD4A;	14	0.01102429	0.27808089	0.291639928	0.444909883
cg23901920	SAMD4A;	14	0.01010631	0.1842696	0.133010672	0.295576259
cg26300500	SBF1	22	0.00434915	0.49573477	0.470821143	0.638374224
cg18019717	SBF1	22	0.00240535	0.4928004	0.514853707	0.637240433
cg04563863	SBNO2	19	0.01496968	0.42127137	0.403303193	0.543489197
cg18693051	SBNO2	19	0.00081789	0.15448578	0.169060094	0.350060725
cg26019472	SBNO2	19	0.01530568	0.199407	0.176426318	0.313432441
cg21080562	SBNO2;SE	19	0.00690449	0.26133519	0.203308834	0.377164151
cg22152492	SCAND3	6	0.01311724	0.42172971	0.408266567	0.528001894
cg20781847	SCARA3;S	8	0.00711398	0.17279858	0.153215044	0.321370829
cg22775642	SCARB1;S	12	0.00290605	0.2160546	0.210400308	0.372542906
cg13075279	SCARB1;S	12	0.00620216	0.45011853	0.466682518	0.659490954
cg14846380	SCARB1;S	12	0.04667899	0.49010178	0.543011926	0.657054345
cg09268718	SCARF1;S	17	0.00668249	0.36994116	0.298815752	0.557474948
cg22091609	SCARF2;S	22	0.00066232	0.16210454	0.248005156	0.349901684
cg18518722	SCARF2;S	22	0.00204405	0.60103325	0.587263671	0.705605346
cg24708308	SCARNA1	12	0.00380968	0.31947005	0.339489162	0.521970924

cg02237755	SCD	10	0.02817047	0.24176254	0.247040379	0.397669712
cg16744911	SCD	10	0.00168732	0.17678491	0.177511951	0.359606147
cg04881673	SCD5;SCD	4	0.00186404	0.49597794	0.505226059	0.677624562
cg23999526	SCFD2	4	0.00317338	0.14633932	0.140206425	0.252250838
cg16061228	SCN4B;SC	11	0.00450197	0.24010992	0.229607113	0.342601219
cg10294363	SCNN1A;S	12	0.01212237	0.38752877	0.371011203	0.556306933
cg12510724	SCNN1A;S	12	0.00245414	0.19049657	0.204926687	0.384130026
cg23245485	SCNN1A;S	12	0.00077468	0.35065015	0.417334772	0.530973066
cg01324261	SCRG1;SC	4	0.01561358	0.17488154	0.130058928	0.276054318
cg04858110	SCRN1;SC	7	0.00065949	0.50018804	0.585358829	0.695238629
cg24733126	SCUBE2;S	11	0.04884648	0.43086431	0.491341649	0.597440243
cg26842303	SDC2	8	0.00747631	0.40520647	0.42961865	0.629384224
cg14830748	SDC2	8	0.00447146	0.758568	0.769635897	0.871833437
cg16962683	SDC2	8	0.00854289	0.27498556	0.197079521	0.459332704
cg12190613	SDC2	8	0.00456818	0.29749128	0.324682394	0.495488095
cg14836522	SDC3	1	0.00104452	0.13912818	0.19703763	0.333848401
cg08443357	SDCBP2	20	0.03110358	0.27463874	0.279887705	0.424169092
cg05007126	SDCBP2	20	0.00244246	0.28801059	0.273411636	0.388758696
cg16786297	SDCBP2;S	20	0.00630552	0.51543095	0.551871674	0.666435157
cg08262429	SDCCAG8	1	0.00725151	0.25108537	0.209542241	0.437944422
cg18022346	SDK1	7	0.0156698	0.56465014	0.542134789	0.676388056
cg04609841	SDK1	7	0.00359376	0.21019544	0.276182156	0.395810051
cg02491557	SDK1	7	0.00752854	0.19782667	0.238766718	0.361428473
cg00711291	SDK2	17	0.00085355	0.14454195	0.181175801	0.42721323
cg14295306	SDK2	17	0.00294138	0.47815275	0.491318995	0.658521477
cg24527434	SDK2	17	0.00090315	0.26450253	0.316426673	0.454340696
cg13699963	SDK2	17	0.00100115	0.16083415	0.196151245	0.347797559
cg26503079	SDR9C7	12	0.00375204	0.57780409	0.590555624	0.714417456
cg20337028	SEC14L1;S	17	0.00405622	0.40927408	0.407398529	0.577010161
cg15046304	SEC14L1;S	17	0.0017637	0.20793461	0.225944256	0.440174196
cg21243064	SEC16A	9	0.028548	0.48057743	0.542675919	0.701800466
cg25135333	SEC16B	1	0.0108413	0.46832572	0.451728596	0.617888842
cg02289040	SEC16B	1	0.04006803	0.53679201	0.516746535	0.640015242
cg07468956	SECTM1	17	0.00556197	0.60984938	0.612853429	0.724472936
cg12862820	SEMA3A	7	0.00462124	0.5207635	0.413792354	0.687003143
cg13381679	SEMA4B;S	15	0.03941479	0.46516142	0.478145128	0.621305694
cg17819119	SEMA4C	2	0.00753573	0.31486161	0.298711617	0.460967545
cg21578050	SEMA6A	5	0.00126466	0.67546077	0.723112998	0.851228694
cg27157619	SEMA7A;S	15	0.00119471	0.23129714	0.270247036	0.487530401
cg14397309	SENP1	12	0.01857513	0.18074107	0.156358904	0.282124756
cg16976499	SENP6;SE	6	0.01613643	0.32154393	0.337484037	0.476515251
cg00871371	SEPT9;SE	17	0.00290119	0.40373196	0.461329023	0.577590423
cg07827420	SEPT9;SE	17	0.00068904	0.2952961	0.343524301	0.492541712
cg01320579	SEPT9;SE	17	0.0035878	0.25621662	0.245266479	0.418644929
cg05865280	SEPT9;SE	17	0.03585699	0.11774174	0.143193374	0.252874965
cg26079706	SEPT9;SE	17	0.00193482	0.46309858	0.487129809	0.654728653

cg17112975	SEPT9;SEI	17	0.00065949	0.15684373	0.207209211	0.336502671
cg07324245	SEPT9;SEI	17	0.00169055	0.1906593	0.186085464	0.306556145
cg17922695	SEPT9;SEI	17	0.00233298	0.43966949	0.429204764	0.591810467
cg14843920	SEPT9;SEI	17	0.00192049	0.61859413	0.625965334	0.757914918
cg02397064	SEPT9;SEI	17	0.00129597	0.62501441	0.629593288	0.746873059
cg12194864	SEPT9;SEI	17	0.00118326	0.63282838	0.66406662	0.806957844
cg17053854	SEPT9;SEI	17	0.00092022	0.69163292	0.704705678	0.815818613
cg06791979	SEPT9;SEI	17	0.00944446	0.40345515	0.387482678	0.511599004
cg01899544	SEPT9;SEI	17	0.00336646	0.54066224	0.533180264	0.645331503
cg21985691	SERGEF	11	0.00925638	0.23284411	0.192954911	0.34177187
cg12673019	SERGEF	11	0.0129251	0.29827797	0.355955391	0.5076088
cg06677021	SERINC5	5	0.00349176	0.64221255	0.599679161	0.762231803
cg24099605	SERPINA4	14	0.00574743	0.38975631	0.370126477	0.522311532
cg23524537	SERPINA4	14	0.00176484	0.25453002	0.254358923	0.389330123
cg07348591	SERPINE2	2	0.01665976	0.64863574	0.64075274	0.796491279
cg01417313	SERTAD4	1	0.00133251	0.18666496	0.184962811	0.441179638
cg17117243	SESN1	6	0.00065949	0.31319368	0.489367081	0.616922883
cg24180006	SETD3;SE	14	0.00359124	0.21783706	0.214386877	0.452792943
cg22633932	SETD7	4	0.00177922	0.48720125	0.489434452	0.683857036
cg22885062	SF1;SF1;S	11	0.01780469	0.56291858	0.563775607	0.709348542
cg03986574	SF1;SF1;S	11	0.00103895	0.57426532	0.58267636	0.746049552
cg03421300	SFN	1	0.01561381	0.5977419	0.592561455	0.717270516
cg02154585	SFRP1	8	0.00140256	0.44180901	0.485632026	0.63508139
cg21977218	SFRS12;SI	5	0.01116167	0.48014437	0.495842912	0.608587382
cg15817163	SFRS8	12	0.03354729	0.40599755	0.409490298	0.560560003
cg06555661	SFRS8	12	0.04790527	0.37038114	0.360124724	0.500652199
cg04582295	SGCA;SG(	17	0.00314038	0.50420157	0.50956231	0.612197994
cg16627285	SGCA;SG(	17	0.00626125	0.47220662	0.535228572	0.674553557
cg15756653	SGCA;SG(	17	0.00592671	0.49696128	0.526157433	0.629401451
cg22086834	SGCE;SG(	7	0.04741646	0.3496088	0.385104042	0.505301914
cg03920161	SGK2;SGK	20	0.00584729	0.39692269	0.386381243	0.507291909
cg13799263	SGMS1	10	0.0191912	0.29435561	0.259769422	0.416173281
cg09806432	SGSM2;S(	17	0.00319723	0.46290809	0.432331545	0.598442469
cg23375247	SGTA	19	0.00301632	0.3085539	0.308919624	0.533242757
cg10362613	SH2D4A	8	0.00129639	0.25091385	0.282790336	0.453046095
cg18403548	SH3BP2;S	4	0.0026101	0.21117373	0.210274767	0.364312884
cg12559925	SH3BP4	2	0.00174783	0.22767035	0.27097247	0.458200821
cg11677744	SH3BP4	2	0.00242331	0.15360799	0.160485768	0.292687594
cg09042791	SH3BP4	2	0.00157573	0.25780386	0.304656019	0.554066751
cg01854776	SH3BP4	2	0.00244246	0.19478257	0.213840627	0.374831413
cg01696784	SH3BP4	2	0.00369078	0.16771063	0.178425028	0.292403611
cg01881549	SH3BP4	2	0.01155671	0.16827076	0.162389511	0.278461874
cg21283680	SH3BP5	3	0.00081925	0.25439604	0.377937351	0.482470756
cg24203758	SH3D20	17	0.01134767	0.33361616	0.307938963	0.440750269
cg20463302	SH3D20	17	0.00388546	0.33029281	0.30735242	0.439580203
cg02030270	SH3GL3;S	15	0.00187198	0.37817503	0.413157284	0.618453372

cg04388244	SH3PXD2.	10	0.00838913	0.32537472	0.314904494	0.425384218
cg23985995	SH3PXD2.	10	0.04052226	0.25747839	0.228237574	0.363429582
cg25693317	SH3PXD2	5	0.00065949	0.14608246	0.356115597	0.540919028
cg15678744	SH3PXD2	5	0.00195549	0.12005908	0.126373081	0.236382918
cg03868944	SH3PXD2	5	0.00271346	0.21386739	0.214296976	0.429867751
cg02772159	SH3PXD2	5	0.00154265	0.35116794	0.372911327	0.53615797
cg13022534	SH3PXD2	5	0.01887509	0.20951396	0.169955536	0.362041314
cg01642901	SH3RF1	4	0.01297808	0.50935987	0.595453474	0.696148848
cg14966782	SH3RF3	2	0.0084821	0.2129175	0.211130684	0.341573262
cg19024599	SH3RF3	2	0.00163361	0.33967997	0.388476633	0.623132747
cg10842314	SH3TC1	4	0.00294511	0.47981634	0.511655013	0.614227909
cg20277282	SH3TC1	4	0.01783758	0.22950646	0.234791078	0.350891063
cg07248017	SH3TC1	4	0.00081599	0.3717793	0.430958894	0.561273475
cg14818279	SH3TC2	5	0.00192049	0.34457042	0.324678264	0.60464165
cg26474043	SH3TC2	5	0.00168532	0.21361536	0.237610395	0.356148032
cg09548638	SH3TC2	5	0.00194312	0.21482832	0.235548729	0.412969026
cg15418783	SHANK2	11	0.00499799	0.34630666	0.352825859	0.501813993
cg06868991	SHANK2	11	0.00948686	0.45212852	0.463740568	0.593297333
cg17959824	SHANK2;S	11	0.00144365	0.49746707	0.454214471	0.645019952
cg12159995	SHANK2;S	11	0.00620309	0.49168531	0.496663168	0.601419453
cg04112058	SHCBP1	16	0.01365382	0.62917803	0.553038148	0.73303494
cg00954833	SHFM1	7	0.01789337	0.28627285	0.286712945	0.462229343
cg12639933	SHMT2;SI	12	0.0074197	0.22695767	0.199615842	0.341459059
cg25004176	SHPK;TRF	17	0.00317348	0.76453158	0.769869813	0.892382287
cg17376853	SIGIRR;SI	11	0.0049312	0.35914376	0.381878256	0.53424015
cg10824582	SIK3	11	0.02823023	0.15873446	0.149290865	0.261802658
cg01324702	SIL1;SIL1	5	0.00161871	0.13260178	0.135283007	0.27143412
cg05452066	SIL1;SIL1	5	0.02775429	0.40114974	0.399312254	0.54063349
cg25462751	SIL1;SIL1	5	0.01358672	0.26210198	0.232016954	0.420736942
cg07536018	SIL1;SIL1	5	0.00520163	0.14914304	0.121477839	0.306470606
cg10538440	SIM2;SIM	21	0.04163515	0.32355105	0.298451975	0.45845057
cg24147428	SIPA1;SIP	11	0.00152023	0.22110337	0.255791464	0.357747483
cg21033440	SIPA1;SIP	11	0.00570412	0.25065821	0.221778699	0.374390973
cg18975839	SIPA1L2	1	0.00521564	0.60729005	0.606534022	0.726297431
cg04400145	SIRPA	20	0.00648443	0.2582561	0.251818579	0.384773453
cg00472837	SIRPD	20	0.00783744	0.61954421	0.602931833	0.72561509
cg24338091	SIX5	19	0.01746225	0.16528906	0.153643579	0.273610559
cg27181142	SIX5	19	0.00597387	0.29182825	0.249106757	0.405783312
cg25686087	SIX5	19	0.00347947	0.24393874	0.228694549	0.348775196
cg02050917	SKI	1	0.00114883	0.23781705	0.268541749	0.380347486
cg24377150	SKI	1	0.00247734	0.40779363	0.434998341	0.613922919
cg02360367	SKI	1	0.00103697	0.31093125	0.377351151	0.604335805
cg12473697	SKI	1	0.00524148	0.23887729	0.259074854	0.419220669
cg05948763	SKI	1	0.01783758	0.41624507	0.411555214	0.542647804
cg16575461	SKI	1	0.00129968	0.61813076	0.659715702	0.759852898
cg04619852	SKI	1	0.00176085	0.26668673	0.283639674	0.401421218

cg21405929	SKI	1	0.00186404	0.25981674	0.277885213	0.400541343
cg17854510	SKI	1	0.0097835	0.29725173	0.306738775	0.442436833
cg14178705	SKIV2L	6	0.00539451	0.63191314	0.589251711	0.742981048
cg13015485	SKIV2L	6	0.00917562	0.31250417	0.325049085	0.472220792
cg01817009	SLC12A4;	16	0.00236877	0.22762312	0.193355635	0.420445295
cg10544611	SLC12A4;	16	0.00186737	0.53045295	0.508222432	0.687346394
cg06378129	SLC12A4;	16	0.00124625	0.3554822	0.349698378	0.516489837
cg02564259	SLC12A4;	16	0.00743161	0.27567842	0.238240657	0.422517921
cg20003638	SLC12A5;	20	0.01393327	0.26814637	0.2581032	0.37571151
cg26803305	SLC14A1;	18	0.00354864	0.16386735	0.168742383	0.335433563
cg19363889	SLC14A1;	18	0.0048909	0.40128748	0.397503586	0.590799835
cg18130079	SLC16A1;	1	0.00471011	0.32927353	0.349856057	0.59985737
cg20822693	SLC16A14	2	0.00931986	0.16420227	0.14650556	0.274108156
cg18247852	SLC16A3;	17	0.0017385	0.2905478	0.30132899	0.467998761
cg03227481	SLC16A9	10	0.00555806	0.64027148	0.645254815	0.766418032
cg22231400	SLC17A7	19	0.00165724	0.1288246	0.188430651	0.326060982
cg19074198	SLC19A1	21	0.0103127	0.33862427	0.332408865	0.479801993
cg13971030	SLC1A2	11	0.00908203	0.16582311	0.191186596	0.31385622
cg13434757	SLC22A1;	6	0.04089782	0.32457573	0.298643712	0.512865763
cg12882392	SLC22A11	11	0.00280849	0.47637472	0.417631925	0.607164363
cg01822289	SLC22A11	11	0.00137441	0.45938554	0.412293646	0.590337122
cg01748193	SLC22A11	11	0.00220808	0.45802659	0.466958478	0.578102813
cg06048910	SLC22A18	11	0.00653182	0.25103506	0.231602679	0.38427178
cg24041239	SLC22A18	11	0.03954748	0.63935172	0.639090201	0.768435246
cg07291601	SLC22A18	11	0.00186737	0.42866588	0.422289014	0.686479737
cg25548316	SLC22A18	11	0.00247655	0.2638734	0.260751987	0.458190196
cg10943932	SLC22A18	11	0.01109894	0.28260986	0.252480195	0.427425137
cg02081198	SLC22A18	11	0.00429569	0.56152834	0.556935449	0.693262416
cg24102242	SLC22A23	6	0.00357777	0.4622761	0.485628101	0.589740955
cg09226986	SLC22A3	6	0.01206679	0.23370555	0.190609826	0.348846574
cg26929925	SLC23A1;	5	0.02047468	0.40690463	0.353384788	0.515978258
cg12205435	SLC23A1;	5	0.01783758	0.35502987	0.32164502	0.47975043
cg11953252	SLC24A3;	20	0.01622063	0.36223798	0.406230956	0.599491005
cg22944803	SLC24A3;	20	0.01263862	0.20112367	0.163267145	0.362512498
cg05200313	SLC24A4;	14	0.00073118	0.11299715	0.157014581	0.264421178
cg11609462	SLC25A10	17	0.01004931	0.47084003	0.480810638	0.629224845
cg04337734	SLC25A12	2	0.00454314	0.3566944	0.346028519	0.543124122
cg14213297	SLC25A25	9	0.01131777	0.36591795	0.373895885	0.480015707
cg16713727	SLC25A34	1	0.00537713	0.33401356	0.30360576	0.466334303
cg10881110	SLC25A34	1	0.00405051	0.2696193	0.264150009	0.383849903
cg09153458	SLC26A1;	4	0.00544367	0.70757999	0.686653652	0.810785845
cg25561754	SLC26A11	17	0.00194312	0.31060075	0.338472525	0.527592792
cg10930502	SLC26A11	17	0.00329066	0.17952237	0.165808074	0.286081143
cg09479512	SLC27A1	19	0.00141699	0.16167365	0.17534925	0.337442076
cg07826642	SLC29A1;	6	0.00917562	0.20636356	0.200543692	0.372502597
cg02354828	SLC29A3	10	0.00554086	0.53888927	0.558689527	0.673752626

cg06702884	SLC29A3	10	0.00124758	0.29320907	0.344893147	0.473393966
cg05345039	SLC2A1	1	0.00088355	0.31205899	0.377659091	0.611085912
cg05942022	SLC2A1	1	0.01786937	0.21125577	0.195476425	0.324770345
cg20294984	SLC2A1	1	0.00073732	0.53368693	0.639397866	0.781460114
cg11381149	SLC2A12	6	0.01481543	0.29908296	0.369317751	0.470078672
cg07656922	SLC38A1(	17	0.00325324	0.30528707	0.282968581	0.454027038
cg02339793	SLC38A1(	17	0.00206218	0.34435226	0.353372599	0.54038016
cg18025838	SLC38A1(	17	0.00429038	0.60223636	0.570170228	0.70658356
cg14019146	SLC38A3	3	0.00856489	0.15288828	0.143966466	0.258524967
cg04682699	SLC38A3	3	0.00279442	0.32453425	0.356947057	0.471794465
cg22059438	SLC39A4	8	0.02911369	0.35678695	0.340274749	0.461467949
cg21461745	SLC39A11	17	0.00442346	0.38781491	0.472876312	0.634322611
cg24136932	SLC39A14	8	0.00604317	0.44149848	0.521539114	0.637005107
cg26750489	SLC39A7;	6	0.00168532	0.58809175	0.536503954	0.759119134
cg25059165	SLC39A7;	6	0.0027878	0.33531256	0.318424743	0.499658228
cg21580456	SLC41A3;	3	0.00203531	0.46300261	0.47060732	0.603324061
cg09478995	SLC43A1	11	0.0048909	0.48452446	0.509405321	0.630943505
cg17330838	SLC43A1	11	0.00381594	0.18407047	0.17885962	0.293206884
cg27315497	SLC43A1	11	0.00065949	0.14946697	0.206009353	0.367168653
cg09361598	SLC43A2	17	0.02595684	0.29645606	0.321173873	0.437494723
cg09197783	SLC43A3;	11	0.00617318	0.65147187	0.639021522	0.759229915
cg06561886	SLC44A2;	19	0.00359274	0.33385304	0.288362627	0.443129643
cg06033721	SLC44A3;	1	0.00481427	0.57932765	0.589055228	0.734007204
cg01921484	SLC45A4	8	0.01943315	0.71305594	0.630448754	0.893973876
cg22073332	SLC4A4;S	4	0.00432931	0.52508952	0.554731308	0.667539113
cg13763232	SLC6A6;S	3	0.00405622	0.27251408	0.269730703	0.422789038
cg04833514	SLC6A9	1	0.0212437	0.47729937	0.474691559	0.584246826
cg10752421	SLC7A1	13	0.00202547	0.44739152	0.503626903	0.65385044
cg26117398	SLC7A1	13	0.00344347	0.1930356	0.160344757	0.305338177
cg06665333	SLC7A5	16	0.00083329	0.70807752	0.7283224	0.843208659
cg04171052	SLC7A5	16	0.00182941	0.28572278	0.304980306	0.471375974
cg26637881	SLC7A5	16	0.00227742	0.27009258	0.276984594	0.450510022
cg12833872	SLC9A3	5	0.00480838	0.13294638	0.118981557	0.24066077
cg08074555	SLC9A3R2	16	0.01009622	0.17930055	0.146486038	0.282675379
cg03158310	SLC9A3R2	16	0.01991471	0.27008964	0.248570691	0.375728414
cg01346158	SLC9A3R2	16	0.00113894	0.60787174	0.61101578	0.864099101
cg04558907	SLC9A9	3	0.02111228	0.61224378	0.593591756	0.712273219
cg04860674	SLC9A9	3	0.03120023	0.36765382	0.415809657	0.519823227
cg13008977	SLIT3	5	0.00101815	0.32554405	0.400487744	0.562567208
cg13908833	SLIT3	5	0.00258198	0.37815148	0.369186629	0.478857645
cg21953891	SLIT3	5	0.00337598	0.23729055	0.216614496	0.428240387
cg05906024	SLIT3	5	0.00133856	0.34700028	0.396034823	0.635970519
cg25984601	SLIT3	5	0.00168656	0.24755521	0.247610717	0.449278365
cg17229698	SLIT3	5	0.00147518	0.32681263	0.383381263	0.581476761
cg18364770	SLIT3	5	0.00156239	0.26273161	0.280242883	0.397968413
cg22631387	SLIT3	5	0.00170786	0.63070971	0.640875123	0.74926929

cg04093349	SMAD6;S	15	0.00459107	0.56723119	0.556225505	0.673942014
cg23172828	SMAD6;S	15	0.01095648	0.63253988	0.624746092	0.766256675
cg00350432	SMAD6;S	15	0.00202967	0.35179358	0.389826025	0.512440907
cg02184281	SMAD7	18	0.00471363	0.54063343	0.521405025	0.710858197
cg19430553	SMAD7	18	0.0135118	0.19256963	0.172678222	0.335508088
cg08151828	SMARCA4	19	0.01980822	0.3002768	0.294848805	0.445144147
cg01982788	SMARCC2	12	0.00440148	0.21179985	0.220611216	0.321219648
cg02596233	SMARCD3	7	0.00333032	0.37157553	0.352947273	0.497715002
cg14636077	SMCR5;R/	17	0.00211366	0.4205669	0.467827663	0.61714116
cg08593157	SMCR5;R/	17	0.00215739	0.12027342	0.119207927	0.229035018
cg06217803	SMO	7	0.00670817	0.26099569	0.268725278	0.373250626
cg18653027	SMOC1;S	14	0.01311902	0.47218933	0.49590922	0.619416077
cg23972474	SMOC1;S	14	0.00126804	0.13628648	0.165670051	0.277318734
cg02873263	SMOC1;S	14	0.00344865	0.49404444	0.479639147	0.631350974
cg19628299	SMOC2;S	6	0.00182941	0.1882879	0.203083733	0.33478041
cg14329365	SMOC2;S	6	0.00141699	0.31903492	0.344705563	0.468328432
cg13705956	SMOC2;S	6	0.00311324	0.41659383	0.469466652	0.595258055
cg10041417	SMOC2;S	6	0.00184921	0.25961034	0.26495154	0.498707108
cg02662873	SMOC2;S	6	0.01163705	0.67035565	0.671686626	0.778278199
cg04967737	SMOC2;S	6	0.00379406	0.43729313	0.429752826	0.61723373
cg09499136	SMOC2;S	6	0.00537713	0.40242774	0.374633943	0.557593819
cg24114708	SMOC2;S	6	0.00289141	0.34360807	0.384623564	0.572441765
cg02129017	SMOC2;S	6	0.00144352	0.58175419	0.603912698	0.743757627
cg25599473	SMOC2;S	6	0.01029727	0.22636618	0.206491075	0.384453704
cg08112248	SMOC2;S	6	0.01313275	0.47037888	0.450623771	0.586480802
cg14806083	SMOC2;S	6	0.02381297	0.31305523	0.367082198	0.495965586
cg17802216	SMOC2;S	6	0.00099652	0.47901904	0.552367512	0.736758604
cg04467832	SMOC2;S	6	0.00104778	0.34765869	0.36933065	0.496574712
cg22967008	SMOC2;S	6	0.00076471	0.46950827	0.586302021	0.708937784
cg26505935	SMOC2;S	6	0.00636561	0.48470146	0.517219651	0.640811761
cg03490766	SMOC2;S	6	0.00179812	0.52589364	0.556737263	0.66383652
cg15741247	SMOC2;S	6	0.0034485	0.58868683	0.595778229	0.724029875
cg21755672	SMOC2;S	6	0.00531242	0.63009296	0.57441894	0.747802001
cg22771904	SMOC2;S	6	0.00271346	0.28235015	0.227162652	0.566583012
cg15792134	SMOC2;S	6	0.03137447	0.19718524	0.178995332	0.310708149
cg07979566	SMOC2;S	6	0.00212345	0.54340523	0.612434329	0.719730268
cg01210622	SMPD3	16	0.01206219	0.38678029	0.340045382	0.498783476
cg27045405	SMPD3	16	0.00160662	0.58521052	0.594909931	0.724166165
cg00532477	SMPD4;SI	2	0.00801038	0.37103123	0.40052987	0.528706486
cg02973057	SMTN;SM	22	0.00713358	0.32336938	0.274317771	0.494733736
cg02415020	SMYD3;SI	1	0.00379918	0.435932	0.451369902	0.604167493
cg22024479	SMYD3;SI	1	0.00403008	0.34003829	0.342705442	0.535377851
cg12461735	SMYD3;SI	1	0.00176816	0.32180146	0.322468983	0.54419971
cg18121962	SMYD3;SI	1	0.0144255	0.28639133	0.355460112	0.466247966
cg18592195	SNAP47	1	0.02548197	0.30275891	0.259906091	0.423528248
cg05073044	SNAPC1	14	0.0019957	0.31569128	0.398697596	0.587402141

cg20643991	SNCAIP	5	0.03750141	0.31447833	0.291882688	0.443723176
cg22171145	SND1	7	0.00958999	0.28037394	0.235647836	0.393973011
cg00092551	SND1	7	0.00201167	0.18074342	0.187989115	0.351414144
cg14678084	SND1	7	0.00314361	0.13256622	0.098547884	0.252502684
cg11857142	SND1	7	0.00097053	0.20248813	0.227906004	0.394395215
cg09991306	SNED1	2	0.01935097	0.33031406	0.364396832	0.470443494
cg22635676	SNED1	2	0.00073265	0.13376029	0.181494609	0.385253222
cg23491743	SNED1	2	0.00556197	0.3815106	0.379177048	0.500349326
cg25241559	SNED1	2	0.00384818	0.62112586	0.692547028	0.796665247
cg21393619	SNORA27	13	0.03434255	0.14289365	0.110765738	0.247359693
cg23234674	SNORA74	5	0.00931986	0.21464403	0.187355448	0.316226468
cg15889424	SNORA80	21	0.00068904	0.33697918	0.400123329	0.565043941
cg09199848	SNORA80	21	0.00245827	0.45726954	0.487907863	0.602679825
cg15075241	SNORA80	2	0.00065949	0.4163127	0.569765073	0.716638431
cg24722354	SNORD10	1	0.00878702	0.47364882	0.498487136	0.667131613
cg27565803	SNORD10	1	0.00589356	0.25663499	0.223146803	0.413469855
cg00581541	SNORD10	1	0.0062271	0.64257696	0.626518424	0.790044902
cg00098985	SNPH	20	0.00159903	0.21781648	0.221848434	0.354713322
cg13399741	SNPH	20	0.0008696	0.21415179	0.229186575	0.352138436
cg14992108	SNTB1	8	0.00354163	0.62417571	0.630437244	0.761742707
cg25352714	SNX1;SNX	15	0.01257717	0.56675375	0.508506776	0.688610613
cg14556808	SNX19	11	0.00195	0.65616904	0.61445509	0.799003116
cg08763673	SNX2	5	0.00209505	0.65135202	0.614993637	0.775983681
cg03512808	SNX29	16	0.0355165	0.48093703	0.476054553	0.597938444
cg08073667	SNX29	16	0.00606409	0.57692273	0.603943959	0.709400985
cg12205429	SNX8	7	0.00679551	0.15256657	0.119561368	0.266112642
cg09672545	SNX9	6	0.00394919	0.59113505	0.551911637	0.71810105
cg00429523	SOBP	6	0.01753222	0.41273941	0.402448762	0.538035372
cg07944936	SOBP	6	0.00491697	0.14075209	0.11796474	0.252544139
cg06583438	SOBP	6	0.00298303	0.52072199	0.612765732	0.713085437
cg17217443	SOCS7	17	0.00569156	0.16611369	0.135354402	0.30560985
cg01123186	SOCS7	17	0.00805596	0.16177441	0.137973798	0.325619276
cg05679440	SOLH	16	0.00204611	0.44250208	0.410949664	0.556616625
cg04076387	SOLH	16	0.00798815	0.52381098	0.51135324	0.642545548
cg17369406	SON;SON	21	0.00975209	0.32242867	0.407699615	0.510429057
cg16622520	SORBS1;S	10	0.00219711	0.27631772	0.282790113	0.459947746
cg12197399	SORBS2	4	0.00367708	0.27406384	0.294675397	0.442686912
cg16871385	SORBS2	4	0.00853102	0.23819324	0.289227272	0.399198222
cg19216056	SORBS2;S	4	0.01771787	0.22087632	0.217306913	0.360413295
cg26583078	SORBS2;S	4	0.00582851	0.17699275	0.196883497	0.384399576
cg15883603	SORBS2;S	4	0.04016502	0.17338522	0.197313894	0.308931062
cg18515303	SORCS1;S	10	0.01033091	0.25733832	0.256497714	0.390285446
cg23649083	SORCS2	4	0.00095484	0.36742708	0.375125037	0.487471952
cg14180415	SORCS2	4	0.00174783	0.35705049	0.385938871	0.515110448
cg18803251	SORCS2	4	0.0040624	0.61395899	0.642221498	0.786444698
cg19593680	SORCS2	4	0.01613643	0.31191601	0.315909126	0.454437135

cg15989318	SORCS3	10	0.01377008	0.19825359	0.178500802	0.309604452
cg01898628	SOST	17	0.00677233	0.31579627	0.332206995	0.478796315
cg10473369	SOX15	17	0.00685368	0.32678384	0.305508146	0.438029026
cg06205574	SOX5;SO›	12	0.00099274	0.33868245	0.433894381	0.675529712
cg06764736	SOX5;SO›	12	0.00079512	0.73196143	0.65915592	0.856708713
cg26716834	SOX8	16	0.00133251	0.63462729	0.638800922	0.789662207
cg08992854	SOX8	16	0.00458661	0.31396218	0.27224507	0.565151065
cg06234051	SOX9	17	0.01365382	0.18640743	0.173170121	0.301621534
cg18693004	SP4	7	0.02308074	0.34100621	0.410701879	0.546624644
cg06571753	SPARC	5	0.00763406	0.31145029	0.34033618	0.483226758
cg27460824	SPATA13;	13	0.00656134	0.23296557	0.232977039	0.463387338
cg25104266	SPATA13;	13	0.01977061	0.47337303	0.498552853	0.634354538
cg01688202	SPATA2;S	20	0.00530406	0.37621862	0.39797243	0.534345113
cg06905367	SPATS2L;!	2	0.01891191	0.22061506	0.210595591	0.362337856
cg00556627	SPATS2L;!	2	0.02169667	0.66543439	0.641567217	0.777380793
cg14106933	SPATS2L;!	2	0.00458537	0.33605379	0.370487988	0.511309934
cg02882004	SPATS2L;!	2	0.00295368	0.18684923	0.142991291	0.366647434
cg07180355	SPEN	1	0.002727	0.50660775	0.524153865	0.626233593
cg15754752	SPG20	13	0.00131453	0.16810494	0.195686865	0.482949306
cg21056788	SPG20	13	0.00279455	0.16116388	0.165447043	0.33581867
cg05635923	SPG20	13	0.00248044	0.26519186	0.305419813	0.57975526
cg21484515	SPG20	13	0.01457962	0.20690294	0.160860286	0.347268183
cg00084432	SPG20	13	0.00756169	0.17767978	0.157115951	0.360415137
cg09410612	SPG20	13	0.00717306	0.24427067	0.267323669	0.368740058
cg01279418	SPG7	16	0.00375204	0.53320351	0.487918374	0.657609315
cg02207944	SPG7;SPG	16	0.01842786	0.56359443	0.542131739	0.668824872
cg01539849	SPI1;SPI1	11	0.02087024	0.31284667	0.269413406	0.425364678
cg14074366	SPIN1	9	0.02050584	0.21235255	0.168813798	0.341159671
cg00401471	SPINT2;SI	19	0.00299315	0.23799513	0.321873705	0.483802777
cg05604800	SPIRE2	16	0.01456284	0.48798368	0.498540457	0.611027068
cg20551922	SPNS3	17	0.00155548	0.30934953	0.322601825	0.50493352
cg02065148	SPON2;SI	4	0.00074381	0.36219039	0.452716374	0.558580972
cg01571977	SPPL2B;SI	19	0.00586842	0.26066585	0.27294132	0.381479324
cg22854448	SPRED2	2	0.00065949	0.19428879	0.361311969	0.617839323
cg08651590	SPRED2;S	2	0.00065949	0.16983606	0.225141283	0.482151588
cg23632432	SPSB1	1	0.00124152	0.31431368	0.347874322	0.528302283
cg09256832	SPSB1	1	0.00459082	0.22365031	0.219600632	0.421916813
cg04778236	SPSB1	1	0.01515174	0.25152851	0.259212495	0.390947636
cg24597774	SPSB1	1	0.01269426	0.19206662	0.162837359	0.305607196
cg26348847	SPTB	14	0.00129968	0.18106985	0.190389594	0.29365035
cg02531794	SRCRB4D	7	0.01162689	0.32735311	0.319216836	0.470604436
cg23875758	SREBF1;S	17	0.00206218	0.17761804	0.174162128	0.331454622
cg17354987	SRGAP1	12	0.00930715	0.44018877	0.533412017	0.638241853
cg04938109	SRGAP1	12	0.03951832	0.44598368	0.423631037	0.592382043
cg11014073	SRGAP1	12	0.00161582	0.27704132	0.309955459	0.572177924
cg12587213	SRGAP2;S	1	0.00398606	0.72439209	0.763376219	0.863975935

cg05905586	SSBP2	5	0.00756169	0.33472461	0.331956424	0.53908603
cg06988422	SSBP3;SSI	1	0.0042395	0.43973424	0.453073612	0.622279847
cg23544107	SSBP4;SSI	19	0.00158181	0.24504738	0.226289701	0.345306551
cg24878115	SSBP4;SSI	19	0.00081925	0.26277577	0.345397466	0.519777143
cg19589652	SSC5D	19	0.00703549	0.32071279	0.308602351	0.446000311
cg14338936	SSH2	17	0.01334584	0.39598067	0.342683275	0.528128929
cg06422039	SSTR5;LO	16	0.01093065	0.2265755	0.181510493	0.350241869
cg18312113	SSU72	1	0.00211366	0.51650334	0.541547767	0.671792933
cg24991874	ST14	11	0.01663906	0.47541753	0.509393309	0.63980568
cg05284912	ST14	11	0.00290605	0.14361063	0.149755205	0.264001192
cg04273299	ST3GAL1;	8	0.00748137	0.54954963	0.523775019	0.71620224
cg12831076	ST3GAL1;	8	0.02133627	0.31574009	0.254018866	0.419573077
cg11015768	ST3GAL5	2	0.00218063	0.19352198	0.192130914	0.355876749
cg00249584	ST5;ST5;S	11	0.00367708	0.14176635	0.147955297	0.296348946
cg11020894	ST6GAL1;	3	0.00245414	0.20045479	0.227681441	0.446197774
cg05720226	ST7;ST7;S	7	0.00234919	0.40302244	0.430954262	0.57309041
cg25784872	STAG1	3	0.02385226	0.4259504	0.432465923	0.597435284
cg03906031	STARD10	11	0.00092849	0.5611794	0.59891609	0.710604454
cg03575969	STARD10	11	0.00353178	0.19331321	0.186719904	0.304191643
cg13860360	STARD13	13	0.0042141	0.12364586	0.097381116	0.224461388
cg01146501	STARD4	5	0.00422785	0.26898541	0.314226033	0.460305557
cg12312086	STARD9	15	0.00113577	0.32717335	0.37758318	0.572223252
cg20173260	STAT1;ST	2	0.00099274	0.21530579	0.239432595	0.534098476
cg14951497	STAT1;ST	2	0.02817047	0.43219424	0.446665999	0.582972434
cg02733918	STAU1;ST	20	0.04539328	0.43391018	0.44275195	0.552161963
cg19728382	STC2	5	0.00065949	0.17728222	0.244424097	0.359640558
cg07109745	STIM1	11	0.00147518	0.21232804	0.260928024	0.451778258
cg10564791	STK10	5	0.00141131	0.43925524	0.466854689	0.59669682
cg23423191	STK10	5	0.00102765	0.47312211	0.523172554	0.684268872
cg03534504	STK10	5	0.00078196	0.27803651	0.37757541	0.619927858
cg26006084	STK10	5	0.01782206	0.33736089	0.324942616	0.447920359
cg16711597	STK24	13	0.00197576	0.52115819	0.476190264	0.694759755
cg09110502	STK24	13	0.00126816	0.41701956	0.545474925	0.686820467
cg07340007	STK24;STI	13	0.0020873	0.11192874	0.118854166	0.271716003
cg16924565	STK24;STI	13	0.00855749	0.56143921	0.515573263	0.687848098
cg01442200	STK24;STI	13	0.00073547	0.49920976	0.548937761	0.736102525
cg11966803	STK24;STI	13	0.00756169	0.54241174	0.563195004	0.696268085
cg23724635	STK24;STI	13	0.00806542	0.66097449	0.67327237	0.791557418
cg10533161	STK24;STI	13	0.00230968	0.46870893	0.44238473	0.606025156
cg14582191	STK32B	4	0.00124152	0.36975572	0.494854403	0.632814275
cg16033410	STK32C	10	0.01240534	0.52119604	0.488922885	0.621603446
cg18518262	STK32C	10	0.02340764	0.2396565	0.22502829	0.342360353
cg15246131	STK32C	10	0.00948686	0.19650219	0.271809507	0.381124693
cg23433370	STK38L	12	0.00639646	0.28409175	0.332685499	0.448960328
cg13345122	STK39	2	0.0065693	0.23376071	0.322395735	0.447671869
cg07992385	STK39	2	0.01440243	0.21322207	0.220362158	0.373680145

cg13431028	STK39	2	0.00480767	0.19755053	0.214646236	0.365628272
cg11775828	STK39	2	0.00123203	0.19600906	0.246518201	0.370047961
cg06631999	STMN1	1	0.00193817	0.35282026	0.403575598	0.600596984
cg13668495	STRBP;ST	9	0.00568196	0.45989224	0.505704413	0.650650116
cg02068351	STX7	6	0.00504019	0.2544512	0.277777798	0.39750826
cg04225902	SUB1	5	0.00971274	0.60435148	0.610211922	0.736598655
cg02662441	SUFU	10	0.00289119	0.72679545	0.71782099	0.842575513
cg03369906	SUFU	10	0.00286384	0.20769143	0.166898549	0.334446332
cg10260044	SULF1;SU	8	0.00209505	0.25068995	0.221876468	0.465269159
cg21130926	SULF2;SU	20	0.02418358	0.48659735	0.109577751	0.287419462
cg01003859	SULT1C2;	2	0.00430406	0.39121473	0.378990427	0.537362684
cg25426867	SVIL;MIR9	10	0.00231479	0.15943507	0.173058407	0.343285177
cg24726783	SVIL;SVIL	10	0.001445	0.66623348	0.663563071	0.774378983
cg25334461	SVIL;SVIL	10	0.01044398	0.59780968	0.586688906	0.737167969
cg03108697	SWAP70	11	0.00714351	0.4543581	0.466574418	0.670748531
cg08010619	SWAP70	11	0.00092022	0.20265991	0.25001902	0.443543145
cg10023915	SWAP70	11	0.01216466	0.67325735	0.682366682	0.78976027
cg15173428	SYN3;SYN	22	0.00747148	0.27483957	0.316692236	0.430436775
cg19987354	SYNE2;SY	14	0.00168532	0.23566126	0.253109598	0.521148141
cg13057576	SYNE2;SY	14	0.0218987	0.57579241	0.558888979	0.680106695
cg01340991	SYNGAP1	6	0.00201017	0.31049141	0.340350708	0.465014618
cg14533068	SYNJ2	6	0.00081925	0.24507996	0.305668715	0.46475945
cg05951351	SYNM;SYI	15	0.03680055	0.54520266	0.541055791	0.663182234
cg05106892	SYNM;SYI	15	0.00537348	0.26599463	0.235544347	0.383223165
cg01228193	SYNPO	5	0.0012838	0.23675454	0.237459206	0.464220213
cg26444282	SYNPO;SY	5	0.00911948	0.30840459	0.260405544	0.432093315
cg05714559	SYNPO;SY	5	0.0091896	0.32721471	0.269571139	0.437740289
cg06559925	SYNPO;SY	5	0.00111057	0.3120802	0.330781553	0.49128599
cg01025434	SYNPO;SY	5	0.00197007	0.18096355	0.181843081	0.356047759
cg03634767	SYNPO;SY	5	0.00656134	0.47980492	0.495603256	0.645183549
cg13991728	SYNPO;SY	5	0.00140436	0.20672209	0.212712099	0.452743775
cg27039501	SYNPO;SY	5	0.00126059	0.41282705	0.400547975	0.661327353
cg18416096	SYNPO;SY	5	0.00101815	0.33701172	0.335448218	0.450079935
cg02991753	SYNPO2	4	0.01311533	0.45639705	0.537029135	0.649266408
cg05697785	SYNPO2L	10	0.0097835	0.41369777	0.440594651	0.552399715
cg27550918	SYNPO2L;	10	0.0018345	0.29221722	0.306841801	0.476696242
cg09575189	SYT8	11	0.00080095	0.38653645	0.45178664	0.594251237
cg02294791	SYT8	11	0.00068904	0.42873127	0.500035546	0.632201228
cg21544658	SYT8	11	0.00066232	0.39963293	0.482058522	0.607422891
cg24331162	SYT8	11	0.00073118	0.36281484	0.418334482	0.556807376
cg12748890	SYTL1	1	0.02469417	0.18200683	0.141754052	0.282316897
cg11123303	TAAR2	6	0.03100155	0.44690492	0.423035266	0.572733357
cg11404751	TACC2;TA	10	0.04904378	0.22316433	0.21945264	0.334075066
cg11535638	TACC2;TA	10	0.00971058	0.53205629	0.524002009	0.666845647
cg02684964	TACR2	10	0.00458992	0.26538549	0.262533988	0.385416858
cg00563768	TAF15;TA	17	0.03035371	0.20444719	0.227369226	0.327470377

cg05595429	TAF1A;TA	1	0.01852003	0.23844977	0.258000942	0.375738757
cg13257421	TAF1B	2	0.00297143	0.12763963	0.130851023	0.251677695
cg00473045	TAF2	8	0.00301844	0.21688955	0.164352011	0.407825052
cg18848959	TAF4	20	0.00092849	0.19813586	0.197304829	0.37280299
cg22778957	TAF4	20	0.00135654	0.16938751	0.171718068	0.278062613
cg20353780	TAOK3	12	0.02802634	0.33053107	0.367174338	0.51070824
cg10117400	TBC1D14,	4	0.00610999	0.15840293	0.127233471	0.263573484
cg17854297	TBC1D16	17	0.00158181	0.54951262	0.578002345	0.686129253
cg07618085	TBC1D16	17	0.03767458	0.63147882	0.646300114	0.769740389
cg17295878	TBC1D16	17	0.0022221	0.25735894	0.262896285	0.453011265
cg23651872	TBC1D16	17	0.01365382	0.278631	0.285807103	0.470386459
cg19004465	TBC1D16	17	0.02252087	0.21638932	0.203806903	0.361380184
cg13466546	TBC1D16	17	0.00065949	0.14995021	0.20062866	0.36734723
cg06856735	TBC1D16	17	0.00258141	0.34687143	0.314267106	0.465161608
cg12314682	TBC1D16	17	0.00520163	0.26592676	0.23862361	0.410875478
cg16461139	TBC1D16	17	0.00463097	0.43579681	0.408991808	0.637268938
cg09675820	TBC1D16	17	0.01426642	0.33699398	0.319959279	0.447169274
cg24805759	TBC1D16	17	0.00921039	0.16986636	0.153106379	0.271246405
cg21048763	TBC1D16	17	0.04196515	0.25873358	0.210376712	0.37023935
cg07397612	TBC1D22/	22	0.00111301	0.26035689	0.270440026	0.601225383
cg09692364	TBC1D4	13	0.00295889	0.40154925	0.362360869	0.608554776
cg12594615	TBC1D8	2	0.00854392	0.38441432	0.382464383	0.51093218
cg02050985	TBC1D8	2	0.02773862	0.39766315	0.419505035	0.527096754
cg17662369	TBC1D9B,	5	0.00552473	0.21240986	0.216655929	0.33132138
cg11142556	TBCD	17	0.03615697	0.49313064	0.480244337	0.622138924
cg00830755	TBCD	17	0.03541631	0.54101333	0.539004772	0.683960238
cg08811443	TBCD	17	0.0130683	0.68108044	0.718983932	0.870363571
cg25399461	TBCD	17	0.00425638	0.17285847	0.172390636	0.27298082
cg13304638	TBCD	17	0.01012853	0.30583436	0.396513358	0.509583113
cg01771850	TBCD	17	0.00249952	0.19240347	0.151657416	0.386163503
cg05398905	TBCD	17	0.00223558	0.18956952	0.163460825	0.34743674
cg03535099	TBCD	17	0.00381594	0.47760693	0.470287857	0.643220114
cg02768197	TBCD	17	0.00757332	0.45423002	0.447962929	0.610204283
cg25055744	TBCD;ZNF	17	0.00832892	0.48446718	0.449182274	0.638798125
cg04216936	TBCK;TBC	4	0.00237565	0.49029486	0.566755305	0.686277758
cg10714160	TBL1XR1	3	0.01195883	0.22011809	0.257778024	0.418136595
cg03656099	TBX2	17	0.00065949	0.12870331	0.247508571	0.407908581
cg22635491	TBX3;TBX	12	0.00243287	0.2341952	0.362411428	0.470768148
cg16277169	TBX3;TBX	12	0.00243925	0.41347978	0.48818017	0.634943914
cg09413529	TBX3;TBX	12	0.00251443	0.38153359	0.418899231	0.527618559
cg11246938	TBX3;TBX	12	0.0035878	0.46129596	0.500930812	0.647922628
cg01078446	TBX3;TBX	12	0.00442269	0.3081052	0.391000726	0.50778132
cg13030332	TBX3;TBX	12	0.00126059	0.58016327	0.64149055	0.814421947
cg09053536	TBX3;TBX	12	0.00283769	0.53278535	0.572023675	0.771087943
cg16406892	TBX3;TBX	12	0.00144365	0.3219171	0.464848156	0.601995158
cg26196480	TBX5;TBX	12	0.00188499	0.17380981	0.2608949	0.395568137

cg16458436	TBX5;TBX	12	0.0194986	0.27169847	0.303899306	0.415128858
cg16559598	TBX5;TBX	12	0.00178269	0.12110663	0.195433595	0.310582738
cg06911121	TBX5;TBX	12	0.00160732	0.1253986	0.189588803	0.341756218
cg12670347	TBX5;TBX	12	0.0135118	0.35516959	0.387356819	0.501124137
cg05555207	TBX5;TBX	12	0.0035878	0.18113093	0.214969532	0.395909276
cg03559073	TBX5;TBX	12	0.00183659	0.12373557	0.194424658	0.296883668
cg12610207	TBX5;TBX	12	0.00455738	0.12935099	0.161720342	0.283106933
cg22929859	TBX5;TBX	12	0.00523353	0.22466763	0.296863071	0.425915173
cg04562909	TBX5;TBX	12	0.03146035	0.23601723	0.259222944	0.391507334
cg06725552	TBX5;TBX	12	0.00165724	0.16008268	0.237890672	0.380591888
cg17554126	TBX5;TBX	12	0.00324717	0.19896223	0.293835361	0.4387511
cg08184159	TBX5;TBX	12	0.0017228	0.19299536	0.292846826	0.398648701
cg25399352	TBX5;TBX	12	0.00623289	0.20584144	0.292359029	0.409433434
cg18689332	TBX5;TBX	12	0.0011414	0.13582829	0.203030795	0.351473391
cg11357746	TBX5;TBX	12	0.00666111	0.15008203	0.189155961	0.331198767
cg07658357	TBX5;TBX	12	0.01068658	0.25349646	0.317209274	0.418907389
cg11841394	TBX5;TBX	12	0.00744196	0.14568772	0.178523501	0.290531793
cg13823415	TBXAS1;T	7	0.00196226	0.66838818	0.630778956	0.79564904
cg09697084	TBXAS1;T	7	0.02332665	0.62696835	0.624761937	0.727033809
cg23759823	TBXAS1;T	7	0.00854392	0.45775707	0.446975155	0.619505717
cg06462921	TBXAS1;T	7	0.00931986	0.44863216	0.420018142	0.590154823
cg24348495	TCEA2;TC	20	0.01158752	0.25446465	0.253332855	0.407834073
cg03946671	TCEA2;TC	20	0.00582851	0.16961401	0.159915927	0.290960787
cg12176783	TCEA2;TC	20	0.01230099	0.11799712	0.114342793	0.235448728
cg12452800	TCERG1L	10	0.01776628	0.34659544	0.341895232	0.48233869
cg03303475	TCF19;TC	6	0.03095521	0.40702512	0.425524758	0.52891297
cg20217307	TCF19;TC	6	0.00828598	0.67144073	0.659394014	0.809997485
cg22207584	TCF7L1	2	0.00189591	0.41369504	0.430250526	0.536998746
cg07168930	TCF7L2;T	10	0.01522043	0.34223713	0.41130414	0.522482248
cg04743859	TCIRG1;T	11	0.00405622	0.30752916	0.305478062	0.415090778
cg04435320	TCP11L1;	11	0.02791506	0.46870778	0.500080157	0.601395911
cg27240158	TEAD1	11	0.04832512	0.40451014	0.401135438	0.526015357
cg18555698	TEAD1	11	0.00866121	0.42167255	0.3920236	0.546977132
cg25878366	TEAD1	11	0.00149451	0.2979337	0.333680637	0.580877713
cg15722533	TEAD1	11	0.00173224	0.27558934	0.385193523	0.54596767
cg24902858	TECTA	11	0.00367963	0.37758848	0.399487077	0.54527514
cg04370442	TEPP;TEP	16	0.00343305	0.34064498	0.267165904	0.45589135
cg19127638	TET1	10	0.00315788	0.40908267	0.524400905	0.627161151
cg01093854	TET1	10	0.00174311	0.31091464	0.340353791	0.604276123
cg04066190	TFAP2A;T	6	0.00205966	0.13943951	0.219627276	0.340229948
cg21082644	TFDP1;TF	13	0.0048909	0.51680765	0.516275252	0.638242339
cg18335233	TG	8	0.00524789	0.3366872	0.407366041	0.564587324
cg11155356	TG;SLA;SL	8	0.00227382	0.38450998	0.409545101	0.606188145
cg16967578	TGFB2;TG	1	0.01321831	0.31565995	0.381947182	0.546433869
cg06270049	TGFBR2;T	3	0.00452504	0.22932142	0.243030977	0.446597474
cg05450916	TGFBR2;T	3	0.00414415	0.34123196	0.36952951	0.555020488

cg24719910	TGFBR2;T	3	0.00456818	0.34907371	0.405853332	0.56189269
cg21814995	TGFBR2;T	3	0.02636662	0.39215758	0.408765018	0.562498313
cg03630790	TGFBR2;T	3	0.00233284	0.47529061	0.546912159	0.680676843
cg06047655	THADA;TI	2	0.00841172	0.11644552	0.119570118	0.233327188
cg06678594	THADA;TI	2	0.02686773	0.47490617	0.500137974	0.633122512
cg04684014	THADA;TI	2	0.00219224	0.37691047	0.495377101	0.630705916
cg21679402	THAP4	2	0.02522711	0.63123503	0.607154249	0.737234451
cg01637789	THBS1	15	0.00997964	0.22853969	0.292138145	0.459460041
cg00836101	THBS1	15	0.01485881	0.23131273	0.248748671	0.425384381
cg18234606	THBS1	15	0.00463727	0.25058341	0.27378525	0.475863729
cg16805094	THBS2	6	0.00139627	0.26410224	0.309435835	0.45340421
cg24391982	THBS2	6	0.00116579	0.1976099	0.225641804	0.339360364
cg14627862	THBS2	6	0.00408989	0.40912022	0.358462095	0.580226621
cg24462200	THOP1	19	0.004607	0.69940575	0.717618156	0.848560949
cg19919217	THRA	17	0.00295256	0.18932111	0.182727027	0.31465504
cg14829516	THRA;NR1	17	0.00337598	0.15057832	0.141910332	0.254193981
cg01341751	THRA;NR1	17	0.00589429	0.27731867	0.278479768	0.390738248
cg02030908	THRA;NR1	17	0.00327239	0.32576156	0.308720325	0.451876723
cg03724728	THRA;THI	17	0.00353146	0.31084559	0.32832519	0.4990453
cg01190276	THRB;THI	3	0.00087343	0.34084888	0.41889252	0.580199982
cg16505204	THRSP	11	0.00144772	0.12715888	0.133672322	0.327386342
cg21864730	THRSP	11	0.0024796	0.29557654	0.289062314	0.49565514
cg13726166	TIAF1;MY	17	0.00165724	0.23476155	0.260911942	0.413284701
cg17730176	TIAF1;MY	17	0.00216343	0.3880886	0.438763783	0.587805751
cg04228709	TIAM2;TI/	6	0.00100011	0.33881585	0.462472421	0.579200479
cg18132964	TIAM2;TI/	6	0.00337598	0.23230392	0.21721042	0.347633428
cg06694381	TIAM2;TI/	6	0.00456818	0.46355944	0.433866795	0.619894917
cg26934881	TIAM2;TI/	6	0.00457701	0.68237394	0.695077526	0.795577699
cg21539594	TIE1	1	0.00080373	0.3474685	0.423535374	0.57890471
cg26195652	TIE1	1	0.00219711	0.46614823	0.529811432	0.673983941
cg15756407	TIMM8B;	11	0.03515143	0.44624417	0.482404095	0.603236265
cg15106744	TIPARP	3	0.00539963	0.33773184	0.402777169	0.573660317
cg07230792	TJAP1;TJ/	6	0.00178025	0.38962425	0.389654969	0.561975902
cg12427941	TJAP1;TJ/	6	0.00602029	0.32213863	0.307638026	0.433712977
cg25069807	TK1	17	0.00119376	0.54901892	0.539608436	0.72211327
cg06098276	TK1	17	0.0023397	0.3891833	0.403144035	0.535930629
cg03702413	TLCD1;TL	17	0.00377297	0.33330084	0.277755974	0.435542355
cg14199629	TLE1	9	0.00480767	0.29084297	0.329845387	0.520355097
cg08025415	TLE2	19	0.01124644	0.2632167	0.258706259	0.407833424
cg24772525	TLK1;TLK1	2	0.00630552	0.60503774	0.649643625	0.795131153
cg02898977	TM9SF1;T	14	0.01314425	0.63153439	0.634176962	0.876182171
cg05679002	TMCC1;TI	3	0.04127719	0.28938149	0.264691331	0.415651123
cg23167246	TMCO3	13	0.02686773	0.22375448	0.182084097	0.33131101
cg24121069	TMCO3	13	0.00587243	0.21598933	0.211005795	0.355020783
cg01383440	TMCO3	13	0.00703549	0.78316712	0.786345778	0.894791834
cg22355895	TMED5;TI	1	0.00223213	0.45659394	0.485655616	0.656231325

cg02768480	TMEM10(	17	0.01039075	0.20551965	0.219280538	0.346176197
cg10976623	TMEM10(	17	0.01342353	0.47917544	0.468096411	0.626448045
cg11758345	TMEM10!	17	0.00143965	0.53549033	0.541728069	0.701657131
cg03841376	TMEM10!	17	0.00590146	0.33015684	0.320057449	0.45600508
cg09695261	TMEM10!	17	0.00134844	0.47381663	0.492202385	0.615489007
cg21735384	TMEM10!	17	0.00243597	0.42798146	0.408464575	0.545920052
cg21591452	TMEM10!	17	0.00207996	0.36507108	0.378493423	0.500345725
cg22532155	TMEM10!	17	0.00152023	0.47255141	0.4645871	0.594623117
cg06406157	TMEM13:	11	0.00385922	0.25536339	0.256102394	0.391140216
cg06630988	TMEM13:	12	0.01148808	0.35067491	0.335415516	0.463876164
cg15936861	TMEM13:	12	0.0235591	0.23478753	0.245769477	0.364728522
cg07803856	TMEM15:	3	0.00113577	0.43779183	0.457519547	0.558676404
cg00584026	TMEM17!	4	0.00457701	0.25184187	0.270908985	0.433281664
cg03424213	TMEM17!	4	0.00510852	0.67322921	0.652433887	0.780873905
cg12989041	TMEM18	6	0.00143343	0.65763297	0.665939631	0.809605686
cg01581326	TMEM18	6	0.00418551	0.18112404	0.122368108	0.319968162
cg18172186	TMEM20(	6	0.00551198	0.45802785	0.470705031	0.645944176
cg03890628	TMEM20:	1	0.00499799	0.24154745	0.250776772	0.452903586
cg00175735	TMEM30(	3	0.01891102	0.27618088	0.247932574	0.478411379
cg18811916	TMEM33	4	0.01236318	0.63054694	0.616053673	0.746662607
cg08844169	TMEM39,	3	0.0482028	0.35090998	0.394340475	0.514508286
cg13075537	TMEM57	1	0.00178025	0.17176221	0.166744485	0.318583601
cg07192718	TMEM61	1	0.00756169	0.33884372	0.294822927	0.443650549
cg21191255	TMEM80,	11	0.00133251	0.1375205	0.152350806	0.258100646
cg12703825	TMEM82	1	0.00472673	0.27930759	0.25565157	0.399646101
cg17977470	TMEM88	17	0.00628685	0.27828722	0.236237468	0.417596444
cg14068176	TMEM8B,	9	0.00207724	0.63689206	0.687206012	0.798673783
cg01263574	TMEM8C	9	0.00095748	0.10574211	0.134695779	0.266141455
cg15844374	TMPRSS5	11	0.00788453	0.27446078	0.263736822	0.398259477
cg18165914	TMPRSS5	11	0.00194185	0.20177667	0.22146333	0.348562201
cg25460340	TMPRSS5	11	0.00124758	0.43713646	0.490514729	0.651907677
cg05183271	TMPRSS5	11	0.00380612	0.18592356	0.194413843	0.318150513
cg23871994	TMPRSS5	11	0.00101054	0.33358091	0.372719125	0.521230311
cg23033870	TMPRSS5	11	0.00128378	0.18204931	0.197544885	0.298923971
cg06733903	TMTC1	12	0.01124644	0.20960595	0.182152886	0.318029726
cg18927181	TMTC1	12	0.00244246	0.26141369	0.244608156	0.532256512
cg01401465	TMTC1	12	0.00201167	0.60233914	0.616960298	0.745594736
cg03191794	TMTC2	12	0.0015244	0.46797569	0.494846839	0.6811013
cg18587137	TNFAIP2	14	0.00730525	0.2894106	0.24881136	0.401304288
cg18620571	TNFAIP2	14	0.01148141	0.27901749	0.204129111	0.386810452
cg10501093	TNFAIP2	14	0.01303821	0.26318189	0.200579115	0.374386767
cg08301307	TNFAIP2	14	0.00128725	0.52007578	0.542583542	0.677179146
cg07086380	TNFAIP8;'	5	0.00277534	0.23628606	0.283587574	0.47635901
cg02134720	TNFRSF1:	8	0.03771398	0.20482628	0.153926645	0.307180188
cg11665613	TNFRSF1E	1	0.00252833	0.21428163	0.219260135	0.419728469
cg08547922	TNFRSF8	1	0.00311547	0.13489982	0.144099138	0.286155355

cg26691200	TNFRSF8	1	0.00154265	0.44587532	0.464843021	0.654994011
cg00569500	TNFRSF8	1	0.00236541	0.14262503	0.13432894	0.282702523
cg10425291	TNFRSF8;	1	0.00094183	0.57507966	0.629272663	0.733815175
cg07499259	TNFRSF8;	1	0.01430511	0.38538338	0.385068793	0.498851032
cg00463840	TNFRSF8;	1	0.01372997	0.2491786	0.216305561	0.357185082
cg15690400	TNFRSF8;	1	0.00614688	0.31515717	0.378095344	0.571170542
cg17267493	TNFSF12;	17	0.00838913	0.54438402	0.529631213	0.659864171
cg23321140	TNIP1	5	0.01172466	0.16570165	0.13204702	0.281713747
cg09813400	TNIP1	5	0.0006736	0.39770837	0.442570764	0.550736645
cg25737411	TNK1	17	0.01907154	0.45629188	0.471843078	0.578250316
cg00190795	TNK2;TNI	3	0.00158181	0.26861767	0.25435244	0.397668708
cg20092936	TNK2;TNI	3	0.00230116	0.54198124	0.534406218	0.705181187
cg21553700	TNK2;TNI	3	0.00221513	0.60108344	0.580360041	0.784001029
cg08767936	TNK2;TNI	3	0.00081413	0.36790637	0.325734289	0.596592221
cg08048178	TNK2;TNI	3	0.00236285	0.69515571	0.583715838	0.868110062
cg19233001	TNNT2;TI	1	0.00520163	0.39158686	0.388379834	0.49178693
cg19673237	TNP1	2	0.01632034	0.34267045	0.411070934	0.516840451
cg20310485	TNP1	2	0.00337568	0.27102237	0.322035801	0.50646081
cg18314765	TNP1	2	0.00140274	0.30406028	0.404194712	0.636038698
cg19384546	TNP1	2	0.0094452	0.26308363	0.278437337	0.412931421
cg12845678	TNRC18	7	0.00214604	0.64763046	0.670361104	0.775902129
cg06947694	TNRC18	7	0.00078196	0.42754457	0.493799341	0.680265521
cg20025824	TNRC18	7	0.00103697	0.45649471	0.541154617	0.673473727
cg10327399	TNRC18	7	0.00182121	0.52125823	0.538266166	0.659670499
cg00795125	TNRC18	7	0.00344958	0.45957877	0.483710339	0.662584503
cg23058673	TNRC18	7	0.00126816	0.31479132	0.33531966	0.459716765
cg14517056	TNRC18	7	0.01863458	0.5845996	0.580386471	0.74410692
cg06420129	TNRC6A	16	0.00264473	0.29502226	0.274973708	0.437442505
cg09029192	TNRC6C;T	17	0.00101423	0.52052525	0.518255475	0.641495809
cg14410503	TNS3	7	0.00519569	0.22611244	0.211891909	0.393598583
cg13800949	TNS3	7	0.00545658	0.31447305	0.280941066	0.42432143
cg27525731	TNS3	7	0.00335524	0.38184114	0.377217818	0.508469199
cg12292947	TNS3	7	0.00568798	0.25003934	0.215445378	0.391246878
cg24661184	TNS3	7	0.00471011	0.37212128	0.468494024	0.575281439
cg03958512	TNS3	7	0.00068904	0.37751572	0.468055091	0.74787922
cg20782724	TNS3	7	0.00067897	0.51365246	0.595184843	0.746276165
cg27275374	TNS3	7	0.00132175	0.28750229	0.344773578	0.525221748
cg06871974	TNS3	7	0.03552968	0.45661896	0.439839449	0.560985454
cg25854703	TNXB	6	0.00354346	0.54523243	0.492602055	0.698775341
cg03822479	TNXB	6	0.00450197	0.5845237	0.575100728	0.728919258
cg04389484	TNXB	6	0.02231558	0.65049431	0.624468643	0.752422852
cg14924781	TNXB	6	0.00796332	0.33915786	0.354622425	0.567205474
cg21042276	TNXB	6	0.00135325	0.12848211	0.138014407	0.238494501
cg14669361	TNXB	6	0.00157286	0.15119064	0.167517958	0.328005784
cg23462632	TNXB	6	0.00626821	0.18360106	0.183466984	0.285316479
cg13798746	TNXB	6	0.00208745	0.15535211	0.14862353	0.304465555

cg15318957	TNXB	6	0.00524148	0.11291052	0.110259907	0.252063324
cg08660959	TNXB	6	0.00228672	0.20516764	0.246214199	0.367526017
cg07485305	TNXB	6	0.00714351	0.13199851	0.122224596	0.23764665
cg20407792	TNXB	6	0.00218799	0.14702968	0.144549943	0.348045235
cg23186098	TNXB	6	0.00549982	0.25865704	0.264266939	0.423940237
cg19609334	TNXB	6	0.00159949	0.44708108	0.450375234	0.602909941
cg02151997	TNXB	6	0.00414201	0.33556747	0.387534277	0.584825747
cg04757901	TNXB	6	0.00276855	0.27660517	0.28404036	0.527241489
cg03130418	TNXB	6	0.01024014	0.30945687	0.337642716	0.4503544
cg14196170	TNXB	6	0.00614688	0.3620051	0.366758567	0.519460337
cg02989255	TNXB	6	0.01456284	0.32082497	0.325665287	0.4359032
cg26266427	TNXB	6	0.00615357	0.33125241	0.380509021	0.496480626
cg10365886	TNXB	6	0.00290119	0.36710914	0.449061511	0.630460872
cg14188106	TNXB	6	0.00183657	0.25984045	0.330853752	0.480752462
cg07524919	TNXB	6	0.02203296	0.29882231	0.362878897	0.480790738
cg27387193	TNXB	6	0.00527609	0.30364106	0.382148222	0.483410229
cg19267551	TNXB	6	0.00269197	0.24834584	0.32642005	0.440449004
cg01992382	TNXB	6	0.00170239	0.17331522	0.23767608	0.381102444
cg16834823	TNXB	6	0.00073265	0.20057536	0.307196899	0.491964581
cg10923662	TNXB	6	0.00158407	0.16093788	0.24373314	0.383810801
cg24882324	TNXB	6	0.00065949	0.16189808	0.324811608	0.451875183
cg15196197	TNXB	6	0.00073265	0.14239092	0.233237265	0.378481151
cg12694372	TNXB	6	0.00081599	0.22240251	0.369165807	0.524327319
cg15265085	TNXB	6	0.00073118	0.26242565	0.44538525	0.576045154
cg08650890	TNXB	6	0.00071094	0.25111109	0.371493407	0.492752198
cg21642103	TNXB	6	0.00071094	0.21671254	0.281217124	0.390220616
cg13698691	TNXB	6	0.00065949	0.1757775	0.269883786	0.370953655
cg01485117	TNXB	6	0.00073547	0.20092235	0.286521547	0.406236529
cg15745284	TNXB	6	0.00113577	0.46136317	0.525609338	0.654334017
cg00979704	TNXB	6	0.00600791	0.17502453	0.133483279	0.286782159
cg13739666	TNXB;TN)	6	0.00467005	0.36626252	0.344553697	0.481752062
cg26537323	TNXB;TN)	6	0.00575836	0.3139684	0.28393119	0.414236775
cg26779330	TOLLIP	11	0.00947108	0.27108208	0.291251893	0.40750861
cg23041020	TOLLIP	11	0.02835259	0.36301162	0.344194978	0.474484852
cg17092096	TOLLIP	11	0.00436875	0.33482161	0.28791765	0.435606641
cg03466237	TOLLIP	11	0.00925638	0.44670839	0.427338481	0.642613791
cg10696445	TOLLIP	11	0.00207762	0.27239234	0.273659594	0.406820806
cg05311626	TOLLIP	11	0.00163336	0.14677262	0.14702831	0.355658015
cg03044471	TOLLIP	11	0.00450197	0.32944558	0.314368495	0.466788956
cg23178186	TOLLIP	11	0.00948869	0.61515607	0.598884902	0.730028897
cg24186398	TOLLIP	11	0.00748137	0.43943784	0.43986894	0.638396938
cg22050611	TOLLIP	11	0.00166045	0.33685399	0.298702283	0.534281157
cg12601909	TOLLIP	11	0.00137973	0.61822193	0.629055408	0.749329454
cg17591574	TOR3A	1	0.0011104	0.16902537	0.185038257	0.376235691
cg13962286	TP53AIP1	11	0.03173228	0.17149785	0.151696416	0.282042942
cg03760518	TP73;TP7	1	0.00380283	0.30003799	0.301476414	0.40812866

cg06624991	TPCN2	11	0.00217555	0.62058456	0.634540116	0.752035821
cg10256045	TPCN2	11	0.00444533	0.23784645	0.203869186	0.461460989
cg18053228	TPCN2	11	0.0097133	0.19764683	0.136626095	0.302550805
cg08847919	TPCN2	11	0.00565706	0.53643556	0.502004736	0.667289309
cg08192033	TPD52L1;	6	0.02899242	0.26737283	0.23850348	0.381139939
cg02807882	TPD52L1;	6	0.01589679	0.23178957	0.216546659	0.344876154
cg12017635	TPH2	12	0.02828378	0.1352745	0.115757531	0.236969735
cg27174787	TPM4	19	0.0219159	0.19344943	0.167012971	0.297783543
cg22546122	TPM4	19	0.01093903	0.16912197	0.136638503	0.276144865
cg01950495	TPPP	5	0.00146177	0.28331512	0.26284605	0.439928646
cg03655389	TPPP	5	0.01256322	0.73880039	0.777209137	0.884930425
cg18389810	TPPP2	14	0.00160583	0.16627629	0.197271393	0.344538432
cg13597809	TRAF2	9	0.00317466	0.32344817	0.317745417	0.466417185
cg00827663	TRAF2	9	0.00810401	0.67048691	0.654377848	0.782666594
cg18372196	TRAM2	6	0.01657372	0.4089394	0.364139925	0.525939885
cg11723904	TRAP1	16	0.00446647	0.47265723	0.490056759	0.660177464
cg18219994	TRAPPC9;	8	0.00108661	0.33983385	0.407482276	0.578405362
cg24317069	TRAPPC9;	8	0.00085355	0.62432842	0.651134876	0.75689783
cg03282105	TRAPPC9;	8	0.00149058	0.26695218	0.28492409	0.443720263
cg02528189	TRAPPC9;	8	0.00083228	0.27757119	0.31927577	0.444933653
cg17290943	TRAPPC9;	8	0.00154265	0.55423154	0.586242616	0.714562945
cg08504029	TRAPPC9;	8	0.00189637	0.31734101	0.358350039	0.48743349
cg24388061	TRAPPC9;	8	0.00702537	0.21784356	0.231974264	0.343610155
cg20946943	TRAPPC9;	8	0.00944201	0.3278417	0.330145319	0.543568632
cg11256152	TRAPPC9;	8	0.03739058	0.47178738	0.496423877	0.616287421
cg14023589	TRHDE	12	0.01198766	0.70691901	0.696850132	0.816552543
cg08322448	TRIM2;TR	4	0.00073118	0.52659293	0.575779256	0.735746111
cg00787996	TRIM31	6	0.00911882	0.5425209	0.546252977	0.693681944
cg27351624	TRIM31	6	0.0008774	0.42380171	0.478750514	0.6508889
cg22262055	TRIM38	6	0.03829642	0.51133121	0.462454067	0.617221116
cg03306027	TRIM62	1	0.00134295	0.22436086	0.233140629	0.366528028
cg12077698	TRIM62	1	0.00094876	0.36839512	0.37768394	0.626179603
cg26780705	TRIM65	17	0.00555868	0.17287217	0.166326852	0.283533668
cg23367683	TRIM7;TR	5	0.02673403	0.52113283	0.525065123	0.64957915
cg00608540	TRIM7;TR	5	0.000777	0.34197914	0.364929445	0.502228881
cg10179300	TRIO	5	0.00129968	0.57193422	0.578762414	0.758479926
cg26689441	TRIO	5	0.01729366	0.47141978	0.483180722	0.600675683
cg22126499	TRMT2A;T	22	0.00204405	0.33268707	0.323419107	0.458597738
cg00530568	TRPC7;TR	5	0.00299125	0.42024792	0.389837742	0.563235814
cg03716852	TRPM4	19	0.00248015	0.2322856	0.209882204	0.367717211
cg18225577	TRPM4	19	0.00133251	0.18720128	0.152067843	0.377591582
cg14220181	TRPM8	2	0.00482325	0.25891802	0.211142643	0.36117369
cg26358374	TRPV4;TR	12	0.00113577	0.12723513	0.143362126	0.297361049
cg12765716	TRPV4;TR	12	0.0010753	0.12357209	0.132705002	0.234944821
cg04717595	TRPV4;TR	12	0.00091163	0.1160977	0.142654141	0.257950571
cg07675656	TSC2;TSC	16	0.00155292	0.24181814	0.257656888	0.417402784

cg02364279	TSC2;TSC	16	0.00157567	0.38949013	0.407147049	0.534810244
cg08936056	TSC2;TSC	16	0.00544367	0.31874606	0.306256496	0.468466276
cg01769813	TSC2;TSC	16	0.00602029	0.36556421	0.336822865	0.515982146
cg22497575	TSC22D1	13	0.00481061	0.15368395	0.15595867	0.314228363
cg03097389	TSC22D1;	13	0.00866099	0.13980604	0.124180719	0.289433777
cg17600231	TSC22D1;	13	0.00840516	0.09550183	0.093064231	0.200994771
cg25247409	TSNARE1	8	0.021196	0.22514496	0.190769039	0.326599471
cg09663584	TSPAN3;T	15	0.00174311	0.70277365	0.743963742	0.848997166
cg13071185	TSPAN4;T	11	0.00679946	0.32278579	0.301530991	0.434209067
cg13614606	TSPAN4;T	11	0.00186737	0.53731899	0.532677783	0.692864338
cg17998346	TSPAN4;T	11	0.00097618	0.52045733	0.537090639	0.646361399
cg09296942	TSPAN5	4	0.01506509	0.38063079	0.337133817	0.494837386
cg23246886	TSPAN5	4	0.00221105	0.63061836	0.592263855	0.750258526
cg00615469	TSPAN5	4	0.00803433	0.20067976	0.194612316	0.323585158
cg20639398	TSPAN9;T	12	0.0026964	0.60777808	0.616325345	0.729732629
cg25672027	TSPO;TSP	22	0.00078196	0.29894394	0.362249186	0.548220859
cg06766636	TSPYL1;TS	6	0.03110358	0.27310999	0.28984864	0.394940206
cg03646994	TSSC1	2	0.03352901	0.38795167	0.369213431	0.51671231
cg03025986	TSSC1	2	0.00336646	0.56415173	0.635427628	0.735618263
cg19536922	TSSC1	2	0.00092849	0.67105988	0.705051534	0.819702169
cg04951371	TSSC1	2	0.00249468	0.26474096	0.242430628	0.390753334
cg11685249	TSSK1B;M	5	0.00140443	0.39232635	0.395766443	0.569400451
cg25714774	TTC15	2	0.00220808	0.68327645	0.642427264	0.788059747
cg12392225	TTC15	2	0.00209654	0.10982347	0.093297914	0.305576559
cg27639897	TTC15	2	0.00215692	0.12866421	0.141546704	0.347677009
cg25113123	TTC15	2	0.00279704	0.54849077	0.543284496	0.670214445
cg19022697	TTC22	1	0.00399104	0.23952702	0.183546469	0.341426802
cg15645660	TTC22	1	0.01151007	0.30880173	0.243422773	0.413622084
cg05401069	TTC39C	18	0.01842724	0.20035493	0.141203587	0.332559157
cg18719665	TTC39C	18	0.01263862	0.14846309	0.10799271	0.273016052
cg11222440	TTC7A	2	0.00806542	0.35875129	0.354529595	0.565977966
cg04437845	TTC7A	2	0.00301844	0.55478137	0.523951304	0.667364778
cg23870587	TTC7B	14	0.03686125	0.32462927	0.343326917	0.445815136
cg10062874	TTC7B	14	0.0120439	0.22170837	0.220320926	0.416008761
cg04458567	TTL	2	0.00500442	0.37927107	0.458780115	0.606919636
cg05545910	TTLL1;TTL	22	0.00093925	0.37440594	0.41728725	0.616607313
cg03959147	TTLL7	1	0.02817047	0.62436874	0.666817871	0.772428185
cg10087754	TTYH3	7	0.03722047	0.26234907	0.23231435	0.367620588
cg22788953	TTYH3	7	0.00768774	0.27134945	0.275451844	0.382239948
cg05519002	TUB;TUB	11	0.00115308	0.28479109	0.302399774	0.48386278
cg10133610	TUBB2B	6	0.01641391	0.43555485	0.48690848	0.625196532
cg10724940	TUBGCP6	22	0.0048909	0.41512094	0.408460467	0.525613993
cg00696711	TULP4;TU	6	0.02367946	0.78661552	0.738386801	0.888460641
cg03467567	TULP4;TU	6	0.01263862	0.59874528	0.549570474	0.755399759
cg07705604	TULP4;TU	6	0.00147208	0.43750342	0.44895755	0.610162108
cg13068216	TULP4;TU	6	0.00070134	0.21670728	0.278336671	0.575340211

cg12601856	TULP4;TU	6	0.00074016	0.2715312	0.315003628	0.643671075
cg18978680	TULP4;TU	6	0.00107573	0.23575003	0.251314934	0.569411303
cg19987965	TULP4;TU	6	0.00113577	0.30002409	0.373187181	0.673621233
cg07165851	TULP4;TU	6	0.00280852	0.15723126	0.156227749	0.278676881
cg23816737	TULP4;TU	6	0.00230768	0.17329653	0.213941666	0.398825987
cg02060443	TULP4;TU	6	0.00102765	0.47508283	0.518430919	0.772210441
cg14755699	TWIST2	2	0.04265641	0.26994805	0.314586976	0.460276325
cg22986947	TXNDC5;T	6	0.00374355	0.49923369	0.495667066	0.610708686
cg21480173	TYK2	19	0.00534171	0.65476914	0.683914689	0.794107975
cg23306063	TYK2	19	0.00890116	0.31843803	0.291389145	0.426518394
cg24509225	UBAC2;U	13	0.00177922	0.23447447	0.245384825	0.437647586
cg20555462	UBASH3B	11	0.03053716	0.45459772	0.48091802	0.610743797
cg23081194	UBE2G2;U	21	0.04568556	0.6636732	0.701904916	0.815140348
cg27031484	UBE2H;U	7	0.0261269	0.25982429	0.278476697	0.430670768
cg10628201	UBE2L3;U	22	0.01109894	0.25185563	0.20841805	0.4218088
cg17957183	UBE2N	12	0.02445578	0.39617978	0.436175516	0.54933073
cg12547839	UBE2O	17	0.00190848	0.15770438	0.156260978	0.275492527
cg02390515	UBE4B;UI	1	0.00429038	0.49933462	0.487674463	0.636212149
cg04440724	UBN1;UB	16	0.0062669	0.79338172	0.792572645	0.910144199
cg22840650	UBTD1	10	0.00357848	0.14245219	0.135509224	0.328780843
cg08499158	UBTF;UBT	17	0.00381594	0.41661422	0.421857327	0.591487616
cg10308265	UBTF;UBT	17	0.00341796	0.33477582	0.294053509	0.452053516
cg10402321	UBXN11;U	1	0.00186737	0.2810318	0.33078103	0.46335739
cg21934504	UBXN6;U	19	0.02510464	0.40189324	0.363473241	0.512549977
cg01637548	UCKL1;M	20	0.00301844	0.17757143	0.18530083	0.289914213
cg27243297	UCMA	10	0.00838112	0.22596694	0.207221123	0.366460531
cg15671083	UCN3	10	0.01511943	0.38710647	0.423199802	0.542765641
cg00621899	UFSP1;UF	7	0.00503527	0.31357062	0.282139207	0.425220033
cg17028658	UFSP2;UF	4	0.0102761	0.50278901	0.510380063	0.693256545
cg19931529	UGP2;UG	2	0.00815824	0.14023118	0.144195703	0.279976873
cg07823755	UGT1A10	2	0.0103127	0.58996171	0.615261047	0.724487666
cg22609233	UGT8	4	0.02002229	0.32925987	0.393071092	0.520608377
cg03240473	UMODL1;	21	0.00359124	0.46569042	0.467360958	0.642972863
cg14380013	UNC119B	12	0.00376892	0.20599159	0.227870358	0.365373882
cg26716839	UNC119B	12	0.02289835	0.44218254	0.464563527	0.595250253
cg12407791	UNC13D	17	0.00065949	0.29142782	0.363285207	0.485771657
cg23470227	UNC13D	17	0.00107905	0.53804833	0.513228096	0.65583454
cg16759416	UNC5B	10	0.01406453	0.4971562	0.468953242	0.616245173
cg16490191	UNKL	16	0.01496968	0.69871718	0.691573027	0.803844253
cg01682111	UNKL	16	0.00377206	0.09777091	0.095789374	0.201285561
cg03622431	UOX;DNA	1	0.01887509	0.41900166	0.396205641	0.536140489
cg07151747	UPP2;UPI	2	0.00100059	0.30600418	0.341144104	0.495421329
cg17710536	URGCP;U	7	0.00664725	0.41649952	0.396381257	0.583312427
cg19985030	USH1G	17	0.00106261	0.43900817	0.466617094	0.640982959
cg04431002	USP10	16	0.0026958	0.30561287	0.346859309	0.530193368
cg07795346	USP10	16	0.00200001	0.15273007	0.116581239	0.28755982

cg01019355	USP12	13	0.00273943	0.25469319	0.236784248	0.401058695
cg02387639	USP13	3	0.00606409	0.32560932	0.354729087	0.554754861
cg02756502	USP20;US	9	0.00362881	0.19720631	0.206266447	0.348204178
cg24427271	USP36	17	0.00330705	0.39841311	0.433906366	0.643391238
cg23496516	USP36	17	0.00186737	0.35268047	0.352197938	0.508422917
cg04491365	USP38	4	0.01713917	0.61993697	0.665958281	0.778689404
cg05638347	USP42	7	0.00177922	0.71971335	0.76514155	0.867734962
cg22849526	USP42	7	0.00798815	0.66457683	0.650836541	0.779191803
cg10752190	USP43	17	0.00575746	0.3221302	0.330732197	0.469251818
cg10098221	USP46;US	4	0.00213077	0.15734502	0.161534339	0.315726747
cg27181013	USP5;USP	12	0.01838071	0.30959183	0.292657755	0.426256991
cg05265574	USP6NL	10	0.00579337	0.16892905	0.210865167	0.342360932
cg06302998	USP6NL;U	10	0.0025513	0.15812782	0.144948667	0.301556713
cg10853431	USP6NL;U	10	0.00263648	0.35944686	0.353512815	0.561760512
cg06528737	USP6NL;U	10	0.00174311	0.42981977	0.48389023	0.647634767
cg18766900	USP6NL;U	10	0.00284268	0.30512256	0.351734816	0.533821106
cg04444027	USP7	16	0.00753569	0.18734248	0.185533821	0.339977781
cg01601926	USP7	16	0.01191142	0.28665784	0.270932702	0.397396092
cg18800739	UTRN	6	0.00569156	0.24456937	0.24983599	0.378175469
cg02475675	UTRN	6	0.01863458	0.65757927	0.648411945	0.769393662
cg20625138	UTS2;UTS	1	0.0009078	0.24341561	0.338124709	0.526573373
cg07783183	UVRAG	11	0.0012785	0.60812428	0.573872105	0.794222931
cg14321837	UXS1	2	0.01561358	0.47548656	0.498075094	0.625261441
cg10262662	VAC14	16	0.00093183	0.40656657	0.424890205	0.581309505
cg05704955	VAC14	16	0.00294138	0.15506909	0.160798511	0.271236108
cg08201451	VAC14	16	0.00358002	0.40433533	0.408580451	0.593586445
cg10216848	VAC14	16	0.00552206	0.52825026	0.436647092	0.665020444
cg02149965	VARS2;VA	6	0.02568798	0.3172493	0.296963212	0.420358426
cg17428922	VARS2;VA	6	0.00594682	0.4187706	0.451519584	0.581521568
cg15673491	VARS2;VA	6	0.00670817	0.44091734	0.432103712	0.566103002
cg26457890	VASN;CO	16	0.00109306	0.15643929	0.147510989	0.351887352
cg01851968	VASN;CO	16	0.00156869	0.38855845	0.402774714	0.529206463
cg13836412	VAV2;VAV	9	0.00521564	0.46263259	0.460666728	0.573319376
cg14286025	VAV2;VAV	9	0.00073118	0.20045684	0.244783304	0.390303442
cg13954067	VAV2;VAV	9	0.00083228	0.09010415	0.109248499	0.302605536
cg13829680	VAV2;VAV	9	0.0023323	0.57614923	0.595086042	0.755752406
cg00788204	VAV2;VAV	9	0.00405622	0.63549661	0.639550372	0.775770845
cg14308466	VAV2;VAV	9	0.00745657	0.17905658	0.186506905	0.306852984
cg13424940	VAV2;VAV	9	0.01688348	0.22116106	0.197966453	0.347440042
cg04223006	VCAN;VCA	5	0.00168532	0.17552392	0.209085526	0.353927939
cg16492597	VDAC1	5	0.00112793	0.12591483	0.135980795	0.274905781
cg25219939	VDR;VDR	12	0.00405622	0.48849149	0.482732935	0.675389664
cg12279019	VEGFA;VE	6	0.00714351	0.31704914	0.309684469	0.495576323
cg16907527	VEGFA;VE	6	0.00408296	0.59789996	0.58077853	0.729408614
cg25547939	VEGFB;DN	11	0.0115311	0.36863287	0.337952354	0.472027701
cg22658311	VEGFC	4	0.00066232	0.30637737	0.399455297	0.552896626

cg01589054	VGLL4	3	0.04187319	0.56022783	0.545076953	0.693078324
cg26015019	VGLL4;VG	3	0.01613093	0.4710436	0.502075522	0.618293663
cg17553677	VGLL4;VG	3	0.00462871	0.17339592	0.175794757	0.308822259
cg22841079	VGLL4;VG	3	0.01160447	0.21870482	0.194096306	0.378563455
cg03182000	VGLL4;VG	3	0.00315788	0.61507956	0.601653584	0.726293568
cg08815359	VGLL4;VG	3	0.00076471	0.60250107	0.637938739	0.806714131
cg23193320	VGLL4;VG	3	0.00883762	0.49272594	0.467427591	0.612175112
cg00510331	VGLL4;VG	3	0.01236318	0.48263441	0.441973462	0.603810559
cg19880465	VILL	3	0.00576931	0.2211934	0.178963765	0.374355814
cg13336326	VIPR2	7	0.02199155	0.38241874	0.47188292	0.630590641
cg14974056	VIT	2	0.01776628	0.5606209	0.559010726	0.680896952
cg17117823	VPS13B;V	8	0.01580766	0.53456892	0.524821745	0.684685051
cg18576301	VPS13B;V	8	0.00073378	0.39129543	0.466079074	0.630583846
cg18224970	VPS13B;V	8	0.00708294	0.38460047	0.423799606	0.550882241
cg07139350	VPS13D;V	1	0.0315497	0.18877201	0.128907474	0.296513678
cg26228280	VPS13D;V	1	0.01236318	0.31597404	0.264807887	0.462633321
cg24692310	VPS37C	11	0.00230116	0.37683655	0.363316734	0.486596203
cg15105901	VPS4A	16	0.00757332	0.68799135	0.694009046	0.80254323
cg23510109	VPS53;VP	17	0.00426883	0.46430569	0.385286225	0.606978449
cg09668627	VWA1;VW	1	0.01100502	0.19250292	0.201367813	0.304959097
cg15104126	VWF	12	0.02172582	0.47435482	0.502420186	0.620644806
cg20837463	VWF	12	0.0048146	0.1558543	0.129512338	0.271577655
cg10775733	WASF1;W	6	0.03752792	0.69956752	0.685917731	0.806686813
cg09537568	WDFY3;W	4	0.00195	0.34132587	0.441916436	0.586592976
cg24789587	WDR18	19	0.01045915	0.63290017	0.650061518	0.762680856
cg01755336	WDR20;V	14	0.01126796	0.33884222	0.303438366	0.456304543
cg09980114	WDR20;V	14	0.00168532	0.62864274	0.671180194	0.772537439
cg04858090	WDR27	6	0.04963055	0.72401139	0.646679749	0.824023112
cg06630010	WDR37	10	0.00896145	0.66079488	0.668606984	0.771024949
cg13504933	WDR51A;	3	0.01488167	0.46035711	0.454993484	0.595020663
cg07822618	WDR60	7	0.00154265	0.25114438	0.272932499	0.471146103
cg01302119	WDR60	7	0.00159903	0.40376061	0.421096401	0.531559632
cg08933790	WFIKKN1	16	0.01109894	0.70370097	0.718646919	0.834939516
cg08674236	WHSC1;W	4	0.0336255	0.42037574	0.440124885	0.547714747
cg00248861	WHSC2	4	0.01045915	0.37323894	0.407225497	0.543322044
cg04996238	WHSC2	4	0.0235712	0.30214216	0.253149158	0.407585217
cg18690368	WIPF3	7	0.01100502	0.23815767	0.215808798	0.382859298
cg26988194	WIPI1	17	0.00315582	0.51044737	0.52740715	0.663531207
cg17667625	WISP1;W	8	0.01317683	0.14043299	0.120469549	0.250565797
cg19804071	WISP3;W	6	0.00642271	0.26878773	0.2903974	0.40816495
cg14723977	WIZ	19	0.00372084	0.33810811	0.306047984	0.491745401
cg13481840	WNK2	9	0.00628685	0.37340095	0.376511724	0.559691662
cg02169981	WNT11	11	0.00769304	0.51692099	0.501549924	0.661845741
cg02298472	WNT5A	3	0.00066232	0.33074447	0.44524463	0.603419626
cg01777121	WNT5A	3	0.00065949	0.20429598	0.287715895	0.429041873
cg18562578	WNT5A	3	0.00092643	0.25321853	0.321381184	0.44575571

cg24216596	WNT5A	3	0.00073118	0.35020289	0.42042661	0.537744713
cg22264616	WT1;WT1	11	0.03169761	0.23702975	0.240087896	0.346812112
cg21038780	WWC1;W	5	0.01482933	0.19957257	0.20167584	0.36701098
cg03119442	WWOX	16	0.00325993	0.74615213	0.710158605	0.850116047
cg26990527	WWOX	16	0.00383265	0.6582364	0.612462777	0.773904201
cg01171429	WWP2;W	16	0.00614711	0.37046127	0.401175904	0.568735778
cg05781582	WWP2;W	16	0.00258141	0.12935148	0.118914207	0.298846572
cg26884345	WWP2;W	16	0.00148491	0.11864113	0.119049781	0.256532937
cg00657810	WWP2;W	16	0.00917376	0.16127408	0.147055859	0.323149818
cg02712546	WWP2;W	16	0.00637792	0.20146414	0.16822977	0.351782485
cg08701134	WWP2;W	16	0.00323448	0.20799215	0.214316557	0.385729414
cg08419373	WWP2;W	16	0.00190308	0.19776485	0.227927172	0.3320074
cg01656717	WWP2;W	16	0.00101054	0.14821844	0.182404151	0.374766235
cg09036558	WWP2;W	16	0.00101054	0.19048207	0.260910169	0.427782643
cg26481896	WWP2;W	16	0.00223182	0.1539091	0.185940132	0.30989549
cg02412345	XPO7;XPO	8	0.02753451	0.28335752	0.306968933	0.432809858
cg22272713	XPO7;XPO	8	0.00149088	0.11764609	0.132135863	0.254470478
cg14514120	XPOT	12	0.00081599	0.30607344	0.330981195	0.53189009
cg27170268	XRCC3;XF	14	0.00553815	0.44162936	0.453664754	0.565551451
cg02259047	XRCC3;XF	14	0.00425638	0.34957245	0.329392685	0.524215706
cg10347949	XRCC3;XF	14	0.00177143	0.52428639	0.496144327	0.684225139
cg13255208	XRCC3;XF	14	0.00113577	0.49537167	0.529275631	0.630730989
cg25561904	XRCC3;XF	14	0.00112793	0.40299058	0.399931297	0.565407515
cg02256455	XRCC3;XF	14	0.00119385	0.40089728	0.401354797	0.508756704
cg07896448	XYLT1	16	0.01024832	0.19752543	0.192963916	0.345651997
cg16546017	XYLT1	16	0.01580766	0.22834372	0.213116422	0.381271496
cg26706077	XYLT1	16	0.0078101	0.48402491	0.521353276	0.62836792
cg06066697	XYLT1	16	0.00620953	0.20948716	0.189462372	0.338248784
cg07248440	XYLT1	16	0.02174344	0.12839357	0.115656955	0.252624648
cg06635133	XYLT1	16	0.00070678	0.13620885	0.176955581	0.42193118
cg04986899	XYLT1	16	0.00151202	0.39042296	0.435647999	0.670749553
cg15839431	YJEFN3	19	0.00855749	0.29863158	0.272793762	0.405533153
cg16640973	YJEFN3	19	0.00575836	0.24413233	0.203908681	0.383534801
cg01653532	YPEL2	17	0.00158181	0.09626482	0.105368906	0.270440152
cg10948088	YPEL2	17	0.01884397	0.58286312	0.599888255	0.715252273
cg26003360	YPEL2	17	0.00339981	0.66469552	0.692834243	0.797208633
cg21364719	ZADH2	18	0.01529615	0.56517365	0.547941909	0.676856013
cg24214000	ZBED4	22	0.00298303	0.28779719	0.309320613	0.410114553
cg11049439	ZBTB12	6	0.00143276	0.18411294	0.201809435	0.43259129
cg11349594	ZBTB12	6	0.00310772	0.37354248	0.349943238	0.547380544
cg03725573	ZBTB16;Z	11	0.00079883	0.18037869	0.20605179	0.342290352
cg07631435	ZBTB16;Z	11	0.00232154	0.13621546	0.164573083	0.282396452
cg12026858	ZBTB16;Z	11	0.00169556	0.32714219	0.362438232	0.600861065
cg14373380	ZBTB17	1	0.00447146	0.33922663	0.362979287	0.495486267
cg07165288	ZBTB20;Z	3	0.00556197	0.17119899	0.151334112	0.330147535
cg27168291	ZBTB22;T	6	0.00244246	0.28885387	0.25078355	0.401440588

cg01620248	ZBTB42	14	0.00190746	0.59448268	0.567386375	0.721970029
cg09255505	ZBTB42	14	0.00997311	0.53201437	0.503861984	0.654070218
cg14918743	ZBTB42	14	0.00118952	0.29006159	0.315716731	0.518530754
cg12840850	ZBTB46	20	0.00566397	0.36838875	0.382898246	0.500895662
cg22735105	ZBTB46	20	0.00355607	0.37891485	0.333469309	0.5308399
cg04634182	ZBTB47	3	0.0051238	0.61831996	0.560710744	0.722433119
cg21250061	ZBTB5	9	0.00701147	0.18118768	0.178127215	0.325527622
cg04377850	ZC3H18	16	0.01846658	0.48403459	0.500576393	0.605823157
cg17517442	ZC3H3	8	0.00627871	0.48436133	0.493923391	0.663372471
cg15170442	ZC3H3	8	0.00474032	0.39986938	0.363753918	0.514356446
cg11329090	ZC3H3	8	0.01465218	0.66823021	0.667618897	0.76959819
cg11497758	ZC3H3	8	0.01580766	0.53420599	0.556016035	0.656034928
cg24974704	ZC3H3	8	0.00360641	0.30596519	0.266943878	0.447607073
cg12688965	ZC3H3	8	0.00097337	0.25222085	0.348021477	0.52998546
cg13191508	ZC3H3	8	0.00177922	0.22245896	0.251218359	0.430026324
cg08380064	ZC3H3	8	0.00092849	0.486919	0.465889313	0.691457004
cg04040861	ZC3H3	8	0.00186737	0.24461109	0.234233543	0.398326453
cg15681229	ZC3H3	8	0.00126059	0.45436665	0.451760631	0.583846357
cg14189808	ZC3H4	19	0.00546552	0.15993133	0.150838956	0.293231999
cg09095525	ZC3H7A	16	0.01157637	0.16845472	0.145773948	0.277237973
cg02896970	ZCCHC14	16	0.00473973	0.45009331	0.461228901	0.57421528
cg04772241	ZCCHC14	16	0.00997311	0.1917501	0.200131606	0.320259205
cg27414087	ZCCHC14	16	0.01311724	0.27856983	0.242326308	0.428999219
cg07894883	ZCCHC24	10	0.00129968	0.49666795	0.446610233	0.617475396
cg04920385	ZCCHC24	10	0.00132036	0.10631085	0.116137241	0.245956162
cg04904468	ZCCHC24	10	0.01842786	0.5574937	0.541095885	0.673235072
cg17310882	ZDHHC14	6	0.00073118	0.16431004	0.220712452	0.385004944
cg04116155	ZEB1;ZEB	10	0.00359124	0.64601629	0.665211807	0.798170224
cg17552088	ZFAND3	6	0.01608519	0.24420218	0.208154111	0.436798048
cg08858926	ZFHX3;ZF	16	0.00244887	0.46829867	0.523227842	0.680546728
cg07395439	ZFHX3;ZF	16	0.01227276	0.63094968	0.62470984	0.735154799
cg06126698	ZFPM1	16	0.01259427	0.48258323	0.387848894	0.600137635
cg04117455	ZFYVE21	14	0.0018485	0.24916298	0.225238073	0.494499731
cg04162497	ZFYVE21	14	0.00670577	0.30142925	0.29712807	0.410720564
cg06946814	ZFYVE21	14	0.00177554	0.56127224	0.556832431	0.758934979
cg13154413	ZFYVE21	14	0.00177922	0.39664143	0.449089429	0.600720079
cg04984818	ZFYVE21	14	0.01026117	0.6249691	0.642858768	0.770724047
cg09924366	ZFYVE21	14	0.00372084	0.26500882	0.243939253	0.365981507
cg10126234	ZFYVE21	14	0.00913085	0.35123214	0.345685167	0.502479046
cg21167502	ZFYVE21	14	0.02897628	0.5494859	0.507684159	0.663194402
cg25580656	ZFYVE21	14	0.00280512	0.51839505	0.515201739	0.658825939
cg08489478	ZFYVE27;	10	0.00205127	0.45277076	0.527880084	0.686632709
cg23859313	ZFYVE28	4	0.00279997	0.44377794	0.436898621	0.588169801
cg06035200	ZHX2	8	0.00519569	0.1624944	0.134079309	0.339249573
cg21926603	ZHX2	8	0.00249952	0.17004825	0.179203559	0.339673669
cg02743070	ZMIZ1	10	0.00067897	0.32870123	0.357484452	0.508985712

cg17823346	ZMIZ1	10	0.00065949	0.08168704	0.106806998	0.214255723
cg17065712	ZMIZ1	10	0.00186737	0.43874247	0.469525955	0.572157499
cg16201273	ZMIZ1	10	0.0009968	0.67884373	0.68766113	0.790332559
cg10812439	ZMIZ1	10	0.03659438	0.48832868	0.483690207	0.615648841
cg14841514	ZMIZ1	10	0.00079224	0.38353122	0.424936829	0.529388016
cg16815157	ZMIZ1	10	0.00159601	0.51423134	0.495116537	0.636011592
cg02851873	ZMIZ1	10	0.00195	0.30005263	0.358816021	0.466139158
cg01053681	ZMIZ1	10	0.00197576	0.7034744	0.702445201	0.806291754
cg09354331	ZMIZ1	10	0.00248026	0.48331846	0.475174955	0.592936482
cg10816687	ZMIZ1	10	0.00317466	0.71029176	0.709273195	0.810566437
cg14825905	ZMIZ1	10	0.00101054	0.20188747	0.249978011	0.411178404
cg20744163	ZMIZ1	10	0.00358002	0.69472628	0.722237861	0.824503472
cg02795981	ZMIZ1	10	0.00227327	0.31497863	0.282598982	0.509326248
cg20679052	ZMIZ1	10	0.00168532	0.40515998	0.39165792	0.610026811
cg25365214	ZMIZ1	10	0.00113577	0.4641514	0.466856067	0.61130682
cg04256907	ZMIZ1	10	0.00405622	0.44974764	0.451938797	0.626207773
cg15733068	ZMIZ1	10	0.00117602	0.39992908	0.416157326	0.581962587
cg24752964	ZMIZ1	10	0.00553981	0.16803086	0.147589386	0.29884247
cg19666787	ZMIZ1	10	0.00183659	0.15637601	0.149676767	0.334672325
cg13676902	ZMIZ1	10	0.00147518	0.58171298	0.651045044	0.766655448
cg19523667	ZMIZ2;ZM	7	0.00099274	0.16614396	0.191074281	0.312358774
cg05141341	ZMIZ2;ZM	7	0.00174311	0.21240528	0.225225744	0.427076151
cg15264273	ZNF169	9	0.00590538	0.69678684	0.709809938	0.82420293
cg14781805	ZNF236	18	0.03515606	0.68003857	0.645311608	0.791382293
cg10061906	ZNF34	8	0.01130868	0.16216497	0.147856595	0.263520316
cg20435267	ZNF354B	5	0.01093903	0.5315955	0.547235967	0.653930387
cg02712553	ZNF365;Z	10	0.00113577	0.61899929	0.665602169	0.783764998
cg14700504	ZNF365;Z	10	0.01057629	0.43334128	0.458352537	0.578804856
cg15872458	ZNF385A;	12	0.0009934	0.40300387	0.522001398	0.652443383
cg17970299	ZNF385A;	12	0.00732161	0.26608498	0.190462567	0.366722575
cg16000504	ZNF385D	3	0.04020956	0.15499844	0.154669443	0.261505697
cg19507160	ZNF395	8	0.00806542	0.17832779	0.174909022	0.395549631
cg10768564	ZNF414;Z	19	0.00459107	0.69565416	0.696852212	0.800277341
cg15560061	ZNF414;Z	19	0.01011462	0.27133447	0.244811152	0.37509082
cg02335656	ZNF423	16	0.01134767	0.43825908	0.394434689	0.580519162
cg27154394	ZNF444	19	0.02003709	0.62408474	0.590046235	0.774583392
cg26700277	ZNF469	16	0.00453677	0.31687789	0.33233069	0.484426524
cg10120897	ZNF469	16	0.00218799	0.33107996	0.370831539	0.539586102
cg16284674	ZNF511;T	10	0.00087343	0.30725755	0.347220096	0.597035012
cg17675150	ZNF532	18	0.02680411	0.3773816	0.405646385	0.56941405
cg25401010	ZNF532	18	0.01212299	0.41710235	0.485578979	0.621163565
cg26804891	ZNF598	16	0.00450394	0.38197828	0.322092998	0.507647387
cg22589697	ZNF608	5	0.02386355	0.41753711	0.416680787	0.524597502
cg05957504	ZNF608	5	0.00156239	0.26311783	0.322866137	0.540940771
cg23598573	ZNF609	15	0.00244887	0.45645044	0.528703568	0.673048206
cg11964338	ZNF610;Z	19	0.00868337	0.35426915	0.3799416	0.549830383

cg05342816	ZNF707;Z	8	0.0051754	0.69452177	0.587183823	0.823297834
cg14110920	ZNF740	12	0.01342633	0.17893703	0.175481847	0.283756551
cg07999732	ZNF775	7	0.00387834	0.55946554	0.551550677	0.669110696
cg21326642	ZNF777	7	0.01925109	0.74216449	0.762401514	0.863394735
cg09427605	ZNF827	4	0.00506149	0.23332922	0.19949436	0.45044939
cg22598458	ZNF92;ZN	7	0.02168773	0.20658768	0.198776908	0.318452553
cg12968041	ZNRF3	22	0.02878476	0.37650484	0.353036342	0.497211281
cg05325182	ZSWIM4	19	0.00622936	0.26778129	0.272867809	0.379982439
cg18590299	ZZEF1	17	0.0075495	0.23264525	0.277919254	0.392785387
cg09089118		7	0.00092849	0.24726873	0.311761717	0.458626571
cg01433527		7	0.00269773	0.37129241	0.391228607	0.551050672
cg18913924		7	0.00103001	0.42169427	0.455092449	0.592455991
cg07115848		7	0.0283256	0.61299861	0.631158759	0.753690353
cg23190268		7	0.02348349	0.57018623	0.644860646	0.791494347
cg05657096		16	0.01378915	0.53535366	0.546702001	0.676099363
cg26976707		11	0.02991399	0.33274073	0.322491561	0.459991491
cg02095697		2	0.04635001	0.32981558	0.310035053	0.445854456
cg23365135		7	0.03645999	0.2747985	0.287145331	0.391006306
cg16784234		7	0.00262388	0.18119543	0.200646423	0.312720619
cg03677126		5	0.01006689	0.36107642	0.410354727	0.52155883
cg15254822		7	0.01596342	0.43051205	0.457290201	0.584238461
cg04063906		7	0.00165724	0.46330015	0.533989127	0.673970845
cg02084467		17	0.00176816	0.14374618	0.168473412	0.33693482
cg04165337		8	0.03917446	0.58489155	0.438819339	0.688922623
cg08900511		1	0.02240881	0.26808822	0.268657118	0.385998073
cg05081908		4	0.00112235	0.23697284	0.268596241	0.401827883
cg18169505		4	0.00193378	0.30249731	0.31873713	0.498343547
cg02064798		4	0.00130462	0.33234944	0.354110881	0.556718441
cg09223687		4	0.00703053	0.2598113	0.226931828	0.382748954
cg15556591		4	0.00997311	0.33576217	0.316390728	0.468380104
cg25428177		4	0.00585587	0.22508865	0.228004201	0.372507058
cg01215742		16	0.00854289	0.20474911	0.165415433	0.31333024
cg08886020		16	0.01366893	0.2424484	0.215392693	0.346828261
cg16525974		16	0.00124521	0.65923915	0.629860195	0.763212748
cg00624589		16	0.00936413	0.36613595	0.32603913	0.473879954
cg18767278		5	0.00338717	0.25091209	0.217212305	0.426202095
cg09776395		17	0.00513612	0.54792309	0.594341949	0.695319798
cg09358961		16	0.0017637	0.49598773	0.516323132	0.619114924
cg06091804		4	0.00756169	0.23399787	0.24393059	0.354181809
cg09807446		16	0.01998122	0.3641908	0.330886533	0.508632352
cg21118819		1	0.00174903	0.23300947	0.244951647	0.385536038
cg25310427		11	0.00471011	0.61759879	0.627982593	0.743402968
cg13750214		19	0.00295368	0.32460322	0.291382664	0.432063914
cg10607454		7	0.00785654	0.35001151	0.328202889	0.455850208
cg11902934		5	0.01296171	0.47698852	0.482357353	0.585841287
cg18590119		6	0.04644539	0.55295399	0.568846825	0.679979852

cg04130549	6	0.00144385	0.13938268	0.159893003	0.31131095
cg05006473	4	0.00179679	0.22062094	0.252114808	0.373221078
cg22244039	4	0.01015703	0.583573	0.544603928	0.746320954
cg11174255	4	0.02248036	0.66011434	0.725538109	0.845187048
cg11599887	5	0.00403008	0.60280184	0.6182312	0.734175551
cg27039593	5	0.00741048	0.18639744	0.189528786	0.296819128
cg21129181	5	0.02310117	0.2379496	0.207793531	0.34874591
cg14706317	5	0.00109472	0.39662861	0.441563218	0.555632126
cg21297366	6	0.01825754	0.54759602	0.530196556	0.736324662
cg04206219	6	0.00663932	0.27125066	0.27530218	0.382264063
cg25460984	6	0.00141131	0.11529016	0.131757559	0.237233736
cg19265546	7	0.00092643	0.55506223	0.558989726	0.699436927
cg23879937	4	0.01465218	0.36174928	0.359820901	0.489438719
cg08488569	4	0.01372997	0.27126852	0.229367222	0.406925119
cg20387391	4	0.01891899	0.33058669	0.291727322	0.438545584
cg10310751	11	0.00649	0.61489414	0.65135645	0.75756236
cg05488279	17	0.04337605	0.32531199	0.337182448	0.444320827
cg07007213	11	0.00133856	0.67266847	0.68568898	0.78941966
cg10626792	11	0.00436875	0.39227216	0.42809167	0.565698232
cg18490269	7	0.00127427	0.760162	0.749336082	0.860892614
cg05006231	19	0.00133856	0.08361054	0.110854376	0.221817676
cg12030226	11	0.0103127	0.21108379	0.196774817	0.354769775
cg20741418	11	0.0106187	0.26478196	0.264918636	0.434649899
cg03858917	11	0.00210972	0.14038707	0.147255901	0.288591442
cg17223748	11	0.00480838	0.25880753	0.23779598	0.383583251
cg16858121	11	0.0023961	0.72318668	0.740733116	0.846487108
cg06258939	11	0.04698917	0.3800254	0.397457194	0.520652299
cg19290938	11	0.00141412	0.36341475	0.382590485	0.543530643
cg24465592	11	0.00756169	0.65751492	0.592530592	0.767844169
cg19425295	11	0.00165724	0.4296935	0.390262996	0.606643493
cg08637446	11	0.00155548	0.44135248	0.458400623	0.572329331
cg26343563	12	0.00218063	0.29332416	0.293124573	0.469872035
cg13821914	11	0.00866121	0.27551954	0.247127493	0.397631414
cg14310533	12	0.00519569	0.41893322	0.419668359	0.654438558
cg17164513	19	0.00218291	0.26817508	0.295899889	0.502071579
cg02263444	5	0.00494816	0.38290191	0.363293778	0.503198424
cg00574742	5	0.04382199	0.20338846	0.202254336	0.314053906
cg24709511	1	0.00637792	0.34906555	0.321182451	0.454269499
cg06250886	6	0.00701189	0.51831468	0.603947546	0.727415941
cg27416412	4	0.00277682	0.55511973	0.569877873	0.690224371
cg00259279	1	0.00589356	0.36252917	0.347423247	0.52063403
cg26195356	5	0.0023961	0.17541882	0.20078763	0.305346082
cg10809958	7	0.00796332	0.36796951	0.381489237	0.572910626
cg05123755	7	0.04220346	0.71827609	0.706812217	0.846289441
cg16252110	5	0.00314361	0.60861197	0.631821905	0.746368838
cg18718469	6	0.02653106	0.29145736	0.323852927	0.427089854

cg14508405	2	0.01109894	0.32146589	0.318098404	0.441252505
cg21964252	1	0.00991052	0.55886979	0.486053903	0.670522622
cg23226249	1	0.00453677	0.25416143	0.282962978	0.425028885
cg04229986	10	0.00420939	0.52295772	0.589326602	0.694056515
cg01269702	2	0.00237009	0.23690417	0.272852025	0.45598876
cg14595617	2	0.01019185	0.25961325	0.263427943	0.437790709
cg07628073	19	0.00149088	0.62625826	0.607713196	0.731923897
cg27070372	19	0.00478553	0.50208574	0.497888276	0.602663401
cg15516052	11	0.01378059	0.30115239	0.348004334	0.458693116
cg18384190	10	0.00490166	0.34896118	0.40060178	0.556678348
cg20244327	10	0.00280262	0.11619558	0.128214524	0.285502209
cg18054281	10	0.00411808	0.21737531	0.176374252	0.426520714
cg10492240	4	0.00184091	0.20764873	0.242378825	0.37952659
cg11256956	12	0.00708294	0.25435098	0.281119151	0.392396124
cg16063018	12	0.00943797	0.33911403	0.3717131	0.516371347
cg14099845	19	0.00548311	0.3576192	0.309485251	0.548447077
cg15197458	19	0.00491697	0.41678551	0.398823751	0.545289789
cg02859421	19	0.00602029	0.34330065	0.321777746	0.451630516
cg07482222	19	0.01957651	0.4826055	0.425122958	0.587138558
cg16377959	5	0.0086963	0.20879803	0.164156483	0.35228425
cg21240684	2	0.00587243	0.4712138	0.428443616	0.59555667
cg17855390	1	0.0063644	0.33038773	0.395875095	0.526217465
cg25367559	12	0.00705117	0.13958391	0.118561243	0.260784631
cg20594961	4	0.00429038	0.32386099	0.305723151	0.432668121
cg03222374	4	0.00457044	0.2953351	0.279553939	0.403993756
cg19480965	6	0.00201346	0.20330397	0.263579982	0.454863132
cg06144418	2	0.01111549	0.58787794	0.551335481	0.712406927
cg24221559	2	0.01463726	0.40955416	0.441085297	0.571647084
cg05562828	17	0.03934159	0.28565735	0.275980428	0.399554462
cg11204139	17	0.00130383	0.24779882	0.25787865	0.371330484
cg09695851	17	0.00088355	0.30181254	0.345801354	0.475400449
cg19504528	10	0.00099363	0.54371653	0.538967663	0.75387955
cg18400751	19	0.01236318	0.58193894	0.583828206	0.696181828
cg17729072	6	0.00287532	0.14344004	0.186313181	0.309051776
cg01385708	16	0.00263648	0.14092782	0.132951677	0.289507213
cg02574863	3	0.01021212	0.3968255	0.424221854	0.562631171
cg09697697	17	0.00466831	0.22953288	0.22728398	0.367204586
cg00349608	17	0.00306113	0.43030073	0.412908055	0.536282211
cg04492459	17	0.00287532	0.29544224	0.269974532	0.511990944
cg23678364	4	0.00104158	0.21499581	0.249041092	0.559893982
cg08653258	3	0.01499384	0.33351961	0.341425361	0.508910853
cg01414572	12	0.00403972	0.50123716	0.494230289	0.608831419
cg06876766	12	0.0167445	0.50540527	0.518006592	0.627577024
cg19993680	10	0.02496653	0.25003581	0.273060064	0.388218777
cg18239481	10	0.0007283	0.1776284	0.244629815	0.457979167
cg10410412	10	0.03931081	0.34469776	0.313964274	0.471911328

cg22321267	7	0.00287555	0.3597264	0.378240999	0.506310539
cg09879604	12	0.02394548	0.5894351	0.633743411	0.739550325
cg05881698	19	0.00362629	0.40054658	0.411465139	0.603611589
cg14390263	9	0.01665976	0.20357373	0.175188517	0.342405925
cg04705429	10	0.00658735	0.58576769	0.589922074	0.705752964
cg03504160	4	0.03160477	0.47318976	0.463144476	0.584572706
cg23418467	4	0.00140256	0.21894862	0.190037036	0.348609267
cg22521263	4	0.00083679	0.24425166	0.268848971	0.406179293
cg23612072	17	0.00364279	0.20642058	0.237005477	0.367576493
cg04536588	10	0.01629158	0.40123988	0.395381698	0.572540968
cg18986956	2	0.00574743	0.49675272	0.486293108	0.606861126
cg24067325	5	0.01861071	0.47917116	0.518502051	0.629143217
cg24384120	10	0.0012278	0.14367247	0.16527587	0.344054275
cg15741354	8	0.00849487	0.23173517	0.231473014	0.340841167
cg09225701	4	0.02003798	0.5437079	0.542826154	0.665682247
cg01962750	2	0.00311324	0.32634077	0.384779137	0.524524264
cg00562598	17	0.00233439	0.47125963	0.46399726	0.605975505
cg14191955	17	0.00144811	0.3702258	0.36635023	0.562996613
cg15406343	17	0.00427027	0.48120068	0.486820755	0.619861456
cg00061635	17	0.00230932	0.17773646	0.202220951	0.320361118
cg15629868	1	0.01040108	0.21000951	0.212942122	0.347864182
cg03874542	10	0.04648826	0.4840981	0.480689209	0.596052011
cg19209355	4	0.00206218	0.62769763	0.630189922	0.738490533
cg05295696	4	0.00463097	0.61892541	0.60397338	0.728155559
cg08490246	4	0.00735709	0.41886874	0.463534418	0.574626256
cg21545013	1	0.00218597	0.28949573	0.284464588	0.445885515
cg19704902	2	0.00130993	0.13148829	0.126418521	0.307524569
cg08786370	4	0.00256449	0.63753091	0.693078582	0.842659079
cg04361019	2	0.00238047	0.16955333	0.186447325	0.37079728
cg06002476	3	0.00230737	0.27119319	0.32780231	0.478081494
cg01099150	2	0.00377723	0.14721271	0.15566699	0.361315759
cg00782839	2	0.00670577	0.23768047	0.267271348	0.455594627
cg12204974	5	0.00444287	0.43175191	0.435969018	0.610833142
cg08127108	2	0.00243597	0.29472264	0.312669994	0.518439711
cg27230594	1	0.00244246	0.27115382	0.263685996	0.420311562
cg01550810	1	0.00664725	0.55117538	0.594616929	0.741686264
cg16455376	16	0.0020588	0.19718695	0.193229077	0.361693553
cg24537275	17	0.00672923	0.5497258	0.486098195	0.657756987
cg08787698	2	0.00485896	0.34606985	0.374672056	0.577250505
cg02525997	8	0.00441677	0.13862881	0.139918468	0.245586542
cg23606162	1	0.00211366	0.53590958	0.576717308	0.69513954
cg08089449	11	0.00845805	0.72758017	0.727686044	0.838670445
cg10992465	1	0.00485896	0.19238123	0.151747712	0.335315022
cg24683121	2	0.00811207	0.66304746	0.617790691	0.821290975
cg05804846	11	0.0019906	0.16290469	0.161320756	0.349177977
cg19988235	2	0.00814151	0.3085593	0.314633891	0.456307957

cg16937583	3	0.00629918	0.34980376	0.345973595	0.508264989
cg06657240	18	0.00118031	0.4934509	0.525993958	0.645836453
cg16588492	12	0.00997527	0.36519201	0.329117711	0.506960644
cg14359798	11	0.00074016	0.21451207	0.399105514	0.603916544
cg25338818	18	0.00347082	0.49045421	0.489418489	0.623140253
cg24998791	8	0.00853827	0.21277654	0.207749399	0.335290116
cg16362189	11	0.00168532	0.28730868	0.298779193	0.573669861
cg12499062	18	0.00342611	0.30030029	0.281456344	0.440703726
cg01774019	4	0.03753328	0.37064161	0.357093933	0.476521052
cg01413548	6	0.00144359	0.31872438	0.312448769	0.52628765
cg26159128	4	0.0190357	0.19003307	0.150055685	0.302718333
cg00594191	10	0.00090002	0.14349402	0.168235838	0.33682287
cg08422172	16	0.00457097	0.50418149	0.511392398	0.616854256
cg22004089	2	0.00197986	0.62195114	0.629133825	0.759682255
cg20462100	1	0.00622936	0.50310844	0.52437493	0.651269865
cg19771350	11	0.014833	0.48378997	0.481256999	0.658102702
cg13866214	18	0.00283173	0.11687928	0.117445067	0.217725196
cg19072083	2	0.02448461	0.23155591	0.267624415	0.373202131
cg15135803	1	0.01366893	0.1720758	0.121496689	0.272471734
cg07230078	4	0.0011104	0.22243529	0.251062374	0.439909342
cg18384893	17	0.04879812	0.52239996	0.515296006	0.632701741
cg26632875	12	0.00628685	0.10918841	0.122388606	0.242212791
cg27511461	10	0.00126059	0.47031996	0.484491382	0.682661594
cg21776813	3	0.00667841	0.24683977	0.231034708	0.368409684
cg13824456	7	0.01006689	0.67491481	0.677106699	0.884612022
cg08879390	19	0.00383289	0.41295774	0.431770003	0.56886712
cg19499614	17	0.00459107	0.17739098	0.172093391	0.328474648
cg08301211	6	0.00114928	0.48318604	0.553057068	0.68778435
cg09425032	19	0.00193378	0.32170443	0.259419257	0.459998778
cg12871937	3	0.00338706	0.4749399	0.5076618	0.609780026
cg12146763	17	0.00774063	0.22984036	0.169956099	0.336301367
cg09148630	6	0.00119376	0.17810445	0.192798239	0.383501511
cg09571082	6	0.00911882	0.25481651	0.221251013	0.364013363
cg24219974	6	0.00146929	0.44257852	0.4878359	0.591073603
cg09858767	1	0.0084915	0.13845506	0.14413559	0.252806453
cg01289290	3	0.00111524	0.58811877	0.651160185	0.76059373
cg02852847	1	0.00173648	0.5423785	0.570098049	0.706139124
cg00366236	17	0.01093065	0.39569473	0.412723948	0.561331852
cg23429664	10	0.00111281	0.28958907	0.379024176	0.630486069
cg16426034	11	0.00158407	0.20262815	0.247425279	0.463216451
cg23332827	11	0.00404842	0.30812437	0.308233574	0.460671213
cg06256007	11	0.00810421	0.22629149	0.199201736	0.327678936
cg06359632	11	0.04390149	0.49682857	0.510804223	0.638222816
cg03624438	3	0.01980822	0.36846482	0.303004378	0.486211193
cg11804725	3	0.00225589	0.1012042	0.101280323	0.242208665
cg22542685	17	0.0147494	0.63065291	0.640876685	0.760378625

cg09011316	1	0.00140256	0.37955578	0.43214833	0.546889271
cg22251148	11	0.01580869	0.21240486	0.219467946	0.35275214
cg20101110	11	0.00401281	0.23080982	0.282926239	0.46767112
cg10969209	1	0.04648826	0.20265509	0.198077334	0.304585667
cg01872122	5	0.00753326	0.24671509	0.226696354	0.35901918
cg19516754	6	0.00219711	0.20712137	0.201313899	0.40548259
cg15842898	4	0.04768316	0.23588935	0.234105691	0.363454375
cg18869244	17	0.00128133	0.17979844	0.192922859	0.407651568
cg17419514	7	0.00595346	0.63376514	0.603853479	0.793770371
cg03291548	22	0.00538453	0.37501836	0.38285809	0.519183293
cg17209471	22	0.0025393	0.26677778	0.312292524	0.547364837
cg12656424	2	0.00150566	0.62608679	0.663720277	0.770256203
cg17882587	2	0.00856489	0.15292784	0.143576932	0.283716068
cg10244770	12	0.02069475	0.46239689	0.458740892	0.589846401
cg09936919	2	0.00173154	0.18488351	0.181957168	0.480724825
cg01230988	17	0.03270435	0.25264546	0.226346606	0.381495383
cg06720014	1	0.00372456	0.36099129	0.412486362	0.57816418
cg07584582	22	0.00146177	0.26559422	0.288094096	0.516072958
cg08866876	8	0.01309134	0.23323598	0.201631284	0.416970074
cg23291376	2	0.01183547	0.45698254	0.484561473	0.612735647
cg00830419	2	0.00521564	0.6330714	0.65394323	0.778330069
cg09139491	13	0.01206219	0.44752931	0.477105857	0.593467567
cg18899220	13	0.01666517	0.43966963	0.443868645	0.547571351
cg08264835	1	0.00634238	0.42583966	0.439439801	0.583816802
cg25430713	2	0.00958999	0.24975381	0.222579616	0.377056451
cg23992493	17	0.00276938	0.38660464	0.328328612	0.514265221
cg08392142	13	0.03166005	0.28552044	0.266585441	0.394339007
cg17404640	6	0.01446513	0.40203729	0.455792332	0.572812123
cg02451502	13	0.00526183	0.2212353	0.187724769	0.334699045
cg00792968	13	0.00195	0.3772175	0.446234516	0.633067445
cg11315081	13	0.00295256	0.70054206	0.721964749	0.826542563
cg14863944	13	0.00331728	0.67819575	0.712847001	0.82748659
cg02067239	16	0.00377723	0.21937785	0.248014433	0.460099031
cg19994159	13	0.00576931	0.30846804	0.33711173	0.480168248
cg06711873	19	0.00392454	0.15306269	0.13592347	0.258165639
cg23864514	7	0.01178392	0.40075427	0.377395571	0.603144277
cg20073893	14	0.00107365	0.32851813	0.409064078	0.558525635
cg23117727	20	0.00658735	0.64355192	0.648656863	0.764820064
cg08026493	8	0.00162163	0.33419806	0.372037043	0.537478254
cg20845639	8	0.00233734	0.22171225	0.217336949	0.333786943
cg19577365	14	0.00345706	0.3522233	0.37566122	0.496825688
cg12646386	1	0.01320626	0.42626109	0.469972397	0.591575958
cg00005740	13	0.00682394	0.36269335	0.369141584	0.49956654
cg05801374	1	0.00123203	0.33014352	0.388677076	0.494569306
cg05210480	7	0.00345706	0.2614354	0.288589045	0.42819091
cg00491577	2	0.01154279	0.27881634	0.246157986	0.459398315

cg06151171	2	0.00679225	0.23853905	0.247528367	0.43383327
cg03160656	4	0.01810942	0.37446587	0.454573569	0.59416763
cg22183373	4	0.02389893	0.51718487	0.503683193	0.674169345
cg02924972	13	0.0252514	0.5614878	0.572342751	0.703729401
cg25772185	10	0.00388546	0.1785362	0.175222825	0.361197987
cg02681163	8	0.00715821	0.35040802	0.377317651	0.535350885
cg17590003	17	0.00391578	0.21566277	0.22494664	0.344157418
cg21897970	17	0.00327239	0.31219163	0.353992467	0.481789146
cg08549390	7	0.00092985	0.1263993	0.1654345	0.368179536
cg09501314	15	0.00107365	0.14402489	0.175397773	0.344571884
cg24985060	15	0.00472614	0.21270036	0.151986789	0.431355117
cg12315995	15	0.00439411	0.20875815	0.146689034	0.37783784
cg27574223	12	0.00230737	0.17849851	0.202297383	0.310301862
cg03665255	6	0.01773077	0.29808169	0.248067319	0.419677568
cg21917512	2	0.02078916	0.22966758	0.222850595	0.403519049
cg08243094	1	0.01543666	0.40404154	0.426844805	0.574440601
cg18985738	11	0.00302287	0.13681517	0.165586496	0.320104539
cg15741935	2	0.00166335	0.31196126	0.341357343	0.446836605
cg17431023	7	0.00638555	0.27147051	0.27553442	0.396196559
cg09976157	6	0.02172582	0.28344783	0.365100463	0.46746487
cg24527636	1	0.00468549	0.19847491	0.22394322	0.375521993
cg03413253	12	0.00296353	0.60038136	0.52683832	0.713032114
cg10143823	14	0.00224923	0.31286763	0.304897313	0.564313124
cg08029969	16	0.00613201	0.32924567	0.307286846	0.448593897
cg02553872	16	0.00806395	0.33089573	0.331721436	0.467556891
cg22609511	7	0.00935896	0.20568672	0.248601002	0.348601607
cg02621030	16	0.01237003	0.64727601	0.685580883	0.808638737
cg09039112	16	0.00453677	0.6717947	0.688598085	0.804786121
cg16528596	16	0.00177922	0.25494905	0.258676986	0.407373574
cg25927044	10	0.01021297	0.2665815	0.283087766	0.391347898
cg10484693	10	0.00112235	0.18382305	0.192475362	0.383250694
cg04649852	16	0.01585745	0.28172752	0.248560368	0.38774794
cg09007841	16	0.00463443	0.28363111	0.240943601	0.416688977
cg09345153	16	0.0157424	0.23398225	0.229205436	0.341144921
cg09320079	16	0.00522751	0.16570454	0.193734434	0.356788497
cg07356338	16	0.00461358	0.34791015	0.371210911	0.495784544
cg27438754	16	0.01332867	0.24523075	0.267018736	0.450023444
cg22840887	10	0.0051502	0.16836	0.15196236	0.373706782
cg00286119	8	0.01888833	0.24673343	0.228885065	0.376822809
cg03147324	15	0.00314981	0.23657068	0.240765219	0.351828394
cg13018639	14	0.03219604	0.5819932	0.495587287	0.683325442
cg23375895	6	0.01585898	0.42579864	0.451533552	0.556967107
cg24335092	3	0.01580766	0.20876445	0.189105698	0.332514499
cg05098589	10	0.00073118	0.54254548	0.626218338	0.791241521
cg19667811	10	0.00117377	0.08395261	0.086797146	0.206067174
cg10533060	16	0.0012026	0.67095553	0.693333023	0.8291932

cg04935436	20	0.00491882	0.19455873	0.194541403	0.315970011
cg16444826	2	0.00556972	0.22350361	0.214376175	0.415536465
cg24167667	3	0.02935375	0.20318096	0.241453627	0.380404472
cg22282089	13	0.02172582	0.41833097	0.361386734	0.558464371
cg21249595	6	0.00263536	0.53607282	0.53398085	0.642627567
cg06369155	13	0.02817047	0.51895201	0.557715971	0.679954003
cg02264079	16	0.0071227	0.34753198	0.357332285	0.476560385
cg04702989	16	0.03174689	0.395375	0.407999697	0.536783708
cg05928033	1	0.00401094	0.26767707	0.232273039	0.394702876
cg24366665	13	0.00669541	0.52288397	0.519597159	0.649418788
cg17382879	13	0.00371278	0.51695229	0.534438836	0.669422881
cg19735151	10	0.00509941	0.41588172	0.45841847	0.600655116
cg11703759	1	0.02641471	0.66613386	0.656471491	0.766790755
cg03727169	10	0.00134295	0.29672992	0.311685243	0.42888928
cg04589649	2	0.00126466	0.24422511	0.283361117	0.600695743
cg25417675	6	0.00670317	0.19744716	0.185203281	0.304273433
cg15417287	12	0.00375959	0.19360846	0.155402818	0.303066592
cg17082026	13	0.00428303	0.39934113	0.474950265	0.621538489
cg09502809	1	0.00127413	0.47293888	0.491729113	0.622468946
cg16311740	3	0.00068904	0.30206101	0.357458347	0.503951637
cg22593432	13	0.01643588	0.15283162	0.163079855	0.2936974
cg23027179	5	0.00412029	0.27964724	0.224724255	0.465693939
cg01481251	11	0.04650849	0.32945765	0.333494849	0.460604705
cg09548241	1	0.00308097	0.14326452	0.129204903	0.291909789
cg26070099	1	0.01398927	0.35836567	0.302749901	0.494663226
cg22501483	7	0.0190357	0.37251677	0.364461815	0.517852021
cg16522412	13	0.00643366	0.12101264	0.116947502	0.243648138
cg14299572	10	0.00092259	0.16345103	0.18369693	0.303213527
cg00527307	13	0.01354962	0.62167995	0.598016938	0.741524739
cg03741824	21	0.00820352	0.31938201	0.325972265	0.437385451
cg18833994	5	0.00532167	0.32107854	0.353451348	0.504763095
cg12370929	9	0.01748472	0.20821117	0.196665599	0.309592262
cg07220149	17	0.02638649	0.3074858	0.268970041	0.428126981
cg17540450	13	0.00087274	0.2080098	0.312278242	0.471452548
cg06228857	15	0.00896145	0.31825568	0.346524949	0.541479063
cg26107642	5	0.00620321	0.23266933	0.271592015	0.419913252
cg12784272	17	0.01997904	0.25955365	0.248585841	0.367848218
cg05686379	7	0.01015703	0.2445814	0.201404222	0.40197922
cg27022420	17	0.00112849	0.5151692	0.528612216	0.680484663
cg26891237	6	0.00246645	0.14479108	0.13478547	0.348628858
cg20098468	8	0.01870855	0.27669359	0.26225169	0.432585426
cg11202634	8	0.00715821	0.44951215	0.42833853	0.55461393
cg19067025	15	0.0011571	0.29421181	0.389245587	0.596480567
cg22286764	3	0.00186737	0.16326023	0.168181409	0.278700418
cg04245294	3	0.00517777	0.37683453	0.382337822	0.570505793
cg11466131	22	0.02049456	0.56494494	0.533905205	0.717275297

cg25695992	2	0.00170239	0.43298044	0.528646633	0.697310783
cg01021196	14	0.03305066	0.24007279	0.213951384	0.349042857
cg18451016	1	0.00447688	0.17642854	0.130563554	0.290620537
cg06444374	1	0.00816896	0.62434736	0.617488857	0.728466776
cg04848137	8	0.01317683	0.2047205	0.185251242	0.326159516
cg26068090	13	0.01896129	0.4066343	0.455133725	0.571560907
cg20684375	8	0.04645191	0.23149576	0.24065344	0.349923841
cg26241893	5	0.00220384	0.46249575	0.374662758	0.613659101
cg23165992	5	0.04406604	0.583657	0.593473306	0.696437786
cg25943481	15	0.001879	0.35030903	0.365461214	0.551330384
cg14410733	15	0.001223	0.27850995	0.294865675	0.395324798
cg00270974	6	0.00953692	0.18956428	0.153170296	0.362561279
cg27657363	22	0.00349377	0.28298431	0.322175235	0.498474099
cg00618626	17	0.00306113	0.40465728	0.407312257	0.528524086
cg21879236	5	0.00705117	0.18043076	0.162325176	0.370399185
cg07849237	17	0.00682394	0.6364198	0.641010625	0.76665611
cg26884359	17	0.00113577	0.16855191	0.189025792	0.379843873
cg14482712	15	0.00067859	0.26037042	0.319944581	0.481369523
cg23114476	5	0.00208745	0.28077893	0.261649184	0.528494922
cg22615203	13	0.00098825	0.24710673	0.321656778	0.555276925
cg20886660	13	0.00318264	0.44314122	0.523706364	0.653024364
cg04754683	8	0.01718572	0.47607379	0.516203783	0.659971614
cg15305352	6	0.00238378	0.17287471	0.212970768	0.395275171
cg14732815	6	0.01980843	0.23492308	0.208427371	0.351368946
cg08244518	22	0.00168532	0.47566604	0.515298585	0.621874387
cg08037719	1	0.0012838	0.35942643	0.451790197	0.63003214
cg06238004	17	0.00766486	0.26379459	0.203518701	0.379393426
cg03656290	4	0.00515785	0.19193221	0.183748799	0.345054395
cg04495313	4	0.00591291	0.2201483	0.218124694	0.334414712
cg08357895	5	0.0020562	0.19185518	0.198726483	0.448062599
cg09772333	19	0.01905936	0.31451103	0.280144962	0.433347798
cg04570228	13	0.02768694	0.34921559	0.355327771	0.461757003
cg06815361	8	0.01715886	0.49226186	0.470419454	0.602180628
cg23201188	3	0.0033048	0.27109289	0.365978721	0.516907303
cg01817364	5	0.03488234	0.34143093	0.327253389	0.447542316
cg17427675	5	0.00548794	0.42662272	0.43266535	0.612490469
cg05422883	2	0.00295256	0.53385316	0.529841207	0.66510315
cg15360478	2	0.01398104	0.2698581	0.238078916	0.404570599
cg13215995	17	0.00241305	0.24743249	0.230824377	0.359641111
cg07621853	2	0.00077582	0.53441017	0.569475749	0.790383857
cg05672223	2	0.00208621	0.3296455	0.307057941	0.443884683
cg14706297	2	0.00233439	0.18658096	0.132746184	0.29436026
cg00909401	2	0.00103447	0.48567497	0.558909558	0.710819517
cg01243072	2	0.00106261	0.22791513	0.306248387	0.421544282
cg05753629	1	0.00275824	0.3814694	0.428825901	0.592376186
cg01403655	13	0.02360575	0.61101881	0.605964411	0.717691621

cg01019249	5	0.0016453	0.6110813	0.61571279	0.721739161
cg27360992	22	0.00298303	0.22212608	0.243915047	0.453384674
cg15526213	10	0.0074197	0.47762623	0.464030629	0.59143264
cg07095818	20	0.00367708	0.18816495	0.188006499	0.360767122
cg04609694	6	0.00563553	0.23498212	0.253499723	0.411864226
cg05215056	10	0.01311902	0.3961423	0.410411606	0.529530149
cg17504641	10	0.00193378	0.31986154	0.321915895	0.481562839
cg01019484	6	0.02938665	0.28981853	0.291041062	0.391445957
cg22757418	6	0.00168741	0.6251986	0.672912473	0.773310592
cg06976222	1	0.02106374	0.48722021	0.589199571	0.707359199
cg07099073	6	0.00180881	0.58908603	0.667162799	0.807325062
cg04809712	7	0.00535203	0.63706634	0.681841545	0.785131509
cg03155825	7	0.00174783	0.22642161	0.262965331	0.478937441
cg22991039	21	0.00252735	0.39415143	0.348541722	0.563673399
cg04914930	13	0.00323448	0.16758746	0.180653167	0.341864316
cg26703511	21	0.00312811	0.34940758	0.357778449	0.520420617
cg14223001	11	0.01009622	0.2123712	0.184274519	0.320285887
cg00615941	3	0.00315582	0.39559375	0.426795771	0.591104549
cg01369207	2	0.00713358	0.48304655	0.458289694	0.600380222
cg02093902	2	0.00539451	0.11780223	0.110790725	0.227142677
cg15765353	3	0.04760642	0.29186795	0.342081604	0.468440374
cg18749015	6	0.00071094	0.08927287	0.138340402	0.293333721
cg12019475	6	0.0026964	0.18251323	0.179221656	0.383051387
cg00348891	13	0.00806542	0.23246557	0.218282751	0.354032114
cg25668878	3	0.00192049	0.57168605	0.593269169	0.702574536
cg00490603	22	0.00274286	0.15765094	0.151686488	0.265681708
cg11378001	21	0.00174311	0.26545838	0.235858256	0.429040254
cg24489344	22	0.00280852	0.36752552	0.367857871	0.489363706
cg03343669	2	0.00663932	0.27792985	0.27638982	0.445239187
cg07704235	18	0.00073118	0.54718578	0.658694328	0.772984542
cg15294263	21	0.00248015	0.29988988	0.268167229	0.41655815
cg09604238	12	0.00845805	0.52752692	0.525868545	0.628761724
cg06350882	7	0.00912728	0.51138275	0.500522778	0.63729728
cg13554773	17	0.0373326	0.48770992	0.519778518	0.648586248
cg14327359	2	0.00733126	0.35521995	0.367759553	0.52662344
cg18064917	2	0.00245414	0.3563401	0.451862431	0.606010572
cg13604150	21	0.00470488	0.63919241	0.670675358	0.808156357
cg00129785	21	0.02241528	0.47308068	0.508567613	0.617811259
cg22964758	12	0.00752854	0.544816	0.593369705	0.709249789
cg25073998	2	0.00551198	0.12929663	0.124462277	0.241370501
cg23101632	2	0.0103161	0.14655506	0.127276138	0.276515279
cg00092565	21	0.00626821	0.21071479	0.196670555	0.390282146
cg16425161	15	0.03853559	0.39308096	0.340357029	0.501910544
cg20430104	7	0.00964959	0.45620912	0.476261633	0.593136053
cg02518338	17	0.0101333	0.40980788	0.3256401	0.520754944
cg05310240	17	0.00908203	0.42384049	0.453793699	0.559126798

cg18553570	12	0.01289795	0.18195103	0.15913309	0.326619405
cg06212297	16	0.00066232	0.45652569	0.553931805	0.670773798
cg02411088	17	0.00152273	0.11696478	0.1253447	0.256690781
cg16401668	1	0.04452792	0.31528681	0.316358678	0.438595054
cg02470587	12	0.00851187	0.1313798	0.104478734	0.263121277
cg22919538	3	0.00181386	0.54833371	0.573439315	0.724400527
cg19513492	12	0.00177922	0.23051534	0.264856345	0.398852098
cg20597127	1	0.00218291	0.23192184	0.257240501	0.444256232
cg03810376	1	0.00088355	0.14073679	0.158815176	0.31636661
cg06260709	20	0.00542561	0.22218978	0.205038639	0.344142351
cg06801658	20	0.02658897	0.45278696	0.426366411	0.606755744
cg09814448	1	0.02389893	0.36805965	0.338572703	0.507988225
cg17152266	19	0.00441677	0.50934814	0.543412261	0.646144689
cg01898377	20	0.00248015	0.31991222	0.309108256	0.5503871
cg05765734	8	0.00694471	0.20105781	0.163914713	0.301488996
cg20384325	15	0.00101423	0.60862544	0.574987506	0.712445269
cg26702098	8	0.00354346	0.37104138	0.409908421	0.571260826
cg16322269	8	0.00100059	0.42462566	0.454608984	0.610177132
cg21463446	8	0.00419158	0.48830273	0.510584387	0.683595449
cg15257489	8	0.00083228	0.17150237	0.217734144	0.444980297
cg24183575	3	0.00129758	0.41024217	0.437869558	0.544540056
cg17516571	8	0.01183117	0.25898682	0.273044489	0.431054362
cg06034711	8	0.00072814	0.53380174	0.593141691	0.695474364
cg01502811	16	0.00092643	0.54861809	0.539514032	0.743283511
cg26811425	16	0.00364379	0.2206755	0.165357052	0.336497659
cg25920857	3	0.01278326	0.43446987	0.398716413	0.546622133
cg03295274	13	0.03431393	0.10287231	0.098698216	0.203430734
cg24800883	5	0.01010425	0.24371261	0.282868236	0.435951175
cg19885001	13	0.01390151	0.46370829	0.496169209	0.624090714
cg00465247	13	0.01244196	0.3284284	0.34553967	0.460893793
cg17388712	7	0.01776318	0.24228179	0.247826681	0.37601654
cg22143274	5	0.01430511	0.47390645	0.449870248	0.606619206
cg04251886	14	0.01356792	0.52684433	0.459804993	0.632004491
cg11807040	13	0.01158752	0.43588755	0.454985623	0.572708164
cg05777602	16	0.01910705	0.55675028	0.547073527	0.701103554
cg24352299	13	0.00642316	0.55587476	0.615432248	0.723635008
cg00750630	14	0.00164959	0.46992735	0.564611066	0.679681866
cg01265068	13	0.04581055	0.72730854	0.738740497	0.847915389
cg05261299	12	0.0012838	0.25479692	0.326637061	0.439323715
cg25351353	12	0.00913085	0.44332378	0.439345686	0.558280687
cg17485988	7	0.0012026	0.27093099	0.29078954	0.502830282
cg05382185	6	0.02999866	0.4671203	0.454311503	0.627634914
cg00491530	12	0.00336153	0.46979595	0.46040959	0.609515486
cg11936809	8	0.0094452	0.34897933	0.363814165	0.528119646
cg05051799	6	0.03525194	0.21549444	0.21601509	0.384745226
cg11841288	1	0.01630619	0.53684499	0.558065103	0.693434413

cg26785370	17	0.004607	0.32121699	0.343553015	0.495964664
cg24543939	5	0.02970062	0.1744583	0.151309458	0.28823503
cg17091056	2	0.00931986	0.22453635	0.226304039	0.409869496
cg05138082	14	0.02362077	0.41463957	0.408994212	0.517737909
cg03221837	16	0.00237687	0.29253272	0.321641952	0.483484436
cg14833626	4	0.00081789	0.21937464	0.307684231	0.502186533
cg22115196	4	0.01463726	0.4429042	0.470977159	0.580522278
cg09290893	17	0.00337574	0.27268679	0.271498471	0.407318413
cg16278514	3	0.0013661	0.26888074	0.339039952	0.464089027
cg17073708	8	0.0347271	0.38831943	0.394968029	0.504971958
cg25249728	2	0.00162163	0.14472551	0.173979055	0.347486058
cg07006398	16	0.01289795	0.48673777	0.519884557	0.623698709
cg04013650	8	0.00830789	0.57704048	0.613531647	0.713927513
cg22680536	4	0.00646891	0.26533054	0.237871856	0.41324433
cg10783149	14	0.01446542	0.31268167	0.261402617	0.420244376
cg06443678	17	0.00315576	0.47973411	0.532628705	0.649857227
cg19595295	17	0.0011206	0.22100201	0.234304237	0.435689047
cg13767779	17	0.00152273	0.39384749	0.457522642	0.589930976
cg13212831	1	0.01998122	0.62494724	0.614946464	0.747325548
cg19241927	1	0.02634735	0.31310794	0.333213326	0.526250593
cg01905690	14	0.00716181	0.23010422	0.200017222	0.339884572
cg14377849	1	0.01164124	0.23559869	0.168601021	0.361340712
cg11818544	5	0.01539932	0.4480764	0.49047118	0.620025806
cg14883605	14	0.02317852	0.28743361	0.278677156	0.421963162
cg17011134	1	0.0010058	0.58195915	0.596942001	0.708317386
cg11738198	1	0.00283456	0.18921517	0.224525257	0.346619522
cg13482375	17	0.00457097	0.21225778	0.199134855	0.345135081
cg24741744	18	0.00186737	0.26747747	0.260511277	0.474002521
cg12741572	18	0.00168656	0.31662693	0.312452339	0.536387684
cg22300615	20	0.03488893	0.36312752	0.374253216	0.504085276
cg17074989	15	0.00524189	0.21571116	0.2872982	0.412949757
cg17479898	1	0.00509941	0.43809451	0.494021756	0.631266899
cg13494055	3	0.00263491	0.60104182	0.624231941	0.738423775
cg14244013	17	0.00066232	0.15840294	0.210429426	0.315326974
cg09767588	1	0.00117894	0.26838671	0.331619049	0.580344545
cg17139283	1	0.00219711	0.70717202	0.719044062	0.840784604
cg07579884	14	0.00403008	0.32109594	0.350356752	0.504474492
cg09798077	5	0.00569156	0.5199839	0.550803053	0.656317796
cg11254532	1	0.0011104	0.26460429	0.30267017	0.448855862
cg25769127	3	0.00142756	0.33782402	0.347578637	0.509427641
cg09213451	14	0.00217555	0.43808296	0.476627624	0.588403699
cg03753942	8	0.0283256	0.39664952	0.384062686	0.509687584
cg22968789	15	0.00323448	0.21893335	0.22015848	0.400683397
cg05917797	14	0.00263954	0.12269625	0.093268055	0.265407213
cg24690715	20	0.00725225	0.32308595	0.350009854	0.507198484
cg05599759	20	0.00113577	0.24147242	0.253970075	0.441281022

cg23819144	10	0.00197515	0.55879359	0.592929669	0.732241661
cg13385601	15	0.01980843	0.20465317	0.173418994	0.339565216
cg27251686	2	0.02577962	0.62483769	0.623124712	0.734193365
cg26987928	15	0.00116059	0.55025976	0.616040463	0.743253442
cg01744607	15	0.00438281	0.3351164	0.433171156	0.543383491
cg15851389	15	0.00116059	0.60617136	0.644961868	0.758708923
cg05276184	15	0.00380612	0.14511267	0.141442824	0.33132993
cg02243157	2	0.01783758	0.47816753	0.497004052	0.640693887
cg09802893	7	0.0080143	0.63312823	0.648031557	0.762231307
cg16993013	15	0.00670577	0.65938426	0.664382146	0.770074834
cg16572600	11	0.00233439	0.26122133	0.242628482	0.445270918
cg26097711	15	0.01831034	0.18555819	0.170445569	0.313924665
cg18528696	12	0.04062029	0.39396407	0.36052605	0.494211469
cg00847221	17	0.03075767	0.35417698	0.377282494	0.563884944
cg05339976	2	0.00629918	0.34295128	0.326076915	0.473772316
cg00997998	14	0.00292358	0.65686439	0.620912445	0.77778132
cg11746048	11	0.00168532	0.26904482	0.328469227	0.572954347
cg01292856	11	0.01164121	0.60438074	0.595921126	0.714027682
cg16175245	11	0.01378915	0.194356	0.193799469	0.306566268
cg20743628	7	0.02457877	0.24223176	0.243468005	0.399010026
cg00978505	1	0.00291636	0.49831432	0.526967371	0.647921023
cg17010895	2	0.01746225	0.4720955	0.544561107	0.655735164
cg03995968	16	0.04406604	0.31387169	0.300979548	0.418456328
cg12905176	3	0.0111106	0.34365873	0.3553207	0.487430704
cg03116409	12	0.01482933	0.36813724	0.390114049	0.530461444
cg03819089	2	0.03227846	0.46469549	0.445591868	0.625735885
cg22375757	3	0.00129968	0.35771709	0.395922997	0.581019698
cg27133959	3	0.04936492	0.44250496	0.354957871	0.543009759
cg19967176	15	0.00223213	0.53199748	0.548480277	0.659127534
cg07909444	17	0.00883762	0.45827609	0.401439099	0.562615967
cg16627836	2	0.04062029	0.30574917	0.29216356	0.41926218
cg11680908	5	0.00155548	0.70319469	0.675724915	0.841634831
cg26893699	17	0.01620455	0.29480013	0.362274654	0.471259707
cg14574066	11	0.00106261	0.16928096	0.18344088	0.334629171
cg09743615	11	0.00294138	0.5990303	0.617240313	0.736970104
cg15857051	14	0.00225672	0.16458363	0.164281373	0.379280689
cg02645287	16	0.00209829	0.51758266	0.506408454	0.633483162
cg06597413	11	0.00068904	0.33018711	0.406415793	0.541746259
cg26290155	14	0.01009664	0.16970586	0.175246714	0.310033722
cg02116942	2	0.01980843	0.1437215	0.115342702	0.245509983
cg01152374	15	0.00367708	0.16445996	0.180904344	0.303995844
cg03025413	8	0.01034491	0.5730842	0.46523865	0.677323912
cg04852323	16	0.00195644	0.42555466	0.451348423	0.607438785
cg00549064	16	0.00108543	0.10341203	0.111215628	0.279111166
cg14445366	17	0.00073547	0.19688258	0.318368584	0.460222429
cg13544294	17	0.00247655	0.18696263	0.189027364	0.293939169

cg02274236	7	0.00179679	0.18427087	0.19639796	0.418882631
cg02327773	7	0.0039229	0.14678308	0.151779935	0.327544442
cg18542967	7	0.00702785	0.14200924	0.156809395	0.313000133
cg14498227	11	0.00147559	0.26529665	0.306489944	0.413341694
cg14041701	11	0.00197986	0.26291989	0.259057263	0.50793335
cg20933897	17	0.00483111	0.41387375	0.503517001	0.610161698
cg19051238	12	0.04237386	0.23221118	0.233303939	0.372264816
cg14125530	12	0.00621701	0.20654069	0.174311902	0.322787489
cg04147969	15	0.00075518	0.08501659	0.112140108	0.244071093
cg09256716	17	0.03221861	0.17225495	0.134012635	0.295963172
cg12109883	15	0.00218429	0.17920626	0.221367841	0.404578516
cg26848957	17	0.0070644	0.41331063	0.531487013	0.637356106
cg23681616	17	0.00126804	0.51535499	0.597120414	0.747975092
cg19298400	17	0.00174721	0.38048525	0.409005119	0.550866267
cg24930059	17	0.012575	0.21983916	0.18881042	0.35164115
cg05565697	15	0.00088355	0.41263133	0.461873642	0.597568669
cg05199904	5	0.00159552	0.14997685	0.148444074	0.311256338
cg04525683	14	0.04382199	0.4840153	0.486556031	0.596114107
cg27540038	15	0.02101935	0.29885762	0.32237917	0.432332116
cg11877773	14	0.00237508	0.42483756	0.445240827	0.557553174
cg00196474	3	0.01411406	0.17862588	0.14148615	0.335076251
cg21470704	2	0.00211366	0.28426319	0.347638873	0.513694864
cg24516106	2	0.00318676	0.16514459	0.182302554	0.333991037
cg22163280	17	0.00569156	0.16921188	0.168164409	0.290575623
cg00051307	5	0.04052436	0.44916006	0.416234229	0.566270259
cg01454395	17	0.00355472	0.19487965	0.194196394	0.30901924
cg12809483	5	0.00168532	0.29134521	0.357839145	0.553838972
cg11423680	5	0.00079224	0.13598831	0.154701723	0.29206955
cg05155038	5	0.00233734	0.49458121	0.523325809	0.659215659
cg25889504	7	0.02473841	0.39247261	0.397135933	0.532092881
cg14297340	9	0.0412314	0.20478304	0.16735083	0.324359282
cg19502359	11	0.00074381	0.1496229	0.19916248	0.302703881
cg12741293	7	0.00405622	0.12784733	0.125681683	0.250801944
cg17500962	16	0.00240097	0.52925229	0.553862991	0.690322356
cg08818377	7	0.00113577	0.33946201	0.32856838	0.543687432
cg00305740	7	0.00852172	0.32672517	0.340321098	0.45962169
cg27298940	7	0.00843889	0.29534308	0.290288406	0.404307137
cg26611650	16	0.01482933	0.41557745	0.416449825	0.555931158
cg23844085	18	0.00648185	0.2517091	0.263852079	0.417160728
cg23096806	10	0.00165724	0.24658466	0.300148538	0.426475246
cg10924875	8	0.00675316	0.24563663	0.222731127	0.347380445
cg25149122	18	0.00079512	0.29752271	0.349281524	0.509465786
cg27421117	2	0.00725763	0.54295083	0.535674781	0.661464599
cg03457984	2	0.00293735	0.4969862	0.524284732	0.625773505
cg03742250	17	0.01896129	0.32807593	0.270814413	0.429938515
cg04524475	13	0.00973492	0.34490684	0.31731961	0.507499616

cg21059878	4	0.00128133	0.49635389	0.420460839	0.656992767
cg10020890	16	0.00126059	0.39499464	0.449334703	0.563034779
cg01555052	14	0.02108987	0.48853806	0.529398917	0.635814124
cg14886850	14	0.02214521	0.22897801	0.277050995	0.381051324
cg10031989	16	0.00745134	0.21025844	0.1931772	0.346576217
cg01824933	17	0.00830789	0.6655099	0.646215767	0.78438687
cg01765623	17	0.01000517	0.19385673	0.1664236	0.33562374
cg01128025	4	0.0016785	0.32194735	0.3263221	0.546283839
cg16087077	15	0.028548	0.50503398	0.504674646	0.605780254
cg23086519	14	0.0012838	0.14632764	0.173441278	0.313416103
cg17990185	17	0.00429038	0.63305425	0.640758307	0.745316506
cg14523804	17	0.00122358	0.44831517	0.559892453	0.713971185
cg17723381	12	0.00576931	0.16079597	0.143442915	0.30399444
cg23499779	12	0.00455738	0.53444774	0.609265824	0.720421672
cg16020437	12	0.00395976	0.16640567	0.116511107	0.287316665
cg27366617	18	0.01663906	0.24779338	0.229823064	0.36496843
cg05843312	7	0.00388546	0.2255257	0.302910256	0.424457872
cg27107963	18	0.0111106	0.30457401	0.328665559	0.488375223
cg11856476	12	0.02524768	0.33382212	0.363879056	0.468907588
cg10108888	7	0.03209984	0.61642425	0.637464382	0.74343861
cg11596779	5	0.00152405	0.3872121	0.323037463	0.528190029
cg13307782	5	0.00857669	0.37797276	0.309894285	0.518952139
cg19109570	18	0.02794807	0.51421725	0.44418446	0.627406069
cg02571436	10	0.00073378	0.147229	0.196487454	0.497366748
cg17141972	6	0.00168532	0.64387497	0.639156154	0.802304803
cg01387072	4	0.002727	0.35823249	0.326512007	0.498530811
cg19547330	5	0.01665976	0.24830653	0.224611594	0.403204343
cg06646139	4	0.00205947	0.15137281	0.157603777	0.402436853
cg04313941	15	0.00152988	0.65503096	0.615143821	0.779277019
cg19359940	13	0.03363259	0.56349502	0.521296536	0.674347457
cg07197092	2	0.01093065	0.48414849	0.502500293	0.64731066
cg00540400	15	0.03153279	0.39729958	0.359709924	0.537344223
cg07311419	11	0.0167445	0.19235017	0.195130822	0.325982423
cg21393885	17	0.00314038	0.51509508	0.577402865	0.70184514
cg21430752	17	0.00138618	0.55798993	0.597593525	0.720897947
cg20219172	17	0.0016785	0.46772065	0.454495484	0.598319063
cg21034531	17	0.00202182	0.64870875	0.680337744	0.821650934
cg02744699	17	0.00166583	0.67381484	0.708900338	0.840940675
cg14951856	10	0.00279455	0.50321313	0.488179604	0.640083559
cg17100047	10	0.0042005	0.1774398	0.158277457	0.280063769
cg08326300	10	0.00228672	0.14772725	0.165079987	0.326885421
cg14962025	11	0.0190357	0.48576338	0.473955495	0.650138607
cg18745317	5	0.00937895	0.42745648	0.443843471	0.579982117
cg26207372	7	0.00783809	0.70154623	0.643442607	0.839300773
cg11411968	3	0.0035878	0.55009952	0.528498118	0.691050519
cg07818978	15	0.01952894	0.28990878	0.235803064	0.407262037

cg22405226	6	0.01126796	0.16779332	0.136599975	0.274363068
cg09214892	16	0.00144958	0.49532914	0.562683771	0.722053945
cg13537774	9	0.00417366	0.56524756	0.606133985	0.721993768
cg26710983	16	0.00336153	0.32339478	0.36117279	0.542043562
cg26955580	16	0.00093183	0.69723689	0.716106491	0.818530598
cg08057411	16	0.00458661	0.47026908	0.467042337	0.61523264
cg06214869	7	0.01177597	0.26886712	0.343069469	0.472112721
cg00925054	1	0.01660506	0.30040646	0.281805268	0.413981035
cg20267580	1	0.00904361	0.28685851	0.270712746	0.418798434
cg02203714	16	0.00132756	0.42567754	0.470294264	0.593765151
cg01275133	16	0.00354864	0.19811439	0.197315943	0.415763713
cg06213047	16	0.00202314	0.23476551	0.275507295	0.501222785
cg06259351	16	0.00283173	0.60501832	0.582747757	0.71959445
cg09274961	16	0.01336583	0.37986424	0.360656149	0.523456598
cg06761167	16	0.00116286	0.24785168	0.293344407	0.451227003
cg09437523	16	0.0012026	0.40803513	0.452910424	0.667534449
cg07725206	1	0.00089205	0.13225928	0.156974242	0.258199949
cg10228981	16	0.00080814	0.57773828	0.626557107	0.744414415
cg08390381	16	0.00521893	0.73451452	0.68918942	0.847215495
cg06984848	16	0.02651696	0.20946167	0.181653924	0.328495313
cg07765595	16	0.00101423	0.33491633	0.360273882	0.531762864
cg02522990	16	0.00066204	0.44389743	0.552749774	0.66079262
cg03096954	16	0.00195613	0.36372486	0.32424369	0.480106157
cg20208979	6	0.00314981	0.32044225	0.327858155	0.528237382
cg05540639	16	0.00429038	0.22607643	0.24257551	0.397483378
cg02244233	10	0.00310446	0.22869108	0.290532087	0.400850952
cg04607671	16	0.00097509	0.39591221	0.436991301	0.582764638
cg00834923	16	0.00081046	0.20687321	0.248645978	0.369185558
cg00866541	16	0.00147559	0.39801064	0.463247227	0.652214878
cg08550517	16	0.00065949	0.17253488	0.2529967	0.449649577
cg08006672	16	0.00703549	0.6305209	0.619501178	0.74101384
cg05824809	16	0.01408624	0.29331241	0.29996051	0.41860715
cg01481978	16	0.00588728	0.29611301	0.308021746	0.471028217
cg27219972	16	0.01430792	0.21923692	0.202540712	0.346179977
cg00101940	16	0.00168532	0.28752498	0.268714716	0.461824571
cg27338109	16	0.00128724	0.42356763	0.412809934	0.553269625
cg05905731	16	0.00130534	0.39938213	0.397157609	0.524039661
cg25045013	15	0.00806542	0.54654335	0.549367939	0.677899495
cg27065717	16	0.00098825	0.61870139	0.675593714	0.784607893
cg10174683	16	0.00220808	0.34261358	0.371970182	0.528399083
cg01072388	16	0.04804069	0.26667284	0.223407396	0.377790888
cg07066163	16	0.00170736	0.63165857	0.597526597	0.769506469
cg11524773	6	0.00578816	0.22935577	0.291289656	0.407772315
cg04213390	2	0.00071044	0.12373085	0.202997424	0.331784186
cg09296742	14	0.0252514	0.27923317	0.273777266	0.436853381
cg01984854	16	0.00777499	0.34784699	0.294768498	0.452473619

cg08183425	2	0.00160732	0.40595525	0.393970258	0.508035548
cg00925244	2	0.0066366	0.21936198	0.246333287	0.347622013
cg05021291	2	0.01782206	0.43364888	0.45491958	0.633091896
cg01234251	11	0.00362881	0.26081344	0.213887708	0.509315467
cg01803461	16	0.00629918	0.22267143	0.206818813	0.384577107
cg08785317	16	0.04180327	0.22106389	0.226124592	0.339355916
cg09941601	16	0.00315582	0.2342818	0.256690045	0.447287831
cg16681649	11	0.00930715	0.61787431	0.696938123	0.799295522
cg24006667	7	0.01207774	0.2706785	0.298402677	0.430255289
cg25830548	6	0.03437206	0.23961389	0.275407472	0.389990449
cg07950220	16	0.00555806	0.17893843	0.159660371	0.357439614
cg04239863	16	0.00076471	0.28719483	0.373664627	0.515589036
cg08317243	16	0.00065949	0.61278041	0.657305302	0.763361292
cg26866349	16	0.00092661	0.64193383	0.693095419	0.814658235
cg07126126	16	0.00702537	0.54982853	0.587140114	0.740724433
cg07347205	16	0.00150458	0.20215646	0.235611147	0.366782686
cg07478240	16	0.00194569	0.19216267	0.19754631	0.364872813
cg03923640	16	0.01321271	0.19935826	0.195882661	0.318534548
cg05932560	16	0.01729366	0.20193088	0.157810751	0.322276736
cg08959182	5	0.0020562	0.51472173	0.544275532	0.673548874
cg26573923	16	0.00950438	0.42269941	0.396416196	0.556997589
cg19742687	11	0.0062271	0.31734457	0.345537248	0.520836389
cg02622846	11	0.00932052	0.39308125	0.343517529	0.536664525
cg10263737	16	0.00317466	0.34513585	0.340708817	0.479670671
cg08959094	16	0.00748137	0.28104873	0.254274075	0.434065664
cg03558269	16	0.01273892	0.2935021	0.276814576	0.416481445
cg03262260	16	0.00768774	0.24620127	0.20467138	0.369437366
cg03512577	16	0.0022496	0.22734775	0.19308282	0.349254999
cg09788978	16	0.0082702	0.32692694	0.284509006	0.46127073
cg09077862	5	0.02686466	0.20999125	0.219352724	0.343560778
cg00934299	16	0.00134295	0.44791334	0.454837148	0.571590008
cg02656871	16	0.00177922	0.19971143	0.198751875	0.342661686
cg01095004	16	0.00285469	0.27434046	0.257046874	0.433123929
cg00837308	16	0.00575247	0.56343904	0.590830085	0.696973202
cg04152616	16	0.00429038	0.15337327	0.129118792	0.299164598
cg03428314	16	0.00316669	0.25585852	0.250109376	0.372906858
cg03396664	16	0.0010144	0.34678594	0.380748294	0.503971441
cg05748238	16	0.00144751	0.41148275	0.447882499	0.575110932
cg07545858	16	0.00183809	0.32552691	0.402723917	0.59016669
cg00685614	16	0.00186737	0.20303357	0.190601395	0.320300166
cg08362283	16	0.00358374	0.19056835	0.183104694	0.323094811
cg17793497	15	0.00385922	0.27354627	0.307330807	0.507487638
cg13935765	9	0.0013522	0.51719442	0.498951929	0.663255899
cg13445230	9	0.00787909	0.18436267	0.166460845	0.355502631
cg06073695	6	0.00908024	0.39932689	0.382042836	0.508849187
cg06645120	16	0.00338706	0.56568489	0.59254228	0.705188352

cg06089606	3	0.00534171	0.24136163	0.247932157	0.417139091
cg08305808	15	0.00855208	0.21524006	0.162077762	0.326547422
cg26365847	14	0.04615769	0.4540456	0.457544497	0.567784532
cg10578701	10	0.0352175	0.21736337	0.219079791	0.335207202
cg14304674	9	0.00504019	0.38562097	0.465962362	0.574429502
cg19910940	1	0.02672791	0.36919217	0.347255944	0.471609203
cg11119301	12	0.00578285	0.15907386	0.139827426	0.358260463
cg24006770	1	0.00106082	0.35504712	0.382117622	0.65093961
cg18774857	15	0.03811638	0.21413241	0.19668762	0.335160073
cg09502464	7	0.01828536	0.22485452	0.24438499	0.388512751
cg23751171	12	0.00398606	0.29878845	0.293233784	0.497625221
cg03973379	7	0.01852917	0.31229186	0.369866747	0.476355551
cg08698198	5	0.00569156	0.24430056	0.252314217	0.354754456
cg12569007	10	0.01615583	0.16053845	0.146236252	0.263714008
cg07889827	10	0.04828533	0.3418288	0.350995856	0.469941453
cg22635041	10	0.02003798	0.45605786	0.454434503	0.560122966
cg02703145	9	0.04850545	0.21519958	0.20507348	0.322190694
cg12311651	15	0.04332612	0.39674861	0.376012013	0.501474549
cg05797224	1	0.0126518	0.12515923	0.106353101	0.239420497
cg21386766	12	0.00246645	0.40668684	0.440633321	0.611234571
cg00165078	1	0.00405622	0.48866246	0.516789859	0.631333414
cg17211890	10	0.01891899	0.66474693	0.633400879	0.773315976
cg20062681	11	0.00073265	0.31707084	0.423326712	0.565218438
cg23660866	14	0.0080158	0.25647916	0.243923327	0.384562097
cg10658307	11	0.00457044	0.18501934	0.159242757	0.323999283
cg27415425	8	0.01802754	0.44339117	0.438672631	0.588806053
cg19363785	8	0.00081925	0.40663427	0.49434341	0.64208435
cg14111437	5	0.04928347	0.18838457	0.142269319	0.304540574
cg01915328	14	0.00325899	0.12984597	0.125344034	0.286788505
cg01869093	14	0.00211366	0.17441058	0.178468826	0.294873312
cg16800228	15	0.00553981	0.67332059	0.680271418	0.793733563
cg13476964	7	0.00803433	0.56744278	0.577855248	0.721992577
cg11603201	12	0.00194981	0.67904421	0.733766125	0.834830376
cg07554408	14	0.00387834	0.17290787	0.209284105	0.35283376
cg23535322	14	0.00387229	0.28862965	0.308200795	0.417928681
cg00931957	1	0.00173751	0.51668055	0.583510313	0.724506605
cg14459158	9	0.008246	0.12877798	0.138044515	0.245799742
cg03595986	8	0.02447745	0.24532534	0.256890694	0.414800305
cg25526960	10	0.00541343	0.24867826	0.272167854	0.430010198
cg21106695	14	0.01370104	0.57659918	0.613137089	0.730410304
cg18220365	1	0.01151933	0.49424766	0.486671248	0.626366367
cg26389913	12	0.04280685	0.30554078	0.281380788	0.416550819
cg06803420	5	0.00254294	0.2336652	0.225735024	0.475097626
cg14123436	9	0.00157286	0.23105291	0.269418299	0.500383101
cg14605447	9	0.00178025	0.14171151	0.139062491	0.282399043
cg26330928	1	0.02995719	0.30761549	0.312289914	0.453324635

cg01655158	8	0.01004931	0.58745654	0.601803247	0.72370669
cg13520053	9	0.00642271	0.21578291	0.240488556	0.470475277
cg13280063	6	0.02683244	0.30096718	0.299598788	0.40886354
cg10421739	6	0.0016785	0.30005394	0.367456817	0.582682455
cg13463465	2	0.00716301	0.37153957	0.385338173	0.513170165
cg11806439	14	0.02433216	0.19476664	0.175020695	0.306470133
cg03548062	14	0.01083395	0.19904672	0.196354844	0.345846628
cg01194073	14	0.00685368	0.31807277	0.304536914	0.442245907
cg24707486	14	0.0194844	0.50725596	0.486303655	0.617721276
cg10506844	10	0.02822075	0.32816137	0.279795191	0.437437548
cg23856536	7	0.00070134	0.08562304	0.135079229	0.251579569
cg02019528	14	0.02673403	0.64344978	0.631339098	0.75040832
cg10548805	14	0.00092783	0.24189127	0.278725654	0.553413853
cg00881378	15	0.00382061	0.59606836	0.576322736	0.71093408
cg03991106	15	0.00240895	0.45130566	0.457428194	0.57337041
cg01099300	10	0.00810401	0.17870954	0.163647382	0.333126109
cg11782714	14	0.00147518	0.22626883	0.244848774	0.365370781
cg15337521	5	0.00177922	0.50906942	0.549167	0.717476553
cg11484721	15	0.00628685	0.27623064	0.295358863	0.434785078
cg16036584	5	0.00864245	0.30067344	0.272617866	0.467387278
cg26366958	2	0.00197007	0.42695492	0.429027211	0.622714871
cg14961690	11	0.03305066	0.29994274	0.279902151	0.453972692
cg01118348	14	0.00196477	0.29323926	0.303685826	0.404134805
cg10632966	10	0.00171661	0.60772521	0.674017018	0.775169649
cg05153781	14	0.00295256	0.23538219	0.209588764	0.397753976
cg13419345	3	0.00426429	0.35039234	0.45818838	0.581315252
cg16106254	12	0.02651696	0.20537064	0.183050277	0.323685804
cg27535538	7	0.0020154	0.4226876	0.489870668	0.593213791
cg14501119	14	0.00079224	0.23202378	0.253663738	0.362774146
cg06535003	6	0.00168532	0.09285399	0.101136213	0.206181142
cg02370923	2	0.02240881	0.23746076	0.226815742	0.408083206
cg12331004	2	0.01230099	0.27367614	0.260220865	0.449718877
cg21034034	2	0.01336583	0.20057259	0.163081817	0.319293653
cg12107466	10	0.00186404	0.46471626	0.497884884	0.672547993
cg06450976	6	0.00065949	0.56054911	0.6807384	0.788451697
cg21661768	2	0.00381594	0.12904947	0.123170518	0.255917032
cg14799129	3	0.00132756	0.44767718	0.53326024	0.67958548
cg26538335	12	0.00816896	0.40402227	0.392518739	0.527332529
cg15339026	13	0.00564684	0.14247763	0.108250796	0.278411748
cg14833024	12	0.03166005	0.23550104	0.213727877	0.351029695
cg11394820	2	0.00359037	0.28173106	0.279472946	0.429494145
cg26072561	8	0.00339056	0.34955485	0.321007626	0.514539003
cg14326909	6	0.03891003	0.34792575	0.309965711	0.467668202
cg01116425	2	0.01136421	0.2970989	0.356735074	0.46736525
cg01894366	3	0.00317432	0.14445681	0.158620198	0.293683542
cg17432647	5	0.01317683	0.34577079	0.356909075	0.522737004

cg12369091	12	0.00410164	0.35995101	0.417922538	0.532317301
cg26699655	11	0.02110372	0.21055047	0.183440061	0.334606604
cg26575105	6	0.00591291	0.38596995	0.387652822	0.501923195
cg11073813	6	0.0037804	0.22296312	0.186247379	0.367265396
cg17635080	12	0.0016682	0.32865458	0.376187094	0.542003387
cg09907439	12	0.01398885	0.38396486	0.365576801	0.489984099
cg08055994	2	0.01398885	0.43081916	0.41735868	0.570191373
cg04690342	2	0.03829642	0.26373293	0.208789797	0.381291886
cg12389397	1	0.00150566	0.39001739	0.414338495	0.587991427
cg10624395	11	0.02614058	0.26256196	0.218526668	0.380643565
cg10088622	1	0.01465218	0.14371082	0.125639265	0.271514802
cg19699170	2	0.0013643	0.29855612	0.337574278	0.487553614
cg25508181	5	0.02757672	0.14761121	0.150732697	0.258859218
cg08374552	5	0.00233476	0.36222628	0.407074083	0.553548533
cg01885839	5	0.04685951	0.52608196	0.550004481	0.655844659
cg19629079	12	0.00999043	0.30543305	0.320447831	0.482785591
cg13235891	12	0.00491882	0.15211403	0.146661024	0.257318308
cg06918928	12	0.01358672	0.21951037	0.211989331	0.323130398
cg22725830	13	0.0048909	0.32119043	0.327362545	0.483114503
cg01552748	13	0.00619212	0.61777284	0.595300851	0.718820925
cg24360892	13	0.00403972	0.23104451	0.257680162	0.36898862
cg18712566	13	0.00753069	0.37177386	0.409860198	0.545705691
cg04853995	12	0.01585898	0.34376105	0.421327768	0.528411524
cg14060417	12	0.00993335	0.33249959	0.349021258	0.544818792
cg13063274	3	0.00555806	0.2432292	0.237728553	0.471929623
cg19763682	13	0.0016785	0.470498	0.478446267	0.631635787
cg19649152	13	0.01116167	0.39356235	0.371429148	0.501852441
cg25114855	2	0.0015244	0.65494915	0.629449168	0.797238474
cg03743262	7	0.00368022	0.17918499	0.202352183	0.348644212
cg24030485	13	0.00798815	0.34927689	0.359780769	0.511920416
cg22122342	13	0.02823023	0.67632985	0.692535901	0.814204515
cg11707796	13	0.00434915	0.30287488	0.2852132	0.432257205
cg19129687	13	0.0035878	0.47989188	0.508503491	0.610057186
cg13099542	13	0.02655172	0.53962717	0.583206187	0.701187848
cg26655004	13	0.00775794	0.73428073	0.703850209	0.845650571
cg22484690	1	0.00330705	0.61079211	0.622353449	0.737682474
cg21408197	2	0.01596342	0.17468479	0.158286927	0.333169006
cg02305412	1	0.03615697	0.42203323	0.409988582	0.566082517
cg23591463	12	0.0011141	0.09411351	0.139415509	0.258500042
cg01184975	12	0.00084118	0.07502445	0.132396558	0.242537956
cg20249252	12	0.00149569	0.11434965	0.186058882	0.306813383
cg03104083	12	0.00762625	0.21907718	0.267679395	0.40672593
cg00386586	13	0.04042709	0.49314286	0.50062928	0.602700338
cg13697968	13	0.00629918	0.34791528	0.348282842	0.498566567
cg02332936	9	0.02050584	0.32602244	0.265823404	0.497846797
cg06482783	5	0.01485881	0.6773159	0.675259273	0.784625509

cg15203566	5	0.00111524	0.26433851	0.251558617	0.383127726
cg12551394	12	0.00151706	0.47645221	0.485314191	0.651436527
cg25109508	1	0.00658287	0.22292742	0.169115511	0.346540535
cg07148025	5	0.0035878	0.15917966	0.135763672	0.304922493
cg01792876	8	0.02385226	0.19367137	0.224136367	0.360794917
cg19576241	12	0.02075001	0.14833682	0.149217483	0.26693334
cg13645530	12	0.00337574	0.16919916	0.163443761	0.3016802
cg01558281	1	0.01064534	0.26174316	0.231122952	0.38406007
cg13761419	9	0.00276502	0.10455479	0.120745417	0.222907288
cg20637688	12	0.00747631	0.47546176	0.500336782	0.626542171
cg04962865	8	0.00521564	0.14739833	0.174855155	0.364757768
cg15317129	3	0.00725763	0.59506074	0.527758718	0.721573504
cg22138219	8	0.0070758	0.50316456	0.567359645	0.677196122
cg01536979	8	0.01614766	0.26226476	0.21227673	0.369038249
cg20366218	2	0.03585699	0.50480396	0.511042243	0.624744837
cg13796936	1	0.00167443	0.19220421	0.205357181	0.424751308
cg26120971	8	0.00377759	0.22796721	0.237727357	0.37138031
cg20778022	7	0.00203938	0.54174323	0.579204979	0.715908357
cg16600876	8	0.04638176	0.31421294	0.269042524	0.441428759
cg27551395	1	0.00368543	0.55913987	0.469208323	0.686817893
cg09221960	11	0.00259677	0.53467869	0.517043937	0.649753574
cg23340777	12	0.0012838	0.21619136	0.272349432	0.494006401
cg27310831	8	0.00181052	0.55871239	0.595054052	0.700946607
cg25306875	2	0.00540086	0.32168151	0.273028078	0.487428947
cg26373051	2	0.00111281	0.15802658	0.192048446	0.359127044
cg00637745	2	0.00077454	0.12490469	0.157293757	0.272516427
cg17870997	2	0.00166583	0.31849301	0.345893545	0.513712458
cg18097872	2	0.00122881	0.31449172	0.359178292	0.484410002
cg15699099	2	0.00131467	0.26993	0.297482206	0.526507387
cg14658300	5	0.00583531	0.56526291	0.590660541	0.709836016
cg23834919	11	0.01359116	0.13090189	0.121332739	0.246097858
cg04522792	8	0.01172344	0.19645487	0.146882328	0.331617194
cg14256511	12	0.00081925	0.45975997	0.544045438	0.692801342
cg24571086	10	0.00432931	0.3097893	0.269684183	0.455367631
cg08349093	10	0.00971274	0.32563684	0.335377856	0.469987359
cg08211704	8	0.00091907	0.46904392	0.571829614	0.681238021
cg22959827	5	0.0086963	0.43115808	0.482213335	0.592886383
cg25051510	12	0.00134844	0.34184029	0.374878213	0.53084784
cg23400222	5	0.0249821	0.24281525	0.265928331	0.376140174
cg25024515	12	0.01980843	0.29872447	0.253825612	0.470581198
cg18648169	12	0.0016785	0.24356243	0.226491924	0.369048162
cg12277789	10	0.00240097	0.28612638	0.308223118	0.496998219
cg11846355	12	0.00219711	0.61743591	0.603851408	0.722788237
cg02807786	12	0.00356915	0.51219294	0.516542065	0.681734358
cg11818127	12	0.00140062	0.15913153	0.152501637	0.298362481
cg24691330	8	0.00589429	0.52080229	0.549597614	0.695166279

cg04658679	12	0.00733128	0.23724065	0.287063908	0.471924659
cg09789874	12	0.00081925	0.07677797	0.114414891	0.225383568
cg15226808	6	0.00318584	0.57092527	0.599598432	0.724830542
cg14267222	10	0.02252087	0.22338173	0.204223366	0.32935178
cg03464765	11	0.0020562	0.49155062	0.545418956	0.732260878
cg10903132	10	0.00989055	0.59120775	0.539993999	0.698457125
cg05872570	10	0.00119385	0.65466739	0.681092585	0.796980684
cg21392435	8	0.0007088	0.27732029	0.339937603	0.458461139
cg27031099	8	0.0008696	0.09013946	0.118769094	0.225872596
cg26781392	6	0.00459107	0.48410515	0.454922773	0.662582961
cg05165087	5	0.0104642	0.20676852	0.243833776	0.354036276
cg10522535	11	0.03431393	0.43927141	0.44523598	0.583048454
cg19153828	2	0.00286125	0.18701561	0.165184791	0.297540611
cg25214552	4	0.02045637	0.18592792	0.203627931	0.339623135
cg23756265	11	0.00065949	0.29704759	0.390515807	0.58040694
cg23487517	11	0.01358672	0.20378011	0.190367906	0.357555707
cg06521347	8	0.0104642	0.3342723	0.421337239	0.532463404
cg01571001	3	0.00960362	0.22125318	0.198110881	0.343707718
cg21121082	11	0.00620216	0.21179797	0.176959398	0.419142696
cg10334741	11	0.00789188	0.22197509	0.162627089	0.328834972
cg15010140	2	0.01172466	0.51128297	0.493911065	0.639454546
cg17297696	11	0.01159673	0.61298362	0.678946744	0.800975946
cg02148562	8	0.00362881	0.18826935	0.192593956	0.319264618
cg02433979	7	0.0018345	0.21945797	0.25119294	0.406702838
cg18261434	2	0.00243028	0.64276659	0.681051573	0.781833356
cg10149870	2	0.04928347	0.22234037	0.201638345	0.346772642
cg21875839	2	0.01390012	0.59642606	0.582042799	0.702627387
cg13807254	9	0.01384967	0.19296128	0.176373776	0.309065271
cg01935436	7	0.01842724	0.1823584	0.153547519	0.288798948
cg10985970	11	0.00086166	0.52228835	0.583225061	0.707489157
cg12441957	10	0.0194844	0.22900164	0.19893271	0.32994509
cg23858650	12	0.00917376	0.24497388	0.210749394	0.376080309
cg02506248	12	0.02076533	0.29254066	0.261271678	0.407811155
cg23653492	12	0.01116167	0.28772449	0.279953153	0.468213794
cg04879750	12	0.0042711	0.25404514	0.235130133	0.380224368
cg02642891	10	0.01218672	0.52598977	0.468146402	0.630942143
cg08372212	10	0.00937465	0.40824871	0.29431786	0.526207301
cg16864819	11	0.00161582	0.41558318	0.47516734	0.651546274
cg01231009	8	0.00103808	0.52986648	0.593600633	0.71113107
cg02506275	12	0.02158529	0.50204365	0.479835603	0.619133696
cg13391028	11	0.00758361	0.17374285	0.149108077	0.312114799
cg19030317	11	0.00827092	0.74685202	0.735309637	0.857066188
cg11584284	6	0.00103447	0.59344101	0.645331153	0.757677264
cg14101442	11	0.00252833	0.27905412	0.278634948	0.529464471
cg14549841	12	0.01174797	0.46134594	0.495328821	0.602705178
cg19248958	10	0.00352201	0.51292589	0.57009355	0.723914493

cg03598731	8	0.01823439	0.28771218	0.259102854	0.389272631
cg17451493	10	0.00462969	0.60578285	0.571660463	0.722298587
cg14360029	9	0.00310772	0.16366517	0.146878366	0.31169958
cg26843278	10	0.00589429	0.45455423	0.45032495	0.591212969
cg12474686	10	0.00431388	0.62986565	0.628309888	0.761939011
cg11826295	10	0.00233284	0.46755071	0.412980342	0.585102407
cg19198993	12	0.00815824	0.31578933	0.3441158	0.450241982
cg08601192	10	0.02336655	0.56170248	0.582407	0.697035145
cg14623910	12	0.0023187	0.52594719	0.551619684	0.651938492
cg14145524	9	0.00368543	0.23768049	0.213340558	0.339731783
cg13253210	10	0.00296695	0.25213519	0.246377821	0.431532877
cg03371778	10	0.00594443	0.48433211	0.399917912	0.599563881
cg20824932	6	0.00312811	0.15025351	0.152347819	0.287894012
cg19546792	10	0.00150075	0.60228237	0.59665871	0.730497108
cg02862059	10	0.0097133	0.52124336	0.520693213	0.62894971
cg01205299	6	0.00727686	0.47475458	0.510408884	0.628343359
cg23200818	12	0.00169055	0.23297947	0.240270374	0.379099443
cg26415787	12	0.00411612	0.24693711	0.21855096	0.405377996
cg04847478	10	0.0012838	0.62016589	0.650656467	0.770701727
cg13406593	9	0.00489904	0.26107782	0.274504885	0.411582205
cg07593205	10	0.00396544	0.20542895	0.18860891	0.354529112
cg09505233	10	0.00798815	0.42028566	0.405633687	0.609203257
cg00753748	3	0.02708404	0.36283007	0.391710617	0.502935482
cg08452095	3	0.00262388	0.48518471	0.523347435	0.650116287
cg24794669	10	0.00174311	0.41685929	0.464259577	0.573397618
cg08981844	8	0.01007472	0.16581116	0.179355057	0.291353509
cg00402352	11	0.00986554	0.45340811	0.4337303	0.564318595
cg24692595	3	0.00230867	0.46316382	0.60697552	0.720265277
cg18121989	8	0.00227742	0.13985667	0.147383739	0.289560676
cg11929693	5	0.00276502	0.55909729	0.510375151	0.728556518
cg08954863	10	0.00309798	0.54208551	0.49219005	0.657583938
cg20824201	2	0.00354346	0.31862655	0.345371221	0.515545582
cg20595634	10	0.00787799	0.30509763	0.331190295	0.44055557
cg16139202	5	0.00890116	0.25860903	0.222750191	0.385720926
cg18992570	5	0.00736707	0.31024208	0.271891327	0.446413933
cg18682731	10	0.01786937	0.29008391	0.267830855	0.411916954
cg14107822	6	0.01532682	0.5686528	0.587632923	0.688012695
cg24680439	10	0.02692184	0.35280096	0.475381924	0.588830354
cg19958511	10	0.01088059	0.44773193	0.455862729	0.588863619
cg20481247	10	0.00174311	0.51353175	0.535784067	0.69986575
cg26509250	2	0.00456818	0.43660574	0.508719558	0.62890769
cg11368900	10	0.01207774	0.5928704	0.625662594	0.729316594
cg06139749	7	0.00099274	0.18707192	0.203825436	0.437153288
cg02516703	5	0.00398122	0.36600114	0.388280438	0.551065488
cg02490659	9	0.00325899	0.27567223	0.288190016	0.426384435
cg02654265	5	0.01019185	0.5425314	0.556517182	0.6752625

cg13429168	9	0.00359124	0.30571558	0.299971046	0.435373544
cg14402385	9	0.0048909	0.24696571	0.234070386	0.35433087
cg02204521	2	0.00279442	0.11568878	0.156387216	0.290753981
cg14463995	9	0.00430896	0.2821115	0.271609462	0.47481381
cg07879474	5	0.0121837	0.28742058	0.275139765	0.430220848
cg08819431	5	0.00357923	0.12866774	0.112373014	0.280327949
cg11698445	5	0.00299125	0.17066291	0.15871459	0.375817676
cg15942368	7	0.00429383	0.35877707	0.400834672	0.609825916
cg00271204	5	0.00145997	0.3158604	0.30261929	0.499813128
cg14130595	9	0.00205947	0.21726611	0.205485295	0.351418913
cg18631996	5	0.00073118	0.18905753	0.267955909	0.387160523
cg13534503	9	0.00221105	0.68391286	0.59865878	0.790234135
cg21924472	4	0.0007901	0.42104109	0.457336566	0.595583115
cg15224626	6	0.00419259	0.47524915	0.57237575	0.679865505
cg12418043	5	0.01317683	0.41278084	0.403551314	0.54557148
cg27411547	8	0.00249468	0.66909622	0.618331362	0.815480015
cg16927416	8	0.00065949	0.31474983	0.361473834	0.49279169
cg06575772	8	0.00068904	0.49101238	0.55603516	0.707751668
cg24134292	8	0.01334709	0.19769746	0.231841402	0.339632317
cg08918272	6	0.036552	0.18205883	0.142097027	0.321059338
cg15171155	3	0.00247655	0.50796224	0.547228912	0.692223038
cg26472326	5	0.00217021	0.25168766	0.374240116	0.477110611
cg07531093	8	0.01191142	0.21049336	0.178093564	0.350783884
cg15554023	1	0.00422785	0.17722108	0.180113117	0.288116371
cg10276948	8	0.00576931	0.43656962	0.43054341	0.547101529
cg06993255	8	0.00207081	0.29446766	0.296241717	0.415112938
cg06228407	2	0.00113894	0.35095555	0.356341373	0.646056892
cg15983072	2	0.00341932	0.35246249	0.461574543	0.573402224
cg08508936	5	0.00971274	0.43369691	0.441612542	0.55235836
cg18879198	2	0.00179741	0.36153484	0.394957146	0.540210566
cg03577128	3	0.03181424	0.60714551	0.601698952	0.714740038
cg04293770	5	0.00777499	0.54490524	0.601810143	0.7233925
cg06192592	6	0.00315576	0.19519096	0.244382815	0.365153255
cg23623851	3	0.00275807	0.29691295	0.278569929	0.421917971
cg25601481	6	0.00349732	0.42230424	0.373764972	0.54205868
cg14548047	5	0.00118039	0.11872972	0.138238386	0.244115791
cg11286742	3	0.00164763	0.52965499	0.585597708	0.739423915
cg06636220	3	0.00608941	0.56968818	0.543736589	0.680181302
cg00927503	2	0.00081925	0.52910032	0.616534953	0.767474965
cg21072457	5	0.00767399	0.59575631	0.572348108	0.70252678
cg09578620	2	0.00269197	0.24398636	0.204791494	0.431839884
cg10476950	7	0.00189311	0.57373878	0.608230141	0.771042519
cg13074572	7	0.00575746	0.53577179	0.549210204	0.684341582
cg12207367	2	0.00291636	0.16908553	0.169893544	0.376792974
cg06883206	4	0.00150566	0.23408169	0.24868524	0.359700336
cg20957370	5	0.0078101	0.29712119	0.347577951	0.463159032

cg04095226	6	0.00186758	0.32518296	0.469952648	0.584709527
cg23409007	6	0.00423787	0.54240827	0.42019024	0.712176161
cg00423598	7	0.00368593	0.32577237	0.352640019	0.492572513
cg23786812	7	0.02757672	0.50607631	0.523251181	0.636267882
cg12159689	7	0.00716301	0.55220818	0.56120053	0.66632113
cg01942797	7	0.00451843	0.21568581	0.222394507	0.343271322
cg05711023	3	0.00938113	0.6532918	0.639437647	0.780093849
cg05225634	6	0.00694471	0.33372454	0.3036352	0.457149609
cg09056876	6	0.01206679	0.25407752	0.220896327	0.369815876
cg04127867	7	0.00109306	0.47003348	0.478171534	0.633399099
cg02627405	5	0.00065949	0.64046043	0.694752504	0.798142401
cg19891924	5	0.0017637	0.15304399	0.159508555	0.295327213
cg00637940	7	0.01760022	0.2552121	0.264104661	0.39632336
cg24455158	7	0.02078916	0.56958254	0.56923118	0.710343342
cg09426420	7	0.02680775	0.52858494	0.537069544	0.665461979
cg20462493	5	0.0015244	0.27780324	0.310202204	0.556891425
cg10525394	6	0.00101436	0.38916566	0.411032674	0.558310758
cg02962647	6	0.00176816	0.45509253	0.448330128	0.587741605
cg25575845	7	0.0097133	0.44855022	0.45358163	0.590534327
cg00089966	5	0.01120433	0.16994179	0.180859666	0.284406186
cg25256538	6	0.02489039	0.39565456	0.38594308	0.500780359
cg18672053	5	0.01863458	0.48270447	0.48649186	0.606797254
cg00730887	4	0.03634588	0.16303454	0.161588635	0.276764076
cg12684999	2	0.00359124	0.30543369	0.298773481	0.457317566
cg25232786	3	0.01310065	0.56711661	0.574746452	0.740549054
cg25116792	3	0.00122621	0.65705358	0.505198254	0.761173779
cg18200075	1	0.00561533	0.3430575	0.350825789	0.493494787
cg18758281	2	0.02249063	0.22667176	0.208675123	0.375200084
cg03662049	2	0.00336646	0.23541113	0.202807084	0.339725524
cg15584084	4	0.02562377	0.35360913	0.384350207	0.542940351
cg16163174	6	0.00118039	0.33604158	0.408436817	0.599228923
cg21413947	1	0.02205285	0.47852448	0.515372464	0.624320001
cg10932916	5	0.0090396	0.31374268	0.402167277	0.502846707
cg23172911	1	0.00209654	0.16274801	0.148922723	0.343429096
cg16206344	6	0.00798815	0.3289044	0.356989375	0.516145458
cg21464704	6	0.03208015	0.20144837	0.152943889	0.301933251
cg15403758	4	0.02096205	0.50785751	0.54620185	0.664445665
cg24603922	6	0.00363129	0.4453251	0.437296453	0.547342686
cg23863157	4	0.01016149	0.35928696	0.342840598	0.525887172
cg03474525	6	0.02697984	0.3961901	0.429693877	0.569118942
cg14705155	6	0.00566397	0.40242834	0.368936575	0.517146972
cg06389605	6	0.01155671	0.24155167	0.226279391	0.38619014
cg03703733	6	0.00362881	0.44953518	0.551057659	0.670505225
cg18469813	6	0.00247734	0.32603073	0.400313842	0.526707296
cg22049902	6	0.02917135	0.67968886	0.659899593	0.793423641
cg03994717	6	0.00218799	0.21322064	0.18332626	0.380156877

cg05315633	6	0.00538453	0.16221604	0.160921625	0.287224924
cg20735682	6	0.00382061	0.35980541	0.3942862	0.496202412
cg14462369	6	0.0047687	0.30802413	0.324602566	0.48953637
cg10075004	6	0.03576735	0.49919868	0.525584813	0.642923842
cg05184395	6	0.00670577	0.1604286	0.134606692	0.27618211
cg05740106	6	0.00458537	0.72958914	0.760618866	0.862883594
cg02383285	6	0.00214604	0.20728181	0.233776645	0.386849883
cg10122103	6	0.00299824	0.26235023	0.247381277	0.369341284
cg09867546	6	0.00431293	0.25746326	0.237697045	0.450870551
cg12201544	6	0.00088355	0.26003205	0.323550418	0.513301752
cg17017878	6	0.00081925	0.18441212	0.206306205	0.486198567
cg23253674	6	0.00103447	0.33091099	0.377297135	0.607395044
cg24148931	6	0.02152265	0.69554755	0.707090308	0.809369528
cg08672919	6	0.00079512	0.25136328	0.342617478	0.523799043
cg23180941	5	0.00211366	0.71720061	0.692678479	0.820380493
cg24527591	3	0.03744929	0.27117449	0.264022257	0.371321335
cg13207606	6	0.00144365	0.64748837	0.694835776	0.797746164
cg07140792	5	0.00337574	0.09839794	0.103950959	0.209412167
cg00532399	5	0.02068126	0.35454925	0.379550203	0.500856715
cg17393083	3	0.0019957	0.18757038	0.175703135	0.473073396
cg08371532	5	0.00385922	0.24433719	0.275601986	0.385046529
cg20683802	5	0.00561533	0.33492444	0.349659935	0.498259548
cg24720604	5	0.02919075	0.21533678	0.179313214	0.322863559
cg14524724	5	0.00332795	0.16038063	0.160450569	0.291871578
cg16213787	2	0.00262388	0.15117777	0.165542017	0.394200536
cg01418539	2	0.00768774	0.12586214	0.116452153	0.232644611
cg25912037	2	0.01612589	0.23526331	0.175195338	0.352718844
cg22992137	2	0.00476647	0.52602742	0.539649819	0.686397395
cg21748138	2	0.00483687	0.50968349	0.525956558	0.642698341
cg00688246	3	0.00344535	0.12662349	0.138211346	0.301635966
cg18607127	5	0.00428303	0.40827922	0.430634163	0.547718374
cg00075103	3	0.0096307	0.17112163	0.190636331	0.293994394
cg20187173	3	0.00816896	0.14614406	0.124217948	0.309918611
cg10541663	3	0.00782077	0.30597414	0.263027965	0.48432325
cg25925252	5	0.0071227	0.3062936	0.306681762	0.4219244
cg09267467	1	0.00330705	0.18844737	0.178006489	0.325765152
cg07535366	5	0.00112679	0.6078162	0.625962573	0.734460878
cg21188409	3	0.0012838	0.2913761	0.305712951	0.517683928
cg24990255	2	0.00379486	0.33790729	0.275970019	0.495939275
cg01549525	1	0.02445578	0.2551065	0.274244772	0.38142001
cg10152627	3	0.01329482	0.26684215	0.283126843	0.383938596
cg13460850	3	0.00340195	0.20161593	0.258077087	0.390343108
cg07467854	3	0.01502763	0.1847996	0.150634958	0.303926633
cg16893634	3	0.00141699	0.16196737	0.178550622	0.329562682
cg00432953	3	0.01077982	0.31844703	0.377443574	0.51180268
cg22607164	3	0.0082702	0.20422825	0.166536864	0.307083927

cg16510460	3	0.00309973	0.18796939	0.190385706	0.291829629
cg00163089	1	0.00133251	0.13790151	0.14038229	0.27855613
cg25556051	4	0.01073914	0.39711912	0.428365879	0.583597019
cg12354056	3	0.00387438	0.38763818	0.424346193	0.565810931
cg01394884	4	0.00297384	0.16285431	0.168658169	0.333380435
cg27338202	3	0.02265145	0.12758713	0.12203584	0.227964798
cg12626957	3	0.0022985	0.11816412	0.107907001	0.233298467
cg15813266	4	0.00227742	0.72339688	0.744371363	0.849102354
cg00317585	4	0.00279494	0.34959565	0.356860343	0.502095116
cg21351437	4	0.00471011	0.29051539	0.337973799	0.489249527
cg13755270	3	0.00076471	0.18801798	0.234943712	0.443583587
cg05782966	2	0.01317021	0.5675965	0.564622352	0.697074309
cg03055271	1	0.03161897	0.41178404	0.351831047	0.520173312
cg03209398	2	0.00689691	0.21228808	0.181685652	0.352357819
cg05970547	4	0.02910643	0.6359426	0.659854693	0.77811658
cg14460008	2	0.00211366	0.54542788	0.609979214	0.713845356
cg27327370	2	0.04601984	0.66899807	0.659903038	0.792726605
cg04051365	3	0.00113577	0.26562853	0.400222785	0.55543386
cg26345916	3	0.00262388	0.17093793	0.162255123	0.316860792
cg27423739	3	0.00149088	0.18895496	0.196819307	0.366809463
cg00271177	3	0.00181052	0.17611697	0.175545924	0.352696225
cg00209623	3	0.00085205	0.31675491	0.339351116	0.473690578
cg23003225	3	0.02944061	0.50285126	0.487395533	0.631339315
cg04485482	3	0.02126887	0.47048842	0.503316856	0.643166485
cg20074526	3	0.01100502	0.16611056	0.168647382	0.302617556
cg15578332	3	0.02979258	0.25507297	0.245652923	0.375414839
cg18229092	2	0.00654696	0.66261216	0.651294041	0.771047191
cg09197279	2	0.00303201	0.2619193	0.274698523	0.440092752
cg18558476	3	0.00331301	0.34247721	0.469415035	0.603580578
cg26853787	2	0.00702941	0.27075098	0.317872401	0.485004561
cg09155881	2	0.00297078	0.16213317	0.182404474	0.287690044
cg11719784	1	0.00213077	0.57709151	0.621589523	0.723734219
cg16516919	1	0.03453375	0.27875259	0.28061694	0.385761557
cg15824962	1	0.00368022	0.15907524	0.197889121	0.413351148
cg22218806	1	0.0126518	0.20688772	0.235682327	0.349523646
cg13235761	1	0.00077582	0.13841733	0.18473719	0.330063647
cg18378955	1	0.04407901	0.23457739	0.238367592	0.393939221
cg15765889	2	0.0094452	0.26244186	0.228235757	0.421754535
cg19990373	2	0.0042395	0.25139823	0.235290438	0.45196302
cg20817822	1	0.00168532	0.67980314	0.670727698	0.779986768
cg18011188	2	0.0157632	0.41529752	0.465859608	0.585249461
cg12277870	1	0.04320918	0.3306143	0.296907671	0.436435345
cg09937500	2	0.00679225	0.15131642	0.15150455	0.291723633
cg02059896	2	0.01580038	0.61133351	0.626536784	0.736086836
cg06653292	2	0.00195	0.51286713	0.508211058	0.731070771
cg16976599	2	0.00279442	0.41349852	0.530314584	0.659188396

cg00506297	1	0.02735855	0.56403167	0.583316347	0.700761975
cg10717691	1	0.00073118	0.14273434	0.249784278	0.389689597
cg26197530	1	0.03659438	0.19408635	0.180673776	0.297816617
cg06123700	1	0.01457962	0.22869908	0.220742059	0.365543398
cg01081168	1	0.03306333	0.24611058	0.245936585	0.421759393
cg24296187	1	0.0033331	0.15682144	0.161166725	0.313119127
cg22761483	2	0.04407901	0.42038622	0.4086471	0.558824018
cg00760938	2	0.00338706	0.49798115	0.516902923	0.622990414
cg19907293	2	0.00101775	0.33718157	0.396697902	0.620914916
cg06271720	2	0.01110735	0.17649621	0.199057197	0.373969699
cg06453241	2	0.00445086	0.18255955	0.163747724	0.339383218
cg01089914	2	0.01488167	0.32703567	0.287400287	0.434618132
cg05783384	2	0.00809353	0.2721903	0.244425759	0.385920676
cg25464265	1	0.00262147	0.33050604	0.349502944	0.605155019
cg15730491	1	0.00137973	0.16777882	0.152551253	0.273574703
cg25001102	1	0.0074197	0.47175006	0.448343664	0.585879651
cg26047066	1	0.00691903	0.39309454	0.355925661	0.53011789
cg17969662	1	0.00099274	0.43720512	0.514308323	0.681390483
cg12804063	1	0.01268304	0.2237788	0.279765007	0.380825427
cg14380474	1	0.00485228	0.45770117	0.503908522	0.684251389
cg03970900	1	0.00403328	0.16511345	0.167214311	0.322283708
cg05998301	2	0.00352201	0.38027331	0.439649802	0.59051158
cg25552018	1	0.00368022	0.32829997	0.404535614	0.55208642
cg06193628	2	0.00590505	0.32397153	0.337458772	0.496463159
cg21663666	2	0.00519569	0.21307494	0.21471692	0.332305349
cg22374586	2	0.0048909	0.21108755	0.20809109	0.375669608
cg09494379	2	0.00177922	0.27262955	0.293703831	0.41438505
cg24172324	2	0.01093065	0.24148527	0.24175692	0.354763863
cg17008160	1	0.00065949	0.43596908	0.515724082	0.660430303
cg16685313	1	0.00187198	0.20470881	0.209414651	0.393172042
cg22699768	1	0.01660506	0.34047021	0.327068929	0.553527328
cg13055709	1	0.00216509	0.19733156	0.18372993	0.403935175
cg15924102	1	0.00255682	0.29656968	0.330369438	0.573227453
cg27584255	1	0.00945582	0.30872713	0.20254791	0.428961643
cg26601431	1	0.00208621	0.29952216	0.245375814	0.526228409
cg23597271	2	0.00170786	0.55043956	0.582649617	0.69618246
cg02800216	2	0.00429569	0.33795633	0.336175955	0.493001481
cg20722637	2	0.00166476	0.71494102	0.729669918	0.850021955
cg15065627	2	0.01050457	0.26328242	0.222029315	0.403179587
cg09326079	2	0.00456818	0.29473683	0.325553948	0.525235835
cg17263013	2	0.0122155	0.34672017	0.375924528	0.496800386
cg08873746	2	0.00152206	0.51335538	0.514925882	0.692492019
cg09050372	2	0.0015244	0.50302504	0.562076319	0.67221944
cg11843673	2	0.00385922	0.56748335	0.605880883	0.726124162
cg19821872	2	0.00189311	0.54692422	0.574055575	0.705930791
cg20342432	2	0.00989476	0.45849969	0.493224554	0.615349473

cg13750219	2	0.00367708	0.56557444	0.582236821	0.697774823
cg26057577	2	0.00973492	0.24533818	0.223327441	0.389545051
cg08200420	2	0.00276502	0.37005069	0.386693551	0.516738694
cg00459898	2	0.00667307	0.22037469	0.171550935	0.342424378
cg03388247	2	0.00085205	0.53445349	0.566981842	0.693656762
cg23899450	2	0.00211366	0.38444149	0.435094106	0.578685945
cg15506703	2	0.03587201	0.36003585	0.322848855	0.472482408
cg16016888	2	0.03452515	0.61317022	0.585473103	0.757164094
cg13298632	2	0.01678444	0.71376303	0.690824996	0.868596827
cg27540865	1	0.02195328	0.12591845	0.116347033	0.302505628
cg12748499	1	0.00087274	0.68327215	0.555963737	0.810658561
cg22036872	1	0.00349658	0.43709685	0.508473956	0.620344867
