# Supplementary material for: Differential DNA methylation and expression of inflammatory and zinc transporter genes defines subgroups of osteoarthritic hip patients
Source: Ann Rheum Dis. 2015 Apr 8;74(9):1778–82. doi: 10.1136/annrheumdis-2014-206752 (PMC4552898; doi:10.1136/annrheumdis-2014-206752)
Supplement: Web table 5 [file annrheumdis-2014-206752-s9.pdf]

Supplementary Table 5. GO terms enriched in genes hypomethylated in OA hip clutser 2. P value is Bonferroni corrected.

Term	Number of genes	P-value
immune system process (GO:0002376)	198	1.28E-19
system development (GO:0048731)	306	2.29E-19
anatomical structure development (GO:0048856)	332	7.60E-18
immune response (GO:0006955)	130	5.76E-15
cell communication (GO:0007154)	361	6.02E-15
signal transduction (GO:0007165)	327	1.17E-13
locomotion (GO:0040011)	118	1.55E-12
defense response (GO:0006952)	128	2.10E-11
anatomical structure morphogenesis (GO:0009653)	175	5.06E-11
response to stress (GO:0006950)	241	1.12E-10
protein binding (GO:0005515)	523	1.35E-10
response to external stimulus (GO:0009605)	154	1.41E-10
response to organic substance (GO:0010033)	183	1.08E-09
regulation of immune response (GO:0050776)	83	1.87E-09
positive regulation of immune response (GO:0050778)	63	2.60E-09
cellular response to chemical stimulus (GO:0070887)	171	3.35E-09
innate immune response (GO:0045087)	87	3.78E-09
neurogenesis (GO:0022008)	123	5.01E-09
regulation of immune system process (GO:0002682)	110	7.36E-09
regulation of cell communication (GO:0010646)	206	8.17E-09
positive regulation of immune system process (GO:0002684)	80	1.27E-08
generation of neurons (GO:0048699)	115	4.39E-08
chemotaxis (GO:0006935)	69	4.85E-08
regulation of signaling (GO:0023051)	202	6.26E-08
localization of cell (GO:0051674)	80	8.92E-08
cell motility (GO:0048870)	80	8.92E-08
cell migration (GO:0016477)	75	1.08E-07
localization (GO:0051179)	294	5.26E-07
regulation of signal transduction (GO:0009966)	179	1.95E-06
cellular response to endogenous stimulus (GO:0071495)	86	2.74E-06
response to oxygen-containing compound (GO:1901700)	104	3.15E-06
regulation of hydrolase activity (GO:0051336)	105	4.06E-06
cell activation (GO:0001775)	66	4.19E-06
extracellular matrix organization (GO:0030198)	46	6.65E-06
activation of immune response (GO:0002253)	45	1.04E-05
immune system development (GO:0002520)	62	1.18E-05
neuron differentiation (GO:0030182)	86	1.23E-05
neuron development (GO:0048666)	74	1.86E-05
hematopoietic or lymphoid organ development (GO:0048534)	59	2.49E-05
immune response-regulating signaling pathway (GO:0002764)	48	4.94E-05
ion binding (GO:0043167)	361	4.97E-05
receptor activity (GO:0004872)	118	8.07E-05

neuron projection development (GO:0031175)	63	8.46E-05
regulation of biological quality (GO:0065008)	192	1.01E-04
positive regulation of phosphorus metabolic process (GO:0010	109	1.09E-04
positive regulation of phosphate metabolic process (GO:00459	109	1.09E-04
neuron projection morphogenesis (GO:0048812)	55	1.30E-04
positive regulation of cell communication (GO:0010647)	103	1.54E-04
cell-cell signaling (GO:0007267)	82	1.97E-04
intracellular signal transduction (GO:0035556)	119	2.04E-04
axon development (GO:0061564)	52	2.14E-04
response to other organism (GO:0051707)	63	2.26E-04
response to external biotic stimulus (GO:0043207)	63	2.26E-04
response to biotic stimulus (GO:0009607)	65	2.29E-04
axon guidance (GO:0007411)	43	2.39E-04
neuron projection guidance (GO:0097485)	43	2.39E-04
positive regulation of hydrolase activity (GO:0051345)	75	2.41E-04
signal transducer activity (GO:0004871)	120	3.25E-04
molecular transducer activity (GO:0060089)	120	3.25E-04
axonogenesis (GO:0007409)	50	3.36E-04
immune response-regulating cell surface receptor signaling pat	38	3.40E-04
positive regulation of lymphocyte activation (GO:0051251)	32	3.41E-04
positive regulation of defense response (GO:0031349)	35	3.42E-04
response to bacterium (GO:0009617)	45	3.85E-04
mRNA metabolic process (GO:0016071)	7	4.47E-04
cellular response to oxygen-containing compound (GO:190170	68	5.13E-04
actin binding (GO:0003779)	42	5.30E-04
regulation of leukocyte activation (GO:0002694)	43	5.58E-04
positive regulation of T cell activation (GO:0050870)	27	5.91E-04
hemopoiesis (GO:0030097)	52	5.97E-04
positive regulation of leukocyte activation (GO:0002696)	33	6.40E-04
immune response-activating signal transduction (GO:0002757)	37	7.19E-04
positive regulation of immune effector process (GO:0002699)	22	7.41E-04
defense response to other organism (GO:0098542)	39	7.46E-04
positive regulation of signaling (GO:0023056)	100	7.47E-04
regulation of defense response (GO:0031347)	52	1.06E-03
blood vessel development (GO:0001568)	46	1.20E-03
leukocyte activation (GO:0045321)	43	1.25E-03
cell projection morphogenesis (GO:0048858)	63	1.40E-03
cell morphogenesis involved in neuron differentiation (GO:004	51	1.41E-03
regulation of immune effector process (GO:0002697)	33	1.48E-03
organ morphogenesis (GO:0009887)	74	1.70E-03
cell projection organization (GO:0030030)	78	1.80E-03
regulation of interleukin-1 beta production (GO:0032651)	11	2.35E-03
regulation of cytokine production (GO:0001817)	48	3.14E-03
cell part morphogenesis (GO:0032990)	63	3.24E-03
regulation of cell proliferation (GO:0042127)	104	3.32E-03
vasculature development (GO:0001944)	47	3.33E-03

proteoglycan biosynthetic process (GO:0030166)	13	3.40E-03
mRNA processing (GO:0006397)	3	3.51E-03
regulation of apoptotic process (GO:0042981)	100	3.60E-03
cytoskeletal protein binding (GO:0008092)	65	3.76E-03
immune effector process (GO:0002252)	43	3.87E-03
positive regulation of cytokine production (GO:0001819)	37	4.11E-03
negative regulation of immune system process (GO:0002683)	35	4.17E-03
cytokine production (GO:0001816)	19	4.45E-03
response to lipopolysaccharide (GO:0032496)	29	4.85E-03
positive regulation of catabolic process (GO:0009896)	63	4.86E-03
regulation of programmed cell death (GO:0043067)	100	4.98E-03
positive regulation of interleukin-1 beta production (GO:00327	9	5.02E-03
response to molecule of bacterial origin (GO:0002237)	30	5.54E-03
response to lipid (GO:0033993)	60	6.11E-03
positive regulation of GTP catabolic process (GO:0033126)	47	6.49E-03
positive regulation of GTPase activity (GO:0043547)	47	6.49E-03
regulation of interleukin-1 production (GO:0032652)	11	6.49E-03
receptor binding (GO:0005102)	97	6.69E-03
regulation of response to stress (GO:0080134)	78	7.52E-03
regulation of cell death (GO:0010941)	103	7.62E-03
cell adhesion (GO:0007155)	70	7.96E-03
proteoglycan metabolic process (GO:0006029)	17	7.97E-03
circulatory system development (GO:0072359)	65	8.34E-03
cardiovascular system development (GO:0072358)	65	8.34E-03
regulation of GTPase activity (GO:0043087)	49	9.06E-03
positive regulation of lymphocyte mediated immunity (GO:000	14	9.09E-03
cellular component organization (GO:0016043)	268	9.88E-03
positive regulation of leukocyte mediated immunity (GO:0002	15	1.01E-02
antigen receptor-mediated signaling pathway (GO:0050851)	19	1.05E-02
immune response-activating cell surface receptor signaling pat	25	1.11E-02
regulation of T cell activation (GO:0050863)	30	1.11E-02
leukocyte differentiation (GO:0002521)	32	1.13E-02
regulation of response to external stimulus (GO:0032101)	54	1.14E-02
regulation of transport (GO:0051049)	106	1.16E-02
T cell costimulation (GO:0031295)	14	1.26E-02
positive regulation of interleukin-1 production (GO:0032732)	9	1.27E-02
T cell activation (GO:0042110)	27	1.28E-02
skeletal system development (GO:0001501)	43	1.30E-02
regulation of lymphocyte activation (GO:0051249)	36	1.38E-02
RNA binding (GO:0003723)	42	1.39E-02
response to interferon-gamma (GO:0034341)	18	1.44E-02
lymphocyte costimulation (GO:0031294)	14	1.48E-02
protein domain specific binding (GO:0019904)	51	1.51E-02
lymphocyte activation (GO:0046649)	35	1.52E-02
RNA splicing (GO:0008380)	2	1.78E-02
cellular response to growth factor stimulus (GO:0071363)	52	1.80E-02

T cell receptor signaling pathway (GO:0050852)	16	1.82E-02
response to growth factor (GO:0070848)	53	1.98E-02
peptide antigen binding (GO:0042605)	8	2.05E-02
secretion (GO:0046903)	49	2.55E-02
carbohydrate derivative binding (GO:0097367)	143	2.61E-02
regulation of lymphocyte mediated immunity (GO:0002706)	16	2.69E-02
cell proliferation (GO:0008283)	56	2.90E-02
metal ion binding (GO:0046872)	241	2.97E-02
synaptic transmission (GO:0007268)	53	3.09E-02
cation binding (GO:0043169)	244	3.74E-02
T cell differentiation (GO:0030217)	18	4.03E-02
regulation of leukocyte mediated immunity (GO:0002703)	18	4.03E-02
identical protein binding (GO:0042802)	80	4.19E-02
glycosaminoglycan biosynthetic process (GO:0006024)	16	4.98E-02
