# Supplementary material for: Differential DNA methylation and expression of inflammatory and zinc transporter genes defines subgroups of osteoarthritic hip patients
Source: Ann Rheum Dis. 2015 Apr 8;74(9):1778–82. doi: 10.1136/annrheumdis-2014-206752 (PMC4552898; doi:10.1136/annrheumdis-2014-206752)
Supplement: Web table 6 [file annrheumdis-2014-206752-s10.pdf]

Supplementary Table 6. GO terms enriched in genes hypermethylated in OA hip cluster 2. P value is Bonferroni corrected.

Term	Number of genes	P-value
regulation of response to stimulus (GO:0048583)	303	9.85E-18
anatomical structure development (GO:0048856)	384	1.06E-17
regulation of signal transduction (GO:0009966)	250	1.91E-17
cell differentiation (GO:0030154)	289	3.01E-16
regulation of signaling (GO:0023051)	267	3.15E-16
regulation of cell communication (GO:0010646)	266	8.20E-16
anatomical structure morphogenesis (GO:0009653)	212	4.88E-14
ion binding (GO:0043167)	474	1.24E-13
localization (GO:0051179)	363	1.06E-10
response to stimulus (GO:0050896)	539	1.38E-10
cellular component organization (GO:0016043)	361	1.84E-10
organ development (GO:0048513)	244	3.03E-10
organ morphogenesis (GO:0009887)	106	5.98E-10
cellular response to stimulus (GO:0051716)	440	8.10E-10
nervous system development (GO:0007399)	190	8.75E-10
regulation of anatomical structure morphogenesis (GO:0022603)	97	1.77E-09
cellular component organization or biogenesis (GO:0071840)	363	2.29E-09
single organism signaling (GO:0044700)	388	2.41E-09
signaling (GO:0023052)	388	2.41E-09
positive regulation of hydrolase activity (GO:0051345)	101	4.71E-09
skeletal system development (GO:0001501)	66	4.73E-09
cell development (GO:0048468)	151	5.01E-09
signal transduction (GO:0007165)	358	6.47E-09
cell communication (GO:0007154)	391	8.28E-09
circulatory system development (GO:0072359)	94	9.86E-09
cardiovascular system development (GO:0072358)	94	9.86E-09
negative regulation of response to stimulus (GO:0048585)	125	1.32E-08
negative regulation of signal transduction (GO:0009968)	107	1.44E-08
tissue development (GO:0009888)	153	3.28E-08
regulation of hydrolase activity (GO:0051336)	128	3.67E-08
metal ion binding (GO:0046872)	320	9.24E-08
regulation of cell morphogenesis (GO:0022604)	61	1.15E-07
actin binding (GO:0003779)	57	1.23E-07
positive regulation of molecular function (GO:0044093)	155	1.31E-07
negative regulation of signaling (GO:0023057)	111	2.06E-07
enzyme binding (GO:0019899)	138	2.24E-07
negative regulation of cell communication (GO:0010648)	111	2.44E-07
cellular component movement (GO:0006928)	131	3.18E-07
negative regulation of Wnt signaling pathway (GO:0030178)	30	3.34E-07
regulation of cellular component organization (GO:0051128)	162	4.32E-07
neurogenesis (GO:0022008)	134	4.95E-07
regulation of cell differentiation (GO:0045595)	131	6.32E-07

regulation of intracellular signal transduction (GO:1902531)	144	6.40E-07
cation binding (GO:0043169)	320	6.58E-07
positive regulation of GTPase activity (GO:0043547)	65	6.87E-07
regulation of GTPase activity (GO:0043087)	68	8.00E-07
regulation of small GTPase mediated signal transduction (GO:005	65	1.44E-06
generation of neurons (GO:0048699)	126	2.02E-06
cell morphogenesis involved in differentiation (GO:0000904)	77	2.63E-06
cell migration (GO:0016477)	81	3.17E-06
heart morphogenesis (GO:0003007)	37	3.78E-06
localization of cell (GO:0051674)	86	4.36E-06
cell motility (GO:0048870)	86	4.36E-06
regulation of Wnt signaling pathway (GO:0030111)	38	5.29E-06
regulation of Ras protein signal transduction (GO:0046578)	57	7.40E-06
locomotion (GO:0040011)	113	7.71E-06
neuron projection development (GO:0031175)	75	8.52E-06
ATP binding (GO:0005524)	136	9.68E-06
regulation of catalytic activity (GO:0050790)	182	1.02E-05
carbohydrate derivative binding (GO:0097367)	186	1.37E-05
positive regulation of catalytic activity (GO:0043085)	130	1.38E-05
tube development (GO:0035295)	77	1.55E-05
blood vessel morphogenesis (GO:0048514)	51	1.90E-05
nucleotide binding (GO:0000166)	194	1.90E-05
nucleoside phosphate binding (GO:1901265)	194	1.96E-05
adenyl ribonucleotide binding (GO:0032559)	137	2.27E-05
cell projection organization (GO:0030030)	97	2.32E-05
heart development (GO:0007507)	56	2.33E-05
intracellular signal transduction (GO:0035556)	142	2.39E-05
sensory perception of smell (GO:0007608)	2	2.48E-05
kinase activity (GO:0016301)	83	2.54E-05
regulation of Ras GTPase activity (GO:0032318)	49	2.56E-05
adenyl nucleotide binding (GO:0030554)	138	2.57E-05
embryo development (GO:0009790)	98	2.61E-05
tissue morphogenesis (GO:0048729)	67	2.66E-05
small molecule binding (GO:0036094)	212	2.87E-05
phosphotransferase activity, alcohol group as acceptor (GO:0016	77	5.10E-05
sensory perception of chemical stimulus (GO:0007606)	4	5.35E-05
embryo development ending in birth or egg hatching (GO:00079	71	5.40E-05
protein domain specific binding (GO:0019904)	67	5.42E-05
purine ribonucleoside binding (GO:0032550)	157	5.88E-05
blood vessel development (GO:0001568)	56	6.41E-05
purine nucleoside binding (GO:0001883)	157	6.52E-05
ribonucleoside binding (GO:0032549)	157	6.74E-05
purine ribonucleoside triphosphate binding (GO:0035639)	156	7.00E-05
GTPase binding (GO:0051020)	35	7.53E-05
epithelial tube morphogenesis (GO:0060562)	44	7.59E-05
chordate embryonic development (GO:0043009)	70	7.87E-05

cell morphogenesis (GO:0000902)	91	8.97E-05
nucleoside binding (GO:0001882)	157	9.45E-05
neuron projection morphogenesis (GO:0048812)	63	1.02E-04
regulation of biological quality (GO:0065008)	224	1.08E-04
vasculature development (GO:0001944)	58	1.17E-04
morphogenesis of an epithelium (GO:0002009)	54	1.23E-04
purine nucleotide binding (GO:0017076)	159	1.26E-04
connective tissue development (GO:0061448)	33	1.29E-04
neuron differentiation (GO:0030182)	95	1.33E-04
Wnt signaling pathway (GO:0016055)	37	1.70E-04
purine ribonucleotide binding (GO:0032555)	157	1.71E-04
protein phosphorylation (GO:0006468)	75	1.74E-04
positive regulation of Ras GTPase activity (GO:0032320)	42	1.75E-04
small GTPase binding (GO:0031267)	33	1.76E-04
neuron development (GO:0048666)	81	2.34E-04
extracellular matrix organization (GO:0030198)	48	2.48E-04
positive regulation of cell differentiation (GO:0045597)	75	2.59E-04
extracellular structure organization (GO:0043062)	48	2.69E-04
ribonucleotide binding (GO:0032553)	157	2.78E-04
angiogenesis (GO:0001525)	41	2.92E-04
cytoskeleton organization (GO:0007010)	79	2.92E-04
actin cytoskeleton organization (GO:0030036)	48	3.41E-04
nucleoside-triphosphatase regulator activity (GO:0060589)	43	3.64E-04
guanyl-nucleotide exchange factor activity (GO:0005085)	31	3.70E-04
GTPase regulator activity (GO:0030695)	41	4.31E-04
small GTPase regulator activity (GO:0005083)	30	4.57E-04
regulation of localization (GO:0032879)	164	5.10E-04
detection of chemical stimulus (GO:0009593)	5	5.28E-04
catalytic activity (GO:0003824)	386	7.56E-04
transferase activity, transferring phosphorus-containing groups (	89	7.78E-04
vesicle-mediated transport (GO:0016192)	99	7.87E-04
cellular component morphogenesis (GO:0032989)	93	7.91E-04
regulation of ossification (GO:0030278)	29	7.93E-04
epithelium development (GO:0060429)	94	8.23E-04
cell morphogenesis involved in neuron differentiation (GO:0048	59	8.37E-04
cellular localization (GO:0051641)	163	8.82E-04
gland development (GO:0048732)	48	9.88E-04
glycosaminoglycan biosynthetic process (GO:0006024)	21	9.91E-04
actin filament binding (GO:0051015)	20	1.08E-03
tube morphogenesis (GO:0035239)	54	1.10E-03
phosphorylation (GO:0016310)	99	1.16E-03
aminoglycan biosynthetic process (GO:0006023)	21	1.16E-03
protein kinase activity (GO:0004672)	64	1.28E-03
heterocyclic compound binding (GO:1901363)	401	1.77E-03
growth (GO:0040007)	47	1.96E-03
regulation of cellular component biogenesis (GO:0044087)	58	1.99E-03

proteoglycan metabolic process (GO:0006029)	20	2.02E-03
Ras GTPase binding (GO:0017016)	29	2.16E-03
establishment of localization (GO:0051234)	271	2.77E-03
negative regulation of ossification (GO:0030279)	16	3.08E-03
organic cyclic compound binding (GO:0097159)	403	3.32E-03
ossification (GO:0001503)	35	3.53E-03
mesenchyme development (GO:0060485)	27	3.68E-03
circadian regulation of gene expression (GO:0032922)	14	3.71E-03
negative regulation of canonical Wnt signaling pathway (GO:009(	19	4.11E-03
regulation of nervous system development (GO:0051960)	62	4.20E-03
regulation of cell development (GO:0060284)	69	4.21E-03
negative regulation of gene expression (GO:0010629)	125	4.65E-03
transport (GO:0006810)	264	4.67E-03
protein complex binding (GO:0032403)	78	4.68E-03
cartilage development (GO:0051216)	25	6.15E-03
GTPase activator activity (GO:0005096)	34	6.27E-03
cell projection morphogenesis (GO:0048858)	70	6.35E-03
regulation of cell shape (GO:0008360)	21	6.70E-03
glycosaminoglycan metabolic process (GO:0030203)	26	7.02E-03
mucopolysaccharide metabolic process (GO:1903510)	22	7.44E-03
in utero embryonic development (GO:0001701)	44	7.77E-03
single-organism localization (GO:1902578)	230	8.01E-03
embryonic morphogenesis (GO:0048598)	59	9.17E-03
developmental growth (GO:0048589)	30	1.06E-02
negative regulation of transcription, DNA-templated (GO:004589	89	1.07E-02
axon development (GO:0061564)	54	1.08E-02
Ras guanyl-nucleotide exchange factor activity (GO:0005088)	21	1.10E-02
skeletal system morphogenesis (GO:0048705)	30	1.16E-02
regulation of gene expression (GO:0010468)	300	1.28E-02
calcium ion binding (GO:0005509)	67	1.34E-02
sensory organ development (GO:0007423)	54	1.35E-02
axonogenesis (GO:0007409)	52	1.40E-02
regulation of neurogenesis (GO:0050767)	55	1.65E-02
positive regulation of gene expression (GO:0010628)	120	1.76E-02
aminoglycan metabolic process (GO:0006022)	26	1.80E-02
chondroitin sulfate metabolic process (GO:0030204)	14	1.94E-02
regulation of canonical Wnt signaling pathway (GO:0060828)	24	2.04E-02
negative regulation of nucleic acid-templated transcription (GO:1	90	2.12E-02
cellular response to endogenous stimulus (GO:0071495)	84	2.15E-02
response to stress (GO:0006950)	235	2.62E-02
regulation of osteoblast differentiation (GO:0045667)	19	2.64E-02
mesenchymal cell development (GO:0014031)	19	2.64E-02
cardiac chamber morphogenesis (GO:0003206)	19	2.64E-02
establishment of cell polarity (GO:0030010)	15	2.72E-02
proteoglycan biosynthetic process (GO:0030166)	13	2.73E-02
cellular response to growth factor stimulus (GO:0071363)	59	3.02E-02

cardiac chamber development (GO:0003205)	21	3.02E-02
regulation of Rho GTPase activity (GO:0032319)	26	3.23E-02
nephron development (GO:0072006)	20	3.24E-02
regulation of locomotion (GO:0040012)	62	3.25E-02
chondroitin sulfate proteoglycan metabolic process (GO:005065[illegible]	14	3.35E-02
urogenital system development (GO:0001655)	36	3.50E-02
regulation of transcription from RNA polymerase II promoter (G[illegible]	126	3.84E-02
morphogenesis of a branching epithelium (GO:0061138)	26	3.90E-02
establishment or maintenance of cell polarity (GO:0007163)	20	4.12E-02
regulation of Rho protein signal transduction (GO:0035023)	28	4.16E-02
carbohydrate derivative biosynthetic process (GO:1901137)	58	4.22E-02
transcription regulatory region DNA binding (GO:0044212)	53	4.29E-02
SH3 domain binding (GO:0017124)	19	4.50E-02
positive regulation of nervous system development (GO:005196[illegible]	38	4.54E-02
embryonic organ development (GO:0048568)	46	4.55E-02
regulation of osteoblast proliferation (GO:0033688)	8	4.67E-02
regulation of myotube differentiation (GO:0010830)	13	4.84E-02
sensory organ morphogenesis (GO:0090596)	32	4.87E-02
stem cell development (GO:0048864)	28	4.94E-02
regulation of cell adhesion (GO:0030155)	40	4.97E-02
