# Supplementary material for: Differential DNA methylation and expression of inflammatory and zinc transporter genes defines subgroups of osteoarthritic hip patients
Source: Ann Rheum Dis. 2015 Apr 8;74(9):1778–82. doi: 10.1136/annrheumdis-2014-206752 (PMC4552898; doi:10.1136/annrheumdis-2014-206752)
Supplement: Web table 7 [file annrheumdis-2014-206752-s11.pdf]

Supplementary Table 7. Details of the differentially methylated probes identified within inflammation associated genes. TSS1500 are probes that are located within 1500 bp upstream of the start codon; TSS200 probes located within 200 bp upstream of the start codon; 1st exon are probes located within the 1st exon of the gene and body are probes located within the gene body.

Gene	CpG probe ID	Chr.	Location	Region	Mean β value NOF	Mean β value OA hip cluster 1	Mean β value OA hip cluster 2	Benjamini-Hochberg p value
TNF	cg21370522	6	31543219	TSS200	0.59	0.64	0.47	0.02
	cg01569083		31543289	TSS200	0.56	0.58	0.46	0.03
	cg03037030		31543300	TSS200	0.44	0.46	0.27	0.01
	cg12681001		31543540	1st Exon	0.47	0.50	0.37	0.02
	cg21222743		31543545	1st Exon	0.33	0.37	0.26	0.005
	cg10717214		31543557	1st Exon	0.45	0.46	0.37	0.04
IL6	cg00087425	7	22766829	1st Exon	0.19	0.19	0.10	0.003
	cg13104385		22767384	Body	0.69	0.71	0.60	0.04
	cg05265849		22767390	Body	0.36	0.36	0.25	0.03
	cg07998387		22767571	Body	0.43	0.44	0.58	0.005
CXCR2	cg25941354	2	218989983	TSS1500	0.58	0.61	0.48	0.02
	cg10591797		218990598	TSS200	0.73	0.76	0.65	0.02
	cg13739417		218990627	TSS200	0.64	0.70	0.58	0.04
CCL5	cg02483931	17	34202461	Body	0.45	0.44	0.31	0.005
	cg08656816		34207454	TSS200	0.47	0.38	0.22	0.0008
CCL2	cg17864156	17	32582828	Body	0.44	0.44	0.32	0.01
IL1A	cg00839584	2	113542091	1st Exon	0.55	0.58	0.45	0.04
