# Supplementary material for: Differential DNA methylation and expression of inflammatory and zinc transporter genes defines subgroups of osteoarthritic hip patients
Source: Ann Rheum Dis. 2015 Apr 8;74(9):1778–82. doi: 10.1136/annrheumdis-2014-206752 (PMC4552898; doi:10.1136/annrheumdis-2014-206752)
Supplement: Web table 8 [file annrheumdis-2014-206752-s12.pdf]

Supplementary Table 8. Correlation between gene expression and methylation at the promoters of selected inflammation associated genes. The results are also shown for all of the probes in each gene combined. In the case of *IL6*, the three probes that showed a negative correlation with gene expression (cg00087425, cg13104385 and cg05265849) were combined. Scatter plots of the correlations below are shown in Supplementary Figure 1 and Supplementary Figure 2.

Gene	CpG probe ID	Spearman correlation	p value
TNF	cg21370522	-0.44	0.088
	cg01569083	-0.37	0.16
	cg03037030	-0.38	0.15
	cg12681001	-0.48	0.06
	cg21222743	-0.44	0.088
	cg10717214	-0.61	0.012
	Combined	-0.61	0.012
IL6	cg00087425	-0.41	0.11
	cg13104385	-0.56	0.024
	cg05265849	-0.57	0.021
	cg07998387	0.16	0.55
	Combined	-0.55	0.027
CXCR2	cg25941354	-0.38	0.15
	cg10591797	-0.4	0.12
	cg13739417	-0.38	0.15
	Combined	-0.5	0.049
CCL5	cg02483931	-0.68	0.0038
	cg08656816	-0.65	0.0064
	Combined	-0.69	0.0031
CCL2	cg17864156	-0.62	0.01
IL1A	cg00839584	-0.58	0.034
