# Supplementary material for: Differential DNA methylation and expression of inflammatory and zinc transporter genes defines subgroups of osteoarthritic hip patients
Source: Ann Rheum Dis. 2015 Apr 8;74(9):1778–82. doi: 10.1136/annrheumdis-2014-206752 (PMC4552898; doi:10.1136/annrheumdis-2014-206752)
Supplement: Web table 9 [file annrheumdis-2014-206752-s13.pdf]

Supplementary Table 9. Details of the differentially methylated probes identified within zinc transporter genes. The methylation differences at cg02362439 were genome-wide significant, however there was not a 10% difference in methylation between NOF and OA hip cluster 2.

Gene	CpG probe ID	Chr.	Location	Region	Mean β value NOF	Mean β value OA hip cluster 1	Mean β value OA hip cluster 2	Benjamini-Hochberg p value
ZIP4	cg22059438	8	145641882	Body	0.36	0.34	0.46	0.03
ZIP7	cg26750489	6	33171106	Body	0.59	0.54	0.76	0.002
	cg25059165		33171467	Body	0.34	0.32	0.50	0.003
	cg02362439		33171324	Body	0.63	0.59	0.72	0.006
ZIP11	cg25067702	17	70723227	Body	0.37	0.38	0.24	0.008
	cg11761483		70723386	Body	0.58	0.64	0.47	0.04
	cg21461745		70948937	Body	0.39	0.47	0.63	0.004
ZIP14	cg24136932	8	22281979	Body	0.44	0.52	0.64	0.006
